# Analysis of reported error in Monte Carlo rendered images

**DOI:** 10.1007/s00371-017-1384-7

**Published:** 2017-05-13

**Authors:** Joss Whittle, Mark W. Jones, Rafał Mantiuk

**Affiliations:** 1 Swansea University, Swansea, UK; 2 Cambridge University, Cambridge, UK

**Keywords:** Image quality assessment, Error metric, Monte Carlo rendering

## Abstract

Evaluating image quality in Monte Carlo rendered images is an important aspect of the rendering process as we often need to determine the relative quality between images computed using different algorithms and with varying amounts of computation. The use of a gold-standard, reference image, or ground truth is a common method to provide a baseline with which to compare experimental results. We show that if not chosen carefully, the quality of reference images used for image quality assessment can skew results leading to significant misreporting of error. We present an analysis of error in Monte Carlo rendered images and discuss practices to avoid or be aware of when designing an experiment.

**Electronic supplementary material:**

The online version of this article (doi:10.1007/s00371-017-1384-7) contains supplementary material, which is available to authorized users.

## Introduction

Monte Carlo rendering algorithms [25] allow for a plethora of photo-realistic and physically based lighting phenomena to be simulated, such as indirect illumination, depth of field, participating media, caustics, and physically based materials. A major problem is slow convergence, and early termination of rendering can leave a large amount of undesirable noise in the images. Many methods have been proposed over the last three decades that attempt to minimize noise using as few samples as possible. These can be roughly classified into path space methods [11, 14, 19, 20, 22, 24, 28, 30, 31, 48] that use extra information available within the renderer to guide sampling in path space and image filtering methods [4, 15, 18, 23, 26, 27, 29, 34, 36, 37, 43, 44] that attempt to reconstruct the GT from a coarse un-converged image.

New methods need to be evaluated relative to existing ones. Often the increase in quality is not clear cut and is dependent on the test scenes used; while a strong improvement can be observed for suitable scenes, it may be that others are ill-suited. This can cause the relative improvement in image quality to be small, though important none the less. In these cases where small improvements in quality are used to justify a method’s performance, the accuracy of these measurements is important.

A commonly accepted methodology for evaluating images is I: to use a known GT which is noise-free; II: that comparisons between the GT and test images use a metric such as mean absolute error (MAE), mean square error (MSE), peak signal-to-noise ratio (PSNR), or more recently Structural Similarity Index (SSIM) [52]; and III: usually equal time and/or equal quality comparisons are reported as results. For these types of metrics to be effective, it is a requirement that the reference image is correct and noise-free.

In this paper, we present analysis of error reported when evaluating Monte Carlo rendered images. We look at the impact of reference image quality on results reported by IQA and highlight practices surrounding sample sets.

## Image quality assessment (IQA)

Thorough analysis of 26 distance metrics applied to image data under varying distortions, spanning from pixel divergence methods such as MSE to those based on pixel correlation, structural features, and spectral measures [2] concluded that MSE most accurately described the level of distortion in images containing additive white noise; while for structural distortions such as blurring or block artefacts measures based on edge similarity or weighted by models of the HVS were more robust. A similar study based on how closely different IQA compare to scores given by human test subjects was conducted [38], with results indicating that the MSE-based metrics can achieve comparable performance to more complex algorithms when images are distorted by additive white noise. From the literature, while there are differing opinions on its effectiveness, image quality metrics based on MSE appear to be most common and trusted when evaluating images corrupted predominantly by additive noise.

Relative quality in error assessment of the MSE and MAE metrics was investigated [55], showing that MSE’s nonlinear weighting with divergence can potentially lead to an exaggerated interpretation of error. Recent work [8] has argued that MSE is in fact preferable over MAE when the error distribution is expected to fit a Gaussian model.

Multi-scale geometric analysis (MGA) works by decomposing image signals into subbands of spatial frequency [17]. In the IQA literature, many MGA methods are used to extract structural information from input images. For the IQA considered in this work, MGA appeared repeatedly in the form of Gaussian and Laplacian pyramids [7], steerable pyramids [42], contrast pyramids [46], wavelet transformations [10], the contourlet transformation [13], and the wavelet-based contourlet transformation [49].

Study of the human visual system (HVS) has led to the creation of models that attempt to describe the likelihood that numerical distortions are actually perceivable by human observers under generalized viewing conditions. These models vary from a simple linear weighting of features in a multi-component error measure [52, 54] to models based on a nonlinear contrast sensitivity function [33] applied at multiple scales in an MGA decomposition.

Universal Quality Index (UQI) [50] splits image comparison into luminance, contrast, and structural components using statistics over the local neighbourhoods of each pixel. SSIM [52] extends this idea by applying a linear weighting to each component using values derived from the HVS. The size of neighbourhood used in SSIM can alter its effectiveness at evaluating image quality; Multi-Scale Structural Similarity Index (MS-SSIM) [54] addresses this by applying SSIM to each level of a Gaussian pyramid decomposition of images. Further discussion of the drawbacks of MSE-based approaches compared to structural measures such as SSIM [51] shows that in many cases the same MSE score can be achieved for distorted images that are given vastly different quality assessments when viewed by human observers. In such cases, measures that consider structural features were significantly more robust and closely matched the assessments of human observers. More recently, an analysis of the mathematical properties of SSIM (and IQA based on it) compared to MSE derivative metrics showed they share several desirable qualities which make them well suited in the areas of parameter optimization and transform domain noise reduction [6].

Information Weighting provides an interesting extension on several existing image metrics by applying a non-uniform weighting scheme to the pooling stage of IQA [53]. An information map is computed at each pixel that represents its relative importance with respect to visually perceivable distortions in the input. This is performed at multiple scales in a Laplacian pyramid decomposition of the input image. The resulting IW-MSE and IW-PSNR metrics perform comparably with several advanced IQA algorithms that take properties of the HVS into account. A third metric that benefits from information content weighting is IW-SSIM which extends the MS-SSIM algorithm making it an IQA that takes multi-scale and HVS information into account during both the distortion and pooling stages.

Visual signal-to-noise ratio (VSNR) applies knowledge of the HVS to determine if image distortions would be noticeable to a human observer [9]. A spatially varying threshold on visible distortion is used to quickly determine if the comparison needs additional analysis which is performed by measuring perceived contrast and global precedence of structures within the images.

Noise quality measure [12] fits input images to a HVS noise model using a contrast pyramid decomposition which has the effect of filtering out distortions the model which is not sensitive to. Conventional SNR can then be applied to the model-fitted images to provide a quality assessment.

Information fidelity criterion (IFC) [40] and visual information fidelity (VIF) [39] apply MGA by decomposing input images via the wavelet transformation. Statistics applied to the wavelet coefficients attempts to capture the mutual structural information between the inputs. By decomposing the images at multiple spatial subbands, the effects of high frequency impulse noise can be directly measured. VIF can be considered a normalized variant on IFC [5].

Recent work has been targeted at quantifying multichannel image distortions that do not present themselves when images are reduced to a single channel. FSIMc which is an extension of Feature Similarity Index (FSIM) [57] considers images in the YIQ colour space [56]. This representation allows for luminance and chrominance features to be extracted and compared independently. Structural Contrast Quality Index (SC-QI) and Structural Contrast Distortion Metric (SC-DM) [3] perform feature extraction in the LMN colour space which has similar properties to YIQ. HDR-VDP-2 (visual difference predictor) [32] takes a different approach to multichannel image analysis by looking at the effects of inter-channel contrast masking in the sRGB colour space. The measure makes a per-pixel prediction on the likelihood a human observer would be able to detect the difference between reference and distorted images and is robust to a wide range of illumination conditions seen in natural images.

In full reference (FR) [38] IQA, input images are compared against a GT image that is known to be correct. We also include two methods categorized as reduced reference (RR) IQA in our analysis. These methods are designed with the assumption that the reference image may contain some distortions, but overall is still representative of the GT. Rather than directly measuring per-pixel deviation, these methods measure the structural similarity of images by using the distribution of features extracted by MGA decomposition. The algorithms considered are based on the contourlet transform [45] and wavelet-based contourlet transform [16], respectively.

New IQA methods are often tested against image databases such as LIVE [41] or TID2013 [35] which couple distorted images with mean opinion scores (MOS) on image quality given by human observers. In our exploration of the literature, we have not found an analysis of how these algorithms (both FR and RR) perform when the reference image being used is the product of an un-converged rendering process, still containing impulse noise. We provide an extensive analysis here.

## Computing error

To compute an error value for a given image, it is compared to a GT that is known to be completely noise-free. In computer graphics, error metrics that operate on single-channel (grayscale) images are most widely used in the literature with more recent research working to create IQA measures that operate on multichannel images. To extend single-channel IQA metrics to multichannel (RGB) images, the luminosity [1] of the RGB values is often used for error evaluation (Eq. 1). In this paper, all single-channel IQA are performed on the luminosity channel of images.

1 $$\begin{aligned} \mathcal {L} = (0.2989 \cdot r) + (0.587 \cdot g) + (0.114 \cdot b) \end{aligned}$$

While IQA measures can use a large variety of methods to compare image similarity, they generally follow a two-stage design pattern. In the first stage, a distortion map is computed by comparing images at each pixel or more generally at a local region around each pixel. Methods can use pixel divergence, structural similarity, statistical models for perceivable difference, or combinations of these and other measures. A secondary pooling stage then consolidates this information to a single representative value which most often takes the form of an average across image space, sometimes weighted further by additional perceptual information based on the HVS.

Other IQA based on natural image statistics leverage decompositions such as the wavelet transformation are more abstract in that image similarity is not compared on a per-pixel basis, but rather on an overall statistical measure of mutual information encoded by the decomposition coefficients.

In our experiment, we chose IQA based on both of the above methodologies and those utilizing a variety of measures on per-pixel distortion to see how these various methods cope under the condition of a degrading and possibly non-representative reference images.

The metrics considered are: (single-channel IQA) MSE, MAE, PSNR, VSNR [9], NQM[12], VIF [39], UQI [50], SSIM [52], MS-SSIM [54], IW-MSE, IW-PSNR, IW-SSIM [53], contourlet [45] and WBCT [16] IQA, IFC [40], FSIM [57]; (multichannel IQA) FSIMc [57], SC-QI [3], SC-DM [3], and HDR-VDP-2 [32].

**Fig. 1:**
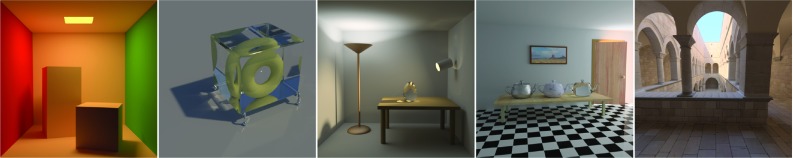
Scenes used for error analysis. From left to right: cornell box, torus, veach bidir, veach door, sponza

## Our experiment

Our experiment is motivated by practices we review in the literature. When examining reference images in some literature, we still see impulse noise, and we wish to explore the effect that reference image quality has on the results reported by IQA. Initially, we performed our analysis on images rendered with a bespoke path tracing software developed for our research. We then validated our experiment using the widely trusted Mitsuba Renderer [21], which are the data we show in this work.

We constructed an experiment where test scenes (Fig. 1) were rendered to increasing numbers of independent samples using each of the rendering algorithms considered. Images were generated on a $2^n$ sample per-pixel (*s.p.p.*) sequence $\mathcal {N} \in \mathbbm {N}:\{2^n | 2 \le n \le \cdots \}$ for each of the test algorithms $\mathcal {A} \in \mathbbm {A}:\{ \textit{PT},~\textit{BDPT},~\textit{PSSMLT}$, $\textit{MLT},~ \textit{Manifold-MLT}$, $\textit{ERPT},~\textit{Manifold-ERPT} \}$ and for each scene $\mathcal {S} \in \mathbbm {S}:\{ \textit{Cornell Box},~\textit{Torus},~\textit{Veach Bidir}$, $\textit{Veach Door},~\textit{Sponza} \}$. This defines a set of images $\mathcal {I}_{\mathcal {S}\mathcal {A}\mathcal {N}}$ where $(\mathcal {S}\mathcal {A}\mathcal {N}) \in (\mathbbm {S}\times \mathbbm {A}\times \mathbbm {N})$ parameterized by scene, rendering algorithm, and sample count with which to perform our analysis. For each scene, we chose a rendering algorithm $\mathcal {A}^G$ to be the reference algorithm based upon its rate of convergence and the lack of structural artefacts at low sample counts. Path tracing was chosen as the reference algorithm for the Cornell Box and Sponza scenes, while the caustic illumination in the Torus, Veach Bidir, and Veach Door scenes was better sampled using bidirectional path tracing.

For each error metric $\mathcal {E} \in \mathbbm {E}:\{ \textit{MSE},~\textit{MAE},~\textit{PSNR}, ~\textit{UQI}$, $\textit{SSIM},~\textit{MS-SSIM},\textit{IW-SSIM}$, $\textit{IW-MSE},~\textit{IW-PSNR}$, $\textit{VSNR}$, $\textit{contourlet},~\textit{WBCT},~\textit{NQM},~\textit{VIF}$, $\textit{IFC},~\textit{FSIM}, \textit{FSIMc}$, $\textit{HDR-}{} { VDP-2}$, $\textit{SC-QI},~\textit{SC-DM} \}$ we compute the true error values to the GT reference image, and we wish to see how degrading the quality of the reference image affects these true error scores. To do this, we select the next highest sampled image as the reference image and recompute the error values. Only images with lower sample counts than the currently selected reference image are computed. By repeating this for all images in the sequence of the reference algorithm, we end up with a triangular matrix for each error metric, algorithm, and scene, where one row represents the true error values, and the remaining rows represent the error values as the reference image is degraded. Formally, for all configurations $\mathcal {C}$ of an error metric, scene, and rendering algorithm we have a lower triangular matrix $\mathcal {M}^\mathcal {C}$ with elements indexed by the number of samples in the test image $\mathcal {N}_j$ and in the reference image $\mathcal {N}_i$, where each element is the error calculated between the reference image $\mathcal {I}_{\mathcal {S}\mathcal {A}^G\mathcal {N}_i}$ and the test image $\mathcal {I}_{\mathcal {S}\mathcal {A}\mathcal {N}_j}$ using an error metric $\mathcal {E}$ (Eq. 2).

2 $$\begin{aligned} \begin{aligned}&\mathcal {M}^\mathcal {C}_{i,j} = \mathcal {E}\left( \mathcal {I}_{\mathcal {S} \mathcal {A}^G\mathcal {N}_i}, \mathcal {I}_{\mathcal {S}\mathcal {A}\mathcal {N}_j}\right) \\&\text {where } i > j \text { and } \mathcal {C} = (\mathcal {E}\mathcal {S} \mathcal {A}) ~~\forall \mathcal {C} \in (\mathbbm {E} \times \mathbbm {S} \times \mathbbm {A}) \end{aligned} \end{aligned}$$

To compare the degraded error values to the true values, we use $\ln \mathcal {Q}$ [47] which measures the difference between an observed and expected value. We chose $\ln \mathcal {Q}$ because, like per cent error, it is a measure of relative change that can be used to compare metrics which operate on different scales, and because it is symmetric between positive and negative values which occur frequently within our data. This is applied to our triangular matrices by taking the natural logarithm of the values in each column divided by the true value in the $\left| \mathbbm {N}\right|$th (bottom) row. This gives a matrix where the bottom row is zeros (referring to the $\ln \mathcal {Q}$ of true values versus themselves) and subsequent rows represent the quality of error evaluations as the reference image is degraded. Formally, from the matrix $\mathcal {M}^\mathcal {C}$ for each configuration in the ensemble we define an equally sized matrix $\mathcal {P}^\mathcal {C}$ with elements defined by Eq. 3.

3 $$\begin{aligned} \begin{aligned} \mathcal {P}^\mathcal {C}_{i,j}&= \ln \left( \frac{\mathcal {M}_{i,j}^\mathcal {C}}{\mathcal {M}_{\left| \mathbbm {N}\right| ,j}^\mathcal {C}}\right) \\ \text {where } i&> j \text { and } \mathcal {C} = (\mathcal {E}\mathcal {S}\mathcal {A}) ~~\forall \mathcal {C} \in (\mathbbm {E} \times \mathbbm {S} \times \mathbbm {A}) \end{aligned} \end{aligned}$$

where $\mathcal {P}^\mathcal {C}$ has positive values and this shows the IQA under test has **overestimated** the amount of error while negative values show the error was **underestimated**.

**Table 1:** $\ln \mathcal {Q}$ of various IQA measures as reference and test image quality are varied

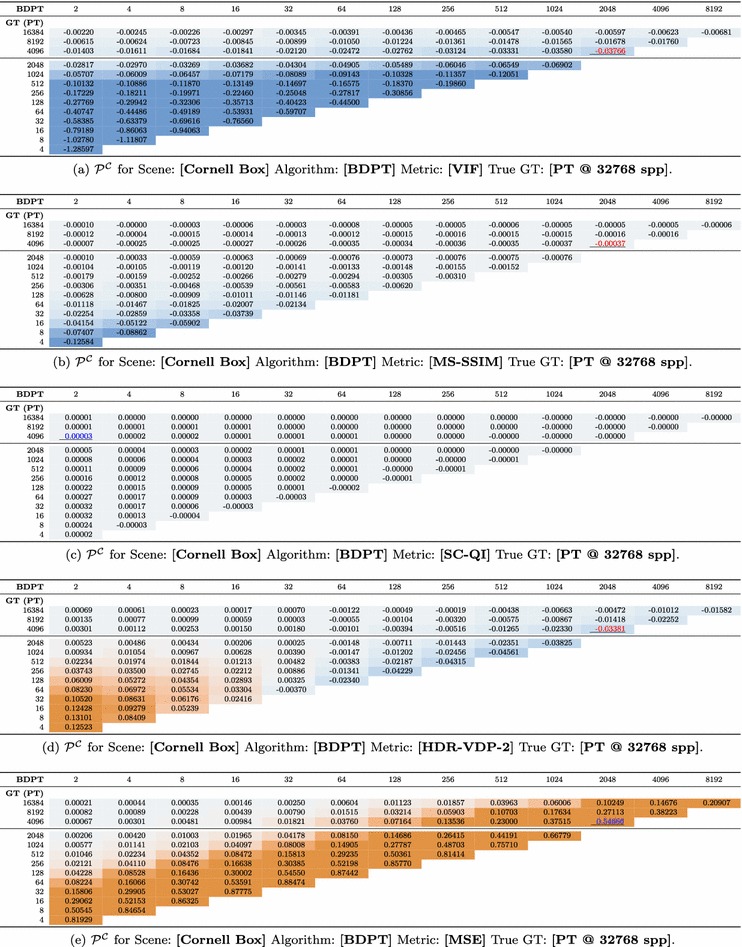	

The vertical axis represents the number of *s.p.p.* in reference images while the horizontal axis denotes the number of *s.p.p.* in test images. Cells are highlighted from underestimation (blue) to overestimation (orange). The horizontal rule between 2048 and 4096 *s.p.p.* separates ground truths that exhibit good visual convergence (above) from sample counts that result in ground truths with visible noise (below). Maximum magnitude for reference images with good visual convergence is shown with a black underline. The matrix has been flipped vertically, and the zero row of reference values versus themselves has been omitted to aid in visualization

## Results

For all scenes, rendering algorithms, and error metrics, there are 735 separate $\mathcal {P}^\mathcal {C}$ matrices in the dataset. We present the full results in supplementary material. Tables 1a–e show $\mathcal {P}^\mathcal {C}$ for the Cornell Box scene rendered with bidirectional path tracing and using error metrics VIF (top), MS-SSIM, SC-QI, HDR-VDP-2, and MSE (bottom). A strong increase in values is visible for MSE, showing that overestimation increases as the number of samples in the reference image decreases to the number of samples in the test image. The increase in misreporting also appears for VIF as a strong underestimation. MS-SSIM and SC-QI also exhibit underestimation but at a significantly lower magnitude. HDR-VDP-2 shows both under- and overestimation at magnitudes comparable to VIF.

**Table 2:** A condensed table of all 735 result tables, showing max $\mathcal {P}^\mathcal {C}$ for all algorithms, scenes, and metrics

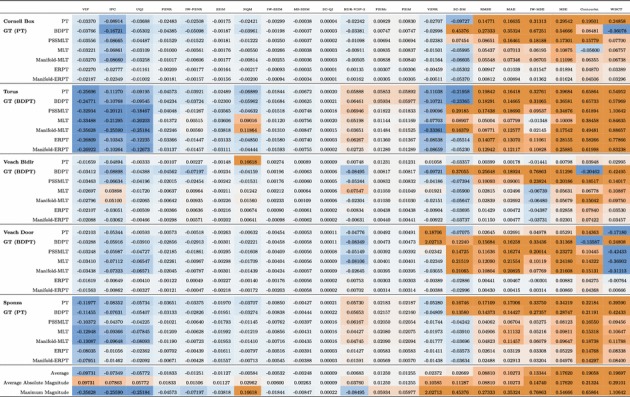	

This shows the observed worst case estimation in each configuration for IQA. These worse cases are from the high sample count ground truths as underlined in Tables 1a–e and in supplementary materials. A value of zero represents an ideal result, showing that error has not been misreported. Ordered (left to right) by column average. Cells are highlighted from underestimation (blue) to overestimation (orange)

To condense this to a manageable set of results, Table 2 displays the maximum magnitude of misreporting within a defined region of each matrix. The maximum magnitude is underlined in each table of results (Table 1a–e and supplementary material). The region is defined for reference images having sufficient samples that they exhibit good visual convergence. Reference images outside this region have lower sample counts and consequently more visible noise. The higher sample reference images are representative of image comparisons that are typically seen in the literature when evaluating rendering algorithms. We signify this region in each table by a horizontal rule. For example, configuration (Cornell Box, BDPT), the maximum magnitude of misreporting in MSE (Table 1e) was $\ln \mathcal {Q}$ of 0.54666, in MS-SSIM (Table 1b) $-0.00037$, and in SC-QI (Table 1c) just $-0.00003$. Columns of Table 2 have been ordered left to right according to the average magnitude of under- or overestimation for each error metric.

Overall, two of the worst performing metrics were the WBCT and contourlet IQA methods which consistently overestimated error, with an average maximum overestimation across all scenes and rendering algorithms of 0.19697 and 0.19058, respectively. These methods are the same measure performed on the different decompositions of the input images which is simply a distance between two coarse histograms over the proportion of visually important coefficients in a multi-scale image decomposition. These are classified as RR IQA methods, meaning that they are designed to work with the assumption that the reference image may contain errors, but are still representative. However, these results show that the measure is highly sensitive to image distortions such as high frequency impulse noise that are prevalent in Monte Carlo rendered images even at high sample counts. The commonly used MSE measure performs just as poorly, consistently overestimating error with an average maximum of 0.1762 overestimation. MAE performs slightly better with an average overestimation of 0.10273 which is to be expected as MSE weights deviations quadratically while MAE weights deviations linearly.

At the other end of the scale, VIF and IFC consistently underestimate error between images with an average maximum of $-0.09731$ and $-0.07349$, respectively. Both methods are based on approximating the two random fields of a GSM noise model. This model assumes that the reference image is correct and does not account of distortions within the reference. Other IQA methods that build off of the GSM model are the information content weighting methods. IW-MSE on average performs slightly better than the standard MSE with an average maximum overestimation of 0.13344; however, due to the poor ability of the GSM to handle noise in the ground truth, this performance is likely due to the addition of multi-scale image analysis rather than because of the GSM noise model. The performance of IW-SSIM which had an average maximum underestimation of $-0.00532$ supports this theory as it is marginally worse than that of MS-SSIM which scored an average maximum underestimation of $-0.00248$. These methods only differ in the use of the GSM noise model. UQI and SSIM which do not perform multi-scale image analysis also support this as they perform worse than MS-SSIM with average maximum underestimations by $-0.05772$ and $-0.01127$, respectively.

Out of the five scenes the Torus scene showed the largest magnitudes of misreported results, likely due to the slow convergence of caustic illumination. The Veach Bidir and Veach Door scenes also feature caustic illumination; however, these converge comparatively quickly compared to the Torus scene and this can be seen in reduced comparative misreporting between the scenes.

## Recommendations and conclusions

It is difficult to find a balance between the desire for a purely numerical distance metric as we are evaluating the quality of a numerical simulation, and the desire to measure only the perceivable noise as observed by the HVS. We argue that a good balance of these features is for a proposed error metric to be monotonic with respect to a simple numerical divergence like MSE such that a reduction in numerical distance always corresponds to a reduction in reported error. Of the IQA considered in this work that were more advanced than a numerical distance MS-SSIM, SC-QI, SC-DM, and NQM were all monotonic with respect to MSE for the types of distortion that are prevalent in Monte Carlo rendered images. The other IQA tested all showed non-monotonicity in the presence of strong impulse noise, primarily from caustic illumination.

IQA which measured per-pixel structural information seemed to be more robust to the effects of impulse noise in the reference image; however, a stronger divide was seen between methods that applied MGA to those that did not. By isolating high frequency noise in one level of a multi-scale decomposition, its effects on image assessment can be bounded or minimized effectively.

Metrics which used perceptual models of the HVS were highly sensitive to the noise in reference images and quickly became unreliable as the quality of the reference was degraded.

Rendering algorithms such as path tracing and bidirectional path tracing, which uniformly sample path space, are better suited to the task of producing reference images than rendering algorithms which use a Markov based random walk such as Metropolis light transport or energy redistribution path tracing. While in certain situations Markov based algorithms exhibit faster convergence than uniform sampling methods, before the simulation has fully converged a uniform method which will have independently distributed error while a Markov algorithm will exhibit noise distributed deterministically with respect to the trajectory the random walk has followed. The result of this is that when we consider the possibility of noise in reference images, noise from Markov processes is more likely to form structural artefacts in the reference, exacerbating misreported error when IQA consider structural features and similarity.

Our recommendations are that MS-SSIM or SC-QI be used for image quality assessments when evaluating images produced by Monte Carlo rendering algorithms as these methods were the most robust when we consider noise in reference images. Reference images should ideally be rendered with uniform sampling methods to avoid the introduction of structural artefacts in IQA. It is important that the reference used is not only visually noise-free, but also that it is of sufficiently higher numerical quality than images tested against it. Reference images should therefore be rendered to at least an order of magnitude higher sample count than test images to minimize the possibility of noise in the reference causing a significant deviation in reported error. And finally that the sample count and method of production of the reference image should be stated clearly to give researchers every confidence in reported results.

## Electronic supplementary material

Below is the link to the electronic supplementary material.

Supplementary material 1 (pdf 2771 KB)
