# Supplementary material for: Analysis of reported error in Monte Carlo rendered images
Source: Vis Comput. 2017 May 13;33(6):705–13. doi: 10.1007/s00371-017-1384-7 (PMC6407833; doi:10.1007/s00371-017-1384-7)
Supplement: Supplementary file 1 — Supplementary material 1 (pdf 2771 KB) [file 371_2017_1384_MOESM1_ESM.pdf]

CGI2017 manuscript No.
(will be inserted by the editor)

Analysis of Reported Error in Monte Carlo Rendered Images Supplementary Material

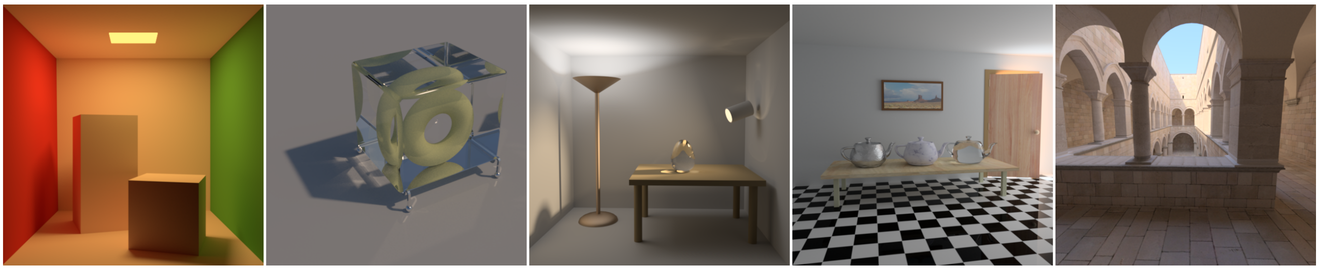

Fig. 1: Scenes used for error analysis. From left to right: Cornell Box, Torus, Veach Bidir, Veach Door, Sponza.

Joss Whittle · Mark Jones · Rafał Mantiuk

Abstract Evaluating image quality in Monte Carlo rendered images is an important aspect of the rendering process as we often need to determine the relative quality between images computed using different algorithms and with varying amounts of computation. The use of a gold-standard, reference image, or ground truth (GT) is a common method to provide a baseline with which to compare experimental results. We show that if not chosen carefully the quality of reference images used for Image Quality Assessment (IQA) can skew results leading to significant misreporting of error. We present an analysis of error in Monte Carlo rendered images and discuss practices to avoid or be aware of when designing an experiment.

Keywords Image Quality Assessment · Error Metric · Monte Carlo Rendering

1 Supplementary Material

Here we provide all 735 $\mathcal{P}^{\mathcal{C}}$ tables computed in our experiment. This paper is organized into sections in the order: Error Metric ▷ Scene ▷ Rendering Algorithm.

The $\mathcal{P}^{\mathcal{C}}$ are computed by taking the natural logarithm of the values in each column divided by the reference value in the $|\mathbb{N}|^{th}$ (bottom) row. This gives a matrix where the bottom row are zeros (referring to the $\ln \mathcal{Q}$ of reference values versus themselves) and subsequent rows represent the quality of error evaluations as the GT is degraded. Formally from the matrix $\mathcal{M}^{\mathcal{C}}$ for each configuration in the ensemble we define an equally sized matrix $\mathcal{P}^{\mathcal{C}}$ with elements defined by equation 1.

$$\mathcal{P}^{\mathcal{C}}_{i,j} = \ln\left(\frac{\mathcal{M}^{\mathcal{C}}_{i,j}}{\mathcal{M}^{\mathcal{C}}_{|\mathbb{N}|,j}}\right) \quad (1)$$
$$\text{where } i > j \text{ and } \mathcal{C} = (\mathcal{E}\mathcal{S}\mathcal{A}) \quad \forall\mathcal{C} \in (\mathbb{E} \times \mathbb{S} \times \mathbb{A})$$

Where $\mathcal{P}^{\mathcal{C}}$ has positive values this shows the IQA under test has **overestimated** the amount of error while negative values show the error was **underestimated**.

Cells in $\mathcal{P}^{\mathcal{C}}$ are highlighted from underestimation (blue) to overestimation (orange). The horizontal rule separates ground truths that exhibit good visual convergence (above) from sample counts that result in ground truths with visible noise (below). Maximum magnitude for GT with good visual convergence shown with a black underline. The matrix has been flipped vertically and the zero row of reference values versus themselves has been omitted to aid in visualization.

Joss Whittle
csjoss@swansea.ac.uk

Mark Jones
m.w.jones@swansea.ac.uk

Rafał Mantiuk
rafal.mantiuk@cl.cam.ac.uk

Contents

1 Supplementary Material 1
2 WBCT 3
2.1 Cornell Box 3
2.2 Torus 4
2.3 Veach Bidir 5

2.4 Veach Door . . . 6
2.5 Sponza . . . 7
3 Contourlet . . . 8
3.1 Cornell Box . . . 8
3.2 Torus . . . 9
3.3 Veach Bidir . . . 10
3.4 Veach Door . . . 11
3.5 Sponza . . . 12
4 MSE . . . 14
4.1 Cornell Box . . . 14
4.2 Torus . . . 15
4.3 Veach Bidir . . . 16
4.4 Veach Door . . . 17
4.5 Sponza . . . 18
5 IW-MSE . . . 19
5.1 Cornell Box . . . 19
5.2 Torus . . . 20
5.3 Veach Bidir . . . 21
5.4 Veach Door . . . 22
5.5 Sponza . . . 23
6 MAE . . . 24
6.1 Cornell Box . . . 24
6.2 Torus . . . 25
6.3 Veach Bidir . . . 26
6.4 Veach Door . . . 27
6.5 Sponza . . . 28
7 RMSE . . . 29
7.1 Cornell Box . . . 29
7.2 Torus . . . 30
7.3 Veach Bidir . . . 31
7.4 Veach Door . . . 32
7.5 Sponza . . . 33
8 SC-DM . . . 34
8.1 Cornell Box . . . 34
8.2 Torus . . . 35
8.3 Veach Bidir . . . 36
8.4 Veach Door . . . 37
8.5 Sponza . . . 38
9 VSNR . . . 39
9.1 Cornell Box . . . 39
9.2 Torus . . . 40
9.3 Veach Bidir . . . 41
9.4 Veach Door . . . 42
9.5 Sponza . . . 43
10 FSIM . . . 44
10.1 Cornell Box . . . 44
10.2 Torus . . . 45
10.3 Veach Bidir . . . 46
10.4 Veach Door . . . 47
10.5 Sponza . . . 48
11 FSIMc . . . 49
11.1 Cornell Box . . . 49
11.2 Torus . . . 50
11.3 Veach Bidir . . . 51
11.4 Veach Door . . . 52
11.5 Sponza . . . 53
12 HDR-VDP-2 . . . 55
12.1 Cornell Box . . . 55
12.2 Torus . . . 56
12.3 Veach Bidir . . . 57
12.4 Veach Door . . . 58
12.5 Sponza . . . 59
13 SC-QI . . . 61
13.1 Cornell Box . . . 61
13.2 Torus . . . 62
13.3 Veach Bidir . . . 63
13.4 Veach Door . . . 64
13.5 Sponza . . . 65
14 MS-SSIM . . . 67
14.1 Cornell Box . . . 67
14.2 Torus . . . 68
14.3 Veach Bidir . . . 69
14.4 Veach Door . . . 70
14.5 Sponza . . . 71
15 IW-SSIM . . . 72
15.1 Cornell Box . . . 72
15.2 Torus . . . 73
15.3 Veach Bidir . . . 74
15.4 Veach Door . . . 75
15.5 Sponza . . . 76
16 NQM . . . 77
16.1 Cornell Box . . . 77
16.2 Torus . . . 78
16.3 Veach Bidir . . . 79
16.4 Veach Door . . . 80
16.5 Sponza . . . 81
17 SSIM . . . 82
17.1 Cornell Box . . . 82
17.2 Torus . . . 83
17.3 Veach Bidir . . . 84
17.4 Veach Door . . . 85
17.5 Sponza . . . 86
18 IW-PSNR . . . 87
18.1 Cornell Box . . . 87
18.2 Torus . . . 88
18.3 Veach Bidir . . . 89
18.4 Veach Door . . . 90
18.5 Sponza . . . 91
19 PSNR . . . 92
19.1 Cornell Box . . . 92
19.2 Torus . . . 93
19.3 Veach Bidir . . . 94
19.4 Veach Door . . . 95
19.5 Sponza . . . 96
20 UQI . . . 97
20.1 Cornell Box . . . 97
20.2 Torus . . . 98
20.3 Veach Bidir . . . 99
20.4 Veach Door . . . 100
20.5 Sponza . . . 101
21 IFC . . . 102
21.1 Cornell Box . . . 102
21.2 Torus . . . 103
21.3 Veach Bidir . . . 104
21.4 Veach Door . . . 105
21.5 Sponza . . . 106
22 VIF . . . 108
22.1 Cornell Box . . . 108
22.2 Torus . . . 109
22.3 Veach Bidir . . . 110
22.4 Veach Door . . . 111
22.5 Sponza . . . 112

2 WBCT

2.1 Cornell Box

PT	2	4	8	16	32	64	128	256	512	1024	2048	4096	8192
**GT (PT)**													
16384	0.00038	0.00045	0.00106	0.00174	0.00228	0.00331	0.00292	0.00000	0.00000	-0.05460	-0.03638	0.06355	0.24858
8192	0.00172	0.00212	0.00397	0.00556	0.00823	0.01089	0.01193	0.02031	0.03824	0.01995	0.08209	0.13526	
4096	0.00405	0.00591	0.00906	0.01304	0.01914	0.02743	0.03372	0.06206	0.11706	0.11083	0.08209		
2048	0.00842	0.01231	0.01835	0.02642	0.03907	0.05355	0.07574	0.12026	0.25249	0.25934			
1024	0.01678	0.02454	0.03679	0.05226	0.07606	0.10683	0.15348	0.26569	0.63541				
512	0.03201	0.04665	0.06768	0.09609	0.13401	0.20616	0.29894	0.57754					
256	0.05958	0.08433	0.11943	0.17256	0.24482	0.39047	0.61792						
128	0.10146	0.14111	0.20132	0.29134	0.43917	0.78636							
64	0.16594	0.22791	0.32971	0.49367	0.84273								
32	0.25707	0.35455	0.52939	0.88785									
16	0.38594	0.55865	0.92133										
8	0.58420	0.92242											
4	0.94797												

(a) $\mathcal{P}^{\mathcal{C}}$ for Scene: [**Cornell Box**] Algorithm: [**PT**] Metric: [**WBCT**] True GT: [**PT @ 32768 spp**].

BDPT	2	4	8	16	32	64	128	256	512	1024	2048	4096	8192
**GT (PT)**													
16384	0.00061	0.00130	0.00213	0.00257	0.00319	0.00571	-0.00300	-0.00788	-0.09087	-0.03379	-0.06355	-0.27405	-0.36078
8192	0.00298	0.00466	0.00742	0.00996	0.01306	0.02124	0.02529	0.02483	-0.06991	0.21655	-0.26126	-0.15362	
4096	0.00696	0.01085	0.01735	0.02426	0.03376	0.05018	0.07203	0.13509	0.14851	0.21655	-0.06355		
2048	0.01393	0.02193	0.03445	0.05057	0.06605	0.09983	0.13265	0.25942	0.18489	-0.03379			
1024	0.02779	0.04272	0.06591	0.09993	0.14106	0.21061	0.30905	0.59003	0.42195				
512	0.05197	0.07977	0.12381	0.18344	0.26481	0.42397	0.78794	0.80657					
256	0.09704	0.14774	0.22469	0.34027	0.52470	1.09355	1.00120						
128	0.16762	0.25575	0.39285	0.62696	1.19407	1.09355							
64	0.28073	0.43151	0.67528	1.25258	1.36458								
32	0.45866	0.71839	1.27626	1.69912									
16	0.73946	1.22042	1.68083										
8	1.34682	1.85581											
4	1.84174												

(b) $\mathcal{P}^{\mathcal{C}}$ for Scene: [**Cornell Box**] Algorithm: [**BDPT**] Metric: [**WBCT**] True GT: [**PT @ 32768 spp**].

PSSMLT	2	4	8	16	32	64	128	256	512	1024	2048	4096	8192
**GT (PT)**													
16384	0.00084	0.00149	0.00221	0.00194	0.00302	0.00208	0.00232	-0.00485	-0.01455	-0.05205	-0.04611	-0.04611	-0.03134
8192	0.00330	0.00497	0.00671	0.00844	0.01182	0.01386	0.01424	0.02021	0.02311	0.00000	0.00000	-0.01599	
4096	0.00772	0.01168	0.01628	0.02035	0.03069	0.03820	0.05081	0.06512	0.07473	0.07700	0.03405		
2048	0.01511	0.02282	0.03284	0.04323	0.05937	0.08553	0.12429	0.11754	0.15820	0.02899			
1024	0.03073	0.04556	0.06436	0.08556	0.12384	0.17938	0.26193	0.25665	0.29920				
512	0.05684	0.08361	0.11622	0.15960	0.23989	0.35214	0.53602	0.48589					
256	0.10361	0.14936	0.20896	0.29286	0.44219	0.72890	1.22733						
128	0.17562	0.25056	0.35681	0.56486	0.98568	1.31195							
64	0.28577	0.40994	0.61156	1.13381	1.46481								
32	0.45093	0.67311	1.21892	1.74916									
16	0.70950	1.15717	1.53367										
8	1.23305	1.77836											
4	1.68549												

(c) $\mathcal{P}^{\mathcal{C}}$ for Scene: [**Cornell Box**] Algorithm: [**PSSMLT**] Metric: [**WBCT**] True GT: [**PT @ 32768 spp**].

MLT	2	4	8	16	32	64	128	256	512	1024	2048	4096	8192
**GT (PT)**													
16384	0.00029	0.00051	0.00068	0.00087	0.00180	0.00239	0.00320	0.00582	0.00752	-0.01197	-0.02068	-0.05129	-0.03472
8192	0.00098	0.00166	0.00250	0.00372	0.00659	0.00796	0.01145	0.01706	0.02328	0.01249	0.00000	0.02277	
4096	0.00261	0.00419	0.00628	0.00897	0.01451	0.02039	0.03141	0.04647	0.06757	0.06343	0.04574		
2048	0.00534	0.00829	0.01234	0.01837	0.02877	0.03987	0.06055	0.08877	0.12693	0.13094			
1024	0.01112	0.01701	0.02488	0.03693	0.05490	0.07967	0.11481	0.17504	0.25012				
512	0.02188	0.03212	0.04644	0.06885	0.10174	0.14877	0.21672	0.31460					
256	0.04163	0.05983	0.08688	0.12397	0.18361	0.27540	0.40686						
128	0.07216	0.10256	0.14744	0.21174	0.31656	0.48739							
64	0.12019	0.16836	0.24370	0.35444	0.54483								
32	0.18988	0.26596	0.38615	0.58890									
16	0.28716	0.41164	0.61124										
8	0.42469	0.62543											
4	0.63809												

(d) $\mathcal{P}^{\mathcal{C}}$ for Scene: [**Cornell Box**] Algorithm: [**MLT**] Metric: [**WBCT**] True GT: [**PT @ 32768 spp**].

M-MLT	2	4	8	16	32	64	128	256	512	1024	2048	4096	8192
**GT (PT)**													
16384	0.00018	0.00045	0.00077	0.00120	0.00171	0.00161	0.00346	0.00277	0.00643	-0.01863	0.00000	-0.04161	-0.05592
8192	0.00111	0.00175	0.00279	0.00393	0.00636	0.00818	0.01331	0.01618	0.02913	0.02505	0.02611	-0.01087	
4096	0.00265	0.00412	0.00650	0.00985	0.01515	0.02005	0.03059	0.04124	0.06738	0.06398	0.04469		
2048	0.00559	0.00864	0.01305	0.01968	0.03010	0.04034	0.05970	0.08361	0.11857	0.12958			
1024	0.01154	0.01737	0.02649	0.03869	0.05976	0.08157	0.12148	0.18264	0.25098				
512	0.02275	0.03326	0.04934	0.07115	0.10475	0.15584	0.22685	0.33981					
256	0.04323	0.06195	0.08905	0.13015	0.18926	0.28444	0.43466						
128	0.07485	0.10533	0.15171	0.22220	0.32871	0.52322							
64	0.12537	0.17370	0.25438	0.37060	0.56985								
32	0.19892	0.27442	0.40310	0.61778									
16	0.30354	0.42387	0.64487										
8	0.44817	0.66045											
4	0.67117												

(e) $\mathcal{P}^{\mathcal{C}}$ for Scene: [**Cornell Box**] Algorithm: [**M-MLT**] Metric: [**WBCT**] True GT: [**PT @ 32768 spp**].

ERPT	2	4	8	16	32	64	128	256	512	1024	2048	4096	8192
**GT (PT)**													
16384	0.00379	0.00383	0.00291	0.00327	0.00336	0.00291	0.00434	0.00333	0.00430	0.00286	0.00234	0.00388	0.00288
8192	0.01360	0.01424	0.01243	0.01192	0.01380	0.01394	0.01282	0.01316	0.01319	0.01071	0.01247	0.01498	
4096	0.03203	0.03069	0.03239	0.03263	0.03141	0.03117	0.02982	0.03219	0.03339	0.03069	0.03389		
2048	0.06489	0.06171	0.06491	0.06510	0.06335	0.06340	0.06157	0.06073	0.06477	0.06300			
1024	0.13234	0.12344	0.13252	0.12898	0.12422	0.12848	0.12903	0.12181	0.12564				
512	0.25350	0.23267	0.23643	0.23457	0.23313	0.24129	0.23691	0.24217					
256	0.45762	0.45240	0.45576	0.44414	0.45815	0.44624	0.46109						
128	0.93500	0.93312	0.97588	0.96792	0.96229	0.94502							
64	1.43019	1.71853	1.57992	1.72321	1.52543								
32	1.43019	1.38556	1.30649	1.31451									
16	1.34810	1.34621	1.34287										
8	1.24641	1.18737											
4	1.13862												

(f) $\mathcal{P}^{\mathcal{C}}$ for Scene: [**Cornell Box**] Algorithm: [**ERPT**] Metric: [**WBCT**] True GT: [**PT @ 32768 spp**].

M-ERPT	2	4	8	16	32	64	128	256	512	1024	2048	4096	8192
**GT (PT)**													
16384	0.00321	0.00314	0.00401	0.00357	0.00368	0.00316	0.00282	0.00271	0.00276	0.00314	0.00227	0.00360	0.00493
8192	0.01318	0.01195	0.01369	0.01230	0.01370	0.01248	0.01353	0.01200	0.01272	0.01337	0.01212	0.01385	
4096	0.03099	0.03031	0.03148	0.03104	0.03214	0.03046	0.03296	0.02941	0.03053	0.03240	0.03023		
2048	0.06201	0.06299	0.06520	0.06599	0.06406	0.06148	0.06261	0.06087	0.06032	0.06421			
1024	0.12370	0.12363	0.12997	0.13289	0.12745	0.11949	0.13062	0.12438	0.12916				
512	0.23231	0.23501	0.23723	0.24086	0.23457	0.22361	0.23133	0.23278					
256	0.44735	0.44209	0.45048	0.46283	0.45654	0.43515	0.45522						
128	0.89986	0.93084	0.93306	0.94684	1.02072	0.94417							
64	1.53427	1.81238	1.44247	1.44203	1.65425								
32	1.35411	1.32178	1.36038	1.32355									
16	1.31772	1.32178	1.36038										
8	1.19526	1.19932											
4	1.14462												

(g) $\mathcal{P}^{\mathcal{C}}$ for Scene: [**Cornell Box**] Algorithm: [**M-ERPT**] Metric: [**WBCT**] True GT: [**PT @ 32768 spp**].

Table 1

2.2 Torus

PT	2	4	8	16	32	64	128	256	512	1024	2048	4096	8192	16384	32768	65536	131072
GT (BDPT)																	
262144	0.00829	0.01471	0.02603	0.03898	0.02606	0.00409	0.00315	0.00575	0.00553	0.00869	0.01144	0.01637	0.02032	0.03515	0.05914	0.11738	0.18016
131072	0.02426	0.04238	0.07358	0.11216	0.06647	0.02121	0.01288	0.01653	0.01906	0.02395	0.03348	0.04740	0.06421	0.10666	0.18873	0.41310	
65536	0.05369	0.09223	0.16876	0.24328	0.10788	0.03965	0.02570	0.03385	0.03959	0.04740	0.06856	0.09610	0.14793	0.24764	0.50584		
32768	0.10868	0.19626	0.38312	0.48592	0.17406	0.06667	0.04598	0.06326	0.07527	0.09472	0.13058	0.20120	0.32932	0.54952			
16384	0.20128	0.37142	0.78155	0.70628	0.22474	0.09904	0.08152	0.10663	0.12921	0.16251	0.22996	0.33524	0.62482				
8192	0.34951	0.70645	1.14778	0.72836	0.29990	0.15825	0.13481	0.16677	0.20358	0.26819	0.38848	0.62237					
4096	0.61562	1.51715	1.07322	0.84827	0.39460	0.23810	0.22546	0.25895	0.31796	0.41956	0.66942						
2048	1.26313	1.17056	0.90946	0.89831	0.55924	0.39933	0.36042	0.40373	0.51945	0.74089							
1024	1.13880	0.92712	0.75243	0.86425	0.82922	0.66755	0.58724	0.65361	0.86325								
512	0.86871	0.74844	0.59670	0.56717	0.70666	0.77148	1.01137	1.15164									
256	0.79911	0.70257	0.56497	0.50290	0.55924	0.73883	1.20773										
128	0.82054	0.67329	0.52973	0.47510	0.52103	0.86699											
64	0.93229	0.69123	0.53985	0.49148	0.66396												
32	0.94537	0.80822	0.60521	0.62118													
16	0.99582	1.20321	0.89859														
8	1.21702	1.59287															
4	1.63011																

(a) $\mathcal{P}^{\mathcal{C}}$ for Scene: [**Torus**] Algorithm: [**PT**] Metric: [**WBCT**] True GT: [**BDPT @ 524288 spp**].

BDPT	2	4	8	16	32	64	128	256	512	1024	2048	4096	8192	16384	32768	65536	131072
GT (BDPT)																	
262144	0.00767	0.01324	0.02419	0.03487	0.02569	0.00783	0.00474	0.00623	0.00645	0.00771	0.01068	0.01390	0.02349	0.03044	0.05258	0.10834	0.17051
131072	0.02239	0.03774	0.06729	0.09871	0.07026	0.02313	0.01205	0.01504	0.01951	0.02210	0.03207	0.04389	0.06538	0.09564	0.17969	0.33247	
65536	0.04899	0.08548	0.14953	0.20261	0.13152	0.04083	0.02570	0.03129	0.03894	0.04983	0.06721	0.09454	0.13521	0.23426	0.41566		
32768	0.10046	0.17802	0.31666	0.38615	0.19087	0.06723	0.04988	0.06057	0.07906	0.09869	0.13069	0.18926	0.28922	0.57969			
16384	0.18304	0.33958	0.58544	0.55416	0.21505	0.10197	0.09195	0.09989	0.13052	0.16437	0.23174	0.33691	0.59118				
8192	0.31821	0.63282	1.04941	0.66657	0.25218	0.16994	0.15104	0.16648	0.20403	0.26860	0.38285	0.67205					
4096	0.53442	1.31182	1.31657	0.82238	0.36566	0.25322	0.24926	0.25872	0.30897	0.41636	0.66378						
2048	0.95848	1.23369	1.11481	0.90964	0.52022	0.41852	0.40496	0.40396	0.49644	0.75703							
1024	1.56630	0.94829	0.92278	0.90964	0.66755	0.68992	0.66016	0.63585	0.80927								
512	1.01815	0.76153	0.70242	0.61975	0.66755	0.80231	1.24412	1.12896									
256	0.90238	0.71566	0.64720	0.54253	0.63048	0.83636	1.32621										
128	0.99449	0.68988	0.62089	0.52586	0.65478	1.01045											
64	1.07111	0.72348	0.63375	0.55416	0.80705												
32	1.00219	0.84415	0.71363	0.72093													
16	0.81386	1.27090	1.15201														
8	0.93231	1.64872															
4	1.36071																

(b) $\mathcal{P}^{\mathcal{C}}$ for Scene: [**Torus**] Algorithm: [**BDPT**] Metric: [**WBCT**] True GT: [**BDPT @ 524288 spp**].

PSSMLT	2	4	8	16	32	64	128	256	512	1024	2048	4096	8192	16384
GT (BDPT)														
262144	0.00815	0.01137	0.01389	0.01573	0.01347	0.01019	0.01109	0.01194	0.01347	0.02108	0.02461	0.03074	0.06299	0.08017
131072	0.02442	0.03196	0.03856	0.04379	0.03316	0.03133	0.03096	0.03504	0.04555	0.05925	0.07961	0.09982	0.18836	0.31141
65536	0.05190	0.06972	0.08529	0.08278	0.06975	0.06226	0.06226	0.06978	0.09467	0.12604	0.17043	0.26607	0.45834	0.78541
32768	0.10022	0.13650	0.15684	0.14758	0.12505	0.11444	0.11158	0.13816	0.18331	0.24994	0.36161	0.71547	1.10642	0.18035
16384	0.17027	0.23236	0.24409	0.22127	0.17985	0.16811	0.19235	0.23866	0.33447	0.46627	0.79998	0.79868	0.50136	
8192	0.27475	0.36223	0.36079	0.32419	0.27596	0.26487	0.30560	0.40023	0.60531	1.16249	0.82324	0.43334		
4096	0.42999	0.54400	0.51107	0.44484	0.38810	0.40192	0.48969	0.65645	1.27136	0.93825	0.51864			
2048	0.65886	0.84921	0.75462	0.63795	0.61080	0.64589	0.89438	1.04634	0.79094	0.55777				
1024	1.08779	1.06203	0.93108	0.85263	0.88359	0.86589	0.96564	0.76421	0.53239					
512	1.01375	0.85946	0.82653	0.74445	0.77903	0.75014	0.68657	0.49148						
256	0.97541	0.81101	0.78366	0.76586	0.77903	0.72872	0.57662							
128	1.10446	0.82958	0.81532	0.85263	0.81489	0.75014								
64	1.26988	0.99663	1.03369	1.04720	1.24553									
32	0.87082	1.39496	1.45821	1.40875										
16	0.61163	0.82017	0.72783											
8	0.61967	0.75297												
4	0.76342													

(c) $\mathcal{P}^{\mathcal{C}}$ for Scene: [**Torus**] Algorithm: [**PSSMLT**] Metric: [**WBCT**] True GT: [**BDPT @ 524288 spp**].

MLT	2	4	8	16	32	64	128	256	512	1024	2048	4096	8192	16384
GT (BDPT)														
262144	0.00388	0.00584	0.00894	0.00966	0.01786	0.01607	0.02265	0.02109	0.02082	0.00587	0.04371	0.06070	0.05443	0.07831
131072	0.01157	0.01700	0.02578	0.03109	0.04998	0.04949	0.06641	0.07561	0.07775	0.10193	0.14250	0.19182	0.19542	0.29158
65536	0.02245	0.03486	0.05190	0.06787	0.10150	0.10913	0.13290	0.16100	0.18963	0.22546	0.27967	0.34204	0.41458	0.51008
32768	0.03905	0.06521	0.09874	0.13140	0.19953	0.20228	0.25403	0.30922	0.37213	0.51886	0.60783	0.70359	0.84635	0.10780
16384	0.06119	0.10516	0.16366	0.22267	0.33243	0.32458	0.44595	0.51184	0.60641	0.56950	0.49311	0.47310	0.41458	
8192	0.09492	0.16020	0.26092	0.35458	0.54942	0.51204	0.60534	0.56777	0.48883	0.39541	0.32768	0.26004		
4096	0.14272	0.24563	0.39868	0.55124	0.90491	0.80018	0.70538	0.65134	0.54343	0.36136	0.26490			
2048	0.20492	0.35894	0.58630	0.88014	0.93896	0.72890	0.61811	0.52507	0.35978	0.17264				
1024	0.26970	0.49441	0.85889	0.86779	0.80487	0.65819	0.51667	0.36452	0.16115					
512	0.33223	0.63880	0.85889	0.76321	0.54942	0.48532	0.31096	0.17487						
256	0.36915	0.70009	0.85889	0.74558	0.47597	0.42174	0.24974							
128	0.32761	0.63380	0.89055	0.71287	0.46680	0.40154								
64	0.28574	0.55191	0.95038	0.83307	0.49521									
32	0.23548	0.42020	0.74776	1.01423										
16	0.19195	0.32994	0.53865											
8	0.19964	0.34658												
4	0.25360													

(d) $\mathcal{P}^{\mathcal{C}}$ for Scene: [**Torus**] Algorithm: [**MLT**] Metric: [**WBCT**] True GT: [**BDPT @ 524288 spp**].

M-MLT	2	4	8	16	32	64	128	256	512	1024	2048	4096	8192	16384
GT (BDPT)														
262144	0.00377	0.00847	0.01153	0.01138	0.01572	0.02261	0.02823	0.03382	0.03601	0.03946	0.06400	0.05995	0.09479	0.00000
131072	0.01332	0.02363	0.03396	0.03330	0.04612	0.06341	0.07783	0.10409	0.16391	0.15820	0.21555	0.27837	0.36822	0.21851
65536	0.02618	0.04794	0.06936	0.07030	0.09773	0.12478	0.15834	0.23957	0.34507	0.49068	0.53066	0.35409	0.21326	-0.11848
32768	0.04880	0.08733	0.13027	0.13114	0.18623	0.24574	0.30467	0.50361	0.80680	0.88657	0.53066	0.12986	-0.05373	-0.37781
16384	0.07736	0.14061	0.20882	0.21160	0.30374	0.39398	0.52580	0.82924	0.51916	0.54783	0.28183	-0.05223	-0.21915	
8192	0.12240	0.21501	0.32130	0.34251	0.47021	0.62951	0.76766	0.65516	0.31102	0.33477	0.10834	-0.13950		
4096	0.18672	0.31600	0.49003	0.50132	0.74962	0.83488	0.91570	0.51783	0.18669	0.18250	-0.01157			
2048	0.26693	0.44374	0.72930	0.77442	0.92234	0.85227	0.70226	0.32935	0.05967	0.04794				
1024	0.35953	0.60455	0.97877	0.88793	0.86429	0.68233	0.48226	0.18561	-0.06731					
512	0.45585	0.80646	0.88042	0.81160	0.66332	0.47482	0.30937	0.06495						
256	0.51335	0.91483	0.84421	0.80193	0.57697	0.40852	0.26144							
128	0.47156	0.78579	0.88042	0.79251	0.56000	0.37563								
64	0.41616	0.67931	1.06282	1.01698	0.61396									
32	0.32961	0.51742	0.91029	1.03437										
16	0.25148	0.41719	0.67412											
8	0.25838	0.43201												
4	0.30460													

(e) $\mathcal{P}^{\mathcal{C}}$ for Scene: [**Torus**] Algorithm: [**M-MLT**] Metric: [**WBCT**] True GT: [**BDPT @ 524288 spp**].

ERPT	2	4	8	16	32	64	128	256	512	1024	2048	4096	8192	16384	32768	65536	131072
GT (BDPT)																	
262144	0.03670	0.03665	0.02991	0.03419	0.03071	0.03194	0.02654	0.03231	0.02834	0.01780	0.02863	0.03447	0.03769	0.04144	0.04231	0.04276	0.03553
131072	0.12871	0.11262	0.08684	0.10888	0.09358	0.09299	0.08069	0.07944	0.07667	0.07754	0.10359	0.12015	0.12154	0.11259	0.11130	0.13314	
65536	0.29181	0.24537	0.19638	0.22090	0.19182	0.19579	0.17534	0.18185	0.17839	0.19937	0.23498	0.25463	0.29062	0.27880	0.30886		
32768	0.72432	0.52844	0.42200	0.46762	0.39225	0.37728	0.36498	0.37635	0.39106	0.43694	0.52527	0.58814	0.73587	0.77866			
16384	0.86363	0.82459	0.69120	1.12138	0.76386	0.65981	0.66417	0.69010	0.75941	0.96834	0.98020	1.05321	0.93223				
8192	0.53547	0.70095	0.65563	0.69685	0.74289	0.77199	0.71877	0.88158	0.92396	0.82904	0.72087	0.53009					
4096	0.43092	0.57279	0.77320	0.57948	0.78592	0.89563	0.73873	0.74470	0.65680	0.54721	0.51371						
2048	0.31547	0.34570	0.47045	0.37424	0.47292	0.50184	0.47899	0.44754	0.40561	0.37445							
1024	0.18849	0.21600	0.32120	0.24180	0.32084	0.34205	0.28615	0.30066	0.28346								
512	0.05706	0.08978	0.16281	0.11937	0.16770	0.19579	0.15970	0.16368									
256	0.03023	0.05865	0.12571	0.09221	0.12485	0.15005	0.12068										
128	-0.00566	0.02483	0.10559	0.05877	0.11191	0.12643											
64	0.00872	0.04276	0.11547	0.07046	0.12485												
32	0.07150	0.10097	0.18333	0.12296													
16	0.28428	0.35418	0.46021														
8	0.49278	0.55743															
4	0.80264																

(f) $\mathcal{P}^{\mathcal{C}}$ for Scene: [**Torus**] Algorithm: [**ERPT**] Metric: [**WBCT**] True GT: [**BDPT @ 524288 spp**].

M-ERPT	2	4	8	16	32	64	128	256	512	1024	2048	4096	8192	16384	32768	65536	131072
GT (BDPT)																	
262144	0.03248	0.03231	0.03283	0.02613	0.04022	0.03356	0.03846	0.03837	0.03179	0.03984	0.03694	0.04511	0.04144	0.03788	0.04444	0.04337	0.04603
131072	0.10976	0.10377	0.11152	0.10472	0.11329	0.12391	0.13775	0.10887	0.11644	0.11707	0.11880	0.13289	0.13255	0.12705	0.13671	0.14646	
65536	0.22222	0.23533	0.23606	0.22019	0.25845	0.27464	0.26918	0.25959	0.25413	0.29892	0.29559	0.29804	0.31403	0.27523	0.32156		
32768	0.45739	0.52633	0.52252	0.46373	0.63678	0.64679	0.61617	0.66642	0.65994	0.69455	0.74084	0.76656	0.83238	0.70985			
16384	0.86392	0.85553	1.03660	0.93683	1.00377	0.86594	1.19466	1.03869	1.13906	0.92010	1.01293	0.93966	0.89337				
8192	0.74699	0.67344	0.70367	0.78371	0.70805	0.68236	0.75969	0.63237	0.67733	0.57718	0.59062	0.50901					
4096	0.72704	0.72567	0.60928	0.72377	0.52440	0.53009	0.51162	0.45361	0.46451	0.44453	0.43809						
2048	0.50867	0.47505	0.41019	0.50302	0.34689	0.33304	0.35762	0.31460	0.32551	0.30553							
1024	0.35165	0.33003	0.29684	0.33435	0.23719	0.23571	0.25309	0.21290	0.22380								
512	0.18765	0.18946	0.16699	0.18756	0.11666	0.11341	0.13425	0.10887									
256	0.15678	0.14994	0.13624	0.15828	0.08770	0.08411	0.10149										
128	0.11919	0.11831	0.10568	0.12552	0.05876	0.05486											
64	0.13565	0.13053	0.11152	0.13494	0.06712												
32	0.20443	0.20127	0.17062	0.20752													
16	0.48726	0.45647	0.37973														
8	0.70801	0.70749															
4	0.95259																

(g) $\mathcal{P}^{\mathcal{C}}$ for Scene: [**Torus**] Algorithm: [**M-ERPT**] Metric: [**WBCT**] True GT: [**BDPT @ 524288 spp**].

Table 2

2.3 Veach Bidir

PT	2	4	8	16	32	64	128	256	512	1024	2048	4096	8192	16384	32768	65536	131072
GT (BDPT)																	
262144	-0.00010	-0.00015	0.00016	0.00030	0.00020	0.00022	0.00067	0.00080	0.00085	0.00127	0.00147	0.00205	0.00176	0.00127	0.00099	0.00189	-0.00435
131072	0.00005	0.00010	0.00033	0.00059	0.00046	0.00066	0.00092	0.00120	0.00135	0.00191	0.00232	0.00352	0.00309	0.00447	0.00601	0.01154	
65536	0.00029	0.00041	0.00087	0.00125	0.00131	0.00178	0.00236	0.00322	0.00407	0.00531	0.00641	0.00712	0.00898	0.01238	0.01218		
32768	0.00034	0.00067	0.00114	0.00167	0.00184	0.00267	0.00347	0.00444	0.00520	0.00728	0.00904	0.01047	0.01175	0.01372			
16384	0.00039	0.00092	0.00131	0.00215	0.00250	0.00327	0.00432	0.00547	0.00671	0.00878	0.01149	0.01389	0.01504				
8192	0.00108	0.00195	0.00296	0.00451	0.00559	0.00747	0.00975	0.01209	0.01518	0.01945	0.02426	0.02995					
4096	0.00098	0.00190	0.00285	0.00408	0.00505	0.00677	0.00895	0.01102	0.01399	0.01733	0.02187						
2048	0.00405	0.00599	0.00912	0.01292	0.01663	0.02155	0.02806	0.03493	0.04407	0.05597							
1024	0.00511	0.00749	0.01104	0.01559	0.02002	0.02602	0.03367	0.04228	0.05274								
512	0.00678	0.00993	0.01489	0.02115	0.02705	0.03519	0.04486	0.05679									
256	0.01051	0.01533	0.02209	0.03117	0.03984	0.05179	0.06604										
128	0.01629	0.02344	0.03295	0.04612	0.06046	0.07770											
64	0.02745	0.03820	0.05335	0.07435	0.09703												
32	0.04455	0.06263	0.08627	0.11902													
16	0.07207	0.10209	0.14243														
8	0.11142	0.15945															
4	0.17170																

(a) $\mathcal{P}^{\mathcal{C}}$ for Scene: [**Veach Bidir**] Algorithm: [**PT**] Metric: [**WBCT**] True GT: [**BDPT @ 524288 spp**].

BDPT	2	4	8	16	32	64	128	256	512	1024	2048	4096	8192	16384	32768	65536	131072
GT (BDPT)																	
262144	0.00040	0.00058	0.00135	0.00126	0.00312	0.00439	0.00276	-0.03438	-0.06628	-0.26126	-0.07171	-0.07171	-0.34304	-0.20717	-0.17687	-0.41826	-0.20717
131072	0.00073	0.00100	0.00180	0.00198	0.00417	0.00707	0.01993	0.00000	0.02459	-0.06355	0.17687	0.17687	-0.13587	0.13587	0.11220	-0.17018	
65536	0.00167	0.00268	0.00432	0.00621	0.01169	0.01813	0.03511	0.03821	0.05064	-0.06355	0.00000	0.17687	-0.34304	0.13587	-0.09496		
32768	0.00234	0.00387	0.00605	0.00921	0.01576	0.02681	0.05119	0.04845	0.10780	-0.06355	0.17687	0.42495	-0.13587	0.30606			
16384	0.00301	0.00506	0.00781	0.01226	0.01915	0.03178	0.06131	0.05900	0.10780	-0.12044	-0.07171	0.17687	-0.24808				
8192	0.00678	0.01066	0.01764	0.02693	0.04475	0.07488	0.12667	0.11693	0.33829	0.00000	0.28907	0.17687					
4096	0.00616	0.00986	0.01614	0.02422	0.04130	0.06887	0.12667	0.12970	0.24883	0.15362	0.17687						
2048	0.01997	0.03159	0.05067	0.08243	0.13723	0.22393	0.36105	0.39906	0.58179	0.00000							
1024	0.02420	0.03846	0.06076	0.09987	0.16917	0.28439	0.50398	0.71276	0.87087								
512	0.03236	0.05211	0.08225	0.13596	0.22878	0.37790	0.66924	0.76412									
256	0.04901	0.07918	0.12335	0.20624	0.35441	0.58675	0.98435										
128	0.07442	0.11965	0.19145	0.31956	0.56067	0.98830											
64	0.12313	0.19848	0.32128	0.55987	1.05743												
32	0.20356	0.33458	0.57578	1.05639													
16	0.35443	0.60501	1.14631														
8	0.61392	1.14444															
4	1.13550																

(b) $\mathcal{P}^{\mathcal{C}}$ for Scene: [**Veach Bidir**] Algorithm: [**BDPT**] Metric: [**WBCT**] True GT: [**BDPT @ 524288 spp**].

PSSMLT	2	4	8	16	32	64	128	256	512	1024	2048	4096	8192	16384
GT (BDPT)														
262144	0.00034	0.00050	0.00099	0.00115	0.00283	0.00410	0.00442	-0.00999	-0.00509	-0.01795	-0.00675	-0.00675	-0.00647	-0.02112
131072	0.00068	0.00091	0.00155	0.00230	0.00455	0.00708	0.01045	0.00341	0.01581	0.00622	0.01393	0.02112	0.01335	0.00000
65536	0.00164	0.00259	0.00390	0.00564	0.00952	0.01819	0.02139	0.01391	0.02682	0.01904	0.01393	0.02848	0.03448	0.02258
32768	0.00240	0.00343	0.00537	0.00802	0.01341	0.02529	0.03284	0.03232	0.05009	0.01256	0.01393	0.05159	0.04936	0.03854
16384	0.00288	0.00445	0.00709	0.01026	0.01707	0.02926	0.04310	0.04003	0.06876	0.03928	0.03601	0.05967	0.07302	
8192	0.00625	0.01009	0.01584	0.02397	0.03899	0.06178	0.09327	0.08657	0.14017	0.08398	0.09405	0.07642		
4096	0.00568	0.00903	0.01451	0.02192	0.03658	0.05638	0.08281	0.08657	0.12459	0.08398	0.09405			
2048	0.01836	0.02846	0.04549	0.07000	0.11168	0.18007	0.24496	0.26070	0.33926	0.26936				
1024	0.02249	0.03413	0.05432	0.08480	0.13647	0.22057	0.31708	0.36216	0.45183					
512	0.02969	0.04569	0.07370	0.11482	0.18621	0.30432	0.44665	0.45979						
256	0.04409	0.06842	0.10908	0.17329	0.28435	0.48756	0.67621							
128	0.06634	0.10463	0.16507	0.27227	0.45440	0.80301								
64	0.10791	0.17084	0.27769	0.46862	0.84309									
32	0.17781	0.28525	0.48575	0.86131										
16	0.29857	0.50907	0.91210											
8	0.50056	0.91371												
4	0.91601													

(c) $\mathcal{P}^{\mathcal{C}}$ for Scene: [**Veach Bidir**] Algorithm: [**PSSMLT**] Metric: [**WBCT**] True GT: [**BDPT @ 524288 spp**].

MLT	2	4	8	16	32	64	128	256	512	1024	2048	4096	8192	16384
GT (BDPT)														
262144	-0.00011	0.00000	0.00019	0.00046	0.00059	0.00124	0.00228	0.00394	0.00294	0.00474	-0.00428	0.00000	-0.01983	-0.02912
131072	0.00005	0.00029	0.00045	0.00077	0.00108	0.00194	0.00343	0.00572	0.00693	0.01203	0.00876	0.01942	0.00689	0.00000
65536	0.00038	0.00070	0.00136	0.00201	0.00305	0.00517	0.00741	0.01162	0.01827	0.03518	0.03188	0.02617	0.02112	0.01558
32768	0.00071	0.00093	0.00176	0.00280	0.00435	0.00702	0.01026	0.01722	0.02690	0.04337	0.03673	0.03306	0.02112	0.01558
16384	0.00077	0.00134	0.00221	0.00366	0.00565	0.00933	0.01390	0.02009	0.03360	0.05760	0.04666	0.04010	0.05159	
8192	0.00182	0.00304	0.00500	0.00830	0.01268	0.02016	0.03114	0.04791	0.07171	0.10192	0.08423	0.06988		
4096	0.00171	0.00287	0.00466	0.00765	0.01163	0.01831	0.02766	0.04354	0.06773	0.10887	0.08423			
2048	0.00604	0.00915	0.01459	0.02377	0.03596	0.05682	0.08986	0.13386	0.20824	0.28779				
1024	0.00741	0.01138	0.01798	0.02860	0.04334	0.06799	0.10817	0.16627	0.26239					
512	0.00997	0.01519	0.02386	0.03866	0.05848	0.09085	0.14302	0.22219						
256	0.01510	0.02311	0.03625	0.05733	0.08725	0.13723	0.22089							
128	0.02257	0.03473	0.05458	0.08498	0.12929	0.20898								
64	0.03705	0.05685	0.08804	0.13657	0.21044									
32	0.05972	0.09167	0.14132	0.22140										
16	0.09639	0.14798	0.23180											
8	0.14607	0.23005												
4	0.22279													

(d) $\mathcal{P}^{\mathcal{C}}$ for Scene: [**Veach Bidir**] Algorithm: [**MLT**] Metric: [**WBCT**] True GT: [**BDPT @ 524288 spp**].

M-MLT	2	4	8	16	32	64	128	256	512	1024	2048	4096	8192	16384
GT (BDPT)														
262144	0.00000	0.00018	0.00033	0.00039	0.00080	0.00143	0.00141	0.00229	0.00197	0.00000	0.00000	-0.00556	-0.01282	-0.02978
131072	0.00022	0.00035	0.00072	0.00063	0.00131	0.00186	0.00307	0.00415	0.00797	0.00696	0.01345	0.01142	0.00661	-0.00770
65536	0.00049	0.00094	0.00138	0.00205	0.00334	0.00506	0.00789	0.01175	0.01942	0.02396	0.01807	0.02937	0.02024	0.01596
32768	0.00066	0.00118	0.00205	0.00276	0.00467	0.00684	0.01085	0.01616	0.02483	0.03946	0.04231	0.02327	0.03448	0.02423
16384	0.00088	0.00159	0.00238	0.00364	0.00613	0.00893	0.01411	0.02168	0.03149	0.04757	0.05258	0.05501	0.04936	
8192	0.00210	0.00351	0.00561	0.00828	0.01315	0.02045	0.03193	0.04718	0.07207	0.09221	0.09151	0.09024		
4096	0.00193	0.00333	0.00514	0.00745	0.01196	0.01805	0.02916	0.04374	0.06544	0.08582	0.09750			
2048	0.00617	0.01028	0.01518	0.02391	0.03820	0.05824	0.09307	0.14514	0.21196	0.22599				
1024	0.00761	0.01250	0.01848	0.02912	0.04602	0.06990	0.11057	0.17893	0.26141					
512	0.01017	0.01672	0.02516	0.03917	0.06097	0.09363	0.14959	0.23732						
256	0.01551	0.02504	0.03743	0.05845	0.09017	0.14199	0.22416							
128	0.02341	0.03698	0.05562	0.08801	0.13606	0.21692								
64	0.03808	0.05934	0.08972	0.14297	0.22120									
32	0.06156	0.09574	0.14565	0.23193										
16	0.09999	0.15447	0.23759											
8	0.15231	0.24088												
4	0.23363													

(e) $\mathcal{P}^{\mathcal{C}}$ for Scene: [**Veach Bidir**] Algorithm: [**M-MLT**] Metric: [**WBCT**] True GT: [**BDPT @ 524288 spp**].

ERPT	2	4	8	16	32	64	128	256	512	1024	2048	4096	8192	16384	32768	65536	131072
GT (BDPT)																	
262144	0.00189	0.00162	0.00240	0.00270	0.00243	0.00236	0.00191	0.00162	0.00244	0.00187	0.00186	0.00187	0.00160	0.00160	0.00266	0.00184	0.00205
131072	0.00325	0.00270	0.00348	0.00407	0.00380	0.00422	0.00356	0.00297	0.00381	0.00350	0.00320	0.00322	0.00268	0.00294	0.00400	0.00343	
65536	0.00822	0.00824	0.00897	0.00794	0.00936	0.00909	0.00833	0.00822	0.00969	0.00901	0.00755	0.00815	0.00844	0.00842	0.00920		
32768	0.01220	0.01193	0.01235	0.01162	0.01221	0.01213	0.01177	0.01191	0.01314	0.01241	0.01117	0.01208	0.01182	0.01150			
16384	0.01509	0.01569	0.01521	0.01479	0.01599	0.01465	0.01527	0.01479	0.01606	0.01528	0.01400	0.01552	0.01526				
8192	0.03442	0.03513	0.03404	0.03313	0.03416	0.03371	0.03422	0.03442	0.03530	0.03260	0.03324	0.03377					
4096	0.03090	0.03159	0.03181	0.03026	0.03190	0.03060	0.03098	0.03090	0.03303	0.03039	0.03041						
2048	0.09771	0.10109	0.09958	0.09953	0.10017	0.10093	0.10245	0.09636	0.10167	0.09833							
1024	0.11968	0.12196	0.12054	0.11918	0.12094	0.12070	0.12317	0.11868	0.12321								
512	0.16170	0.16584	0.16048	0.16046	0.16394	0.16120	0.16587	0.16170									
256	0.23921	0.25094	0.24470	0.24283	0.25285	0.24333	0.25096										
128	0.37751	0.39074	0.38435	0.37103	0.38390	0.37131											
64	0.63056	0.65237	0.67343	0.62217	0.65237												
32	1.12025	1.19810	1.20024	1.10286													
16	1.75378	1.75351	1.75565														
8	1.69023	1.63307															
4	1.53527																

(f) $\mathcal{P}^{\mathcal{C}}$ for Scene: [**Veach Bidir**] Algorithm: [**ERPT**] Metric: [**WBCT**] True GT: [**BDPT @ 524288 spp**].

M-ERPT	2	4	8	16	32	64	128	256	512	1024	2048	4096	8192	16384	32768	65536	131072
GT (BDPT)																	
262144	0.00223	0.00174	0.00251	0.00171	0.00222	0.00201	0.00174	0.00225	0.00251	0.00273	0.00228	0.00220	0.00246	0.00178	0.00195	0.00199	0.00193
131072	0.00348	0.00300	0.00353	0.00294	0.00371	0.00328	0.00299	0.00300	0.00353	0.00374	0.00331	0.00318	0.00370	0.00306	0.00317	0.00324	
65536	0.00882	0.00759	0.00816	0.00872	0.00853	0.00845	0.00835	0.00761	0.00868	0.00859	0.00879	0.00844	0.00900	0.00828	0.00817		
32768	0.01142	0.01203	0.01106	0.01155	0.01190	0.01109	0.01175	0.01074	0.01185	0.01198	0.01280	0.01178	0.01185	0.01176			
16384	0.01460	0.01524	0.01454	0.01469	0.01507	0.01485	0.01495	0.01421	0.01535	0.01517	0.01581	0.01386	0.01447				
8192	0.03312	0.03457	0.03273	0.03244	0.03327	0.03253	0.03332	0.03169	0.03243	0.03412	0.03374	0.03263					
4096	0.03019	0.03101	0.02976	0.02926	0.03035	0.02985	0.03037	0.02934	0.02976	0.03116	0.03222						
2048	0.09665	0.10004	0.09459	0.09208	0.09781	0.09577	0.09525	0.09493	0.09335	0.09699							
1024	0.11492	0.11831	0.11336	0.11282	0.11795	0.11378	0.11531	0.11457	0.11474								
512	0.15640	0.15800	0.15280	0.15446	0.15997	0.15455	0.15373	0.15299									
256	0.23817	0.24114	0.23157	0.24096	0.23891	0.23940	0.23718										
128	0.36799	0.37274	0.36111	0.36700	0.37021	0.36207											
64	0.62543	0.62819	0.61902	0.63953	0.60701												
32	1.19281	1.13441	1.09911	1.21573													
16	1.70563	1.76794	1.76670														
8	1.64874	1.76794															
4	1.55067																

(g) $\mathcal{P}^{\mathcal{C}}$ for Scene: [**Veach Bidir**] Algorithm: [**M-ERPT**] Metric: [**WBCT**] True GT: [**BDPT @ 524288 spp**].

Table 3

2.4 Veach Door

PT	2	4	8	16	32	64	128	256	512	1024	2048	4096	8192	16384	32768	65536	131072
GT (BDPT)																	
262144	0.00014	-0.00006	0.00026	0.00007	0.00008	0.00029	0.00049	0.00017	0.00025	0.00046	0.00000	-0.00300	-0.00968	-0.02948	-0.06355	-0.06355	-0.09496
131072	0.00014	-0.00013	0.00026	0.00007	0.00041	0.00039	0.00074	0.00050	0.00125	0.00092	-0.00103	-0.00300	-0.03718	-0.05716	-0.17180	-0.17180	
65536	0.00028	0.00006	0.00032	0.00049	0.00065	0.00126	0.00160	0.00218	0.00276	0.00323	0.00417	0.00000	-0.00968	-0.02948	-0.12044		
32768	0.00056	0.00078	0.00110	0.00155	0.00230	0.00343	0.00484	0.00628	0.00840	0.01081	0.01607	0.02529	0.05282	0.06530			
16384	0.00113	0.00123	0.00195	0.00305	0.00455	0.00663	0.00879	0.01209	0.01639	0.02379	0.03577	0.05313	0.07633				
8192	0.00198	0.00260	0.00366	0.00587	0.00846	0.01229	0.01700	0.02395	0.03204	0.04730	0.07527	0.10954					
4096	0.00427	0.00552	0.00755	0.01198	0.01741	0.02415	0.03326	0.04802	0.06656	0.10065	0.16656						
2048	0.00807	0.01049	0.01479	0.02228	0.03264	0.04602	0.06323	0.09045	0.12960	0.19402							
1024	0.01465	0.01874	0.02635	0.03939	0.05838	0.08367	0.11660	0.16543	0.24469								
512	0.02496	0.03156	0.04528	0.06784	0.10122	0.14556	0.20344	0.29551									
256	0.04163	0.05097	0.07350	0.11129	0.16882	0.24503	0.35293										
128	0.06581	0.08108	0.11751	0.17919	0.28162	0.42246											
64	0.10012	0.12259	0.18023	0.28147	0.45893												
32	0.15016	0.18549	0.27873	0.45430													
16	0.22847	0.28887	0.44256														
8	0.33836	0.43126															
4	0.47862																

(a) $\mathcal{P}^{\mathcal{C}}$ for Scene: [**Veach Door**] Algorithm: [**PT**] Metric: [**WBCT**] True GT: [**BDPT @ 524288 spp**].

BDPT	2	4	8	16	32	64	128	256	512	1024	2048	4096	8192	16384	32768	65536	131072
GT (BDPT)																	
262144	-0.00006	0.00007	0.00000	0.00025	0.00022	0.00034	0.00031	0.00000	0.00488	0.00870	0.02459	0.05136	-0.06355	0.08191	-0.09496	-0.11220	0.00000
131072	-0.00013	-0.00007	0.00014	0.00017	0.00067	0.00084	0.00093	0.00157	0.00000	-0.01676	-0.04532	-0.08946	-0.12044	0.00000	0.00000	-0.11220	
65536	0.00013	0.00020	0.00057	0.00077	0.00122	0.00220	0.00249	0.00556	0.00735	-0.01676	-0.04532	-0.04671	-0.06355	0.08191	0.24808		
32768	0.00064	0.00105	0.00173	0.00266	0.00404	0.00650	0.00986	0.01884	0.03073	0.03621	0.10780	0.24351	0.07171	0.59513			
16384	0.00142	0.00204	0.00318	0.00483	0.00795	0.01272	0.01923	0.03854	0.05635	0.03621	0.10780	0.10825	0.00000				
8192	0.00266	0.00390	0.00614	0.00928	0.01484	0.02433	0.03838	0.06969	0.10526	0.13456	0.17310	0.10825					
4096	0.00576	0.00811	0.01238	0.01924	0.02959	0.04862	0.07982	0.14572	0.22233	0.34677	0.51008						
2048	0.01154	0.01538	0.02334	0.03683	0.05690	0.09325	0.15854	0.29560	0.46438	0.68367							
1024	0.01989	0.02733	0.04149	0.06616	0.10514	0.17108	0.29721	0.54551	0.97847								
512	0.03371	0.04666	0.07166	0.11482	0.18631	0.31610	0.55378	1.08449									
256	0.05423	0.07610	0.11847	0.19285	0.32251	0.57875	1.09405										
128	0.08555	0.12012	0.19488	0.31602	0.56779	1.06749											
64	0.12995	0.18573	0.30544	0.52207	1.07750												
32	0.19834	0.28982	0.48971	0.91553													
16	0.30796	0.46614	0.84290														
8	0.46759	0.75566															
4	0.71489																

(b) $\mathcal{P}^{\mathcal{C}}$ for Scene: [**Veach Door**] Algorithm: [**BDPT**] Metric: [**WBCT**] True GT: [**BDPT @ 524288 spp**].

PSSMLT	2	4	8	16	32	64	128	256	512	1024	2048	4096	8192	16384
GT (BDPT)														
262144	0.00008	0.00009	0.00000	0.00059	0.00047	0.00173	0.00202	0.00000	0.00807	0.01739	-0.03151	-0.07171	0.00000	-0.11220
131072	0.00008	0.00017	0.00011	0.00089	0.00047	0.00087	-0.00200	-0.00267	-0.01558	-0.03265	-0.13931	-0.13526	-0.17180	-0.42433
65536	0.00045	0.00069	0.00097	0.00178	0.00282	0.00393	0.00304	0.00000	-0.00788	-0.01666	-0.06099	-0.13526	0.00000	-0.20717
32768	0.00160	0.00224	0.00324	0.00509	0.00764	0.01248	0.01670	0.02242	0.03353	0.05460	0.10952	0.08191	0.49666	0.13587
16384	0.00321	0.00417	0.00644	0.01002	0.01485	0.02386	0.03124	0.04686	0.02483	0.09552	0.15227	0.00000	0.36078	
8192	0.00593	0.00836	0.01303	0.01936	0.02829	0.04495	0.06212	0.09187	0.07071	0.16543	0.30723	-0.07171		
4096	0.01178	0.01691	0.02659	0.03918	0.05636	0.08700	0.13235	0.19430	0.21331	0.43242	0.52439			
2048	0.02217	0.03373	0.04961	0.07369	0.11081	0.17105	0.25834	0.39474	0.62641	0.80454				
1024	0.03983	0.06004	0.08895	0.13393	0.20307	0.32388	0.46679	0.74096	1.42367					
512	0.06737	0.10134	0.15296	0.22992	0.35997	0.55473	0.79094	1.40466						
256	0.10917	0.16703	0.25074	0.38802	0.60093	1.01398	1.64348							
128	0.17310	0.26616	0.41181	0.64320	1.03665	1.66490								
64	0.26486	0.41896	0.65757	1.10195	1.84999									
32	0.40613	0.68162	1.16698	1.92852										
16	0.64093	1.21528	1.69010											
8	1.13669	1.36683												
4	1.20740													

(c) $\mathcal{P}^{\mathcal{C}}$ for Scene: [**Veach Door**] Algorithm: [**PSSMLT**] Metric: [**WBCT**] True GT: [**BDPT @ 524288 spp**].

MLT	2	4	8	16	32	64	128	256	512	1024	2048	4096	8192	16384
GT (BDPT)														
262144	-0.00008	0.00036	0.00024	0.00053	0.00031	0.00000	0.00156	0.00000	-0.00941	-0.02096	-0.04671	-0.05689	-0.08191	-0.09496
131072	0.00000	0.00018	0.00024	0.00053	0.00063	0.00068	0.00000	-0.01482	-0.03621	-0.04092	-0.16519	-0.19771	-0.15362	-0.36902
65536	0.00046	0.00071	0.00131	0.00211	0.00347	0.00272	0.00313	-0.00378	-0.01858	-0.04092	-0.08946	-0.10825	0.09496	-0.09496
32768	0.00179	0.00241	0.00385	0.00606	0.00897	0.01397	0.01609	0.01564	0.01963	0.04532	0.05136	0.06355	0.20717	0.11220
16384	0.00320	0.00469	0.00729	0.01234	0.01702	0.02586	0.03332	0.02795	0.00968	0.06991	-0.08946	-0.05689	0.09496	
8192	0.00566	0.00914	0.01480	0.02341	0.03350	0.05098	0.06367	0.07318	0.07406	0.12364	0.00000	0.06355		
4096	0.01171	0.01865	0.02951	0.04520	0.06920	0.10532	0.13007	0.17679	0.19839	0.43497	0.24351			
2048	0.02259	0.03475	0.05604	0.08593	0.13240	0.20392	0.25335	0.37542	0.46369	0.80399				
1024	0.04069	0.06081	0.09963	0.15515	0.23787	0.35854	0.45416	0.57902	0.80243					
512	0.06877	0.10343	0.16926	0.26398	0.41486	0.58326	0.77370	0.98360						
256	0.10993	0.16853	0.28302	0.43441	0.65344	0.90763	1.21726							
128	0.17210	0.27280	0.45500	0.68434	0.99780	1.20444								
64	0.26348	0.42224	0.71447	1.04812	1.60528									
32	0.40071	0.65623	1.16390	1.65629										
16	0.63772	1.13422	1.63056											
8	1.06556	1.50593												
4	1.26011													

(d) $\mathcal{P}^{\mathcal{C}}$ for Scene: [**Veach Door**] Algorithm: [**MLT**] Metric: [**WBCT**] True GT: [**BDPT @ 524288 spp**].

M-MLT	2	4	8	16	32	64	128	256	512	1024	2048	4096	8192	16384
GT (BDPT)														
262144	0.00030	0.00009	0.00024	0.00018	0.00160	0.00282	0.00000	0.00000	0.02019	-0.02326	-0.03379	0.00000	-0.09496	-0.11220
131072	0.00023	0.00027	0.00036	0.00054	0.00160	0.00211	-0.00515	-0.00863	-0.00968	-0.10527	-0.12246	-0.24351	-0.31213	-0.28907
65536	0.00053	0.00089	0.00132	0.00217	0.00387	0.00496	0.00000	-0.00435	0.00995	-0.10527	-0.09479	-0.19215	-0.24858	-0.20717
32768	0.00181	0.00267	0.00375	0.00640	0.01051	0.01601	0.01428	0.03238	0.05282	0.05064	0.07573	0.17687	0.00000	0.30606
16384	0.00303	0.00521	0.00784	0.01208	0.02022	0.03008	0.02750	0.04740	0.05282	0.00000	0.03638	0.28907	0.00000	
8192	0.00582	0.00928	0.01543	0.02220	0.03773	0.05218	0.06060	0.06867	0.11467	0.07831	0.07573	-0.07171		
4096	0.01211	0.01905	0.03044	0.04673	0.07322	0.10332	0.13709	0.18758	0.30089	0.33829	0.49060			
2048	0.02306	0.03566	0.05732	0.08808	0.13941	0.20143	0.27095	0.35973	0.85630	0.87087				
1024	0.04061	0.06371	0.10182	0.15764	0.24876	0.36668	0.47822	0.71230	0.85630					
512	0.06905	0.11094	0.17832	0.27372	0.42647	0.57569	0.72924	1.18630						
256	0.11197	0.18101	0.29867	0.45591	0.68722	0.97132	1.36277							
128	0.17651	0.29745	0.49555	0.74163	1.02300	1.50410								
64	0.27441	0.45998	0.78506	1.29049	1.79364									
32	0.41929	0.73754	1.31438	1.76029										
16	0.67701	1.27091	1.77031											
8	1.23531	1.42842												
4	1.14854													

(e) $\mathcal{P}^{\mathcal{C}}$ for Scene: [**Veach Door**] Algorithm: [**M-MLT**] Metric: [**WBCT**] True GT: [**BDPT @ 524288 spp**].

ERPT	2	4	8	16	32	64	128	256	512	1024	2048	4096	8192	16384	32768	65536	131072
GT (BDPT)																	
262144	0.00424	0.00236	0.00000	-0.00257	0.00252	0.00257	-0.00265	0.00522	0.00000	-0.00247	0.00000	0.00549	0.00255	0.00507	0.00000	0.00554	0.00000
131072	-0.00209	-0.00234	-0.00684	-0.00764	-0.00250	-0.00756	-0.00265	0.00000	-0.00735	0.00000	-0.00262	-0.00270	-0.00252	0.00000	-0.00764	0.00000	
65536	0.00424	0.00000	0.00000	-0.00257	0.00252	0.00517	0.00538	0.00260	-0.00247	0.00250	0.00265	-0.00270	0.00000	0.00000	0.00000		
32768	0.01293	0.01450	0.02418	0.01324	0.02637	0.02130	0.01931	0.02718	0.01800	0.01800	0.02483	0.01972	0.02109	0.02915			
16384	0.03124	0.02723	0.03984	0.03005	0.04353	0.03262	0.04322	0.03887	0.03731	0.03731	0.03068	0.03474	0.03807				
8192	0.06406	0.06954	0.07117	0.07387	0.07813	0.07306	0.08350	0.07728	0.06754	0.07401	0.06528	0.06749					
4096	0.13425	0.13854	0.15112	0.15589	0.15905	0.16763	0.16666	0.15589	0.15278	0.15713	0.14619						
2048	0.25836	0.28145	0.29245	0.32669	0.33432	0.34521	0.34305	0.30410	0.30704	0.33682							
1024	0.41292	0.47153	0.48740	0.57801	0.54130	0.53623	0.54002	0.51995	0.54379								
512	0.58718	0.73081	0.85911	1.00032	1.00796	0.89337	0.99246	1.00032									
256	0.97811	1.35793	1.22733	1.33329	1.34093	1.51273	1.40733										
128	1.19466	1.28622	1.36259	1.33329	1.05071	0.92717											
64	1.01450	0.98562	0.85911	0.75150	0.75913												
32	0.82960	0.73081	0.73547	0.68521													
16	0.64751	0.58729	0.60730														
8	0.43054	0.37556															
4	0.22327																

(f) $\mathcal{P}^{\mathcal{C}}$ for Scene: [**Veach Door**] Algorithm: [**ERPT**] Metric: [**WBCT**] True GT: [**BDPT @ 524288 spp**].

M-ERPT	2	4	8	16	32	64	128	256	512	1024	2048	4096	8192	16384	32768	65536	131072
GT (BDPT)																	
262144	0.00183	-0.00201	-0.00204	0.00000	0.00000	0.00000	0.00000	0.00000	0.00666	0.00000	0.00000	-0.00211	-0.00224	0.00207	0.00224	0.00000	0.00236
131072	-0.00182	-0.00598	-0.00607	0.00000	-0.00438	0.00000	-0.00420	0.00000	-0.00218	-0.00649	-0.00207	-0.00628	-0.00446	-0.00206	-0.00222	-0.00622	
65536	0.00554	0.00000	0.00000	0.00209	-0.00220	0.00413	0.00000	0.00211	0.00442	0.00220	0.00209	0.00213	0.00226	0.00417	0.00000		
32768	0.01699	0.01667	0.01477	0.01948	0.02070	0.02361	0.01981	0.02192	0.02052	0.02052	0.01724	0.02211	0.02107	0.02609			
16384	0.03313	0.02544	0.03510	0.03812	0.03547	0.03985	0.04127	0.03846	0.03515	0.03767	0.03572	0.04629	0.03613				
8192	0.06176	0.05367	0.07028	0.06615	0.07649	0.07571	0.07574	0.06949	0.07284	0.06997	0.07158	0.07858					
4096	0.13672	0.12443	0.14397	0.14338	0.16242	0.15110	0.15759	0.15220	0.16462	0.14898	0.16563						
2048	0.27804	0.27947	0.30423	0.29412	0.32357	0.31655	0.32103	0.30411	0.31242	0.33266							
1024	0.46576	0.49983	0.52941	0.51371	0.54732	0.57969	0.58507	0.52318	0.53675								
512	0.75400	0.86594	0.95054	0.94641	0.96519	1.02072	1.05173	1.01450									
256	1.28677	1.32539	1.47494	1.38890	1.55075	1.68210	1.67377										
128	1.35032	1.32539	1.20088	1.31719	1.30217	1.39303											
64	0.97954	0.83989	0.78797	0.85774	0.81668												
32	0.79491	0.72906	0.74495	0.76178													
16	0.65139	0.62646	0.60762														
8	0.44814	0.42321															
4	0.25849																

(g) $\mathcal{P}^{\mathcal{C}}$ for Scene: [**Veach Door**] Algorithm: [**M-ERPT**] Metric: [**WBCT**] True GT: [**BDPT @ 524288 spp**].

Table 4

2.5 Sponza

PT	2	4	8	16	32	64	128	256	512	1024	2048	4096	8192
GT (PT)													
16384	0.00048	0.00106	0.00132	0.00212	0.00316	0.00534	0.00940	0.01471	0.03330	0.05443	0.12246	0.15496	0.13526
8192	0.00144	0.00226	0.00351	0.00540	0.00874	0.01395	0.02463	0.03697	0.06790	0.07872	0.15884	0.04671	
4096	0.00368	0.00527	0.00812	0.01237	0.02025	0.03261	0.05550	0.09078	0.18671	0.25002	0.39590		
2048	0.00732	0.01067	0.01609	0.02531	0.04080	0.06524	0.10882	0.18485	0.34005	0.50936			
1024	0.01509	0.02131	0.03185	0.04985	0.07990	0.12995	0.22317	0.39080	0.68262				
512	0.02889	0.04064	0.06065	0.09455	0.15113	0.25157	0.45968	0.84343					
256	0.05315	0.07405	0.11056	0.17211	0.28362	0.49431	0.99591						
128	0.09155	0.12919	0.19179	0.30707	0.53689	1.02995							
64	0.15054	0.21274	0.32549	0.54007	1.03542								
32	0.24455	0.34587	0.55820	1.03500									
16	0.37892	0.55652	0.98978										
8	0.58789	0.96771											
4	0.91074												

(a) $\mathcal{P}^{\mathcal{C}}$ for Scene: [**Sponza**] Algorithm: [**PT**] Metric: [**WBCT**] True GT: [**PT @ 32768 spp**].

BDPT	2	4	8	16	32	64	128	256	512	1024	2048	4096	8192
GT (PT)													
16384	0.00049	0.00080	0.00106	0.00173	0.00301	0.00381	0.00670	0.01186	0.01944	0.06427	0.07126	0.27344	0.42433
8192	0.00123	0.00203	0.00274	0.00436	0.00775	0.01156	0.01892	0.03591	0.05378	0.10193	0.09122	0.21655	
4096	0.00290	0.00467	0.00684	0.01026	0.01745	0.02652	0.04387	0.07860	0.13801	0.26132	0.40633		
2048	0.00599	0.00922	0.01373	0.02063	0.03443	0.05296	0.09125	0.16148	0.27447	0.49560			
1024	0.01289	0.01864	0.02724	0.04105	0.06672	0.10620	0.18496	0.33156	0.57437				
512	0.02512	0.03553	0.05163	0.07796	0.12561	0.20748	0.36899	0.69884					
256	0.04662	0.06519	0.09400	0.14105	0.23151	0.39065	0.73525						
128	0.08042	0.11142	0.15958	0.25021	0.42025	0.77238							
64	0.13328	0.18453	0.26688	0.43208	0.78424								
32	0.21327	0.30060	0.45348	0.79428									
16	0.32897	0.47019	0.76530										
8	0.50603	0.75916											
4	0.76234												

(b) $\mathcal{P}^{\mathcal{C}}$ for Scene: [**Sponza**] Algorithm: [**BDPT**] Metric: [**WBCT**] True GT: [**PT @ 32768 spp**].

PSSMLT	2	4	8	16	32	64	128	256	512	1024	2048	4096	8192
GT (PT)													
16384	0.00095	0.00121	0.00176	0.00253	0.00365	0.00495	0.00737	0.01129	0.01247	0.01694	0.01493	0.02046	0.01838
8192	0.00259	0.00336	0.00445	0.00670	0.00936	0.01378	0.01948	0.02954	0.03596	0.04263	0.04012	0.04702	
4096	0.00593	0.00768	0.01073	0.01507	0.02170	0.03000	0.04604	0.06360	0.08127	0.09456	0.09259		
2048	0.01159	0.01530	0.02136	0.03020	0.04359	0.06151	0.08828	0.12785	0.15643	0.17265			
1024	0.02296	0.03050	0.04242	0.05971	0.08395	0.12095	0.17951	0.25708	0.32397				
512	0.04350	0.05648	0.07786	0.11099	0.15719	0.22835	0.34131	0.49462					
256	0.07814	0.10135	0.13816	0.19603	0.28295	0.43225	0.68686						
128	0.13265	0.17179	0.23637	0.34233	0.51119	0.80589							
64	0.21102	0.27906	0.39431	0.58764	0.95715								
32	0.33406	0.45604	0.67025	1.06855									
16	0.51240	0.73515	1.13941										
8	0.81238	1.25870											
4	1.32804												

(c) $\mathcal{P}^{\mathcal{C}}$ for Scene: [**Sponza**] Algorithm: [**PSSMLT**] Metric: [**WBCT**] True GT: [**PT @ 32768 spp**].

MLT	2	4	8	16	32	64	128	256	512	1024	2048	4096	8192
GT (PT)													
16384	0.00099	0.00157	0.00207	0.00383	0.00540	0.00910	0.01285	0.01670	0.01527	0.01219	0.01839	0.01970	0.01576
8192	0.00251	0.00439	0.00609	0.01014	0.01480	0.02441	0.03374	0.03598	0.03669	0.03656	0.04452	0.04331	
4096	0.00609	0.00962	0.01446	0.02255	0.03383	0.05558	0.08022	0.08318	0.08820	0.09128	0.10647		
2048	0.01230	0.01887	0.02914	0.04566	0.06887	0.11178	0.14899	0.16877	0.16966	0.18021			
1024	0.02422	0.03713	0.05717	0.09138	0.13869	0.21652	0.29436	0.32376	0.35527				
512	0.04543	0.06881	0.10739	0.16851	0.27029	0.42316	0.55737	0.66064					
256	0.08099	0.12210	0.19333	0.31185	0.51015	0.82482	1.16030						
128	0.13939	0.21211	0.34486	0.57356	0.98230	1.53758							
64	0.22891	0.35778	0.60257	1.04617	2.05644								
32	0.37241	0.61033	1.09387	1.95399									
16	0.58528	1.05361	1.92997										
8	0.96640	1.90073											
4	1.64651												

(d) $\mathcal{P}^{\mathcal{C}}$ for Scene: [**Sponza**] Algorithm: [**MLT**] Metric: [**WBCT**] True GT: [**PT @ 32768 spp**].

M-MLT	2	4	8	16	32	64	128	256	512	1024	2048	4096	8192
GT (PT)													
16384	0.00100	0.00144	0.00229	0.00398	0.00591	0.00925	0.01379	0.01594	0.01517	0.01930	0.01773	0.02050	0.02634
8192	0.00281	0.00382	0.00639	0.01028	0.01630	0.02365	0.03162	0.03822	0.03645	0.04023	0.05536	0.04281	
4096	0.00615	0.00949	0.01503	0.02305	0.03655	0.05711	0.07628	0.08875	0.09611	0.09543	0.11788		
2048	0.01307	0.01944	0.02991	0.04649	0.07325	0.11483	0.14622	0.17672	0.18296	0.19227			
1024	0.02636	0.03848	0.05873	0.09354	0.14747	0.22357	0.30868	0.33829	0.37959				
512	0.04902	0.07168	0.10953	0.17356	0.28260	0.42481	0.59255	0.71504					
256	0.08869	0.12895	0.20068	0.31985	0.54260	0.83089	1.20712						
128	0.15204	0.22040	0.35899	0.59934	1.05734	1.58584							
64	0.25139	0.37425	0.64586	1.13435	1.87067								
32	0.41092	0.64123	1.15971	1.87598									
16	0.66072	1.09658	2.17592										
8	1.14594	1.96255											
4	1.76354												

(e) $\mathcal{P}^{\mathcal{C}}$ for Scene: [**Sponza**] Algorithm: [**M-MLT**] Metric: [**WBCT**] True GT: [**PT @ 32768 spp**].

ERPT	2	4	8	16	32	64	128	256	512	1024	2048	4096	8192
GT (PT)													
16384	0.01386	0.01237	0.01444	0.01214	0.01283	0.01361	0.01455	0.01805	0.01183	0.01499	0.01346	0.01392	0.01269
8192	0.03474	0.03063	0.03624	0.03220	0.03547	0.03351	0.03286	0.03682	0.02782	0.03465	0.03492	0.03524	
4096	0.07931	0.07357	0.07859	0.07770	0.08117	0.08082	0.08039	0.08338	0.07760	0.07479	0.07980		
2048	0.15197	0.15493	0.14806	0.15410	0.14879	0.15260	0.15880	0.16688	0.15188	0.15341			
1024	0.30092	0.30753	0.30621	0.29754	0.31397	0.31475	0.31319	0.34219	0.30298				
512	0.61204	0.58847	0.61926	0.60056	0.63484	0.65279	0.61771	0.64967					
256	1.12287	1.29188	1.32777	1.24255	1.48195	1.32777	1.24412						
128	1.62635	1.62485	1.48273	1.69677	1.48195	1.69989							
64	1.49109	1.48959	1.48273	1.54315	1.48195								
32	1.43420	1.38134	1.42584	1.37135									
16	1.29338	1.33463	1.32777										
8	1.02129	1.01979											
4	0.82672												

(f) $\mathcal{P}^{\mathcal{C}}$ for Scene: [**Sponza**] Algorithm: [**ERPT**] Metric: [**WBCT**] True GT: [**PT @ 32768 spp**].

M-ERPT	2	4	8	16	32	64	128	256	512	1024	2048	4096	8192
GT (PT)													
16384	0.01164	0.01323	0.01466	0.01105	0.01264	0.01471	0.01185	0.01174	0.01346	0.01552	0.01401	0.01417	0.01105
8192	0.03453	0.03227	0.03562	0.03002	0.03492	0.03399	0.03179	0.03376	0.03492	0.03575	0.03603	0.03376	
4096	0.07870	0.07933	0.08147	0.07893	0.07870	0.08072	0.07514	0.08133	0.07651	0.08400	0.08360		
2048	0.15706	0.16145	0.15493	0.15709	0.15188	0.15270	0.14782	0.15041	0.14888	0.15123			
1024	0.29134	0.31040	0.30360	0.31164	0.31134	0.31383	0.29781	0.30451	0.31134				
512	0.58019	0.57876	0.58293	0.59097	0.57688	0.58218	0.57662	0.59264					
256	1.16325	1.22713	1.15235	1.30142	1.21160	1.29263	1.26136						
128	1.81412	1.71773	1.62635	1.63439	1.79717	1.55389							
64	1.56553	1.50056	1.43420	1.56268	1.42815								
32	1.44510	1.44368	1.38284	1.49913									
16	1.30427	1.34560	1.33613										
8	1.05545	1.05403											
4	0.85183												

(g) $\mathcal{P}^{\mathcal{C}}$ for Scene: [**Sponza**] Algorithm: [**M-ERPT**] Metric: [**WBCT**] True GT: [**PT @ 32768 spp**].

Table 5

3 Contourlet

3.1 Cornell Box

PT	2	4	8	16	32	64	128	256	512	1024	2048	4096	8192
GT (PT)													
16384	0.00099	0.00108	0.00104	0.00241	0.00222	0.00000	0.02010	-0.02862	0.02862	-0.03476	-0.03302	-0.07988	-0.07988
8192	0.00232	0.00271	0.00454	0.00485	0.00899	-0.02304	0.00000	0.00000	0.05859	0.00000	0.07143	-0.07988	
4096	0.00547	0.00711	0.01025	0.01313	0.03010	0.01602	0.02010	0.06139	0.09002	0.11655	0.19501		
2048	0.01086	0.01504	0.01946	0.02957	0.06305	0.09574	0.06248	0.16584	0.19446	0.16025			
1024	0.02162	0.02875	0.03619	0.06062	0.10231	0.13589	-0.01943	0.12917	0.19446				
512	0.04002	0.05514	0.07638	0.12859	0.20770	0.29405	0.26965	0.12917					
256	0.07480	0.10051	0.14584	0.23166	0.39331	0.45851	0.33409						
128	0.12771	0.17420	0.26059	0.41146	0.65093	0.68899							
64	0.21565	0.30453	0.46127	0.75069	1.04489								
32	0.35141	0.50217	0.79695	1.31461									
16	0.56584	0.86393	1.32418										
8	0.91290	1.44243											
4	1.51445												

(a) $\mathcal{P}^{\mathcal{C}}$ for Scene: [**Cornell Box**] Algorithm: [**PT**] Metric: [**Contourlet**] True GT: [**PT @ 32768 spp**].

BDPT	2	4	8	16	32	64	128	256	512	1024	2048	4096	8192
GT (PT)													
16384	0.00116	0.00212	0.00423	0.00851	0.01268	-0.05140	0.00000	0.00000	-0.07988	-0.04111	0.00000	-0.04370	0.00000
8192	0.00280	0.00425	0.00423	0.00423	0.01268	-0.02623	-0.07143	-0.06778	-0.07988	-0.04111	0.08481	-0.04370	
4096	0.00876	0.01298	0.02027	0.02162	0.03899	0.02738	-0.03667	0.03667	0.00000	0.00000	0.00000		
2048	0.01813	0.03197	0.04354	0.06869	0.14284	0.11740	-0.10445	0.07544	-0.07988	-0.07988			
1024	0.03650	0.05637	0.08839	0.11618	0.15942	0.05600	-0.03667	0.07544	-0.04111				
512	0.07100	0.11308	0.16036	0.23789	0.33542	0.15042	-0.10445	0.00000					
256	0.12837	0.20610	0.29873	0.41481	0.49024	0.15042	-0.10445						
128	0.22843	0.34937	0.52434	0.65516	0.69486	0.22184							
64	0.39291	0.60766	0.84467	1.11514	0.55163								
32	0.65366	0.98590	1.37118	1.11514									
16	1.12864	1.53746	1.43257										
8	1.68349	1.73927											
4	1.86828												

(b) $\mathcal{P}^{\mathcal{C}}$ for Scene: [**Cornell Box**] Algorithm: [**BDPT**] Metric: [**Contourlet**] True GT: [**PT @ 32768 spp**].

PSSMLT	2	4	8	16	32	64	128	256	512	1024	2048	4096	8192
GT (PT)													
16384	0.00192	0.00173	0.00139	0.00833	0.00000	0.06476	-0.02738	0.02997	-0.02623	-0.02738	0.15779	0.00000	0.02738
8192	0.00468	0.00231	0.00841	0.00414	-0.01181	0.00000	0.00000	0.06139	0.00000	0.05859	0.05859	-0.02997	
4096	0.01032	0.01360	0.02147	0.02116	0.02446	0.06476	-0.02738	0.02997	0.00000	0.09002	0.00000		
2048	0.01963	0.02794	0.04134	0.05281	0.05012	0.06476	0.00000	-0.05600	-0.02623	0.05859			
1024	0.03691	0.05420	0.08888	0.11896	0.13554	0.06476	0.00000	-0.02862	0.05600				
512	0.07099	0.10801	0.17148	0.24622	0.29512	0.21958	0.05859	0.00000					
256	0.12843	0.19377	0.32366	0.41258	0.43246	0.31400	0.15779						
128	0.22689	0.33398	0.52266	0.68058	0.48608	0.34875							
64	0.39452	0.58507	0.95179	0.99430	0.64387								
32	0.66549	0.99085	1.43675	1.05570									
16	1.08501	1.59365	1.37535										
8	1.72039	1.82689											
4	1.86402												

(c) $\mathcal{P}^{\mathcal{C}}$ for Scene: [**Cornell Box**] Algorithm: [**PSSMLT**] Metric: [**Contourlet**] True GT: [**PT @ 32768 spp**].

MLT	2	4	8	16	32	64	128	256	512	1024	2048	4096	8192
GT (PT)													
16384	0.00049	0.00106	0.00122	0.00125	0.00512	0.00000	0.00837	0.01763	0.00000	-0.05600	0.00000	-0.03143	0.02997
8192	0.00148	0.00249	0.00387	0.00420	0.00824	0.01147	0.00837	0.01763	-0.02623	-0.02862	0.02997	0.03302	
4096	0.00388	0.00611	0.00971	0.01063	0.01887	0.02634	0.04342	0.01763	-0.05140	-0.02862	0.02997		
2048	0.00819	0.01254	0.01833	0.02494	0.03904	0.05802	0.09116	0.13652	0.08597	0.00000			
1024	0.01676	0.02352	0.03358	0.05113	0.06445	0.10739	0.13301	0.05462	0.00000				
512	0.03136	0.04348	0.06251	0.09149	0.13039	0.18634	0.26981	0.15890					
256	0.05786	0.08035	0.11789	0.16482	0.25217	0.37455	0.49460						
128	0.09853	0.13556	0.19372	0.29568	0.40533	0.58781							
64	0.16939	0.23490	0.34233	0.51179	0.74993								
32	0.27278	0.37531	0.57290	0.85695									
16	0.42884	0.61725	0.92689										
8	0.65934	0.96407											
4	1.02225												

(d) $\mathcal{P}^{\mathcal{C}}$ for Scene: [**Cornell Box**] Algorithm: [**MLT**] Metric: [**Contourlet**] True GT: [**PT @ 32768 spp**].

M-MLT	2	4	8	16	32	64	128	256	512	1024	2048	4096	8192
GT (PT)													
16384	0.00077	0.00086	0.00129	0.00183	0.00441	0.00661	0.00000	0.01521	-0.05140	-0.04742	-0.05600	0.05859	-0.03302
8192	0.00197	0.00210	0.00302	0.00553	0.00777	0.00000	-0.00884	-0.01480	0.00000	-0.02417	0.02997	0.05859	
4096	0.00458	0.00509	0.00807	0.01261	0.01923	0.02020	0.03709	0.06355	0.02738	0.00000	0.02997		
2048	0.00892	0.01109	0.01714	0.02488	0.04358	0.05266	0.05675	0.09827	0.08597	0.16879			
1024	0.01715	0.02270	0.03523	0.04531	0.07988	0.10494	0.13246	0.09827	-0.02623				
512	0.03294	0.04536	0.06503	0.09244	0.14287	0.22972	0.20771	0.23955					
256	0.06006	0.08284	0.11770	0.16562	0.27589	0.34930	0.36287						
128	0.10457	0.14109	0.20480	0.29271	0.44700	0.54436							
64	0.17825	0.24639	0.36127	0.52522	0.78644								
32	0.28808	0.39861	0.61443	0.94849									
16	0.46249	0.66662	1.01501										
8	0.70627	1.09007											
4	1.10830												

(e) $\mathcal{P}^{\mathcal{C}}$ for Scene: [**Cornell Box**] Algorithm: [**M-MLT**] Metric: [**Contourlet**] True GT: [**PT @ 32768 spp**].

ERPT	2	4	8	16	32	64	128	256	512	1024	2048	4096	8192
GT (PT)													
16384	0.00954	0.01819	0.01890	0.01752	0.00993	0.00936	0.00000	0.00954	0.01819	0.00918	0.02232	0.03171	0.01966
8192	0.00000	-0.00884	0.02863	0.02652	0.00000	0.00000	-0.01854	-0.00936	0.00901	0.00918	0.00000	0.02092	
4096	0.00000	0.03709	0.04870	0.04506	0.04098	0.01890	0.00954	0.02921	0.02754	0.01854	0.02232		
2048	0.03934	0.07723	0.15053	0.09475	0.09744	0.09145	0.06026	0.10491	0.09860	0.08959			
1024	0.07106	0.07723	0.21721	0.13844	0.13458	0.10273	0.06026	0.10491	0.10964				
512	0.16684	0.23540	0.36288	0.23889	0.29842	0.21721	0.18016	0.23705					
256	0.33532	0.31156	0.48596	0.34566	0.33424	0.32705	0.26791						
128	0.41183	0.48176	0.53338	0.47771	0.41337	0.40109							
64	0.66138	0.68893	0.76995	0.70645	0.64211								
32	0.66138	0.65896	0.67074	0.67648									
16	0.63142	0.68893	0.64078										
8	0.63142	0.65896											
4	0.57541												

(f) $\mathcal{P}^{\mathcal{C}}$ for Scene: [**Cornell Box**] Algorithm: [**ERPT**] Metric: [**Contourlet**] True GT: [**PT @ 32768 spp**].

M-ERPT	2	4	8	16	32	64	128	256	512	1024	2048	4096	8192
GT (PT)													
16384	0.00000	0.01928	0.00868	0.00000	0.01035	0.01720	0.01104	0.00884	0.00954	0.01752	0.01785	0.00936	0.00901
8192	-0.01014	0.00954	0.00868	0.00000	-0.01014	0.01720	-0.01080	0.00000	-0.00936	0.00868	0.00000	-0.00918	
4096	0.03171	0.02921	0.04506	0.00000	0.02092	0.03505	0.03386	0.03638	0.01928	0.03570	0.02703		
2048	0.07738	0.09337	0.08440	0.05072	0.08946	0.11384	0.09579	0.08607	0.03934	0.07427			
1024	0.11451	0.11672	0.09475	0.06152	0.08946	0.13568	0.13680	0.12976	0.08209				
512	0.26126	0.28402	0.26732	0.14431	0.18291	0.27584	0.26427	0.30429					
256	0.35239	0.31769	0.29733	0.29106	0.29598	0.35418	0.38316						
128	0.41488	0.49985	0.45690	0.49030	0.46050	0.53018							
64	0.59208	0.69281	0.64786	0.65184	0.65347								
32	0.62204	0.79726	0.59424	0.68327									
16	0.62204	0.60280	0.77089										
8	0.62204	0.60280											
4	0.53607												

(g) $\mathcal{P}^{\mathcal{C}}$ for Scene: [**Cornell Box**] Algorithm: [**M-ERPT**] Metric: [**Contourlet**] True GT: [**PT @ 32768 spp**].

Table 6

3.2 Torus

PT	2	4	8	16	32	64	128	256	512	1024	2048	4096	8192	16384	32768	65536	131072
GT (BDPT)																	
262144	0.01507	0.02331	0.03413	0.01585	0.01217	0.01024	0.00866	0.00877	0.01073	0.01598	0.01553	0.01962	0.03285	0.06226	0.03042	0.01943	0.12620
131072	0.04352	0.06348	0.09175	0.04962	0.03401	0.03025	0.02524	0.02653	0.02936	0.04035	0.04826	0.06116	0.08784	0.15236	0.16089	0.23048	
65536	0.09280	0.14021	0.18732	0.10531	0.07460	0.06382	0.05826	0.05999	0.06326	0.08257	0.10286	0.13694	0.18935	0.33714	0.39256		
32768	0.17786	0.27092	0.34351	0.18254	0.13721	0.11663	0.11519	0.11302	0.12477	0.15276	0.19437	0.25128	0.36735	0.65864			
16384	0.32934	0.47975	0.56318	0.31651	0.24236	0.21009	0.19454	0.19876	0.21679	0.26943	0.36271	0.50263	0.82006				
8192	0.57854	0.81288	0.87153	0.51544	0.38080	0.33662	0.32214	0.32262	0.35776	0.43295	0.61406	0.89882					
4096	0.98928	1.17456	1.19478	0.74827	0.55614	0.50439	0.47167	0.48347	0.53910	0.69605	1.08249						
2048	1.59610	1.70794	1.56053	1.15082	0.84527	0.78017	0.69546	0.71333	0.82307	1.16449							
1024	1.77648	1.51925	1.25833	1.57516	1.17710	1.11783	0.99600	1.02167	1.24270								
512	1.46055	1.32510	1.00742	1.28608	1.44584	1.42475	1.35792	1.44320									
256	1.29877	1.15638	0.87947	0.99587	1.46909	1.53693	1.72142										
128	1.21986	1.05275	0.79223	0.93305	1.30457	1.51276											
64	1.24982	1.03184	0.76037	0.92331	1.36098												
32	1.35337	1.03184	0.75427	0.94298													
16	1.58088	1.37775	0.97701														
8	1.77648	1.90975															
4	1.91925																

(a) $\mathcal{P}^{\mathcal{C}}$ for Scene: [**Torus**] Algorithm: [**PT**] Metric: [**Contourlet**] True GT: [**BDPT @ 524288 spp**].

BDPT	2	4	8	16	32	64	128	256	512	1024	2048	4096	8192	16384	32768	65536	131072
GT (BDPT)																	
262144	0.01348	0.02074	0.02821	0.01777	0.01279	0.00989	0.01242	0.00953	0.00950	0.01016	0.01688	0.01847	0.02985	0.04521	0.08440	0.08830	0.02738
131072	0.03959	0.06227	0.07074	0.05133	0.03560	0.03255	0.02956	0.02778	0.03011	0.03563	0.04512	0.05723	0.09240	0.16013	0.21191	0.26517	
65536	0.08587	0.13513	0.15128	0.10517	0.07043	0.06606	0.06577	0.05871	0.06498	0.07777	0.09689	0.13255	0.19471	0.35832	0.41737		
32768	0.17077	0.26237	0.27498	0.18032	0.13754	0.12167	0.11876	0.11551	0.12617	0.14664	0.18003	0.26016	0.38251	0.65733			
16384	0.30901	0.48040	0.44133	0.30043	0.23189	0.20783	0.20922	0.19762	0.21277	0.26251	0.33600	0.48934	0.84992				
8192	0.53994	0.82187	0.69685	0.48363	0.36433	0.33685	0.34138	0.32713	0.34683	0.44282	0.58905	0.94337					
4096	0.90728	1.30212	0.99339	0.72890	0.53496	0.50178	0.49805	0.48918	0.54048	0.70215	0.96884						
2048	1.48708	1.90155	1.38330	1.11117	0.79777	0.75383	0.74239	0.74053	0.83246	1.15529							
1024	1.90746	1.46101	1.40209	1.37697	1.16283	1.09493	1.04335	1.02926	1.21315								
512	1.49945	1.22802	1.19662	1.19659	1.41531	1.46732	1.49157	1.46103									
256	1.31321	1.10706	1.02978	1.06104	1.48251	1.69606	1.77255										
128	1.28002	1.00528	0.92054	0.99401	1.41531	1.51127											
64	1.32189	0.99926	0.93577	0.97353	1.39450												
32	1.38748	0.99926	0.93577	1.06104													
16	1.51213	1.26900	1.34747														
8	1.59532	1.64139															
4	1.79092																

(b) $\mathcal{P}^{\mathcal{C}}$ for Scene: [**Torus**] Algorithm: [**BDPT**] Metric: [**Contourlet**] True GT: [**BDPT @ 524288 spp**].

PSSMLT	2	4	8	16	32	64	128	256	512	1024	2048	4096	8192	16384
GT (BDPT)														
262144	0.01142	0.01447	0.01325	0.01024	0.01386	0.01424	0.01368	0.01728	0.02339	0.02222	0.03273	0.05000	0.04969	0.08064
131072	0.03412	0.04081	0.03719	0.03535	0.03678	0.03821	0.03945	0.04438	0.06506	0.06973	0.09710	0.17922	0.14118	0.21717
65536	0.07099	0.08490	0.07711	0.07376	0.07500	0.07871	0.08193	0.10283	0.12992	0.16850	0.25348	0.30476	0.31769	0.31213
32768	0.13481	0.16102	0.13668	0.13348	0.13481	0.14396	0.15770	0.18475	0.24565	0.33351	0.44930	0.61894	0.49985	0.48878
16384	0.23186	0.27463	0.23583	0.24189	0.23702	0.24783	0.27787	0.33479	0.48227	0.65168	0.89462	0.84083	0.47660	
8192	0.37656	0.43013	0.37667	0.37648	0.39126	0.40661	0.47613	0.58992	0.85591	1.23725	1.00273	0.58311		
4096	0.57665	0.63300	0.55164	0.55712	0.57330	0.63302	0.79360	0.90003	1.46760	1.16467	0.78690			
2048	0.87474	0.92880	0.79161	0.85327	0.86594	0.99835	1.34418	1.52190	1.15974	0.94033				
1024	1.17015	1.24087	1.08972	1.13829	1.12592	1.27967	1.30597	1.14275	0.89959					
512	1.25398	1.31107	1.21249	1.23670	1.24938	1.27967	1.09362	0.90976						
256	1.31446	1.32629	1.28657	1.28504	1.23416	1.10787	0.94309							
128	1.32745	1.25419	1.31926	1.30213	1.17728	1.07244								
64	1.30178	1.28188	1.19883	1.20669	1.19094									
32	1.36846	1.39172	1.27093	1.35675										
16	0.97571	1.17894	0.99112											
8	0.95112	1.02044												
4	1.05137													

(c) $\mathcal{P}^{\mathcal{C}}$ for Scene: [**Torus**] Algorithm: [**PSSMLT**] Metric: [**Contourlet**] True GT: [**BDPT @ 524288 spp**].

MLT	2	4	8	16	32	64	128	256	512	1024	2048	4096	8192	16384
GT (BDPT)														
262144	0.00551	0.00871	0.01044	0.01606	0.01929	0.01638	0.02728	0.01285	0.01536	0.00548	0.03538	0.02266	0.00837	0.08320
131072	0.01670	0.02464	0.03070	0.04349	0.05535	0.04486	0.06370	0.05443	0.04801	0.06450	0.09450	0.10731	0.11164	0.13016
65536	0.03557	0.04869	0.06681	0.09001	0.11375	0.10754	0.14477	0.11495	0.13291	0.14771	0.19636	0.22158	0.20313	0.19967
32768	0.06479	0.09259	0.12664	0.16480	0.20539	0.18313	0.26468	0.22637	0.23949	0.26867	0.32612	0.38458	0.31422	0.21846
16384	0.11121	0.16127	0.21763	0.27892	0.38719	0.33936	0.48215	0.43651	0.51505	0.60292	0.56807	0.59327	0.61114	
8192	0.17749	0.26001	0.35584	0.45670	0.63339	0.54201	0.70791	0.71259	0.92235	0.79858	0.86416	0.59327		
4096	0.26106	0.38449	0.51994	0.67596	0.93150	0.78039	0.89992	0.82976	0.83098	0.68205	0.60506			
2048	0.37604	0.55322	0.77995	0.99991	1.21396	1.08099	0.98298	0.99996	0.77064	0.51731				
1024	0.48969	0.72353	1.07635	1.04960	1.14446	0.98824	0.93167	0.79801	0.57849					
512	0.59383	0.91726	1.11344	1.06017	1.00162	0.89710	0.72975	0.59544						
256	0.66642	1.00909	1.22062	1.10482	0.86044	0.78039	0.58570							
128	0.61059	0.96381	1.22062	1.07096	0.73647	0.69419								
64	0.52909	0.86061	1.17495	1.00945	0.71522									
32	0.47381	0.75539	1.15359	1.06017										
16	0.37706	0.58178	0.88262											
8	0.39383	0.59106												
4	0.49267													

(d) $\mathcal{P}^{\mathcal{C}}$ for Scene: [**Torus**] Algorithm: [**MLT**] Metric: [**Contourlet**] True GT: [**BDPT @ 524288 spp**].

M-MLT	2	4	8	16	32	64	128	256	512	1024	2048	4096	8192	16384
GT (BDPT)														
262144	0.00699	0.00993	0.01469	0.01241	0.02059	0.02591	0.02602	0.04752	0.02850	0.07230	0.03105	0.04780	0.08108	0.06248
131072	0.01944	0.02901	0.04098	0.03815	0.05514	0.06323	0.07760	0.13145	0.09184	0.09423	0.17865	0.14368	0.19415	0.13228
65536	0.04101	0.05912	0.08452	0.08174	0.11367	0.12716	0.14980	0.23657	0.21638	0.21301	0.34310	0.31846	0.31304	0.21106
32768	0.07601	0.11358	0.15885	0.15333	0.21617	0.21131	0.27528	0.36569	0.45177	0.47138	0.49481	0.43064	0.35867	0.13228
16384	0.13245	0.19097	0.27307	0.26673	0.36358	0.38214	0.52893	0.71977	0.74377	0.86485	0.47064	0.43064	0.33542	
8192	0.21750	0.30477	0.45176	0.42428	0.57623	0.61784	0.90608	0.97749	0.69814	0.65768	0.36253	0.24195		
4096	0.32464	0.46140	0.68689	0.63155	0.86460	0.87006	0.97349	0.73740	0.59744	0.59520	0.20785			
2048	0.45401	0.68009	1.04287	0.88581	1.22716	1.02895	1.09238	0.66999	0.42409	0.44052				
1024	0.59677	0.93678	1.21450	1.03529	1.22716	0.98454	0.89043	0.48417	0.30418					
512	0.74665	1.19692	1.24993	1.08399	1.06773	0.91786	0.67751	0.33190						
256	0.85440	1.33810	1.21450	1.14956	0.93610	0.81603	0.54416							
128	0.78469	1.32573	1.19218	1.17345	0.82487	0.70651								
64	0.67736	1.16938	1.14040	1.04465	0.84036									
32	0.60651	0.95855	1.26230	1.17345										
16	0.48005	0.71453	1.26230											
8	0.48346	0.73305												
4	0.59200													

(e) $\mathcal{P}^{\mathcal{C}}$ for Scene: [**Torus**] Algorithm: [**M-MLT**] Metric: [**Contourlet**] True GT: [**BDPT @ 524288 spp**].

ERPT	2	4	8	16	32	64	128	256	512	1024	2048	4096	8192	16384	32768	65536	131072
GT (BDPT)																	
262144	0.04333	0.02002	0.02618	0.02693	0.01189	0.02175	0.02201	0.04046	0.04471	0.02893	0.03492	0.02842	0.05995	0.02637	0.05032	0.03602	0.04520
131072	0.10151	0.05971	0.06942	0.09551	0.05562	0.06475	0.07351	0.08906	0.09254	0.09461	0.11591	0.12817	0.16953	0.13055	0.13063	0.09965	
65536	0.23001	0.18883	0.15888	0.22150	0.15936	0.14409	0.15829	0.18042	0.20291	0.23047	0.25320	0.28129	0.31475	0.31511	0.27587		
32768	0.42135	0.35257	0.29635	0.36805	0.29669	0.27061	0.30233	0.32606	0.35587	0.42512	0.50464	0.52415	0.58266	0.55978			
16384	0.73740	0.66211	0.59544	0.82656	0.56310	0.50628	0.59696	0.63590	0.68204	0.86484	1.10576	1.01439	0.89017				
8192	0.87867	1.02967	0.84635	0.95410	0.90778	0.90856	1.04435	1.06281	1.02984	1.08673	0.89859	0.75528					
4096	0.75559	0.86609	0.97839	0.80777	1.02668	1.13198	1.04435	1.04043	1.02984	0.84664	0.69664						
2048	0.70268	0.77195	0.93748	0.73827	0.90778	0.99970	0.85177	0.80846	0.79029	0.67816							
1024	0.50699	0.57097	0.71259	0.55917	0.68918	0.73676	0.66113	0.66830	0.62429								
512	0.33190	0.40919	0.51820	0.40067	0.55392	0.58942	0.49758	0.51603									
256	0.24323	0.30903	0.41458	0.31600	0.44848	0.47679	0.40891										
128	0.15268	0.21588	0.32447	0.21588	0.34541	0.38091											
64	0.11621	0.16839	0.28560	0.15853	0.30183												
32	0.09673	0.14890	0.26490	0.13950													
16	0.25686	0.27573	0.46749														
8	0.54325	0.58305															
4	0.97749																

(f) $\mathcal{P}^{\mathcal{C}}$ for Scene: [**Torus**] Algorithm: [**ERPT**] Metric: [**Contourlet**] True GT: [**BDPT @ 524288 spp**].

M-ERPT	2	4	8	16	32	64	128	256	512	1024	2048	4096	8192	16384	32768	65536	131072
GT (BDPT)																	
262144	0.04360	0.02245	0.02388	0.02916	0.04213	0.03231	0.02432	0.04169	0.03980	0.04813	0.02790	0.04466	0.05116	0.04361	0.06377	0.04310	0.04361
131072	0.10932	0.09111	0.09737	0.09182	0.15964	0.10100	0.11256	0.10327	0.12751	0.11896	0.10141	0.12518	0.12204	0.14085	0.13198	0.15188	
65536	0.20373	0.21000	0.20378	0.20318	0.28886	0.25570	0.24985	0.23382	0.28438	0.31782	0.27488	0.33928	0.33991	0.34858	0.32003		
32768	0.36986	0.37614	0.42015	0.37028	0.52891	0.46896	0.48037	0.46113	0.57681	0.47960	0.45727	0.61068	0.58458	0.61998			
16384	0.63072	0.63699	0.80122	0.64970	0.92424	0.91089	0.94087	0.92822	1.08038	0.91207	0.93126	0.88549	0.96195				
8192	0.85561	0.97000	1.02311	1.03255	0.89908	0.88671	1.04382	0.95445	0.85440	0.93830	0.80506	0.59409					
4096	0.74789	0.84246	0.90192	0.96275	0.69276	0.79870	0.81334	0.71436	0.62247	0.73520	0.59637						
2048	0.74789	0.77075	0.71561	0.82622	0.55522	0.56703	0.64888	0.55920	0.48336	0.49169							
1024	0.58059	0.59895	0.51875	0.62525	0.42475	0.43656	0.51208	0.42873	0.37383								
512	0.42749	0.41657	0.36944	0.47214	0.28886	0.27020	0.37085	0.26980									
256	0.33990	0.31807	0.28220	0.38455	0.19017	0.18340	0.27553										
128	0.22550	0.22075	0.18755	0.28148	0.11491	0.09605											
64	0.17305	0.19438	0.14713	0.23276	0.05569												
32	0.15379	0.16007	0.12832	0.20796													
16	0.30503	0.31807	0.25652														
8	0.60505	0.58687															
4	1.09529																

(g) $\mathcal{P}^{\mathcal{C}}$ for Scene: [**Torus**] Algorithm: [**M-ERPT**] Metric: [**Contourlet**] True GT: [**BDPT @ 524288 spp**].

Table 7

3.3 Veach Bidir

PT	2	4	8	16	32	64	128	256	512	1024	2048	4096	8192	16384	32768	65536	131072
GT (BDPT)																	
262144	-0.00004	0.00009	-0.00005	-0.00007	-0.00009	0.00000	0.00015	0.00022	-0.00068	-0.00111	-0.00163	0.00000	-0.00471	-0.00783	-0.01659	-0.03822	0.00000
131072	0.00008	0.00027	0.00048	0.00034	0.00060	0.00068	0.00124	0.00135	0.00068	0.00111	0.00164	0.00268	-0.00236	0.00000	0.00852	-0.01943	
65536	0.00031	0.00081	0.00081	0.00108	0.00146	0.00192	0.00280	0.00294	0.00240	0.00223	0.00412	0.00675	0.00000	-0.00394	-0.00837		
32768	0.00035	0.00086	0.00092	0.00115	0.00163	0.00215	0.00311	0.00316	0.00274	0.00336	0.00579	0.00811	0.00238	0.00398			
16384	0.00078	0.00108	0.00162	0.00203	0.00275	0.00329	0.00437	0.00545	0.00482	0.00505	0.00579	0.00811	0.00718				
8192	0.00268	0.00364	0.00524	0.00643	0.00874	0.01003	0.01256	0.01703	0.01730	0.02265	0.02749	0.03085					
4096	0.00368	0.00488	0.00718	0.00930	0.01218	0.01373	0.01720	0.02243	0.02399	0.03192	0.03948						
2048	0.00683	0.00938	0.01322	0.01726	0.02248	0.02605	0.03233	0.04163	0.04816	0.06044							
1024	0.00662	0.00915	0.01293	0.01703	0.02239	0.02579	0.03215	0.04136	0.04526								
512	0.01087	0.01464	0.02044	0.02705	0.03491	0.04068	0.05157	0.06621									
256	0.01725	0.02308	0.03223	0.04215	0.05345	0.06361	0.08071										
128	0.02891	0.03914	0.05422	0.07226	0.09193	0.10792											
64	0.04692	0.06368	0.08682	0.11670	0.14607												
32	0.07991	0.10763	0.14686	0.19479													
16	0.13226	0.17705	0.24139														
8	0.20939	0.28582															
4	0.31586																

(a) $\mathcal{P}^{\mathcal{C}}$ for Scene: [**Veach Bidir**] Algorithm: [**PT**] Metric: [**Contourlet**] True GT: [**BDPT @ 524288 spp**].

BDPT	2	4	8	16	32	64	128	256	512	1024	2048	4096	8192	16384	32768	65536	131072
GT (BDPT)																	
262144	0.00011	-0.00020	0.00000	-0.00166	-0.01405	-0.04934	-0.05140	-0.07544	-0.05859	0.00000	0.00000	-0.07143	0.08481	0.00000	-0.09030	0.04370	0.00000
131072	0.00075	0.00141	0.00049	0.00334	0.00000	-0.02516	0.00000	-0.03878	0.00000	0.00000	0.04111	-0.03667	0.04111	-0.04111	-0.13141	0.04370	
65536	0.00205	0.00427	0.00539	0.01012	0.01450	-0.04934	0.08597	-0.03878	-0.02997	0.00000	-0.07544	-0.07143	0.00000	-0.04111	-0.09030		
32768	0.00226	0.00427	0.00638	0.01356	0.02948	-0.04934	0.15042	0.00000	0.03143	0.07143	-0.07544	0.00000	0.00000	-0.04111			
16384	0.00390	0.00571	0.00938	0.02057	0.00000	-0.09496	0.02738	-0.11020	0.13587	0.11020	-0.14322	-0.03667	-0.03878				
8192	0.01080	0.01870	0.03255	0.05407	0.06099	0.00000	0.18517	-0.11020	0.09921	0.19501	0.04111	0.07988					
4096	0.01528	0.02561	0.04267	0.07635	0.08611	-0.02516	0.08597	-0.20462	0.06445	0.07143	0.00000						
2048	0.02872	0.04676	0.07957	0.11866	0.12181	-0.02516	-0.07557	-0.23324	0.03143	0.00000							
1024	0.02847	0.04601	0.07825	0.11630	0.15044	0.00000	0.02738	-0.14322	0.03143								
512	0.04583	0.07236	0.12634	0.20102	0.25997	0.11220	0.08597	-0.11020									
256	0.07134	0.11429	0.19652	0.30344	0.38343	0.05361	-0.02623										
128	0.12285	0.20069	0.34007	0.53433	0.65519	0.17665											
64	0.19823	0.33500	0.57719	0.84026	0.79255												
32	0.35427	0.62149	1.05395	1.23559													
16	0.64312	1.07227	1.63747														
8	1.20009	1.84057															
4	1.94101																

(b) $\mathcal{P}^{\mathcal{C}}$ for Scene: [**Veach Bidir**] Algorithm: [**BDPT**] Metric: [**Contourlet**] True GT: [**BDPT @ 524288 spp**].

PSSMLT	2	4	8	16	32	64	128	256	512	1024	2048	4096	8192	16384
GT (BDPT)														
262144	-0.00020	-0.00019	-0.00086	-0.00131	-0.01117	-0.03368	0.00000	0.05140	0.00000	0.00000	0.00000	0.00000	0.00000	0.00000
131072	0.00071	0.00112	0.00086	0.00132	0.00000	0.03582	0.03302	0.02516	-0.05140	-0.02862	-0.02738	0.00000	-0.07258	-0.05361
65536	0.00164	0.00300	0.00302	0.00801	0.00000	0.01763	-0.09002	0.05140	-0.02623	0.06139	0.02862	0.11740	0.05361	0.00000
32768	0.00184	0.00338	0.00389	0.01072	0.01147	0.05462	-0.09002	0.05140	0.00000	0.06139	0.02862	0.05600	0.00000	0.00000
16384	0.00277	0.00451	0.00651	0.00936	0.01147	0.00000	-0.11740	-0.02417	0.02738	0.09441	0.02862	0.11740	0.05361	
8192	0.01026	0.01583	0.02288	0.03779	0.05422	0.03582	0.00000	0.07878	0.08597	0.06139	-0.05361	0.18517		
4096	0.01362	0.02152	0.03295	0.04993	0.07390	0.05462	0.03302	0.07878	0.15042	0.12917	0.09002			
2048	0.02581	0.04066	0.06033	0.09829	0.15449	0.01763	-0.06139	0.10740	0.05600	-0.05600				
1024	0.02535	0.03977	0.06033	0.09829	0.17079	0.03582	-0.03143	0.20181	0.02738					
512	0.04081	0.06240	0.09973	0.16811	0.24229	0.11495	0.00000	0.16879						
256	0.06342	0.09755	0.15451	0.28246	0.34947	0.20632	-0.09002							
128	0.10827	0.16988	0.27994	0.53173	0.66539	0.25772								
64	0.17646	0.28144	0.48809	0.80847	0.92556									
32	0.30884	0.52031	0.89969	1.23538										
16	0.53217	0.94906	1.47702											
8	0.96586	1.68706												
4	1.62758													

(c) $\mathcal{P}^{\mathcal{C}}$ for Scene: [**Veach Bidir**] Algorithm: [**PSSMLT**] Metric: [**Contourlet**] True GT: [**BDPT @ 524288 spp**].

MLT	2	4	8	16	32	64	128	256	512	1024	2048	4096	8192	16384
GT (BDPT)														
262144	-0.00010	-0.00012	0.00000	0.00000	-0.00054	-0.00066	-0.00419	-0.01342	-0.01610	-0.04563	0.00000	-0.06139	-0.05600	0.05361
131072	0.00010	0.00018	0.00017	0.00070	0.00027	0.00267	0.00000	-0.00676	0.01659	0.00000	0.00000	0.00000	0.00000	0.00000
65536	0.00063	0.00090	0.00154	0.00210	0.00218	0.00536	0.00000	-0.01998	-0.01610	-0.06720	0.00000	-0.06139	0.02997	0.02623
32768	0.00072	0.00102	0.00145	0.00238	0.00246	0.00604	0.00211	-0.01998	0.01659	-0.02325	0.05859	0.00000	-0.02862	-0.02516
16384	0.00116	0.00162	0.00231	0.00336	0.00633	0.00740	0.00854	-0.01998	-0.03175	-0.06720	-0.05361	-0.06139	0.02997	
8192	0.00379	0.00522	0.00817	0.01218	0.01779	0.03092	0.03785	0.01383	0.05131	-0.02325	0.00000	0.06778		
4096	0.00512	0.00739	0.01113	0.01726	0.02525	0.04078	0.05226	0.02811	0.03368	0.02417	0.05859			
2048	0.00964	0.01393	0.02057	0.03152	0.04587	0.07791	0.10225	0.05808	0.06950	-0.02325				
1024	0.00943	0.01367	0.02029	0.03120	0.04554	0.07968	0.10225	0.07383	0.06950					
512	0.01506	0.02207	0.03313	0.04966	0.07180	0.12071	0.16693	0.13355						
256	0.02377	0.03446	0.05163	0.07563	0.11116	0.18084	0.24508							
128	0.03963	0.05876	0.08768	0.12870	0.18996	0.31369								
64	0.06334	0.09358	0.13774	0.20726	0.31628									
32	0.10625	0.15763	0.23207	0.35279										
16	0.17264	0.25700	0.38167											
8	0.27012	0.40441												
4	0.40326													

(d) $\mathcal{P}^{\mathcal{C}}$ for Scene: [**Veach Bidir**] Algorithm: [**MLT**] Metric: [**Contourlet**] True GT: [**BDPT @ 524288 spp**].

M-MLT	2	4	8	16	32	64	128	256	512	1024	2048	4096	8192	16384
GT (BDPT)														
262144	-0.00005	-0.00006	0.00009	-0.00030	0.00000	-0.00147	-0.00250	-0.01405	-0.01943	-0.03952	0.00000	0.00000	0.05361	0.02997
131072	0.00014	0.00037	0.00078	0.00059	0.00142	0.00148	0.00251	0.00000	0.02010	0.00000	0.02738	0.09441	0.02623	0.02997
65536	0.00063	0.00124	0.00173	0.00254	0.00313	0.00371	0.00251	-0.00708	-0.05641	-0.02010	-0.02623	0.00000	0.05361	0.09441
32768	0.00063	0.00136	0.00182	0.00284	0.00370	0.00446	0.00760	0.00719	-0.01943	0.02081	-0.02623	0.00000	0.05361	0.02997
16384	0.00107	0.00198	0.00278	0.00389	0.00543	0.00823	0.01017	0.00719	-0.01943	-0.02010	-0.07557	0.00000	0.11220	
8192	0.00366	0.00570	0.00784	0.01317	0.01914	0.02871	0.03973	0.07759	0.02010	0.00000	0.11740	0.06139		
4096	0.00500	0.00755	0.01110	0.01797	0.02631	0.04212	0.05989	0.09479	0.08486	0.06476	0.15042			
2048	0.00948	0.01436	0.02115	0.03291	0.04718	0.07486	0.11402	0.16038	0.08486	0.02081				
1024	0.00943	0.01396	0.02096	0.03273	0.04649	0.07486	0.12094	0.18086	0.04091					
512	0.01513	0.02283	0.03412	0.05202	0.07549	0.12300	0.20221	0.24789						
256	0.02399	0.03574	0.05378	0.07922	0.11759	0.19748	0.30989							
128	0.04080	0.06063	0.09008	0.13482	0.20872	0.35103								
64	0.06486	0.09701	0.14436	0.21212	0.33216									
32	0.10934	0.16087	0.24523	0.37260										
16	0.17862	0.26506	0.40266											
8	0.27680	0.42779												
4	0.41956													

(e) $\mathcal{P}^{\mathcal{C}}$ for Scene: [**Veach Bidir**] Algorithm: [**M-MLT**] Metric: [**Contourlet**] True GT: [**BDPT @ 524288 spp**].

ERPT	2	4	8	16	32	64	128	256	512	1024	2048	4096	8192	16384	32768	65536	131072
GT (BDPT)																	
262144	0.00321	-0.00602	-0.00588	-0.00300	-0.00592	-0.00267	-0.00578	-0.00293	-0.00531	-0.00569	-0.00560	-0.00323	-0.00288	-0.00543	-0.00548	-0.00520	-0.00535
131072	0.00644	0.00305	0.00000	0.00000	0.00300	0.00000	-0.00290	0.00295	0.00269	-0.00286	-0.00281	0.00326	0.00290	0.00275	0.00000	0.00527	
65536	0.00000	0.00305	0.00597	0.00607	-0.00297	0.00269	-0.00290	0.00000	0.00000	0.00000	0.00569	0.00000	0.00000	0.00552	0.00000		
32768	0.00644	0.00922	0.01204	0.01224	0.00300	0.00539	0.00293	0.00592	0.00539	0.00578	0.01147	0.00655	0.00583	0.01112			
16384	0.00644	0.00612	0.00899	0.00914	0.00602	0.01087	0.00000	0.00892	0.01364	-0.00569	0.01440	0.00655	0.00583				
8192	0.05123	0.03834	0.05072	0.04816	0.05116	0.05481	0.03999	0.03706	0.06108	0.03933	0.05802	0.05592					
4096	0.06997	0.05205	0.06809	0.06569	0.05807	0.07070	0.04986	0.06052	0.07397	0.05900	0.07840						
2048	0.10195	0.09250	0.10933	0.11951	0.11033	0.10825	0.09969	0.11228	0.13423	0.10548							
1024	0.10614	0.10033	0.11330	0.11541	0.11435	0.11186	0.09969	0.11228	0.13809								
512	0.17483	0.16929	0.18780	0.19157	0.19457	0.19203	0.19368	0.18597									
256	0.23364	0.21922	0.27888	0.26112	0.30687	0.27556	0.29078										
128	0.47997	0.42579	0.48837	0.48239	0.53508	0.44670											
64	0.74471	0.64621	0.68219	0.69025	0.75330												
32	0.96054	0.95597	0.98821	0.95899													
16	1.18652	1.31540	1.24895														
8	1.25795	1.23996															
4	1.18652																

(f) $\mathcal{P}^{\mathcal{C}}$ for Scene: [**Veach Bidir**] Algorithm: [**ERPT**] Metric: [**Contourlet**] True GT: [**BDPT @ 524288 spp**].

M-ERPT	2	4	8	16	32	64	128	256	512	1024	2048	4096	8192	16384	32768	65536	131072
GT (BDPT)																	
262144	-0.00275	-0.00512	-0.00267	-0.00257	-0.00531	-0.00539	-0.00531	-0.00288	-0.00273	-0.00539	-0.00501	-0.00248	-0.00501	-0.00273	-0.00508	-0.00484	-0.00458
131072	0.00277	0.00000	0.00000	0.00259	-0.00267	-0.00271	0.00000	0.00583	0.00000	0.00273	0.00253	0.00000	-0.00251	0.00552	0.00000	0.00000	
65536	-0.00548	-0.00512	0.00539	0.00000	0.00269	0.00000	0.00269	0.00583	0.00000	0.00548	0.00508	0.01010	0.00508	-0.00273	0.00516		
32768	0.00000	0.00000	0.00812	0.00520	0.00539	0.00548	0.00812	0.01175	0.00552	0.01104	0.01024	0.01525	0.01024	0.00275			
16384	0.00556	0.00520	0.00269	0.00782	0.01364	0.00824	0.01087	0.00878	0.00275	0.00824	0.01024	0.01267	0.01024				
8192	0.05984	0.05570	0.04256	0.04678	0.04256	0.04019	0.04256	0.04945	0.04669	0.05254	0.04003	0.04504					
4096	0.05984	0.07422	0.06108	0.06484	0.06426	0.05569	0.05171	0.05950	0.06258	0.06857	0.05443						
2048	0.11576	0.10730	0.10825	0.10045	0.10114	0.09575	0.09072	0.11417	0.12229	0.10286							
1024	0.11201	0.11428	0.11186	0.10386	0.10468	0.10286	0.09072	0.11417	0.12229								
512	0.19014	0.19205	0.17891	0.19205	0.17463	0.18663	0.16621	0.18239									
256	0.27572	0.29972	0.24919	0.28328	0.25954	0.24380	0.24919										
128	0.52363	0.54763	0.47989	0.45983	0.44670	0.46598											
64	0.72194	0.76035	0.79287	0.69194	0.76201												
32	1.06327	1.14089	1.10037	1.03794													
16	1.31135	1.29868	1.12775														
8	1.27468	1.41523															
4	1.27468																

(g) $\mathcal{P}^{\mathcal{C}}$ for Scene: [**Veach Bidir**] Algorithm: [**M-ERPT**] Metric: [**Contourlet**] True GT: [**BDPT @ 524288 spp**].

Table 8

3.4 Veach Door

PT	2	4	8	16	32	64	128	256	512	1024	2048	4096	8192	16384	32768	65536	131072
GT (BDPT)																	
262144	0.00032	0.00025	0.00015	0.00040	0.00078	0.00077	0.00089	0.00606	0.00205	0.00562	0.02092	0.03472	0.02623	0.06445	0.11020	-0.03302	0.03667
131072	0.00057	0.00057	0.00030	0.00069	0.00109	0.00077	0.00179	0.00693	0.00619	0.02889	0.02092	0.07171	0.11220	0.06445	0.00000	0.00000	
65536	0.00076	0.00076	0.00044	0.00119	0.00203	0.00206	0.00269	0.00606	0.00411	0.03491	0.02092	0.07171	0.14363	0.09921	0.00000		
32768	0.00196	0.00172	0.00215	0.00289	0.00408	0.00544	0.00677	0.01314	0.01249	0.02889	0.04275	0.07171	0.05361	0.13587			
16384	0.00299	0.00319	0.00343	0.00572	0.00712	0.01048	0.01327	0.02133	0.02545	0.04720	0.01035	0.07171	0.08223				
8192	0.00603	0.00598	0.00738	0.01109	0.01517	0.02064	0.02779	0.03944	0.05777	0.07287	0.08946	0.11123					
4096	0.01018	0.01083	0.01292	0.01965	0.02741	0.03452	0.04693	0.06839	0.10150	0.11440	0.06557						
2048	0.01837	0.02035	0.02511	0.03538	0.05086	0.06784	0.09331	0.13109	0.18754	0.22901							
1024	0.03313	0.03665	0.04546	0.06558	0.09109	0.12697	0.17971	0.24067	0.31299								
512	0.05497	0.06132	0.07863	0.11175	0.15829	0.22682	0.32147	0.43686									
256	0.08705	0.09802	0.12762	0.18359	0.26483	0.40116	0.56134										
128	0.13289	0.15052	0.20423	0.29750	0.45754	0.67949											
64	0.19881	0.22846	0.31762	0.49412	0.83094												
32	0.29045	0.34951	0.50116	0.83298													
16	0.44492	0.55195	0.83842														
8	0.67169	0.86614															
4	1.00984																

(a) $\mathcal{P}^{\mathcal{C}}$ for Scene: [**Veach Door**] Algorithm: [**PT**] Metric: [**Contourlet**] True GT: [**BDPT @ 524288 spp**].

BDPT	2	4	8	16	32	64	128	256	512	1024	2048	4096	8192	16384	32768	65536	131072
GT (BDPT)																	
262144	0.00026	0.00045	0.00032	0.00053	0.00113	0.00304	0.00899	0.01602	0.04696	0.04395	0.03143	-0.09441	-0.03667	-0.03667	-0.03667	0.07988	-0.11020
131072	0.00046	0.00060	0.00074	0.00142	0.00226	0.00304	0.01512	0.02425	0.08064	0.04395	0.00000	-0.03302	-0.03667	0.03878	0.03878	0.00000	
65536	0.00078	0.00106	0.00106	0.00195	0.00454	0.00818	0.01822	0.01602	0.04696	0.00000	0.06445	-0.09441	-0.13587	-0.10445	0.00000		
32768	0.00223	0.00243	0.00309	0.00464	0.00801	0.01447	0.02767	0.04114	0.04696	0.00000	0.03143	0.00000	0.00000	-0.03667			
16384	0.00355	0.00412	0.00536	0.00827	0.01509	0.02307	0.03738	0.04982	0.04696	0.00000	0.03143	0.07143	-0.10445				
8192	0.00643	0.00801	0.01032	0.01746	0.02738	0.04340	0.05757	0.08620	0.09827	0.14277	-0.02997	-0.12303					
4096	0.01142	0.01385	0.01900	0.03152	0.04843	0.07937	0.08629	0.08620	0.06355	0.14277	0.03143						
2048	0.02075	0.02612	0.03643	0.05805	0.08966	0.15507	0.17361	0.25304	0.23955	0.26016							
1024	0.03756	0.04723	0.06754	0.10618	0.16487	0.28245	0.32286	0.40389	0.33836								
512	0.06264	0.08090	0.11523	0.18469	0.28767	0.44360	0.61717	0.66161									
256	0.10039	0.13059	0.18944	0.31319	0.51555	0.81060	1.18118										
128	0.15609	0.20773	0.31213	0.52448	0.80642	1.19167											
64	0.23729	0.32608	0.52699	0.93718	1.45076												
32	0.35737	0.51193	0.92045	1.45947													
16	0.58781	0.90684	1.56198														
8	0.93606	1.50285															
4	1.46787																

(b) $\mathcal{P}^{\mathcal{C}}$ for Scene: [**Veach Door**] Algorithm: [**BDPT**] Metric: [**Contourlet**] True GT: [**BDPT @ 524288 spp**].

PSSMLT	2	4	8	16	32	64	128	256	512	1024	2048	4096	8192	16384
GT (BDPT)														
262144	0.00043	0.00034	0.00150	0.00060	0.00311	0.00390	0.00808	0.01366	0.02325	-0.02417	-0.02862	-0.03302	-0.03143	-0.07143
131072	0.00087	0.00134	0.00150	0.00363	0.00625	0.00390	0.01630	0.01366	0.00000	0.00000	0.00000	0.00000	0.10445	0.00000
65536	0.00119	0.00202	0.00332	0.00363	0.00783	0.00000	0.00000	0.00000	-0.06476	0.00000	-0.02862	-0.03302	0.10445	0.00000
32768	0.00272	0.00509	0.00546	0.00918	0.01586	0.01582	0.01630	0.01366	-0.04395	0.02516	0.00000	-0.03302	0.14322	0.03878
16384	0.00526	0.00873	0.01012	0.01548	0.02078	0.02813	0.00808	0.01366	-0.04395	0.00000	0.02997	0.07143	-0.06139	
8192	0.00999	0.01547	0.01939	0.03058	0.04300	0.07212	0.04187	0.04209	-0.02238	-0.02417	0.12917	0.03476		
4096	0.01872	0.02754	0.03700	0.05312	0.07455	0.09115	0.05071	0.07210	-0.10429	0.02516	-0.08223			
2048	0.03459	0.04840	0.06854	0.09769	0.15070	0.20197	0.11760	0.27405	0.00000	0.05140				
1024	0.06208	0.08880	0.12723	0.19322	0.25786	0.35049	0.25842	0.23167	0.15482					
512	0.10669	0.15585	0.22074	0.33202	0.45404	0.54072	0.57810	0.54567						
256	0.17310	0.26075	0.38035	0.58513	0.82557	0.88671	0.77107							
128	0.27855	0.42533	0.64096	0.92609	1.12068	0.98966								
64	0.45476	0.70854	1.07123	1.43389	1.50611									
32	0.74350	1.19010	1.56132	1.77693										
16	1.22562	2.07355	1.98552											
8	1.96667	1.90771												
4	1.96667													

(c) $\mathcal{P}^{\mathcal{C}}$ for Scene: [**Veach Door**] Algorithm: [**PSSMLT**] Metric: [**Contourlet**] True GT: [**BDPT @ 524288 spp**].

MLT	2	4	8	16	32	64	128	256	512	1024	2048	4096	8192	16384
GT (BDPT)														
262144	0.00055	0.00035	0.00111	0.00242	0.00385	0.00746	0.00719	0.01181	0.01943	0.05361	0.14322	0.03143	-0.03476	0.03476
131072	0.00088	0.00105	0.00148	0.00323	0.00579	0.00746	0.00719	0.03626	0.03952	0.08223	0.00000	0.03143	-0.03476	-0.03302
65536	0.00121	0.00211	0.00335	0.00568	0.00385	0.00746	0.00719	0.01181	0.00000	0.08223	0.00000	0.09921	0.00000	0.11020
32768	0.00265	0.00352	0.00674	0.00899	0.01169	0.01506	0.01450	0.03626	0.00000	0.17665	0.06778	0.09921	0.00000	0.07143
16384	0.00533	0.00639	0.01251	0.01656	0.01368	0.01124	0.02948	0.03626	-0.01879	0.05361	-0.06139	0.03143	-0.12917	
8192	0.01023	0.01391	0.02488	0.03508	0.03424	0.02675	0.07759	0.06193	0.06034	0.11220	0.10445	0.09921		
4096	0.01788	0.02561	0.04316	0.05587	0.06530	0.05123	0.07759	0.06193	0.03952	0.05361	-0.09002			
2048	0.03492	0.04871	0.07820	0.10839	0.11782	0.13002	0.24789	0.14735	0.23048	0.08223				
1024	0.06342	0.08912	0.13741	0.20255	0.20929	0.24028	0.39908	0.24860	0.12754					
512	0.10521	0.15194	0.23498	0.32257	0.34436	0.35220	0.65519	0.41910						
256	0.17146	0.24891	0.39836	0.51125	0.58650	0.77810	0.89176							
128	0.27147	0.40250	0.65326	0.76372	0.89235	1.06717								
64	0.44011	0.67469	1.06458	1.23730	1.36592									
32	0.71948	1.10207	1.47327	1.53339										
16	1.20939	1.70022	1.89822											
8	1.79412	1.99340												
4	1.93689													

(d) $\mathcal{P}^{\mathcal{C}}$ for Scene: [**Veach Door**] Algorithm: [**MLT**] Metric: [**Contourlet**] True GT: [**BDPT @ 524288 spp**].

M-MLT	2	4	8	16	32	64	128	256	512	1024	2048	4096	8192	16384
GT (BDPT)														
262144	0.00032	0.00092	0.00078	0.00086	0.00195	0.00842	0.00768	0.04566	-0.02157	0.15042	0.03302	0.09002	0.11020	-0.13587
131072	0.00065	0.00147	0.00117	0.00347	0.00195	0.00842	0.01549	0.07835	0.00000	0.08597	0.06778	0.05859	0.15131	-0.03667
65536	0.00163	0.00257	0.00273	0.00434	0.00195	0.00419	0.01549	0.06176	-0.04238	0.05600	0.00000	0.09002	0.11020	0.03878
32768	0.00338	0.00480	0.00510	0.00875	0.00988	0.01702	0.04810	0.09544	-0.08191	0.18517	-0.03143	0.05859	0.03476	0.00000
16384	0.00570	0.00800	0.01111	0.01595	0.01596	0.01702	0.03151	0.07835	0.04563	0.02738	0.00000	0.02862	-0.06445	
8192	0.01031	0.01534	0.02276	0.03381	0.04141	0.03480	0.09233	0.15006	0.04563	0.11740	0.06778	0.15779		
4096	0.01761	0.02787	0.04043	0.05287	0.05489	0.05339	0.12096	0.09544	0.04563	0.08597	0.06778			
2048	0.03489	0.05278	0.07790	0.10740	0.11202	0.13169	0.23049	0.30176	0.17720	0.02738				
1024	0.06266	0.09484	0.13612	0.19977	0.20006	0.22049	0.35395	0.27759	0.27161					
512	0.10730	0.16029	0.24510	0.33458	0.35345	0.47546	0.55590	0.43913						
256	0.17428	0.26743	0.40116	0.57749	0.63429	0.81296	0.86228							
128	0.28376	0.45206	0.65499	0.90212	0.88656	0.90792								
64	0.46313	0.76404	1.08923	1.43202	1.20971									
32	0.75424	1.27756	1.61325	1.68010										
16	1.31924	1.98411	1.84982											
8	2.05711	1.95109												
4	1.81852													

(e) $\mathcal{P}^{\mathcal{C}}$ for Scene: [**Veach Door**] Algorithm: [**M-MLT**] Metric: [**Contourlet**] True GT: [**BDPT @ 524288 spp**].

ERPT	2	4	8	16	32	64	128	256	512	1024	2048	4096	8192	16384	32768	65536	131072
GT (BDPT)																	
262144	0.00697	0.01689	0.00884	0.00993	0.02505	0.02183	0.03171	0.00993	0.01080	0.01035	0.01890	0.00973	0.00954	0.02007	0.00936	0.02282	0.02980
131072	0.01405	0.02557	0.00884	0.02007	0.02505	0.02183	0.04275	0.00993	0.03311	0.01035	0.01890	0.01966	0.02921	0.02007	0.02863	0.03463	
65536	0.01405	0.00837	0.01785	0.02007	0.03804	0.00000	0.03171	0.00993	0.02183	0.02092	0.00936	0.01966	0.01928	0.02007	0.02863		
32768	0.02855	0.04342	0.02703	0.04098	0.03804	0.02183	0.07738	0.00000	0.02183	0.01035	0.00936	0.02980	0.03934	0.05178			
16384	0.02124	0.06195	0.00000	0.03042	0.02505	0.03311	0.05403	0.00993	0.01080	0.03171	0.00000	0.04015	0.02921				
8192	0.06696	0.07150	0.06559	0.06282	0.10825	0.03311	0.10183	0.04098	0.03311	0.10183	0.03856	0.07255					
4096	0.08327	0.06195	0.07573	0.08564	0.06503	0.05646	0.14082	0.05178	0.02183	0.11451	0.04870						
2048	0.19491	0.15533	0.14130	0.31605	0.24351	0.19200	0.24467	0.20298	0.14759	0.22857							
1024	0.28640	0.29901	0.28865	0.48057	0.30384	0.27506	0.41488	0.35304	0.25744								
512	0.55705	0.47379	0.56040	0.64211	0.37104	0.63255	0.59208	0.50474									
256	0.77664	0.69337	0.69777	0.45732	0.44661	0.54254	0.50984										
128	0.80660	0.66475	0.61180	0.55614	0.34780	0.51515											
64	0.72063	0.51617	0.51298	0.39256	0.26293												
32	0.64507	0.56180	0.53623	0.45732													
16	0.57787	0.45369	0.42812														
8	0.51753	0.45369															
4	0.42923																

(f) $\mathcal{P}^{\mathcal{C}}$ for Scene: [**Veach Door**] Algorithm: [**ERPT**] Metric: [**Contourlet**] True GT: [**BDPT @ 524288 spp**].

M-ERPT	2	4	8	16	32	64	128	256	512	1024	2048	4096	8192	16384	32768	65536	131072
GT (BDPT)																	
262144	0.00794	0.00918	0.01057	0.02007	0.01128	0.00973	0.01819	0.00954	0.00901	0.01057	0.02137	0.00936	0.00901	0.00973	0.00936	0.01057	0.01128
131072	0.00794	0.00918	0.02137	0.04098	0.02282	0.00973	0.02754	0.01928	0.01819	0.01057	0.04368	0.01890	0.00901	0.01966	0.01890	0.02137	
65536	0.00794	0.01854	0.03240	0.03042	0.01128	0.01966	0.01819	0.00954	0.00901	0.01057	0.03240	0.01890	0.01819	0.00973	0.01890		
32768	0.03262	0.00918	0.05522	0.03042	0.02282	0.04015	0.05675	0.01928	0.00901	0.01057	0.02137	0.03856	0.04682	0.02980			
16384	0.03262	0.00918	0.05522	0.03042	0.00000	0.05072	0.06689	0.02921	0.03709	0.00000	0.03240	0.02863	0.03709				
8192	0.06766	0.05788	0.06703	0.10953	0.04671	0.04015	0.09860	0.07106	0.06689	0.04368	0.05522	0.08042					
4096	0.08620	0.06823	0.09149	0.13458	0.07176	0.06152	0.12092	0.10491	0.10964	0.10416	0.04368						
2048	0.19111	0.21237	0.26800	0.26474	0.18582	0.19831	0.27981	0.19383	0.23540	0.20257							
1024	0.30846	0.28645	0.34205	0.37246	0.30964	0.32578	0.41928	0.31769	0.32814								
512	0.56280	0.71135	0.67614	0.58352	0.57929	0.62188	0.57673	0.54918									
256	0.74758	0.59395	0.55310	0.58352	0.57929	0.59325	0.55156										
128	0.71762	0.49514	0.58173	0.50474	0.46709	0.56587											
64	0.63538	0.43037	0.38296	0.35304	0.37213												
32	0.63538	0.51838	0.45016	0.50474													
16	0.51885	0.41027	0.36214														
8	0.45851	0.37206															
4	0.37021																

(g) $\mathcal{P}^{\mathcal{C}}$ for Scene: [**Veach Door**] Algorithm: [**M-ERPT**] Metric: [**Contourlet**] True GT: [**BDPT @ 524288 spp**].

Table 9

3.5 Sponza

PT	2	4	8	16	32	64	128	256	512	1024	2048	4096	8192
**GT (PT)**													
16384	0.00144	0.00189	0.00227	0.00327	0.00485	0.00689	0.01702	0.02540	0.02920	0.08597	0.10445	0.07544	-0.03476
8192	0.00401	0.00519	0.00720	0.01010	0.01467	0.02474	0.05114	0.07540	0.07616	0.15042	0.10445	0.07544	
4096	0.00838	0.01132	0.01605	0.02292	0.03295	0.05782	0.09629	0.15965	0.20398	0.22184	0.14322		
2048	0.01779	0.02258	0.03233	0.04667	0.06926	0.11631	0.19668	0.30037	0.29199	0.30173			
1024	0.03467	0.04495	0.06247	0.09311	0.13953	0.23646	0.40215	0.54309	0.36756				
512	0.06390	0.08162	0.11180	0.16876	0.26395	0.45866	0.75060	0.84001					
256	0.11269	0.14559	0.19792	0.30974	0.50959	0.91329	1.49497						
128	0.18385	0.24110	0.33936	0.53924	0.92000	1.48418							
64	0.29108	0.39401	0.58617	1.02226	1.61169								
32	0.47005	0.66848	1.10074	1.81820									
16	0.76781	1.18190	1.91469										
8	1.26225	1.87420											
4	1.91801												

(a) $\mathcal{P}^{\mathcal{C}}$ for Scene: [**Sponza**] Algorithm: [**PT**] Metric: [**Contourlet**] True GT: [**PT @ 32768 spp**].

BDPT	2	4	8	16	32	64	128	256	512	1024	2048	4096	8192
**GT (PT)**													
16384	0.00126	0.00150	0.00232	0.00325	0.00469	0.00651	0.00967	0.01737	0.01752	0.06980	0.05361	0.03302	-0.10445
8192	0.00366	0.00477	0.00609	0.00877	0.01310	0.01939	0.03883	0.07066	0.10532	-0.02157	0.17665	0.03302	
4096	0.00806	0.01040	0.01325	0.01889	0.02885	0.04359	0.08144	0.12289	0.21191	0.14858	0.08223		
2048	0.01625	0.02092	0.02769	0.03984	0.05798	0.09834	0.16852	0.25912	0.28211	0.30637			
1024	0.03288	0.04043	0.05393	0.07778	0.11692	0.19382	0.34132	0.47699	0.52166				
512	0.05872	0.07228	0.09854	0.14130	0.21787	0.36357	0.65493	0.80996					
256	0.10193	0.12772	0.17543	0.25535	0.40056	0.72452	1.19967						
128	0.16595	0.21227	0.29719	0.43930	0.75205	1.32781							
64	0.26283	0.34643	0.49929	0.79069	1.38609								
32	0.42101	0.57409	0.88391	1.46546									
16	0.66452	0.97637	1.54974										
8	1.07298	1.56426											
4	1.62734												

(b) $\mathcal{P}^{\mathcal{C}}$ for Scene: [**Sponza**] Algorithm: [**BDPT**] Metric: [**Contourlet**] True GT: [**PT @ 32768 spp**].

PSSMLT	2	4	8	16	32	64	128	256	512	1024	2048	4096	8192
**GT (PT)**													
16384	0.00201	0.00247	0.00237	0.00381	0.00581	0.00683	0.01343	0.01330	0.02304	0.04153	0.01575	0.00852	0.01659
8192	0.00562	0.00690	0.00875	0.01092	0.01593	0.02201	0.03536	0.04900	0.06472	0.08724	0.09401	0.04423	
4096	0.01225	0.01456	0.01911	0.02514	0.03551	0.04820	0.07228	0.10889	0.13666	0.16550	0.13335		
2048	0.02481	0.02950	0.03971	0.05180	0.07258	0.10145	0.14297	0.20271	0.23001	0.24759			
1024	0.04834	0.05812	0.07618	0.10419	0.14254	0.19982	0.29455	0.38184	0.37453				
512	0.08604	0.10502	0.13791	0.18900	0.27067	0.38973	0.55004	0.75111					
256	0.15056	0.18377	0.24379	0.34770	0.52302	0.75468	1.10076						
128	0.24480	0.30492	0.41235	0.61214	0.89006	1.27729							
64	0.39344	0.50749	0.70083	1.06924	1.51003								
32	0.64965	0.86629	1.28054	1.81300									
16	1.04363	1.48373	1.88207										
8	1.58914	2.06546											
4	2.15315												

(c) $\mathcal{P}^{\mathcal{C}}$ for Scene: [**Sponza**] Algorithm: [**PSSMLT**] Metric: [**Contourlet**] True GT: [**PT @ 32768 spp**].

MLT	2	4	8	16	32	64	128	256	512	1024	2048	4096	8192
**GT (PT)**													
16384	0.00200	0.00330	0.00385	0.00547	0.00831	0.00949	0.01288	0.00986	0.02605	0.01602	0.02229	0.01785	0.03856
8192	0.00553	0.00854	0.01105	0.01712	0.02579	0.03831	0.04360	0.07445	0.10664	0.05866	0.09643	0.04593	
4096	0.01239	0.01834	0.02488	0.03715	0.05917	0.08719	0.08090	0.14364	0.14851	0.09574	0.15318		
2048	0.02495	0.03634	0.05111	0.07641	0.11860	0.17540	0.17305	0.22555	0.21410	0.24005			
1024	0.04937	0.07045	0.10158	0.15022	0.23572	0.35273	0.38561	0.43690	0.37872				
512	0.08903	0.12524	0.18566	0.28195	0.44429	0.63083	0.74789	0.79557					
256	0.15359	0.22084	0.33602	0.53627	0.83936	1.09013	1.26113						
128	0.25079	0.36737	0.59769	0.94000	1.39660	1.41619							
64	0.40623	0.63818	1.07476	1.55623	1.69767								
32	0.68592	1.14190	1.75132	2.06106									
16	1.18052	1.81916	2.11594										
8	1.76196	2.28446											
4	2.22195												

(d) $\mathcal{P}^{\mathcal{C}}$ for Scene: [**Sponza**] Algorithm: [**MLT**] Metric: [**Contourlet**] True GT: [**PT @ 32768 spp**].

M-MLT	2	4	8	16	32	64	128	256	512	1024	2048	4096	8192
**GT (PT)**													
16384	0.00221	0.00286	0.00432	0.00645	0.00945	0.01461	0.01005	0.00998	0.01884	0.03716	0.02383	0.01689	0.02467
8192	0.00629	0.00825	0.01295	0.01848	0.02943	0.04439	0.04562	0.05245	0.08070	0.06922	0.08465	0.08123	
4096	0.01344	0.01854	0.02613	0.03891	0.06701	0.09594	0.11035	0.11876	0.14378	0.14071	0.18738		
2048	0.02680	0.03777	0.05290	0.08117	0.13243	0.18584	0.19830	0.25444	0.30228	0.24789			
1024	0.05378	0.07146	0.10268	0.15848	0.26665	0.35904	0.33846	0.48465	0.40522				
512	0.09577	0.12787	0.18822	0.30179	0.50373	0.64010	0.68475	0.72347					
256	0.16796	0.22962	0.34388	0.57394	0.91894	0.98637	1.07911						
128	0.27626	0.39032	0.63477	0.98029	1.30681	1.33690							
64	0.45592	0.67917	1.11198	1.49535	1.68193								
32	0.77332	1.22958	1.85853	2.01369									
16	1.29587	1.90149	2.23019										
8	2.00390	2.27878											
4	2.33185												

(e) $\mathcal{P}^{\mathcal{C}}$ for Scene: [**Sponza**] Algorithm: [**M-MLT**] Metric: [**Contourlet**] True GT: [**PT @ 32768 spp**].

ERPT	2	4	8	16	32	64	128	256	512	1024	2048	4096	8192
**GT (PT)**													
16384	0.00987	0.01873	0.01358	0.02133	0.01855	0.01014	0.00775	0.01230	0.01157	0.01435	0.01346	0.02756	0.02432
8192	0.04479	0.06341	0.05751	0.07205	0.07138	0.05365	0.06678	0.04710	0.05283	0.07349	0.05315	0.06608	
4096	0.11253	0.12861	0.11145	0.13997	0.14768	0.12043	0.11496	0.11090	0.10868	0.11847	0.10598		
2048	0.19411	0.23093	0.20046	0.22131	0.22820	0.22310	0.21220	0.19252	0.19108	0.23892			
1024	0.42671	0.42873	0.35818	0.39441	0.41310	0.37420	0.38137	0.38059	0.40409				
512	0.69130	0.76298	0.65223	0.69870	0.78541	0.69664	0.76304	0.78288					
256	1.05705	1.10227	1.10576	1.00621	1.10591	1.10576	1.05211						
128	1.00343	1.13529	1.20497	1.12360	1.10591	1.17021							
64	1.25151	1.17005	1.24164	1.12360	1.07448								
32	1.25151	1.17005	1.20497	1.19138									
16	1.29029	1.20672	1.28041										
8	1.33139	1.33030											
4	1.25151												

(f) $\mathcal{P}^{\mathcal{C}}$ for Scene: [**Sponza**] Algorithm: [**ERPT**] Metric: [**Contourlet**] True GT: [**PT @ 32768 spp**].

M-ERPT	2	4	8	16	32	64	128	256	512	1024	2048	4096	8192
**GT (PT)**													
16384	0.01266	0.01837	0.02094	0.01277	0.00354	0.01005	0.01435	0.01124	0.01769	0.01463	0.00711	0.01092	0.02195
8192	0.04634	0.06638	0.07890	0.06869	0.04875	0.04562	0.05277	0.03880	0.05573	0.05382	0.05277	0.05382	
4096	0.11141	0.12588	0.12289	0.12530	0.09987	0.10165	0.14297	0.12494	0.11142	0.11614	0.10906		
2048	0.19952	0.20722	0.22223	0.22310	0.19677	0.16685	0.23892	0.20842	0.20374	0.23818			
1024	0.39997	0.43598	0.36747	0.46702	0.42381	0.33846	0.43336	0.35220	0.35254				
512	0.75416	0.70072	0.73825	0.71929	0.72442	0.71561	0.69172	0.61364					
256	1.13153	1.25274	1.04044	1.04244	1.05523	1.17353	0.99923						
128	1.23074	1.17729	1.16348	1.15984	1.14964	1.20829							
64	1.30618	1.17729	1.13046	1.34416	1.08520								
32	1.26741	1.17729	1.16348	1.19285									
16	1.26741	1.21396	1.23490										
8	1.30618	1.25274											
4	1.26741												

(g) $\mathcal{P}^{\mathcal{C}}$ for Scene: [**Sponza**] Algorithm: [**M-ERPT**] Metric: [**Contourlet**] True GT: [**PT @ 32768 spp**].

Table 10

4 MSE

4.1 Cornell Box

PT	2	4	8	16	32	64	128	256	512	1024	2048	4096	8192
GT (PT)													
16384	0.00012	0.00026	0.00018	0.00008	0.00093	0.00167	0.00355	0.00563	0.01535	0.02839	0.05330	0.08002	0.13762
8192	0.00031	0.00092	0.00054	0.00123	0.00184	0.00433	0.01138	0.02022	0.04137	0.07853	0.14181	0.22576	
4096	0.00063	0.00121	0.00138	0.00309	0.00660	0.01088	0.02821	0.05360	0.09839	0.17714	0.29542		
2048	0.00164	0.00187	0.00384	0.00707	0.01388	0.02877	0.05596	0.11083	0.19988	0.34685			
1024	0.00185	0.00345	0.00782	0.01545	0.02930	0.05674	0.11217	0.20577	0.37356				
512	0.00418	0.00838	0.01455	0.03059	0.06123	0.11134	0.21416	0.37398					
256	0.00838	0.01580	0.02833	0.06105	0.11253	0.22048	0.40112						
128	0.01621	0.03161	0.06163	0.11839	0.22075	0.40148							
64	0.03102	0.05953	0.11768	0.22525	0.40007								
32	0.06404	0.11610	0.22345	0.40178									
16	0.12294	0.22531	0.40003										
8	0.22077	0.40574											
4	0.39661												

(a) $\mathcal{P}^{\mathcal{C}}$ for Scene: **[Cornell Box]** Algorithm: **[PT]** Metric: **[MSE]** True GT: **[PT @ 32768 spp]**.

BDPT	2	4	8	16	32	64	128	256	512	1024	2048	4096	8192
GT (PT)													
16384	0.00021	0.00044	0.00035	0.00146	0.00250	0.00604	0.01123	0.01857	0.03963	0.06006	0.10249	0.14676	0.20907
8192	0.00082	0.00089	0.00228	0.00439	0.00790	0.01515	0.03214	0.05903	0.10703	0.17634	0.27113	0.38223	
4096	0.00067	0.00301	0.00481	0.00984	0.01821	0.03760	0.07164	0.13536	0.23000	0.37515	0.54666		
2048	0.00206	0.00420	0.01003	0.01965	0.04178	0.08150	0.14686	0.26415	0.44191	0.66779			
1024	0.00577	0.01141	0.02103	0.04097	0.08008	0.14905	0.27787	0.48703	0.75710				
512	0.01046	0.02234	0.04352	0.08472	0.15813	0.29235	0.50361	0.81414					
256	0.02121	0.04110	0.08476	0.16638	0.30385	0.52198	0.85770						
128	0.04228	0.08528	0.16436	0.30002	0.54550	0.87442							
64	0.08224	0.16066	0.30742	0.53591	0.88474								
32	0.15806	0.29905	0.53027	0.87775									
16	0.29062	0.52153	0.86325										
8	0.50545	0.84654											
4	0.81929												

(b) $\mathcal{P}^{\mathcal{C}}$ for Scene: **[Cornell Box]** Algorithm: **[BDPT]** Metric: **[MSE]** True GT: **[PT @ 32768 spp]**.

PSSMLT	2	4	8	16	32	64	128	256	512	1024	2048	4096	8192
GT (PT)													
16384	0.00016	0.00007	0.00032	0.00074	0.00226	0.00614	0.00998	0.01371	0.01806	0.02520	0.02775	0.03102	0.03068
8192	0.00060	0.00081	0.00091	0.00280	0.00660	0.01445	0.02217	0.03672	0.05004	0.06507	0.07511	0.08238	
4096	0.00107	0.00204	0.00480	0.00871	0.01752	0.03373	0.05686	0.08718	0.12014	0.15096	0.17301		
2048	0.00253	0.00468	0.00972	0.01826	0.03840	0.06819	0.11399	0.17703	0.23587	0.29476			
1024	0.00515	0.01108	0.02138	0.03953	0.07309	0.13399	0.22017	0.33279	0.43428				
512	0.01087	0.02140	0.04114	0.08006	0.14949	0.26047	0.41209	0.59543					
256	0.02301	0.04619	0.08810	0.16322	0.28883	0.47954	0.71833						
128	0.04525	0.08898	0.16590	0.30181	0.51430	0.80007							
64	0.08894	0.16842	0.30567	0.53115	0.84936								
32	0.16533	0.31154	0.53101	0.86310									
16	0.29947	0.53452	0.87151										
8	0.52155	0.86170											
4	0.85404												

(c) $\mathcal{P}^{\mathcal{C}}$ for Scene: **[Cornell Box]** Algorithm: **[PSSMLT]** Metric: **[MSE]** True GT: **[PT @ 32768 spp]**.

MLT	2	4	8	16	32	64	128	256	512	1024	2048	4096	8192
GT (PT)													
16384	0.00000	-0.00001	0.00015	-0.00004	0.00020	0.00075	0.00145	0.00426	0.00747	0.01141	0.01402	0.02277	0.02576
8192	0.00006	0.00025	0.00032	0.00065	0.00116	0.00309	0.00606	0.01193	0.02072	0.03328	0.04593	0.06046	
4096	0.00014	0.00045	0.00082	0.00170	0.00451	0.00921	0.01649	0.02940	0.05148	0.07625	0.10875		
2048	0.00062	0.00106	0.00167	0.00255	0.00726	0.01532	0.03028	0.05886	0.10029	0.15300			
1024	0.00117	0.00213	0.00433	0.00842	0.01751	0.03483	0.06496	0.11706	0.19571				
512	0.00297	0.00492	0.01010	0.01894	0.03701	0.07037	0.12961	0.22842					
256	0.00504	0.01080	0.02160	0.03943	0.07583	0.13958	0.24661						
128	0.00915	0.01731	0.03459	0.06706	0.13104	0.24501							
64	0.01652	0.03460	0.07347	0.13868	0.25597								
32	0.03678	0.06894	0.13624	0.25032									
16	0.06999	0.12981	0.25545										
8	0.12349	0.23553											
4	0.24328												

(d) $\mathcal{P}^{\mathcal{C}}$ for Scene: **[Cornell Box]** Algorithm: **[MLT]** Metric: **[MSE]** True GT: **[PT @ 32768 spp]**.

M-MLT	2	4	8	16	32	64	128	256	512	1024	2048	4096	8192
GT (PT)													
16384	0.00008	-0.00022	-0.00012	-0.00002	-0.00021	0.00069	0.00212	0.00488	0.00746	0.01234	0.01736	0.02176	0.02616
8192	0.00018	0.00024	0.00061	0.00105	0.00196	0.00360	0.00627	0.01225	0.02377	0.03538	0.04937	0.06028	
4096	0.00018	0.00035	0.00094	0.00211	0.00411	0.00870	0.01693	0.02974	0.05241	0.07919	0.11096		
2048	0.00088	0.00120	0.00244	0.00494	0.00886	0.01706	0.03165	0.06051	0.10295	0.15919			
1024	0.00130	0.00170	0.00444	0.00938	0.01875	0.03650	0.06735	0.12335	0.20225				
512	0.00209	0.00541	0.01037	0.02006	0.03602	0.07059	0.12861	0.22772					
256	0.00541	0.01085	0.01991	0.03779	0.07178	0.13756	0.24846						
128	0.00882	0.01808	0.03794	0.07390	0.14009	0.26279							
64	0.01734	0.03858	0.07472	0.13827	0.25603								
32	0.03911	0.07567	0.14513	0.26494									
16	0.07483	0.14636	0.26852										
8	0.14260	0.25865											
4	0.25770												

(e) $\mathcal{P}^{\mathcal{C}}$ for Scene: **[Cornell Box]** Algorithm: **[M-MLT]** Metric: **[MSE]** True GT: **[PT @ 32768 spp]**.

ERPT	2	4	8	16	32	64	128	256	512	1024	2048	4096	8192
GT (PT)													
16384	0.00265	0.00324	0.00203	0.00275	0.00202	0.00274	0.00276	0.00262	0.00359	0.00289	0.00170	0.00261	0.00325
8192	0.00800	0.00769	0.00806	0.00649	0.00735	0.00710	0.00682	0.00831	0.00769	0.00762	0.00696	0.00832	
4096	0.01661	0.01805	0.01597	0.01502	0.01619	0.01894	0.01758	0.01705	0.01679	0.01730	0.01653		
2048	0.03587	0.03642	0.03626	0.03503	0.03440	0.03746	0.03515	0.03385	0.03387	0.03663			
1024	0.07286	0.06999	0.07011	0.07041	0.06982	0.07522	0.07298	0.07067	0.07518				
512	0.14193	0.13887	0.13975	0.14001	0.14183	0.15017	0.14215	0.14426					
256	0.26811	0.27609	0.27112	0.26963	0.26964	0.27842	0.27050						
128	0.48064	0.48666	0.48520	0.48618	0.47604	0.49390							
64	0.80139	0.80901	0.80426	0.80263	0.78995								
32	1.23194	1.23834	1.24273	1.23918									
16	1.75032	1.76970	1.76886										
8	2.33824	2.35550											
4	2.95076												

(f) $\mathcal{P}^{\mathcal{C}}$ for Scene: **[Cornell Box]** Algorithm: **[ERPT]** Metric: **[MSE]** True GT: **[PT @ 32768 spp]**.

M-ERPT	2	4	8	16	32	64	128	256	512	1024	2048	4096	8192
GT (PT)													
16384	0.00247	0.00257	0.00265	0.00304	0.00265	0.00356	0.00291	0.00292	0.00316	0.00179	0.00124	0.00271	0.00254
8192	0.00770	0.00688	0.00665	0.00897	0.00677	0.00613	0.00614	0.00728	0.00582	0.00644	0.00638	0.00729	
4096	0.01546	0.01498	0.01553	0.01624	0.01623	0.01558	0.01548	0.01494	0.01567	0.01511	0.01602		
2048	0.03412	0.03277	0.03313	0.03467	0.03582	0.03417	0.03229	0.03141	0.03114	0.03353			
1024	0.06525	0.06640	0.07017	0.06763	0.06869	0.06611	0.06472	0.07149	0.06610				
512	0.12856	0.13168	0.12994	0.13558	0.13412	0.12894	0.13417	0.13028					
256	0.25427	0.25852	0.25728	0.25574	0.26021	0.25315	0.26168						
128	0.45492	0.45801	0.45614	0.46572	0.46522	0.45678							
64	0.77885	0.77498	0.76759	0.77032	0.78194								
32	1.19105	1.19851	1.18891	1.19575									
16	1.70519	1.71815	1.70129										
8	2.28710	2.29650											
4	2.90121												

(g) $\mathcal{P}^{\mathcal{C}}$ for Scene: **[Cornell Box]** Algorithm: **[M-ERPT]** Metric: **[MSE]** True GT: **[PT @ 32768 spp]**.

Table 11

4.2 Torus

PT	2	4	8	16	32	64	128	256	512	1024	2048	4096	8192	16384	32768	65536	131072
GT (BDPT)																	
262144	-0.00133	-0.00208	-0.00155	-0.00136	-0.00231	-0.00131	-0.00064	0.00080	-0.00028	0.00104	0.00286	0.00672	0.01491	0.02663	0.04862	0.10045	0.17094
131072	-0.00212	-0.00121	-0.00216	-0.00129	-0.00322	0.00045	0.00133	0.00233	0.00455	0.00517	0.01172	0.02898	0.04752	0.09447	0.17006	0.28736	
65536	-0.00163	0.00012	-0.00167	-0.00012	0.00079	0.00089	0.00487	0.00626	0.01069	0.01687	0.03190	0.05989	0.11307	0.19554	0.33428		
32768	0.00508	0.00883	0.01025	0.01314	0.01347	0.01322	0.01379	0.02175	0.02867	0.04344	0.06958	0.12656	0.23054	0.39684			
16384	0.01392	0.02293	0.02857	0.03215	0.02903	0.03715	0.03639	0.04984	0.05964	0.08847	0.13401	0.24801	0.41467				
8192	0.03025	0.04269	0.05010	0.05724	0.06229	0.06853	0.07113	0.09590	0.10646	0.16747	0.24862	0.43046					
4096	0.06559	0.08934	0.10257	0.12068	0.11175	0.12650	0.13795	0.16298	0.19662	0.28385	0.43011						
2048	0.10954	0.15264	0.17995	0.20294	0.20256	0.21344	0.23012	0.26961	0.33265	0.45880							
1024	0.14695	0.21569	0.25462	0.28547	0.28396	0.30611	0.32976	0.38793	0.48038								
512	0.18780	0.27245	0.32561	0.35937	0.36870	0.40762	0.42903	0.50196									
256	0.19588	0.27937	0.33723	0.36784	0.38130	0.43223	0.47318										
128	0.11497	0.18642	0.22516	0.28243	0.32849	0.38231											
64	0.05806	0.14513	0.15406	0.21857	0.27360												
32	-0.01540	0.04435	0.05091	0.14350													
16	-0.10917	-0.06859	-0.09244														
8	-0.11026	-0.06408															
4	-0.05572																

(a) $\mathcal{P}^{\mathcal{C}}$ for Scene: [**Torus**] Algorithm: [**PT**] Metric: [**MSE**] True GT: [**BDPT @ 524288 spp**].

BDPT	2	4	8	16	32	64	128	256	512	1024	2048	4096	8192	16384	32768	65536	131072
GT (BDPT)																	
262144	-0.00160	-0.00231	-0.00233	-0.00183	0.00035	-0.00140	-0.00083	-0.00087	0.00002	0.00067	0.00558	0.00396	0.01441	0.02459	0.04727	0.09986	0.17333
131072	-0.00153	-0.00279	-0.00232	-0.00145	-0.00151	0.00035	0.00104	0.00121	0.00341	0.00774	0.01550	0.02330	0.04713	0.08562	0.14775	0.28790	
65536	-0.00161	-0.00190	-0.00180	-0.00039	0.00120	0.00096	0.00439	0.00858	0.00983	0.01732	0.03450	0.05187	0.09912	0.18736	0.32224		
32768	0.00401	0.00886	0.01042	0.01446	0.01072	0.01558	0.01813	0.02506	0.02340	0.04473	0.07652	0.12633	0.21122	0.36581			
16384	0.01285	0.02221	0.02581	0.02970	0.02966	0.03313	0.03721	0.04772	0.06035	0.09217	0.14827	0.23927	0.38056				
8192	0.02527	0.03915	0.04868	0.05066	0.05081	0.06418	0.07718	0.08423	0.12043	0.16153	0.24303	0.40680					
4096	0.05425	0.08203	0.09684	0.11605	0.11301	0.12284	0.13362	0.16291	0.19803	0.27156	0.42558						
2048	0.10176	0.14731	0.16547	0.18635	0.19517	0.20725	0.22713	0.25778	0.32955	0.44411							
1024	0.14021	0.19977	0.24467	0.26594	0.28688	0.28740	0.33806	0.37846	0.48389								
512	0.17597	0.25324	0.30939	0.33827	0.35870	0.37978	0.43279	0.48225									
256	0.17141	0.26307	0.32109	0.34952	0.38040	0.42377	0.46704										
128	0.10090	0.16972	0.22615	0.25271	0.29533	0.35144											
64	0.05015	0.10223	0.15118	0.20743	0.27726												
32	-0.02305	-0.00729	0.06359	0.11440													
16	-0.11832	-0.11032	-0.05215														
8	-0.12158	-0.11027															
4	-0.06180																

(b) $\mathcal{P}^{\mathcal{C}}$ for Scene: [**Torus**] Algorithm: [**BDPT**] Metric: [**MSE**] True GT: [**BDPT @ 524288 spp**].

PSSMLT	2	4	8	16	32	64	128	256	512	1024	2048	4096	8192	16384
GT (BDPT)														
262144	-0.00211	-0.00235	-0.00188	-0.00243	-0.00157	-0.00196	0.00039	0.00106	0.00288	0.00594	0.00812	0.01068	0.02114	0.01529
131072	-0.00153	-0.00205	-0.00195	-0.00177	0.00060	0.00224	0.00571	0.01050	0.01145	0.02107	0.03337	0.04343	0.07758	0.06576
65536	-0.00236	-0.00019	0.00307	0.00247	0.00664	0.00934	0.01413	0.02577	0.03446	0.05618	0.08232	0.11020	0.17799	0.16191
32768	0.00116	0.00694	0.01001	0.01235	0.02027	0.02849	0.04010	0.06314	0.08837	0.13314	0.18153	0.23150	0.34876	0.32689
16384	0.01385	0.02580	0.03465	0.04538	0.05698	0.07209	0.09946	0.13513	0.17981	0.25578	0.34548	0.43516	0.61855	
8192	0.02283	0.03879	0.05498	0.06333	0.09275	0.13025	0.17337	0.23951	0.32422	0.44445	0.59862	0.73122		
4096	0.04558	0.08055	0.10202	0.12098	0.16503	0.21969	0.29440	0.40202	0.53021	0.70868	0.91162			
2048	0.09896	0.15508	0.19584	0.23084	0.29242	0.37928	0.48228	0.63834	0.80504	1.04515				
1024	0.13579	0.21759	0.27846	0.32346	0.40779	0.52334	0.67889	0.87288	1.09309					
512	0.17330	0.27625	0.34939	0.40411	0.52529	0.67166	0.83931	1.06891						
256	0.18247	0.29894	0.39499	0.46306	0.59707	0.77041	0.96623							
128	0.11111	0.23236	0.33681	0.41510	0.57024	0.75842								
64	0.08727	0.21370	0.32188	0.39039	0.57581									
32	0.01363	0.14536	0.26781	0.34369										
16	-0.05142	0.08085	0.20869											
8	-0.05534	0.08022												
4	-0.00087													

(c) $\mathcal{P}^{\mathcal{C}}$ for Scene: [**Torus**] Algorithm: [**PSSMLT**] Metric: [**MSE**] True GT: [**BDPT @ 524288 spp**].

MLT	2	4	8	16	32	64	128	256	512	1024	2048	4096	8192	16384
GT (BDPT)														
262144	-0.00110	-0.00150	-0.00069	-0.00062	0.00025	-0.00006	0.00131	0.00273	0.00113	0.00286	0.00276	0.00303	0.00363	-0.00040
131072	-0.00174	-0.00032	0.00277	0.00398	0.00625	0.00752	0.00877	0.01165	0.01144	0.01328	0.01528	0.01501	0.01736	0.01898
65536	-0.00336	-0.00128	0.00330	0.00625	0.01289	0.01435	0.01851	0.02654	0.02688	0.03161	0.03349	0.03512	0.03979	0.04481
32768	-0.00212	-0.00011	0.01107	0.01693	0.03035	0.03128	0.03972	0.06058	0.05814	0.06718	0.07121	0.07299	0.08493	0.10008
16384	-0.00252	0.00162	0.01882	0.02789	0.05841	0.05759	0.07272	0.10051	0.10333	0.12098	0.12580	0.13285	0.15080	
8192	-0.00069	0.01027	0.05050	0.06006	0.11174	0.12120	0.15515	0.20577	0.21098	0.24073	0.25184	0.26142		
4096	-0.00045	0.01973	0.08558	0.11464	0.19893	0.21037	0.26746	0.34640	0.35225	0.39826	0.41595			
2048	0.00126	0.03950	0.14812	0.18920	0.32785	0.34748	0.42141	0.53124	0.54266	0.60231				
1024	0.01000	0.07141	0.22030	0.28595	0.48214	0.51589	0.61889	0.75308	0.76373					
512	0.00735	0.08739	0.29132	0.36512	0.61730	0.65004	0.77069	0.94305						
256	-0.00470	0.10551	0.34619	0.42938	0.71134	0.76142	0.89913							
128	-0.03534	0.08837	0.33424	0.42970	0.72442	0.77306								
64	-0.04845	0.09415	0.33598	0.44321	0.76379									
32	-0.07653	0.06871	0.30959	0.42123										
16	-0.10742	0.04788	0.27698											
8	-0.09554	0.05608												
4	-0.10231													

(d) $\mathcal{P}^{\mathcal{C}}$ for Scene: [**Torus**] Algorithm: [**MLT**] Metric: [**MSE**] True GT: [**BDPT @ 524288 spp**].

M-MLT	2	4	8	16	32	64	128	256	512	1024	2048	4096	8192	16384
GT (BDPT)														
262144	-0.00054	-0.00044	-0.00058	-0.00060	-0.00151	0.00034	0.00393	0.00696	0.00588	0.00759	0.00966	0.01035	0.01084	0.00739
131072	-0.00097	-0.00058	0.00188	0.00055	0.00266	0.00902	0.01320	0.01422	0.01863	0.02382	0.02579	0.02807	0.03258	0.03627
65536	-0.00228	-0.00029	0.00418	0.00587	0.00815	0.01800	0.02788	0.03998	0.04232	0.05077	0.05913	0.05967	0.06978	0.08369
32768	-0.00208	0.00176	0.01473	0.01769	0.02702	0.04125	0.06177	0.07993	0.09006	0.10303	0.11833	0.12233	0.13860	0.17542
16384	-0.00194	0.00694	0.02691	0.02481	0.04871	0.07478	0.10654	0.14527	0.15895	0.18613	0.20967	0.21378	0.24599	
8192	-0.00058	0.01889	0.05204	0.05679	0.09972	0.15297	0.20112	0.27377	0.31020	0.34564	0.39389	0.40062		
4096	0.00439	0.03490	0.10224	0.09389	0.16993	0.26204	0.34698	0.44654	0.48779	0.55464	0.61549			
2048	0.00750	0.05896	0.16837	0.17245	0.29987	0.41174	0.53036	0.67465	0.73395	0.81976				
1024	0.01747	0.10057	0.25719	0.26315	0.42350	0.59990	0.76000	0.92826	0.99694					
512	0.02184	0.12831	0.35626	0.34252	0.52980	0.74086	0.93649	1.13408						
256	0.01917	0.14791	0.41270	0.40235	0.62366	0.86172	1.07869							
128	-0.00179	0.11954	0.42664	0.39981	0.63480	0.89302								
64	-0.01039	0.11341	0.46788	0.43071	0.68383									
32	-0.02847	0.09045	0.45835	0.41264										
16	-0.03735	0.07535	0.45534											
8	-0.03529	0.08151												
4	-0.03684													

(e) $\mathcal{P}^{\mathcal{C}}$ for Scene: [**Torus**] Algorithm: [**M-MLT**] Metric: [**MSE**] True GT: [**BDPT @ 524288 spp**].

ERPT	2	4	8	16	32	64	128	256	512	1024	2048	4096	8192	16384	32768	65536	131072
GT (BDPT)																	
262144	-0.00135	0.00023	0.00346	0.00316	0.01085	0.01003	0.01045	0.01054	0.01293	0.01345	0.01443	0.01814	0.01932	0.01694	0.01790	0.01583	0.01618
131072	0.00653	0.01277	0.01422	0.02270	0.02110	0.02900	0.03229	0.03508	0.04273	0.05046	0.05033	0.06245	0.06414	0.05987	0.05802	0.05734	
65536	0.01803	0.02947	0.03740	0.04511	0.05514	0.05816	0.07289	0.07681	0.08714	0.10069	0.11826	0.13300	0.14036	0.13490	0.13302		
32768	0.04517	0.06430	0.07635	0.10291	0.11764	0.12881	0.14691	0.16474	0.17867	0.21445	0.24408	0.27632	0.28155	0.26077			
16384	0.09685	0.13500	0.16365	0.19103	0.20564	0.23989	0.27646	0.31789	0.33980	0.39575	0.44811	0.49017	0.50845				
8192	0.18072	0.25401	0.29656	0.34592	0.36977	0.42743	0.48832	0.55570	0.58292	0.66855	0.73996	0.79442					
4096	0.31743	0.41424	0.49313	0.56803	0.61385	0.68766	0.75123	0.85275	0.89756	1.00163	1.09014						
2048	0.49363	0.65442	0.76657	0.85357	0.90000	0.98536	1.08405	1.21543	1.25614	1.38989							
1024	0.69515	0.90997	1.00213	1.13701	1.19789	1.29182	1.41260	1.55561	1.60904								
512	0.85472	1.10504	1.22666	1.37983	1.43005	1.54099	1.65469	1.81696									
256	0.96701	1.24984	1.38345	1.51291	1.59451	1.69991	1.83741										
128	0.94050	1.24567	1.38415	1.55403	1.63878	1.74407											
64	0.95408	1.28020	1.43398	1.60126	1.68031												
32	0.92815	1.28184	1.42016	1.57939													
16	0.87239	1.26277	1.40764														
8	0.90697	1.29915															
4	1.03688																

(f) $\mathcal{P}^{\mathcal{C}}$ for Scene: [**Torus**] Algorithm: [**ERPT**] Metric: [**MSE**] True GT: [**BDPT @ 524288 spp**].

M-ERPT	2	4	8	16	32	64	128	256	512	1024	2048	4096	8192	16384	32768	65536	131072
GT (BDPT)																	
262144	-0.00061	0.00260	0.00085	0.00581	0.00457	0.00654	0.01552	0.01409	0.01513	0.01570	0.02043	0.01826	0.02097	0.02071	0.01799	0.01821	0.01877
131072	0.00532	0.01029	0.00854	0.01068	0.01908	0.02222	0.03514	0.04452	0.05054	0.04942	0.05596	0.05524	0.05517	0.05684	0.05884	0.05756	
65536	0.01019	0.01923	0.02881	0.03710	0.05430	0.06177	0.08246	0.09860	0.11097	0.11287	0.12523	0.12224	0.12196	0.12618	0.12962		
32768	0.02921	0.04715	0.05754	0.07734	0.10821	0.14802	0.17634	0.20037	0.21620	0.23214	0.25885	0.25684	0.25404	0.25357			
16384	0.05696	0.07830	0.13051	0.14884	0.20509	0.27407	0.31701	0.35748	0.40022	0.42276	0.46549	0.46278	0.46276				
8192	0.10693	0.14942	0.23609	0.27548	0.37600	0.47951	0.55765	0.61219	0.68498	0.70900	0.76563	0.75886					
4096	0.19305	0.26576	0.40457	0.46974	0.60997	0.76370	0.84358	0.92790	1.00859	1.04659	1.12138						
2048	0.32801	0.41491	0.62656	0.70776	0.90653	1.10136	1.20754	1.29260	1.39599	1.44154							
1024	0.49127	0.60526	0.86777	0.97015	1.21735	1.43452	1.54547	1.64711	1.75984								
512	0.61641	0.76186	1.04671	1.18357	1.44294	1.67984	1.80128	1.92212									
256	0.72443	0.86759	1.18233	1.32026	1.59232	1.84180	1.97992										
128	0.72900	0.85893	1.18976	1.35710	1.62776	1.87579											
64	0.76594	0.87082	1.19789	1.40203	1.64554												
32	0.74438	0.85855	1.17817	1.37713													
16	0.70793	0.83415	1.14937														
8	0.72262	0.86635															
4	0.83500																

(g) $\mathcal{P}^{\mathcal{C}}$ for Scene: [**Torus**] Algorithm: [**M-ERPT**] Metric: [**MSE**] True GT: [**BDPT @ 524288 spp**].

Table 12

4.3 Veach Bidir

PT	2	4	8	16	32	64	128	256	512	1024	2048	4096	8192	16384	32768	65536	131072
GT (BDPT)																	
262144	-0.00011	-0.00014	-0.00014	-0.00015	-0.00014	0.00009	-0.00035	-0.00024	-0.00050	-0.00051	-0.00015	-0.00067	0.00041	0.00077	0.00076	0.00153	0.00225
131072	-0.00021	-0.00024	-0.00029	-0.00023	-0.00021	-0.00011	-0.00010	0.00000	0.00002	0.00000	-0.00040	0.00054	0.00038	0.00044	0.00193	0.00332	
65536	-0.00020	-0.00021	-0.00026	-0.00041	-0.00036	-0.00036	-0.00055	-0.00037	0.00022	-0.00002	-0.00063	-0.00051	0.00096	0.00405	0.00501		
32768	-0.00022	-0.00009	-0.00026	-0.00041	-0.00000	-0.00013	-0.00024	-0.00028	0.00058	0.00109	0.00122	0.00235	0.00387	0.00678			
16384	-0.00043	-0.00064	-0.00086	-0.00088	-0.00100	-0.00083	-0.00030	-0.00012	-0.00039	0.00041	0.00111	0.00269	0.00576				
8192	-0.00037	-0.00035	-0.00064	-0.00071	-0.00046	-0.00125	-0.00018	-0.00032	-0.00001	0.00224	0.00399	0.00798					
4096	0.00005	-0.00014	0.00012	0.00017	0.00099	0.00127	0.00278	0.00449	0.00450	0.00557	0.00630						
2048	-0.00051	-0.00034	-0.00058	-0.00038	-0.00077	-0.00072	0.00259	0.00287	0.00317	0.01045							
1024	-0.00094	-0.00114	-0.00198	-0.00286	-0.00261	-0.00313	0.00171	0.00071	0.00093								
512	-0.00102	-0.00172	-0.00255	-0.00200	-0.00164	-0.00511	0.00380	0.00567									
256	-0.00085	-0.00098	-0.00191	-0.00139	0.00493	-0.00209	0.01423										
128	-0.00067	-0.00051	-0.00271	0.00019	0.00795	0.00241											
64	-0.00024	0.00023	0.00037	0.00299	0.01585												
32	0.00162	0.00467	0.00927	0.01783													
16	0.00760	0.01519	0.02604														
8	0.01970	0.03645															
4	0.04075																

(a) $\mathcal{P}^{\mathcal{C}}$ for Scene: [**Veach Bidir**] Algorithm: [**PT**] Metric: [**MSE**] True GT: [**BDPT @ 524288 spp**].

BDPT	2	4	8	16	32	64	128	256	512	1024	2048	4096	8192	16384	32768	65536	131072
GT (BDPT)																	
262144	-0.00021	-0.00027	-0.00038	-0.00094	-0.00072	-0.00092	-0.00250	0.00255	-0.00085	-0.01967	0.00417	0.06492	0.00800	-0.00953	0.03021	0.04258	0.14344
131072	-0.00029	-0.00043	-0.00027	0.00049	0.00112	0.00063	0.00104	-0.00008	0.01060	0.01844	0.04361	0.02805	0.05505	0.04984	0.06518	0.11067	
65536	-0.00075	-0.00076	-0.00101	-0.00171	-0.00001	0.00026	0.00173	0.00443	-0.00519	0.02208	0.02897	0.07995	0.06474	0.17347	0.19254		
32768	-0.00010	0.00025	0.00047	0.00006	0.00326	0.00907	0.01074	0.01703	0.00466	0.04235	0.05516	0.23348	0.29287	0.32068			
16384	-0.00138	-0.00255	-0.00350	-0.00043	-0.01035	-0.01489	-0.02369	-0.00434	-0.02019	0.03043	0.07381	0.37069	0.31088				
8192	-0.00113	-0.00164	-0.00425	-0.00290	-0.00270	-0.00717	-0.01112	0.03119	0.03423	0.12088	0.23892	0.51296					
4096	0.00109	0.00240	0.00698	0.01162	0.02268	0.03335	0.05799	0.09204	0.14874	0.26571	0.41930						
2048	-0.00079	-0.00158	0.00115	0.01298	0.00019	0.01354	0.02278	0.13249	0.03714	0.31843							
1024	-0.00462	-0.00686	-0.01133	-0.00513	-0.01781	-0.05069	-0.05730	0.10584	0.08821								
512	-0.00636	-0.01076	-0.01573	-0.01071	-0.01200	-0.04871	0.00169	0.24755									
256	-0.00333	-0.00566	-0.00683	0.01477	0.01381	0.00313	0.13129										
128	-0.00093	0.00322	0.01025	0.08396	0.07211	0.03017											
64	0.01108	0.02258	0.05005	0.15418	0.20765												
32	0.03988	0.07732	0.16147	0.33606													
16	0.10156	0.18343	0.33124														
8	0.20024	0.35875															
4	0.37029																

(b) $\mathcal{P}^{\mathcal{C}}$ for Scene: [**Veach Bidir**] Algorithm: [**BDPT**] Metric: [**MSE**] True GT: [**BDPT @ 524288 spp**].

PSSMLT	2	4	8	16	32	64	128	256	512	1024	2048	4096	8192	16384
GT (BDPT)														
262144	-0.00026	-0.00035	-0.00037	-0.00047	-0.00113	-0.00094	-0.00082	0.00040	-0.00106	-0.00175	-0.00904	-0.00760	-0.00152	-0.00155
131072	-0.00052	-0.00038	-0.00031	0.00013	0.00009	0.00039	0.00093	0.00110	0.00571	0.00630	0.00459	0.00503	0.00867	0.00614
65536	-0.00066	-0.00073	-0.00043	-0.00076	-0.00244	-0.00275	-0.00312	0.00371	-0.00432	0.00319	0.00698	0.00974	0.01410	0.01850
32768	-0.00053	-0.00044	0.00003	0.00056	0.00220	0.00176	0.00290	0.00887	0.01088	0.01972	0.01850	0.03722	0.04407	0.04641
16384	-0.00110	-0.00210	-0.00335	-0.00518	-0.00645	-0.01137	-0.01358	-0.01250	0.02741	0.04212	0.06201	0.06109	0.07044	
8192	-0.00100	-0.00141	-0.00292	-0.00471	-0.00802	-0.00950	-0.01301	-0.00431	0.03259	0.04816	0.08138	0.10079		
4096	0.00091	0.00296	0.00605	0.01117	0.01477	0.02396	0.03784	0.08127	0.13455	0.14152	0.20186			
2048	-0.00073	-0.00108	-0.00083	-0.00034	0.00435	0.00255	0.00497	0.04905	0.19551	0.22554				
1024	-0.00384	-0.00622	-0.01267	-0.02287	-0.03313	-0.05016	-0.06855	-0.01223	0.15175					
512	-0.00406	-0.00774	-0.01499	-0.02781	-0.03523	-0.06410	-0.08452	0.10230						
256	-0.00323	-0.00440	-0.00635	-0.00954	-0.00426	-0.00298	-0.00918							
128	-0.00049	0.00240	0.00799	0.01649	0.03894	0.06324								
64	0.00941	0.01989	0.03791	0.08191	0.14430									
32	0.03930	0.07816	0.13363	0.25157										
16	0.08849	0.17277	0.30206											
8	0.17504	0.32348												
4	0.32699													

(c) $\mathcal{P}^{\mathcal{C}}$ for Scene: [**Veach Bidir**] Algorithm: [**PSSMLT**] Metric: [**MSE**] True GT: [**BDPT @ 524288 spp**].

MLT	2	4	8	16	32	64	128	256	512	1024	2048	4096	8192	16384
GT (BDPT)														
262144	-0.00017	-0.00016	-0.00017	-0.00018	-0.00024	-0.00062	-0.00091	-0.00089	-0.00098	-0.00099	-0.00341	-0.00390	-0.00336	-0.00307
131072	-0.00046	-0.00054	-0.00065	-0.00067	-0.00074	-0.00119	-0.00097	-0.00074	-0.00006	0.00092	0.00135	0.00108	0.00194	0.00237
65536	-0.00055	-0.00068	-0.00083	-0.00128	-0.00159	-0.00241	-0.00283	-0.00349	-0.00438	-0.00449	-0.00616	-0.00581	-0.00541	-0.00420
32768	-0.00036	-0.00036	-0.00034	-0.00080	-0.00097	-0.00150	-0.00118	0.00088	0.00129	0.00277	0.00235	0.00637	0.00609	0.00730
16384	-0.00060	-0.00081	-0.00122	-0.00211	-0.00301	-0.00461	-0.00600	-0.00777	-0.01202	-0.01676	-0.01311	-0.01840	-0.02040	
8192	-0.00060	-0.00078	-0.00130	-0.00239	-0.00222	-0.00315	-0.00655	-0.01040	-0.01267	-0.01553	-0.01922	-0.01868		
4096	-0.00046	-0.00049	0.00015	0.00110	0.00172	0.00493	0.01203	0.01746	0.02947	0.04329	0.05631			
2048	-0.00099	-0.00146	-0.00214	-0.00163	-0.00161	-0.00091	0.00015	0.00323	0.00552	0.00135				
1024	-0.00122	-0.00270	-0.00452	-0.00739	-0.00715	-0.01357	-0.02474	-0.04675	-0.06444					
512	-0.00234	-0.00394	-0.00706	-0.00897	-0.00807	-0.01355	-0.02421	-0.04168						
256	-0.00144	-0.00252	-0.00522	-0.00790	-0.00532	-0.00507	-0.00985							
128	-0.00158	-0.00254	-0.00225	0.00065	0.00967	0.01791								
64	-0.00086	0.00001	0.00239	0.01213	0.03623									
32	0.00284	0.00713	0.01676	0.04563										
16	0.01115	0.02581	0.05454											
8	0.02458	0.05337												
4	0.04773													

(d) $\mathcal{P}^{\mathcal{C}}$ for Scene: [**Veach Bidir**] Algorithm: [**MLT**] Metric: [**MSE**] True GT: [**BDPT @ 524288 spp**].

M-MLT	2	4	8	16	32	64	128	256	512	1024	2048	4096	8192	16384
GT (BDPT)														
262144	-0.00024	-0.00028	-0.00040	-0.00038	-0.00073	-0.00062	-0.00078	-0.00109	-0.00161	-0.00097	-0.00106	-0.00158	-0.00157	-0.00299
131072	-0.00049	-0.00055	-0.00066	-0.00076	-0.00105	-0.00073	-0.00085	-0.00030	-0.00040	0.00090	0.00290	0.00137	0.00301	0.00268
65536	-0.00050	-0.00055	-0.00083	-0.00072	-0.00179	-0.00133	-0.00235	-0.00302	-0.00343	-0.00205	-0.00109	-0.00346	-0.00241	-0.00335
32768	-0.00048	-0.00042	-0.00057	-0.00013	-0.00063	-0.00006	0.00002	0.00098	0.00282	0.00846	0.00546	0.00849	0.00868	0.00852
16384	-0.00065	-0.00078	-0.00097	-0.00235	-0.00267	-0.00432	-0.00630	-0.00902	-0.01195	-0.01784	-0.01679	-0.02329	-0.02522	
8192	-0.00082	-0.00153	-0.00172	-0.00257	-0.00374	-0.00524	-0.00586	-0.00736	-0.01317	-0.01065	-0.00953	-0.01545		
4096	-0.00029	0.00034	0.00051	0.00192	0.00381	0.00655	0.01263	0.01887	0.03192	0.04540	0.05679			
2048	-0.00046	-0.00018	-0.00032	-0.00104	-0.00229	0.00031	0.00325	-0.00015	0.00307	0.01052				
1024	-0.00136	-0.00288	-0.00258	-0.00642	-0.01076	-0.02012	-0.02742	-0.04678	-0.06408					
512	-0.00166	-0.00381	-0.00371	-0.00900	-0.01410	-0.02286	-0.03008	-0.05059						
256	-0.00154	-0.00256	-0.00209	-0.00675	-0.00886	-0.01904	-0.01733							
128	-0.00126	-0.00311	-0.00138	-0.00399	-0.00096	0.00167								
64	-0.00096	-0.00240	0.00326	0.00413	0.01833									
32	0.00251	0.00648	0.01798	0.03997										
16	0.00982	0.02261	0.05397											
8	0.02485	0.05345												
4	0.04891													

(e) $\mathcal{P}^{\mathcal{C}}$ for Scene: [**Veach Bidir**] Algorithm: [**M-MLT**] Metric: [**MSE**] True GT: [**BDPT @ 524288 spp**].

ERPT	2	4	8	16	32	64	128	256	512	1024	2048	4096	8192	16384	32768	65536	131072
GT (BDPT)																	
262144	-0.00056	-0.00043	-0.00109	-0.00069	-0.00127	-0.00089	-0.00182	-0.00041	-0.00105	0.00054	0.00017	0.00069	-0.00084	0.00093	0.00013	-0.00080	-0.00102
131072	0.00009	-0.00044	0.00034	0.00027	-0.00056	-0.00106	0.00073	-0.00063	0.00044	-0.00031	-0.00057	0.00010	-0.00023	0.00034	-0.00028	-0.00099	
65536	-0.00062	-0.00180	-0.00076	-0.00120	-0.00069	-0.00123	-0.00012	0.00009	0.00330	0.00126	0.00331	0.00176	0.00213	0.00198	0.00120		
32768	0.00177	0.00168	0.00154	0.00008	0.00102	0.00338	0.00202	0.00137	0.00358	0.00467	0.00447	0.00433	0.00380	0.00365			
16384	-0.00474	-0.00644	-0.00770	-0.00638	-0.00797	0.00416	-0.00273	0.00908	0.00944	0.00576	0.00660	0.00968	0.00707				
8192	-0.00688	-0.00612	-0.00568	-0.00441	-0.00211	0.01292	0.00195	0.00173	0.00877	0.01089	0.01221	0.01285					
4096	0.01847	0.01370	0.01822	0.01445	0.01729	0.01184	0.01556	0.01319	0.02858	0.01844	0.01990						
2048	0.00392	0.00433	-0.00043	0.00115	-0.00084	0.02159	0.01212	0.03049	0.03642	0.03450							
1024	-0.02769	-0.02931	-0.03118	-0.02962	-0.01253	0.04319	-0.00563	0.02877	0.04872								
512	-0.03085	-0.03880	-0.03629	-0.03346	0.00290	0.09242	0.02274	0.07335									
256	-0.00857	-0.01220	-0.00717	-0.00601	0.04343	0.13819	0.09185										
128	0.01975	0.02198	0.02477	0.01941	0.08512	0.25267											
64	0.11765	0.10981	0.12545	0.11975	0.20143												
32	0.33778	0.35903	0.36604	0.36870													
16	0.68246	0.67910	0.70353														
8	1.09329	1.10935															
4	1.60379																

(f) $\mathcal{P}^{\mathcal{C}}$ for Scene: [**Veach Bidir**] Algorithm: [**ERPT**] Metric: [**MSE**] True GT: [**BDPT @ 524288 spp**].

M-ERPT	2	4	8	16	32	64	128	256	512	1024	2048	4096	8192	16384	32768	65536	131072
GT (BDPT)																	
262144	-0.00026	-0.00121	-0.00091	-0.00119	-0.00046	-0.00040	0.00116	-0.00021	0.00120	0.00022	-0.00018	-0.00066	-0.00008	-0.00009	-0.00001	0.00003	-0.00159
131072	0.00020	-0.00005	-0.00027	0.00045	-0.00003	0.00032	0.00050	0.00020	0.00087	0.00010	-0.00027	-0.00027	-0.00076	-0.00055	-0.00058	-0.00041	
65536	-0.00098	-0.00194	-0.00020	-0.00091	0.00040	0.00030	0.00217	0.00111	0.00190	0.00065	0.00179	0.00132	0.00130	0.00133	0.00018		
32768	0.00067	0.00099	0.00146	0.00103	0.00247	0.00269	0.00473	0.00198	0.00257	0.00279	0.00338	0.00384	0.00389	0.00260			
16384	-0.00728	-0.00572	0.00302	0.00316	0.00055	0.00445	0.00524	0.00615	0.00444	0.00833	0.00745	0.00733	0.00773				
8192	-0.00471	-0.00631	0.00700	0.00559	0.00277	0.00698	0.01667	0.00704	0.01144	0.01120	0.01318	0.01168					
4096	0.01595	0.01301	0.02264	0.02094	0.01688	0.02301	0.01974	0.02275	0.02054	0.02036	0.02021						
2048	-0.00526	0.00062	0.02873	0.02943	0.02433	0.03305	0.02386	0.03145	0.02160	0.03777							
1024	-0.02863	-0.03621	0.04537	0.02788	0.01710	0.04367	0.05802	0.04725	0.05008								
512	-0.03569	-0.03166	0.10581	0.07379	0.06203	0.10445	0.10487	0.10934									
256	-0.01611	-0.01918	0.14064	0.13242	0.10826	0.15006	0.14838										
128	0.01062	0.00257	0.26868	0.23932	0.21955	0.26779											
64	0.09816	0.10219	0.39759	0.37068	0.35684												
32	0.30314	0.32039	0.57338	0.54527													
16	0.63685	0.63612	0.85790														
8	1.02889	1.04320															
4	1.53069																

(g) $\mathcal{P}^{\mathcal{C}}$ for Scene: [**Veach Bidir**] Algorithm: [**M-ERPT**] Metric: [**MSE**] True GT: [**BDPT @ 524288 spp**].

Table 13

4.4 Veach Door

PT	2	4	8	16	32	64	128	256	512	1024	2048	4096	8192	16384	32768	65536	131072
GT (BDPT)																	
262144	-0.00007	-0.00002	0.00000	-0.00002	0.00011	0.00000	0.00015	0.00027	0.00027	0.00065	0.00074	0.00118	0.00246	0.00465	0.00802	0.01922	0.02875
131072	-0.00014	0.00001	0.00001	0.00008	0.00025	0.00009	0.00039	0.00074	0.00089	0.00122	0.00202	0.00398	0.00624	0.01176	0.02476	0.04269	
65536	-0.00006	-0.00003	-0.00002	-0.00007	0.00024	0.00038	0.00052	0.00122	0.00174	0.00272	0.00507	0.00794	0.01504	0.02932	0.05291		
32768	0.00013	0.00020	0.00004	0.00022	0.00050	0.00058	0.00157	0.00290	0.00317	0.00538	0.00952	0.01888	0.03234	0.06238			
16384	0.00031	0.00027	0.00022	0.00040	0.00056	0.00089	0.00218	0.00361	0.00696	0.01086	0.01827	0.03513	0.06594				
8192	0.00084	0.00052	0.00039	0.00034	0.00081	0.00221	0.00420	0.00631	0.01211	0.02236	0.03882	0.07164					
4096	0.00151	0.00112	0.00088	0.00182	0.00313	0.00544	0.00769	0.01508	0.02467	0.04664	0.07806						
2048	0.00301	0.00204	0.00122	0.00382	0.00558	0.01027	0.01614	0.02944	0.05056	0.08520							
1024	0.00630	0.00420	0.00420	0.00662	0.01197	0.02151	0.03606	0.06312	0.09899								
512	0.01124	0.00891	0.00858	0.01302	0.02474	0.04217	0.06931	0.11770									
256	0.02009	0.01516	0.01716	0.02533	0.04978	0.08694	0.13655										
128	0.03275	0.02848	0.03376	0.05412	0.09759	0.16434											
64	0.05400	0.05268	0.06025	0.10582	0.18791												
32	0.08755	0.09097	0.12255	0.20270													
16	0.14351	0.15745	0.22686														
8	0.23689	0.27939															
4	0.36480																

(a) $\mathcal{P}^{\mathcal{C}}$ for Scene: [**Veach Door**] Algorithm: [**PT**] Metric: [**MSE**] True GT: [**BDPT @ 524288 spp**].

BDPT	2	4	8	16	32	64	128	256	512	1024	2048	4096	8192	16384	32768	65536	131072
GT (BDPT)																	
262144	-0.00004	-0.00003	0.00002	-0.00002	0.00010	0.00020	0.00018	0.00006	0.00150	0.00188	0.00496	0.00853	0.01511	0.03031	0.05818	0.09621	0.14864
131072	-0.00003	0.00001	0.00002	0.00019	0.00024	0.00033	0.00112	0.00192	0.00414	0.00648	0.01245	0.02067	0.04348	0.08319	0.14124	0.23949	
65536	-0.00006	-0.00002	0.00009	0.00032	0.00073	0.00085	0.00198	0.00346	0.00663	0.01324	0.02723	0.05307	0.09920	0.18218	0.31368		
32768	0.00004	0.00010	0.00010	0.00077	0.00113	0.00172	0.00359	0.00780	0.01737	0.03054	0.06032	0.10988	0.21229	0.36902			
16384	0.00021	0.00020	0.00033	0.00056	0.00143	0.00352	0.00771	0.01354	0.03007	0.05668	0.10869	0.20922	0.37493				
8192	0.00053	0.00024	0.00028	0.00133	0.00280	0.00716	0.01435	0.02938	0.05890	0.11359	0.21520	0.39475					
4096	0.00067	0.00118	0.00163	0.00337	0.00647	0.01509	0.02988	0.05782	0.11757	0.21721	0.40316						
2048	0.00180	0.00212	0.00309	0.00614	0.01266	0.02876	0.05739	0.11348	0.21185	0.39332							
1024	0.00395	0.00437	0.00646	0.01303	0.02783	0.05818	0.11527	0.21749	0.39490								
512	0.00838	0.00795	0.01448	0.02658	0.05419	0.11363	0.21768	0.39255									
256	0.01619	0.01715	0.02628	0.05277	0.10436	0.20997	0.37846										
128	0.02948	0.03340	0.05575	0.10921	0.20695	0.38414											
64	0.05018	0.06460	0.11268	0.20854	0.37890												
32	0.09101	0.12177	0.20892	0.37953													
16	0.16671	0.23840	0.38285														
8	0.28829	0.41236															
4	0.46995																

(b) $\mathcal{P}^{\mathcal{C}}$ for Scene: [**Veach Door**] Algorithm: [**BDPT**] Metric: [**MSE**] True GT: [**BDPT @ 524288 spp**].

PSSMLT	2	4	8	16	32	64	128	256	512	1024	2048	4096	8192	16384
GT (BDPT)														
262144	0.00000	0.00000	-0.00003	-0.00001	0.00007	0.00035	0.00101	0.00160	0.00275	0.00548	0.00899	0.01584	0.02664	0.04421
131072	0.00003	0.00008	0.00032	0.00029	0.00084	0.00123	0.00219	0.00378	0.00602	0.01131	0.02338	0.04240	0.07166	0.10869
65536	0.00010	0.00019	0.00038	0.00033	0.00105	0.00183	0.00350	0.00690	0.01349	0.02644	0.05129	0.09068	0.15068	0.23272
32768	0.00018	0.00038	0.00055	0.00121	0.00255	0.00513	0.00917	0.01727	0.03284	0.05991	0.10897	0.18909	0.30490	0.44102
16384	0.00012	0.00027	0.00066	0.00183	0.00392	0.00773	0.01597	0.02913	0.05687	0.10742	0.19770	0.33928	0.52897	
8192	0.00059	0.00100	0.00182	0.00374	0.00796	0.01615	0.03161	0.06069	0.11452	0.20961	0.36941	0.59911		
4096	0.00120	0.00164	0.00332	0.00778	0.01701	0.03208	0.06229	0.11953	0.21811	0.39514	0.63934			
2048	0.00186	0.00339	0.00789	0.01704	0.03180	0.06337	0.12140	0.21772	0.38889	0.65504				
1024	0.00423	0.00859	0.01704	0.03423	0.06683	0.12723	0.22935	0.40698	0.67315					
512	0.00892	0.01740	0.03531	0.06926	0.12879	0.23897	0.41384	0.68811						
256	0.01983	0.03681	0.06897	0.13363	0.24149	0.42272	0.68933							
128	0.03983	0.07420	0.13601	0.24697	0.42829	0.71306								
64	0.08224	0.14395	0.25762	0.44427	0.72174									
32	0.14957	0.27235	0.46622	0.74378										
16	0.28233	0.47875	0.77401											
8	0.48869	0.78565												
4	0.78342													

(c) $\mathcal{P}^{\mathcal{C}}$ for Scene: [**Veach Door**] Algorithm: [**PSSMLT**] Metric: [**MSE**] True GT: [**BDPT @ 524288 spp**].

MLT	2	4	8	16	32	64	128	256	512	1024	2048	4096	8192	16384
GT (BDPT)														
262144	-0.00007	-0.00006	0.00003	0.00017	0.00019	0.00057	0.00074	0.00236	0.00251	0.00501	0.01003	0.01932	0.03224	0.04350
131072	-0.00006	0.00014	0.00037	0.00038	0.00080	0.00140	0.00245	0.00448	0.00743	0.01362	0.02957	0.04938	0.07258	0.11389
65536	0.00005	0.00010	0.00059	0.00063	0.00151	0.00355	0.00519	0.00995	0.01786	0.03184	0.05844	0.10737	0.16081	0.24180
32768	-0.00007	0.00044	0.00087	0.00088	0.00288	0.00526	0.01022	0.01904	0.03550	0.06472	0.12172	0.21687	0.32538	0.45557
16384	0.00006	0.00044	0.00109	0.00222	0.00468	0.00912	0.01925	0.03893	0.07139	0.13029	0.24112	0.39172	0.56177	
8192	0.00007	0.00060	0.00171	0.00447	0.00979	0.01837	0.04100	0.07650	0.13952	0.25768	0.43275	0.68041		
4096	0.00062	0.00235	0.00487	0.00916	0.02005	0.03847	0.07649	0.15383	0.26519	0.45298	0.73895			
2048	0.00160	0.00348	0.00811	0.01921	0.03701	0.07520	0.14226	0.26735	0.46704	0.75009				
1024	0.00410	0.00898	0.02010	0.04037	0.07994	0.15178	0.27186	0.48560	0.77617					
512	0.00844	0.01844	0.03891	0.08195	0.15135	0.27488	0.48881	0.80523						
256	0.01750	0.03703	0.07954	0.15408	0.27556	0.47955	0.79006							
128	0.03373	0.07650	0.15693	0.28921	0.49397	0.80174								
64	0.07781	0.14888	0.29810	0.51211	0.82007									
32	0.14782	0.28568	0.52613	0.84563										
16	0.27734	0.50805	0.86886											
8	0.49539	0.83503												
4	0.79586													

(d) $\mathcal{P}^{\mathcal{C}}$ for Scene: [**Veach Door**] Algorithm: [**MLT**] Metric: [**MSE**] True GT: [**BDPT @ 524288 spp**].

M-MLT	2	4	8	16	32	64	128	256	512	1024	2048	4096	8192	16384
GT (BDPT)														
262144	-0.00000	0.00001	0.00016	-0.00000	-0.00038	0.00046	0.00079	0.00189	0.00414	0.00461	0.01109	0.01692	0.02706	0.04076
131072	0.00008	0.00007	0.00026	0.00042	0.00066	0.00184	0.00311	0.00413	0.00881	0.01346	0.02875	0.04276	0.07487	0.09625
65536	0.00013	0.00010	0.00051	0.00055	0.00104	0.00353	0.00509	0.01097	0.01865	0.03257	0.06219	0.09284	0.16859	0.21608
32768	0.00016	0.00041	0.00110	0.00111	0.00283	0.00530	0.01166	0.01863	0.03700	0.06569	0.12936	0.19117	0.32250	0.40987
16384	0.00024	0.00030	0.00117	0.00241	0.00583	0.00909	0.01826	0.03740	0.07089	0.12643	0.23482	0.34415	0.55681	
8192	0.00021	0.00088	0.00201	0.00495	0.01019	0.02025	0.03582	0.07351	0.13906	0.24190	0.43356	0.61087		
4096	0.00155	0.00236	0.00502	0.01005	0.02178	0.03939	0.08498	0.14650	0.26264	0.44172	0.73649			
2048	0.00218	0.00373	0.01003	0.01803	0.03978	0.07713	0.15097	0.27059	0.46203	0.73509				
1024	0.00471	0.01004	0.02245	0.03987	0.08521	0.15489	0.27927	0.47950	0.78148					
512	0.00954	0.01907	0.04213	0.08122	0.15866	0.28639	0.49943	0.79749						
256	0.02108	0.04057	0.08345	0.15943	0.29112	0.49702	0.80443							
128	0.04313	0.08363	0.16315	0.29745	0.51267	0.82213								
64	0.08371	0.16179	0.30895	0.52496	0.84629									
32	0.16839	0.30395	0.55091	0.86469										
16	0.30894	0.53089	0.89726											
8	0.53422	0.86650												
4	0.84477													

(e) $\mathcal{P}^{\mathcal{C}}$ for Scene: [**Veach Door**] Algorithm: [**M-MLT**] Metric: [**MSE**] True GT: [**BDPT @ 524288 spp**].

ERPT	2	4	8	16	32	64	128	256	512	1024	2048	4096	8192	16384	32768	65536	131072
GT (BDPT)																	
262144	0.00056	0.00106	0.00123	0.00060	0.00146	0.00100	0.00196	0.00135	0.00166	0.00113	0.00145	0.00130	0.00194	0.00124	0.00203	0.00154	0.00110
131072	0.00321	0.00315	0.00391	0.00362	0.00432	0.00403	0.00485	0.00425	0.00460	0.00419	0.00359	0.00425	0.00484	0.00291	0.00324	0.00432	
65536	0.00610	0.00636	0.00863	0.00760	0.00758	0.00859	0.00820	0.00861	0.00839	0.00790	0.00676	0.00882	0.00798	0.00747	0.00790		
32768	0.01303	0.01300	0.01642	0.01591	0.01772	0.01581	0.01618	0.01542	0.01662	0.01661	0.01453	0.01627	0.01698	0.01546			
16384	0.02062	0.02444	0.02647	0.02935	0.03073	0.03080	0.02914	0.03058	0.02729	0.03193	0.02999	0.03126	0.02944				
8192	0.04174	0.04153	0.05472	0.05858	0.05706	0.06126	0.05993	0.06235	0.05539	0.06209	0.05971	0.05906					
4096	0.08841	0.09406	0.10474	0.11416	0.11293	0.11675	0.11460	0.12012	0.11452	0.11674	0.11821						
2048	0.16736	0.17095	0.19909	0.21436	0.21421	0.21454	0.21079	0.21801	0.21263	0.21586							
1024	0.30766	0.32456	0.36338	0.39176	0.38765	0.39776	0.38693	0.39463	0.38402								
512	0.53892	0.55911	0.63200	0.65907	0.66243	0.67095	0.65946	0.67653									
256	0.86061	0.88683	0.97186	1.02529	1.02840	1.03822	1.03397										
128	1.29715	1.34293	1.44793	1.50644	1.51028	1.51551											
64	1.81570	1.85525	1.97594	2.04208	2.04309												
32	2.39316	2.44020	2.56538	2.63477													
16	3.00176	3.05333	3.17783														
8	3.62338	3.67783															
4	4.19733																

(f) $\mathcal{P}^{\mathcal{C}}$ for Scene: [**Veach Door**] Algorithm: [**ERPT**] Metric: [**MSE**] True GT: [**BDPT @ 524288 spp**].

M-ERPT	2	4	8	16	32	64	128	256	512	1024	2048	4096	8192	16384	32768	65536	131072
GT (BDPT)																	
262144	0.00030	0.00104	0.00112	0.00100	0.00136	0.00123	0.00157	0.00096	0.00125	0.00186	0.00050	0.00102	0.00128	0.00084	0.00168	0.00147	0.00105
131072	0.00228	0.00246	0.00352	0.00320	0.00271	0.00352	0.00363	0.00341	0.00302	0.00407	0.00317	0.00373	0.00402	0.00299	0.00312	0.00382	
65536	0.00576	0.00639	0.00601	0.00669	0.00754	0.00725	0.00839	0.00770	0.00662	0.00689	0.00636	0.00770	0.00860	0.00655	0.00770		
32768	0.00974	0.01266	0.01217	0.01324	0.01577	0.01425	0.01394	0.01357	0.01530	0.01342	0.01413	0.01433	0.01471	0.01406			
16384	0.01782	0.02247	0.02362	0.02681	0.02600	0.02762	0.02646	0.02635	0.02469	0.02637	0.02617	0.02791	0.02772				
8192	0.03740	0.04436	0.04727	0.04982	0.05134	0.04936	0.05123	0.05399	0.05153	0.05127	0.05521	0.05321					
4096	0.07666	0.09156	0.09896	0.09682	0.10284	0.09905	0.10372	0.10381	0.10670	0.10458	0.10195						
2048	0.14818	0.17337	0.18620	0.18370	0.18853	0.18980	0.19275	0.19655	0.19477	0.19214							
1024	0.27577	0.31934	0.34428	0.34090	0.35341	0.35050	0.36015	0.36302	0.35598								
512	0.48646	0.55076	0.57719	0.59051	0.60011	0.60021	0.61268	0.61473									
256	0.79510	0.88267	0.92277	0.93885	0.94698	0.94488	0.96032										
128	1.21227	1.31854	1.37551	1.39881	1.41536	1.40188											
64	1.71024	1.84263	1.89491	1.92638	1.94213												
32	2.28296	2.42248	2.48188	2.51297													
16	2.88349	3.02786	3.09815														
8	3.50184	3.65384															
4	4.07506																

(g) $\mathcal{P}^{\mathcal{C}}$ for Scene: [**Veach Door**] Algorithm: [**M-ERPT**] Metric: [**MSE**] True GT: [**BDPT @ 524288 spp**].

Table 14

4.5 Sponza

PT	2	4	8	16	32	64	128	256	512	1024	2048	4096	8192
GT (PT)													
16384	0.00013	0.00014	0.00025	0.00075	0.00101	0.00191	0.00273	0.00960	0.01533	0.02849	0.05797	0.10462	0.17329
8192	0.00007	0.00039	0.00063	0.00122	0.00360	0.00610	0.00991	0.02328	0.04781	0.08622	0.15935	0.27957	
4096	0.00049	0.00117	0.00209	0.00369	0.00779	0.01399	0.02516	0.05400	0.10552	0.18874	0.34219		
2048	0.00098	0.00177	0.00336	0.00631	0.01510	0.02813	0.05388	0.11070	0.20442	0.36827			
1024	0.00228	0.00470	0.00755	0.01522	0.03042	0.06047	0.11449	0.21397	0.38680				
512	0.00531	0.00963	0.01597	0.03155	0.05999	0.11415	0.22079	0.39267					
256	0.00939	0.01693	0.03017	0.06003	0.11798	0.21847	0.39376						
128	0.01901	0.03260	0.06080	0.11679	0.22366	0.40518							
64	0.03584	0.06391	0.11937	0.22451	0.40465								
32	0.07001	0.12282	0.21677	0.39685									
16	0.13089	0.22673	0.38950										
8	0.23057	0.39278											
4	0.37528												

(a) $\mathcal{P}^{\mathcal{C}}$ for Scene: [**Sponza**] Algorithm: [**PT**] Metric: [**MSE**] True GT: [**PT @ 32768 spp**].

BDPT	2	4	8	16	32	64	128	256	512	1024	2048	4096	8192
GT (PT)													
16384	-0.00013	-0.00013	0.00017	0.00032	0.00067	0.00102	0.00281	0.00560	0.01288	0.02679	0.04360	0.08090	0.14387
8192	0.00014	0.00018	0.00067	0.00093	0.00242	0.00489	0.00863	0.01820	0.03628	0.07255	0.12729	0.23111	
4096	0.00025	0.00047	0.00150	0.00266	0.00620	0.01050	0.02132	0.04176	0.08105	0.15959	0.28747		
2048	0.00106	0.00169	0.00330	0.00564	0.01153	0.02242	0.04567	0.08903	0.16929	0.31238			
1024	0.00172	0.00239	0.00688	0.01168	0.02366	0.04597	0.08890	0.17184	0.32242				
512	0.00326	0.00753	0.01180	0.02532	0.04663	0.09072	0.17856	0.32939					
256	0.00711	0.01294	0.02408	0.04630	0.09150	0.16940	0.32908						
128	0.01500	0.02625	0.04973	0.08853	0.17549	0.32909							
64	0.02663	0.05035	0.09275	0.17257	0.32280								
32	0.05330	0.10165	0.17426	0.31619									
16	0.10526	0.17553	0.31622										
8	0.18872	0.31523											
4	0.31101												

(b) $\mathcal{P}^{\mathcal{C}}$ for Scene: [**Sponza**] Algorithm: [**BDPT**] Metric: [**MSE**] True GT: [**PT @ 32768 spp**].

PSSMLT	2	4	8	16	32	64	128	256	512	1024	2048	4096	8192
GT (PT)													
16384	0.00015	0.00036	0.00023	0.00049	0.00076	0.00093	0.00258	0.00397	0.00747	0.01015	0.01197	0.01467	0.01533
8192	0.00052	0.00067	0.00124	0.00235	0.00300	0.00461	0.00922	0.01341	0.02162	0.02861	0.03603	0.04334	
4096	0.00072	0.00150	0.00243	0.00411	0.00739	0.01193	0.02127	0.03318	0.05048	0.06609	0.08123		
2048	0.00156	0.00276	0.00463	0.00833	0.01491	0.02383	0.04310	0.06992	0.10438	0.14065			
1024	0.00313	0.00509	0.00958	0.01763	0.02895	0.05129	0.08778	0.14014	0.20843				
512	0.00744	0.01263	0.02082	0.03385	0.06168	0.10201	0.17116	0.26945					
256	0.01374	0.02281	0.03963	0.06853	0.11980	0.19778	0.31890						
128	0.02961	0.04699	0.07840	0.13316	0.22949	0.36561							
64	0.05423	0.08978	0.15361	0.25459	0.41210								
32	0.10230	0.16598	0.27188	0.44339									
16	0.19455	0.30813	0.48711										
8	0.33599	0.51119											
4	0.53551												

(c) $\mathcal{P}^{\mathcal{C}}$ for Scene: [**Sponza**] Algorithm: [**PSSMLT**] Metric: [**MSE**] True GT: [**PT @ 32768 spp**].

MLT	2	4	8	16	32	64	128	256	512	1024	2048	4096	8192
GT (PT)													
16384	0.00027	0.00046	0.00054	0.00087	0.00210	0.00282	0.00458	0.00835	0.01064	0.01306	0.01604	0.01592	0.01507
8192	0.00036	0.00055	0.00227	0.00322	0.00599	0.00962	0.01916	0.02448	0.03386	0.03958	0.04609	0.04428	
4096	0.00127	0.00250	0.00496	0.00836	0.01513	0.02440	0.04010	0.05812	0.07580	0.09182	0.09811		
2048	0.00233	0.00457	0.00901	0.01620	0.03252	0.05441	0.08618	0.12132	0.15831	0.18453			
1024	0.00468	0.01002	0.01778	0.03505	0.06714	0.11378	0.17550	0.24095	0.30683				
512	0.00984	0.01948	0.03639	0.06924	0.13002	0.21055	0.32650	0.43908					
256	0.01823	0.03886	0.07180	0.14206	0.24619	0.39501	0.56840						
128	0.03638	0.07246	0.14065	0.25681	0.44317	0.68075							
64	0.06707	0.13785	0.25940	0.45428	0.73209								
32	0.12827	0.24960	0.46037	0.75337									
16	0.23200	0.44399	0.75290										
8	0.39902	0.71775											
4	0.63080												

(d) $\mathcal{P}^{\mathcal{C}}$ for Scene: [**Sponza**] Algorithm: [**MLT**] Metric: [**MSE**] True GT: [**PT @ 32768 spp**].

M-MLT	2	4	8	16	32	64	128	256	512	1024	2048	4096	8192
GT (PT)													
16384	0.00040	0.00031	0.00088	0.00062	0.00229	0.00286	0.00614	0.00930	0.01085	0.01388	0.01431	0.01509	0.01574
8192	0.00020	0.00073	0.00172	0.00317	0.00703	0.01153	0.01877	0.02612	0.03486	0.03827	0.04185	0.04327	
4096	0.00147	0.00191	0.00397	0.00766	0.01615	0.02775	0.04123	0.06254	0.07616	0.09113	0.09647		
2048	0.00247	0.00407	0.00923	0.01818	0.03298	0.05534	0.08979	0.12653	0.15875	0.18230			
1024	0.00611	0.00895	0.01864	0.03605	0.06480	0.11474	0.17540	0.24718	0.30552				
512	0.01179	0.01842	0.03558	0.07093	0.13080	0.21463	0.33484	0.45174					
256	0.02389	0.03389	0.07200	0.13951	0.24484	0.39430	0.58203						
128	0.04622	0.07016	0.14008	0.26348	0.44667	0.67864							
64	0.08749	0.13311	0.26180	0.46669	0.75010								
32	0.16060	0.24659	0.45621	0.77026									
16	0.29475	0.43861	0.76311										
8	0.49127	0.70986											
4	0.75425												

(e) $\mathcal{P}^{\mathcal{C}}$ for Scene: [**Sponza**] Algorithm: [**M-MLT**] Metric: [**MSE**] True GT: [**PT @ 32768 spp**].

ERPT	2	4	8	16	32	64	128	256	512	1024	2048	4096	8192
GT (PT)													
16384	0.00829	0.00751	0.00643	0.00576	0.00766	0.00802	0.00850	0.00734	0.00748	0.00777	0.00873	0.00732	0.00673
8192	0.02070	0.02110	0.02175	0.02064	0.02277	0.02260	0.02176	0.02092	0.02155	0.02015	0.02145	0.02252	
4096	0.04605	0.04693	0.04765	0.04980	0.05100	0.05229	0.04960	0.04970	0.04954	0.05103	0.04922		
2048	0.09440	0.10004	0.10179	0.10196	0.10317	0.10599	0.10433	0.10562	0.10726	0.10280			
1024	0.18955	0.19919	0.20389	0.20265	0.20853	0.20848	0.20817	0.20219	0.20427				
512	0.35381	0.36928	0.37108	0.37689	0.37899	0.38584	0.38060	0.38139					
256	0.61094	0.63228	0.64541	0.65113	0.65636	0.65580	0.65511						
128	0.99732	1.02676	1.03886	1.05165	1.05315	1.05605							
64	1.46969	1.50782	1.52642	1.53818	1.54051								
32	2.02629	2.06171	2.07669	2.09415									
16	2.61952	2.66523	2.67977										
8	3.20897	3.25313											
4	3.75588												

(f) $\mathcal{P}^{\mathcal{C}}$ for Scene: [**Sponza**] Algorithm: [**ERPT**] Metric: [**MSE**] True GT: [**PT @ 32768 spp**].

M-ERPT	2	4	8	16	32	64	128	256	512	1024	2048	4096	8192
GT (PT)													
16384	0.00665	0.00663	0.00626	0.00741	0.00786	0.00584	0.00654	0.00588	0.00701	0.00580	0.00733	0.00637	0.00607
8192	0.01922	0.02164	0.02032	0.02104	0.02088	0.02099	0.01973	0.01995	0.01965	0.02000	0.02054	0.02008	
4096	0.04560	0.04776	0.04831	0.04803	0.04976	0.04650	0.04790	0.04577	0.04459	0.04668	0.04679		
2048	0.09262	0.09584	0.09846	0.09594	0.10274	0.09861	0.09485	0.09757	0.10056	0.09712			
1024	0.18771	0.18445	0.19419	0.19185	0.19746	0.19494	0.19778	0.20151	0.19794				
512	0.34073	0.35362	0.35780	0.35945	0.37030	0.36572	0.36108	0.36278					
256	0.58843	0.61640	0.62260	0.62074	0.63935	0.63316	0.62003						
128	0.97044	1.00100	1.00577	1.00131	1.02926	1.02077							
64	1.44080	1.46913	1.48303	1.49089	1.50765								
32	1.98754	2.02535	2.03925	2.04493									
16	2.58149	2.62183	2.63902										
8	3.17037	3.20975											
4	3.71860												

(g) $\mathcal{P}^{\mathcal{C}}$ for Scene: [**Sponza**] Algorithm: [**M-ERPT**] Metric: [**MSE**] True GT: [**PT @ 32768 spp**].

Table 15

5 IW-MSE

5.1 Cornell Box

PT	2	4	8	16	32	64	128	256	512	1024	2048	4096	8192
GT (PT)													
16384	0.00126	0.00037	0.00122	0.00106	0.00617	-0.00528	0.00332	0.01323	0.01852	0.01907	0.04438	0.07692	0.16533
8192	0.00437	0.00300	0.00430	0.00490	0.00625	0.00715	0.02290	0.01737	0.06094	0.09316	0.13770	0.26311	
4096	0.00637	0.00811	0.00839	0.00894	0.01675	0.01454	0.03636	0.05907	0.09627	0.20161	0.31313		
2048	0.01187	0.01193	0.01422	0.01643	0.02101	0.03648	0.06324	0.12519	0.23114	0.36796			
1024	0.01452	0.01584	0.02075	0.02696	0.03367	0.07238	0.13710	0.17810	0.43411				
512	0.02251	0.02359	0.02884	0.05070	0.07160	0.11059	0.19954	0.36202					
256	0.02300	0.03324	0.04071	0.08268	0.12202	0.22868	0.46667						
128	0.03110	0.03318	0.05302	0.09947	0.19953	0.37929							
64	0.04177	0.05478	0.10712	0.16150	0.35728								
32	0.06719	0.09258	0.18066	0.30936									
16	0.07992	0.13911	0.22908										
8	0.10017	0.21965											
4	0.16770												

(a) $\mathcal{P}^{\mathcal{C}}$ for Scene: **[Cornell Box]** Algorithm: **[PT]** Metric: **[IW-MSE]** True GT: **[PT @ 32768 spp]**.

BDPT	2	4	8	16	32	64	128	256	512	1024	2048	4096	8192
GT (PT)													
16384	0.00063	0.00138	0.00360	0.00465	0.00086	0.00640	0.00615	0.01178	0.04948	0.05407	0.13049	0.14643	0.22085
8192	0.00459	0.00421	0.00710	0.01233	0.00901	0.01300	0.04149	0.05610	0.14075	0.18494	0.33533	0.47047	
4096	0.00588	0.00921	0.01224	0.01174	0.01941	0.03416	0.06208	0.13990	0.24290	0.42330	0.67251		
2048	0.00870	0.00966	0.01906	0.02987	0.04325	0.08061	0.15877	0.26274	0.48209	0.71892			
1024	0.01717	0.01922	0.02640	0.04652	0.07091	0.11045	0.28160	0.51316	0.83843				
512	0.02459	0.03239	0.05601	0.09526	0.14332	0.26914	0.45763	0.82802					
256	0.03609	0.05727	0.09649	0.17918	0.27186	0.47272	0.87363						
128	0.04885	0.08298	0.12904	0.23796	0.48186	0.82753							
64	0.07495	0.15726	0.28439	0.48275	0.79898								
32	0.12505	0.21827	0.41245	0.82819									
16	0.18844	0.35644	0.68949										
8	0.31365	0.61596											
4	0.62139												

(b) $\mathcal{P}^{\mathcal{C}}$ for Scene: **[Cornell Box]** Algorithm: **[BDPT]** Metric: **[IW-MSE]** True GT: **[PT @ 32768 spp]**.

PSSMLT	2	4	8	16	32	64	128	256	512	1024	2048	4096	8192
GT (PT)													
16384	0.00134	0.00174	0.00161	0.00187	0.00300	0.00956	0.01499	0.01564	0.01487	0.03689	0.04016	0.04853	0.03560
8192	0.00556	0.00651	0.00538	0.00777	0.00766	0.01755	0.02167	0.02988	0.04999	0.06609	0.08702	0.10108	
4096	0.00768	0.00967	0.01108	0.01055	0.01449	0.02583	0.03885	0.05452	0.08688	0.13528	0.18188		
2048	0.01269	0.01348	0.01849	0.02386	0.03709	0.05291	0.07725	0.13506	0.20062	0.28902			
1024	0.01808	0.02265	0.02722	0.03385	0.05329	0.10456	0.17003	0.27426	0.38241				
512	0.02825	0.03208	0.04367	0.05689	0.09338	0.17124	0.29942	0.45932					
256	0.03611	0.04967	0.07656	0.12729	0.20719	0.34940	0.57149						
128	0.04876	0.07455	0.12084	0.21236	0.36987	0.59638							
64	0.07551	0.12598	0.21407	0.35823	0.60227								
32	0.11812	0.21241	0.35121	0.61340									
16	0.15179	0.31456	0.59341										
8	0.24629	0.52964											
4	0.50144												

(c) $\mathcal{P}^{\mathcal{C}}$ for Scene: **[Cornell Box]** Algorithm: **[PSSMLT]** Metric: **[IW-MSE]** True GT: **[PT @ 32768 spp]**.

MLT	2	4	8	16	32	64	128	256	512	1024	2048	4096	8192
GT (PT)													
16384	0.00152	0.00133	0.00141	0.00087	0.00031	-0.00080	0.00039	0.00484	0.00453	0.00476	0.00133	0.01038	0.00759
8192	0.00319	0.00319	0.00295	0.00327	0.00297	0.00200	0.00435	0.00825	0.01844	0.03348	0.03735	0.04787	
4096	0.00456	0.00450	0.00435	0.00346	0.00362	0.00392	0.00921	0.01869	0.03353	0.04107	0.06195		
2048	0.00720	0.00700	0.00670	0.00435	0.00764	0.01205	0.01909	0.03454	0.07026	0.12022			
1024	0.00891	0.00826	0.00820	0.01080	0.01519	0.02604	0.04318	0.07284	0.12500				
512	0.01003	0.01040	0.01289	0.01653	0.02522	0.03762	0.07669	0.14251					
256	0.00989	0.01164	0.01786	0.02644	0.05057	0.08545	0.16326						
128	0.01070	0.01265	0.01518	0.02232	0.05180	0.11436							
64	0.01216	0.01793	0.03338	0.06337	0.13709								
32	0.02078	0.03072	0.05768	0.10902									
16	0.01942	0.04336	0.09716										
8	0.03028	0.08538											
4	0.05351												

(d) $\mathcal{P}^{\mathcal{C}}$ for Scene: **[Cornell Box]** Algorithm: **[MLT]** Metric: **[IW-MSE]** True GT: **[PT @ 32768 spp]**.

M-MLT	2	4	8	16	32	64	128	256	512	1024	2048	4096	8192
GT (PT)													
16384	0.00124	0.00111	0.00114	0.00077	0.00067	0.00058	0.00312	0.00667	0.00779	0.01063	0.01829	0.02583	0.02091
8192	0.00268	0.00333	0.00349	0.00356	0.00423	0.00385	0.00573	0.01081	0.02139	0.02071	0.03399	0.03849	
4096	0.00408	0.00477	0.00453	0.00571	0.00738	0.00826	0.01294	0.01799	0.03698	0.05111	0.06705		
2048	0.00614	0.00661	0.00706	0.00843	0.01046	0.01017	0.01616	0.03801	0.07046	0.11312			
1024	0.00812	0.00743	0.00850	0.01021	0.01430	0.01895	0.03679	0.07991	0.12759				
512	0.00847	0.01158	0.01152	0.01986	0.02713	0.03513	0.07067	0.12779					
256	0.01133	0.01190	0.01565	0.02353	0.03784	0.06276	0.12724						
128	0.00659	0.00789	0.01306	0.03157	0.05977	0.12097							
64	0.00967	0.02479	0.04056	0.06938	0.14033								
32	0.02124	0.03967	0.07741	0.15215									
16	0.01910	0.04830	0.10983										
8	0.04977	0.08899											
4	0.08057												

(e) $\mathcal{P}^{\mathcal{C}}$ for Scene: **[Cornell Box]** Algorithm: **[M-MLT]** Metric: **[IW-MSE]** True GT: **[PT @ 32768 spp]**.

ERPT	2	4	8	16	32	64	128	256	512	1024	2048	4096	8192
GT (PT)													
16384	0.00361	0.00310	0.00180	-0.00071	0.00055	0.00118	0.00014	0.00088	0.00154	0.00070	-0.00061	-0.00062	-0.00064
8192	0.00977	0.00697	0.00859	0.00342	0.00708	0.00386	0.00333	0.00804	0.00862	0.00560	0.00654	0.00879	
4096	0.00551	0.01397	0.00744	0.00588	0.00899	0.01547	0.00888	0.00951	0.00540	0.00826	0.00528		
2048	0.01954	0.01800	0.02101	0.01974	0.01874	0.02307	0.01676	0.01585	0.01413	0.02023			
1024	0.03890	0.02645	0.03759	0.03177	0.03171	0.03281	0.03510	0.02985	0.04103				
512	0.07044	0.06615	0.06017	0.07057	0.07314	0.07612	0.07913	0.07771					
256	0.14708	0.13953	0.13354	0.11931	0.14377	0.14380	0.15292						
128	0.26114	0.24403	0.24876	0.28237	0.23061	0.28942							
64	0.47650	0.44329	0.46029	0.48413	0.44510								
32	0.82389	0.77284	0.81293	0.84016									
16	1.27184	1.25340	1.29248										
8	1.88609	1.85464											
4	2.51671												

(f) $\mathcal{P}^{\mathcal{C}}$ for Scene: **[Cornell Box]** Algorithm: **[ERPT]** Metric: **[IW-MSE]** True GT: **[PT @ 32768 spp]**.

M-ERPT	2	4	8	16	32	64	128	256	512	1024	2048	4096	8192
GT (PT)													
16384	0.00511	0.00247	0.00160	0.00320	0.00292	0.00224	0.00287	0.00179	0.00279	0.00434	0.00039	0.00328	0.00246
8192	0.00843	0.00785	0.00444	0.00692	0.00805	0.00436	0.00570	0.00584	0.00270	0.00330	0.00513	0.00808	
4096	0.01362	0.00694	0.00956	0.00896	0.00953	0.00846	0.00633	0.00786	0.01252	0.00655	0.01149		
2048	0.02688	0.01893	0.01806	0.02244	0.02062	0.01627	0.02020	0.01869	0.00687	0.01932			
1024	0.03459	0.03899	0.03289	0.02659	0.03707	0.03253	0.02802	0.03629	0.02249				
512	0.06681	0.05648	0.05323	0.06940	0.06412	0.07213	0.06453	0.07040					
256	0.13243	0.15562	0.13264	0.12694	0.14551	0.12135	0.13620						
128	0.23647	0.26112	0.24456	0.25652	0.29656	0.25624							
64	0.47510	0.44906	0.42603	0.42796	0.49081								
32	0.75631	0.84547	0.74200	0.77430									
16	1.21612	1.33257	1.15352										
8	1.82801	1.91921											
4	2.48574												

(g) $\mathcal{P}^{\mathcal{C}}$ for Scene: **[Cornell Box]** Algorithm: **[M-ERPT]** Metric: **[IW-MSE]** True GT: **[PT @ 32768 spp]**.

Table 16

5.2 Torus

PT	2	4	8	16	32	64	128	256	512	1024	2048	4096	8192	16384	32768	65536	131072
GT (BDPT)																	
262144	-0.00215	-0.00370	-0.00114	-0.00306	-0.00445	-0.00105	-0.00415	-0.00485	-0.00509	-0.00543	-0.00871	-0.00689	-0.00151	0.00908	0.02337	0.06083	0.14260
131072	-0.00451	-0.00242	-0.00270	-0.00234	-0.00986	-0.00365	-0.00567	-0.00303	-0.00378	-0.00827	-0.01748	0.00775	0.00412	0.07716	0.15776	0.19763	
65536	-0.00236	-0.00091	-0.00333	-0.00538	-0.00772	-0.00845	-0.00752	-0.01029	-0.01129	-0.01126	-0.00995	0.01062	0.04386	0.13167	0.25634		
32768	0.00291	0.00195	0.00331	-0.00113	-0.00745	-0.01380	-0.01078	-0.00796	-0.00288	-0.00379	-0.00003	0.06330	0.16426	0.32761			
16384	0.00141	0.00268	0.00374	-0.00304	-0.01266	-0.00747	-0.01124	0.00015	0.00207	0.03110	0.02568	0.15281	0.27079				
8192	0.02077	0.01768	0.01686	0.00926	0.00359	-0.00225	-0.01138	0.00297	0.00381	0.02788	0.04836	0.28086					
4096	0.04585	0.04675	0.05142	0.04321	0.00106	0.01872	0.01864	0.02144	0.03437	0.09167	0.17385						
2048	0.07248	0.06761	0.09533	0.07321	0.03913	0.03785	0.04894	0.06140	0.08123	0.17339							
1024	0.06661	0.07579	0.10057	0.06810	0.02066	0.03020	0.04612	0.08529	0.14332								
512	0.09436	0.10384	0.13760	0.10741	0.05564	0.07027	0.07158	0.06269									
256	0.04651	0.04491	0.11101	0.03609	-0.02349	0.00746	0.00298										
128	-0.09527	-0.11274	-0.05680	-0.12398	-0.16817	-0.18558											
64	-0.23147	-0.22710	-0.20423	-0.26578	-0.30953												
32	-0.42949	-0.44536	-0.42251	-0.44233													
16	-0.63731	-0.70340	-0.69674														
8	-0.77202	-0.76751															
4	-0.95130																

(a) $\mathcal{P}^{\mathcal{C}}$ for Scene: [**Torus**] Algorithm: [**PT**] Metric: [**IW-MSE**] True GT: [**BDPT @ 524288 spp**].

BDPT	2	4	8	16	32	64	128	256	512	1024	2048	4096	8192	16384	32768	65536	131072
GT (BDPT)																	
262144	-0.00326	-0.00357	-0.00383	-0.00196	-0.00133	-0.00434	-0.00375	-0.00311	-0.00423	-0.00698	0.00145	-0.01130	0.00863	0.00871	-0.01037	0.04491	0.12034
131072	-0.00382	-0.00417	-0.00536	-0.00319	-0.00762	-0.00611	-0.00516	-0.00778	-0.00923	-0.00480	-0.00202	-0.00860	0.02040	0.02635	0.07883	0.19925	
65536	-0.00359	-0.00263	-0.00558	-0.00621	-0.00615	-0.00808	-0.00981	-0.00816	-0.00888	-0.01264	-0.00037	-0.00210	0.03546	0.14900	0.18562		
32768	-0.00080	0.00874	0.00131	0.00398	-0.00813	-0.00678	-0.00567	-0.00658	-0.01000	0.00781	0.02223	0.05871	0.14034	0.31065			
16384	-0.00269	0.01019	-0.00105	0.00194	-0.00817	-0.01022	-0.00766	-0.00170	-0.00208	0.01850	0.05753	0.11965	0.24487				
8192	0.00871	0.02596	0.00688	0.00482	-0.01323	-0.00442	0.00223	-0.01500	0.00453	0.02825	0.07306	0.20031					
4096	0.02793	0.05462	0.03352	0.04912	0.01709	0.01769	0.01735	0.03062	0.02973	0.05922	0.19419						
2048	0.06576	0.09787	0.05951	0.05743	0.03823	0.03605	0.01721	0.02599	0.08023	0.14168							
1024	0.05599	0.09537	0.06837	0.04792	0.03779	0.01234	0.06398	0.04470	0.11405								
512	0.07283	0.13491	0.11434	0.09084	0.06527	0.04991	0.06826	0.02267									
256	0.01980	0.08226	0.03935	0.03065	-0.01660	0.01746	-0.03942										
128	-0.13043	-0.06233	-0.09555	-0.14738	-0.21699	-0.17447											
64	-0.24398	-0.21042	-0.25058	-0.26616	-0.34612												
32	-0.44712	-0.45707	-0.45881	-0.49225													
16	-0.69595	-0.68851	-0.65335														
8	-0.79191	-0.80888															
4	-0.99681																

(b) $\mathcal{P}^{\mathcal{C}}$ for Scene: [**Torus**] Algorithm: [**BDPT**] Metric: [**IW-MSE**] True GT: [**BDPT @ 524288 spp**].

PSSMLT	2	4	8	16	32	64	128	256	512	1024	2048	4096	8192	16384
GT (BDPT)														
262144	-0.00137	-0.00200	-0.00094	-0.00223	-0.00355	-0.00607	-0.00590	-0.00556	-0.00738	-0.00433	-0.00726	-0.00874	-0.01980	-0.00844
131072	0.00030	-0.00293	-0.00377	-0.00478	-0.00606	-0.00749	-0.00936	-0.00595	-0.00934	-0.00541	-0.00363	-0.00498	-0.01561	0.00154
65536	-0.00152	-0.00181	0.00157	-0.00276	-0.00181	-0.00516	-0.00737	-0.00240	-0.01434	-0.00387	0.00549	0.00616	0.01299	0.03651
32768	-0.00205	-0.00060	0.00026	-0.00152	-0.00198	-0.00640	-0.00836	0.00352	0.00355	0.02328	0.03050	0.04137	0.05778	0.09537
16384	0.00463	0.00791	0.00612	0.01314	0.01211	0.01323	0.02046	0.03693	0.03327	0.07441	0.11170	0.11885	0.18371	
8192	0.00750	0.00600	0.01047	0.00339	0.01457	0.01760	0.02654	0.06637	0.09943	0.16348	0.26208	0.27749		
4096	0.01187	0.02246	0.01646	0.00636	0.02886	0.04268	0.07637	0.13220	0.19038	0.28587	0.40612			
2048	0.05585	0.06538	0.07673	0.08331	0.10320	0.12611	0.17314	0.28232	0.36129	0.55284				
1024	0.06497	0.08598	0.09622	0.10466	0.13422	0.16207	0.27553	0.40500	0.60738					
512	0.07466	0.09674	0.10254	0.08672	0.15700	0.21863	0.38081	0.63442						
256	0.07184	0.09247	0.11784	0.12633	0.23252	0.38463	0.62665							
128	-0.00027	0.02466	0.06299	0.07538	0.27986	0.48753								
64	-0.01331	0.01806	0.05258	0.08055	0.36203									
32	-0.10266	-0.04637	0.00821	0.05205										
16	-0.19053	-0.11538	-0.02974											
8	-0.24105	-0.16755												
4	-0.29975													

(c) $\mathcal{P}^{\mathcal{C}}$ for Scene: [**Torus**] Algorithm: [**PSSMLT**] Metric: [**IW-MSE**] True GT: [**BDPT @ 524288 spp**].

MLT	2	4	8	16	32	64	128	256	512	1024	2048	4096	8192	16384
GT (BDPT)														
262144	-0.00101	-0.00113	-0.00053	-0.00258	-0.00209	-0.00231	-0.00225	-0.00240	-0.00363	-0.00230	-0.00311	-0.00328	-0.00376	-0.00677
131072	-0.00429	-0.00155	-0.00165	-0.00206	-0.00300	-0.00364	-0.00391	-0.00506	-0.00745	-0.00781	-0.00676	-0.00859	-0.00942	-0.00960
65536	-0.01044	-0.00601	-0.00643	-0.00879	-0.00717	-0.00870	-0.00767	-0.00755	-0.00847	-0.00711	-0.00665	-0.00764	-0.00879	-0.00792
32768	-0.01348	-0.01151	-0.00861	-0.01035	-0.00735	-0.01286	-0.00764	-0.00341	-0.00803	-0.00850	-0.00721	-0.00908	-0.00572	0.00164
16384	-0.02194	-0.01695	-0.01539	-0.01352	-0.00656	-0.01431	-0.00358	-0.00739	-0.00711	-0.00286	-0.00311	0.00180	0.00562	
8192	-0.01945	-0.00826	0.00378	-0.00086	0.01050	0.00942	0.03311	0.03809	0.03811	0.04568	0.05507	0.05894		
4096	-0.02437	-0.00168	0.01003	0.02751	0.05492	0.04451	0.09740	0.10705	0.11562	0.13103	0.14959			
2048	-0.02630	0.01930	0.04481	0.06466	0.12079	0.11247	0.17757	0.19689	0.20864	0.22214				
1024	-0.00510	0.06841	0.08448	0.12789	0.22349	0.22492	0.31665	0.33950	0.35051					
512	0.00318	0.08855	0.13688	0.18111	0.30860	0.30864	0.42264	0.47578						
256	-0.00494	0.12621	0.18771	0.25477	0.40871	0.42296	0.56102							
128	-0.02722	0.10967	0.17516	0.25130	0.40994	0.42175								
64	-0.03166	0.12567	0.17855	0.27764	0.48508									
32	-0.08788	0.07889	0.13302	0.24858										
16	-0.13918	0.02386	0.07071											
8	-0.16739	-0.00920												
4	-0.21499													

(d) $\mathcal{P}^{\mathcal{C}}$ for Scene: [**Torus**] Algorithm: [**MLT**] Metric: [**IW-MSE**] True GT: [**BDPT @ 524288 spp**].

M-MLT	2	4	8	16	32	64	128	256	512	1024	2048	4096	8192	16384
GT (BDPT)														
262144	-0.00068	-0.00005	-0.00136	-0.00244	-0.00295	-0.00428	-0.00153	0.00047	-0.00174	-0.00102	0.00060	0.00016	-0.00123	-0.00320
131072	-0.00234	-0.00265	-0.00147	-0.00572	-0.00417	-0.00203	-0.00111	-0.00476	-0.00383	-0.00103	-0.00159	-0.00108	-0.00218	-0.00333
65536	-0.00674	-0.00497	-0.00525	-0.00937	-0.00629	-0.00890	-0.00491	-0.00275	-0.00421	-0.00337	0.00149	-0.00178	0.00045	0.00260
32768	-0.01428	-0.00993	-0.00278	-0.01041	-0.00397	-0.00833	0.00224	-0.00136	0.00202	0.00161	0.00677	0.01152	0.00790	0.02145
16384	-0.02014	-0.01320	-0.00677	-0.02238	-0.00486	-0.00667	0.00498	0.01353	0.01925	0.02055	0.03633	0.04103	0.04468	
8192	-0.01682	-0.00522	0.00228	-0.00963	0.02139	0.02865	0.04096	0.06061	0.09267	0.08754	0.12429	0.13020		
4096	-0.00984	0.00307	0.03650	0.00182	0.05270	0.08001	0.11075	0.14697	0.18870	0.20277	0.25221			
2048	-0.00084	0.01723	0.07263	0.04450	0.14227	0.15042	0.20052	0.26429	0.33372	0.35102				
1024	0.02402	0.05854	0.14122	0.10335	0.20976	0.26962	0.35836	0.43196	0.52668					
512	0.03903	0.08458	0.22428	0.15403	0.28045	0.35895	0.47163	0.57794						
256	0.05073	0.11243	0.28287	0.20546	0.38677	0.48092	0.63739							
128	0.03225	0.08953	0.30338	0.19974	0.38291	0.51016								
64	0.03501	0.09307	0.36680	0.25253	0.46438									
32	-0.01143	0.06132	0.35570	0.21461										
16	-0.04840	0.02995	0.30929											
8	-0.08860	-0.01589												
4	-0.12723													

(e) $\mathcal{P}^{\mathcal{C}}$ for Scene: [**Torus**] Algorithm: [**M-MLT**] Metric: [**IW-MSE**] True GT: [**BDPT @ 524288 spp**].

ERPT	2	4	8	16	32	64	128	256	512	1024	2048	4096	8192	16384	32768	65536	131072
GT (BDPT)																	
262144	-0.00420	-0.00483	-0.00430	-0.00249	0.00428	-0.00029	-0.00016	-0.00477	0.00006	-0.00972	-0.00790	-0.00518	-0.00575	-0.01607	-0.00927	-0.01125	-0.00833
131072	-0.00487	-0.00235	-0.00606	-0.00040	0.00096	0.00642	0.00392	0.00338	0.01184	0.01000	0.00778	0.02381	0.01788	0.01644	-0.00056	0.00434	
65536	-0.00291	0.00048	-0.00145	0.00168	0.01070	-0.00098	0.01222	0.01409	0.01550	0.01914	0.02985	0.04031	0.04465	0.03507	0.02411		
32768	0.00069	-0.00133	-0.01084	0.01027	0.03563	0.01808	0.01978	0.04122	0.04229	0.07183	0.08534	0.09457	0.11061	0.07874			
16384	0.01969	0.02058	0.01447	0.02667	0.03985	0.04333	0.05900	0.08793	0.07789	0.14259	0.20504	0.24311	0.24935				
8192	0.04397	0.06811	0.05873	0.07605	0.10439	0.11247	0.17846	0.21779	0.23725	0.34394	0.41697	0.46534					
4096	0.10646	0.11028	0.12870	0.16150	0.23262	0.25078	0.32526	0.43010	0.45950	0.60003	0.65899						
2048	0.17607	0.24195	0.29138	0.32699	0.40342	0.43308	0.58301	0.76703	0.80097	1.01227							
1024	0.29320	0.41536	0.38899	0.52700	0.65352	0.68628	0.93172	1.16009	1.25143								
512	0.35871	0.55271	0.58349	0.74147	0.86201	0.96749	1.16786	1.49247									
256	0.48407	0.74933	0.81567	0.90836	1.08243	1.23940	1.49627										
128	0.50490	0.79172	0.86883	1.03116	1.19899	1.40417											
64	0.58857	0.91483	1.01141	1.13816	1.29212												
32	0.61523	0.93107	1.01665	1.14044													
16	0.53261	0.88619	0.98212														
8	0.48668	0.84738															
4	0.43223																

(f) $\mathcal{P}^{\mathcal{C}}$ for Scene: [**Torus**] Algorithm: [**ERPT**] Metric: [**IW-MSE**] True GT: [**BDPT @ 524288 spp**].

M-ERPT	2	4	8	16	32	64	128	256	512	1024	2048	4096	8192	16384	32768	65536	131072
GT (BDPT)																	
262144	0.00017	-0.00046	-0.00240	-0.00030	-0.00245	-0.00434	0.00344	0.00016	-0.00118	-0.00085	-0.00792	-0.00216	-0.00301	0.00190	-0.00458	-0.00320	0.00169
131072	-0.00158	0.00010	-0.00390	-0.00710	-0.00690	-0.01148	0.00466	0.00449	0.00854	0.00328	0.01870	0.01057	0.00810	0.00703	0.00685	0.01185	
65536	-0.00527	-0.00429	0.00177	-0.00049	0.00906	-0.00310	0.01056	0.02627	0.03631	0.03260	0.03827	0.02498	0.02622	0.03484	0.03052		
32768	0.00301	0.00504	-0.00220	0.00418	0.01218	0.03276	0.04569	0.06722	0.06849	0.07292	0.10828	0.09183	0.07822	0.10006			
16384	0.01051	0.01313	0.02449	0.02109	0.03055	0.05801	0.08046	0.10094	0.15016	0.16938	0.22819	0.22367	0.19313				
8192	0.03991	0.04237	0.07348	0.07371	0.10498	0.14129	0.20763	0.24296	0.34863	0.36931	0.46499	0.42672					
4096	0.10019	0.10677	0.14989	0.17496	0.20035	0.29399	0.37872	0.43496	0.56726	0.61450	0.74705						
2048	0.18785	0.17161	0.27205	0.30176	0.36281	0.50812	0.66996	0.74191	0.95342	1.01751							
1024	0.33114	0.29904	0.43016	0.48415	0.62318	0.81708	1.02823	1.12882	1.40624								
512	0.42307	0.42016	0.53334	0.64875	0.80519	1.05722	1.28383	1.46631									
256	0.53906	0.51018	0.70361	0.80704	1.02690	1.34526	1.59988										
128	0.55911	0.52488	0.76741	0.90305	1.14978	1.51878											
64	0.62597	0.58164	0.80400	1.00423	1.23304												
32	0.59118	0.55992	0.82126	0.97288													
16	0.48958	0.49874	0.76124														
8	0.42884	0.46844															
4	0.38028																

(g) $\mathcal{P}^{\mathcal{C}}$ for Scene: [**Torus**] Algorithm: [**M-ERPT**] Metric: [**IW-MSE**] True GT: [**BDPT @ 524288 spp**].

Table 17

5.3 Veach Bidir

PT	2	4	8	16	32	64	128	256	512	1024	2048	4096	8192	16384	32768	65536	131072
GT (BDPT)																	
262144	-0.00031	-0.00035	-0.00053	-0.00076	-0.00015	-0.00058	-0.00103	-0.00028	-0.00152	-0.00281	-0.00310	-0.00437	-0.00419	-0.00209	-0.00763	-0.00547	-0.01137
131072	-0.00029	-0.00076	-0.00023	-0.00041	-0.00045	-0.00100	-0.00057	-0.00038	-0.00138	-0.00184	-0.00271	-0.00228	-0.00152	-0.00670	-0.00820	-0.00312	
65536	-0.00024	-0.00049	-0.00057	-0.00142	-0.00082	-0.00117	-0.00252	0.00124	-0.00049	-0.00292	-0.00590	-0.00648	-0.00342	-0.00457	-0.00409		
32768	-0.00016	-0.00077	-0.00099	-0.00127	-0.00133	-0.00170	-0.00150	0.00030	0.00035	-0.00029	-0.00306	-0.00283	-0.00047	0.00370			
16384	-0.00131	-0.00335	-0.00505	-0.00616	-0.00793	-0.01003	-0.00826	-0.01090	-0.01441	-0.00901	-0.00928	-0.00241	0.00308				
8192	-0.00135	-0.00190	-0.00460	-0.00609	-0.00552	-0.01013	-0.00761	-0.01105	-0.01199	-0.00672	-0.00546	0.00718					
4096	0.00070	-0.00022	0.00066	-0.00033	0.00111	-0.00095	0.00288	0.00685	0.00104	0.00083	-0.00083						
2048	-0.00194	-0.00302	-0.00730	-0.00909	-0.01515	-0.02076	-0.01147	-0.01874	-0.01819	-0.00103							
1024	-0.00678	-0.01282	-0.02636	-0.03441	-0.03753	-0.06249	-0.03870	-0.05454	-0.06816								
512	-0.01053	-0.01963	-0.03753	-0.04860	-0.04640	-0.09522	-0.04244	-0.05919									
256	-0.01021	-0.01726	-0.03709	-0.04733	-0.03447	-0.09692	-0.03241										
128	-0.01321	-0.02342	-0.05620	-0.05927	-0.03193	-0.12181											
64	-0.01737	-0.02714	-0.05496	-0.06554	-0.03273												
32	-0.01971	-0.02443	-0.04588	-0.06042													
16	-0.01499	-0.02177	-0.03594														
8	-0.01384	-0.01323															
4	-0.00283																

(a) $\mathcal{P}^{\mathcal{C}}$ for Scene: [**Veach Bidir**] Algorithm: [**PT**] Metric: [**IW-MSE**] True GT: [**BDPT @ 524288 spp**].

BDPT	2	4	8	16	32	64	128	256	512	1024	2048	4096	8192	16384	32768	65536	131072
GT (BDPT)																	
262144	-0.00137	-0.00137	-0.00249	-0.00408	-0.00222	-0.00226	-0.00472	-0.00037	-0.00228	-0.03318	0.04872	0.04256	-0.05911	-0.09508	-0.02148	-0.01360	0.04910
131072	-0.00196	-0.00258	-0.00220	-0.00149	0.00097	0.00032	0.00107	-0.00152	0.00862	0.01876	0.05205	-0.12296	-0.01733	0.00165	0.01930	-0.12079	
65536	-0.00241	-0.00339	-0.00328	-0.00801	-0.00024	0.00116	0.00217	-0.00241	-0.00750	0.00534	0.07724	-0.21177	0.01340	0.21724	0.11016		
32768	-0.00213	-0.00204	-0.00171	-0.00573	0.00252	0.00794	0.00745	0.00409	-0.02048	-0.00709	-0.01968	0.15842	0.22902	0.02737			
16384	-0.01691	-0.02506	-0.03411	-0.03768	-0.05423	-0.05295	-0.07754	-0.10031	-0.11379	-0.22261	-0.26110	0.76863	0.44959				
8192	-0.01716	-0.02701	-0.04153	-0.05310	-0.05243	-0.05544	-0.07082	-0.10003	-0.07914	-0.10791	0.08394	0.61591					
4096	-0.00417	-0.00374	-0.00132	-0.00786	0.00750	0.01245	0.02268	0.01570	0.04326	0.11634	0.48766						
2048	-0.02880	-0.05155	-0.06841	-0.08227	-0.11005	-0.10596	-0.14178	-0.16566	-0.28566	-0.16924							
1024	-0.09670	-0.15332	-0.23735	-0.33094	-0.34528	-0.40951	-0.53858	-0.82871	-0.84679								
512	-0.15710	-0.25273	-0.39373	-0.55221	-0.57085	-0.74198	-0.87298	-0.99715									
256	-0.16139	-0.26947	-0.44098	-0.60844	-0.67318	-0.86676	-0.99242										
128	-0.21734	-0.33933	-0.59004	-0.54260	-0.89464	-1.48673											
64	-0.24356	-0.42465	-0.64245	-0.57504	-0.94537												
32	-0.23757	-0.41924	-0.53317	-0.39608													
16	-0.17370	-0.29728	-0.40217														
8	-0.13019	-0.20617															
4	-0.04947																

(b) $\mathcal{P}^{\mathcal{C}}$ for Scene: [**Veach Bidir**] Algorithm: [**BDPT**] Metric: [**IW-MSE**] True GT: [**BDPT @ 524288 spp**].

PSSMLT	2	4	8	16	32	64	128	256	512	1024	2048	4096	8192	16384
GT (BDPT)														
262144	-0.00113	-0.00180	-0.00223	-0.00231	-0.00309	-0.00192	-0.00193	-0.00172	-0.02010	-0.01610	-0.04201	-0.05727	-0.03274	-0.01192
131072	-0.00180	-0.00154	-0.00137	-0.00074	-0.00042	0.00033	0.00030	0.00011	0.01141	0.00788	-0.00746	-0.00200	-0.00228	0.00030
65536	-0.00173	-0.00191	-0.00107	-0.00171	-0.00356	-0.00263	-0.00321	0.01034	-0.02619	-0.02524	-0.01241	-0.01171	0.00052	0.02224
32768	-0.00210	-0.00240	-0.00170	-0.00119	0.00035	-0.00055	-0.00050	0.00361	-0.02709	-0.01167	-0.03715	0.02214	-0.00432	0.01072
16384	-0.00839	-0.01622	-0.02394	-0.03162	-0.03591	-0.04514	-0.04658	-0.10982	-0.11075	-0.09589	-0.10586	-0.10745	-0.08323	
8192	-0.00915	-0.01680	-0.02480	-0.03767	-0.04662	-0.05146	-0.05832	-0.11930	-0.18139	-0.12348	0.00071	-0.08089		
4096	-0.00003	0.00188	0.00346	0.00577	0.00479	0.01075	0.01466	0.06370	0.12083	0.06136	0.23824			
2048	-0.01637	-0.02970	-0.04540	-0.06568	-0.07347	-0.10012	-0.11220	-0.19287	-0.04033	-0.04760				
1024	-0.04698	-0.08886	-0.14773	-0.22950	-0.29347	-0.35836	-0.40083	-0.73553	-0.49254					
512	-0.06876	-0.13374	-0.23264	-0.37617	-0.48988	-0.64876	-0.73880	-0.98398						
256	-0.07989	-0.15313	-0.25850	-0.42994	-0.56124	-0.74209	-0.87081							
128	-0.09859	-0.18788	-0.32444	-0.55444	-0.76787	-1.07359								
64	-0.10603	-0.21007	-0.39136	-0.62540	-0.85273									
32	-0.08800	-0.16897	-0.33951	-0.48867										
16	-0.05771	-0.09983	-0.18866											
8	-0.02512	-0.02545												
4	0.04303													

(c) $\mathcal{P}^{\mathcal{C}}$ for Scene: [**Veach Bidir**] Algorithm: [**PSSMLT**] Metric: [**IW-MSE**] True GT: [**BDPT @ 524288 spp**].

MLT	2	4	8	16	32	64	128	256	512	1024	2048	4096	8192	16384
GT (BDPT)														
262144	0.00013	0.00007	0.00008	0.00014	-0.00067	-0.00091	-0.00079	-0.00052	-0.00098	-0.00062	-0.00398	-0.00473	-0.00363	-0.00368
131072	-0.00023	-0.00048	-0.00056	-0.00075	-0.00160	-0.00259	-0.00191	-0.00068	0.00041	0.00184	0.00176	0.00073	0.00088	0.00090
65536	-0.00036	-0.00045	-0.00038	-0.00146	-0.00274	-0.00396	-0.00297	-0.00313	-0.00306	-0.00239	-0.00501	-0.00553	-0.00567	-0.00535
32768	0.00037	0.00044	0.00071	-0.00017	-0.00123	-0.00251	-0.00085	0.00332	0.00118	0.00177	-0.00118	0.00027	-0.00145	-0.00231
16384	-0.00025	-0.00158	-0.00304	-0.00724	-0.01358	-0.02154	-0.03064	-0.03257	-0.04275	-0.04418	-0.04242	-0.05148	-0.05354	
8192	-0.00043	-0.00168	-0.00449	-0.00992	-0.01564	-0.02447	-0.03749	-0.04440	-0.05239	-0.05520	-0.06276	-0.06739		
4096	0.00061	0.00049	0.00109	0.00056	-0.00283	-0.00287	-0.00033	-0.00057	0.00472	0.01219	0.01633			
2048	-0.00232	-0.00579	-0.01205	-0.01844	-0.03392	-0.05022	-0.07777	-0.08255	-0.10346	-0.11790				
1024	-0.00610	-0.01487	-0.02791	-0.05024	-0.08476	-0.14566	-0.23288	-0.29164	-0.36792					
512	-0.01280	-0.02543	-0.04662	-0.07593	-0.12594	-0.23884	-0.37267	-0.48276						
256	-0.01416	-0.02904	-0.05379	-0.08986	-0.15432	-0.27915	-0.45606							
128	-0.01803	-0.03660	-0.06135	-0.09885	-0.18067	-0.35011								
64	-0.02617	-0.04514	-0.07848	-0.11966	-0.19119									
32	-0.02581	-0.04729	-0.08333	-0.11245										
16	-0.02225	-0.03485	-0.05750											
8	-0.02816	-0.04169												
4	-0.02640													

(d) $\mathcal{P}^{\mathcal{C}}$ for Scene: [**Veach Bidir**] Algorithm: [**MLT**] Metric: [**IW-MSE**] True GT: [**BDPT @ 524288 spp**].

M-MLT	2	4	8	16	32	64	128	256	512	1024	2048	4096	8192	16384
GT (BDPT)														
262144	-0.00013	-0.00036	-0.00052	-0.00056	-0.00149	-0.00125	-0.00090	-0.00156	-0.00160	-0.00111	-0.00187	-0.00190	-0.00217	-0.00363
131072	-0.00040	-0.00058	-0.00090	-0.00091	-0.00209	-0.00075	-0.00053	-0.00011	0.00023	0.00227	0.00289	0.00107	0.00152	0.00181
65536	-0.00010	-0.00040	-0.00043	-0.00019	-0.00292	-0.00174	-0.00422	-0.00386	-0.00341	0.00008	-0.00014	-0.00293	-0.00246	-0.00408
32768	0.00016	0.00008	0.00007	0.00086	-0.00082	-0.00017	-0.00061	0.00009	0.00187	0.00660	0.00022	0.00183	0.00047	-0.00105
16384	-0.00052	-0.00177	-0.00166	-0.00753	-0.00938	-0.01553	-0.02107	-0.03400	-0.03525	-0.04612	-0.04906	-0.05204	-0.05615	
8192	-0.00128	-0.00369	-0.00543	-0.01087	-0.01706	-0.02656	-0.03377	-0.04539	-0.05371	-0.05493	-0.05700	-0.06480		
4096	0.00080	0.00131	0.00067	0.00244	0.00124	0.00037	0.00579	0.00359	0.01028	0.01667	0.01454			
2048	-0.00108	-0.00340	-0.00577	-0.01670	-0.02481	-0.03678	-0.05284	-0.08729	-0.09509	-0.11181				
1024	-0.00752	-0.01708	-0.02267	-0.05411	-0.08150	-0.13644	-0.19049	-0.30027	-0.33764					
512	-0.01302	-0.02952	-0.04222	-0.08976	-0.13104	-0.21471	-0.30230	-0.50675						
256	-0.01495	-0.03048	-0.04965	-0.09946	-0.15392	-0.25877	-0.37323							
128	-0.02173	-0.04400	-0.06657	-0.13424	-0.18555	-0.32409								
64	-0.02933	-0.05640	-0.07889	-0.16390	-0.22270									
32	-0.03342	-0.05851	-0.08576	-0.16341										
16	-0.02696	-0.05032	-0.06053											
8	-0.03256	-0.05500												
4	-0.03127													

(e) $\mathcal{P}^{\mathcal{C}}$ for Scene: [**Veach Bidir**] Algorithm: [**M-MLT**] Metric: [**IW-MSE**] True GT: [**BDPT @ 524288 spp**].

ERPT	2	4	8	16	32	64	128	256	512	1024	2048	4096	8192	16384	32768	65536	131072
GT (BDPT)																	
262144	-0.00143	-0.00141	-0.00240	-0.00221	-0.00273	-0.00275	-0.00504	-0.00176	-0.00412	-0.00122	-0.00077	-0.00092	-0.00197	0.00118	-0.00178	-0.00264	-0.00267
131072	0.00110	-0.00092	0.00023	-0.00052	-0.00107	-0.00434	-0.00193	-0.00209	-0.00113	-0.00539	-0.00303	-0.00313	-0.00298	-0.00143	-0.00392	-0.00447	
65536	0.00034	-0.00183	-0.00040	-0.00172	0.00123	-0.01000	-0.00176	-0.00210	0.00114	0.00018	0.00105	-0.00245	0.00133	0.00093	-0.00056		
32768	0.00120	0.00108	0.00070	-0.00146	-0.00101	0.00374	-0.00013	-0.00087	0.00422	-0.00106	0.00526	0.00063	0.00222	0.00093			
16384	-0.02642	-0.03132	-0.03394	-0.03223	-0.04387	-0.00149	-0.03281	0.00041	0.00565	0.00312	0.00608	0.01839	0.00552				
8192	-0.03933	-0.03818	-0.03841	-0.03643	-0.04132	0.00069	-0.03252	-0.02914	-0.00229	0.00868	0.00111	0.01025					
4096	0.01087	0.00230	0.00684	0.00112	0.01309	-0.00315	0.00022	-0.00639	0.03681	0.01653	0.01773						
2048	-0.06298	-0.07090	-0.07981	-0.07509	-0.09401	-0.00743	-0.06135	0.00327	0.03644	0.02508							
1024	-0.23193	-0.24260	-0.25967	-0.25081	-0.25705	0.02724	-0.22231	-0.03358	0.07716								
512	-0.38961	-0.41296	-0.43205	-0.41147	-0.36798	0.15267	-0.25845	0.05944									
256	-0.45837	-0.48637	-0.50523	-0.48205	-0.41558	0.15986	-0.26702										
128	-0.64933	-0.65287	-0.69317	-0.69015	-0.48280	0.48418											
64	-0.70975	-0.73990	-0.71339	-0.69746	-0.41018												
32	-0.60225	-0.54460	-0.53257	-0.48807													
16	-0.29777	-0.35436	-0.30879														
8	-0.09097	-0.09851															
4	0.34500																

(f) $\mathcal{P}^{\mathcal{C}}$ for Scene: [**Veach Bidir**] Algorithm: [**ERPT**] Metric: [**IW-MSE**] True GT: [**BDPT @ 524288 spp**].

M-ERPT	2	4	8	16	32	64	128	256	512	1024	2048	4096	8192	16384	32768	65536	131072
GT (BDPT)																	
262144	0.00018	-0.00248	-0.00238	-0.00512	-0.00133	-0.00084	0.00438	-0.00270	0.00180	-0.00115	-0.00033	-0.00173	-0.00177	-0.00121	-0.00210	-0.00076	-0.00190
131072	0.00042	-0.00050	-0.00256	0.00219	0.00059	-0.00125	-0.00035	-0.00145	0.00109	-0.00171	-0.00243	-0.00358	-0.00369	-0.00198	-0.00538	-0.00322	
65536	0.00078	-0.00188	-0.00298	-0.00185	-0.00057	-0.00099	0.00476	-0.00057	0.00283	-0.00346	0.00112	-0.00093	-0.00026	-0.00032	-0.00371		
32768	0.00061	0.00069	-0.00023	-0.00331	0.00111	0.00303	0.00321	-0.00165	-0.00278	0.00037	0.00281	0.00636	-0.00005	0.00009			
16384	-0.02822	-0.02886	-0.00647	-0.01010	-0.02055	0.00144	0.00429	0.00650	-0.00086	0.01983	0.01086	0.01538	0.01246				
8192	-0.03363	-0.03731	-0.00651	-0.01011	-0.02717	-0.00744	0.02999	-0.00809	0.01260	0.00853	0.01784	0.01310					
4096	0.00865	0.00150	0.02974	0.03234	0.01249	0.02992	0.02689	0.02987	0.03152	0.02644	0.02326						
2048	-0.07916	-0.06953	0.01222	0.02530	-0.02401	0.02374	0.00061	0.03007	-0.00596	0.05371							
1024	-0.22228	-0.25229	0.07255	-0.00438	-0.09078	0.09195	0.12957	0.10189	0.08686								
512	-0.37063	-0.38953	0.27701	0.10918	0.00949	0.29973	0.26241	0.30371									
256	-0.44537	-0.49368	0.28013	0.17502	0.02263	0.31795	0.29029										
128	-0.61257	-0.68692	0.61569	0.44990	0.31215	0.64178											
64	-0.66097	-0.72439	0.75945	0.59179	0.49084												
32	-0.54853	-0.54332	0.72710	0.56816													
16	-0.27970	-0.35032	0.78599														
8	-0.10752	-0.14570															
4	0.30209																

(g) $\mathcal{P}^{\mathcal{C}}$ for Scene: [**Veach Bidir**] Algorithm: [**M-ERPT**] Metric: [**IW-MSE**] True GT: [**BDPT @ 524288 spp**].

Table 18

5.4 Veach Door

PT	2	4	8	16	32	64	128	256	512	1024	2048	4096	8192	16384	32768	65536	131072
GT (BDPT)																	
262144	0.00001	0.00015	0.00006	0.00031	0.00069	0.00004	0.00029	0.00023	0.00068	0.00080	0.00036	0.00154	0.00179	0.00464	0.00288	0.02717	0.02894
131072	0.00012	0.00059	0.00031	0.00066	0.00112	0.00073	0.00164	0.00281	0.00288	0.00319	0.00470	0.00722	0.00794	0.01703	0.03616	0.04584	
65536	0.00046	0.00107	0.00113	0.00119	0.00161	0.00156	0.00218	0.00111	0.00387	0.00412	0.00840	0.00929	0.00961	0.02850	0.04978		
32768	0.00118	0.00218	0.00205	0.00204	0.00205	0.00224	0.00291	0.00328	0.00330	0.00527	0.00814	0.01440	0.02397	0.05394			
16384	0.00235	0.00344	0.00331	0.00267	0.00048	-0.00118	0.00080	-0.00114	-0.00193	0.00286	0.00614	0.01205	0.03176				
8192	0.00391	0.00571	0.00438	0.00230	0.00060	-0.00228	-0.00359	-0.00370	-0.00451	0.00123	0.00260	0.02519					
4096	0.00565	0.00763	0.00450	0.00319	0.00055	-0.00389	-0.00288	-0.00408	0.00018	0.01373	0.01999						
2048	0.00951	0.01141	0.00616	0.00193	-0.00509	-0.00560	-0.01007	-0.01212	-0.00579	0.01344							
1024	0.01410	0.01634	0.00606	-0.00056	-0.00728	-0.01392	-0.01157	-0.00473	0.00282								
512	0.01907	0.02413	0.00671	-0.00385	-0.01698	-0.01912	-0.02491	-0.01448									
256	0.02996	0.03206	0.01007	-0.00701	-0.01451	-0.01740	-0.00678										
128	0.04177	0.04793	0.01159	-0.00764	-0.01683	-0.00376											
64	0.06172	0.06802	0.02512	0.02334	0.01374												
32	0.08607	0.09687	0.04330	0.03704													
16	0.12629	0.13474	0.10585														
8	0.20142	0.24041															
4	0.29800																

(a) $\mathcal{P}^{\mathcal{C}}$ for Scene: [**Veach Door**] Algorithm: [**PT**] Metric: [**IW-MSE**] True GT: [**BDPT @ 524288 spp**].

BDPT	2	4	8	16	32	64	128	256	512	1024	2048	4096	8192	16384	32768	65536	131072
GT (BDPT)																	
262144	-0.00003	0.00027	0.00056	0.00014	0.00022	0.00017	-0.00008	0.00033	0.00266	-0.00008	0.00786	-0.00088	0.02570	0.01909	0.05568	0.10054	0.15892
131072	0.00024	0.00043	0.00075	0.00078	0.00163	0.00211	0.00243	0.00420	0.00931	0.01020	0.01903	0.01641	0.05489	0.08513	0.12113	0.22125	
65536	0.00083	0.00143	0.00134	0.00159	0.00203	0.00208	0.00221	0.00290	0.00888	0.00868	0.02202	0.04422	0.11440	0.17255	0.33436		
32768	0.00169	0.00164	0.00169	0.00251	0.00153	-0.00029	0.00169	0.00430	0.01242	0.01868	0.05378	0.06946	0.20005	0.33046			
16384	0.00263	0.00222	0.00173	-0.00092	-0.00163	-0.00218	-0.00262	-0.00130	0.00705	0.01993	0.04508	0.13452	0.30409				
8192	0.00479	0.00289	0.00050	-0.00278	-0.00525	-0.00718	-0.00564	-0.00071	0.01793	0.04934	0.11627	0.27888					
4096	0.00510	0.00381	0.00074	-0.00491	-0.00568	-0.00598	0.00071	0.00500	0.05558	0.10553	0.24638						
2048	0.00872	0.00321	-0.00326	-0.01055	-0.01649	-0.01427	-0.00439	0.01622	0.06154	0.18315							
1024	0.01240	0.00279	-0.00644	-0.01464	-0.02081	-0.01729	0.00333	0.04632	0.15035								
512	0.01898	-0.00268	-0.01189	-0.02537	-0.03486	-0.01413	0.01219	0.10565									
256	0.02570	-0.00039	-0.02386	-0.03232	-0.03736	0.00586	0.06695										
128	0.03870	0.00012	-0.02908	-0.04236	-0.02441	0.06399											
64	0.05339	0.00214	-0.01388	0.00838	0.07137												
32	0.07697	0.01869	0.01168	0.07853													
16	0.13071	0.08673	0.08702														
8	0.21142	0.20247															
4	0.35099																

(b) $\mathcal{P}^{\mathcal{C}}$ for Scene: [**Veach Door**] Algorithm: [**BDPT**] Metric: [**IW-MSE**] True GT: [**BDPT @ 524288 spp**].

PSSMLT	2	4	8	16	32	64	128	256	512	1024	2048	4096	8192	16384
GT (BDPT)														
262144	0.00014	0.00027	0.00003	-0.00024	-0.00049	0.00005	0.00035	0.00231	0.00265	0.00560	0.00562	0.00942	0.02140	0.04813
131072	0.00042	0.00017	-0.00004	-0.00023	0.00022	-0.00013	0.00037	0.00161	0.00286	0.00817	0.01411	0.02933	0.05920	0.10277
65536	0.00047	0.00020	-0.00052	-0.00121	-0.00116	-0.00118	-0.00157	-0.00015	0.00317	0.01152	0.03034	0.05306	0.09870	0.20014
32768	0.00089	-0.00031	-0.00189	-0.00256	-0.00256	-0.00096	-0.00007	0.00290	0.01192	0.02711	0.05491	0.11859	0.21405	0.36235
16384	0.00049	-0.00170	-0.00416	-0.00585	-0.00569	-0.00538	-0.00259	-0.00076	0.01220	0.03201	0.08682	0.18392	0.34301	
8192	0.00093	-0.00315	-0.00708	-0.00906	-0.00964	-0.00780	-0.00841	0.00304	0.02571	0.07047	0.19091	0.37719		
4096	0.00061	-0.00480	-0.01028	-0.01206	-0.00927	-0.00686	0.00117	0.02579	0.07738	0.19484	0.35510			
2048	-0.00052	-0.00735	-0.01415	-0.01686	-0.01368	-0.01014	0.00857	0.04490	0.13686	0.30656				
1024	-0.00040	-0.00796	-0.01515	-0.01668	-0.00478	0.00736	0.04998	0.14489	0.32244					
512	-0.00175	-0.00975	-0.01749	-0.01247	0.00722	0.04138	0.12973	0.31353						
256	0.00507	-0.00476	-0.00989	0.00899	0.05433	0.13624	0.30266							
128	0.00917	0.00492	0.00394	0.04291	0.12742	0.30402								
64	0.03041	0.03928	0.06300	0.15118	0.32045									
32	0.05289	0.09084	0.15948	0.31556										
16	0.12197	0.20140	0.37092											
8	0.25266	0.41412												
4	0.50274													

(c) $\mathcal{P}^{\mathcal{C}}$ for Scene: [**Veach Door**] Algorithm: [**PSSMLT**] Metric: [**IW-MSE**] True GT: [**BDPT @ 524288 spp**].

MLT	2	4	8	16	32	64	128	256	512	1024	2048	4096	8192	16384
GT (BDPT)														
262144	0.00024	-0.00028	-0.00029	-0.00052	-0.00072	-0.00076	-0.00102	0.00067	-0.00008	-0.00349	0.00282	0.00545	0.01310	0.01437
131072	0.00014	-0.00026	-0.00023	-0.00076	-0.00109	-0.00116	-0.00102	-0.00075	-0.00268	-0.00013	0.00818	0.01288	0.00348	0.04773
65536	0.00006	-0.00099	-0.00122	-0.00194	-0.00273	-0.00213	-0.00279	-0.00271	-0.00028	0.00100	0.01473	0.03255	0.03618	0.10119
32768	-0.00094	-0.00262	-0.00367	-0.00604	-0.00615	-0.00687	-0.00791	-0.00860	-0.00317	-0.00203	0.02751	0.07696	0.13860	0.23598
16384	-0.00200	-0.00532	-0.00781	-0.01069	-0.01274	-0.01626	-0.01644	-0.01533	-0.00710	0.00259	0.07244	0.13916	0.24499	
8192	-0.00427	-0.00870	-0.01218	-0.01646	-0.01898	-0.02435	-0.02150	-0.01437	0.00287	0.06019	0.15667	0.29709		
4096	-0.00553	-0.01135	-0.01634	-0.02403	-0.02496	-0.02584	-0.02043	0.00805	0.04719	0.13817	0.32796			
2048	-0.00969	-0.01682	-0.02517	-0.03044	-0.03250	-0.03544	-0.01708	0.01158	0.11510	0.26428				
1024	-0.01245	-0.02074	-0.02880	-0.03687	-0.03299	-0.02816	0.00309	0.09495	0.24510					
512	-0.01581	-0.02479	-0.03516	-0.03396	-0.02767	-0.01413	0.08216	0.26737						
256	-0.01339	-0.02041	-0.02319	-0.01932	-0.00216	0.05046	0.23751							
128	-0.01526	-0.00884	-0.00734	0.02162	0.07826	0.18574								
64	0.00769	0.02515	0.06045	0.13685	0.25968									
32	0.03007	0.08533	0.16276	0.33092										
16	0.09609	0.21860	0.37499											
8	0.24851	0.46436												
4	0.52750													

(d) $\mathcal{P}^{\mathcal{C}}$ for Scene: [**Veach Door**] Algorithm: [**MLT**] Metric: [**IW-MSE**] True GT: [**BDPT @ 524288 spp**].

M-MLT	2	4	8	16	32	64	128	256	512	1024	2048	4096	8192	16384
GT (BDPT)														
262144	0.00013	-0.00014	-0.00025	-0.00050	-0.00143	-0.00041	-0.00077	-0.00072	-0.00051	-0.00303	0.00167	0.00258	0.00155	0.01436
131072	0.00009	-0.00059	-0.00080	-0.00059	-0.00085	-0.00024	0.00102	-0.00106	-0.00044	-0.00207	0.00699	0.00776	0.03254	0.03300
65536	-0.00017	-0.00137	-0.00106	-0.00170	-0.00305	-0.00106	-0.00253	0.00029	0.00121	0.00029	0.00929	0.01459	0.06550	0.07769
32768	-0.00112	-0.00313	-0.00351	-0.00473	-0.00543	-0.00611	-0.00324	-0.00739	-0.00079	0.00246	0.03731	0.04161	0.12711	0.12552
16384	-0.00266	-0.00568	-0.00788	-0.00878	-0.01228	-0.01216	-0.01534	-0.01107	-0.00225	0.00466	0.06474	0.08546	0.22833	
8192	-0.00457	-0.00938	-0.01364	-0.01571	-0.01927	-0.01835	-0.02235	-0.00944	0.00439	0.04591	0.14571	0.19596		
4096	-0.00551	-0.01257	-0.01782	-0.02054	-0.02467	-0.01983	-0.00435	0.01569	0.04191	0.12590	0.30067			
2048	-0.00931	-0.01826	-0.02580	-0.02966	-0.03358	-0.02619	-0.00421	0.03898	0.09983	0.24399				
1024	-0.01209	-0.02387	-0.02920	-0.03479	-0.02967	-0.00961	0.01105	0.10823	0.23264					
512	-0.01498	-0.02950	-0.03686	-0.03828	-0.02779	0.02455	0.09916	0.25803						
256	-0.01155	-0.02625	-0.03422	-0.02268	0.00450	0.11436	0.23448							
128	-0.00703	-0.01035	-0.01554	0.04182	0.07551	0.26870								
64	0.00795	0.02222	0.05547	0.14646	0.29105									
32	0.05060	0.07862	0.16129	0.37669										
16	0.12976	0.21113	0.39486											
8	0.29097	0.44318												
4	0.55993													

(e) $\mathcal{P}^{\mathcal{C}}$ for Scene: [**Veach Door**] Algorithm: [**M-MLT**] Metric: [**IW-MSE**] True GT: [**BDPT @ 524288 spp**].

ERPT	2	4	8	16	32	64	128	256	512	1024	2048	4096	8192	16384	32768	65536	131072
GT (BDPT)																	
262144	-0.00149	-0.00063	-0.00129	-0.00209	-0.00067	-0.00066	0.00038	0.00114	0.00094	0.00035	0.00067	-0.00053	0.00092	-0.00083	0.00216	-0.00006	-0.00009
131072	-0.00045	0.00009	-0.00035	0.00071	0.00122	-0.00040	0.00110	0.00047	0.00179	0.00138	0.00141	0.00039	0.00165	-0.00087	-0.00054	0.00207	
65536	-0.00301	-0.00161	-0.00161	-0.00069	-0.00151	-0.00083	-0.00205	0.00015	0.00127	-0.00048	-0.00045	0.00009	-0.00025	-0.00106	-0.00003		
32768	-0.00542	-0.00548	-0.00216	-0.00522	-0.00323	-0.00309	-0.00110	-0.00302	-0.00190	-0.00034	-0.00159	-0.00287	-0.00289	-0.00277			
16384	-0.01564	-0.01135	-0.01135	-0.00797	-0.00614	-0.00667	-0.00790	-0.00533	-0.01258	-0.00506	-0.00729	-0.00452	-0.01146				
8192	-0.01838	-0.02369	-0.01773	-0.01151	-0.01188	-0.01027	-0.01508	-0.00148	-0.01639	-0.00230	-0.01232	-0.01003					
4096	-0.01019	-0.00419	-0.00626	0.00022	-0.00089	0.00400	0.00171	0.01327	-0.00372	0.01188	0.00292						
2048	0.00084	-0.00498	0.00327	0.02516	0.02299	0.02901	0.01699	0.02873	0.01530	0.02956							
1024	0.03403	0.04107	0.05286	0.08200	0.06328	0.10718	0.05967	0.09404	0.05446								
512	0.12640	0.12307	0.19045	0.23361	0.23044	0.25956	0.22700	0.25660									
256	0.29711	0.28074	0.36983	0.48097	0.48516	0.53507	0.47552										
128	0.56370	0.61614	0.75470	0.91333	0.91561	0.92317											
64	1.01696	1.04366	1.25551	1.43473	1.43799												
32	1.51144	1.57522	1.83077	2.01948													
16	2.09879	2.17393	2.41621														
8	2.79791	2.89675															
4	3.45779																

(f) $\mathcal{P}^{\mathcal{C}}$ for Scene: [**Veach Door**] Algorithm: [**ERPT**] Metric: [**IW-MSE**] True GT: [**BDPT @ 524288 spp**].

M-ERPT	2	4	8	16	32	64	128	256	512	1024	2048	4096	8192	16384	32768	65536	131072
GT (BDPT)																	
262144	-0.00254	-0.00065	-0.00018	-0.00123	-0.00019	-0.00034	-0.00074	0.00026	0.00017	0.00080	-0.00103	-0.00005	-0.00014	-0.00158	0.00015	0.00090	0.00007
131072	-0.00134	-0.00138	0.00049	0.00068	0.00073	0.00140	0.00059	-0.00001	0.00079	0.00171	0.00138	0.00191	0.00286	0.00051	0.00125	0.00183	
65536	-0.00245	-0.00125	-0.00195	0.00024	0.00221	-0.00041	0.00043	0.00314	-0.00019	0.00038	-0.00061	0.00137	0.00309	-0.00111	0.00146		
32768	-0.00843	-0.00306	-0.00348	-0.00320	0.00027	-0.00461	0.00037	-0.00401	-0.00088	0.00105	-0.00086	0.00048	-0.00252	-0.00165			
16384	-0.01421	-0.00978	-0.00673	-0.00267	-0.00996	-0.00660	-0.00643	-0.00881	-0.01297	-0.00353	-0.00791	-0.00267	-0.00592				
8192	-0.01984	-0.01344	-0.01149	-0.00891	-0.01000	-0.01634	-0.00784	-0.00859	-0.00836	-0.00927	-0.00747	-0.01010					
4096	-0.01418	-0.00591	0.00700	-0.00265	0.00484	-0.00232	0.01388	-0.00158	0.00321	0.00471	-0.00147						
2048	-0.00718	0.01423	0.02137	0.00932	0.02211	0.01507	0.03054	0.01400	0.01174	0.01459							
1024	0.02312	0.07276	0.08365	0.05214	0.07223	0.07255	0.09580	0.08099	0.07086								
512	0.10017	0.15568	0.18180	0.14783	0.17075	0.15933	0.24887	0.18768									
256	0.27959	0.37306	0.40364	0.34993	0.35949	0.39971	0.51682										
128	0.52489	0.67420	0.76995	0.72601	0.78957	0.78510											
64	0.93315	1.16660	1.24627	1.23691	1.33268												
32	1.44696	1.72417	1.82283	1.84901													
16	2.00305	2.30635	2.44693														
8	2.68635	3.02544															
4	3.34562																

(g) $\mathcal{P}^{\mathcal{C}}$ for Scene: [**Veach Door**] Algorithm: [**M-ERPT**] Metric: [**IW-MSE**] True GT: [**BDPT @ 524288 spp**].

Table 19

5.5 Sponza

PT	2	4	8	16	32	64	128	256	512	1024	2048	4096	8192
GT (PT)													
16384	0.00070	0.00049	-0.00015	0.00035	0.00019	0.00186	0.00118	0.01642	0.01058	0.02687	0.06279	0.11880	0.15860
8192	0.00021	0.00095	0.00167	0.00074	0.00453	0.00525	0.01074	0.02139	0.04808	0.08759	0.14080	0.29927	
4096	0.00149	0.00337	0.00326	0.00582	0.01040	0.01480	0.02447	0.05084	0.09197	0.18977	0.33750		
2048	0.00233	0.00216	0.00314	0.00596	0.01636	0.02013	0.03896	0.09478	0.17143	0.34484			
1024	0.00430	0.00616	0.00865	0.01192	0.02012	0.04804	0.10067	0.18050	0.36056				
512	0.00649	0.01274	0.01484	0.02703	0.04510	0.08365	0.18305	0.33546					
256	0.00741	0.01359	0.02128	0.04204	0.08083	0.15631	0.29598						
128	0.01404	0.01986	0.03522	0.06753	0.14511	0.28520							
64	0.02134	0.03667	0.06381	0.13581	0.22975								
32	0.03736	0.06749	0.10507	0.21298									
16	0.07404	0.11657	0.18057										
8	0.11355	0.16858											
4	0.16489												

(a) $\mathcal{P}^{\mathcal{C}}$ for Scene: [**Sponza**] Algorithm: [**PT**] Metric: [**IW-MSE**] True GT: [**PT @ 32768 spp**].

BDPT	2	4	8	16	32	64	128	256	512	1024	2048	4096	8192
GT (PT)													
16384	-0.00004	-0.00007	0.00019	0.00180	-0.00066	0.00131	-0.00020	0.00756	0.00875	0.03105	0.03304	0.07601	0.12172
8192	0.00033	-0.00001	0.00167	0.00118	0.00141	0.00545	0.00725	0.01880	0.02370	0.07603	0.12450	0.20458	
4096	-0.00010	0.00265	0.00284	0.00637	0.00913	0.00877	0.01759	0.03171	0.06797	0.15917	0.27357		
2048	0.00119	0.00281	0.00682	0.00909	0.01280	0.01844	0.03629	0.06875	0.15513	0.29105			
1024	0.00155	0.00403	0.00955	0.01044	0.01749	0.03268	0.06628	0.14722	0.28892				
512	0.00191	0.00695	0.01347	0.02695	0.03424	0.07247	0.11890	0.27649					
256	0.00156	0.01074	0.02364	0.02619	0.05506	0.11643	0.24341						
128	0.00868	0.01918	0.03573	0.05233	0.09836	0.22692							
64	0.00887	0.03051	0.05939	0.10024	0.17625								
32	0.02398	0.04703	0.09303	0.16879									
16	0.05781	0.09201	0.16561										
8	0.08893	0.14052											
4	0.12380												

(b) $\mathcal{P}^{\mathcal{C}}$ for Scene: [**Sponza**] Algorithm: [**BDPT**] Metric: [**IW-MSE**] True GT: [**PT @ 32768 spp**].

PSSMLT	2	4	8	16	32	64	128	256	512	1024	2048	4096	8192
GT (PT)													
16384	0.00014	0.00056	0.00069	0.00075	-0.00002	-0.00067	-0.00002	0.00078	0.00339	0.00456	0.00505	0.01363	0.01116
8192	0.00056	0.00093	0.00121	0.00255	0.00133	0.00192	0.00671	0.01106	0.01575	0.01589	0.02468	0.02824	
4096	0.00070	0.00171	0.00202	0.00322	0.00500	0.00716	0.01338	0.01852	0.02913	0.03511	0.05275		
2048	0.00099	0.00269	0.00329	0.00417	0.00752	0.01063	0.02278	0.04290	0.07140	0.10694			
1024	0.00078	0.00275	0.00436	0.00903	0.01490	0.02333	0.05344	0.08886	0.14889				
512	0.00422	0.00886	0.01231	0.01780	0.03165	0.05253	0.10489	0.18708					
256	0.00530	0.01015	0.01758	0.03110	0.06156	0.09963	0.19009						
128	0.01491	0.02376	0.03484	0.05504	0.10621	0.18486							
64	0.01876	0.03454	0.06460	0.10710	0.19430								
32	0.03465	0.06252	0.10029	0.17585									
16	0.06669	0.12087	0.20720										
8	0.12658	0.18852											
4	0.20893												

(c) $\mathcal{P}^{\mathcal{C}}$ for Scene: [**Sponza**] Algorithm: [**PSSMLT**] Metric: [**IW-MSE**] True GT: [**PT @ 32768 spp**].

MLT	2	4	8	16	32	64	128	256	512	1024	2048	4096	8192
GT (PT)													
16384	0.00009	0.00001	-0.00052	-0.00100	0.00138	-0.00139	0.00267	0.00726	0.00253	0.01205	0.01113	0.01717	0.01124
8192	-0.00052	-0.00072	-0.00000	-0.00130	0.00297	0.00158	0.00898	0.00955	0.02095	0.01972	0.02467	0.02512	
4096	0.00103	0.00213	0.00449	0.00212	0.00878	0.00788	0.02050	0.02859	0.03907	0.05216	0.04728		
2048	0.00120	0.00288	0.00361	0.00736	0.01654	0.02695	0.05639	0.07561	0.11435	0.13013			
1024	0.00184	0.00535	0.00452	0.01245	0.03478	0.07028	0.10540	0.14316	0.21543				
512	0.00480	0.00777	0.01450	0.03139	0.07342	0.11047	0.20830	0.29357					
256	0.00750	0.01897	0.03266	0.08157	0.14647	0.24393	0.37115						
128	0.00950	0.02697	0.05140	0.14112	0.25537	0.43244							
64	0.01681	0.05814	0.12471	0.26934	0.44415								
32	0.04353	0.10306	0.22661	0.48568									
16	0.06864	0.20543	0.41661										
8	0.15811	0.40866											
4	0.34676												

(d) $\mathcal{P}^{\mathcal{C}}$ for Scene: [**Sponza**] Algorithm: [**MLT**] Metric: [**IW-MSE**] True GT: [**PT @ 32768 spp**].

M-MLT	2	4	8	16	32	64	128	256	512	1024	2048	4096	8192
GT (PT)													
16384	0.00038	0.00060	0.00095	-0.00091	-0.00006	0.00039	0.00721	0.00367	0.00160	0.01081	0.01154	0.00776	0.00665
8192	-0.00008	0.00061	0.00157	0.00051	0.00065	0.00430	0.01463	0.01455	0.02345	0.02255	0.02543	0.01737	
4096	0.00131	0.00175	0.00100	0.00126	0.00626	0.01559	0.02271	0.03750	0.05810	0.04329	0.06079		
2048	0.00007	0.00204	0.00480	0.00764	0.01474	0.02516	0.05762	0.08430	0.10303	0.11773			
1024	0.00393	0.00535	0.01099	0.01619	0.02650	0.06169	0.10433	0.15595	0.20341				
512	0.00498	0.01021	0.01438	0.03211	0.06682	0.10920	0.21488	0.31442					
256	0.01114	0.01393	0.03488	0.06691	0.12893	0.22353	0.40799						
128	0.01924	0.02751	0.06157	0.12699	0.23638	0.41041							
64	0.03991	0.06213	0.13271	0.23217	0.45715								
32	0.05904	0.10712	0.22702	0.42616									
16	0.12527	0.21790	0.44426										
8	0.24043	0.38294											
4	0.42344												

(e) $\mathcal{P}^{\mathcal{C}}$ for Scene: [**Sponza**] Algorithm: [**M-MLT**] Metric: [**IW-MSE**] True GT: [**PT @ 32768 spp**].

ERPT	2	4	8	16	32	64	128	256	512	1024	2048	4096	8192
GT (PT)													
16384	0.00457	0.00130	0.00064	-0.00117	0.00505	0.00636	0.00077	0.00324	0.00196	0.00013	0.00507	0.00132	0.00341
8192	0.00912	0.01103	0.00792	0.00943	0.01998	0.02127	0.00592	0.00878	0.01292	0.01006	0.01717	0.01625	
4096	0.01785	0.01995	0.02409	0.02615	0.02974	0.03308	0.02142	0.02785	0.02815	0.02751	0.02979		
2048	0.04953	0.05817	0.05715	0.05961	0.06688	0.06714	0.06207	0.05991	0.07127	0.06189			
1024	0.11090	0.10935	0.11449	0.12446	0.13334	0.14099	0.12548	0.12042	0.12818				
512	0.21201	0.22638	0.23929	0.26281	0.25104	0.27527	0.24669	0.24577					
256	0.38247	0.40670	0.43962	0.46031	0.47323	0.49088	0.44787						
128	0.70603	0.72910	0.76727	0.85797	0.84059	0.85447							
64	1.13560	1.19580	1.22389	1.33170	1.29911								
32	1.71627	1.75676	1.80850	1.91897									
16	2.35183	2.43338	2.49071										
8	3.03492	3.12140											
4	3.66778												

(f) $\mathcal{P}^{\mathcal{C}}$ for Scene: [**Sponza**] Algorithm: [**ERPT**] Metric: [**IW-MSE**] True GT: [**PT @ 32768 spp**].

M-ERPT	2	4	8	16	32	64	128	256	512	1024	2048	4096	8192
GT (PT)													
16384	0.00526	0.00336	0.00023	0.00541	0.00482	0.00407	0.00331	0.00483	-0.00086	0.00068	0.00586	0.00394	0.00028
8192	0.00828	0.01208	0.01056	0.01684	0.01204	0.01566	0.01331	0.01561	0.00635	0.00663	0.00761	0.01185	
4096	0.02498	0.02992	0.03193	0.03185	0.03204	0.03181	0.03087	0.02044	0.02381	0.02308	0.03073		
2048	0.05011	0.05104	0.06095	0.06151	0.06452	0.06286	0.06144	0.06657	0.06672	0.06068			
1024	0.11814	0.09624	0.12707	0.12320	0.13225	0.12429	0.13643	0.15371	0.12588				
512	0.21180	0.21536	0.23052	0.24896	0.25073	0.25983	0.24189	0.26441					
256	0.38003	0.42685	0.43769	0.43204	0.47998	0.46754	0.44545						
128	0.68783	0.75667	0.80270	0.78101	0.82543	0.83062							
64	1.12260	1.18521	1.24915	1.27324	1.28956								
32	1.65879	1.78939	1.84325	1.86274									
16	2.31166	2.42978	2.51295										
8	2.99864	3.12341											
4	3.62657												

(g) $\mathcal{P}^{\mathcal{C}}$ for Scene: [**Sponza**] Algorithm: [**M-ERPT**] Metric: [**IW-MSE**] True GT: [**PT @ 32768 spp**].

Table 20

6 MAE

6.1 Cornell Box

PT	2	4	8	16	32	64	128	256	512	1024	2048	4096	8192
GT (PT)													
16384	0.00003	0.00003	0.00004	-0.00008	0.00045	0.00104	0.00210	0.00308	0.00744	0.01603	0.02983	0.04987	0.09516
8192	0.00011	0.00045	0.00029	0.00070	0.00114	0.00251	0.00553	0.00996	0.02102	0.04242	0.08041	0.13929	
4096	0.00019	0.00051	0.00073	0.00157	0.00333	0.00542	0.01389	0.02631	0.05032	0.09495	0.16635		
2048	0.00083	0.00095	0.00201	0.00357	0.00678	0.01427	0.02755	0.05509	0.10260	0.18486			
1024	0.00102	0.00155	0.00383	0.00756	0.01452	0.02929	0.05534	0.10277	0.19066				
512	0.00167	0.00408	0.00727	0.01545	0.03086	0.05690	0.10642	0.18777					
256	0.00381	0.00796	0.01421	0.02932	0.05688	0.11089	0.20051						
128	0.00718	0.01564	0.03125	0.05962	0.11084	0.20183							
64	0.01525	0.03008	0.06006	0.11189	0.20056								
32	0.02998	0.05811	0.11212	0.20082									
16	0.05768	0.11107	0.20112										
8	0.10396	0.19865											
4	0.18603												

(a) $\mathcal{P}^{\mathcal{C}}$ for Scene: [**Cornell Box**] Algorithm: [**PT**] Metric: [**MAE**] True GT: [**PT @ 32768 spp**].

BDPT	2	4	8	16	32	64	128	256	512	1024	2048	4096	8192
GT (PT)													
16384	0.00010	0.00011	-0.00001	0.00085	0.00161	0.00284	0.00597	0.01098	0.02431	0.03979	0.07242	0.11215	0.16951
8192	0.00042	0.00031	0.00095	0.00242	0.00428	0.00837	0.01738	0.03323	0.06089	0.11033	0.18556	0.27932	
4096	0.00039	0.00143	0.00253	0.00467	0.01003	0.02055	0.03850	0.07537	0.13198	0.22871	0.35324		
2048	0.00105	0.00240	0.00558	0.01012	0.02127	0.04249	0.07949	0.14345	0.24651	0.39029			
1024	0.00299	0.00578	0.01109	0.02154	0.04115	0.07757	0.14608	0.25707	0.40891				
512	0.00513	0.01109	0.02258	0.04365	0.08230	0.15196	0.25980	0.42103					
256	0.01053	0.02091	0.04373	0.08462	0.15454	0.26478	0.43365						
128	0.02092	0.04330	0.08398	0.15178	0.27425	0.43829							
64	0.03958	0.08035	0.15497	0.26739	0.43801								
32	0.07768	0.14871	0.26591	0.43275									
16	0.14152	0.25727	0.42696										
8	0.24363	0.41359											
4	0.39417												

(b) $\mathcal{P}^{\mathcal{C}}$ for Scene: [**Cornell Box**] Algorithm: [**BDPT**] Metric: [**MAE**] True GT: [**PT @ 32768 spp**].

PSSMLT	2	4	8	16	32	64	128	256	512	1024	2048	4096	8192
GT (PT)													
16384	0.00015	0.00007	0.00012	0.00059	0.00137	0.00344	0.00544	0.00780	0.01334	0.02241	0.03079	0.04166	0.04801
8192	0.00041	0.00049	0.00057	0.00154	0.00419	0.00816	0.01285	0.02360	0.03729	0.05791	0.07897	0.10350	
4096	0.00057	0.00097	0.00250	0.00469	0.00936	0.01768	0.03196	0.05242	0.08358	0.12325	0.16461		
2048	0.00131	0.00274	0.00519	0.01045	0.02082	0.03668	0.06408	0.10536	0.16007	0.22701			
1024	0.00260	0.00548	0.01146	0.02091	0.03902	0.07246	0.12260	0.19316	0.27837				
512	0.00561	0.01102	0.02199	0.04262	0.07922	0.13674	0.22243	0.33290					
256	0.01218	0.02425	0.04586	0.08444	0.15003	0.24706	0.37610						
128	0.02300	0.04682	0.08710	0.15842	0.26590	0.41091							
64	0.04517	0.08638	0.15821	0.27249	0.43093								
32	0.08412	0.15980	0.27310	0.43831									
16	0.15206	0.27241	0.44244										
8	0.26063	0.43508											
4	0.42224												

(c) $\mathcal{P}^{\mathcal{C}}$ for Scene: [**Cornell Box**] Algorithm: [**PSSMLT**] Metric: [**MAE**] True GT: [**PT @ 32768 spp**].

MLT	2	4	8	16	32	64	128	256	512	1024	2048	4096	8192
GT (PT)													
16384	-0.00004	-0.00004	-0.00001	-0.00009	0.00019	0.00065	0.00090	0.00199	0.00340	0.00629	0.00898	0.01627	0.02524
8192	0.00006	0.00015	0.00018	0.00023	0.00061	0.00159	0.00327	0.00641	0.01078	0.01860	0.03066	0.04867	
4096	0.00008	0.00020	0.00027	0.00084	0.00207	0.00478	0.00876	0.01513	0.02688	0.04322	0.07013		
2048	0.00026	0.00033	0.00058	0.00128	0.00376	0.00784	0.01583	0.03085	0.05295	0.08689			
1024	0.00053	0.00106	0.00204	0.00459	0.00934	0.01736	0.03352	0.06067	0.10222				
512	0.00138	0.00244	0.00493	0.00976	0.01841	0.03550	0.06651	0.11719					
256	0.00209	0.00489	0.01002	0.01952	0.03817	0.06990	0.12581						
128	0.00385	0.00803	0.01662	0.03406	0.06806	0.12478							
64	0.00690	0.01593	0.03646	0.06892	0.12827								
32	0.01521	0.03203	0.06736	0.12584									
16	0.03024	0.06062	0.12509										
8	0.05256	0.11073											
4	0.10496												

(d) $\mathcal{P}^{\mathcal{C}}$ for Scene: [**Cornell Box**] Algorithm: [**MLT**] Metric: [**MAE**] True GT: [**PT @ 32768 spp**].

M-MLT	2	4	8	16	32	64	128	256	512	1024	2048	4096	8192
GT (PT)													
16384	0.00003	-0.00003	-0.00012	-0.00004	-0.00031	0.00049	0.00078	0.00212	0.00415	0.00730	0.01101	0.01833	0.02686
8192	0.00008	0.00009	0.00023	0.00058	0.00095	0.00183	0.00327	0.00675	0.01346	0.02090	0.03430	0.04989	
4096	0.00005	0.00023	0.00047	0.00105	0.00202	0.00470	0.00807	0.01569	0.02797	0.04696	0.07346		
2048	0.00039	0.00059	0.00123	0.00244	0.00451	0.00872	0.01660	0.03216	0.05567	0.09196			
1024	0.00058	0.00078	0.00220	0.00456	0.00966	0.01905	0.03574	0.06458	0.10682				
512	0.00088	0.00248	0.00482	0.00987	0.01823	0.03659	0.06714	0.11807					
256	0.00228	0.00524	0.00973	0.01901	0.03810	0.07162	0.12916						
128	0.00366	0.00813	0.01863	0.03729	0.07198	0.13486							
64	0.00715	0.01807	0.03680	0.07015	0.12947								
32	0.01707	0.03517	0.07120	0.13285									
16	0.03207	0.06832	0.13025										
8	0.06176	0.12216											
4	0.11108												

(e) $\mathcal{P}^{\mathcal{C}}$ for Scene: [**Cornell Box**] Algorithm: [**M-MLT**] Metric: [**MAE**] True GT: [**PT @ 32768 spp**].

ERPT	2	4	8	16	32	64	128	256	512	1024	2048	4096	8192
GT (PT)													
16384	0.00114	0.00181	0.00086	0.00108	0.00058	0.00164	0.00161	0.00137	0.00162	0.00126	0.00067	0.00115	0.00148
8192	0.00424	0.00408	0.00449	0.00324	0.00339	0.00416	0.00330	0.00397	0.00361	0.00417	0.00389	0.00409	
4096	0.00851	0.00995	0.00853	0.00756	0.00847	0.01039	0.00901	0.00825	0.00928	0.00925	0.00919		
2048	0.01856	0.01978	0.01961	0.01810	0.01776	0.02007	0.01926	0.01774	0.01783	0.01930			
1024	0.03877	0.03580	0.03676	0.03591	0.03666	0.04033	0.03866	0.03699	0.03914				
512	0.07342	0.07312	0.07423	0.07274	0.07353	0.07737	0.07360	0.07479					
256	0.13930	0.14169	0.14043	0.13769	0.13833	0.14413	0.13890						
128	0.24646	0.24720	0.24817	0.24436	0.24196	0.25119							
64	0.40299	0.40509	0.40470	0.39902	0.39561								
32	0.61480	0.61533	0.61768	0.61169									
16	0.86713	0.87413	0.87304										
8	1.15589	1.16114											
4	1.45829												

(f) $\mathcal{P}^{\mathcal{C}}$ for Scene: [**Cornell Box**] Algorithm: [**ERPT**] Metric: [**MAE**] True GT: [**PT @ 32768 spp**].

M-ERPT	2	4	8	16	32	64	128	256	512	1024	2048	4096	8192
GT (PT)													
16384	0.00119	0.00098	0.00130	0.00136	0.00127	0.00201	0.00179	0.00170	0.00134	0.00107	0.00060	0.00146	0.00140
8192	0.00407	0.00364	0.00342	0.00437	0.00362	0.00349	0.00346	0.00404	0.00312	0.00329	0.00344	0.00386	
4096	0.00820	0.00796	0.00830	0.00853	0.00830	0.00846	0.00813	0.00789	0.00809	0.00797	0.00894		
2048	0.01840	0.01785	0.01733	0.01857	0.01905	0.01806	0.01646	0.01699	0.01599	0.01785			
1024	0.03425	0.03469	0.03620	0.03603	0.03530	0.03589	0.03401	0.03706	0.03410				
512	0.06813	0.06934	0.06812	0.07085	0.07150	0.06863	0.06907	0.06740					
256	0.13118	0.13277	0.13228	0.13115	0.13387	0.13151	0.13348						
128	0.23195	0.23489	0.23311	0.23616	0.23721	0.23340							
64	0.38947	0.38883	0.38437	0.38564	0.39044								
32	0.59138	0.59410	0.58808	0.59403									
16	0.84132	0.84672	0.83832										
8	1.12636	1.13192											
4	1.42942												

(g) $\mathcal{P}^{\mathcal{C}}$ for Scene: [**Cornell Box**] Algorithm: [**M-ERPT**] Metric: [**MAE**] True GT: [**PT @ 32768 spp**].

Table 21

6.2 Torus

PT	2	4	8	16	32	64	128	256	512	1024	2048	4096	8192	16384	32768	65536	131072
GT (BDPT)																	
262144	-0.00102	-0.00164	-0.00219	-0.00271	-0.00307	-0.00303	-0.00308	-0.00306	-0.00357	-0.00374	-0.00200	-0.00048	0.00226	0.00728	0.01363	0.04047	0.07777
131072	-0.00131	-0.00178	-0.00234	-0.00295	-0.00336	-0.00252	-0.00258	-0.00228	-0.00142	-0.00022	0.00233	0.00971	0.01791	0.03764	0.07010	0.12584	
65536	-0.00264	-0.00299	-0.00516	-0.00672	-0.00729	-0.00735	-0.00708	-0.00728	-0.00582	-0.00328	0.00293	0.01298	0.03435	0.07926	0.14517		
32768	-0.00128	-0.00255	-0.00369	-0.00461	-0.00487	-0.00485	-0.00501	-0.00310	-0.00009	0.00547	0.01451	0.03684	0.08015	0.16418			
16384	0.00069	0.00068	-0.00025	-0.00095	-0.00222	0.00018	0.00088	0.00415	0.00628	0.01545	0.02991	0.07380	0.14758				
8192	0.00221	0.00164	-0.00261	-0.00378	-0.00387	-0.00320	-0.00166	0.00444	0.00650	0.02447	0.05350	0.12583					
4096	0.00810	0.00842	0.00242	0.00075	-0.00434	-0.00320	-0.00053	0.00309	0.01148	0.03947	0.09850						
2048	0.01190	0.01035	0.00129	-0.00553	-0.01300	-0.01718	-0.01381	-0.00708	0.01314	0.06345							
1024	0.00415	-0.00144	-0.02012	-0.03552	-0.05475	-0.06492	-0.06096	-0.04795	-0.00172								
512	-0.01073	-0.02773	-0.06083	-0.09194	-0.11860	-0.13483	-0.13012	-0.09745									
256	-0.04007	-0.07249	-0.12521	-0.17529	-0.21645	-0.23932	-0.21113										
128	-0.08707	-0.13730	-0.21503	-0.27552	-0.31602	-0.34026											
64	-0.11643	-0.17150	-0.26229	-0.32498	-0.35426												
32	-0.13556	-0.19298	-0.28110	-0.32144													
16	-0.14211	-0.18663	-0.25332														
8	-0.11035	-0.12232															
4	-0.03711																

(a) $\mathcal{P}^{\mathcal{C}}$ for Scene: [**Torus**] Algorithm: [**PT**] Metric: [**MAE**] True GT: [**BDPT @ 524288 spp**].

BDPT	2	4	8	16	32	64	128	256	512	1024	2048	4096	8192	16384	32768	65536	131072
GT (BDPT)																	
262144	-0.00135	-0.00166	-0.00219	-0.00270	-0.00252	-0.00348	-0.00298	-0.00375	-0.00325	-0.00301	-0.00150	-0.00273	0.00211	0.00593	0.01704	0.03766	0.08043
131072	-0.00136	-0.00202	-0.00229	-0.00308	-0.00320	-0.00297	-0.00228	-0.00287	-0.00161	0.00032	0.00296	0.00652	0.01751	0.03331	0.06282	0.12365	
65536	-0.00282	-0.00365	-0.00534	-0.00648	-0.00702	-0.00839	-0.00762	-0.00699	-0.00584	-0.00313	0.00263	0.00855	0.02879	0.07069	0.13918		
32768	-0.00166	-0.00186	-0.00328	-0.00445	-0.00616	-0.00520	-0.00463	-0.00260	-0.00378	0.00653	0.01554	0.03322	0.07043	0.14665			
16384	-0.00013	0.00022	-0.00088	-0.00167	-0.00264	-0.00158	-0.00044	0.00223	0.00709	0.01800	0.03466	0.06413	0.12698				
8192	0.00143	0.00049	-0.00096	-0.00428	-0.00701	-0.00513	-0.00181	-0.00088	0.00947	0.02316	0.04818	0.10965					
4096	0.00660	0.00757	0.00287	0.00154	-0.00359	-0.00389	-0.00432	0.00336	0.01006	0.03531	0.09104						
2048	0.01189	0.01048	0.00091	-0.00581	-0.01210	-0.01724	-0.01903	-0.01203	0.00750	0.05836							
1024	0.00697	-0.00181	-0.01569	-0.03329	-0.04904	-0.06547	-0.06198	-0.05141	-0.00426								
512	-0.00769	-0.02515	-0.05418	-0.08593	-0.11372	-0.13415	-0.13467	-0.10443									
256	-0.03721	-0.06716	-0.11457	-0.16487	-0.20468	-0.22589	-0.22403										
128	-0.08006	-0.12961	-0.19580	-0.26658	-0.31462	-0.33095											
64	-0.10850	-0.16741	-0.24181	-0.30710	-0.34214												
32	-0.12793	-0.19094	-0.25651	-0.31257													
16	-0.13559	-0.18368	-0.23204														
8	-0.10814	-0.12806															
4	-0.03914																

(b) $\mathcal{P}^{\mathcal{C}}$ for Scene: [**Torus**] Algorithm: [**BDPT**] Metric: [**MAE**] True GT: [**BDPT @ 524288 spp**].

PSSMLT	2	4	8	16	32	64	128	256	512	1024	2048	4096	8192	16384
GT (BDPT)														
262144	-0.00237	-0.00305	-0.00333	-0.00367	-0.00306	-0.00308	-0.00225	-0.00194	-0.00053	0.00112	0.00178	0.00438	0.01394	0.00432
131072	-0.00319	-0.00356	-0.00391	-0.00399	-0.00244	-0.00155	0.00089	0.00276	0.00471	0.00831	0.01429	0.02118	0.04690	0.02691
65536	-0.00693	-0.00794	-0.00738	-0.00839	-0.00628	-0.00424	-0.00073	0.00427	0.01207	0.02037	0.03075	0.04759	0.09865	0.06465
32768	-0.00901	-0.00930	-0.00860	-0.00909	-0.00433	0.00059	0.00908	0.02003	0.03597	0.05453	0.07676	0.10618	0.18690	0.13795
16384	-0.00935	-0.00754	-0.00547	-0.00408	0.00406	0.01338	0.03169	0.05038	0.07528	0.10555	0.14274	0.19008	0.30304	
8192	-0.01475	-0.01253	-0.00850	-0.00813	0.00668	0.02663	0.05513	0.08668	0.13160	0.17506	0.23792	0.30727		
4096	-0.01516	-0.01020	-0.00534	-0.00473	0.01684	0.04411	0.08948	0.14158	0.20635	0.27232	0.35351			
2048	-0.01468	-0.00735	0.00093	0.00446	0.03630	0.07933	0.13848	0.21561	0.30118	0.39041				
1024	-0.03513	-0.03078	-0.02241	-0.02010	0.02391	0.08637	0.17669	0.27893	0.39173					
512	-0.06534	-0.06621	-0.06042	-0.05833	0.00987	0.09470	0.20603	0.33263						
256	-0.11588	-0.12220	-0.11256	-0.10664	-0.01191	0.10309	0.24061							
128	-0.18612	-0.19404	-0.18280	-0.16876	-0.03823	0.10247								
64	-0.22095	-0.21636	-0.19721	-0.17718	-0.01527									
32	-0.23987	-0.21432	-0.18124	-0.14875										
16	-0.22803	-0.17205	-0.12074											
8	-0.16376	-0.07315												
4	-0.04547													

(c) $\mathcal{P}^{\mathcal{C}}$ for Scene: [**Torus**] Algorithm: [**PSSMLT**] Metric: [**MAE**] True GT: [**BDPT @ 524288 spp**].

MLT	2	4	8	16	32	64	128	256	512	1024	2048	4096	8192	16384
GT (BDPT)														
262144	-0.00089	-0.00142	-0.00170	-0.00193	-0.00033	-0.00087	0.00054	0.00179	0.00222	0.00260	0.00379	0.00316	0.00368	0.00116
131072	-0.00149	-0.00174	-0.00082	-0.00071	0.00386	0.00367	0.00475	0.00845	0.01044	0.01036	0.01473	0.01349	0.01434	0.01773
65536	-0.00334	-0.00404	-0.00395	-0.00301	0.00606	0.00719	0.01009	0.01846	0.02241	0.02191	0.02944	0.02827	0.03093	0.03802
32768	-0.00410	-0.00534	-0.00320	0.00059	0.01699	0.01755	0.02555	0.04203	0.04705	0.04792	0.06110	0.05797	0.06559	0.07799
16384	-0.00584	-0.00737	-0.00201	0.00285	0.03679	0.03777	0.05131	0.07432	0.08588	0.09071	0.10890	0.10650	0.11970	
8192	-0.00917	-0.00982	0.00545	0.01246	0.06389	0.06918	0.09406	0.13370	0.14838	0.16013	0.18305	0.18110		
4096	-0.01297	-0.01205	0.01541	0.03197	0.10702	0.11590	0.14962	0.21000	0.22596	0.24607	0.27289			
2048	-0.01786	-0.01226	0.02971	0.05442	0.16397	0.17325	0.21605	0.30085	0.32153	0.35124				
1024	-0.02523	-0.01524	0.03319	0.07096	0.22206	0.23516	0.29003	0.40410	0.42571					
512	-0.03762	-0.02872	0.02916	0.07632	0.27415	0.28657	0.35298	0.49918						
256	-0.05574	-0.04506	0.01761	0.07895	0.31550	0.33717	0.41237							
128	-0.08104	-0.07499	-0.00513	0.07440	0.34295	0.36940								
64	-0.09719	-0.09176	-0.00263	0.09118	0.37975									
32	-0.11404	-0.11364	0.01147	0.11455										
16	-0.13144	-0.13108	0.04752											
8	-0.13628	-0.13390												
4	-0.14772													

(d) $\mathcal{P}^{\mathcal{C}}$ for Scene: [**Torus**] Algorithm: [**MLT**] Metric: [**MAE**] True GT: [**BDPT @ 524288 spp**].

M-MLT	2	4	8	16	32	64	128	256	512	1024	2048	4096	8192	16384
GT (BDPT)														
262144	-0.00063	-0.00107	-0.00140	-0.00109	-0.00183	0.00052	0.00315	0.00565	0.00576	0.00884	0.00822	0.00913	0.01047	0.00650
131072	-0.00111	-0.00162	-0.00055	-0.00059	-0.00072	0.00507	0.00875	0.01239	0.01681	0.02415	0.02340	0.02474	0.02762	0.03013
65536	-0.00273	-0.00353	-0.00200	-0.00009	-0.00070	0.01372	0.02068	0.03387	0.03607	0.05235	0.05143	0.05167	0.05950	0.06321
32768	-0.00348	-0.00442	0.00096	0.00370	0.00679	0.02959	0.04293	0.06553	0.07068	0.09650	0.09639	0.09646	0.10988	0.12577
16384	-0.00505	-0.00492	0.00520	0.00767	0.01543	0.05297	0.07522	0.11300	0.11993	0.16131	0.15851	0.15826	0.18007	
8192	-0.00754	-0.00440	0.01301	0.02225	0.03481	0.09802	0.13290	0.19046	0.20648	0.25919	0.26232	0.26351		
4096	-0.00947	-0.00036	0.03428	0.04276	0.06309	0.15768	0.20771	0.28053	0.29878	0.37033	0.37327			
2048	-0.01288	0.00317	0.05825	0.07713	0.10991	0.23141	0.29652	0.38629	0.41633	0.49812				
1024	-0.01657	0.00627	0.08323	0.10882	0.14748	0.32000	0.40157	0.50620	0.54310					
512	-0.02374	-0.00466	0.10592	0.13126	0.17621	0.38538	0.48841	0.60586						
256	-0.03501	-0.02463	0.11298	0.14806	0.20755	0.44777	0.56319							
128	-0.05347	-0.06212	0.11695	0.15133	0.22738	0.48829								
64	-0.06641	-0.08659	0.13777	0.17489	0.26086									
32	-0.07878	-0.10399	0.15697	0.19695										
16	-0.08857	-0.10540	0.19533											
8	-0.09481	-0.08056												
4	-0.10522													

(e) $\mathcal{P}^{\mathcal{C}}$ for Scene: [**Torus**] Algorithm: [**M-MLT**] Metric: [**MAE**] True GT: [**BDPT @ 524288 spp**].

ERPT	2	4	8	16	32	64	128	256	512	1024	2048	4096	8192	16384	32768	65536	131072
GT (BDPT)																	
262144	-0.00228	-0.00097	0.00150	0.00134	0.00649	0.00386	0.00643	0.00587	0.00774	0.00865	0.00687	0.00942	0.00886	0.00869	0.00827	0.00918	0.00938
131072	0.00087	0.00592	0.00836	0.01327	0.01397	0.01698	0.02095	0.02081	0.02431	0.02682	0.02592	0.03197	0.03040	0.02987	0.02893	0.03064	
65536	0.00054	0.00929	0.01997	0.02481	0.03635	0.03433	0.04924	0.04593	0.05392	0.05647	0.05893	0.06598	0.06493	0.06701	0.06468		
32768	0.01340	0.02767	0.04458	0.05951	0.07195	0.07745	0.09945	0.09698	0.10772	0.11498	0.11858	0.13070	0.12826	0.12457			
16384	0.03266	0.06208	0.09391	0.11082	0.12394	0.14074	0.17385	0.17405	0.18918	0.19847	0.20575	0.22272	0.22041				
8192	0.06395	0.10901	0.15837	0.18546	0.20975	0.23201	0.27948	0.28127	0.29902	0.31391	0.32515	0.34379					
4096	0.11150	0.16687	0.24246	0.28091	0.31701	0.34572	0.40129	0.40441	0.43060	0.44590	0.46053						
2048	0.15899	0.24224	0.34516	0.38968	0.43758	0.46671	0.53618	0.54086	0.57047	0.59030							
1024	0.20162	0.31094	0.43175	0.49375	0.56178	0.58147	0.66764	0.66842	0.70759								
512	0.22750	0.35595	0.51025	0.58272	0.66233	0.67785	0.76885	0.77043									
256	0.24938	0.40298	0.57728	0.64534	0.74398	0.75221	0.85559										
128	0.25173	0.42778	0.61306	0.69518	0.80134	0.80581											
64	0.28739	0.47800	0.66560	0.74662	0.85137												
32	0.33646	0.53639	0.71306	0.79043													
16	0.41590	0.62523	0.79159														
8	0.54513	0.74778															
4	0.73306																

(f) $\mathcal{P}^{\mathcal{C}}$ for Scene: [**Torus**] Algorithm: [**ERPT**] Metric: [**MAE**] True GT: [**BDPT @ 524288 spp**].

M-ERPT	2	4	8	16	32	64	128	256	512	1024	2048	4096	8192	16384	32768	65536	131072
GT (BDPT)																	
262144	-0.00284	0.00026	-0.00117	0.00140	0.00105	0.00269	0.00709	0.00720	0.00799	0.00858	0.01047	0.00942	0.01073	0.01042	0.01007	0.00901	0.01024
131072	-0.00048	0.00279	0.00221	0.00566	0.00891	0.01226	0.01880	0.02264	0.02547	0.02531	0.02779	0.02799	0.02768	0.03053	0.02846	0.02923	
65536	-0.00301	0.00413	0.00617	0.01792	0.02220	0.02615	0.04247	0.05034	0.05488	0.05652	0.06149	0.05912	0.06096	0.06290	0.06222		
32768	0.00267	0.01542	0.01756	0.03717	0.04793	0.06449	0.08887	0.10114	0.10885	0.11462	0.12080	0.12038	0.12117	0.12008			
16384	0.01240	0.02975	0.04757	0.07485	0.09126	0.11998	0.15898	0.17484	0.18753	0.19683	0.20722	0.20552	0.20881				
8192	0.02171	0.05637	0.08633	0.13351	0.16072	0.20141	0.26062	0.28318	0.30405	0.31198	0.32481	0.31946					
4096	0.04344	0.09862	0.14433	0.21016	0.25134	0.30693	0.37921	0.40852	0.43042	0.44114	0.45834						
2048	0.06774	0.14689	0.21624	0.30264	0.35847	0.42707	0.51521	0.54597	0.57336	0.58773							
1024	0.09013	0.20303	0.28706	0.40069	0.46266	0.54054	0.64640	0.67881	0.70871								
512	0.09442	0.24905	0.33909	0.48470	0.53950	0.62726	0.74763	0.78723									
256	0.10945	0.28828	0.38521	0.55136	0.60295	0.69700	0.83099										
128	0.11766	0.30640	0.41292	0.60114	0.65006	0.74289											
64	0.15744	0.34196	0.45088	0.65059	0.69311												
32	0.20085	0.38476	0.49607	0.68989													
16	0.27866	0.45698	0.57029														
8	0.39804	0.56583															
4	0.58416																

(g) $\mathcal{P}^{\mathcal{C}}$ for Scene: [**Torus**] Algorithm: [**M-ERPT**] Metric: [**MAE**] True GT: [**BDPT @ 524288 spp**].

Table 22

6.3 Veach Bidir

PT	2	4	8	16	32	64	128	256	512	1024	2048	4096	8192	16384	32768	65536	131072
GT (BDPT)																	
262144	-0.00012	-0.00014	-0.00017	-0.00022	-0.00020	-0.00019	-0.00026	-0.00029	-0.00048	-0.00035	-0.00048	-0.00085	-0.00042	-0.00081	-0.00096	-0.00068	0.00008
131072	-0.00024	-0.00030	-0.00038	-0.00044	-0.00047	-0.00058	-0.00066	-0.00078	-0.00087	-0.00106	-0.00134	-0.00145	-0.00160	-0.00177	-0.00129	-0.00032	
65536	-0.00025	-0.00029	-0.00036	-0.00049	-0.00051	-0.00054	-0.00073	-0.00062	-0.00068	-0.00066	-0.00110	-0.00160	-0.00108	-0.00032	-0.00022		
32768	-0.00025	-0.00024	-0.00039	-0.00048	-0.00033	-0.00048	-0.00065	-0.00063	-0.00055	-0.00060	-0.00070	-0.00074	0.00015	0.00087			
16384	-0.00034	-0.00041	-0.00055	-0.00069	-0.00069	-0.00064	-0.00066	-0.00053	-0.00057	-0.00057	-0.00063	-0.00041	0.00072				
8192	-0.00036	-0.00034	-0.00051	-0.00053	-0.00043	-0.00073	-0.00070	-0.00039	-0.00034	0.00002	0.00092	0.00144					
4096	-0.00023	-0.00033	-0.00040	-0.00040	-0.00033	-0.00023	-0.00001	0.00040	0.00040	0.00083	0.00178						
2048	-0.00033	-0.00036	-0.00043	-0.00055	-0.00026	-0.00025	0.00041	0.00133	0.00146	0.00334							
1024	-0.00050	-0.00055	-0.00085	-0.00092	-0.00060	-0.00022	0.00116	0.00214	0.00282								
512	-0.00048	-0.00070	-0.00074	-0.00019	0.00021	0.00063	0.00287	0.00551									
256	-0.00048	-0.00028	-0.00027	0.00059	0.00227	0.00340	0.00818										
128	-0.00054	-0.00014	-0.00014	0.00200	0.00555	0.00825											
64	-0.00022	0.00016	0.00152	0.00480	0.01033												
32	-0.00023	0.00194	0.00532	0.01177													
16	0.00135	0.00575	0.01290														
8	0.00562	0.01518															
4	0.01185																

(a) $\mathcal{P}^{\mathcal{C}}$ for Scene: [**Veach Bidir**] Algorithm: [**PT**] Metric: [**MAE**] True GT: [**BDPT @ 524288 spp**].

BDPT	2	4	8	16	32	64	128	256	512	1024	2048	4096	8192	16384	32768	65536	131072
GT (BDPT)																	
262144	-0.00024	-0.00016	-0.00017	-0.00024	-0.00025	-0.00014	-0.00009	-0.00001	-0.00027	-0.00090	-0.00107	0.00088	-0.00575	-0.01196	-0.01696	-0.02800	-0.03121
131072	-0.00037	-0.00047	-0.00039	-0.00025	-0.00059	-0.00033	0.00008	0.00015	0.00007	0.00083	0.00160	0.00254	0.00068	-0.00372	0.00295	0.00843	
65536	-0.00048	-0.00046	-0.00041	-0.00026	-0.00022	0.00005	0.00108	0.00122	0.00246	0.00601	0.00851	0.02096	0.02469	0.05345	0.09535		
32768	-0.00035	-0.00031	-0.00015	0.00001	0.00027	0.00109	0.00284	0.00419	0.00609	0.01198	0.02406	0.05325	0.08889	0.14187			
16384	-0.00064	-0.00062	-0.00074	-0.00029	-0.00044	0.00020	0.00187	0.00553	0.01012	0.02174	0.04804	0.10123	0.15946				
8192	-0.00042	-0.00047	-0.00069	0.00033	0.00109	0.00240	0.00560	0.01409	0.02678	0.05333	0.10009	0.18496					
4096	-0.00006	0.00011	0.00071	0.00151	0.00377	0.00760	0.01495	0.02940	0.05529	0.10224	0.18924						
2048	-0.00030	-0.00019	0.00092	0.00320	0.00519	0.01231	0.02609	0.05434	0.09709	0.18815							
1024	-0.00043	0.00008	0.00123	0.00470	0.01005	0.02192	0.04752	0.10102	0.17842								
512	-0.00012	0.00102	0.00385	0.01093	0.02248	0.04762	0.09899	0.19018									
256	0.00201	0.00483	0.01178	0.02642	0.05084	0.09759	0.18619										
128	0.00501	0.01259	0.02622	0.05559	0.09979	0.18316											
64	0.01171	0.02573	0.05439	0.10548	0.18685												
32	0.02586	0.05529	0.10569	0.19331													
16	0.05392	0.10473	0.19061														
8	0.10246	0.19295															
4	0.18564																

(b) $\mathcal{P}^{\mathcal{C}}$ for Scene: [**Veach Bidir**] Algorithm: [**BDPT**] Metric: [**MAE**] True GT: [**BDPT @ 524288 spp**].

PSSMLT	2	4	8	16	32	64	128	256	512	1024	2048	4096	8192	16384
GT (BDPT)														
262144	-0.00033	-0.00032	-0.00037	-0.00031	-0.00037	-0.00041	-0.00028	-0.00015	0.00001	0.00010	0.00026	0.00061	-0.00008	-0.00036
131072	-0.00070	-0.00061	-0.00071	-0.00051	-0.00075	-0.00064	-0.00055	-0.00038	0.00032	0.00017	0.00075	0.00175	0.00281	0.00222
65536	-0.00072	-0.00071	-0.00063	-0.00056	-0.00062	-0.00046	0.00009	0.00123	0.00163	0.00242	0.00490	0.00734	0.00935	0.01357
32768	-0.00069	-0.00059	-0.00040	-0.00010	0.00014	0.00063	0.00148	0.00292	0.00499	0.00663	0.01224	0.01903	0.02781	0.03586
16384	-0.00088	-0.00092	-0.00093	-0.00070	-0.00079	-0.00045	0.00089	0.00299	0.00751	0.01181	0.02332	0.03414	0.05032	
8192	-0.00077	-0.00069	-0.00069	-0.00033	0.00007	0.00175	0.00402	0.00791	0.01633	0.02651	0.04499	0.06811		
4096	-0.00052	-0.00020	0.00036	0.00134	0.00263	0.00546	0.01073	0.01908	0.03456	0.05535	0.09001			
2048	-0.00060	-0.00032	0.00037	0.00193	0.00424	0.01009	0.01936	0.03644	0.06494	0.10091				
1024	-0.00105	-0.00044	0.00029	0.00277	0.00748	0.01543	0.03245	0.06190	0.11221					
512	-0.00054	0.00072	0.00283	0.00742	0.01812	0.03681	0.06845	0.12538						
256	0.00100	0.00424	0.01048	0.02245	0.04477	0.08008	0.13749							
128	0.00361	0.01029	0.02313	0.04655	0.08720	0.15010								
64	0.00933	0.02192	0.04630	0.09020	0.16081									
32	0.02323	0.05176	0.09588	0.17616										
16	0.04568	0.09856	0.17670											
8	0.09051	0.17711												
4	0.16643													

(c) $\mathcal{P}^{\mathcal{C}}$ for Scene: [**Veach Bidir**] Algorithm: [**PSSMLT**] Metric: [**MAE**] True GT: [**BDPT @ 524288 spp**].

MLT	2	4	8	16	32	64	128	256	512	1024	2048	4096	8192	16384
GT (BDPT)														
262144	-0.00018	-0.00023	-0.00026	-0.00034	-0.00042	-0.00054	-0.00040	-0.00036	-0.00050	-0.00020	-0.00089	-0.00056	-0.00077	-0.00061
131072	-0.00042	-0.00051	-0.00064	-0.00079	-0.00089	-0.00102	-0.00078	-0.00095	-0.00113	-0.00074	-0.00176	-0.00110	-0.00130	-0.00019
65536	-0.00046	-0.00059	-0.00073	-0.00097	-0.00108	-0.00118	-0.00070	-0.00096	-0.00065	0.00022	-0.00014	0.00123	0.00156	0.00436
32768	-0.00039	-0.00051	-0.00063	-0.00080	-0.00089	-0.00085	-0.00034	-0.00017	-0.00001	0.00180	0.00154	0.00407	0.00675	0.01228
16384	-0.00051	-0.00066	-0.00089	-0.00119	-0.00128	-0.00119	-0.00096	-0.00095	-0.00040	0.00146	0.00350	0.00766	0.01284	
8192	-0.00049	-0.00059	-0.00089	-0.00109	-0.00086	-0.00045	-0.00020	0.00054	0.00170	0.00562	0.01017	0.01938		
4096	-0.00050	-0.00057	-0.00067	-0.00063	-0.00044	0.00040	0.00201	0.00379	0.00685	0.01431	0.02496			
2048	-0.00060	-0.00074	-0.00094	-0.00081	-0.00024	0.00126	0.00260	0.00663	0.01366	0.02675				
1024	-0.00071	-0.00112	-0.00143	-0.00146	-0.00036	0.00148	0.00457	0.01029	0.02320					
512	-0.00105	-0.00130	-0.00141	-0.00081	0.00133	0.00641	0.01496	0.02933						
256	-0.00065	-0.00042	-0.00050	0.00101	0.00607	0.01649	0.03187							
128	-0.00095	-0.00030	0.00120	0.00568	0.01553	0.03499								
64	-0.00099	0.00035	0.00406	0.01493	0.03379									
32	-0.00014	0.00258	0.01064	0.03066										
16	0.00129	0.00890	0.02694											
8	0.00528	0.02096												
4	0.00998													

(d) $\mathcal{P}^{\mathcal{C}}$ for Scene: [**Veach Bidir**] Algorithm: [**MLT**] Metric: [**MAE**] True GT: [**BDPT @ 524288 spp**].

M-MLT	2	4	8	16	32	64	128	256	512	1024	2048	4096	8192	16384
GT (BDPT)														
262144	-0.00020	-0.00027	-0.00033	-0.00035	-0.00048	-0.00036	-0.00062	-0.00059	-0.00080	-0.00057	-0.00023	-0.00049	-0.00016	-0.00072
131072	-0.00042	-0.00056	-0.00067	-0.00078	-0.00098	-0.00082	-0.00087	-0.00089	-0.00104	-0.00084	0.00022	-0.00124	0.00080	0.00075
65536	-0.00044	-0.00053	-0.00075	-0.00074	-0.00106	-0.00077	-0.00090	-0.00083	-0.00083	-0.00069	0.00151	0.00034	0.00359	0.00394
32768	-0.00044	-0.00053	-0.00066	-0.00062	-0.00085	-0.00050	-0.00051	0.00006	0.00053	0.00190	0.00395	0.00426	0.00880	0.01329
16384	-0.00052	-0.00062	-0.00070	-0.00111	-0.00136	-0.00123	-0.00118	-0.00118	-0.00085	0.00096	0.00474	0.00587	0.01345	
8192	-0.00059	-0.00083	-0.00093	-0.00103	-0.00118	-0.00097	-0.00085	0.00019	0.00135	0.00532	0.01154	0.01775		
4096	-0.00044	-0.00042	-0.00038	-0.00033	-0.00006	0.00047	0.00170	0.00378	0.00771	0.01380	0.02692			
2048	-0.00043	-0.00046	-0.00038	-0.00056	-0.00061	0.00044	0.00311	0.00638	0.01328	0.02591				
1024	-0.00073	-0.00110	-0.00071	-0.00100	-0.00056	0.00098	0.00430	0.01093	0.02423					
512	-0.00081	-0.00110	-0.00041	0.00011	0.00141	0.00622	0.01379	0.02938						
256	-0.00077	-0.00056	0.00055	0.00265	0.00665	0.01655	0.03291							
128	-0.00065	-0.00024	0.00225	0.00618	0.01519	0.03499								
64	-0.00081	-0.00002	0.00449	0.01445	0.03177									
32	-0.00003	0.00317	0.01259	0.03357										
16	0.00113	0.00825	0.02692											
8	0.00564	0.02146												
4	0.01027													

(e) $\mathcal{P}^{\mathcal{C}}$ for Scene: [**Veach Bidir**] Algorithm: [**M-MLT**] Metric: [**MAE**] True GT: [**BDPT @ 524288 spp**].

ERPT	2	4	8	16	32	64	128	256	512	1024	2048	4096	8192	16384	32768	65536	131072
GT (BDPT)																	
262144	-0.00034	-0.00035	-0.00039	-0.00023	-0.00040	-0.00023	-0.00034	-0.00023	-0.00030	-0.00020	-0.00009	-0.00001	-0.00037	0.00008	-0.00017	-0.00052	-0.00094
131072	-0.00029	-0.00079	-0.00053	-0.00042	-0.00057	-0.00059	-0.00053	-0.00054	-0.00023	-0.00055	-0.00052	-0.00026	-0.00049	-0.00026	-0.00072	-0.00120	
65536	0.00005	-0.00056	-0.00007	-0.00019	-0.00036	-0.00042	-0.00040	-0.00012	0.00001	-0.00011	0.00027	0.00008	-0.00002	0.00017	-0.00025		
32768	0.00033	-0.00008	0.00040	-0.00002	-0.00006	0.00006	-0.00009	0.00015	0.00016	0.00041	0.00093	0.00056	0.00051	0.00071			
16384	0.00016	-0.00070	-0.00068	-0.00035	-0.00061	0.00021	-0.00036	0.00099	0.00132	0.00065	0.00101	0.00149	0.00127				
8192	0.00038	-0.00003	0.00071	0.00058	0.00086	0.00173	0.00110	0.00106	0.00164	0.00221	0.00277	0.00312					
4096	0.00364	0.00237	0.00305	0.00274	0.00326	0.00307	0.00303	0.00307	0.00472	0.00374	0.00397						
2048	0.00511	0.00536	0.00385	0.00452	0.00467	0.00541	0.00542	0.00677	0.00723	0.00647							
1024	0.00778	0.00687	0.00727	0.00747	0.00861	0.01290	0.00904	0.01255	0.01386								
512	0.01743	0.01680	0.01719	0.01809	0.02030	0.02606	0.02212	0.02437									
256	0.03996	0.03889	0.04179	0.04085	0.04401	0.04772	0.04706										
128	0.08169	0.07984	0.08303	0.07965	0.08660	0.09122											
64	0.15186	0.15100	0.15396	0.14929	0.15849												
32	0.27302	0.27223	0.27634	0.27601													
16	0.44216	0.44036	0.44750														
8	0.66528	0.66487															
4	0.92686																

(f) $\mathcal{P}^{\mathcal{C}}$ for Scene: [**Veach Bidir**] Algorithm: [**ERPT**] Metric: [**MAE**] True GT: [**BDPT @ 524288 spp**].

M-ERPT	2	4	8	16	32	64	128	256	512	1024	2048	4096	8192	16384	32768	65536	131072
GT (BDPT)																	
262144	-0.00021	-0.00042	-0.00032	-0.00027	-0.00029	-0.00009	-0.00002	-0.00011	0.00002	-0.00018	-0.00015	-0.00029	-0.00036	-0.00031	-0.00040	-0.00025	-0.00103
131072	-0.00059	-0.00045	-0.00078	-0.00039	-0.00067	-0.00014	-0.00019	-0.00014	-0.00020	-0.00046	-0.00043	-0.00030	-0.00075	-0.00082	-0.00091	-0.00082	
65536	-0.00052	-0.00070	-0.00063	-0.00023	-0.00057	-0.00002	0.00026	0.00012	0.00032	-0.00016	0.00026	0.00019	0.00006	0.00002	-0.00062		
32768	-0.00024	0.00010	0.00017	0.00020	-0.00020	0.00061	0.00096	0.00084	0.00052	0.00043	0.00085	0.00095	0.00051	0.00039			
16384	-0.00060	-0.00084	0.00020	0.00011	-0.00066	0.00063	0.00082	0.00127	0.00100	0.00141	0.00128	0.00172	0.00143				
8192	0.00054	-0.00023	0.00108	0.00113	0.00025	0.00150	0.00265	0.00196	0.00244	0.00227	0.00288	0.00258					
4096	0.00292	0.00238	0.00356	0.00391	0.00218	0.00405	0.00393	0.00468	0.00477	0.00427	0.00476						
2048	0.00353	0.00311	0.00647	0.00642	0.00548	0.00706	0.00634	0.00761	0.00670	0.00761							
1024	0.00685	0.00604	0.01194	0.00962	0.00880	0.01247	0.01452	0.01292	0.01341								
512	0.01673	0.01546	0.02440	0.02356	0.02145	0.02532	0.02805	0.02695									
256	0.03461	0.03655	0.04429	0.04604	0.04342	0.04856	0.04808										
128	0.07489	0.07352	0.08763	0.08519	0.08567	0.08971											
64	0.13810	0.14231	0.15458	0.15503	0.15452												
32	0.25043	0.25378	0.26677	0.26624													
16	0.41446	0.41700	0.42945														
8	0.62977	0.63452															
4	0.88580																

(g) $\mathcal{P}^{\mathcal{C}}$ for Scene: [**Veach Bidir**] Algorithm: [**M-ERPT**] Metric: [**MAE**] True GT: [**BDPT @ 524288 spp**].

Table 23

6.4 Veach Door

PT	2	4	8	16	32	64	128	256	512	1024	2048	4096	8192	16384	32768	65536	131072
GT (BDPT)																	
262144	-0.00006	-0.00004	-0.00004	-0.00003	-0.00000	-0.00009	0.00000	0.00001	-0.00006	0.00011	-0.00012	0.00026	0.00061	0.00223	0.00350	0.00783	0.01190
131072	-0.00010	-0.00002	-0.00004	-0.00000	0.00004	-0.00010	0.00007	0.00006	0.00018	0.00058	0.00073	0.00161	0.00290	0.00602	0.01252	0.02226	
65536	-0.00010	-0.00009	-0.00008	-0.00012	-0.00001	-0.00002	0.00004	0.00032	0.00064	0.00119	0.00205	0.00388	0.00775	0.01492	0.02691		
32768	-0.00003	-0.00000	-0.00012	-0.00005	0.00002	0.00004	0.00052	0.00115	0.00139	0.00290	0.00510	0.01032	0.01731	0.03246			
16384	-0.00006	-0.00016	-0.00023	-0.00025	-0.00022	0.00010	0.00082	0.00167	0.00338	0.00633	0.01048	0.01996	0.03625				
8192	-0.00003	-0.00028	-0.00053	-0.00071	-0.00043	0.00058	0.00164	0.00282	0.00647	0.01232	0.02271	0.04145					
4096	0.00010	-0.00021	-0.00057	-0.00020	0.00054	0.00173	0.00316	0.00742	0.01393	0.02674	0.04505						
2048	0.00022	-0.00045	-0.00137	-0.00031	0.00032	0.00367	0.00789	0.01494	0.02808	0.04950							
1024	0.00084	-0.00065	-0.00149	-0.00093	0.00165	0.00800	0.01663	0.03241	0.05486								
512	0.00124	-0.00073	-0.00238	-0.00138	0.00519	0.01628	0.03183	0.06028									
256	0.00232	-0.00159	-0.00304	-0.00130	0.01312	0.03687	0.06563										
128	0.00338	-0.00095	-0.00241	0.00602	0.03244	0.07327											
64	0.00524	0.00123	-0.00030	0.02111	0.07128												
32	0.00697	0.00399	0.01286	0.05534													
16	0.01155	0.01257	0.04163														
8	0.02163	0.03757															
4	0.03784																

(a) $\mathcal{P}^{\mathcal{C}}$ for Scene: [**Veach Door**] Algorithm: [**PT**] Metric: [**MAE**] True GT: [**BDPT @ 524288 spp**].

BDPT	2	4	8	16	32	64	128	256	512	1024	2048	4096	8192	16384	32768	65536	131072
GT (BDPT)																	
262144	-0.00005	-0.00005	-0.00003	-0.00005	-0.00003	0.00004	0.00000	-0.00002	0.00072	0.00053	0.00195	0.00345	0.00659	0.01232	0.02485	0.04157	0.07205
131072	-0.00003	-0.00004	-0.00004	0.00003	0.00003	-0.00002	0.00048	0.00093	0.00185	0.00278	0.00551	0.01049	0.02038	0.03940	0.07271	0.12873	
65536	-0.00011	-0.00012	-0.00006	-0.00003	0.00012	0.00017	0.00059	0.00149	0.00323	0.00594	0.01284	0.02588	0.04737	0.08911	0.16258		
32768	-0.00009	-0.00007	-0.00007	0.00017	0.00024	0.00046	0.00128	0.00323	0.00744	0.01421	0.02826	0.05489	0.10418	0.18407			
16384	-0.00016	-0.00023	-0.00029	-0.00028	-0.00000	0.00100	0.00257	0.00545	0.01335	0.02649	0.05365	0.10533	0.19109				
8192	-0.00025	-0.00055	-0.00074	-0.00043	-0.00010	0.00178	0.00484	0.01220	0.02663	0.05367	0.10520	0.19669					
4096	-0.00041	-0.00035	-0.00039	-0.00015	0.00123	0.00494	0.01113	0.02435	0.05331	0.10329	0.19582						
2048	-0.00068	-0.00089	-0.00091	-0.00027	0.00183	0.00950	0.02267	0.04968	0.09883	0.19119							
1024	-0.00079	-0.00141	-0.00155	0.00088	0.00655	0.01983	0.04688	0.09757	0.18688								
512	-0.00119	-0.00250	-0.00106	0.00215	0.01374	0.04120	0.09117	0.17837									
256	-0.00127	-0.00299	-0.00170	0.00901	0.03069	0.08339	0.16802										
128	-0.00084	-0.00186	0.00461	0.02711	0.07317	0.16271											
64	-0.00024	0.00235	0.02006	0.06449	0.14918												
32	0.00307	0.01326	0.05061	0.13836													
16	0.01702	0.04844	0.11996														
8	0.04324	0.10897															
4	0.09912																

(b) $\mathcal{P}^{\mathcal{C}}$ for Scene: [**Veach Door**] Algorithm: [**BDPT**] Metric: [**MAE**] True GT: [**BDPT @ 524288 spp**].

PSSMLT	2	4	8	16	32	64	128	256	512	1024	2048	4096	8192	16384
GT (BDPT)														
262144	-0.00005	-0.00007	-0.00013	-0.00009	-0.00007	0.00002	0.00042	0.00052	0.00117	0.00224	0.00390	0.00628	0.01335	0.02550
131072	-0.00001	-0.00005	0.00009	0.00006	0.00016	0.00046	0.00091	0.00158	0.00281	0.00594	0.01234	0.02297	0.04212	0.07671
65536	-0.00004	-0.00002	-0.00002	-0.00009	0.00017	0.00054	0.00156	0.00306	0.00696	0.01408	0.02820	0.05250	0.09289	0.16274
32768	-0.00006	0.00002	-0.00001	0.00030	0.00100	0.00217	0.00474	0.00810	0.01644	0.03143	0.06109	0.11044	0.18849	0.30456
16384	-0.00031	-0.00029	-0.00027	0.00017	0.00114	0.00291	0.00738	0.01352	0.02991	0.05970	0.11371	0.19943	0.32637	
8192	-0.00048	-0.00057	-0.00043	0.00054	0.00204	0.00617	0.01470	0.02948	0.05987	0.11430	0.20664	0.34191		
4096	-0.00055	-0.00067	-0.00007	0.00191	0.00611	0.01347	0.03040	0.06091	0.11632	0.21414	0.35348			
2048	-0.00141	-0.00119	0.00054	0.00443	0.01137	0.02810	0.05845	0.11139	0.20692	0.35351				
1024	-0.00215	-0.00132	0.00198	0.00953	0.02585	0.05791	0.11448	0.21198	0.35644					
512	-0.00338	-0.00098	0.00628	0.02198	0.05221	0.11043	0.20781	0.35644						
256	-0.00370	0.00174	0.01671	0.04722	0.10380	0.20277	0.35043							
128	-0.00173	0.01242	0.04272	0.09818	0.19527	0.34900								
64	0.00671	0.03456	0.09361	0.19262	0.34290									
32	0.02195	0.08468	0.19218	0.34287										
16	0.06829	0.17759	0.34540											
8	0.15054	0.32995												
4	0.29502													

(c) $\mathcal{P}^{\mathcal{C}}$ for Scene: [**Veach Door**] Algorithm: [**PSSMLT**] Metric: [**MAE**] True GT: [**BDPT @ 524288 spp**].

MLT	2	4	8	16	32	64	128	256	512	1024	2048	4096	8192	16384
GT (BDPT)														
262144	-0.00006	-0.00008	-0.00001	-0.00003	0.00012	0.00018	0.00025	0.00106	0.00125	0.00228	0.00526	0.01120	0.02116	0.03170
131072	-0.00005	0.00001	0.00018	0.00011	0.00034	0.00074	0.00138	0.00296	0.00520	0.00851	0.01861	0.03432	0.06068	0.10319
65536	-0.00008	-0.00007	0.00010	0.00010	0.00053	0.00161	0.00273	0.00587	0.01118	0.02103	0.03987	0.07981	0.13184	0.21554
32768	-0.00016	0.00002	0.00008	-0.00010	0.00098	0.00196	0.00535	0.01105	0.02348	0.04299	0.08574	0.15778	0.25883	0.38532
16384	-0.00043	-0.00040	-0.00027	0.00011	0.00102	0.00356	0.01071	0.02283	0.04596	0.08936	0.16669	0.28033	0.42866	
8192	-0.00079	-0.00081	-0.00052	0.00036	0.00257	0.00697	0.02113	0.04308	0.08938	0.16811	0.28963	0.45629		
4096	-0.00102	-0.00086	-0.00010	0.00188	0.00641	0.01515	0.04108	0.08804	0.16659	0.28793	0.46907			
2048	-0.00185	-0.00185	-0.00062	0.00391	0.01251	0.03073	0.07773	0.15853	0.28789	0.46250				
1024	-0.00288	-0.00194	0.00174	0.01071	0.03029	0.06755	0.15014	0.28241	0.46835					
512	-0.00482	-0.00231	0.00448	0.02386	0.05934	0.12634	0.26932	0.45799						
256	-0.00684	-0.00041	0.01625	0.05368	0.11954	0.23396	0.43678							
128	-0.00742	0.00813	0.04560	0.11636	0.23041	0.40317								
64	0.00018	0.03141	0.10607	0.22594	0.39738									
32	0.01501	0.08587	0.21615	0.39820										
16	0.05651	0.18473	0.38848											
8	0.14118	0.35044												
4	0.29152													

(d) $\mathcal{P}^{\mathcal{C}}$ for Scene: [**Veach Door**] Algorithm: [**MLT**] Metric: [**MAE**] True GT: [**BDPT @ 524288 spp**].

M-MLT	2	4	8	16	32	64	128	256	512	1024	2048	4096	8192	16384
GT (BDPT)														
262144	-0.00005	-0.00005	0.00004	0.00001	-0.00013	0.00001	0.00032	0.00086	0.00182	0.00313	0.00613	0.01097	0.01995	0.03219
131072	-0.00001	0.00003	0.00010	0.00027	0.00022	0.00063	0.00173	0.00228	0.00539	0.01002	0.01906	0.03351	0.05901	0.09505
65536	-0.00005	-0.00004	-0.00001	0.00006	0.00039	0.00127	0.00243	0.00587	0.01168	0.02479	0.04293	0.07733	0.13181	0.20825
32768	-0.00009	0.00001	0.00023	0.00026	0.00096	0.00201	0.00559	0.01044	0.02376	0.04657	0.08859	0.15188	0.24654	0.36788
16384	-0.00035	-0.00042	-0.00008	0.00028	0.00171	0.00352	0.00975	0.02111	0.04400	0.09220	0.16198	0.27200	0.41099	
8192	-0.00075	-0.00069	-0.00064	0.00052	0.00297	0.00759	0.01777	0.04127	0.08698	0.17109	0.28439	0.44340		
4096	-0.00072	-0.00062	0.00004	0.00159	0.00791	0.01627	0.04233	0.08429	0.16403	0.29934	0.46619			
2048	-0.00160	-0.00164	0.00068	0.00323	0.01444	0.03296	0.07789	0.15661	0.28308	0.47857				
1024	-0.00233	-0.00085	0.00319	0.00955	0.03434	0.07029	0.14651	0.27681	0.46497					
512	-0.00366	-0.00182	0.00714	0.02404	0.06530	0.13268	0.26199	0.45256						
256	-0.00425	0.00150	0.01947	0.05352	0.13071	0.24167	0.42736							
128	-0.00189	0.01345	0.05140	0.11877	0.24564	0.41288								
64	0.00609	0.04016	0.11645	0.22841	0.41679									
32	0.02988	0.09848	0.23470	0.40033										
16	0.07986	0.20495	0.41024											
8	0.17570	0.37875												
4	0.33591													

(e) $\mathcal{P}^{\mathcal{C}}$ for Scene: [**Veach Door**] Algorithm: [**M-MLT**] Metric: [**MAE**] True GT: [**BDPT @ 524288 spp**].

ERPT	2	4	8	16	32	64	128	256	512	1024	2048	4096	8192	16384	32768	65536	131072
GT (BDPT)																	
262144	0.00058	0.00040	0.00061	0.00043	0.00100	0.00022	0.00070	0.00062	0.00052	0.00043	0.00057	0.00051	0.00076	0.00060	0.00079	0.00046	0.00057
131072	0.00178	0.00171	0.00241	0.00184	0.00225	0.00219	0.00240	0.00189	0.00249	0.00224	0.00214	0.00200	0.00247	0.00156	0.00186	0.00213	
65536	0.00364	0.00340	0.00441	0.00404	0.00401	0.00419	0.00382	0.00463	0.00467	0.00389	0.00337	0.00418	0.00408	0.00385	0.00406		
32768	0.00738	0.00752	0.00908	0.00848	0.00903	0.00825	0.00812	0.00835	0.00838	0.00800	0.00780	0.00825	0.00875	0.00781			
16384	0.01083	0.01247	0.01515	0.01529	0.01587	0.01667	0.01517	0.01679	0.01396	0.01562	0.01514	0.01544	0.01489				
8192	0.02257	0.02219	0.03069	0.03121	0.02993	0.03345	0.03047	0.03256	0.02872	0.03138	0.03039	0.02933					
4096	0.04932	0.04997	0.05951	0.06105	0.06081	0.06389	0.06063	0.06417	0.06025	0.05902	0.06135						
2048	0.09439	0.09640	0.11301	0.11577	0.11654	0.11904	0.11106	0.11904	0.11106	0.11317							
1024	0.17529	0.17874	0.20574	0.20950	0.21191	0.21867	0.20650	0.21492	0.20609								
512	0.30387	0.31085	0.35357	0.35257	0.35908	0.36739	0.35100	0.36668									
256	0.48388	0.49252	0.54072	0.54739	0.55633	0.56436	0.55022										
128	0.71636	0.73180	0.79007	0.79624	0.80728	0.81408											
64	0.98491	0.99744	1.06150	1.07062	1.07911												
32	1.28309	1.30083	1.36515	1.37431													
16	1.59837	1.61705	1.68096														
8	1.93145	1.95110															
4	2.24791																

(f) $\mathcal{P}^{\mathcal{C}}$ for Scene: [**Veach Door**] Algorithm: [**ERPT**] Metric: [**MAE**] True GT: [**BDPT @ 524288 spp**].

M-ERPT	2	4	8	16	32	64	128	256	512	1024	2048	4096	8192	16384	32768	65536	131072
GT (BDPT)																	
262144	0.00028	0.00036	0.00018	0.00030	0.00068	0.00044	0.00083	0.00038	0.00050	0.00076	0.00014	0.00058	0.00047	0.00037	0.00067	0.00056	0.00044
131072	0.00177	0.00135	0.00160	0.00155	0.00134	0.00162	0.00199	0.00173	0.00159	0.00219	0.00156	0.00212	0.00186	0.00168	0.00156	0.00178	
65536	0.00342	0.00294	0.00268	0.00315	0.00350	0.00311	0.00393	0.00358	0.00300	0.00329	0.00320	0.00350	0.00415	0.00328	0.00334		
32768	0.00551	0.00659	0.00646	0.00648	0.00734	0.00730	0.00690	0.00674	0.00738	0.00648	0.00673	0.00685	0.00709	0.00682			
16384	0.01029	0.01111	0.01219	0.01344	0.01294	0.01281	0.01308	0.01310	0.01214	0.01280	0.01293	0.01403	0.01282				
8192	0.02066	0.02265	0.02324	0.02380	0.02511	0.02433	0.02613	0.02631	0.02478	0.02517	0.02752	0.02680					
4096	0.04374	0.04872	0.04922	0.04878	0.05096	0.04952	0.05421	0.05361	0.05318	0.05226	0.05299						
2048	0.08434	0.09072	0.09547	0.09375	0.09624	0.09671	0.10150	0.10154	0.09865	0.09882							
1024	0.15768	0.17091	0.17824	0.17642	0.17962	0.18033	0.18902	0.18917	0.18411								
512	0.27597	0.29580	0.30240	0.30703	0.31160	0.31183	0.32265	0.32282									
256	0.44949	0.47487	0.48458	0.49078	0.49349	0.49290	0.50811										
128	0.67085	0.70453	0.72060	0.72712	0.73561	0.72881											
64	0.92946	0.97298	0.98649	0.99694	1.00477												
32	1.22638	1.27279	1.28887	1.29846													
16	1.53591	1.58569	1.60771														
8	1.86729	1.92105															
4	2.18269																

(g) $\mathcal{P}^{\mathcal{C}}$ for Scene: [**Veach Door**] Algorithm: [**M-ERPT**] Metric: [**MAE**] True GT: [**BDPT @ 524288 spp**].

Table 24

6.5 Sponza

PT	2	4	8	16	32	64	128	256	512	1024	2048	4096	8192
**GT (PT)**													
16384	0.00002	-0.00002	0.00009	0.00039	0.00057	0.00082	0.00121	0.00451	0.00806	0.01511	0.02804	0.05240	0.08884
8192	-0.00002	0.00010	0.00020	0.00042	0.00167	0.00311	0.00480	0.01113	0.02356	0.04315	0.07896	0.14249	
4096	0.00004	0.00037	0.00075	0.00180	0.00379	0.00681	0.01270	0.02724	0.05291	0.09530	0.17006		
2048	0.00005	0.00040	0.00112	0.00276	0.00696	0.01350	0.02623	0.05455	0.10223	0.18399			
1024	0.00038	0.00134	0.00299	0.00668	0.01473	0.02873	0.05615	0.10521	0.19437				
512	0.00113	0.00325	0.00669	0.01449	0.02836	0.05485	0.10844	0.19536					
256	0.00147	0.00530	0.01242	0.02773	0.05598	0.10734	0.19417						
128	0.00337	0.01036	0.02493	0.05356	0.10715	0.19993							
64	0.00628	0.02142	0.05069	0.10401	0.19617								
32	0.01315	0.04116	0.09240	0.18749									
16	0.02621	0.08060	0.17177										
8	0.05070	0.14737											
4	0.09339												

(a) $\mathcal{P}^{\mathcal{C}}$ for Scene: [**Sponza**] Algorithm: [**PT**] Metric: [**MAE**] True GT: [**PT @ 32768 spp**].

BDPT	2	4	8	16	32	64	128	256	512	1024	2048	4096	8192
**GT (PT)**													
16384	-0.00005	-0.00013	-0.00000	0.00018	0.00026	0.00037	0.00111	0.00259	0.00651	0.01300	0.02189	0.04097	0.07308
8192	-0.00002	-0.00003	0.00027	0.00045	0.00132	0.00223	0.00416	0.00879	0.01795	0.03596	0.06351	0.11737	
4096	0.00000	0.00005	0.00057	0.00113	0.00313	0.00519	0.01018	0.02078	0.04085	0.07980	0.14427		
2048	0.00010	0.00030	0.00122	0.00241	0.00533	0.01056	0.02284	0.04399	0.08505	0.15719			
1024	-0.00000	0.00023	0.00237	0.00487	0.01114	0.02205	0.04337	0.08511	0.16069				
512	0.00001	0.00211	0.00451	0.01141	0.02251	0.04437	0.08798	0.16460					
256	0.00031	0.00304	0.00942	0.02056	0.04369	0.08203	0.16240						
128	0.00073	0.00668	0.01931	0.03971	0.08350	0.16179							
64	0.00081	0.01345	0.03691	0.07939	0.15523								
32	0.00237	0.02818	0.07083	0.14595									
16	0.00911	0.05025	0.13061										
8	0.02154	0.09840											
4	0.04388												

(b) $\mathcal{P}^{\mathcal{C}}$ for Scene: [**Sponza**] Algorithm: [**BDPT**] Metric: [**MAE**] True GT: [**PT @ 32768 spp**].

PSSMLT	2	4	8	16	32	64	128	256	512	1024	2048	4096	8192
**GT (PT)**													
16384	0.00006	0.00018	0.00005	0.00013	0.00052	0.00048	0.00118	0.00239	0.00450	0.00750	0.01064	0.01616	0.02284
8192	0.00016	0.00025	0.00044	0.00105	0.00147	0.00227	0.00491	0.00761	0.01332	0.02039	0.03128	0.04549	
4096	0.00021	0.00067	0.00127	0.00203	0.00390	0.00617	0.01137	0.01846	0.03014	0.04610	0.06704		
2048	0.00039	0.00113	0.00211	0.00405	0.00851	0.01274	0.02315	0.03864	0.06195	0.09352			
1024	0.00081	0.00182	0.00436	0.00879	0.01500	0.02790	0.04712	0.07636	0.12088				
512	0.00275	0.00567	0.00992	0.01756	0.03230	0.05429	0.09087	0.14526					
256	0.00439	0.00931	0.01830	0.03454	0.06309	0.10550	0.16976						
128	0.00957	0.01904	0.03665	0.06699	0.12094	0.19326							
64	0.01774	0.03758	0.07338	0.12870	0.21236								
32	0.03438	0.07095	0.12954	0.22396									
16	0.06736	0.13359	0.23533										
8	0.12238	0.22757											
4	0.20778												

(c) $\mathcal{P}^{\mathcal{C}}$ for Scene: [**Sponza**] Algorithm: [**PSSMLT**] Metric: [**MAE**] True GT: [**PT @ 32768 spp**].

MLT	2	4	8	16	32	64	128	256	512	1024	2048	4096	8192
**GT (PT)**													
16384	0.00011	0.00027	0.00043	0.00029	0.00128	0.00159	0.00324	0.00552	0.00814	0.01353	0.02041	0.02655	0.03332
8192	0.00013	0.00019	0.00121	0.00151	0.00346	0.00513	0.01114	0.01635	0.02684	0.03970	0.05510	0.06998	
4096	0.00057	0.00111	0.00252	0.00405	0.00770	0.01305	0.02287	0.03782	0.05850	0.08593	0.11132		
2048	0.00100	0.00209	0.00469	0.00844	0.01685	0.02847	0.04968	0.07673	0.11606	0.16004			
1024	0.00183	0.00471	0.00943	0.01824	0.03456	0.06003	0.09760	0.14664	0.21204				
512	0.00389	0.00899	0.01756	0.03495	0.06536	0.10911	0.17900	0.25666					
256	0.00713	0.01793	0.03570	0.07309	0.12546	0.20427	0.30652						
128	0.01389	0.03363	0.07046	0.13072	0.22376	0.34787							
64	0.02639	0.06355	0.12853	0.22922	0.36707								
32	0.04949	0.11574	0.22897	0.38060									
16	0.09213	0.20899	0.37757										
8	0.16400	0.34549											
4	0.27359												

(d) $\mathcal{P}^{\mathcal{C}}$ for Scene: [**Sponza**] Algorithm: [**MLT**] Metric: [**MAE**] True GT: [**PT @ 32768 spp**].

M-MLT	2	4	8	16	32	64	128	256	512	1024	2048	4096	8192
**GT (PT)**													
16384	0.00021	0.00020	0.00045	0.00026	0.00121	0.00145	0.00397	0.00613	0.00899	0.01398	0.02087	0.02643	0.03273
8192	0.00008	0.00034	0.00079	0.00169	0.00351	0.00552	0.01122	0.01662	0.02898	0.03902	0.05561	0.06952	
4096	0.00061	0.00106	0.00193	0.00385	0.00832	0.01451	0.02354	0.04195	0.06051	0.08625	0.11457		
2048	0.00119	0.00189	0.00439	0.00926	0.01758	0.02879	0.05108	0.07997	0.11926	0.16012			
1024	0.00248	0.00404	0.00945	0.01854	0.03396	0.06077	0.09880	0.15107	0.21582				
512	0.00487	0.00861	0.01729	0.03541	0.06666	0.11214	0.18381	0.26733					
256	0.01059	0.01552	0.03603	0.07168	0.12518	0.20492	0.31452						
128	0.01893	0.03298	0.06972	0.13301	0.22697	0.34813							
64	0.03590	0.06134	0.12973	0.23396	0.37797								
32	0.06652	0.11411	0.22556	0.38891									
16	0.12455	0.20584	0.38130										
8	0.21607	0.34023											
4	0.34630												

(e) $\mathcal{P}^{\mathcal{C}}$ for Scene: [**Sponza**] Algorithm: [**M-MLT**] Metric: [**MAE**] True GT: [**PT @ 32768 spp**].

ERPT	2	4	8	16	32	64	128	256	512	1024	2048	4096	8192
**GT (PT)**													
16384	0.00516	0.00423	0.00396	0.00376	0.00492	0.00503	0.00521	0.00428	0.00480	0.00463	0.00504	0.00421	0.00408
8192	0.01259	0.01238	0.01322	0.01203	0.01391	0.01401	0.01306	0.01262	0.01318	0.01197	0.01331	0.01308	
4096	0.02748	0.02775	0.02854	0.02877	0.02995	0.03129	0.02897	0.02916	0.03000	0.02955	0.02944		
2048	0.05691	0.05981	0.06005	0.05999	0.06151	0.06342	0.06112	0.06305	0.06370	0.06061			
1024	0.11159	0.11533	0.11811	0.11705	0.12090	0.12090	0.11910	0.11711	0.11873				
512	0.20184	0.20805	0.20816	0.21122	0.21151	0.21571	0.21282	0.21420					
256	0.33990	0.34836	0.35422	0.35538	0.36068	0.35998	0.35760						
128	0.53769	0.54937	0.55411	0.55926	0.56043	0.56288							
64	0.77396	0.78969	0.79859	0.80118	0.80476								
32	1.05511	1.07028	1.07582	1.08326									
16	1.35613	1.37660	1.38193										
8	1.66502	1.68471											
4	1.96704												

(f) $\mathcal{P}^{\mathcal{C}}$ for Scene: [**Sponza**] Algorithm: [**ERPT**] Metric: [**MAE**] True GT: [**PT @ 32768 spp**].

M-ERPT	2	4	8	16	32	64	128	256	512	1024	2048	4096	8192
**GT (PT)**													
16384	0.00378	0.00395	0.00353	0.00455	0.00448	0.00354	0.00400	0.00312	0.00452	0.00369	0.00464	0.00324	0.00335
8192	0.01154	0.01283	0.01237	0.01297	0.01246	0.01252	0.01192	0.01192	0.01153	0.01190	0.01259	0.01173	
4096	0.02678	0.02775	0.02804	0.02752	0.02913	0.02745	0.02780	0.02615	0.02557	0.02709	0.02657		
2048	0.05447	0.05548	0.05739	0.05542	0.05953	0.05725	0.05482	0.05667	0.05848	0.05651			
1024	0.10790	0.10644	0.11130	0.10991	0.11286	0.11174	0.11305	0.11415	0.11163				
512	0.19064	0.19663	0.19880	0.19929	0.20414	0.20265	0.19988	0.20025					
256	0.32337	0.33548	0.33870	0.33881	0.34771	0.34339	0.33716						
128	0.51827	0.53017	0.53468	0.53084	0.54404	0.54046							
64	0.75311	0.76563	0.77364	0.77395	0.78255								
32	1.02982	1.04607	1.05346	1.05488									
16	1.33149	1.34951	1.35865										
8	1.64027	1.65839											
4	1.94197												

(g) $\mathcal{P}^{\mathcal{C}}$ for Scene: [**Sponza**] Algorithm: [**M-ERPT**] Metric: [**MAE**] True GT: [**PT @ 32768 spp**].

Table 25

7 RMSE

7.1 Cornell Box

PT	2	4	8	16	32	64	128	256	512	1024	2048	4096	8192
GT (PT)													
16384	0.00006	0.00013	0.00009	0.00004	0.00047	0.00083	0.00178	0.00281	0.00768	0.01419	0.02665	0.04001	0.06881
8192	0.00015	0.00046	0.00027	0.00061	0.00092	0.00217	0.00569	0.01011	0.02069	0.03927	0.07090	0.11288	
4096	0.00031	0.00061	0.00069	0.00154	0.00330	0.00544	0.01410	0.02680	0.04919	0.08857	0.14771		
2048	0.00082	0.00093	0.00192	0.00353	0.00694	0.01439	0.02798	0.05542	0.09994	0.17343			
1024	0.00093	0.00172	0.00391	0.00773	0.01465	0.02837	0.05609	0.10289	0.18678				
512	0.00209	0.00419	0.00727	0.01529	0.03062	0.05567	0.10708	0.18699					
256	0.00419	0.00790	0.01417	0.03052	0.05627	0.11024	0.20056						
128	0.00810	0.01580	0.03082	0.05920	0.11038	0.20074							
64	0.01551	0.02976	0.05884	0.11263	0.20004								
32	0.03202	0.05805	0.11172	0.20089									
16	0.06147	0.11266	0.20002										
8	0.11039	0.20287											
4	0.19831												

(a) $\mathcal{P}^{\mathcal{C}}$ for Scene: [**Cornell Box**] Algorithm: [**PT**] Metric: [**RMSE**] True GT: [**PT @ 32768 spp**].

BDPT	2	4	8	16	32	64	128	256	512	1024	2048	4096	8192
GT (PT)													
16384	0.00010	0.00022	0.00018	0.00073	0.00125	0.00302	0.00562	0.00928	0.01981	0.03003	0.05124	0.07338	0.10453
8192	0.00041	0.00044	0.00114	0.00219	0.00395	0.00758	0.01607	0.02951	0.05351	0.08817	0.13557	0.19111	
4096	0.00033	0.00150	0.00240	0.00492	0.00911	0.01880	0.03582	0.06768	0.11500	0.18757	0.27333		
2048	0.00103	0.00210	0.00501	0.00982	0.02089	0.04075	0.07343	0.13208	0.22096	0.33390			
1024	0.00289	0.00571	0.01052	0.02049	0.04004	0.07453	0.13893	0.24352	0.37855				
512	0.00523	0.01117	0.02176	0.04236	0.07907	0.14617	0.25181	0.40707					
256	0.01060	0.02055	0.04238	0.08319	0.15192	0.26099	0.42885						
128	0.02114	0.04264	0.08218	0.15001	0.27275	0.43721							
64	0.04112	0.08033	0.15371	0.26795	0.44237								
32	0.07903	0.14953	0.26513	0.43887									
16	0.14531	0.26076	0.43162										
8	0.25273	0.42327											
4	0.40964												

(b) $\mathcal{P}^{\mathcal{C}}$ for Scene: [**Cornell Box**] Algorithm: [**BDPT**] Metric: [**RMSE**] True GT: [**PT @ 32768 spp**].

PSSMLT	2	4	8	16	32	64	128	256	512	1024	2048	4096	8192
GT (PT)													
16384	0.00008	0.00004	0.00016	0.00037	0.00113	0.00307	0.00499	0.00686	0.00903	0.01260	0.01387	0.01551	0.01534
8192	0.00030	0.00040	0.00045	0.00140	0.00330	0.00722	0.01109	0.01836	0.02502	0.03253	0.03756	0.04119	
4096	0.00053	0.00102	0.00240	0.00436	0.00876	0.01686	0.02843	0.04359	0.06007	0.07548	0.08651		
2048	0.00127	0.00234	0.00486	0.00913	0.01920	0.03410	0.05700	0.08851	0.11794	0.14738			
1024	0.00258	0.00554	0.01069	0.01977	0.03655	0.06699	0.11008	0.16640	0.21714				
512	0.00544	0.01070	0.02057	0.04003	0.07475	0.13024	0.20605	0.29771					
256	0.01151	0.02309	0.04405	0.08161	0.14442	0.23977	0.35917						
128	0.02262	0.04449	0.08295	0.15090	0.25715	0.40004							
64	0.04447	0.08421	0.15284	0.26557	0.42468								
32	0.08266	0.15577	0.26551	0.43155									
16	0.14974	0.26726	0.43576										
8	0.26078	0.43085											
4	0.42702												

(c) $\mathcal{P}^{\mathcal{C}}$ for Scene: [**Cornell Box**] Algorithm: [**PSSMLT**] Metric: [**RMSE**] True GT: [**PT @ 32768 spp**].

MLT	2	4	8	16	32	64	128	256	512	1024	2048	4096	8192
GT (PT)													
16384	0.00000	-0.00001	0.00007	-0.00002	0.00010	0.00037	0.00073	0.00213	0.00374	0.00570	0.00701	0.01138	0.01288
8192	0.00003	0.00012	0.00016	0.00032	0.00058	0.00155	0.00303	0.00596	0.01036	0.01664	0.02296	0.03023	
4096	0.00007	0.00023	0.00041	0.00085	0.00225	0.00460	0.00824	0.01470	0.02574	0.03812	0.05437		
2048	0.00031	0.00053	0.00084	0.00127	0.00363	0.00766	0.01514	0.02943	0.05015	0.07650			
1024	0.00059	0.00107	0.00217	0.00421	0.00875	0.01742	0.03248	0.05853	0.09786				
512	0.00149	0.00246	0.00505	0.00947	0.01850	0.03519	0.06480	0.11421					
256	0.00252	0.00540	0.01080	0.01971	0.03791	0.06979	0.12331						
128	0.00457	0.00866	0.01729	0.03353	0.06552	0.12251							
64	0.00826	0.01730	0.03673	0.06934	0.12799								
32	0.01839	0.03447	0.06812	0.12516									
16	0.03499	0.06491	0.12772										
8	0.06175	0.11777											
4	0.12164												

(d) $\mathcal{P}^{\mathcal{C}}$ for Scene: [**Cornell Box**] Algorithm: [**MLT**] Metric: [**RMSE**] True GT: [**PT @ 32768 spp**].

M-MLT	2	4	8	16	32	64	128	256	512	1024	2048	4096	8192
GT (PT)													
16384	0.00004	-0.00011	-0.00006	-0.00001	-0.00010	0.00035	0.00106	0.00244	0.00373	0.00617	0.00868	0.01088	0.01308
8192	0.00009	0.00012	0.00030	0.00052	0.00098	0.00180	0.00313	0.00613	0.01188	0.01769	0.02469	0.03014	
4096	0.00009	0.00018	0.00047	0.00105	0.00206	0.00435	0.00847	0.01487	0.02621	0.03960	0.05548		
2048	0.00044	0.00060	0.00122	0.00247	0.00443	0.00853	0.01583	0.03025	0.05148	0.07959			
1024	0.00065	0.00085	0.00222	0.00469	0.00937	0.01825	0.03368	0.06167	0.10112				
512	0.00104	0.00270	0.00518	0.01003	0.01801	0.03529	0.06430	0.11386					
256	0.00271	0.00542	0.00995	0.01889	0.03589	0.06878	0.12423						
128	0.00441	0.00904	0.01897	0.03695	0.07005	0.13139							
64	0.00867	0.01929	0.03736	0.06913	0.12802								
32	0.01955	0.03783	0.07257	0.13247									
16	0.03742	0.07318	0.13426										
8	0.07130	0.12932											
4	0.12885												

(e) $\mathcal{P}^{\mathcal{C}}$ for Scene: [**Cornell Box**] Algorithm: [**M-MLT**] Metric: [**RMSE**] True GT: [**PT @ 32768 spp**].

ERPT	2	4	8	16	32	64	128	256	512	1024	2048	4096	8192
GT (PT)													
16384	0.00132	0.00162	0.00101	0.00137	0.00101	0.00137	0.00138	0.00131	0.00180	0.00144	0.00085	0.00130	0.00163
8192	0.00400	0.00384	0.00403	0.00324	0.00367	0.00355	0.00341	0.00415	0.00385	0.00381	0.00348	0.00416	
4096	0.00830	0.00902	0.00798	0.00751	0.00810	0.00947	0.00879	0.00852	0.00840	0.00865	0.00826		
2048	0.01794	0.01821	0.01813	0.01751	0.01720	0.01873	0.01758	0.01692	0.01693	0.01831			
1024	0.03643	0.03499	0.03505	0.03520	0.03491	0.03761	0.03649	0.03533	0.03759				
512	0.07096	0.06944	0.06987	0.07001	0.07091	0.07509	0.07107	0.07213					
256	0.13405	0.13805	0.13556	0.13481	0.13482	0.13921	0.13525						
128	0.24032	0.24333	0.24260	0.24309	0.23802	0.24695							
64	0.40070	0.40451	0.40213	0.40131	0.39498								
32	0.61597	0.61917	0.62137	0.61959									
16	0.87516	0.88485	0.88443										
8	1.16912	1.17775											
4	1.47538												

(f) $\mathcal{P}^{\mathcal{C}}$ for Scene: [**Cornell Box**] Algorithm: [**ERPT**] Metric: [**RMSE**] True GT: [**PT @ 32768 spp**].

M-ERPT	2	4	8	16	32	64	128	256	512	1024	2048	4096	8192
GT (PT)													
16384	0.00124	0.00128	0.00132	0.00152	0.00132	0.00178	0.00145	0.00146	0.00158	0.00090	0.00062	0.00136	0.00127
8192	0.00385	0.00344	0.00333	0.00448	0.00339	0.00306	0.00307	0.00364	0.00291	0.00322	0.00319	0.00365	
4096	0.00773	0.00749	0.00776	0.00812	0.00812	0.00779	0.00774	0.00747	0.00784	0.00755	0.00801		
2048	0.01706	0.01639	0.01656	0.01734	0.01791	0.01709	0.01614	0.01570	0.01557	0.01677			
1024	0.03263	0.03320	0.03509	0.03381	0.03434	0.03305	0.03236	0.03574	0.03305				
512	0.06428	0.06584	0.06497	0.06779	0.06706	0.06447	0.06708	0.06514					
256	0.12714	0.12926	0.12864	0.12787	0.13011	0.12657	0.13084						
128	0.22746	0.22900	0.22807	0.23286	0.23261	0.22839							
64	0.38943	0.38749	0.38379	0.38516	0.39097								
32	0.59553	0.59925	0.59446	0.59788									
16	0.85259	0.85908	0.85064										
8	1.14355	1.14825											
4	1.45060												

(g) $\mathcal{P}^{\mathcal{C}}$ for Scene: [**Cornell Box**] Algorithm: [**M-ERPT**] Metric: [**RMSE**] True GT: [**PT @ 32768 spp**].

Table 26

7.2 Torus

PT	2	4	8	16	32	64	128	256	512	1024	2048	4096	8192	16384	32768	65536	131072
GT (BDPT)																	
262144	-0.00066	-0.00104	-0.00078	-0.00068	-0.00116	-0.00066	-0.00032	0.00040	-0.00014	0.00052	0.00143	0.00336	0.00745	0.01331	0.02431	0.05022	0.08547
131072	-0.00106	-0.00061	-0.00108	-0.00064	-0.00161	0.00023	0.00066	0.00117	0.00227	0.00258	0.00586	0.01449	0.02376	0.04723	0.08503	0.14368	
65536	-0.00082	0.00006	-0.00083	-0.00006	0.00039	0.00045	0.00243	0.00313	0.00534	0.00844	0.01595	0.02994	0.05654	0.09777	0.16714		
32768	0.00254	0.00442	0.00512	0.00657	0.00673	0.00661	0.00690	0.01088	0.01434	0.02172	0.03479	0.06328	0.11527	0.19842			
16384	0.00696	0.01146	0.01429	0.01608	0.01452	0.01857	0.01819	0.02492	0.02982	0.04423	0.06700	0.12400	0.20734				
8192	0.01512	0.02135	0.02505	0.02862	0.03115	0.03427	0.03557	0.04795	0.05323	0.08374	0.12431	0.21523					
4096	0.03280	0.04467	0.05128	0.06034	0.05587	0.06325	0.06897	0.08149	0.09831	0.14192	0.21505						
2048	0.05477	0.07632	0.08997	0.10147	0.10128	0.10672	0.11506	0.13481	0.16633	0.22940							
1024	0.07348	0.10784	0.12731	0.14274	0.14198	0.15306	0.16488	0.19396	0.24019								
512	0.09390	0.13623	0.16280	0.17969	0.18435	0.20381	0.21452	0.25098									
256	0.09794	0.13968	0.16861	0.18392	0.19065	0.21612	0.23659										
128	0.05748	0.09321	0.11258	0.14121	0.16425	0.19115											
64	0.02903	0.07257	0.07703	0.10928	0.13680												
32	-0.00770	0.02218	0.02546	0.07175													
16	-0.05458	-0.03429	-0.04622														
8	-0.05513	-0.03204															
4	-0.02786																

(a) $\mathcal{P}^{\mathcal{C}}$ for Scene: [**Torus**] Algorithm: [**PT**] Metric: [**RMSE**] True GT: [**BDPT @ 524288 spp**].

BDPT	2	4	8	16	32	64	128	256	512	1024	2048	4096	8192	16384	32768	65536	131072
GT (BDPT)																	
262144	-0.00080	-0.00116	-0.00117	-0.00091	0.00018	-0.00070	-0.00042	-0.00044	0.00001	0.00034	0.00279	0.00198	0.00721	0.01229	0.02363	0.04993	0.08667
131072	-0.00077	-0.00140	-0.00116	-0.00072	-0.00075	0.00018	0.00052	0.00061	0.00171	0.00387	0.00775	0.01165	0.02356	0.04281	0.07388	0.14395	
65536	-0.00080	-0.00095	-0.00090	-0.00020	0.00060	0.00048	0.00219	0.00429	0.00492	0.00866	0.01725	0.02593	0.04956	0.09368	0.16112		
32768	0.00201	0.00443	0.00521	0.00723	0.00536	0.00779	0.00906	0.01253	0.01170	0.02236	0.03826	0.06316	0.10561	0.18291			
16384	0.00642	0.01111	0.01291	0.01485	0.01483	0.01657	0.01860	0.02386	0.03017	0.04609	0.07413	0.11963	0.19028				
8192	0.01263	0.01957	0.02434	0.02533	0.02540	0.03209	0.03859	0.04211	0.06022	0.08076	0.12151	0.20340					
4096	0.02713	0.04102	0.04842	0.05802	0.05651	0.06142	0.06681	0.08145	0.09902	0.13578	0.21279						
2048	0.05088	0.07366	0.08274	0.09318	0.09759	0.10363	0.11357	0.12889	0.16478	0.22206							
1024	0.07011	0.09989	0.12234	0.13297	0.14344	0.14370	0.16903	0.18923	0.24195								
512	0.08798	0.12662	0.15469	0.16914	0.17935	0.18989	0.21639	0.24112									
256	0.08570	0.13154	0.16055	0.17476	0.19020	0.21189	0.23352										
128	0.05045	0.08486	0.11308	0.12636	0.14766	0.17572											
64	0.02508	0.05112	0.07559	0.10371	0.13863												
32	-0.01152	-0.00365	0.03179	0.05720													
16	-0.05916	-0.05516	-0.02607														
8	-0.06079	-0.05514															
4	-0.03090																

(b) $\mathcal{P}^{\mathcal{C}}$ for Scene: [**Torus**] Algorithm: [**BDPT**] Metric: [**RMSE**] True GT: [**BDPT @ 524288 spp**].

PSSMLT	2	4	8	16	32	64	128	256	512	1024	2048	4096	8192	16384
GT (BDPT)														
262144	-0.00106	-0.00117	-0.00094	-0.00121	-0.00079	-0.00098	0.00019	0.00053	0.00144	0.00297	0.00406	0.00534	0.01057	0.00764
131072	-0.00077	-0.00102	-0.00098	-0.00088	0.00030	0.00112	0.00286	0.00525	0.00572	0.01054	0.01668	0.02171	0.03879	0.03288
65536	-0.00118	-0.00010	0.00154	0.00124	0.00332	0.00467	0.00706	0.01288	0.01723	0.02809	0.04116	0.05510	0.08900	0.08096
32768	0.00058	0.00347	0.00501	0.00618	0.01013	0.01425	0.02005	0.03157	0.04419	0.06657	0.09076	0.11575	0.17438	0.16344
16384	0.00692	0.01290	0.01732	0.02269	0.02849	0.03605	0.04973	0.06756	0.08990	0.12789	0.17274	0.21758	0.30928	
8192	0.01142	0.01940	0.02749	0.03166	0.04638	0.06513	0.08669	0.11976	0.16211	0.22223	0.29931	0.36561		
4096	0.02279	0.04028	0.05101	0.06049	0.08252	0.10984	0.14720	0.20101	0.26510	0.35434	0.45581			
2048	0.04948	0.07754	0.09792	0.11542	0.14621	0.18964	0.24114	0.31917	0.40252	0.52258				
1024	0.06789	0.10880	0.13923	0.16173	0.20390	0.26167	0.33945	0.43644	0.54654					
512	0.08665	0.13813	0.17470	0.20205	0.26265	0.33583	0.41965	0.53446						
256	0.09124	0.14947	0.19749	0.23153	0.29854	0.38520	0.48312							
128	0.05556	0.11618	0.16840	0.20755	0.28512	0.37921								
64	0.04364	0.10685	0.16094	0.19519	0.28790									
32	0.00682	0.07268	0.13390	0.17185										
16	-0.02571	0.04043	0.10435											
8	-0.02767	0.04011												
4	-0.00044													

(c) $\mathcal{P}^{\mathcal{C}}$ for Scene: [**Torus**] Algorithm: [**PSSMLT**] Metric: [**RMSE**] True GT: [**BDPT @ 524288 spp**].

MLT	2	4	8	16	32	64	128	256	512	1024	2048	4096	8192	16384
GT (BDPT)														
262144	-0.00055	-0.00075	-0.00034	-0.00031	0.00013	-0.00003	0.00066	0.00137	0.00057	0.00143	0.00138	0.00152	0.00182	-0.00020
131072	-0.00087	-0.00016	0.00139	0.00199	0.00313	0.00376	0.00438	0.00582	0.00572	0.00664	0.00764	0.00750	0.00868	0.00949
65536	-0.00168	-0.00064	0.00165	0.00313	0.00644	0.00718	0.00925	0.01327	0.01344	0.01580	0.01675	0.01756	0.01989	0.02240
32768	-0.00106	-0.00005	0.00554	0.00846	0.01517	0.01564	0.01986	0.03029	0.02907	0.03359	0.03560	0.03650	0.04246	0.05004
16384	-0.00126	0.00081	0.00941	0.01395	0.02920	0.02879	0.03636	0.05026	0.05167	0.06049	0.06290	0.06643	0.07540	
8192	-0.00035	0.00514	0.02525	0.03003	0.05587	0.06060	0.07758	0.10288	0.10549	0.12037	0.12592	0.13071		
4096	-0.00023	0.00986	0.04279	0.05732	0.09947	0.10518	0.13373	0.17320	0.17613	0.19913	0.20798			
2048	0.00063	0.01975	0.07406	0.09460	0.16393	0.17374	0.21071	0.26562	0.27133	0.30115				
1024	0.00500	0.03570	0.11015	0.14298	0.24107	0.25794	0.30944	0.37654	0.38186					
512	0.00368	0.04370	0.14566	0.18256	0.30865	0.32502	0.38534	0.47152						
256	-0.00235	0.05275	0.17309	0.21469	0.35567	0.38071	0.44956							
128	-0.01767	0.04418	0.16712	0.21485	0.36221	0.38653								
64	-0.02422	0.04708	0.16799	0.22160	0.38189									
32	-0.03827	0.03436	0.15479	0.21061										
16	-0.05371	0.02394	0.13849											
8	-0.04777	0.02804												
4	-0.05115													

(d) $\mathcal{P}^{\mathcal{C}}$ for Scene: [**Torus**] Algorithm: [**MLT**] Metric: [**RMSE**] True GT: [**BDPT @ 524288 spp**].

M-MLT	2	4	8	16	32	64	128	256	512	1024	2048	4096	8192	16384
GT (BDPT)														
262144	-0.00027	-0.00022	-0.00029	-0.00030	-0.00076	0.00017	0.00196	0.00348	0.00294	0.00380	0.00483	0.00518	0.00542	0.00369
131072	-0.00049	-0.00029	0.00094	0.00027	0.00133	0.00451	0.00660	0.00711	0.00931	0.01191	0.01290	0.01404	0.01629	0.01814
65536	-0.00114	-0.00014	0.00209	0.00293	0.00407	0.00900	0.01394	0.01999	0.02116	0.02538	0.02956	0.02984	0.03489	0.04184
32768	-0.00104	0.00088	0.00737	0.00885	0.01351	0.02062	0.03089	0.03996	0.04503	0.05152	0.05917	0.06117	0.06930	0.08771
16384	-0.00097	0.00347	0.01346	0.01241	0.02436	0.03739	0.05327	0.07264	0.07947	0.09306	0.10483	0.10689	0.12300	
8192	-0.00029	0.00944	0.02602	0.02840	0.04986	0.07649	0.10056	0.13688	0.15510	0.17282	0.19695	0.20031		
4096	0.00220	0.01745	0.05112	0.04694	0.08497	0.13102	0.17349	0.22327	0.24390	0.27732	0.30774			
2048	0.00375	0.02948	0.08418	0.08623	0.14993	0.20587	0.26518	0.33732	0.36697	0.40988				
1024	0.00873	0.05029	0.12859	0.13158	0.21175	0.29995	0.38000	0.46413	0.49847					
512	0.01092	0.06416	0.17813	0.17126	0.26490	0.37043	0.46824	0.56704						
256	0.00959	0.07396	0.20635	0.20118	0.31183	0.43086	0.53934							
128	-0.00089	0.05977	0.21332	0.19991	0.31740	0.44651								
64	-0.00520	0.05670	0.23394	0.21536	0.34192									
32	-0.01423	0.04522	0.22918	0.20632										
16	-0.01868	0.03767	0.22767											
8	-0.01765	0.04076												
4	-0.01842													

(e) $\mathcal{P}^{\mathcal{C}}$ for Scene: [**Torus**] Algorithm: [**M-MLT**] Metric: [**RMSE**] True GT: [**BDPT @ 524288 spp**].

ERPT	2	4	8	16	32	64	128	256	512	1024	2048	4096	8192	16384	32768	65536	131072
GT (BDPT)																	
262144	-0.00067	0.00012	0.00173	0.00158	0.00542	0.00501	0.00522	0.00527	0.00646	0.00673	0.00721	0.00907	0.00966	0.00847	0.00895	0.00791	0.00809
131072	0.00326	0.00639	0.00711	0.01135	0.01055	0.01450	0.01615	0.01754	0.02136	0.02523	0.02516	0.03123	0.03207	0.02994	0.02901	0.02867	
65536	0.00901	0.01474	0.01870	0.02255	0.02757	0.02908	0.03644	0.03841	0.04357	0.05034	0.05913	0.06650	0.07018	0.06745	0.06651		
32768	0.02259	0.03215	0.03817	0.05145	0.05882	0.06440	0.07345	0.08237	0.08934	0.10722	0.12204	0.13816	0.14077	0.13039			
16384	0.04843	0.06750	0.08182	0.09552	0.10282	0.11995	0.13823	0.15895	0.16990	0.19788	0.22406	0.24509	0.25423				
8192	0.09036	0.12701	0.14828	0.17296	0.18488	0.21372	0.24416	0.27785	0.29146	0.33428	0.36998	0.39721					
4096	0.15871	0.20712	0.24657	0.28401	0.30692	0.34383	0.37562	0.42638	0.44878	0.50081	0.54507						
2048	0.24682	0.32721	0.38329	0.42679	0.45000	0.49268	0.54202	0.60772	0.62807	0.69494							
1024	0.34758	0.45499	0.50106	0.56851	0.59894	0.64591	0.70630	0.77781	0.80452								
512	0.42736	0.55252	0.61333	0.68991	0.71502	0.77049	0.82734	0.90848									
256	0.48350	0.62492	0.69172	0.75645	0.79725	0.84995	0.91870										
128	0.47025	0.62284	0.69208	0.77702	0.81939	0.87204											
64	0.47704	0.64010	0.71699	0.80063	0.84016												
32	0.46408	0.64092	0.71008	0.78970													
16	0.43619	0.63138	0.70382														
8	0.45349	0.64957															
4	0.51844																

(f) $\mathcal{P}^{\mathcal{C}}$ for Scene: [**Torus**] Algorithm: [**ERPT**] Metric: [**RMSE**] True GT: [**BDPT @ 524288 spp**].

M-ERPT	2	4	8	16	32	64	128	256	512	1024	2048	4096	8192	16384	32768	65536	131072
GT (BDPT)																	
262144	-0.00030	0.00130	0.00042	0.00290	0.00229	0.00327	0.00776	0.00704	0.00757	0.00785	0.01021	0.00913	0.01049	0.01036	0.00899	0.00910	0.00939
131072	0.00266	0.00514	0.00427	0.00534	0.00954	0.01111	0.01757	0.02226	0.02527	0.02471	0.02798	0.02762	0.02759	0.02842	0.02942	0.02878	
65536	0.00509	0.00961	0.01441	0.01855	0.02715	0.03088	0.04123	0.04930	0.05549	0.05644	0.06262	0.06112	0.06098	0.06309	0.06481		
32768	0.01461	0.02357	0.02877	0.03867	0.05410	0.07401	0.08817	0.10018	0.10810	0.11607	0.12942	0.12842	0.12702	0.12679			
16384	0.02848	0.03915	0.06526	0.07442	0.10255	0.13704	0.15850	0.17874	0.20011	0.21138	0.23274	0.23139	0.23138				
8192	0.05346	0.07471	0.11805	0.13774	0.18800	0.23976	0.27883	0.30610	0.34249	0.35450	0.38282	0.37943					
4096	0.09653	0.13288	0.20229	0.23487	0.30498	0.38185	0.42179	0.46395	0.50430	0.52330	0.56069						
2048	0.16401	0.20745	0.31328	0.35388	0.45326	0.55068	0.60377	0.64630	0.69800	0.72077							
1024	0.24563	0.30263	0.43388	0.48508	0.60868	0.71726	0.77273	0.82356	0.87992								
512	0.30821	0.38093	0.52335	0.59178	0.72147	0.83992	0.90064	0.96106									
256	0.36221	0.43380	0.59117	0.66013	0.79616	0.92090	0.98996										
128	0.36450	0.42946	0.59488	0.67855	0.81388	0.93790											
64	0.38297	0.43541	0.59894	0.70102	0.82277												
32	0.37219	0.42928	0.58908	0.68857													
16	0.35397	0.41708	0.57469														
8	0.36131	0.43317															
4	0.41750																

(g) $\mathcal{P}^{\mathcal{C}}$ for Scene: [**Torus**] Algorithm: [**M-ERPT**] Metric: [**RMSE**] True GT: [**BDPT @ 524288 spp**].

Table 27

7.3 Veach Bidir

PT	2	4	8	16	32	64	128	256	512	1024	2048	4096	8192	16384	32768	65536	131072
GT (BDPT)																	
262144	-0.00005	-0.00007	-0.00007	-0.00007	-0.00007	0.00004	-0.00017	-0.00012	-0.00025	-0.00025	-0.00008	-0.00034	0.00020	0.00038	0.00038	0.00076	0.00113
131072	-0.00011	-0.00012	-0.00015	-0.00011	-0.00010	-0.00006	-0.00005	0.00000	0.00001	0.00000	-0.00020	0.00027	0.00019	0.00022	0.00096	0.00166	
65536	-0.00010	-0.00010	-0.00013	-0.00020	-0.00018	-0.00018	-0.00028	-0.00018	0.00011	-0.00001	-0.00032	-0.00025	0.00048	0.00203	0.00251		
32768	-0.00011	-0.00005	-0.00013	-0.00020	-0.00000	-0.00006	-0.00012	-0.00014	0.00029	0.00055	0.00061	0.00117	0.00194	0.00339			
16384	-0.00022	-0.00032	-0.00043	-0.00044	-0.00050	-0.00041	-0.00015	-0.00006	-0.00019	0.00021	0.00056	0.00134	0.00288				
8192	-0.00019	-0.00017	-0.00032	-0.00035	-0.00023	-0.00063	-0.00009	-0.00016	-0.00000	0.00112	0.00199	0.00399					
4096	0.00002	-0.00007	0.00006	0.00008	0.00050	0.00064	0.00139	0.00225	0.00225	0.00278	0.00315						
2048	-0.00026	-0.00017	-0.00029	-0.00019	-0.00038	-0.00036	0.00129	0.00144	0.00159	0.00523							
1024	-0.00047	-0.00057	-0.00099	-0.00143	-0.00130	-0.00157	0.00085	0.00036	0.00046								
512	-0.00051	-0.00086	-0.00127	-0.00100	-0.00082	-0.00256	0.00190	0.00283									
256	-0.00043	-0.00049	-0.00096	-0.00069	0.00247	-0.00104	0.00711										
128	-0.00033	-0.00026	-0.00135	0.00009	0.00398	0.00121											
64	-0.00012	0.00011	0.00018	0.00150	0.00792												
32	0.00081	0.00234	0.00464	0.00892													
16	0.00380	0.00760	0.01302														
8	0.00985	0.01822															
4	0.02038																

(a) $\mathcal{P}^{\mathcal{C}}$ for Scene: [**Veach Bidir**] Algorithm: [**PT**] Metric: [**RMSE**] True GT: [**BDPT @ 524288 spp**].

BDPT	2	4	8	16	32	64	128	256	512	1024	2048	4096	8192	16384	32768	65536	131072
GT (BDPT)																	
262144	-0.00010	-0.00014	-0.00019	-0.00047	-0.00036	-0.00046	-0.00125	0.00128	-0.00043	-0.00984	0.00209	0.03246	0.00400	-0.00477	0.01511	0.02129	0.07172
131072	-0.00015	-0.00021	-0.00014	0.00025	0.00056	0.00031	0.00052	-0.00004	0.00530	0.00922	0.02181	0.01403	0.02753	0.02492	0.03259	0.05533	
65536	-0.00038	-0.00038	-0.00051	-0.00085	-0.00000	0.00013	0.00086	0.00222	-0.00259	0.01104	0.01448	0.03997	0.03237	0.08673	0.09627		
32768	-0.00005	0.00013	0.00024	0.00003	0.00163	0.00453	0.00537	0.00851	0.00233	0.02117	0.02758	0.11674	0.14644	0.16034			
16384	-0.00069	-0.00128	-0.00175	-0.00022	-0.00518	-0.00744	-0.01184	-0.00217	-0.01009	0.01522	0.03691	0.18535	0.15544				
8192	-0.00056	-0.00082	-0.00213	-0.00145	-0.00135	-0.00358	-0.00556	0.01559	0.01712	0.06044	0.11946	0.25648					
4096	0.00054	0.00120	0.00349	0.00581	0.01134	0.01667	0.02899	0.04602	0.07437	0.13285	0.20965						
2048	-0.00039	-0.00079	0.00057	0.00649	0.00010	0.00677	0.01139	0.06625	0.01857	0.15922							
1024	-0.00231	-0.00343	-0.00566	-0.00257	-0.00891	-0.02534	-0.02865	0.05292	0.04410								
512	-0.00318	-0.00538	-0.00787	-0.00535	-0.00600	-0.02435	0.00085	0.12378									
256	-0.00166	-0.00283	-0.00342	0.00738	0.00691	0.00156	0.06565										
128	-0.00047	0.00161	0.00512	0.04198	0.03605	0.01508											
64	0.00554	0.01129	0.02502	0.07709	0.10382												
32	0.01994	0.03866	0.08073	0.16803													
16	0.05078	0.09171	0.16562														
8	0.10012	0.17938															
4	0.18515																

(b) $\mathcal{P}^{\mathcal{C}}$ for Scene: [**Veach Bidir**] Algorithm: [**BDPT**] Metric: [**RMSE**] True GT: [**BDPT @ 524288 spp**].

PSSMLT	2	4	8	16	32	64	128	256	512	1024	2048	4096	8192	16384
GT (BDPT)														
262144	-0.00013	-0.00017	-0.00019	-0.00024	-0.00056	-0.00047	-0.00041	0.00020	-0.00053	-0.00087	-0.00452	-0.00380	-0.00076	-0.00078
131072	-0.00026	-0.00019	-0.00015	0.00006	0.00005	0.00020	0.00047	0.00055	0.00286	0.00315	0.00229	0.00251	0.00434	0.00307
65536	-0.00033	-0.00037	-0.00021	-0.00038	-0.00122	-0.00138	-0.00156	0.00185	-0.00216	0.00160	0.00349	0.00487	0.00705	0.00925
32768	-0.00026	-0.00022	0.00001	0.00028	0.00110	0.00088	0.00145	0.00444	0.00544	0.00986	0.00925	0.01861	0.02204	0.02320
16384	-0.00055	-0.00105	-0.00167	-0.00259	-0.00323	-0.00569	-0.00679	-0.00625	0.01370	0.02106	0.03101	0.03054	0.03522	
8192	-0.00050	-0.00071	-0.00146	-0.00235	-0.00401	-0.00475	-0.00650	-0.00215	0.01630	0.02408	0.04069	0.05040		
4096	0.00045	0.00148	0.00303	0.00558	0.00739	0.01198	0.01892	0.04064	0.06727	0.07076	0.10093			
2048	-0.00037	-0.00054	-0.00042	-0.00017	0.00217	0.00128	0.00249	0.02452	0.09776	0.11277				
1024	-0.00192	-0.00311	-0.00633	-0.01144	-0.01657	-0.02508	-0.03427	-0.00612	0.07588					
512	-0.00203	-0.00387	-0.00750	-0.01390	-0.01761	-0.03205	-0.04226	0.05115						
256	-0.00162	-0.00220	-0.00317	-0.00477	-0.00213	-0.00149	-0.00459							
128	-0.00024	0.00120	0.00400	0.00824	0.01947	0.03162								
64	0.00470	0.00994	0.01895	0.04096	0.07215									
32	0.01965	0.03908	0.06681	0.12578										
16	0.04425	0.08639	0.15103											
8	0.08752	0.16174												
4	0.16349													

(c) $\mathcal{P}^{\mathcal{C}}$ for Scene: [**Veach Bidir**] Algorithm: [**PSSMLT**] Metric: [**RMSE**] True GT: [**BDPT @ 524288 spp**].

MLT	2	4	8	16	32	64	128	256	512	1024	2048	4096	8192	16384
GT (BDPT)														
262144	-0.00008	-0.00008	-0.00009	-0.00009	-0.00012	-0.00031	-0.00046	-0.00044	-0.00049	-0.00049	-0.00170	-0.00195	-0.00168	-0.00154
131072	-0.00023	-0.00027	-0.00033	-0.00033	-0.00037	-0.00059	-0.00049	-0.00037	-0.00003	0.00046	0.00067	0.00054	0.00097	0.00119
65536	-0.00028	-0.00034	-0.00041	-0.00064	-0.00079	-0.00121	-0.00141	-0.00175	-0.00219	-0.00225	-0.00308	-0.00290	-0.00271	-0.00210
32768	-0.00018	-0.00018	-0.00017	-0.00040	-0.00048	-0.00075	-0.00059	0.00044	0.00064	0.00138	0.00118	0.00319	0.00305	0.00365
16384	-0.00030	-0.00041	-0.00061	-0.00105	-0.00151	-0.00231	-0.00300	-0.00388	-0.00601	-0.00838	-0.00655	-0.00920	-0.01020	
8192	-0.00030	-0.00039	-0.00065	-0.00120	-0.00111	-0.00158	-0.00328	-0.00520	-0.00634	-0.00776	-0.00961	-0.00934		
4096	-0.00023	-0.00024	0.00008	0.00055	0.00086	0.00246	0.00601	0.00873	0.01473	0.02164	0.02815			
2048	-0.00049	-0.00073	-0.00107	-0.00081	-0.00081	-0.00045	0.00008	0.00161	0.00276	0.00068				
1024	-0.00061	-0.00135	-0.00226	-0.00370	-0.00357	-0.00678	-0.01237	-0.02338	-0.03222					
512	-0.00117	-0.00197	-0.00353	-0.00449	-0.00404	-0.00678	-0.01211	-0.02084						
256	-0.00072	-0.00126	-0.00261	-0.00395	-0.00266	-0.00254	-0.00493							
128	-0.00079	-0.00127	-0.00113	0.00033	0.00484	0.00895								
64	-0.00043	0.00001	0.00119	0.00607	0.01812									
32	0.00142	0.00356	0.00838	0.02281										
16	0.00558	0.01290	0.02727											
8	0.01229	0.02669												
4	0.02387													

(d) $\mathcal{P}^{\mathcal{C}}$ for Scene: [**Veach Bidir**] Algorithm: [**MLT**] Metric: [**RMSE**] True GT: [**BDPT @ 524288 spp**].

M-MLT	2	4	8	16	32	64	128	256	512	1024	2048	4096	8192	16384
GT (BDPT)														
262144	-0.00012	-0.00014	-0.00020	-0.00019	-0.00037	-0.00031	-0.00039	-0.00055	-0.00080	-0.00048	-0.00053	-0.00079	-0.00079	-0.00150
131072	-0.00024	-0.00027	-0.00033	-0.00038	-0.00052	-0.00036	-0.00043	-0.00015	-0.00020	0.00045	0.00145	0.00069	0.00150	0.00134
65536	-0.00025	-0.00028	-0.00041	-0.00036	-0.00089	-0.00066	-0.00117	-0.00151	-0.00172	-0.00102	-0.00055	-0.00173	-0.00120	-0.00167
32768	-0.00024	-0.00021	-0.00028	-0.00006	-0.00032	-0.00003	0.00001	0.00049	0.00141	0.00423	0.00273	0.00424	0.00434	0.00426
16384	-0.00032	-0.00039	-0.00048	-0.00117	-0.00133	-0.00216	-0.00315	-0.00451	-0.00598	-0.00892	-0.00840	-0.01164	-0.01261	
8192	-0.00041	-0.00076	-0.00086	-0.00129	-0.00187	-0.00262	-0.00293	-0.00368	-0.00659	-0.00533	-0.00477	-0.00772		
4096	-0.00014	0.00017	0.00025	0.00096	0.00191	0.00328	0.00631	0.00944	0.01596	0.02270	0.02839			
2048	-0.00023	-0.00009	-0.00016	-0.00052	-0.00114	0.00015	0.00163	-0.00008	0.00153	0.00526				
1024	-0.00068	-0.00144	-0.00129	-0.00321	-0.00538	-0.01006	-0.01371	-0.02339	-0.03204					
512	-0.00083	-0.00190	-0.00185	-0.00450	-0.00705	-0.01143	-0.01504	-0.02530						
256	-0.00077	-0.00128	-0.00104	-0.00337	-0.00443	-0.00952	-0.00867							
128	-0.00063	-0.00155	-0.00069	-0.00200	-0.00048	0.00084								
64	-0.00048	-0.00120	0.00163	0.00206	0.00917									
32	0.00126	0.00324	0.00899	0.01998										
16	0.00491	0.01130	0.02699											
8	0.01242	0.02673												
4	0.02445													

(e) $\mathcal{P}^{\mathcal{C}}$ for Scene: [**Veach Bidir**] Algorithm: [**M-MLT**] Metric: [**RMSE**] True GT: [**BDPT @ 524288 spp**].

ERPT	2	4	8	16	32	64	128	256	512	1024	2048	4096	8192	16384	32768	65536	131072
GT (BDPT)																	
262144	-0.00028	-0.00022	-0.00055	-0.00034	-0.00063	-0.00044	-0.00091	-0.00021	-0.00052	0.00027	0.00008	0.00035	-0.00042	0.00046	0.00007	-0.00040	-0.00051
131072	0.00004	-0.00022	0.00017	0.00013	-0.00028	-0.00053	0.00036	-0.00031	0.00022	-0.00016	-0.00029	0.00005	-0.00011	0.00017	-0.00014	-0.00049	
65536	-0.00031	-0.00090	-0.00038	-0.00060	-0.00035	-0.00061	-0.00006	0.00004	0.00165	0.00063	0.00165	0.00088	0.00106	0.00099	0.00060		
32768	0.00088	0.00084	0.00077	0.00004	0.00051	0.00169	0.00101	0.00069	0.00179	0.00233	0.00223	0.00217	0.00190	0.00183			
16384	-0.00237	-0.00322	-0.00385	-0.00319	-0.00398	0.00208	-0.00136	0.00454	0.00472	0.00288	0.00330	0.00484	0.00354				
8192	-0.00344	-0.00306	-0.00284	-0.00220	-0.00105	0.00646	0.00098	0.00087	0.00439	0.00545	0.00610	0.00643					
4096	0.00923	0.00685	0.00911	0.00723	0.00864	0.00592	0.00778	0.00659	0.01429	0.00922	0.00995						
2048	0.00196	0.00216	-0.00021	0.00057	-0.00042	0.01079	0.00606	0.01524	0.01821	0.01725							
1024	-0.01384	-0.01465	-0.01559	-0.01481	-0.00626	0.02159	-0.00282	0.01439	0.02436								
512	-0.01543	-0.01940	-0.01815	-0.01673	0.00145	0.04621	0.01137	0.03667									
256	-0.00429	-0.00610	-0.00359	-0.00301	0.02171	0.06909	0.04592										
128	0.00987	0.01099	0.01238	0.00970	0.04256	0.12634											
64	0.05882	0.05491	0.06273	0.05988	0.10072												
32	0.16889	0.17951	0.18302	0.18435													
16	0.34123	0.33955	0.35177														
8	0.54665	0.55468															
4	0.80189																

(f) $\mathcal{P}^{\mathcal{C}}$ for Scene: [**Veach Bidir**] Algorithm: [**ERPT**] Metric: [**RMSE**] True GT: [**BDPT @ 524288 spp**].

M-ERPT	2	4	8	16	32	64	128	256	512	1024	2048	4096	8192	16384	32768	65536	131072
GT (BDPT)																	
262144	-0.00013	-0.00061	-0.00046	-0.00060	-0.00023	-0.00020	0.00058	-0.00011	0.00060	0.00011	-0.00009	-0.00033	-0.00004	-0.00004	-0.00001	0.00002	-0.00080
131072	0.00010	-0.00002	-0.00013	0.00023	-0.00002	0.00016	0.00025	0.00010	0.00043	0.00005	-0.00013	-0.00014	-0.00038	-0.00028	-0.00029	-0.00021	
65536	-0.00049	-0.00097	-0.00010	-0.00045	0.00020	0.00015	0.00108	0.00055	0.00095	0.00033	0.00089	0.00066	0.00065	0.00067	0.00009		
32768	0.00034	0.00049	0.00073	0.00051	0.00123	0.00135	0.00236	0.00099	0.00129	0.00139	0.00169	0.00192	0.00195	0.00130			
16384	-0.00364	-0.00286	0.00151	0.00158	0.00028	0.00223	0.00262	0.00307	0.00222	0.00416	0.00373	0.00367	0.00387				
8192	-0.00236	-0.00315	0.00350	0.00279	0.00139	0.00349	0.00833	0.00352	0.00572	0.00560	0.00659	0.00584					
4096	0.00798	0.00651	0.01132	0.01047	0.00844	0.01150	0.00987	0.01138	0.01027	0.01018	0.01011						
2048	-0.00263	0.00031	0.01436	0.01472	0.01216	0.01652	0.01193	0.01572	0.01080	0.01889							
1024	-0.01431	-0.01810	0.02269	0.01394	0.00855	0.02183	0.02901	0.02363	0.02504								
512	-0.01784	-0.01583	0.05290	0.03690	0.03101	0.05223	0.05243	0.05467									
256	-0.00805	-0.00959	0.07032	0.06621	0.05413	0.07503	0.07419										
128	0.00531	0.00129	0.13434	0.11966	0.10977	0.13390											
64	0.04908	0.05110	0.19880	0.18534	0.17842												
32	0.15157	0.16020	0.28669	0.27264													
16	0.31843	0.31806	0.42895														
8	0.51444	0.52160															
4	0.76535																

(g) $\mathcal{P}^{\mathcal{C}}$ for Scene: [**Veach Bidir**] Algorithm: [**M-ERPT**] Metric: [**RMSE**] True GT: [**BDPT @ 524288 spp**].

Table 28

7.4 Veach Door

PT	2	4	8	16	32	64	128	256	512	1024	2048	4096	8192	16384	32768	65536	131072
GT (BDPT)																	
262144	-0.00004	-0.00001	0.00000	-0.00001	0.00005	0.00000	0.00007	0.00014	0.00013	0.00033	0.00037	0.00059	0.00123	0.00233	0.00401	0.00961	0.01437
131072	-0.00007	0.00001	0.00001	0.00004	0.00012	0.00004	0.00020	0.00037	0.00045	0.00061	0.00101	0.00199	0.00312	0.00588	0.01238	0.02134	
65536	-0.00003	-0.00001	-0.00001	-0.00004	0.00012	0.00019	0.00026	0.00061	0.00087	0.00136	0.00253	0.00397	0.00752	0.01466	0.02645		
32768	0.00007	0.00010	0.00002	0.00011	0.00025	0.00029	0.00078	0.00145	0.00159	0.00269	0.00476	0.00944	0.01617	0.03119			
16384	0.00016	0.00014	0.00011	0.00020	0.00028	0.00045	0.00109	0.00180	0.00348	0.00543	0.00913	0.01756	0.03297				
8192	0.00042	0.00026	0.00019	0.00017	0.00040	0.00111	0.00210	0.00315	0.00605	0.01118	0.01941	0.03582					
4096	0.00075	0.00056	0.00044	0.00091	0.00156	0.00272	0.00385	0.00754	0.01234	0.02332	0.03903						
2048	0.00151	0.00102	0.00061	0.00191	0.00279	0.00514	0.00807	0.01472	0.02528	0.04260							
1024	0.00315	0.00210	0.00210	0.00331	0.00599	0.01076	0.01803	0.03156	0.04949								
512	0.00562	0.00445	0.00429	0.00651	0.01237	0.02108	0.03465	0.05885									
256	0.01004	0.00758	0.00858	0.01266	0.02489	0.04347	0.06828										
128	0.01637	0.01424	0.01688	0.02706	0.04879	0.08217											
64	0.02700	0.02634	0.03013	0.05291	0.09396												
32	0.04378	0.04549	0.06128	0.10135													
16	0.07175	0.07872	0.11343														
8	0.11844	0.13970															
4	0.18240																

(a) $\mathcal{P}^{\mathcal{C}}$ for Scene: [**Veach Door**] Algorithm: [**PT**] Metric: [**RMSE**] True GT: [**BDPT @ 524288 spp**].

BDPT	2	4	8	16	32	64	128	256	512	1024	2048	4096	8192	16384	32768	65536	131072
GT (BDPT)																	
262144	-0.00002	-0.00002	0.00001	-0.00001	0.00005	0.00010	0.00009	0.00003	0.00075	0.00094	0.00248	0.00426	0.00756	0.01516	0.02909	0.04810	0.07432
131072	-0.00001	0.00001	0.00001	0.00009	0.00012	0.00017	0.00056	0.00096	0.00207	0.00324	0.00622	0.01033	0.02174	0.04160	0.07062	0.11974	
65536	-0.00003	-0.00001	0.00004	0.00016	0.00036	0.00042	0.00099	0.00173	0.00332	0.00662	0.01361	0.02653	0.04960	0.09109	0.15684		
32768	0.00002	0.00005	0.00005	0.00039	0.00057	0.00086	0.00179	0.00390	0.00869	0.01527	0.03016	0.05494	0.10614	0.18451			
16384	0.00010	0.00010	0.00017	0.00028	0.00071	0.00176	0.00386	0.00677	0.01504	0.02834	0.05435	0.10461	0.18746				
8192	0.00027	0.00012	0.00014	0.00067	0.00140	0.00358	0.00717	0.01469	0.02945	0.05680	0.10760	0.19737					
4096	0.00034	0.00059	0.00081	0.00168	0.00324	0.00754	0.01494	0.02891	0.05878	0.10861	0.20158						
2048	0.00090	0.00106	0.00154	0.00307	0.00633	0.01438	0.02870	0.05674	0.10593	0.19666							
1024	0.00197	0.00219	0.00323	0.00651	0.01391	0.02909	0.05763	0.10874	0.19745								
512	0.00419	0.00397	0.00724	0.01329	0.02709	0.05682	0.10884	0.19627									
256	0.00810	0.00857	0.01314	0.02638	0.05218	0.10498	0.18923										
128	0.01474	0.01670	0.02788	0.05461	0.10347	0.19207											
64	0.02509	0.03230	0.05634	0.10427	0.18945												
32	0.04551	0.06088	0.10446	0.18976													
16	0.08336	0.11920	0.19143														
8	0.14414	0.20618															
4	0.23498																

(b) $\mathcal{P}^{\mathcal{C}}$ for Scene: [**Veach Door**] Algorithm: [**BDPT**] Metric: [**RMSE**] True GT: [**BDPT @ 524288 spp**].

PSSMLT	2	4	8	16	32	64	128	256	512	1024	2048	4096	8192	16384
GT (BDPT)														
262144	0.00000	0.00000	-0.00002	-0.00001	0.00004	0.00017	0.00051	0.00080	0.00137	0.00274	0.00450	0.00792	0.01332	0.02211
131072	0.00002	0.00004	0.00016	0.00015	0.00042	0.00061	0.00110	0.00189	0.00301	0.00565	0.01169	0.02120	0.03583	0.05435
65536	0.00005	0.00010	0.00019	0.00017	0.00052	0.00091	0.00175	0.00345	0.00674	0.01322	0.02564	0.04534	0.07534	0.11636
32768	0.00009	0.00019	0.00027	0.00060	0.00128	0.00256	0.00458	0.00864	0.01642	0.02995	0.05448	0.09455	0.15245	0.22051
16384	0.00006	0.00013	0.00033	0.00091	0.00196	0.00386	0.00799	0.01456	0.02843	0.05371	0.09885	0.16964	0.26449	
8192	0.00029	0.00050	0.00091	0.00187	0.00398	0.00808	0.01581	0.03035	0.05726	0.10480	0.18471	0.29956		
4096	0.00060	0.00082	0.00166	0.00389	0.00850	0.01604	0.03115	0.05977	0.10906	0.19757	0.31967			
2048	0.00093	0.00169	0.00395	0.00852	0.01590	0.03169	0.06070	0.10886	0.19444	0.32752				
1024	0.00211	0.00430	0.00852	0.01711	0.03341	0.06361	0.11467	0.20349	0.33657					
512	0.00446	0.00870	0.01766	0.03463	0.06439	0.11948	0.20692	0.34405						
256	0.00991	0.01841	0.03449	0.06681	0.12075	0.21136	0.34466							
128	0.01991	0.03710	0.06801	0.12348	0.21415	0.35653								
64	0.04112	0.07198	0.12881	0.22213	0.36087									
32	0.07479	0.13617	0.23311	0.37189										
16	0.14117	0.23938	0.38701											
8	0.24435	0.39282												
4	0.39171													

(c) $\mathcal{P}^{\mathcal{C}}$ for Scene: [**Veach Door**] Algorithm: [**PSSMLT**] Metric: [**RMSE**] True GT: [**BDPT @ 524288 spp**].

MLT	2	4	8	16	32	64	128	256	512	1024	2048	4096	8192	16384
GT (BDPT)														
262144	-0.00004	-0.00003	0.00002	0.00008	0.00009	0.00028	0.00037	0.00118	0.00125	0.00250	0.00502	0.00966	0.01612	0.02175
131072	-0.00003	0.00007	0.00019	0.00019	0.00040	0.00070	0.00123	0.00224	0.00372	0.00681	0.01478	0.02469	0.03629	0.05694
65536	0.00002	0.00005	0.00030	0.00032	0.00076	0.00178	0.00259	0.00498	0.00893	0.01592	0.02922	0.05369	0.08041	0.12090
32768	-0.00003	0.00022	0.00044	0.00044	0.00144	0.00263	0.00511	0.00952	0.01775	0.03236	0.06086	0.10844	0.16269	0.22779
16384	0.00003	0.00022	0.00055	0.00111	0.00234	0.00456	0.00963	0.01947	0.03569	0.06514	0.12056	0.19586	0.28088	
8192	0.00003	0.00030	0.00085	0.00223	0.00490	0.00918	0.02050	0.03825	0.06976	0.12884	0.21637	0.34021		
4096	0.00031	0.00117	0.00244	0.00458	0.01003	0.01923	0.03825	0.07692	0.13259	0.22649	0.36948			
2048	0.00080	0.00174	0.00405	0.00960	0.01851	0.03760	0.07113	0.13367	0.23352	0.37505				
1024	0.00205	0.00449	0.01005	0.02019	0.03997	0.07589	0.13593	0.24280	0.38809					
512	0.00422	0.00922	0.01946	0.04098	0.07568	0.13744	0.24440	0.40261						
256	0.00875	0.01852	0.03977	0.07704	0.13778	0.23978	0.39503							
128	0.01686	0.03825	0.07847	0.14460	0.24699	0.40087								
64	0.03891	0.07444	0.14905	0.25605	0.41004									
32	0.07391	0.14284	0.26307	0.42282										
16	0.13867	0.25403	0.43443											
8	0.24770	0.41751												
4	0.39793													

(d) $\mathcal{P}^{\mathcal{C}}$ for Scene: [**Veach Door**] Algorithm: [**MLT**] Metric: [**RMSE**] True GT: [**BDPT @ 524288 spp**].

M-MLT	2	4	8	16	32	64	128	256	512	1024	2048	4096	8192	16384
GT (BDPT)														
262144	-0.00000	0.00000	0.00008	-0.00000	-0.00019	0.00023	0.00040	0.00095	0.00207	0.00230	0.00554	0.00846	0.01353	0.02038
131072	0.00004	0.00004	0.00013	0.00021	0.00033	0.00092	0.00156	0.00207	0.00440	0.00673	0.01438	0.02138	0.03744	0.04813
65536	0.00007	0.00005	0.00025	0.00027	0.00052	0.00176	0.00254	0.00549	0.00932	0.01629	0.03110	0.04642	0.08429	0.10804
32768	0.00008	0.00020	0.00055	0.00055	0.00142	0.00265	0.00583	0.00932	0.01850	0.03284	0.06468	0.09558	0.16125	0.20494
16384	0.00012	0.00015	0.00058	0.00120	0.00292	0.00455	0.00913	0.01870	0.03545	0.06321	0.11741	0.17208	0.27840	
8192	0.00010	0.00044	0.00101	0.00247	0.00509	0.01012	0.01791	0.03675	0.06953	0.12095	0.21678	0.30543		
4096	0.00077	0.00118	0.00251	0.00502	0.01089	0.01969	0.04249	0.07325	0.13132	0.22086	0.36824			
2048	0.00109	0.00186	0.00501	0.00901	0.01989	0.03856	0.07548	0.13529	0.23101	0.36754				
1024	0.00236	0.00502	0.01123	0.01993	0.04261	0.07745	0.13964	0.23975	0.39074					
512	0.00477	0.00953	0.02107	0.04061	0.07933	0.14320	0.24971	0.39874						
256	0.01054	0.02029	0.04173	0.07972	0.14556	0.24851	0.40221							
128	0.02157	0.04182	0.08158	0.14872	0.25633	0.41106								
64	0.04185	0.08089	0.15447	0.26248	0.42314									
32	0.08419	0.15198	0.27546	0.43235										
16	0.15447	0.26544	0.44863											
8	0.26711	0.43325												
4	0.42239													

(e) $\mathcal{P}^{\mathcal{C}}$ for Scene: [**Veach Door**] Algorithm: [**M-MLT**] Metric: [**RMSE**] True GT: [**BDPT @ 524288 spp**].

ERPT	2	4	8	16	32	64	128	256	512	1024	2048	4096	8192	16384	32768	65536	131072
GT (BDPT)																	
262144	0.00028	0.00053	0.00061	0.00030	0.00073	0.00050	0.00098	0.00067	0.00083	0.00056	0.00073	0.00065	0.00097	0.00062	0.00101	0.00077	0.00055
131072	0.00161	0.00158	0.00196	0.00181	0.00216	0.00202	0.00243	0.00212	0.00230	0.00210	0.00180	0.00213	0.00242	0.00146	0.00162	0.00216	
65536	0.00305	0.00318	0.00432	0.00380	0.00379	0.00430	0.00410	0.00430	0.00420	0.00395	0.00338	0.00441	0.00399	0.00374	0.00395		
32768	0.00652	0.00650	0.00821	0.00795	0.00886	0.00790	0.00809	0.00771	0.00831	0.00830	0.00727	0.00814	0.00849	0.00773			
16384	0.01031	0.01222	0.01324	0.01468	0.01537	0.01540	0.01457	0.01529	0.01365	0.01596	0.01499	0.01563	0.01472				
8192	0.02087	0.02077	0.02736	0.02929	0.02853	0.03063	0.02996	0.03118	0.02770	0.03105	0.02986	0.02953					
4096	0.04421	0.04703	0.05237	0.05708	0.05647	0.05837	0.05730	0.06006	0.05726	0.05837	0.05910						
2048	0.08368	0.08548	0.09954	0.10718	0.10711	0.10727	0.10540	0.10901	0.10632	0.10793							
1024	0.15383	0.16228	0.18169	0.19588	0.19383	0.19888	0.19347	0.19732	0.19201								
512	0.26946	0.27956	0.31600	0.32953	0.33122	0.33547	0.32973	0.33827									
256	0.43031	0.44341	0.48593	0.51265	0.51420	0.51911	0.51699										
128	0.64858	0.67147	0.72397	0.75322	0.75514	0.75776											
64	0.90785	0.92763	0.98797	1.02104	1.02155												
32	1.19658	1.22010	1.28269	1.31738													
16	1.50088	1.52666	1.58891														
8	1.81169	1.83891															
4	2.09867																

(f) $\mathcal{P}^{\mathcal{C}}$ for Scene: [**Veach Door**] Algorithm: [**ERPT**] Metric: [**RMSE**] True GT: [**BDPT @ 524288 spp**].

M-ERPT	2	4	8	16	32	64	128	256	512	1024	2048	4096	8192	16384	32768	65536	131072
GT (BDPT)																	
262144	0.00015	0.00052	0.00056	0.00050	0.00068	0.00062	0.00078	0.00048	0.00063	0.00093	0.00025	0.00051	0.00064	0.00042	0.00084	0.00073	0.00053
131072	0.00114	0.00123	0.00176	0.00160	0.00136	0.00176	0.00182	0.00170	0.00151	0.00204	0.00159	0.00187	0.00201	0.00149	0.00156	0.00191	
65536	0.00288	0.00320	0.00300	0.00334	0.00377	0.00362	0.00420	0.00385	0.00331	0.00344	0.00318	0.00385	0.00430	0.00327	0.00385		
32768	0.00487	0.00633	0.00609	0.00662	0.00789	0.00712	0.00697	0.00679	0.00765	0.00671	0.00706	0.00717	0.00736	0.00703			
16384	0.00891	0.01123	0.01181	0.01341	0.01300	0.01381	0.01323	0.01318	0.01234	0.01318	0.01308	0.01395	0.01386				
8192	0.01870	0.02218	0.02363	0.02491	0.02567	0.02468	0.02562	0.02700	0.02576	0.02563	0.02760	0.02661					
4096	0.03833	0.04578	0.04948	0.04841	0.05142	0.04953	0.05186	0.05191	0.05335	0.05229	0.05098						
2048	0.07409	0.08668	0.09310	0.09185	0.09427	0.09490	0.09637	0.09828	0.09739	0.09607							
1024	0.13789	0.15967	0.17214	0.17045	0.17671	0.17525	0.18007	0.18151	0.17799								
512	0.24323	0.27538	0.28859	0.29526	0.30005	0.30011	0.30634	0.30736									
256	0.39755	0.44133	0.46138	0.46943	0.47349	0.47244	0.48016										
128	0.60614	0.65927	0.68776	0.69940	0.70768	0.70094											
64	0.85512	0.92132	0.94745	0.96319	0.97107												
32	1.14148	1.21124	1.24094	1.25649													
16	1.44174	1.51393	1.54907														
8	1.75092	1.82692															
4	2.03753																

(g) $\mathcal{P}^{\mathcal{C}}$ for Scene: [**Veach Door**] Algorithm: [**M-ERPT**] Metric: [**RMSE**] True GT: [**BDPT @ 524288 spp**].

Table 29

7.5 Sponza

PT	2	4	8	16	32	64	128	256	512	1024	2048	4096	8192
GT (PT)													
16384	0.00007	0.00007	0.00013	0.00038	0.00051	0.00096	0.00136	0.00480	0.00766	0.01424	0.02898	0.05231	0.08664
8192	0.00003	0.00020	0.00031	0.00061	0.00180	0.00305	0.00495	0.01164	0.02391	0.04311	0.07967	0.13979	
4096	0.00025	0.00058	0.00104	0.00184	0.00390	0.00700	0.01258	0.02700	0.05276	0.09437	0.17109		
2048	0.00049	0.00089	0.00168	0.00315	0.00755	0.01406	0.02694	0.05535	0.10221	0.18413			
1024	0.00114	0.00235	0.00377	0.00761	0.01521	0.03023	0.05724	0.10698	0.19340				
512	0.00266	0.00482	0.00799	0.01577	0.03000	0.05708	0.11039	0.19633					
256	0.00470	0.00847	0.01508	0.03001	0.05899	0.10923	0.19688						
128	0.00951	0.01630	0.03040	0.05840	0.11183	0.20259							
64	0.01792	0.03195	0.05968	0.11225	0.20232								
32	0.03500	0.06141	0.10838	0.19843									
16	0.06544	0.11336	0.19475										
8	0.11529	0.19639											
4	0.18764												

(a) $\mathcal{P}^{\mathcal{C}}$ for Scene: [**Sponza**] Algorithm: [**PT**] Metric: [**RMSE**] True GT: [**PT @ 32768 spp**].

BDPT	2	4	8	16	32	64	128	256	512	1024	2048	4096	8192
GT (PT)													
16384	-0.00007	-0.00006	0.00008	0.00016	0.00033	0.00051	0.00141	0.00280	0.00644	0.01339	0.02180	0.04045	0.07194
8192	0.00007	0.00009	0.00033	0.00047	0.00121	0.00245	0.00431	0.00910	0.01814	0.03628	0.06364	0.11555	
4096	0.00013	0.00024	0.00075	0.00133	0.00310	0.00525	0.01066	0.02088	0.04053	0.07979	0.14373		
2048	0.00053	0.00084	0.00165	0.00282	0.00576	0.01121	0.02284	0.04452	0.08465	0.15619			
1024	0.00086	0.00119	0.00344	0.00584	0.01183	0.02298	0.04445	0.08592	0.16121				
512	0.00163	0.00377	0.00590	0.01266	0.02331	0.04536	0.08928	0.16470					
256	0.00355	0.00647	0.01204	0.02315	0.04575	0.08470	0.16454						
128	0.00750	0.01312	0.02486	0.04427	0.08775	0.16455							
64	0.01331	0.02517	0.04637	0.08628	0.16140								
32	0.02665	0.05083	0.08713	0.15810									
16	0.05263	0.08777	0.15811										
8	0.09436	0.15761											
4	0.15550												

(b) $\mathcal{P}^{\mathcal{C}}$ for Scene: [**Sponza**] Algorithm: [**BDPT**] Metric: [**RMSE**] True GT: [**PT @ 32768 spp**].

PSSMLT	2	4	8	16	32	64	128	256	512	1024	2048	4096	8192
GT (PT)													
16384	0.00007	0.00018	0.00012	0.00025	0.00038	0.00047	0.00129	0.00198	0.00374	0.00508	0.00599	0.00734	0.00767
8192	0.00026	0.00034	0.00062	0.00117	0.00150	0.00230	0.00461	0.00671	0.01081	0.01430	0.01801	0.02167	
4096	0.00036	0.00075	0.00122	0.00205	0.00369	0.00597	0.01064	0.01659	0.02524	0.03305	0.04062		
2048	0.00078	0.00138	0.00232	0.00416	0.00745	0.01191	0.02155	0.03496	0.05219	0.07033			
1024	0.00156	0.00255	0.00479	0.00881	0.01448	0.02564	0.04389	0.07007	0.10422				
512	0.00372	0.00631	0.01041	0.01693	0.03084	0.05101	0.08558	0.13472					
256	0.00687	0.01141	0.01982	0.03426	0.05990	0.09889	0.15945						
128	0.01480	0.02350	0.03920	0.06658	0.11474	0.18281							
64	0.02712	0.04489	0.07681	0.12730	0.20605								
32	0.05115	0.08299	0.13594	0.22170									
16	0.09728	0.15407	0.24355										
8	0.16800	0.25560											
4	0.26775												

(c) $\mathcal{P}^{\mathcal{C}}$ for Scene: [**Sponza**] Algorithm: [**PSSMLT**] Metric: [**RMSE**] True GT: [**PT @ 32768 spp**].

MLT	2	4	8	16	32	64	128	256	512	1024	2048	4096	8192
GT (PT)													
16384	0.00013	0.00023	0.00027	0.00044	0.00105	0.00141	0.00229	0.00418	0.00532	0.00653	0.00802	0.00796	0.00753
8192	0.00018	0.00028	0.00113	0.00161	0.00299	0.00481	0.00958	0.01224	0.01693	0.01979	0.02305	0.02214	
4096	0.00064	0.00125	0.00248	0.00418	0.00756	0.01220	0.02005	0.02906	0.03790	0.04591	0.04906		
2048	0.00117	0.00228	0.00451	0.00810	0.01626	0.02721	0.04309	0.06066	0.07915	0.09227			
1024	0.00234	0.00501	0.00889	0.01753	0.03357	0.05689	0.08775	0.12047	0.15342				
512	0.00492	0.00974	0.01819	0.03462	0.06501	0.10527	0.16325	0.21954					
256	0.00911	0.01943	0.03590	0.07103	0.12309	0.19750	0.28420						
128	0.01819	0.03623	0.07032	0.12841	0.22158	0.34038							
64	0.03354	0.06893	0.12970	0.22714	0.36604								
32	0.06414	0.12480	0.23018	0.37669									
16	0.11600	0.22200	0.37645										
8	0.19951	0.35887											
4	0.31540												

(d) $\mathcal{P}^{\mathcal{C}}$ for Scene: [**Sponza**] Algorithm: [**MLT**] Metric: [**RMSE**] True GT: [**PT @ 32768 spp**].

M-MLT	2	4	8	16	32	64	128	256	512	1024	2048	4096	8192
GT (PT)													
16384	0.00020	0.00015	0.00044	0.00031	0.00114	0.00143	0.00307	0.00465	0.00543	0.00694	0.00715	0.00755	0.00787
8192	0.00010	0.00036	0.00086	0.00158	0.00352	0.00576	0.00938	0.01306	0.01743	0.01914	0.02092	0.02163	
4096	0.00074	0.00096	0.00198	0.00383	0.00808	0.01387	0.02062	0.03127	0.03808	0.04556	0.04823		
2048	0.00124	0.00204	0.00461	0.00909	0.01649	0.02767	0.04489	0.06327	0.07937	0.09115			
1024	0.00306	0.00447	0.00932	0.01803	0.03240	0.05737	0.08770	0.12359	0.15276				
512	0.00589	0.00921	0.01779	0.03546	0.06540	0.10731	0.16742	0.22587					
256	0.01195	0.01695	0.03600	0.06975	0.12242	0.19715	0.29102						
128	0.02311	0.03508	0.07004	0.13174	0.22333	0.33932							
64	0.04374	0.06655	0.13090	0.23334	0.37505								
32	0.08030	0.12329	0.22810	0.38513									
16	0.14738	0.21930	0.38155										
8	0.24563	0.35493											
4	0.37712												

(e) $\mathcal{P}^{\mathcal{C}}$ for Scene: [**Sponza**] Algorithm: [**M-MLT**] Metric: [**RMSE**] True GT: [**PT @ 32768 spp**].

ERPT	2	4	8	16	32	64	128	256	512	1024	2048	4096	8192
GT (PT)													
16384	0.00415	0.00376	0.00322	0.00288	0.00383	0.00401	0.00425	0.00367	0.00374	0.00389	0.00437	0.00366	0.00337
8192	0.01035	0.01055	0.01087	0.01032	0.01138	0.01130	0.01088	0.01046	0.01078	0.01007	0.01073	0.01126	
4096	0.02303	0.02346	0.02383	0.02490	0.02550	0.02614	0.02480	0.02485	0.02477	0.02552	0.02461		
2048	0.04720	0.05002	0.05090	0.05098	0.05159	0.05300	0.05216	0.05281	0.05363	0.05140			
1024	0.09478	0.09960	0.10195	0.10132	0.10426	0.10424	0.10408	0.10110	0.10214				
512	0.17690	0.18464	0.18554	0.18845	0.18949	0.19292	0.19030	0.19070					
256	0.30547	0.31614	0.32270	0.32557	0.32818	0.32790	0.32755						
128	0.49866	0.51338	0.51943	0.52583	0.52658	0.52803							
64	0.73484	0.75391	0.76321	0.76909	0.77025								
32	1.01315	1.03086	1.03835	1.04708									
16	1.30976	1.33261	1.33988										
8	1.60448	1.62656											
4	1.87794												

(f) $\mathcal{P}^{\mathcal{C}}$ for Scene: [**Sponza**] Algorithm: [**ERPT**] Metric: [**RMSE**] True GT: [**PT @ 32768 spp**].

M-ERPT	2	4	8	16	32	64	128	256	512	1024	2048	4096	8192
GT (PT)													
16384	0.00333	0.00332	0.00313	0.00370	0.00393	0.00292	0.00327	0.00294	0.00351	0.00290	0.00366	0.00319	0.00304
8192	0.00961	0.01082	0.01016	0.01052	0.01044	0.01049	0.00987	0.00998	0.00982	0.01000	0.01027	0.01004	
4096	0.02280	0.02388	0.02416	0.02402	0.02488	0.02325	0.02395	0.02288	0.02230	0.02334	0.02340		
2048	0.04631	0.04792	0.04923	0.04797	0.05137	0.04930	0.04742	0.04878	0.05028	0.04856			
1024	0.09385	0.09223	0.09709	0.09593	0.09873	0.09747	0.09889	0.10076	0.09897				
512	0.17037	0.17681	0.17890	0.17972	0.18515	0.18286	0.18054	0.18139					
256	0.29422	0.30820	0.31130	0.31037	0.31967	0.31658	0.31002						
128	0.48522	0.50050	0.50288	0.50066	0.51463	0.51039							
64	0.72040	0.73457	0.74151	0.74544	0.75382								
32	0.99377	1.01268	1.01962	1.02246									
16	1.29075	1.31091	1.31951										
8	1.58518	1.60487											
4	1.85930												

(g) $\mathcal{P}^{\mathcal{C}}$ for Scene: [**Sponza**] Algorithm: [**M-ERPT**] Metric: [**RMSE**] True GT: [**PT @ 32768 spp**].

Table 30

8 SC-DM

8.1 Cornell Box

PT	2	4	8	16	32	64	128	256	512	1024	2048	4096	8192
**GT (PT)**													
16384	-0.00362	-0.00457	-0.00617	-0.00862	-0.01044	-0.01390	-0.01771	-0.01980	-0.02340	-0.02488	-0.01314	0.01483	0.09444
8192	-0.00975	-0.01247	-0.01637	-0.02131	-0.02893	-0.03604	-0.04276	-0.05711	-0.06048	-0.05611	-0.02575	0.08521	
4096	-0.01969	-0.02471	-0.03185	-0.04190	-0.05409	-0.06992	-0.08439	-0.09717	-0.09727	-0.06397	0.04795		
2048	-0.03268	-0.04100	-0.05380	-0.06939	-0.09045	-0.11407	-0.12998	-0.14406	-0.12048	-0.01400			
1024	-0.05405	-0.06604	-0.08709	-0.11084	-0.13784	-0.16570	-0.18164	-0.16675	-0.06611				
512	-0.08217	-0.09999	-0.13143	-0.15881	-0.19760	-0.22331	-0.22428	-0.13564					
256	-0.12217	-0.14521	-0.18796	-0.22111	-0.25533	-0.25039	-0.15876						
128	-0.17234	-0.20580	-0.25272	-0.27690	-0.28435	-0.21756							
64	-0.23949	-0.27394	-0.31142	-0.33038	-0.24849								
32	-0.30058	-0.32792	-0.35182	-0.27831									
16	-0.36486	-0.36836	-0.31919										
8	-0.40499	-0.33882											
4	-0.37472												

(a) $\mathcal{P}^{\mathcal{C}}$ for Scene: [**Cornell Box**] Algorithm: [**PT**] Metric: [**SC-DM**] True GT: [**PT @ 32768 spp**].

BDPT	2	4	8	16	32	64	128	256	512	1024	2048	4096	8192
**GT (PT)**													
16384	-0.00424	-0.00593	-0.00774	-0.00947	-0.01361	-0.01586	-0.01967	-0.02432	-0.01046	0.00988	0.05509	0.12629	0.23848
8192	-0.01189	-0.01561	-0.02039	-0.02663	-0.03445	-0.04589	-0.05106	-0.04788	-0.02338	0.05104	0.18773	0.36778	
4096	-0.02339	-0.03156	-0.03998	-0.05152	-0.06717	-0.08008	-0.08320	-0.06211	0.01501	0.20182	0.45376		
2048	-0.03920	-0.05262	-0.06851	-0.08772	-0.10919	-0.11630	-0.10487	-0.05640	0.14516	0.47666			
1024	-0.06512	-0.08436	-0.10677	-0.13061	-0.14900	-0.16001	-0.09240	0.09176	0.40510				
512	-0.09531	-0.12575	-0.15203	-0.18979	-0.18661	-0.13789	0.01672	0.36128					
256	-0.14285	-0.17732	-0.21017	-0.22411	-0.17982	-0.03771	0.30672						
128	-0.19284	-0.22885	-0.25044	-0.22897	-0.06432	0.25513							
64	-0.24851	-0.27094	-0.24605	-0.11340	0.19685								
32	-0.29874	-0.26918	-0.15963	0.12907									
16	-0.29870	-0.18982	0.08785										
8	-0.22048	0.03453											
4	-0.01983												

(b) $\mathcal{P}^{\mathcal{C}}$ for Scene: [**Cornell Box**] Algorithm: [**BDPT**] Metric: [**SC-DM**] True GT: [**PT @ 32768 spp**].

PSSMLT	2	4	8	16	32	64	128	256	512	1024	2048	4096	8192
**GT (PT)**													
16384	-0.00365	-0.00498	-0.00684	-0.01021	-0.01110	-0.01264	-0.01777	-0.01852	-0.01383	-0.00448	0.00399	0.00747	0.01373
8192	-0.01104	-0.01458	-0.01867	-0.02570	-0.03024	-0.03498	-0.04491	-0.04382	-0.03034	-0.01042	0.01571	0.02995	
4096	-0.02143	-0.02857	-0.03689	-0.04866	-0.06134	-0.06794	-0.07115	-0.06128	-0.01942	0.02652	0.07454		
2048	-0.03564	-0.04738	-0.05979	-0.08191	-0.09375	-0.10230	-0.09972	-0.05730	0.01143	0.12007			
1024	-0.05747	-0.07452	-0.09445	-0.11603	-0.13725	-0.12487	-0.08583	0.01578	0.16342				
512	-0.08707	-0.11220	-0.13317	-0.15727	-0.15739	-0.10472	0.01207	0.21645					
256	-0.12991	-0.15314	-0.17496	-0.17631	-0.12885	0.00117	0.25093						
128	-0.17486	-0.19887	-0.20382	-0.15504	-0.00419	0.28312							
64	-0.22205	-0.22665	-0.17935	-0.02700	0.27884								
32	-0.26346	-0.21029	-0.05797	0.21840									
16	-0.24547	-0.10555	0.18683										
8	-0.15156	0.13100											
4	0.07645												

(c) $\mathcal{P}^{\mathcal{C}}$ for Scene: [**Cornell Box**] Algorithm: [**PSSMLT**] Metric: [**SC-DM**] True GT: [**PT @ 32768 spp**].

MLT	2	4	8	16	32	64	128	256	512	1024	2048	4096	8192
**GT (PT)**													
16384	-0.00223	-0.00259	-0.00375	-0.00492	-0.00728	-0.00918	-0.01126	-0.01289	-0.01460	-0.01556	-0.01605	-0.00541	-0.00341
8192	-0.00518	-0.00643	-0.00902	-0.01197	-0.01652	-0.02155	-0.02597	-0.03204	-0.03535	-0.03055	-0.02147	-0.01016	
4096	-0.01038	-0.01333	-0.01771	-0.02299	-0.03161	-0.04005	-0.04875	-0.05648	-0.05995	-0.05241	-0.02373		
2048	-0.01714	-0.02166	-0.03011	-0.04005	-0.05366	-0.07005	-0.08275	-0.08986	-0.08111	-0.05314			
1024	-0.02873	-0.03730	-0.04889	-0.06242	-0.08085	-0.10227	-0.11828	-0.11961	-0.08265				
512	-0.04466	-0.05581	-0.07105	-0.09002	-0.11321	-0.13730	-0.14248	-0.11133					
256	-0.06787	-0.08351	-0.10569	-0.12823	-0.15137	-0.16448	-0.13526						
128	-0.09524	-0.11690	-0.14528	-0.17135	-0.18761	-0.16818							
64	-0.13386	-0.15906	-0.18498	-0.19043	-0.17679								
32	-0.17757	-0.20280	-0.22135	-0.19530									
16	-0.22558	-0.23961	-0.23132										
8	-0.26025	-0.24585											
4	-0.26239												

(d) $\mathcal{P}^{\mathcal{C}}$ for Scene: [**Cornell Box**] Algorithm: [**MLT**] Metric: [**SC-DM**] True GT: [**PT @ 32768 spp**].

M-MLT	2	4	8	16	32	64	128	256	512	1024	2048	4096	8192
**GT (PT)**													
16384	-0.00202	-0.00275	-0.00336	-0.00414	-0.00596	-0.00805	-0.01003	-0.01051	-0.01804	-0.01428	-0.00877	-0.00402	0.00318
8192	-0.00507	-0.00734	-0.00929	-0.01171	-0.01540	-0.02130	-0.02774	-0.03167	-0.03426	-0.03647	-0.02691	-0.01145	
4096	-0.01086	-0.01400	-0.01786	-0.02281	-0.03046	-0.04088	-0.05091	-0.05920	-0.06605	-0.05143	-0.02381		
2048	-0.01870	-0.02404	-0.03097	-0.03917	-0.05271	-0.06843	-0.08489	-0.08913	-0.08615	-0.04639			
1024	-0.03023	-0.03890	-0.04905	-0.06251	-0.07947	-0.09685	-0.11131	-0.10930	-0.07709				
512	-0.04782	-0.05857	-0.07469	-0.09114	-0.11661	-0.13866	-0.13932	-0.10445					
256	-0.07125	-0.08969	-0.11157	-0.13424	-0.15864	-0.16719	-0.12367						
128	-0.10099	-0.12335	-0.14927	-0.16856	-0.17803	-0.14414							
64	-0.14226	-0.16689	-0.19227	-0.20072	-0.17183								
32	-0.18334	-0.20910	-0.22126	-0.18817									
16	-0.22838	-0.25015	-0.22391										
8	-0.25801	-0.24265											
4	-0.26076												

(e) $\mathcal{P}^{\mathcal{C}}$ for Scene: [**Cornell Box**] Algorithm: [**M-MLT**] Metric: [**SC-DM**] True GT: [**PT @ 32768 spp**].

ERPT	2	4	8	16	32	64	128	256	512	1024	2048	4096	8192
**GT (PT)**													
16384	-0.00979	-0.00975	-0.01187	-0.00934	-0.01142	-0.01144	-0.01058	-0.00998	-0.00982	-0.01282	-0.01110	-0.01078	-0.01029
8192	-0.02674	-0.02543	-0.02637	-0.02808	-0.02958	-0.02779	-0.02663	-0.02551	-0.02473	-0.03023	-0.02659	-0.02669	
4096	-0.05144	-0.04911	-0.04764	-0.04800	-0.05061	-0.04751	-0.05032	-0.05132	-0.05302	-0.04981	-0.04715		
2048	-0.07761	-0.07460	-0.08183	-0.08054	-0.08160	-0.07802	-0.07885	-0.07643	-0.08361	-0.07986			
1024	-0.10787	-0.11620	-0.11090	-0.11535	-0.11486	-0.10938	-0.10418	-0.10913	-0.10218				
512	-0.12100	-0.13175	-0.12849	-0.13124	-0.12754	-0.12296	-0.13129	-0.12336					
256	-0.10221	-0.10577	-0.10865	-0.10832	-0.10913	-0.10618	-0.10841						
128	0.01113	0.00611	0.01314	0.00680	0.00250	0.02007							
64	0.23978	0.25567	0.25997	0.24799	0.21811								
32	0.61690	0.62478	0.61864	0.61511									
16	1.09279	1.09297	1.09997										
8	1.59802	1.60800											
4	2.09339												

(f) $\mathcal{P}^{\mathcal{C}}$ for Scene: [**Cornell Box**] Algorithm: [**ERPT**] Metric: [**SC-DM**] True GT: [**PT @ 32768 spp**].

M-ERPT	2	4	8	16	32	64	128	256	512	1024	2048	4096	8192
**GT (PT)**													
16384	-0.01030	-0.01040	-0.01021	-0.01074	-0.00891	-0.00913	-0.01024	-0.01075	-0.01067	-0.01064	-0.00974	-0.01057	-0.01024
8192	-0.02835	-0.02463	-0.02548	-0.02756	-0.03026	-0.02780	-0.02620	-0.02809	-0.02718	-0.02814	-0.02600	-0.02588	
4096	-0.05088	-0.04982	-0.04916	-0.05205	-0.04693	-0.05137	-0.04871	-0.05370	-0.05001	-0.04972	-0.04925		
2048	-0.07693	-0.07710	-0.08013	-0.07766	-0.08076	-0.08237	-0.08022	-0.08506	-0.08179	-0.08028			
1024	-0.11494	-0.11442	-0.11094	-0.11836	-0.11264	-0.11845	-0.11262	-0.11762	-0.11397				
512	-0.13268	-0.12567	-0.13625	-0.13971	-0.13681	-0.13180	-0.12322	-0.13969					
256	-0.11389	-0.11742	-0.10828	-0.13302	-0.12587	-0.12407	-0.11299						
128	-0.00851	-0.00539	-0.00631	-0.00272	-0.02299	-0.01128							
64	0.23311	0.22201	0.22103	0.21754	0.21879								
32	0.59060	0.58801	0.57501	0.59380									
16	1.05852	1.05858	1.04644										
8	1.57032	1.56058											
4	2.07006												

(g) $\mathcal{P}^{\mathcal{C}}$ for Scene: [**Cornell Box**] Algorithm: [**M-ERPT**] Metric: [**SC-DM**] True GT: [**PT @ 32768 spp**].

Table 31

8.2 Torus

PT	2	4	8	16	32	64	128	256	512	1024	2048	4096	8192	16384	32768	65536	131072
GT (BDPT)																	
262144	-0.01191	-0.01677	-0.01764	-0.01703	-0.01959	-0.01894	-0.02124	-0.02492	-0.03128	-0.02979	-0.03379	-0.04477	-0.05400	-0.06386	-0.06514	-0.01667	0.05488
131072	-0.03051	-0.03935	-0.04249	-0.04117	-0.04737	-0.04398	-0.05187	-0.05849	-0.06740	-0.07244	-0.08686	-0.09996	-0.11983	-0.09063	-0.08578	0.05330	
65536	-0.05436	-0.06312	-0.06605	-0.06656	-0.07546	-0.07628	-0.08692	-0.10150	-0.11570	-0.12173	-0.13452	-0.14869	-0.16748	-0.12305	-0.02696		
32768	-0.08738	-0.10209	-0.10391	-0.10357	-0.11671	-0.12840	-0.14171	-0.16568	-0.18266	-0.19483	-0.21958	-0.21713	-0.20042	-0.07073			
16384	-0.12513	-0.14786	-0.15033	-0.14666	-0.16550	-0.18366	-0.19963	-0.23841	-0.25885	-0.27417	-0.31243	-0.28424	-0.17132				
8192	-0.15121	-0.17022	-0.16325	-0.16275	-0.18668	-0.22065	-0.25341	-0.29343	-0.32059	-0.32911	-0.34219	-0.23026					
4096	-0.17432	-0.18968	-0.17046	-0.17621	-0.20982	-0.24041	-0.30419	-0.35710	-0.37308	-0.36960	-0.31752						
2048	-0.18216	-0.18052	-0.13503	-0.13426	-0.17029	-0.24019	-0.29028	-0.35554	-0.38580	-0.33482							
1024	-0.17982	-0.16175	-0.09555	-0.09963	-0.13181	-0.18930	-0.26090	-0.34856	-0.34136								
512	-0.16053	-0.13169	-0.04601	-0.03779	-0.08215	-0.13784	-0.21648	-0.28335									
256	-0.14836	-0.11726	-0.01652	-0.01667	-0.06500	-0.10425	-0.15534										
128	-0.17871	-0.15629	-0.07966	-0.06810	-0.08651	-0.12282											
64	-0.20928	-0.20035	-0.10037	-0.08240	-0.09272												
32	-0.27046	-0.26444	-0.17680	-0.12985													
16	-0.33391	-0.31951	-0.26514														
8	-0.37369	-0.32116															
4	-0.38441																

(a) $\mathcal{P}^{\mathcal{C}}$ for Scene: [**Torus**] Algorithm: [**PT**] Metric: [**SC-DM**] True GT: [**BDPT @ 524288 spp**].

BDPT	2	4	8	16	32	64	128	256	512	1024	2048	4096	8192	16384	32768	65536	131072
GT (BDPT)																	
262144	-0.01296	-0.01584	-0.01635	-0.01788	-0.01985	-0.02223	-0.02249	-0.02294	-0.02773	-0.03147	-0.03161	-0.04707	-0.04695	-0.05769	-0.07372	-0.02456	0.05632
131072	-0.03072	-0.03801	-0.04164	-0.04287	-0.04357	-0.04710	-0.05223	-0.05391	-0.06812	-0.07434	-0.08254	-0.10161	-0.10336	-0.11602	-0.10574	0.03346	
65536	-0.05455	-0.06572	-0.06844	-0.06909	-0.07444	-0.07906	-0.09363	-0.10023	-0.11510	-0.12229	-0.14074	-0.16094	-0.17393	-0.14068	-0.05006		
32768	-0.08969	-0.10481	-0.10795	-0.11115	-0.11793	-0.12597	-0.14623	-0.15949	-0.18297	-0.20461	-0.21681	-0.23365	-0.21026	-0.12533			
16384	-0.12945	-0.15292	-0.14819	-0.15073	-0.16592	-0.18042	-0.20826	-0.23329	-0.26366	-0.28349	-0.29297	-0.26517	-0.20787				
8192	-0.15602	-0.17957	-0.17117	-0.17606	-0.20201	-0.21981	-0.25388	-0.29339	-0.32163	-0.34379	-0.33990	-0.24867					
4096	-0.18876	-0.20152	-0.19089	-0.19541	-0.21731	-0.25448	-0.29540	-0.34352	-0.38033	-0.39084	-0.30984						
2048	-0.19624	-0.19956	-0.15922	-0.17044	-0.19606	-0.24403	-0.31041	-0.35106	-0.37868	-0.33387							
1024	-0.18792	-0.17960	-0.13124	-0.14027	-0.14628	-0.20486	-0.29613	-0.34156	-0.33797								
512	-0.17593	-0.14956	-0.08879	-0.08032	-0.09691	-0.15959	-0.24182	-0.28230									
256	-0.16574	-0.13618	-0.06681	-0.06472	-0.08269	-0.10446	-0.20022										
128	-0.18707	-0.17168	-0.09531	-0.09871	-0.10049	-0.13328											
64	-0.21384	-0.21181	-0.12930	-0.12121	-0.11893												
32	-0.27245	-0.28759	-0.19692	-0.19460													
16	-0.35074	-0.33859	-0.23140														
8	-0.38407	-0.35190															
4	-0.38397																

(b) $\mathcal{P}^{\mathcal{C}}$ for Scene: [**Torus**] Algorithm: [**BDPT**] Metric: [**SC-DM**] True GT: [**BDPT @ 524288 spp**].

PSSMLT	2	4	8	16	32	64	128	256	512	1024	2048	4096	8192	16384
GT (BDPT)														
262144	-0.01555	-0.01632	-0.01558	-0.01812	-0.01756	-0.01886	-0.02210	-0.02007	-0.01922	-0.02144	-0.02690	-0.02214	-0.01532	-0.00615
131072	-0.03728	-0.03777	-0.03705	-0.04080	-0.04341	-0.04275	-0.04854	-0.05062	-0.05473	-0.05430	-0.04955	-0.03621	-0.00681	0.02459
65536	-0.06101	-0.06234	-0.05956	-0.06300	-0.07061	-0.07546	-0.07739	-0.08186	-0.08899	-0.08533	-0.07127	-0.04838	0.03495	0.10376
32768	-0.10132	-0.10013	-0.09637	-0.10565	-0.11588	-0.12293	-0.12865	-0.12282	-0.11591	-0.08841	-0.04605	0.02269	0.16911	0.29185
16384	-0.14224	-0.13686	-0.13233	-0.13525	-0.14687	-0.14747	-0.14619	-0.12981	-0.10823	-0.05792	0.03900	0.19661	0.42330	
8192	-0.19687	-0.19074	-0.17792	-0.18200	-0.17587	-0.17296	-0.14731	-0.12098	-0.04365	0.07159	0.25598	0.46602		
4096	-0.24491	-0.22203	-0.21105	-0.20665	-0.19331	-0.18361	-0.14594	-0.05725	0.08528	0.24885	0.50291			
2048	-0.26207	-0.20537	-0.17557	-0.16874	-0.16195	-0.11783	-0.02869	0.10742	0.30573	0.53786				
1024	-0.26511	-0.18973	-0.14698	-0.13254	-0.09803	-0.03601	0.08441	0.25286	0.50456					
512	-0.22169	-0.13655	-0.07897	-0.05772	-0.00687	0.08892	0.23274	0.43636						
256	-0.17758	-0.07126	-0.01420	0.01548	0.06537	0.17007	0.32138							
128	-0.16747	-0.07946	0.00237	0.00796	0.07042	0.18058								
64	-0.11961	-0.03923	0.03267	0.05198	0.13752									
32	-0.12313	-0.03868	0.05250	0.06576										
16	-0.11302	-0.02215	0.09845											
8	-0.09855	0.00224												
4	-0.01767													

(c) $\mathcal{P}^{\mathcal{C}}$ for Scene: [**Torus**] Algorithm: [**PSSMLT**] Metric: [**SC-DM**] True GT: [**BDPT @ 524288 spp**].

MLT	2	4	8	16	32	64	128	256	512	1024	2048	4096	8192	16384
GT (BDPT)														
262144	-0.00827	-0.00620	-0.00927	-0.00733	-0.00917	-0.00572	-0.00550	-0.00792	-0.00666	-0.00886	-0.00800	-0.00600	-0.00587	-0.00670
131072	-0.01825	-0.01460	-0.02206	-0.01437	-0.01702	-0.01439	-0.01088	-0.01146	-0.00463	-0.00912	-0.00603	-0.00297	0.00105	0.00597
65536	-0.03158	-0.03059	-0.04069	-0.02867	-0.02880	-0.02253	-0.01289	-0.01756	-0.00216	-0.00393	-0.00197	0.00596	0.01137	0.03090
32768	-0.05045	-0.04543	-0.05926	-0.03632	-0.03648	-0.02031	-0.00683	0.00098	0.01576	0.02248	0.03257	0.04178	0.06098	0.08907
16384	-0.07666	-0.07869	-0.08898	-0.05683	-0.04690	-0.01797	0.01538	0.02365	0.05365	0.06951	0.08598	0.10517	0.13318	
8192	-0.11523	-0.10803	-0.11286	-0.06556	-0.03763	-0.00485	0.05416	0.08778	0.12495	0.15727	0.18988	0.21827		
4096	-0.14425	-0.13203	-0.13553	-0.04714	-0.00172	0.04181	0.13132	0.19982	0.24781	0.29956	0.33827			
2048	-0.17825	-0.16124	-0.13133	-0.02848	0.07332	0.14550	0.25035	0.35011	0.39881	0.47134				
1024	-0.18865	-0.15439	-0.09645	0.03764	0.18760	0.27059	0.39959	0.51088	0.56033					
512	-0.18455	-0.13516	-0.05854	0.11227	0.27958	0.37033	0.51174	0.66876						
256	-0.16536	-0.11686	-0.02685	0.15285	0.34856	0.41132	0.59099							
128	-0.13650	-0.08999	-0.00701	0.18282	0.36227	0.44012								
64	-0.11688	-0.06437	0.00983	0.19048	0.36925									
32	-0.11954	-0.03821	0.00620	0.20325										
16	-0.14063	-0.05104	-0.00392											
8	-0.11223	-0.01733												
4	-0.11490													

(d) $\mathcal{P}^{\mathcal{C}}$ for Scene: [**Torus**] Algorithm: [**MLT**] Metric: [**SC-DM**] True GT: [**BDPT @ 524288 spp**].

M-MLT	2	4	8	16	32	64	128	256	512	1024	2048	4096	8192	16384
GT (BDPT)														
262144	-0.00648	-0.00849	-0.00744	-0.00767	-0.01161	-0.00642	-0.00196	-0.00898	-0.01191	-0.00723	-0.00687	-0.00613	-0.00357	0.00154
131072	-0.01522	-0.01935	-0.02200	-0.01571	-0.02359	-0.01276	-0.00764	-0.01733	-0.00986	0.00327	0.00364	0.00733	0.01614	0.03111
65536	-0.02765	-0.03644	-0.03590	-0.02199	-0.04075	-0.01556	-0.00831	-0.02309	-0.00793	0.00610	0.01473	0.02513	0.03797	0.06739
32768	-0.04313	-0.05600	-0.05023	-0.03220	-0.05004	-0.01095	-0.00010	-0.01277	0.01884	0.04455	0.06404	0.07920	0.10915	0.16379
16384	-0.06628	-0.08449	-0.07500	-0.04476	-0.05874	0.00071	0.01041	0.01999	0.06398	0.11882	0.16095	0.18013	0.23357	
8192	-0.09994	-0.11356	-0.09034	-0.04916	-0.06044	0.02702	0.05540	0.10815	0.18432	0.24146	0.33156	0.36314		
4096	-0.12318	-0.14421	-0.08734	-0.03224	-0.03549	0.09905	0.13702	0.21864	0.32088	0.42428	0.54457			
2048	-0.14832	-0.15854	-0.06214	-0.00296	0.04286	0.18726	0.27634	0.41651	0.53207	0.64486				
1024	-0.16626	-0.15756	-0.00886	0.06419	0.12626	0.32897	0.45310	0.62576	0.75807					
512	-0.13750	-0.13031	0.04557	0.13372	0.23245	0.41597	0.56847	0.78752						
256	-0.12690	-0.12065	0.10269	0.17830	0.28958	0.49109	0.64208							
128	-0.10142	-0.08650	0.13361	0.19519	0.30519	0.51327								
64	-0.08074	-0.08870	0.13082	0.20349	0.34390									
32	-0.07376	-0.09016	0.13845	0.20918										
16	-0.07327	-0.09934	0.15162											
8	-0.02892	-0.07836												
4	-0.03226													

(e) $\mathcal{P}^{\mathcal{C}}$ for Scene: [**Torus**] Algorithm: [**M-MLT**] Metric: [**SC-DM**] True GT: [**BDPT @ 524288 spp**].

ERPT	2	4	8	16	32	64	128	256	512	1024	2048	4096	8192	16384	32768	65536	131072
GT (BDPT)																	
262144	-0.01793	-0.01150	-0.00994	-0.00910	0.00035	-0.01184	-0.01095	-0.01696	-0.01968	-0.01870	-0.01853	-0.01816	-0.01592	-0.01519	-0.02154	-0.02021	-0.01407
131072	-0.04030	-0.01331	-0.02102	-0.01193	-0.01803	-0.02252	-0.04030	-0.04101	-0.03291	-0.04527	-0.04184	-0.04002	-0.03626	-0.04079	-0.03747	-0.03575	
65536	-0.04464	-0.01114	-0.02394	-0.01972	-0.01870	-0.03324	-0.03981	-0.05037	-0.05192	-0.05514	-0.04581	-0.03832	-0.02577	-0.02526	-0.03610		
32768	-0.05135	-0.00267	-0.02255	0.00200	0.00595	-0.01407	-0.03123	-0.04890	-0.03129	-0.04033	-0.01038	0.00289	0.01700	0.00120			
16384	-0.05522	0.03686	0.02130	0.04287	0.04273	0.04264	0.02412	0.02291	0.04864	0.05629	0.09416	0.14412	0.14140				
8192	0.00260	0.12215	0.09190	0.15787	0.15290	0.15962	0.16723	0.16559	0.20854	0.23323	0.31586	0.35063					
4096	0.09649	0.22336	0.20312	0.29873	0.30007	0.34628	0.33676	0.38698	0.43304	0.48984	0.57835						
2048	0.24350	0.41072	0.38720	0.54180	0.52876	0.56975	0.60669	0.67929	0.73224	0.81004							
1024	0.38956	0.61588	0.55501	0.75217	0.76879	0.80577	0.86670	0.93716	1.00575								
512	0.52611	0.76090	0.72676	0.91332	0.92125	0.99669	1.04584	1.15113									
256	0.59808	0.82663	0.80240	0.99950	1.01064	1.08332	1.17053										
128	0.57313	0.80805	0.79315	1.00752	1.04944	1.11770											
64	0.55607	0.77773	0.78590	1.00879	1.02161												
32	0.54231	0.79030	0.77612	0.98458													
16	0.53299	0.79933	0.80036														
8	0.57616	0.84845															
4	0.79733																

(f) $\mathcal{P}^{\mathcal{C}}$ for Scene: [**Torus**] Algorithm: [**ERPT**] Metric: [**SC-DM**] True GT: [**BDPT @ 524288 spp**].

M-ERPT	2	4	8	16	32	64	128	256	512	1024	2048	4096	8192	16384	32768	65536	131072
GT (BDPT)																	
262144	-0.00155	-0.00778	-0.00953	-0.00397	-0.01068	-0.01578	-0.00883	-0.01584	-0.02047	-0.02083	-0.01282	-0.01502	-0.01447	-0.01860	-0.02354	-0.01736	-0.01739
131072	-0.00757	-0.00976	-0.02175	-0.01867	-0.02870	-0.04344	-0.03298	-0.03300	-0.02810	-0.04993	-0.03747	-0.04061	-0.04174	-0.03918	-0.03954	-0.03754	
65536	-0.01802	-0.01585	-0.02217	-0.01098	-0.03791	-0.05230	-0.04455	-0.04215	-0.03783	-0.04548	-0.04907	-0.04418	-0.04037	-0.03995	-0.04677		
32768	-0.01571	-0.01496	-0.03226	0.00928	-0.03231	-0.02576	-0.01617	-0.02694	-0.00704	-0.01881	-0.00307	-0.01454	-0.00548	0.00331			
16384	-0.00688	-0.00222	-0.01840	0.03713	-0.00412	0.04146	0.05142	0.06323	0.08791	0.08789	0.12560	0.10822	0.11394				
8192	0.01763	0.03180	0.06152	0.14500	0.11081	0.17380	0.20603	0.22611	0.28726	0.30870	0.35548	0.32076					
4096	0.09172	0.09254	0.15532	0.27875	0.28742	0.41560	0.40550	0.44378	0.53143	0.56799	0.60886						
2048	0.17550	0.21246	0.33452	0.48226	0.49947	0.68910	0.68925	0.74930	0.84304	0.90412							
1024	0.32296	0.34877	0.48135	0.66670	0.74860	0.96164	0.96277	1.02939	1.14183								
512	0.41562	0.45194	0.63397	0.82245	0.92064	1.15407	1.14888	1.22836									
256	0.46415	0.51696	0.70291	0.91059	1.01844	1.25501	1.26105										
128	0.45127	0.52067	0.71191	0.92812	1.02951	1.28594											
64	0.44954	0.48942	0.68809	0.92964	1.00591												
32	0.46300	0.50233	0.67716	0.89915													
16	0.45728	0.49213	0.65904														
8	0.48839	0.52093															
4	0.66718																

(g) $\mathcal{P}^{\mathcal{C}}$ for Scene: [**Torus**] Algorithm: [**M-ERPT**] Metric: [**SC-DM**] True GT: [**BDPT @ 524288 spp**].

Table 32

8.3 Veach Bidir

PT	2	4	8	16	32	64	128	256	512	1024	2048	4096	8192	16384	32768	65536	131072
GT (BDPT)																	
262144	-0.00012	-0.00015	-0.00008	-0.00027	-0.00024	-0.00030	-0.00030	-0.00037	-0.00055	-0.00106	-0.00055	-0.00108	-0.00015	-0.00155	-0.00103	-0.00157	-0.00149
131072	-0.00038	-0.00044	-0.00023	-0.00051	-0.00044	-0.00058	-0.00086	-0.00069	-0.00069	-0.00111	-0.00127	-0.00106	-0.00144	-0.00188	-0.00103	-0.00173	
65536	-0.00072	-0.00096	-0.00094	-0.00148	-0.00162	-0.00163	-0.00207	-0.00204	-0.00271	-0.00342	-0.00353	-0.00444	-0.00435	-0.00484	-0.00566		
32768	-0.00156	-0.00211	-0.00212	-0.00250	-0.00302	-0.00352	-0.00433	-0.00470	-0.00551	-0.00665	-0.00676	-0.00914	-0.00859	-0.00990			
16384	-0.00239	-0.00313	-0.00383	-0.00460	-0.00513	-0.00593	-0.00783	-0.00850	-0.01058	-0.01115	-0.01179	-0.01491	-0.01406				
8192	-0.00431	-0.00548	-0.00652	-0.00827	-0.00902	-0.01084	-0.01287	-0.01458	-0.01735	-0.01980	-0.02099	-0.02052					
4096	-0.00737	-0.00922	-0.01139	-0.01330	-0.01515	-0.01720	-0.02069	-0.02377	-0.02732	-0.03117	-0.03357						
2048	-0.01227	-0.01555	-0.01835	-0.02167	-0.02453	-0.02900	-0.03408	-0.04013	-0.04480	-0.05142							
1024	-0.01914	-0.02455	-0.02852	-0.03325	-0.03973	-0.04508	-0.05206	-0.06113	-0.07044								
512	-0.02907	-0.03710	-0.04394	-0.04981	-0.05830	-0.06458	-0.07737	-0.08982									
256	-0.04326	-0.05482	-0.06305	-0.07305	-0.08490	-0.09440	-0.10944										
128	-0.06518	-0.07796	-0.09501	-0.10463	-0.12223	-0.13272											
64	-0.09210	-0.11339	-0.13354	-0.14812	-0.16499												
32	-0.13112	-0.15661	-0.17963	-0.19885													
16	-0.17829	-0.20597	-0.22953														
8	-0.22993	-0.26825															
4	-0.28026																

(a) $\mathcal{P}^{\mathcal{C}}$ for Scene: [**Veach Bidir**] Algorithm: [**PT**] Metric: [**SC-DM**] True GT: [**BDPT @ 524288 spp**].

BDPT	2	4	8	16	32	64	128	256	512	1024	2048	4096	8192	16384	32768	65536	131072
GT (BDPT)																	
262144	-0.00054	-0.00049	-0.00050	-0.00082	-0.00105	-0.00121	-0.00319	-0.00405	-0.00184	-0.01369	-0.00363	0.03648	-0.04240	-0.07359	-0.05407	0.00310	0.02700
131072	-0.00067	-0.00068	-0.00049	-0.00110	-0.00089	-0.00183	-0.00154	-0.00194	-0.00090	0.01046	0.01774	0.05507	-0.03304	0.01341	-0.01668	0.07975	
65536	-0.00211	-0.00241	-0.00279	-0.00404	-0.00574	-0.00678	-0.00859	-0.01205	-0.01926	-0.01261	-0.00839	0.03089	-0.05580	0.04152	0.10435		
32768	-0.00437	-0.00497	-0.00634	-0.00763	-0.00956	-0.00880	-0.01058	-0.01142	-0.01431	0.00150	0.01578	0.17925	0.11276	0.21446			
16384	-0.00729	-0.00959	-0.01260	-0.01637	-0.02164	-0.02985	-0.04338	-0.05143	-0.06523	-0.05583	-0.03021	0.20041	0.22376				
8192	-0.01236	-0.01539	-0.02099	-0.02643	-0.03482	-0.04462	-0.05454	-0.07035	-0.05832	-0.03021	0.08558	0.37055					
4096	-0.01898	-0.02466	-0.03187	-0.04339	-0.05327	-0.06852	-0.08402	-0.10343	-0.08228	-0.07224	0.10402						
2048	-0.03248	-0.04169	-0.05382	-0.07269	-0.09348	-0.11608	-0.14535	-0.18281	-0.22004	-0.04805							
1024	-0.04971	-0.06391	-0.08234	-0.11601	-0.14214	-0.17402	-0.19378	-0.23418	-0.15599								
512	-0.07475	-0.09454	-0.11896	-0.16205	-0.19224	-0.21220	-0.21682	-0.18634									
256	-0.10664	-0.13327	-0.16698	-0.21463	-0.23576	-0.21879	-0.16655										
128	-0.15135	-0.18818	-0.22746	-0.26399	-0.27175	-0.20616											
64	-0.20683	-0.25527	-0.28667	-0.29563	-0.25057												
32	-0.27284	-0.30588	-0.32284	-0.24925													
16	-0.32164	-0.32839	-0.27311														
8	-0.34491	-0.29042															
4	-0.31488																

(b) $\mathcal{P}^{\mathcal{C}}$ for Scene: [**Veach Bidir**] Algorithm: [**BDPT**] Metric: [**SC-DM**] True GT: [**BDPT @ 524288 spp**].

PSSMLT	2	4	8	16	32	64	128	256	512	1024	2048	4096	8192	16384
GT (BDPT)														
262144	-0.00030	-0.00046	-0.00061	-0.00109	-0.00127	-0.00157	-0.00131	-0.00134	-0.00261	-0.00302	-0.00176	-0.00292	-0.00202	-0.00174
131072	-0.00059	-0.00054	-0.00104	-0.00073	-0.00114	-0.00076	0.00038	0.00043	0.00059	0.00161	-0.00180	0.00259	0.00196	0.00195
65536	-0.00145	-0.00195	-0.00264	-0.00347	-0.00479	-0.00531	-0.00590	-0.00609	-0.00703	-0.00843	-0.00396	-0.00125	0.00062	0.00931
32768	-0.00288	-0.00420	-0.00582	-0.00731	-0.00871	-0.00964	-0.00936	-0.00611	-0.00639	-0.01089	-0.01060	0.00781	0.01371	0.01864
16384	-0.00502	-0.00710	-0.00975	-0.01312	-0.01654	-0.02293	-0.02691	-0.03019	-0.02347	-0.01283	0.00419	0.01501	0.02987	
8192	-0.00844	-0.01194	-0.01587	-0.02284	-0.02918	-0.03671	-0.04053	-0.04750	-0.04366	-0.02804	-0.00788	0.00344		
4096	-0.01452	-0.01954	-0.02657	-0.03474	-0.04495	-0.05509	-0.06506	-0.07394	-0.06373	-0.04872	-0.00799			
2048	-0.02416	-0.03268	-0.04399	-0.05731	-0.07531	-0.09739	-0.11760	-0.13805	-0.10954	-0.07336				
1024	-0.03883	-0.05182	-0.06761	-0.08765	-0.11354	-0.14020	-0.15575	-0.18399	-0.13076					
512	-0.05794	-0.07704	-0.09887	-0.12487	-0.14975	-0.17432	-0.17159	-0.18253						
256	-0.08345	-0.10640	-0.13545	-0.16262	-0.18345	-0.19208	-0.13925							
128	-0.11597	-0.14672	-0.17993	-0.20852	-0.21026	-0.16994								
64	-0.16380	-0.20229	-0.23914	-0.25470	-0.20414									
32	-0.20826	-0.23760	-0.25932	-0.20625										
16	-0.25343	-0.26121	-0.21145											
8	-0.27934	-0.23396												
4	-0.24264													

(c) $\mathcal{P}^{\mathcal{C}}$ for Scene: [**Veach Bidir**] Algorithm: [**PSSMLT**] Metric: [**SC-DM**] True GT: [**BDPT @ 524288 spp**].

MLT	2	4	8	16	32	64	128	256	512	1024	2048	4096	8192	16384
GT (BDPT)														
262144	0.00000	-0.00006	-0.00017	-0.00012	-0.00027	-0.00032	-0.00049	-0.00046	-0.00109	-0.00211	-0.00416	-0.00307	-0.00328	-0.00312
131072	-0.00024	-0.00033	-0.00047	-0.00061	-0.00081	-0.00085	-0.00084	-0.00108	-0.00123	-0.00199	-0.00118	-0.00060	0.00267	0.00504
65536	-0.00066	-0.00089	-0.00116	-0.00138	-0.00157	-0.00290	-0.00327	-0.00387	-0.00515	-0.00665	-0.00958	-0.00828	-0.00702	-0.00527
32768	-0.00136	-0.00169	-0.00249	-0.00355	-0.00404	-0.00523	-0.00645	-0.00667	-0.00718	-0.00803	-0.00817	0.00075	0.00429	0.01585
16384	-0.00213	-0.00276	-0.00351	-0.00494	-0.00660	-0.01024	-0.01448	-0.01815	-0.02258	-0.02957	-0.02834	-0.02753	-0.02585	
8192	-0.00420	-0.00580	-0.00684	-0.00895	-0.01184	-0.01697	-0.02466	-0.02891	-0.03645	-0.04383	-0.04764	-0.04234		
4096	-0.00639	-0.00903	-0.01134	-0.01461	-0.01886	-0.02608	-0.03445	-0.04397	-0.05411	-0.05900	-0.05302			
2048	-0.01073	-0.01467	-0.01850	-0.02471	-0.03213	-0.04380	-0.05695	-0.07189	-0.08791	-0.10066				
1024	-0.01757	-0.02284	-0.02939	-0.03802	-0.04855	-0.06638	-0.08642	-0.10541	-0.12402					
512	-0.02677	-0.03486	-0.04397	-0.05765	-0.07239	-0.09613	-0.11859	-0.13719						
256	-0.04076	-0.05263	-0.06503	-0.08227	-0.10268	-0.12987	-0.15672							
128	-0.06125	-0.07875	-0.09405	-0.11928	-0.14288	-0.16875								
64	-0.08856	-0.11045	-0.13276	-0.16019	-0.18200									
32	-0.12252	-0.14861	-0.17543	-0.20324										
16	-0.16733	-0.19936	-0.21736											
8	-0.21457	-0.23891												
4	-0.25573													

(d) $\mathcal{P}^{\mathcal{C}}$ for Scene: [**Veach Bidir**] Algorithm: [**MLT**] Metric: [**SC-DM**] True GT: [**BDPT @ 524288 spp**].

M-MLT	2	4	8	16	32	64	128	256	512	1024	2048	4096	8192	16384
GT (BDPT)														
262144	-0.00010	-0.00004	-0.00005	-0.00025	-0.00040	-0.00091	-0.00099	-0.00098	-0.00060	-0.00097	-0.00097	-0.00017	-0.00281	-0.00152
131072	-0.00040	-0.00024	-0.00039	-0.00043	-0.00060	-0.00066	-0.00018	-0.00005	0.00034	0.00074	0.00320	0.00329	0.00580	0.00546
65536	-0.00058	-0.00076	-0.00078	-0.00143	-0.00191	-0.00233	-0.00361	-0.00559	-0.00562	-0.00556	-0.00419	-0.00564	-0.00548	-0.00165
32768	-0.00131	-0.00163	-0.00187	-0.00316	-0.00391	-0.00572	-0.00662	-0.00852	-0.00928	-0.00291	0.00371	0.00705	0.01722	0.01916
16384	-0.00220	-0.00278	-0.00352	-0.00561	-0.00727	-0.01020	-0.01257	-0.01756	-0.02251	-0.02617	-0.02351	-0.02801	-0.02864	
8192	-0.00375	-0.00477	-0.00657	-0.00901	-0.01148	-0.01588	-0.02173	-0.02481	-0.02914	-0.03037	-0.02989	-0.02807		
4096	-0.00653	-0.00890	-0.01113	-0.01510	-0.01995	-0.02646	-0.03625	-0.04204	-0.04977	-0.05647	-0.04857			
2048	-0.01094	-0.01492	-0.01926	-0.02637	-0.03345	-0.04628	-0.06095	-0.07260	-0.09405	-0.09442				
1024	-0.01751	-0.02389	-0.02958	-0.03880	-0.04951	-0.06757	-0.09029	-0.11271	-0.12834					
512	-0.02781	-0.03639	-0.04445	-0.05970	-0.07515	-0.09763	-0.12690	-0.13526						
256	-0.04063	-0.05297	-0.06626	-0.08564	-0.10492	-0.13207	-0.16300							
128	-0.06171	-0.07816	-0.09603	-0.12166	-0.14553	-0.16970								
64	-0.08849	-0.11119	-0.13262	-0.16083	-0.18319									
32	-0.12466	-0.15386	-0.18059	-0.20799										
16	-0.16496	-0.20126	-0.22186											
8	-0.21968	-0.24960												
4	-0.26130													

(e) $\mathcal{P}^{\mathcal{C}}$ for Scene: [**Veach Bidir**] Algorithm: [**M-MLT**] Metric: [**SC-DM**] True GT: [**BDPT @ 524288 spp**].

ERPT	2	4	8	16	32	64	128	256	512	1024	2048	4096	8192	16384	32768	65536	131072
GT (BDPT)																	
262144	-0.00011	-0.00118	-0.00134	-0.00063	-0.00101	-0.00119	-0.00066	-0.00049	-0.00123	-0.00045	-0.00026	-0.00070	-0.00060	-0.00065	-0.00047	-0.00066	-0.00063
131072	-0.00005	-0.00128	-0.00122	-0.00037	-0.00061	-0.00116	-0.00048	-0.00064	-0.00076	-0.00085	-0.00071	-0.00072	-0.00068	-0.00085	-0.00017	-0.00076	
65536	-0.00301	-0.00355	-0.00371	-0.00314	-0.00324	-0.00351	-0.00245	-0.00256	-0.00279	-0.00201	-0.00256	-0.00284	-0.00196	-0.00267	-0.00232		
32768	-0.00601	-0.00667	-0.00724	-0.00672	-0.00535	-0.00500	-0.00479	-0.00672	-0.00677	-0.00661	-0.00504	-0.00579	-0.00558	-0.00588			
16384	-0.01308	-0.01463	-0.01451	-0.01305	-0.01501	-0.01270	-0.01262	-0.00967	-0.01095	-0.01008	-0.00980	-0.00943	-0.00960				
8192	-0.02169	-0.02157	-0.02495	-0.02334	-0.02100	-0.02044	-0.02077	-0.02273	-0.02242	-0.01977	-0.02086	-0.01958					
4096	-0.03437	-0.03458	-0.03627	-0.03511	-0.03695	-0.03588	-0.03456	-0.03453	-0.03401	-0.03369	-0.03418						
2048	-0.06116	-0.06076	-0.06363	-0.06091	-0.06576	-0.05961	-0.05986	-0.05692	-0.05749	-0.05529							
1024	-0.08766	-0.09042	-0.09271	-0.09220	-0.09958	-0.08515	-0.08856	-0.08315	-0.09014								
512	-0.12012	-0.11789	-0.12372	-0.11819	-0.13035	-0.11630	-0.11825	-0.11699									
256	-0.14471	-0.15574	-0.15492	-0.15275	-0.16471	-0.15199	-0.15284										
128	-0.16781	-0.17534	-0.17860	-0.17428	-0.18132	-0.17186											
64	-0.16629	-0.17843	-0.17632	-0.17271	-0.16569												
32	-0.06104	-0.05300	-0.05876	-0.05295													
16	0.19080	0.18197	0.18135														
8	0.54227	0.54729															
4	0.97715																

(f) $\mathcal{P}^{\mathcal{C}}$ for Scene: [**Veach Bidir**] Algorithm: [**ERPT**] Metric: [**SC-DM**] True GT: [**BDPT @ 524288 spp**].

M-ERPT	2	4	8	16	32	64	128	256	512	1024	2048	4096	8192	16384	32768	65536	131072
GT (BDPT)																	
262144	-0.00074	-0.00037	-0.00034	-0.00081	-0.00072	-0.00050	0.00025	-0.00086	-0.00043	-0.00001	-0.00033	-0.00105	-0.00044	-0.00058	-0.00035	-0.00002	-0.00056
131072	-0.00074	-0.00016	0.00009	-0.00044	-0.00059	-0.00071	0.00052	-0.00053	-0.00049	-0.00033	-0.00054	-0.00097	-0.00087	-0.00095	-0.00053	-0.00015	
65536	-0.00316	-0.00297	-0.00297	-0.00327	-0.00367	-0.00308	-0.00144	-0.00274	-0.00225	-0.00236	-0.00190	-0.00310	-0.00226	-0.00294	-0.00227		
32768	-0.00592	-0.00550	-0.00613	-0.00781	-0.00656	-0.00623	-0.00571	-0.00674	-0.00739	-0.00691	-0.00558	-0.00630	-0.00700	-0.00695			
16384	-0.01381	-0.01290	-0.01045	-0.01154	-0.01360	-0.01015	-0.01097	-0.01052	-0.01147	-0.00935	-0.00791	-0.01040	-0.00954				
8192	-0.02078	-0.02167	-0.01970	-0.02087	-0.02218	-0.02210	-0.01598	-0.02037	-0.01980	-0.01969	-0.01692	-0.01993					
4096	-0.03411	-0.03506	-0.03359	-0.03377	-0.03737	-0.03462	-0.03307	-0.03192	-0.03424	-0.03452	-0.03183						
2048	-0.06053	-0.06238	-0.05559	-0.05645	-0.06213	-0.05653	-0.05877	-0.05799	-0.06038	-0.05592							
1024	-0.08926	-0.09116	-0.08104	-0.09232	-0.09453	-0.08140	-0.07649	-0.08153	-0.08555								
512	-0.12013	-0.12694	-0.10956	-0.11853	-0.12449	-0.11278	-0.11251	-0.11683									
256	-0.15343	-0.15347	-0.14275	-0.14687	-0.16428	-0.14574	-0.14955										
128	-0.18570	-0.18790	-0.15965	-0.17683	-0.17612	-0.16633											
64	-0.19802	-0.19589	-0.15699	-0.17470	-0.17923												
32	-0.08386	-0.10157	-0.06830	-0.08118													
16	0.14169	0.12673	0.17057														
8	0.49916	0.48218															
4	0.91689																

(g) $\mathcal{P}^{\mathcal{C}}$ for Scene: [**Veach Bidir**] Algorithm: [**M-ERPT**] Metric: [**SC-DM**] True GT: [**BDPT @ 524288 spp**].

Table 33

8.4 Veach Door

PT	2	4	8	16	32	64	128	256	512	1024	2048	4096	8192	16384	32768	65536	131072
GT (BDPT)																	
262144	-0.00075	-0.00096	-0.00117	-0.00168	-0.00195	-0.00261	-0.00265	-0.00287	-0.00369	-0.00442	-0.00599	-0.00620	-0.00813	-0.01347	-0.01230	-0.00777	-0.01079
131072	-0.00183	-0.00249	-0.00326	-0.00422	-0.00488	-0.00607	-0.00694	-0.00830	-0.00986	-0.01210	-0.01527	-0.01796	-0.02254	-0.03306	-0.03364	-0.03590	
65536	-0.00372	-0.00544	-0.00749	-0.00923	-0.01109	-0.01289	-0.01496	-0.01778	-0.02142	-0.02690	-0.03180	-0.03959	-0.04990	-0.06303	-0.07075		
32768	-0.00700	-0.00995	-0.01378	-0.01650	-0.02026	-0.02324	-0.02615	-0.03135	-0.03726	-0.04701	-0.05528	-0.06665	-0.08296	-0.09989			
16384	-0.01216	-0.01651	-0.02268	-0.02785	-0.03332	-0.03805	-0.04374	-0.05129	-0.06181	-0.07527	-0.08966	-0.10519	-0.11971				
8192	-0.02081	-0.02808	-0.03778	-0.04488	-0.05216	-0.06029	-0.06959	-0.08080	-0.09320	-0.11009	-0.12835	-0.14641					
4096	-0.03319	-0.04357	-0.05772	-0.06869	-0.08053	-0.09020	-0.10347	-0.11733	-0.13485	-0.16158	-0.17749						
2048	-0.05085	-0.06605	-0.08537	-0.10057	-0.11537	-0.13163	-0.14664	-0.16780	-0.18497	-0.21060							
1024	-0.07653	-0.09734	-0.12545	-0.14679	-0.16578	-0.18418	-0.20275	-0.21958	-0.24146								
512	-0.10992	-0.13535	-0.17005	-0.19394	-0.21860	-0.23632	-0.26235	-0.26589									
256	-0.14622	-0.17995	-0.22357	-0.25500	-0.28034	-0.29650	-0.30125										
128	-0.18527	-0.22575	-0.27526	-0.30510	-0.31280	-0.31923											
64	-0.23230	-0.28370	-0.32725	-0.34597	-0.34185												
32	-0.27820	-0.33105	-0.37151	-0.35900													
16	-0.32938	-0.37377	-0.38674														
8	-0.36754	-0.40215															
4	-0.39742																

(a) $\mathcal{P}^{\mathcal{C}}$ for Scene: [**Veach Door**] Algorithm: [**PT**] Metric: [**SC-DM**] True GT: [**BDPT @ 524288 spp**].

BDPT	2	4	8	16	32	64	128	256	512	1024	2048	4096	8192	16384	32768	65536	131072
GT (BDPT)																	
262144	-0.00098	-0.00156	-0.00161	-0.00213	-0.00246	-0.00334	-0.00449	-0.00581	-0.00665	-0.00904	-0.01108	-0.01291	-0.01348	-0.00375	0.01453	0.04992	0.12240
131072	-0.00285	-0.00357	-0.00449	-0.00599	-0.00698	-0.00911	-0.01122	-0.01495	-0.01711	-0.02327	-0.03357	-0.04068	-0.04047	-0.02359	0.01598	0.11560	
65536	-0.00577	-0.00777	-0.00983	-0.01295	-0.01523	-0.02020	-0.02507	-0.03297	-0.04031	-0.05322	-0.06776	-0.06892	-0.06046	-0.01518	0.11551		
32768	-0.01102	-0.01437	-0.01853	-0.02180	-0.02699	-0.03448	-0.04319	-0.05439	-0.06654	-0.08588	-0.10243	-0.10543	-0.06413	0.08935			
16384	-0.01826	-0.02354	-0.02958	-0.03675	-0.04489	-0.05508	-0.06764	-0.08816	-0.10494	-0.12795	-0.14367	-0.11156	-0.00522				
8192	-0.02997	-0.03882	-0.04691	-0.05881	-0.07034	-0.08836	-0.10433	-0.12891	-0.14708	-0.16441	-0.14704	-0.03027					
4096	-0.04626	-0.05953	-0.07193	-0.08827	-0.10463	-0.12713	-0.14838	-0.17697	-0.19301	-0.18100	-0.07574						
2048	-0.07075	-0.08714	-0.10711	-0.12668	-0.15092	-0.18153	-0.19888	-0.22457	-0.21732	-0.13425							
1024	-0.10371	-0.12684	-0.15146	-0.17894	-0.20790	-0.23361	-0.24414	-0.24933	-0.15987								
512	-0.14227	-0.17304	-0.20580	-0.23144	-0.26718	-0.28051	-0.26340	-0.17629									
256	-0.19366	-0.22589	-0.25929	-0.28974	-0.30750	-0.29570	-0.20603										
128	-0.24267	-0.27618	-0.31544	-0.32727	-0.30190	-0.22878											
64	-0.29214	-0.32935	-0.34869	-0.33619	-0.26419												
32	-0.34253	-0.36875	-0.36166	-0.27619													
16	-0.37992	-0.38245	-0.33600														
8	-0.40741	-0.35756															
4	-0.38431																

(b) $\mathcal{P}^{\mathcal{C}}$ for Scene: [**Veach Door**] Algorithm: [**BDPT**] Metric: [**SC-DM**] True GT: [**BDPT @ 524288 spp**].

PSSMLT	2	4	8	16	32	64	128	256	512	1024	2048	4096	8192	16384
GT (BDPT)														
262144	-0.00093	-0.00140	-0.00165	-0.00168	-0.00241	-0.00336	-0.00439	-0.00576	-0.00646	-0.00669	-0.00703	-0.00234	0.00589	0.02927
131072	-0.00282	-0.00336	-0.00374	-0.00507	-0.00608	-0.00821	-0.01075	-0.01409	-0.01878	-0.02115	-0.02373	-0.02038	0.00223	0.04555
65536	-0.00567	-0.00735	-0.00910	-0.01112	-0.01391	-0.01889	-0.02511	-0.03038	-0.03641	-0.04081	-0.04231	-0.01390	0.03666	0.14725
32768	-0.01102	-0.01315	-0.01706	-0.02060	-0.02576	-0.03088	-0.04033	-0.04987	-0.05707	-0.06037	-0.04692	0.01504	0.15440	0.35450
16384	-0.01867	-0.02282	-0.02735	-0.03343	-0.04248	-0.05206	-0.06226	-0.07486	-0.08367	-0.07975	-0.03082	0.10434	0.34807	
8192	-0.03025	-0.03718	-0.04439	-0.05350	-0.06426	-0.07938	-0.09253	-0.10273	-0.10027	-0.05903	0.06214	0.31764		
4096	-0.04800	-0.05867	-0.06930	-0.08095	-0.09775	-0.11686	-0.13212	-0.13699	-0.10093	0.02537	0.27261			
2048	-0.07211	-0.08659	-0.10077	-0.11853	-0.13336	-0.14952	-0.15476	-0.13237	-0.02152	0.22000				
1024	-0.10904	-0.12648	-0.14239	-0.16261	-0.17550	-0.18062	-0.15300	-0.04962	0.18500					
512	-0.14804	-0.16679	-0.18457	-0.19752	-0.19381	-0.16316	-0.05027	0.16321						
256	-0.18900	-0.21095	-0.22365	-0.22121	-0.18110	-0.08488	0.12143							
128	-0.22985	-0.23986	-0.23016	-0.19865	-0.09769	0.10411								
64	-0.26838	-0.26110	-0.22674	-0.12891	0.06454									
32	-0.29247	-0.25202	-0.15393	0.02515										
16	-0.28714	-0.18171	0.00577											
8	-0.24851	-0.06738												
4	-0.15403													

(c) $\mathcal{P}^{\mathcal{C}}$ for Scene: [**Veach Door**] Algorithm: [**PSSMLT**] Metric: [**SC-DM**] True GT: [**BDPT @ 524288 spp**].

MLT	2	4	8	16	32	64	128	256	512	1024	2048	4096	8192	16384
GT (BDPT)														
262144	-0.00138	-0.00139	-0.00190	-0.00191	-0.00254	-0.00335	-0.00434	-0.00525	-0.00622	-0.00724	-0.00877	-0.00570	0.00631	0.02942
131072	-0.00316	-0.00414	-0.00527	-0.00663	-0.00783	-0.00901	-0.01093	-0.01413	-0.01750	-0.01870	-0.01366	-0.00450	0.02045	0.07314
65536	-0.00712	-0.00877	-0.01047	-0.01268	-0.01542	-0.01914	-0.02481	-0.03039	-0.03279	-0.03665	-0.02892	0.01221	0.08648	0.21519
32768	-0.01301	-0.01528	-0.01860	-0.02310	-0.02619	-0.03347	-0.04279	-0.05177	-0.05126	-0.03935	-0.00984	0.08543	0.23087	0.44888
16384	-0.02169	-0.02601	-0.03171	-0.03857	-0.04402	-0.05248	-0.06125	-0.06739	-0.05721	-0.04266	0.04478	0.20684	0.45345	
8192	-0.03593	-0.04280	-0.05106	-0.06063	-0.07024	-0.07850	-0.08526	-0.08762	-0.05379	0.01835	0.18275	0.48654		
4096	-0.05469	-0.06504	-0.07731	-0.08842	-0.09969	-0.10510	-0.11058	-0.08604	-0.00602	0.16563	0.46950			
2048	-0.08155	-0.09350	-0.11182	-0.12366	-0.13100	-0.13652	-0.10916	-0.03057	0.13263	0.41699				
1024	-0.11757	-0.13349	-0.15206	-0.16801	-0.16390	-0.13276	-0.05834	0.11552	0.40464					
512	-0.15711	-0.17553	-0.19695	-0.18826	-0.14690	-0.07181	0.11649	0.42141						
256	-0.20242	-0.21919	-0.21732	-0.19377	-0.10931	0.06948	0.34729							
128	-0.23822	-0.24790	-0.21484	-0.13646	0.03564	0.31493								
64	-0.27250	-0.25221	-0.17917	-0.02091	0.24769									
32	-0.29161	-0.22239	-0.06465	0.18416										
16	-0.28134	-0.14984	0.10989											
8	-0.22198	-0.00499												
4	-0.10593													

(d) $\mathcal{P}^{\mathcal{C}}$ for Scene: [**Veach Door**] Algorithm: [**MLT**] Metric: [**SC-DM**] True GT: [**BDPT @ 524288 spp**].

M-MLT	2	4	8	16	32	64	128	256	512	1024	2048	4096	8192	16384
GT (BDPT)														
262144	-0.00121	-0.00135	-0.00196	-0.00258	-0.00337	-0.00402	-0.00458	-0.00664	-0.00558	-0.00710	-0.00348	-0.00184	0.01299	0.02615
131072	-0.00335	-0.00396	-0.00477	-0.00601	-0.00739	-0.00983	-0.01229	-0.01645	-0.01795	-0.02019	-0.01577	-0.00173	0.02303	0.06170
65536	-0.00715	-0.00848	-0.01057	-0.01261	-0.01686	-0.02004	-0.02619	-0.03377	-0.03539	-0.03276	-0.02294	0.01352	0.10301	0.21065
32768	-0.01352	-0.01588	-0.01891	-0.02315	-0.02819	-0.03628	-0.03904	-0.04681	-0.05564	-0.04279	-0.01083	0.07968	0.25719	0.42497
16384	-0.02206	-0.02669	-0.03209	-0.03764	-0.04631	-0.05586	-0.06894	-0.07659	-0.07369	-0.04221	0.04393	0.20325	0.48170	
8192	-0.03684	-0.04280	-0.05183	-0.05891	-0.07025	-0.08300	-0.09853	-0.09510	-0.07270	0.01541	0.19203	0.47717		
4096	-0.05647	-0.06658	-0.07796	-0.09132	-0.10480	-0.11550	-0.11424	-0.09141	-0.00681	0.17265	0.48657			
2048	-0.08407	-0.09528	-0.10798	-0.12656	-0.13866	-0.14076	-0.11825	-0.03736	0.12759	0.43375				
1024	-0.11948	-0.13783	-0.15671	-0.16454	-0.16767	-0.14584	-0.06749	0.11460	0.40706					
512	-0.16240	-0.17763	-0.18825	-0.19391	-0.15853	-0.08263	0.09597	0.41883						
256	-0.20594	-0.21752	-0.22572	-0.19401	-0.10281	0.05397	0.35567							
128	-0.24482	-0.24369	-0.22378	-0.13177	0.03398	0.31943								
64	-0.28012	-0.25205	-0.16577	-0.01724	0.27355									
32	-0.28317	-0.21038	-0.06158	0.18643										
16	-0.27438	-0.13019	0.13032											
8	-0.20223	0.01828												
4	-0.07686													

(e) $\mathcal{P}^{\mathcal{C}}$ for Scene: [**Veach Door**] Algorithm: [**M-MLT**] Metric: [**SC-DM**] True GT: [**BDPT @ 524288 spp**].

ERPT	2	4	8	16	32	64	128	256	512	1024	2048	4096	8192	16384	32768	65536	131072
GT (BDPT)																	
262144	-0.00306	-0.00446	-0.00449	-0.00551	-0.00402	-0.00439	-0.00575	-0.00476	-0.00352	-0.00408	-0.00340	-0.00604	-0.00406	-0.00461	-0.00442	-0.00480	-0.00503
131072	-0.01028	-0.01213	-0.01132	-0.01196	-0.01221	-0.01271	-0.01246	-0.01315	-0.01199	-0.01183	-0.01121	-0.01278	-0.01276	-0.01357	-0.01267	-0.01234	
65536	-0.01953	-0.02328	-0.02374	-0.02539	-0.02546	-0.02759	-0.02651	-0.02601	-0.02732	-0.02886	-0.02613	-0.02664	-0.02531	-0.02529	-0.02742		
32768	-0.03329	-0.03726	-0.03977	-0.04280	-0.04125	-0.04364	-0.04611	-0.04595	-0.04495	-0.04665	-0.04584	-0.04412	-0.04346	-0.04772			
16384	-0.04921	-0.05746	-0.06484	-0.06626	-0.06462	-0.06940	-0.06737	-0.06716	-0.06959	-0.06789	-0.06836	-0.06707	-0.06966				
8192	-0.07193	-0.08109	-0.08708	-0.08851	-0.09152	-0.09185	-0.09502	-0.09554	-0.09389	-0.09420	-0.09557	-0.09352					
4096	-0.08097	-0.08618	-0.09853	-0.10450	-0.11048	-0.10946	-0.11104	-0.10780	-0.10653	-0.11898	-0.10950						
2048	-0.06256	-0.07628	-0.08712	-0.09405	-0.10187	-0.09934	-0.10304	-0.09966	-0.09264	-0.10075							
1024	0.00011	0.00208	-0.00622	-0.00505	-0.02733	-0.01168	-0.02369	-0.02028	-0.01328								
512	0.17921	0.19408	0.18571	0.18198	0.17554	0.20484	0.19599	0.19636									
256	0.45175	0.45482	0.48630	0.47521	0.47741	0.49154	0.49465										
128	0.81973	0.85170	0.88342	0.88831	0.88113	0.90374											
64	1.21677	1.23904	1.29217	1.30787	1.30351												
32	1.62226	1.65925	1.71428	1.72828													
16	1.99164	2.04163	2.09728														
8	2.34664	2.39351															
4	2.66112																

(f) $\mathcal{P}^{\mathcal{C}}$ for Scene: [**Veach Door**] Algorithm: [**ERPT**] Metric: [**SC-DM**] True GT: [**BDPT @ 524288 spp**].

M-ERPT	2	4	8	16	32	64	128	256	512	1024	2048	4096	8192	16384	32768	65536	131072
GT (BDPT)																	
262144	-0.00497	-0.00469	-0.00478	-0.00475	-0.00499	-0.00451	-0.00613	-0.00598	-0.00460	-0.00486	-0.00502	-0.00492	-0.00446	-0.00563	-0.00449	-0.00470	-0.00567
131072	-0.01040	-0.01294	-0.01170	-0.01191	-0.01319	-0.01336	-0.01208	-0.01449	-0.01264	-0.01175	-0.01243	-0.01225	-0.01246	-0.01423	-0.01406	-0.01359	
65536	-0.02365	-0.02702	-0.02745	-0.02557	-0.02749	-0.02899	-0.02881	-0.02906	-0.02978	-0.02691	-0.02737	-0.02847	-0.02691	-0.02996	-0.02897		
32768	-0.03983	-0.04437	-0.04236	-0.04362	-0.04627	-0.04994	-0.04787	-0.04958	-0.04693	-0.04635	-0.04598	-0.04832	-0.04906	-0.04612			
16384	-0.05824	-0.06516	-0.06939	-0.06793	-0.07137	-0.06871	-0.07756	-0.07306	-0.07466	-0.07038	-0.07075	-0.07139	-0.07062				
8192	-0.08288	-0.09104	-0.09598	-0.09445	-0.09802	-0.10055	-0.10069	-0.10178	-0.10367	-0.09650	-0.09421	-0.09860					
4096	-0.09596	-0.11636	-0.11288	-0.11861	-0.12219	-0.12629	-0.12150	-0.12480	-0.12696	-0.12693	-0.12632						
2048	-0.10029	-0.11211	-0.11103	-0.11298	-0.11644	-0.12466	-0.12256	-0.11493	-0.12024	-0.12064							
1024	-0.04904	-0.04804	-0.04120	-0.05727	-0.04353	-0.05720	-0.04914	-0.05347	-0.06247								
512	0.11899	0.13278	0.12532	0.12913	0.13187	0.13222	0.12735	0.13668									
256	0.36851	0.39502	0.40551	0.39390	0.41690	0.41299	0.41964										
128	0.73139	0.76931	0.79990	0.80284	0.81980	0.81914											
64	1.12135	1.17630	1.20641	1.21194	1.23626												
32	1.52616	1.59292	1.63052	1.63299													
16	1.90076	1.96888	2.01220														
8	2.26235	2.33517															
4	2.57900																

(g) $\mathcal{P}^{\mathcal{C}}$ for Scene: [**Veach Door**] Algorithm: [**M-ERPT**] Metric: [**SC-DM**] True GT: [**BDPT @ 524288 spp**].

Table 34

8.5 Sponza

PT	2	4	8	16	32	64	128	256	512	1024	2048	4096	8192
GT (PT)													
16384	-0.00350	-0.00432	-0.00622	-0.00716	-0.00783	-0.01109	-0.01529	-0.01305	-0.01435	-0.01098	0.01191	0.05466	0.14100
8192	-0.00983	-0.01180	-0.01418	-0.01810	-0.02240	-0.02619	-0.03342	-0.03740	-0.02683	-0.01056	0.04752	0.16746	
4096	-0.01923	-0.02334	-0.02880	-0.03463	-0.04215	-0.05182	-0.05973	-0.05444	-0.03861	0.02969	0.14704		
2048	-0.03350	-0.04020	-0.04971	-0.05992	-0.07176	-0.08435	-0.09028	-0.07912	-0.03065	0.10378			
1024	-0.05332	-0.06491	-0.07954	-0.09606	-0.10778	-0.11715	-0.11236	-0.07368	0.06070				
512	-0.08471	-0.10028	-0.12083	-0.14257	-0.15852	-0.16276	-0.12523	-0.01095					
256	-0.11870	-0.14409	-0.16670	-0.18791	-0.19017	-0.16173	-0.07168						
128	-0.17207	-0.20019	-0.22935	-0.23978	-0.21509	-0.11324							
64	-0.22522	-0.25128	-0.26942	-0.24871	-0.14607								
32	-0.26509	-0.27906	-0.27177	-0.20113									
16	-0.29937	-0.28961	-0.23129										
8	-0.29257	-0.24314											
4	-0.25333												

(a) $\mathcal{P}^{\mathcal{C}}$ for Scene: [**Sponza**] Algorithm: [**PT**] Metric: [**SC-DM**] True GT: [**PT @ 32768 spp**].

BDPT	2	4	8	16	32	64	128	256	512	1024	2048	4096	8192
GT (PT)													
16384	-0.00330	-0.00480	-0.00559	-0.00686	-0.00828	-0.01087	-0.01149	-0.01307	-0.01451	-0.01161	-0.00039	0.03402	0.09618
8192	-0.00864	-0.01162	-0.01440	-0.01670	-0.02225	-0.02587	-0.03076	-0.03453	-0.03687	-0.01464	0.02049	0.13580	
4096	-0.01707	-0.02282	-0.02692	-0.03451	-0.03976	-0.04746	-0.05546	-0.06182	-0.04643	-0.00597	0.11523		
2048	-0.02979	-0.03924	-0.04602	-0.05767	-0.06698	-0.07915	-0.08594	-0.08367	-0.05272	0.06326			
1024	-0.04810	-0.06421	-0.07697	-0.09094	-0.10354	-0.11656	-0.12099	-0.08800	0.00741				
512	-0.07789	-0.09718	-0.11890	-0.13989	-0.15557	-0.16389	-0.13936	-0.05386					
256	-0.11089	-0.13955	-0.16174	-0.18330	-0.19885	-0.18421	-0.10753						
128	-0.15811	-0.19526	-0.22058	-0.24357	-0.23764	-0.15997							
64	-0.20900	-0.24991	-0.26464	-0.27164	-0.20668								
32	-0.25090	-0.28875	-0.28983	-0.25249									
16	-0.28445	-0.29850	-0.27326										
8	-0.29335	-0.27334											
4	-0.26354												

(b) $\mathcal{P}^{\mathcal{C}}$ for Scene: [**Sponza**] Algorithm: [**BDPT**] Metric: [**SC-DM**] True GT: [**PT @ 32768 spp**].

PSSMLT	2	4	8	16	32	64	128	256	512	1024	2048	4096	8192
GT (PT)													
16384	-0.00317	-0.00340	-0.00423	-0.00543	-0.00664	-0.00872	-0.00876	-0.00907	-0.01086	-0.00768	-0.00671	-0.00208	-0.00015
8192	-0.00843	-0.01015	-0.01190	-0.01383	-0.01700	-0.02134	-0.02446	-0.02338	-0.02191	-0.01537	-0.00866	0.00221	
4096	-0.01617	-0.02013	-0.02362	-0.02731	-0.03326	-0.03803	-0.04242	-0.04167	-0.03435	-0.02558	-0.00967		
2048	-0.02724	-0.03399	-0.03956	-0.04733	-0.05516	-0.06282	-0.06560	-0.06030	-0.04510	-0.02643			
1024	-0.04454	-0.05510	-0.06351	-0.07321	-0.08524	-0.09502	-0.09212	-0.07266	-0.03242				
512	-0.07020	-0.08388	-0.09714	-0.11070	-0.12174	-0.12846	-0.11026	-0.06282					
256	-0.09917	-0.11809	-0.13203	-0.14586	-0.14611	-0.13056	-0.07519						
128	-0.14096	-0.15785	-0.17329	-0.17819	-0.14751	-0.09513							
64	-0.17912	-0.19290	-0.19509	-0.17329	-0.10666								
32	-0.20235	-0.20509	-0.18038	-0.11631									
16	-0.20774	-0.18353	-0.09263										
8	-0.17342	-0.11466											
4	-0.09393												

(c) $\mathcal{P}^{\mathcal{C}}$ for Scene: [**Sponza**] Algorithm: [**PSSMLT**] Metric: [**SC-DM**] True GT: [**PT @ 32768 spp**].

MLT	2	4	8	16	32	64	128	256	512	1024	2048	4096	8192
GT (PT)													
16384	-0.00367	-0.00404	-0.00533	-0.00597	-0.00797	-0.00857	-0.01045	-0.00574	-0.00382	-0.00451	0.00134	0.00031	0.00122
8192	-0.00993	-0.01174	-0.01377	-0.01737	-0.01843	-0.01999	-0.01990	-0.01862	-0.00931	-0.00479	0.00210	0.00320	
4096	-0.01883	-0.02237	-0.02617	-0.03171	-0.03463	-0.03910	-0.03718	-0.02766	-0.01138	-0.00013	0.01016		
2048	-0.03116	-0.03722	-0.04618	-0.05068	-0.05432	-0.05850	-0.04653	-0.03268	-0.00142	0.01732			
1024	-0.05208	-0.06058	-0.07369	-0.07926	-0.08035	-0.07715	-0.04108	-0.01221	0.04489				
512	-0.08103	-0.09237	-0.10802	-0.11152	-0.10740	-0.08428	-0.02380	0.04807					
256	-0.11238	-0.12658	-0.14233	-0.13056	-0.10635	-0.03303	0.08033						
128	-0.15940	-0.17129	-0.17597	-0.13921	-0.05710	0.08136							
64	-0.20004	-0.20251	-0.16659	-0.09234	0.08771								
32	-0.22345	-0.18906	-0.11384	0.03981									
16	-0.21555	-0.13217	0.03414										
8	-0.17557	-0.01326											
4	-0.07105												

(d) $\mathcal{P}^{\mathcal{C}}$ for Scene: [**Sponza**] Algorithm: [**MLT**] Metric: [**SC-DM**] True GT: [**PT @ 32768 spp**].

M-MLT	2	4	8	16	32	64	128	256	512	1024	2048	4096	8192
GT (PT)													
16384	-0.00393	-0.00496	-0.00531	-0.00656	-0.00801	-0.00844	-0.00858	-0.00519	-0.00677	-0.00207	0.00039	-0.00127	0.00134
8192	-0.01029	-0.01249	-0.01420	-0.01735	-0.01888	-0.02131	-0.01911	-0.01549	-0.01179	-0.00326	0.00337	0.00354	
4096	-0.01999	-0.02381	-0.02883	-0.03317	-0.03696	-0.03635	-0.03402	-0.01955	-0.01329	-0.00024	0.01152		
2048	-0.03468	-0.03984	-0.04722	-0.05313	-0.05911	-0.05573	-0.04774	-0.02070	-0.00395	0.01900			
1024	-0.05618	-0.06501	-0.07103	-0.08130	-0.08357	-0.07141	-0.05348	-0.00729	0.04456				
512	-0.08500	-0.09902	-0.10900	-0.11563	-0.11001	-0.07947	-0.02285	0.05642					
256	-0.11947	-0.12940	-0.13923	-0.12881	-0.10088	-0.02273	0.08286						
128	-0.16467	-0.17379	-0.17402	-0.13293	-0.05433	0.09865							
64	-0.20534	-0.19684	-0.15809	-0.06336	0.11095								
32	-0.22028	-0.18458	-0.10415	0.08775									
16	-0.21620	-0.12595	0.06111										
8	-0.14849	0.01017											
4	-0.03655												

(e) $\mathcal{P}^{\mathcal{C}}$ for Scene: [**Sponza**] Algorithm: [**M-MLT**] Metric: [**SC-DM**] True GT: [**PT @ 32768 spp**].

ERPT	2	4	8	16	32	64	128	256	512	1024	2048	4096	8192
GT (PT)													
16384	-0.00801	-0.00729	-0.00941	-0.00802	-0.00815	-0.00845	-0.00956	-0.00922	-0.00572	-0.00763	-0.00596	-0.00812	-0.00899
8192	-0.01632	-0.01458	-0.02084	-0.01555	-0.01635	-0.02037	-0.02061	-0.01723	-0.01979	-0.01899	-0.01778	-0.01664	
4096	-0.02908	-0.02923	-0.03573	-0.03091	-0.02818	-0.02899	-0.03278	-0.02952	-0.02963	-0.02968	-0.02994		
2048	-0.04247	-0.03871	-0.03999	-0.03818	-0.03695	-0.03734	-0.04049	-0.04438	-0.03756	-0.03926			
1024	-0.02479	-0.02501	-0.02997	-0.03272	-0.02492	-0.03309	-0.02835	-0.03399	-0.03512				
512	0.00220	0.00160	0.00683	0.00486	0.00605	0.01344	0.01421	0.00368					
256	0.10897	0.13107	0.13237	0.13079	0.14437	0.13797	0.14006						
128	0.35044	0.37291	0.36395	0.37967	0.37892	0.37629							
64	0.69251	0.70976	0.71360	0.73096	0.72700								
32	1.08508	1.10517	1.10827	1.12348									
16	1.51615	1.55094	1.54820										
8	1.90394	1.92802											
4	2.22878												

(f) $\mathcal{P}^{\mathcal{C}}$ for Scene: [**Sponza**] Algorithm: [**ERPT**] Metric: [**SC-DM**] True GT: [**PT @ 32768 spp**].

M-ERPT	2	4	8	16	32	64	128	256	512	1024	2048	4096	8192
GT (PT)													
16384	-0.00633	-0.00780	-0.00963	-0.00671	-0.00872	-0.00877	-0.00696	-0.00889	-0.00738	-0.00492	-0.00590	-0.00959	-0.00724
8192	-0.01704	-0.02056	-0.01574	-0.01776	-0.01806	-0.01892	-0.01748	-0.02042	-0.02105	-0.02013	-0.01702	-0.01708	
4096	-0.02901	-0.03286	-0.02601	-0.03121	-0.03550	-0.03549	-0.02853	-0.03580	-0.03437	-0.03634	-0.03377		
2048	-0.03834	-0.04559	-0.04251	-0.04112	-0.04060	-0.04330	-0.04762	-0.04411	-0.04172	-0.04286			
1024	-0.03426	-0.04893	-0.03347	-0.04192	-0.04357	-0.03814	-0.03361	-0.03827	-0.04321				
512	-0.00669	-0.00686	-0.00586	-0.00546	-0.00054	0.00089	-0.00261	-0.00220					
256	0.10676	0.10982	0.11356	0.10921	0.11891	0.11140	0.11446						
128	0.32694	0.34582	0.35655	0.35062	0.34168	0.35138							
64	0.66470	0.68394	0.69169	0.70299	0.69930								
32	1.04823	1.07630	1.08879	1.09592									
16	1.48929	1.51563	1.52569										
8	1.87424	1.90395											
4	2.20141												

(g) $\mathcal{P}^{\mathcal{C}}$ for Scene: [**Sponza**] Algorithm: [**M-ERPT**] Metric: [**SC-DM**] True GT: [**PT @ 32768 spp**].

Table 35

9 VSNR

9.1 Cornell Box

PT	2	4	8	16	32	64	128	256	512	1024	2048	4096	8192
GT (PT)													
16384	-0.00135	0.00502	-0.00087	-0.00077	0.00185	0.00165	-0.00063	-0.00053	-0.00432	-0.00315	-0.00324	-0.00475	-0.00329
8192	0.00482	0.00289	-0.00267	-0.00232	0.00061	0.00045	-0.00176	-0.00239	-0.00608	-0.00858	-0.01111	-0.01108	
4096	0.00066	-0.00069	-0.00096	-0.00143	0.00085	-0.00222	-0.00508	-0.00791	-0.01430	-0.01973	-0.02707		
2048	0.00090	-0.00113	-0.00240	-0.00339	-0.00423	-0.00735	-0.00982	-0.01718	-0.02534	-0.04011			
1024	-0.00622	-0.00226	-0.00479	-0.00642	-0.00845	-0.01186	-0.01993	-0.03390	-0.05335				
512	-0.00820	-0.00667	-0.01063	-0.01052	-0.01454	-0.02240	-0.03672	-0.05871					
256	-0.01170	-0.01401	-0.01654	-0.02228	-0.02774	-0.04638	-0.07000						
128	-0.02090	-0.02245	-0.02927	-0.03891	-0.05268	-0.08265							
64	-0.03275	-0.03360	-0.04886	-0.06698	-0.08914								
32	-0.05067	-0.05315	-0.07417	-0.10840									
16	-0.06558	-0.08063	-0.11774										
8	-0.08932	-0.13582											
4	-0.14488												

(a) $\mathcal{P}^{\mathcal{C}}$ for Scene: **[Cornell Box]** Algorithm: **[PT]** Metric: **[VSNR]** True GT: **[PT @ 32768 spp]**.

BDPT	2	4	8	16	32	64	128	256	512	1024	2048	4096	8192
GT (PT)													
16384	0.00413	-0.00071	-0.00085	0.00165	-0.00103	-0.00118	-0.00099	-0.00117	-0.00450	-0.00347	-0.00291	-0.00219	-0.00196
8192	0.00218	-0.00264	-0.00232	0.00051	-0.00200	-0.00242	-0.00354	-0.00697	-0.01040	-0.01439	-0.01113	-0.00828	
4096	-0.00058	-0.00154	-0.00465	-0.00218	-0.00444	-0.00497	-0.00767	-0.01557	-0.02313	-0.02998	-0.02769		
2048	-0.00175	-0.00279	-0.00375	-0.00436	-0.00991	-0.01203	-0.01806	-0.03149	-0.04820	-0.05792			
1024	-0.00345	-0.00571	-0.00819	-0.00841	-0.01565	-0.02260	-0.03788	-0.06271	-0.08563				
512	-0.00885	-0.01278	-0.01553	-0.01858	-0.03092	-0.04659	-0.07278	-0.11273					
256	-0.01262	-0.02003	-0.02860	-0.03728	-0.05759	-0.08428	-0.12727						
128	-0.02371	-0.03475	-0.04741	-0.06544	-0.10418	-0.14412							
64	-0.03980	-0.05598	-0.08158	-0.11298	-0.16748								
32	-0.06361	-0.08930	-0.13245	-0.18306									
16	-0.09726	-0.14386	-0.20218										
8	-0.15477	-0.22094											
4	-0.23815												

(b) $\mathcal{P}^{\mathcal{C}}$ for Scene: **[Cornell Box]** Algorithm: **[BDPT]** Metric: **[VSNR]** True GT: **[PT @ 32768 spp]**.

PSSMLT	2	4	8	16	32	64	128	256	512	1024	2048	4096	8192
GT (PT)													
16384	0.00443	-0.00081	0.00261	-0.00060	-0.00068	-0.00093	-0.00108	-0.00111	-0.00467	-0.00189	-0.00356	-0.00403	-0.00357
8192	0.00235	0.00144	0.00081	0.00024	-0.00032	-0.00292	-0.00317	-0.00404	-0.00762	-0.00886	-0.00999	-0.01067	
4096	-0.00082	-0.00129	-0.00172	0.00088	-0.00244	-0.00517	-0.00560	-0.01136	-0.01675	-0.02219	-0.02383		
2048	-0.00194	-0.00289	-0.00046	-0.00431	-0.00779	-0.01080	-0.01725	-0.02527	-0.03440	-0.04107			
1024	-0.00344	-0.00575	-0.00715	-0.00883	-0.01335	-0.02390	-0.03620	-0.05072	-0.06590				
512	-0.00935	-0.00895	-0.01185	-0.01849	-0.02981	-0.04660	-0.06529	-0.09316					
256	-0.01352	-0.02037	-0.02559	-0.03412	-0.05427	-0.08526	-0.11845						
128	-0.02426	-0.03470	-0.04332	-0.06471	-0.09909	-0.14280							
64	-0.04078	-0.05322	-0.07728	-0.11024	-0.16047								
32	-0.06197	-0.09008	-0.12615	-0.17491									
16	-0.09549	-0.13932	-0.19493										
8	-0.15329	-0.21489											
4	-0.23749												

(c) $\mathcal{P}^{\mathcal{C}}$ for Scene: **[Cornell Box]** Algorithm: **[PSSMLT]** Metric: **[VSNR]** True GT: **[PT @ 32768 spp]**.

MLT	2	4	8	16	32	64	128	256	512	1024	2048	4096	8192
GT (PT)													
16384	-0.00178	0.00308	-0.00110	-0.00089	-0.00077	-0.00080	0.00185	-0.00103	-0.00105	-0.00052	-0.00028	-0.00070	-0.00139
8192	0.00157	0.00044	0.00290	0.00182	0.00106	-0.00231	0.00031	-0.00234	-0.00467	-0.00294	-0.00615	-0.00692	
4096	0.00292	-0.00388	-0.00043	-0.00104	-0.00147	-0.00483	-0.00224	-0.00502	-0.00977	-0.00965	-0.01501		
2048	-0.00134	-0.00335	-0.00104	-0.00218	-0.00296	-0.00376	-0.00691	-0.01047	-0.01576	-0.02150			
1024	-0.00240	-0.00234	-0.00263	-0.00498	-0.00649	-0.01075	-0.01140	-0.02044	-0.03194				
512	-0.01228	-0.01302	-0.00731	-0.01128	-0.01371	-0.01906	-0.02448	-0.03866					
256	-0.02037	-0.01667	-0.01546	-0.01688	-0.02260	-0.03405	-0.04615						
128	-0.02155	-0.02644	-0.02320	-0.02835	-0.03584	-0.05335							
64	-0.03332	-0.03167	-0.03857	-0.04915	-0.06584								
32	-0.05069	-0.04926	-0.05512	-0.07204									
16	-0.05316	-0.06347	-0.08020										
8	-0.06511	-0.09451											
4	-0.10060												

(d) $\mathcal{P}^{\mathcal{C}}$ for Scene: **[Cornell Box]** Algorithm: **[MLT]** Metric: **[VSNR]** True GT: **[PT @ 32768 spp]**.

M-MLT	2	4	8	16	32	64	128	256	512	1024	2048	4096	8192
GT (PT)													
16384	-0.00167	-0.00129	0.00492	-0.00084	-0.00068	0.00228	-0.00084	-0.00107	-0.00099	-0.00117	-0.00147	-0.00124	-0.00389
8192	0.00161	-0.00384	0.00280	-0.00271	-0.00240	0.00077	-0.00216	-0.00001	-0.00265	-0.00294	-0.00747	-0.00772	
4096	0.00269	0.00038	-0.00053	-0.00559	-0.00141	0.00125	-0.00459	-0.00278	-0.00496	-0.01024	-0.01564		
2048	-0.00171	-0.00788	-0.00158	-0.00723	-0.00343	-0.00381	-0.00442	-0.00773	-0.01386	-0.02269			
1024	-0.00295	-0.00699	-0.00279	-0.00964	-0.00682	-0.00797	-0.01239	-0.01761	-0.02897				
512	-0.01301	-0.00985	-0.00793	-0.01182	-0.01390	-0.01330	-0.02414	-0.03537					
256	-0.01574	-0.01439	-0.01561	-0.02139	-0.02182	-0.02996	-0.04614						
128	-0.02424	-0.02463	-0.02493	-0.03548	-0.03955	-0.05695							
64	-0.03687	-0.03901	-0.03469	-0.04928	-0.06483								
32	-0.04596	-0.05082	-0.05912	-0.08082									
16	-0.05962	-0.07417	-0.08912										
8	-0.08390	-0.09763											
4	-0.10099												

(e) $\mathcal{P}^{\mathcal{C}}$ for Scene: **[Cornell Box]** Algorithm: **[M-MLT]** Metric: **[VSNR]** True GT: **[PT @ 32768 spp]**.

ERPT	2	4	8	16	32	64	128	256	512	1024	2048	4096	8192
GT (PT)													
16384	-0.00065	-0.00086	-0.00034	-0.00084	-0.00066	-0.00056	-0.00089	-0.00079	-0.00082	-0.00083	-0.00058	-0.00076	-0.00081
8192	-0.00185	-0.00211	-0.00185	-0.00200	-0.00192	0.00068	-0.00237	-0.00214	0.00015	-0.00220	-0.00167	-0.00248	
4096	-0.00408	-0.00239	-0.00184	-0.00179	-0.00447	-0.00181	-0.00458	-0.00459	0.00058	-0.00452	-0.00197		
2048	-0.00678	-0.00426	-0.00381	-0.00408	-0.00387	-0.00645	-0.00427	-0.00399	-0.00414	-0.00413			
1024	-0.01371	-0.01096	-0.01033	-0.01077	-0.01354	-0.01172	-0.01410	-0.01417	-0.01189				
512	-0.02613	-0.02300	-0.02611	-0.02399	-0.02636	-0.02690	-0.02651	-0.02620					
256	-0.04733	-0.04611	-0.04635	-0.04650	-0.04808	-0.04846	-0.04540						
128	-0.08547	-0.08553	-0.08527	-0.08541	-0.08436	-0.08556							
64	-0.14266	-0.14081	-0.14057	-0.13769	-0.13666								
32	-0.21807	-0.21876	-0.22265	-0.22053									
16	-0.31981	-0.32274	-0.32529										
8	-0.44837	-0.45520											
4	-0.59473												

(f) $\mathcal{P}^{\mathcal{C}}$ for Scene: **[Cornell Box]** Algorithm: **[ERPT]** Metric: **[VSNR]** True GT: **[PT @ 32768 spp]**.

M-ERPT	2	4	8	16	32	64	128	256	512	1024	2048	4096	8192
GT (PT)													
16384	-0.00062	-0.00067	-0.00062	-0.00103	-0.00073	-0.00114	-0.00088	-0.00079	-0.00074	-0.00047	-0.00059	0.00199	-0.00043
8192	-0.00223	-0.00186	-0.00211	-0.00262	-0.00211	-0.00225	-0.00238	-0.00219	-0.00182	-0.00162	-0.00214	0.00051	
4096	-0.00511	-0.00445	-0.00480	-0.00503	-0.00196	-0.00453	-0.00421	-0.00441	-0.00457	-0.00461	-0.00465		
2048	-0.00731	-0.00695	-0.00939	-0.00504	-0.00473	-0.00707	-0.00704	-0.00614	-0.00644	-0.00446			
1024	-0.01363	-0.01372	-0.01454	-0.01436	-0.01221	-0.01396	-0.01407	-0.01455	-0.01408				
512	-0.02673	-0.02706	-0.02588	-0.02640	-0.02435	-0.02606	-0.02781	-0.02576					
256	-0.04595	-0.04664	-0.04935	-0.04542	-0.04677	-0.04692	-0.04969						
128	-0.08335	-0.08150	-0.08590	-0.08564	-0.08208	-0.08502							
64	-0.14137	-0.14012	-0.13999	-0.13964	-0.14181								
32	-0.21977	-0.22117	-0.21820	-0.21706									
16	-0.31752	-0.32374	-0.31729										
8	-0.45030	-0.45141											
4	-0.59413												

(g) $\mathcal{P}^{\mathcal{C}}$ for Scene: **[Cornell Box]** Algorithm: **[M-ERPT]** Metric: **[VSNR]** True GT: **[PT @ 32768 spp]**.

Table 36

9.2 Torus

PT	2	4	8	16	32	64	128	256	512	1024	2048	4096	8192	16384	32768	65536	131072
GT (BDPT)																	
262144	-0.02052	0.00935	-0.01905	-0.02095	-0.00443	0.01370	-0.02379	0.00820	0.00464	-0.00037	-0.00310	-0.00436	-0.00554	-0.00563	-0.01532	-0.02572	-0.03372
131072	-0.02309	0.00265	-0.00063	-0.00963	-0.00449	0.01069	-0.02426	-0.01251	-0.00689	-0.01181	-0.00674	-0.01256	-0.01859	-0.02609	-0.04589	-0.06329	
65536	-0.03296	-0.01036	-0.01258	-0.00404	-0.01131	0.00477	-0.03140	-0.01028	-0.02151	-0.02008	-0.02207	-0.02845	-0.04389	-0.05228	-0.08759		
32768	-0.04495	-0.02491	-0.00806	-0.01505	-0.01660	0.00033	-0.03547	-0.02782	-0.04362	-0.04706	-0.04060	-0.05931	-0.08153	-0.11038			
16384	-0.03618	-0.00138	-0.00898	-0.00498	0.00240	0.00983	-0.02540	-0.04763	-0.05618	-0.05536	-0.06919	-0.09262	-0.13161				
8192	-0.03427	-0.00087	0.03176	0.03158	0.01872	0.02696	-0.03826	-0.04991	-0.04962	-0.08018	-0.10304	-0.15502					
4096	0.01390	0.04051	0.06851	0.06571	0.10251	0.07609	0.00904	-0.02041	-0.06041	-0.10236	-0.15228						
2048	0.08687	0.10486	0.12983	0.13963	0.16615	0.13842	0.06823	-0.00065	-0.06562	-0.12356							
1024	0.17545	0.17924	0.20505	0.24789	0.27624	0.23154	0.13942	0.03485	-0.05472								
512	0.26857	0.26775	0.26486	0.32619	0.36260	0.28972	0.21083	0.09982									
256	0.36468	0.34244	0.33132	0.40505	0.44542	0.36151	0.26441										
128	0.42442	0.40021	0.39106	0.44038	0.44788	0.37185											
64	0.47688	0.40863	0.42389	0.45660	0.45397												
32	0.49481	0.44063	0.44372	0.44477													
16	0.48414	0.41829	0.42373														
8	0.45465	0.39449															
4	0.38217																

(a) $\mathcal{P}^{\mathcal{C}}$ for Scene: [**Torus**] Algorithm: [**PT**] Metric: [**VSNR**] True GT: [**BDPT @ 524288 spp**].

BDPT	2	4	8	16	32	64	128	256	512	1024	2048	4096	8192	16384	32768	65536	131072
GT (BDPT)																	
262144	-0.00470	-0.01917	0.01050	0.01267	0.01603	-0.02356	0.01252	-0.00513	0.00305	-0.00108	-0.01234	-0.00393	-0.00526	-0.00695	-0.01075	-0.02554	-0.03091
131072	-0.00910	-0.02522	0.00606	0.00910	0.01766	-0.02488	0.00786	-0.01096	-0.00584	-0.01324	-0.01600	-0.01878	-0.01880	-0.02748	-0.03753	-0.06426	
65536	-0.01982	-0.01266	-0.00373	-0.00001	0.01131	-0.02940	-0.00173	-0.02311	-0.02254	-0.01842	-0.02793	-0.03221	-0.04172	-0.05090	-0.08415		
32768	-0.03260	-0.03340	0.01026	-0.00987	0.00875	-0.03416	-0.01135	-0.03745	-0.02571	-0.03493	-0.05098	-0.06490	-0.07970	-0.10721			
16384	-0.02382	-0.03173	0.01745	0.00719	0.03137	-0.01028	-0.00590	-0.03511	-0.05755	-0.05693	-0.07955	-0.10178	-0.12236				
8192	-0.02087	-0.01359	0.04595	0.04993	0.06147	0.00881	-0.02253	-0.03301	-0.07021	-0.07804	-0.10271	-0.14767					
4096	0.02450	0.03953	0.10212	0.08880	0.10529	0.05961	0.02506	-0.03107	-0.05915	-0.08245	-0.15053						
2048	0.07796	0.08276	0.18178	0.18963	0.19533	0.12883	0.09735	0.01248	-0.06952	-0.11058							
1024	0.17192	0.16707	0.24228	0.28543	0.27590	0.23692	0.13487	0.05663	-0.06149								
512	0.27364	0.23608	0.32512	0.36544	0.39128	0.29234	0.19812	0.11555									
256	0.37661	0.30539	0.40446	0.44134	0.46131	0.35824	0.26756										
128	0.43791	0.37105	0.45571	0.50701	0.50387	0.38513											
64	0.48056	0.41101	0.48354	0.49603	0.47632												
32	0.49964	0.44941	0.49014	0.49290													
16	0.48714	0.42564	0.44188														
8	0.46904	0.40666															
4	0.38235																

(b) $\mathcal{P}^{\mathcal{C}}$ for Scene: [**Torus**] Algorithm: [**BDPT**] Metric: [**VSNR**] True GT: [**BDPT @ 524288 spp**].

PSSMLT	2	4	8	16	32	64	128	256	512	1024	2048	4096	8192	16384
GT (BDPT)														
262144	-0.02739	0.01796	-0.00465	0.01086	-0.01813	0.00281	0.00046	-0.00232	-0.00237	-0.00335	-0.00278	-0.00334	-0.00511	-0.00368
131072	-0.02249	0.01665	-0.00548	0.00596	-0.02534	-0.00669	0.00416	-0.01369	-0.00484	-0.00821	-0.01618	-0.01205	-0.02294	-0.01716
65536	-0.01502	0.01233	-0.01472	-0.00413	-0.01742	-0.00616	-0.00090	-0.01169	-0.01617	-0.02044	-0.02980	-0.03011	-0.04903	-0.04104
32768	-0.05358	0.00723	-0.02127	0.00923	-0.03322	-0.02744	-0.02255	-0.02472	-0.03443	-0.03590	-0.05274	-0.06681	-0.09096	-0.08222
16384	-0.04826	-0.00671	-0.00537	-0.01352	-0.04011	-0.02817	-0.03327	-0.05077	-0.06259	-0.07298	-0.09739	-0.12073	-0.15875	
8192	0.00026	0.02561	0.01528	0.02581	-0.02070	-0.03804	-0.03919	-0.06495	-0.09878	-0.11999	-0.16521	-0.19480		
4096	0.11744	0.10812	0.09197	0.07221	0.01134	-0.01863	-0.04770	-0.09447	-0.13555	-0.18433	-0.23854			
2048	0.19707	0.16703	0.12422	0.11487	0.02756	-0.03268	-0.07039	-0.13207	-0.19467	-0.26870				
1024	0.32330	0.24881	0.20004	0.16752	0.06902	-0.00329	-0.08614	-0.17715	-0.26391					
512	0.46598	0.35702	0.30411	0.27095	0.12130	0.01987	-0.07722	-0.18350						
256	0.55704	0.44523	0.34220	0.29662	0.14275	0.03253	-0.08685							
128	0.61456	0.46891	0.38495	0.32252	0.15817	0.01970								
64	0.58676	0.45257	0.36501	0.30888	0.11360									
32	0.59838	0.44324	0.32918	0.27683										
16	0.52265	0.37434	0.23201											
8	0.49646	0.32665												
4	0.42998													

(c) $\mathcal{P}^{\mathcal{C}}$ for Scene: [**Torus**] Algorithm: [**PSSMLT**] Metric: [**VSNR**] True GT: [**BDPT @ 524288 spp**].

MLT	2	4	8	16	32	64	128	256	512	1024	2048	4096	8192	16384
GT (BDPT)														
262144	0.00646	0.02739	-0.03375	-0.04039	0.00725	0.01184	0.00824	0.00614	0.00642	0.00586	0.00620	0.00579	0.00512	0.00226
131072	0.00910	0.05997	-0.02363	-0.02290	-0.00019	0.00917	0.00503	0.00047	0.00267	0.00148	0.00228	0.00225	0.01964	-0.00303
65536	0.00862	0.03377	-0.01203	-0.07703	-0.01074	0.00460	-0.00369	-0.00800	-0.00604	-0.00758	0.01497	-0.00505	0.01167	0.00048
32768	0.02104	0.05160	-0.04994	-0.02945	-0.02209	0.00076	0.01483	0.00254	0.00921	0.00590	0.00987	0.01053	0.02348	0.00370
16384	-0.00793	0.05844	-0.03425	0.00056	-0.01605	0.02262	0.02999	0.01588	0.02471	0.01859	0.04292	0.04225	0.03540	
8192	-0.04709	-0.06102	-0.02022	0.03856	-0.00305	0.02255	0.02599	0.02586	0.01995	0.01498	0.03797	0.03887		
4096	-0.18883	-0.17457	0.05772	0.07678	0.02768	0.05933	0.04579	0.02532	0.03951	0.03027	0.05459			
2048	-0.37557	-0.68707	0.16051	0.22561	0.05094	0.11790	0.05474	0.04633	0.04697	0.03725				
1024	-0.65146	-1.18979	0.30230	0.35456	0.06627	0.12698	0.05439	0.02559	0.04307					
512	-1.28784	-1.67452	0.42809	0.50934	0.10041	0.17379	0.08533	0.04298						
256	-2.41019	-1.05250	0.48484	0.56582	0.12047	0.19910	0.08172							
128	-2.83200	-0.95782	0.48224	0.56038	0.10516	0.17518								
64	-3.13895	-1.18795	0.48522	0.50033	0.05063									
32	-3.04854	-1.63145	0.44164	0.47009										
16	-1.58401	-3.02872	0.34577											
8	-1.27586	-1.64271												
4	-1.32869													

(d) $\mathcal{P}^{\mathcal{C}}$ for Scene: [**Torus**] Algorithm: [**MLT**] Metric: [**VSNR**] True GT: [**BDPT @ 524288 spp**].

M-MLT	2	4	8	16	32	64	128	256	512	1024	2048	4096	8192	16384
GT (BDPT)														
262144	0.00442	0.06920	0.01780	0.00062	0.00934	0.00353	-0.01613	0.00010	0.00057	0.00069	-0.00015	0.00097	-0.01157	0.00831
131072	0.00538	-0.17468	0.01782	0.01332	0.00489	-0.00575	-0.00726	-0.00719	-0.00715	-0.00711	-0.00736	-0.00538	-0.00560	0.00151
65536	0.03275	-0.33361	-0.02592	-0.03846	-0.00621	-0.01876	-0.02136	-0.00802	-0.00580	-0.00659	-0.00697	-0.00299	-0.00503	-0.01048
32768	0.01002	-0.26683	-0.02963	-0.01503	-0.01811	-0.01586	-0.02361	-0.01026	-0.02203	-0.00896	-0.00800	-0.00196	-0.01674	-0.01658
16384	0.00705	-0.22058	-0.03104	0.00434	-0.00892	-0.01920	-0.02726	-0.01674	-0.02567	-0.01143	-0.01137	-0.00278	-0.01966	
8192	-0.05599	0.18175	0.00190	0.03189	0.00296	-0.03438	-0.02831	-0.03350	-0.04340	-0.02799	-0.04094	-0.04185		
4096	-0.13262	0.63381	0.03032	0.10917	0.01971	-0.03170	-0.04251	-0.06133	-0.05746	-0.06957	-0.08182			
2048	-0.28832	1.05118	0.10314	0.21873	0.03077	-0.02916	-0.04724	-0.07409	-0.09747	-0.10821				
1024	-0.50683	1.39821	0.16736	0.32561	0.07658	-0.04546	-0.09111	-0.12059	-0.14853					
512	-0.95935	1.73491	0.24265	0.47182	0.13631	-0.00884	-0.06599	-0.11257						
256	-1.47025	1.93194	0.29579	0.52957	0.13877	-0.00863	-0.07634							
128	-1.57549	1.94811	0.24778	0.53672	0.12229	-0.02488								
64	-1.64383	1.98169	0.19861	0.51336	0.06361									
32	-1.39962	1.94621	0.14268	0.45943										
16	-1.02335	1.73066	-0.00070											
8	-0.89044	1.68954												
4	-0.84805													

(e) $\mathcal{P}^{\mathcal{C}}$ for Scene: [**Torus**] Algorithm: [**M-MLT**] Metric: [**VSNR**] True GT: [**BDPT @ 524288 spp**].

ERPT	2	4	8	16	32	64	128	256	512	1024	2048	4096	8192	16384	32768	65536	131072
GT (BDPT)																	
262144	0.00281	0.00104	0.00020	-0.00176	-0.00228	-0.01200	-0.00366	-0.00417	-0.00338	-0.00377	-0.00445	-0.00417	-0.00477	-0.00477	-0.00518	-0.00506	-0.00401
131072	-0.00477	-0.00772	-0.00958	-0.01148	-0.00137	-0.01433	-0.00618	-0.01582	-0.01783	-0.01722	-0.01830	-0.01603	-0.01997	-0.01840	-0.01847	-0.01808	
65536	-0.00553	-0.01127	-0.01457	-0.01818	-0.01823	-0.02195	-0.01689	-0.02838	-0.02888	-0.03657	-0.04167	-0.04081	-0.04323	-0.04173	-0.04226		
32768	-0.00380	-0.00301	-0.00859	-0.02026	-0.03311	-0.03632	-0.04057	-0.04815	-0.04960	-0.06581	-0.07288	-0.08156	-0.08538	-0.07841			
16384	0.00467	-0.01152	-0.01216	-0.03450	-0.03624	-0.05408	-0.06290	-0.08259	-0.09050	-0.11164	-0.12936	-0.13973	-0.14180				
8192	0.01862	-0.02032	-0.03332	-0.05478	-0.06184	-0.09402	-0.11548	-0.13735	-0.15066	-0.18637	-0.20959	-0.22320					
4096	0.03209	-0.01950	-0.05396	-0.08833	-0.12002	-0.14243	-0.16536	-0.21120	-0.23543	-0.26923	-0.29956						
2048	0.04328	-0.06548	-0.10600	-0.13766	-0.18253	-0.19711	-0.25172	-0.29298	-0.31968	-0.37600							
1024	0.02848	-0.09971	-0.12049	-0.20500	-0.23316	-0.25884	-0.32067	-0.39322	-0.41701								
512	0.05215	-0.10800	-0.14310	-0.23935	-0.25729	-0.29696	-0.35272	-0.42817									
256	0.04953	-0.13668	-0.15511	-0.23968	-0.28916	-0.30861	-0.38302										
128	0.05647	-0.13565	-0.15254	-0.27230	-0.32388	-0.33809											
64	0.01622	-0.18464	-0.20624	-0.31736	-0.35725												
32	-0.01163	-0.24803	-0.25252	-0.35318													
16	-0.08117	-0.32761	-0.34056														
8	-0.10023	-0.36964															
4	-0.16080																

(f) $\mathcal{P}^{\mathcal{C}}$ for Scene: [**Torus**] Algorithm: [**ERPT**] Metric: [**VSNR**] True GT: [**BDPT @ 524288 spp**].

M-ERPT	2	4	8	16	32	64	128	256	512	1024	2048	4096	8192	16384	32768	65536	131072
GT (BDPT)																	
262144	0.01109	0.00503	-0.00092	-0.00081	-0.00232	-0.00409	-0.00448	-0.00582	-0.00532	-0.00532	-0.00692	-0.00954	-0.00970	-0.01017	-0.00618	-0.00507	-0.00692
131072	0.00985	-0.00208	0.00190	0.00105	-0.01150	-0.00683	-0.01424	-0.01866	-0.02259	-0.01362	-0.02231	-0.02194	-0.02181	-0.02257	-0.02262	-0.01967	
65536	0.00423	0.00821	-0.00347	-0.00467	-0.02385	-0.01923	-0.02885	-0.03321	-0.04059	-0.03854	-0.04204	-0.04199	-0.04168	-0.04704	-0.04441		
32768	0.00120	-0.00529	-0.00929	-0.01548	-0.02997	-0.04988	-0.05744	-0.06327	-0.07144	-0.07360	-0.08617	-0.08659	-0.08506	-0.08651			
16384	0.02652	0.01908	-0.02152	-0.02412	-0.05660	-0.07226	-0.08931	-0.09822	-0.12192	-0.12556	-0.14165	-0.14414	-0.14439				
8192	0.05819	0.03014	-0.02397	-0.04909	-0.09044	-0.12213	-0.15308	-0.16652	-0.20534	-0.21313	-0.23066	-0.22648					
4096	0.11483	0.04161	-0.04149	-0.07250	-0.13755	-0.19675	-0.21427	-0.24766	-0.28717	-0.30369	-0.32349						
2048	0.12408	0.07804	-0.08434	-0.11785	-0.20525	-0.27658	-0.31326	-0.33882	-0.39317	-0.41121							
1024	0.13063	0.07123	-0.10674	-0.15905	-0.28446	-0.37209	-0.40178	-0.43585	-0.49292								
512	0.18999	0.08666	-0.10289	-0.18472	-0.30949	-0.40451	-0.44089	-0.48920									
256	0.17181	0.10544	-0.11307	-0.18785	-0.32152	-0.42555	-0.47607										
128	0.14354	0.09705	-0.12155	-0.22188	-0.34122	-0.44420											
64	0.05232	0.06913	-0.13767	-0.27217	-0.36748												
32	-0.02753	0.02563	-0.17272	-0.30138													
16	-0.14560	-0.08809	-0.24207														
8	-0.15570	-0.11924															
4	-0.22776																

(g) $\mathcal{P}^{\mathcal{C}}$ for Scene: [**Torus**] Algorithm: [**M-ERPT**] Metric: [**VSNR**] True GT: [**BDPT @ 524288 spp**].

Table 37

9.3 Veach Bidir

PT	2	4	8	16	32	64	128	256	512	1024	2048	4096	8192	16384	32768	65536	131072
GT (BDPT)																	
262144	0.00011	0.00003	0.00003	-0.00001	-0.00000	-0.00001	0.00005	-0.00005	0.00005	0.00012	0.00004	-0.00008	-0.00005	0.00014	-0.00005	0.00029	-0.00023
131072	-0.00282	-0.00189	0.01058	-0.00131	-0.00117	-0.00107	-0.00098	-0.00092	-0.00088	-0.00075	0.00533	-0.00092	-0.00073	-0.00046	-0.00060	-0.00064	
65536	0.00668	-0.00652	0.00703	0.00479	0.00355	-0.00352	-0.00335	-0.00295	-0.00280	-0.00244	0.00383	-0.00251	-0.00214	-0.00196	0.00117		
32768	0.00088	0.00747	0.00405	0.00210	0.00120	-0.00569	0.00011	-0.00468	0.00299	-0.00426	0.00210	-0.00390	-0.00343	0.00039			
16384	-0.00206	0.00565	0.00261	0.00083	0.00004	-0.00683	0.00411	-0.00568	0.00261	-0.00443	0.00164	-0.00429	-0.00425				
8192	-0.00948	-0.00304	0.00572	0.00123	-0.00124	-0.00872	0.00116	-0.00903	-0.00096	-0.00860	-0.00280	-0.00421					
4096	-0.00047	-0.00067	0.00558	-0.00891	-0.00310	-0.00490	-0.00687	-0.00762	-0.00071	-0.00868	-0.00261						
2048	-0.01435	-0.01674	-0.01075	-0.00769	-0.01165	-0.00840	-0.01132	-0.01371	-0.00709	-0.00885							
1024	-0.01694	-0.01842	-0.00049	-0.00854	-0.01231	-0.00902	-0.00687	-0.01386	-0.00674								
512	-0.04266	-0.02485	-0.01115	-0.01254	-0.01125	-0.01623	-0.01154	-0.01556									
256	-0.05602	-0.03037	-0.01331	-0.02003	-0.01639	-0.01773	-0.01601										
128	-0.06336	-0.04064	-0.03273	-0.02904	-0.03011	-0.02385											
64	-0.08431	-0.06476	-0.03789	-0.04437	-0.03940												
32	-0.09808	-0.07956	-0.05371	-0.05805													
16	-0.12471	-0.08381	-0.06394														
8	-0.11331	-0.08945															
4	-0.10998																

(a) $\mathcal{P}^{\mathcal{C}}$ for Scene: [**Veach Bidir**] Algorithm: [**PT**] Metric: [**VSNR**] True GT: [**BDPT @ 524288 spp**].

BDPT	2	4	8	16	32	64	128	256	512	1024	2048	4096	8192	16384	32768	65536	131072
GT (BDPT)																	
262144	-0.00009	-0.00002	-0.00005	0.00004	0.00002	-0.00002	0.00030	-0.00048	-0.00293	0.00344	-0.00756	-0.01481	-0.00165	-0.00488	0.00467	0.00264	-0.00031
131072	-0.00066	-0.00040	0.00338	-0.00043	-0.00059	-0.00007	-0.00033	0.00017	-0.00263	-0.00633	-0.01035	-0.00396	-0.00446	0.00297	0.00509	0.00187	
65536	-0.00244	-0.00190	0.00200	-0.00122	-0.00138	-0.00161	-0.00172	0.00020	-0.00037	-0.00078	-0.00280	-0.01217	-0.00688	-0.01196	0.00052		
32768	-0.00383	-0.00321	0.00097	-0.00241	-0.00270	-0.00307	-0.00222	-0.00171	0.00074	-0.00686	-0.01931	-0.02453	-0.03512	-0.03641			
16384	-0.00444	-0.00357	0.00085	-0.00281	-0.00108	0.00003	0.00367	0.00196	0.00283	-0.00060	-0.03170	-0.06212	-0.04749				
8192	-0.00758	-0.00720	0.00151	-0.00282	-0.00226	-0.00132	0.00179	-0.00640	-0.01343	-0.01438	-0.04413	-0.07809					
4096	-0.00616	-0.00682	-0.00355	-0.00441	-0.00859	-0.00686	-0.00687	-0.01120	-0.01741	-0.04423	-0.09721						
2048	-0.01160	-0.00769	-0.00567	-0.00680	-0.00550	-0.00934	-0.00686	-0.01903	0.01991	-0.05308							
1024	-0.01145	-0.00729	-0.00408	-0.00482	-0.00581	0.00288	0.00345	-0.03578	-0.01331								
512	-0.01180	-0.00980	-0.00752	-0.00872	-0.01854	-0.00224	-0.01992	-0.08979									
256	-0.01698	-0.01734	-0.01294	-0.01925	-0.02487	-0.01591	-0.04331										
128	-0.02821	-0.02712	-0.02563	-0.04173	-0.04412	-0.02861											
64	-0.04044	-0.03858	-0.04092	-0.06305	-0.08216												
32	-0.05554	-0.06150	-0.07528	-0.10651													
16	-0.07143	-0.08398	-0.10529														
8	-0.10024	-0.12684															
4	-0.14490																

(b) $\mathcal{P}^{\mathcal{C}}$ for Scene: [**Veach Bidir**] Algorithm: [**BDPT**] Metric: [**VSNR**] True GT: [**BDPT @ 524288 spp**].

PSSMLT	2	4	8	16	32	64	128	256	512	1024	2048	4096	8192	16384
GT (BDPT)														
262144	0.00007	0.00005	0.00004	0.00001	0.00010	0.00000	-0.00002	-0.00040	0.00070	0.00116	0.00607	0.00204	-0.00003	0.00096
131072	-0.00072	-0.00063	0.00370	-0.00048	-0.00045	-0.00043	0.00129	-0.00044	-0.00108	-0.00155	0.00445	-0.00083	-0.00165	-0.00083
65536	0.00152	-0.00217	0.00229	0.00156	-0.00139	-0.00153	0.00032	-0.00156	0.00066	0.00062	0.00365	-0.00028	-0.00118	-0.00129
32768	0.00405	0.00220	0.00126	0.00064	-0.00017	-0.00233	-0.00033	-0.00216	-0.00131	-0.00158	0.00078	-0.00862	-0.00640	-0.01050
16384	0.00349	0.00193	0.00114	0.00105	0.00071	-0.00037	0.00206	0.00183	-0.00345	-0.00759	-0.01760	-0.01793	-0.01986	
8192	0.00069	-0.00151	-0.00228	0.00108	-0.00097	-0.00043	0.00272	0.00467	0.00206	-0.00521	-0.01150	-0.02058		
4096	-0.00559	-0.00119	-0.00306	-0.00063	-0.00385	-0.00434	-0.00429	-0.01017	-0.02384	-0.02683	-0.04186			
2048	-0.01008	-0.00663	-0.00432	-0.00555	-0.00572	-0.00392	0.00017	-0.00282	-0.02918	-0.03353				
1024	-0.00603	-0.00600	-0.00272	0.00112	-0.00023	0.00431	0.01144	0.00658	-0.02474					
512	-0.00914	-0.00809	-0.00566	-0.00260	-0.00324	0.00351	0.01132	-0.01912						
256	-0.01341	-0.01528	-0.01016	-0.00853	-0.01448	-0.01212	-0.00971							
128	-0.02315	-0.02323	-0.02111	-0.02145	-0.02933	-0.03584								
64	-0.03209	-0.03256	-0.03463	-0.04280	-0.05878									
32	-0.05278	-0.05527	-0.06220	-0.08441										
16	-0.06560	-0.07829	-0.09636											
8	-0.09052	-0.11595												
4	-0.12619													

(c) $\mathcal{P}^{\mathcal{C}}$ for Scene: [**Veach Bidir**] Algorithm: [**PSSMLT**] Metric: [**VSNR**] True GT: [**BDPT @ 524288 spp**].

MLT	2	4	8	16	32	64	128	256	512	1024	2048	4096	8192	16384
GT (BDPT)														
262144	0.00008	0.00003	0.00005	-0.00000	-0.00000	-0.00007	-0.00006	-0.00008	-0.00001	-0.00008	0.00056	0.00058	0.00040	0.00032
131072	0.01921	-0.00112	-0.00075	0.00289	0.00473	-0.00038	-0.00030	0.00230	0.00182	-0.00030	-0.00039	0.00009	0.00001	-0.00026
65536	0.01423	-0.00418	-0.00292	0.00470	0.00334	0.00249	-0.00118	0.00186	0.00138	-0.00057	-0.00055	-0.00054	-0.00052	-0.00084
32768	0.00985	0.00248	-0.00471	0.00325	0.00224	0.00153	-0.00207	0.00086	0.00080	-0.00077	-0.00033	-0.00091	-0.00076	-0.00109
16384	0.00758	0.00122	-0.00569	0.00286	0.00159	0.00097	-0.00250	0.00090	0.00151	0.00053	-0.00041	0.00151	0.00268	
8192	-0.00087	-0.00684	-0.00756	-0.00016	-0.00153	0.00159	-0.00192	0.00184	0.00272	0.00190	0.00391	0.00454		
4096	0.00284	-0.00762	-0.00430	0.00081	-0.00157	0.00062	-0.00433	-0.00387	-0.00331	-0.00511	-0.00601			
2048	-0.01304	-0.01401	-0.01262	-0.00420	-0.00212	-0.00449	-0.00457	-0.00106	0.00139	0.00315				
1024	-0.01531	-0.01492	-0.00781	-0.00392	-0.00129	-0.00320	-0.00207	0.00446	0.01011					
512	-0.01977	-0.01720	-0.01432	-0.00495	-0.00423	-0.00275	-0.00582	0.00078						
256	-0.02254	-0.02327	-0.02020	-0.01030	-0.01137	-0.01166	-0.01126							
128	-0.04521	-0.03729	-0.03077	-0.02207	-0.02012	-0.01894								
64	-0.06428	-0.04794	-0.04191	-0.03336	-0.03151									
32	-0.07228	-0.05496	-0.05498	-0.04363										
16	-0.08192	-0.07254	-0.06865											
8	-0.08623	-0.07403												
4	-0.08463													

(d) $\mathcal{P}^{\mathcal{C}}$ for Scene: [**Veach Bidir**] Algorithm: [**MLT**] Metric: [**VSNR**] True GT: [**BDPT @ 524288 spp**].

M-MLT	2	4	8	16	32	64	128	256	512	1024	2048	4096	8192	16384
GT (BDPT)														
262144	0.00008	0.00005	0.00004	0.00005	0.00006	0.00010	0.00005	-0.00005	-0.00007	-0.00021	0.00001	-0.00010	-0.00016	0.00022
131072	-0.00194	-0.00119	-0.00087	-0.00062	0.00447	-0.00033	-0.00029	-0.00032	-0.00020	-0.00036	-0.00030	0.00089	-0.00027	-0.00019
65536	-0.00671	0.00487	-0.00312	-0.00240	0.00308	0.00219	0.00194	-0.00101	0.00090	-0.00109	-0.00135	-0.00007	-0.00128	-0.00122
32768	-0.01095	0.00231	-0.00491	0.00288	0.00187	0.00116	0.00078	-0.00195	0.00011	-0.00260	-0.00087	-0.00084	-0.00149	-0.00177
16384	-0.01300	0.00103	-0.00047	0.00268	0.00148	0.00093	0.00078	-0.00154	0.00092	0.00048	0.00255	0.00363	0.00461	
8192	-0.02151	0.00198	-0.00764	-0.00038	-0.00143	-0.00159	0.00135	-0.00105	0.00236	0.00069	0.00122	0.00311		
4096	-0.01843	0.00095	-0.00435	0.00057	-0.00204	-0.00307	-0.00100	-0.00398	-0.00383	-0.00520	-0.00504			
2048	-0.01550	-0.00599	-0.00775	-0.00458	-0.00265	-0.00460	-0.00223	-0.00377	0.00025	-0.00436				
1024	-0.01755	-0.00676	-0.00857	-0.00447	-0.00195	-0.00234	0.00182	0.00304	0.00994					
512	-0.02344	-0.01796	-0.01497	-0.00916	-0.00503	-0.00644	-0.00332	-0.00124						
256	-0.04530	-0.02410	-0.01597	-0.01166	-0.01230	-0.01101	-0.00756							
128	-0.05209	-0.03070	-0.03090	-0.02314	-0.02147	-0.02218								
64	-0.07399	-0.04252	-0.04310	-0.03399	-0.03275									
32	-0.08631	-0.05210	-0.04847	-0.05067										
16	-0.08671	-0.05833	-0.06125											
8	-0.10781	-0.07343												
4	-0.09173													

(e) $\mathcal{P}^{\mathcal{C}}$ for Scene: [**Veach Bidir**] Algorithm: [**M-MLT**] Metric: [**VSNR**] True GT: [**BDPT @ 524288 spp**].

ERPT	2	4	8	16	32	64	128	256	512	1024	2048	4096	8192	16384	32768	65536	131072
GT (BDPT)																	
262144	0.00011	-0.00002	0.00010	-0.00004	0.00001	-0.00005	0.00026	0.00017	0.00025	0.00011	0.00002	0.00004	0.00007	-0.00006	0.00002	0.00007	0.00009
131072	-0.00017	-0.00037	-0.00027	0.00248	-0.00043	0.00275	-0.00023	-0.00024	-0.00062	-0.00029	-0.00051	-0.00036	-0.00046	-0.00054	-0.00049	0.00270	
65536	-0.00139	0.00150	0.00139	0.00132	0.00142	0.00180	-0.00142	-0.00127	-0.00189	-0.00170	-0.00198	0.00137	-0.00196	-0.00191	-0.00185		
32768	0.00049	0.00035	0.00045	0.00071	0.00067	-0.00003	-0.00235	-0.00231	0.00034	-0.00245	-0.00298	0.00026	0.00020	0.00037			
16384	0.00033	0.00081	0.00101	0.00323	0.00063	-0.00015	-0.00268	-0.00399	-0.00068	-0.00360	-0.00333	-0.00089	-0.00010				
8192	-0.00171	0.00079	0.00098	0.00043	0.00045	-0.00417	-0.00284	-0.00176	-0.00322	-0.00397	-0.00411	-0.00396					
4096	-0.00123	-0.00111	-0.00419	-0.00142	-0.00422	-0.00155	-0.00461	-0.00343	-0.00753	-0.00904	-0.00595						
2048	-0.00597	-0.00531	-0.00157	-0.00246	-0.00180	-0.00736	-0.00772	-0.01223	-0.00874	-0.01515							
1024	-0.00275	0.00154	0.00223	0.00101	-0.00330	-0.01779	-0.00505	-0.01550	-0.01803								
512	-0.00231	-0.00091	-0.00096	-0.00262	-0.00863	-0.03496	-0.02011	-0.03055									
256	-0.00866	-0.00693	-0.00672	-0.00807	-0.02154	-0.04128	-0.03509										
128	-0.02320	-0.01939	-0.02415	-0.02038	-0.03691	-0.08060											
64	-0.04775	-0.04249	-0.04744	-0.04487	-0.06553												
32	-0.09261	-0.09524	-0.09663	-0.09847													
16	-0.15009	-0.14563	-0.15545														
8	-0.23462	-0.23589															
4	-0.34130																

(f) $\mathcal{P}^{\mathcal{C}}$ for Scene: [**Veach Bidir**] Algorithm: [**ERPT**] Metric: [**VSNR**] True GT: [**BDPT @ 524288 spp**].

M-ERPT	2	4	8	16	32	64	128	256	512	1024	2048	4096	8192	16384	32768	65536	131072
GT (BDPT)																	
262144	-0.00007	-0.00007	-0.00001	0.00035	0.00009	0.00019	-0.00026	0.00022	-0.00015	0.00002	0.00001	0.00009	0.00005	0.00003	-0.00000	-0.00007	0.00349
131072	-0.00039	-0.00033	0.00280	-0.00068	0.00276	-0.00039	-0.00064	-0.00045	-0.00056	-0.00051	-0.00038	0.00281	0.00291	-0.00045	-0.00037	0.00268	
65536	0.00122	-0.00151	0.00162	-0.00146	0.00171	-0.00169	0.00138	-0.00159	-0.00188	0.00137	-0.00170	0.00152	0.00152	-0.00177	-0.00162		
32768	0.00065	-0.00273	0.00037	-0.00274	0.00027	0.00004	0.00021	0.00028	0.00048	0.00033	0.00031	0.00014	0.00052	-0.00275			
16384	0.00034	-0.00257	-0.00009	-0.00345	0.00390	-0.00026	-0.00023	-0.00045	0.00006	-0.00082	-0.00079	-0.00076	-0.00071				
8192	0.00061	-0.00207	-0.00014	-0.00272	0.00102	-0.00331	-0.00537	-0.00314	-0.00441	-0.00416	-0.00460	-0.00426					
4096	-0.00422	-0.00383	-0.00303	-0.00922	-0.00144	-0.00639	-0.00311	-0.00627	-0.00627	-0.00692	-0.00634						
2048	-0.00251	-0.00564	-0.00845	-0.01173	-0.00717	-0.01284	-0.00718	-0.01247	-0.00959	-0.01324							
1024	-0.00016	-0.00129	-0.02003	-0.01169	-0.00640	-0.01918	-0.02081	-0.01972	-0.01913								
512	-0.00365	-0.00259	-0.04555	-0.03103	-0.02096	-0.04485	-0.03971	-0.04488									
256	-0.00774	-0.01090	-0.04760	-0.04886	-0.03395	-0.05193	-0.05131										
128	-0.02541	-0.02778	-0.09007	-0.08202	-0.07014	-0.09293											
64	-0.04929	-0.05265	-0.11953	-0.11506	-0.10445												
32	-0.09139	-0.09723	-0.14712	-0.14304													
16	-0.15102	-0.15057	-0.19713														
8	-0.22834	-0.23359															
4	-0.33696																

(g) $\mathcal{P}^{\mathcal{C}}$ for Scene: [**Veach Bidir**] Algorithm: [**M-ERPT**] Metric: [**VSNR**] True GT: [**BDPT @ 524288 spp**].

Table 38

9.4 Veach Door

PT	2	4	8	16	32	64	128	256	512	1024	2048	4096	8192	16384	32768	65536	131072
GT (BDPT)																	
262144	0.00160	0.01623	-0.00113	-0.00061	-0.00041	-0.00034	-0.00030	0.00803	-0.00021	-0.00017	-0.00018	-0.00016	-0.00048	-0.00252	-0.00026	-0.00061	-0.00195
131072	0.00390	0.03896	-0.00272	-0.00125	-0.00099	-0.00070	-0.00081	0.00767	-0.00054	-0.00046	0.00378	-0.00066	-0.00056	-0.00305	-0.00536	-0.00606	
65536	0.02224	0.18706	-0.01345	-0.00623	-0.00455	-0.00375	-0.00326	0.00541	0.00406	-0.00210	0.00176	-0.00222	-0.00245	-0.00589	-0.00833		
32768	0.05345	0.40215	0.00160	-0.00127	-0.01092	-0.00902	-0.00790	0.00135	0.00079	0.00005	-0.00087	-0.00519	-0.00565	-0.00937			
16384	0.03482	0.30330	-0.02779	-0.00146	-0.00292	-0.00378	-0.00411	0.00392	0.00183	0.00063	-0.00502	-0.00634	-0.01350				
8192	0.06657	0.51812	-0.02019	-0.00184	-0.00540	-0.00688	-0.00809	-0.00011	-0.00212	-0.00884	-0.00996	-0.01567					
4096	0.05816	0.52212	-0.03366	-0.01412	-0.00918	-0.01222	-0.01365	-0.00670	-0.00991	-0.01684	-0.02273						
2048	0.12064	0.60263	-0.06382	-0.03720	-0.03819	-0.02640	-0.02780	-0.02143	-0.02483	-0.02699							
1024	0.14702	0.82681	-0.09657	-0.05411	-0.04295	-0.03705	-0.03329	-0.03028	-0.04389								
512	0.12324	1.04012	-0.11765	-0.06229	-0.04726	-0.04792	-0.04986	-0.05253									
256	0.11322	0.90716	-0.14747	-0.06570	-0.06510	-0.06305	-0.07995										
128	0.01033	0.37909	-0.06270	-0.06073	-0.08243	-0.08823											
64	-0.35137	-0.13432	0.03861	-0.05064	-0.09753												
32	-1.56633	1.57883	0.16854	-0.02190													
16	-0.35733	2.46776	0.39968														
8	0.71116	3.07464															
4	1.24258																

(a) $\mathcal{P}^{\mathcal{C}}$ for Scene: [**Veach Door**] Algorithm: [**PT**] Metric: [**VSNR**] True GT: [**BDPT @ 524288 spp**].

BDPT	2	4	8	16	32	64	128	256	512	1024	2048	4096	8192	16384	32768	65536	131072
GT (BDPT)																	
262144	0.34794	-0.00107	-0.00053	-0.00023	-0.00031	-0.00014	-0.00010	-0.00027	-0.00025	-0.00013	-0.00053	-0.00033	-0.00070	-0.00067	0.00019	0.00207	0.00138
131072	0.63727	-0.00241	-0.00110	-0.00066	-0.00063	-0.00049	-0.00038	-0.00054	-0.00055	-0.00049	-0.00082	-0.00124	-0.00354	-0.00386	0.00050	0.00305	
65536	2.02713	-0.01256	-0.00568	0.00945	-0.00300	-0.00238	0.00275	-0.00183	-0.00229	-0.00241	-0.00297	-0.00829	-0.01449	-0.01163	-0.00192		
32768	0.19787	-0.03062	-0.00156	0.00429	0.00202	-0.00559	-0.00006	-0.00507	-0.00495	-0.00523	-0.01193	-0.01657	-0.02904	-0.02546			
16384	1.43328	-0.02629	-0.00191	-0.00347	-0.00424	-0.00466	-0.00478	-0.00852	-0.00907	-0.01043	-0.01720	-0.03860	-0.05052				
8192	1.13479	-0.05181	-0.00325	-0.00661	-0.00786	-0.00835	-0.00398	-0.01060	-0.01794	-0.02548	-0.03998	-0.06898					
4096	1.15815	-0.03257	-0.01598	-0.01130	-0.00576	-0.01618	-0.01208	-0.01880	-0.03302	-0.04516	-0.07933						
2048	2.94192	-0.09392	-0.03734	-0.02414	-0.01959	-0.02362	-0.03247	-0.03993	-0.05890	-0.08854							
1024	3.35753	-0.09217	-0.05717	-0.03461	-0.03477	-0.04349	-0.05368	-0.07333	-0.10851								
512	3.02612	-0.11598	-0.06855	-0.04551	-0.04482	-0.06586	-0.08356	-0.11869									
256	3.18689	-0.12105	-0.07533	-0.06634	-0.07510	-0.10239	-0.13834										
128	1.45885	-0.08983	-0.07773	-0.08190	-0.10880	-0.15937											
64	3.67810	-0.00215	-0.07353	-0.11359	-0.16410												
32	4.91891	0.15897	-0.05203	-0.15650													
16	5.60988	0.34236	-0.02212														
8	6.17863	0.57916															
4	6.53878																

(b) $\mathcal{P}^{\mathcal{C}}$ for Scene: [**Veach Door**] Algorithm: [**BDPT**] Metric: [**VSNR**] True GT: [**BDPT @ 524288 spp**].

PSSMLT	2	4	8	16	32	64	128	256	512	1024	2048	4096	8192	16384
GT (BDPT)														
262144	0.02342	-0.00058	-0.00036	-0.00021	-0.00020	-0.00018	-0.00015	-0.00016	-0.00023	-0.00022	0.00022	-0.00063	-0.00394	-0.00166
131072	0.02239	-0.00107	-0.00077	-0.00055	-0.00057	-0.00038	-0.00039	-0.00022	-0.00028	-0.00056	-0.00677	-0.00697	-0.01337	-0.00615
65536	0.01432	0.01665	-0.00370	-0.00282	0.00453	-0.00198	-0.00166	-0.00156	-0.00149	-0.00188	-0.00805	-0.01453	-0.02309	-0.01222
32768	0.02435	0.00940	-0.00881	-0.00701	0.00113	0.00011	-0.00039	-0.00444	-0.00497	-0.01177	-0.01855	-0.03723	-0.04888	-0.03751
16384	0.00265	-0.00211	-0.00344	-0.00388	-0.00406	0.00117	-0.00445	-0.00823	-0.01203	-0.01492	-0.03461	-0.06627	-0.08696	
8192	0.00624	-0.00392	-0.00677	-0.00770	-0.00097	-0.00280	-0.00799	-0.01276	-0.02362	-0.03712	-0.07099	-0.11519		
4096	0.01677	-0.00460	-0.01112	-0.01302	-0.00749	-0.00962	-0.02001	-0.02545	-0.04767	-0.07663	-0.12326			
2048	-0.03327	-0.01664	-0.02485	-0.02787	-0.02145	-0.02354	-0.03881	-0.05151	-0.08344	-0.12656				
1024	-0.04162	-0.03732	-0.03517	-0.03327	-0.03275	-0.04376	-0.06561	-0.09348	-0.14344					
512	-0.04942	-0.03183	-0.04507	-0.05031	-0.05186	-0.07175	-0.11037	-0.15670						
256	-0.05004	-0.04644	-0.06811	-0.07879	-0.09162	-0.12546	-0.18261							
128	-0.04444	-0.05248	-0.07729	-0.09943	-0.13337	-0.19923								
64	-0.00181	-0.05884	-0.11272	-0.16033	-0.22281									
32	0.10663	-0.06009	-0.16387	-0.25039										
16	0.24326	-0.05352	-0.24016											
8	0.38136	-0.08354												
4	0.45597													

(c) $\mathcal{P}^{\mathcal{C}}$ for Scene: [**Veach Door**] Algorithm: [**PSSMLT**] Metric: [**VSNR**] True GT: [**BDPT @ 524288 spp**].

MLT	2	4	8	16	32	64	128	256	512	1024	2048	4096	8192	16384
GT (BDPT)														
262144	0.01917	-0.00045	-0.00027	-0.00021	-0.00017	-0.00009	-0.00010	-0.00027	0.00010	0.00037	0.00039	-0.00521	-0.00366	-0.00335
131072	0.01834	-0.00098	-0.00068	-0.00047	-0.00034	-0.00034	-0.00050	-0.00015	0.00016	-0.00002	-0.00604	-0.00498	-0.00932	-0.01145
65536	0.01141	0.00442	-0.00317	-0.00257	0.00379	-0.00178	-0.00170	-0.00142	-0.00099	-0.00084	-0.00564	-0.01652	-0.02112	-0.02349
32768	0.01940	0.00698	-0.00766	0.00176	0.00063	-0.00002	-0.00410	-0.00014	-0.00621	-0.00839	-0.01811	-0.03305	-0.04529	-0.04270
16384	0.00084	-0.00317	-0.00394	-0.00393	0.00200	-0.00358	-0.00718	-0.00642	-0.01196	-0.01613	-0.03311	-0.06055	-0.07829	
8192	0.00286	-0.00511	-0.00717	0.00018	-0.00155	-0.00693	-0.01063	-0.01295	-0.02482	-0.04169	-0.06400	-0.10870		
4096	0.00942	-0.00854	-0.01310	-0.00617	-0.00787	-0.01347	-0.02097	-0.02869	-0.04662	-0.07472	-0.11754			
2048	-0.03593	-0.02034	-0.01566	-0.01919	-0.02087	-0.02594	-0.03703	-0.05018	-0.08522	-0.13322				
1024	-0.04824	-0.02516	-0.02824	-0.02596	-0.03611	-0.04884	-0.06329	-0.09468	-0.14576					
512	-0.04399	-0.04348	-0.04182	-0.04588	-0.05293	-0.07957	-0.11383	-0.16105						
256	-0.06855	-0.06049	-0.06702	-0.07850	-0.09431	-0.13520	-0.18829							
128	-0.04295	-0.05877	-0.08511	-0.11114	-0.14808	-0.21754								
64	-0.01077	-0.06771	-0.12746	-0.17490	-0.24729									
32	0.07272	-0.08954	-0.18610	-0.27865										
16	0.16676	-0.10346	-0.28581											
8	0.25121	-0.15548												
4	0.32336													

(d) $\mathcal{P}^{\mathcal{C}}$ for Scene: [**Veach Door**] Algorithm: [**MLT**] Metric: [**VSNR**] True GT: [**BDPT @ 524288 spp**].

M-MLT	2	4	8	16	32	64	128	256	512	1024	2048	4096	8192	16384
GT (BDPT)														
262144	-0.00065	0.00819	-0.00029	-0.00020	-0.00011	-0.00014	-0.00036	0.00010	-0.00020	0.00019	-0.00027	0.00067	-0.00827	-0.00786
131072	-0.00152	0.00781	-0.00050	-0.00062	-0.00050	-0.00029	-0.00071	0.00003	-0.00046	0.00013	-0.00614	-0.00418	-0.01298	-0.01340
65536	-0.00783	0.01268	0.00731	-0.00239	-0.00208	-0.00193	-0.00180	-0.00176	-0.00183	-0.00136	-0.01247	-0.00944	-0.02791	-0.03055
32768	-0.00075	0.00657	0.00285	-0.00591	0.00051	-0.00436	-0.00465	-0.00346	-0.00319	-0.00922	-0.02077	-0.02835	-0.05433	-0.06132
16384	0.00008	0.00575	0.00658	-0.00387	-0.00418	-0.00407	-0.00382	-0.00690	-0.01317	-0.01709	-0.04040	-0.04539	-0.09298	
8192	0.00118	0.01095	0.00330	-0.00766	-0.00785	-0.00821	-0.00700	-0.01432	-0.02314	-0.03668	-0.07393	-0.08684		
4096	-0.02974	-0.00882	-0.00252	-0.01326	-0.00840	-0.01400	-0.01972	-0.02932	-0.04632	-0.07129	-0.12841			
2048	-0.03612	-0.02103	-0.01618	-0.01860	-0.02081	-0.03270	-0.04038	-0.05456	-0.08547	-0.12685				
1024	-0.05110	-0.02660	-0.02902	-0.03451	-0.03767	-0.05035	-0.06929	-0.09877	-0.14976					
512	-0.05117	-0.03177	-0.03414	-0.05308	-0.06035	-0.08640	-0.11930	-0.16758						
256	-0.07612	-0.05096	-0.05898	-0.08320	-0.10294	-0.14799	-0.19519							
128	-0.05899	-0.06640	-0.07828	-0.11613	-0.15863	-0.22760								
64	-0.03040	-0.07391	-0.12116	-0.18391	-0.25879									
32	0.01071	-0.10545	-0.19784	-0.29068										
16	0.10090	-0.12245	-0.29802											
8	0.17451	-0.19150												
4	0.24541													

(e) $\mathcal{P}^{\mathcal{C}}$ for Scene: [**Veach Door**] Algorithm: [**M-MLT**] Metric: [**VSNR**] True GT: [**BDPT @ 524288 spp**].

ERPT	2	4	8	16	32	64	128	256	512	1024	2048	4096	8192	16384	32768	65536	131072
GT (BDPT)																	
262144	-0.00013	-0.00023	-0.00012	0.00002	-0.00026	0.00004	-0.00344	-0.00019	-0.00020	-0.00019	-0.00024	-0.00004	-0.00023	-0.00327	-0.00020	-0.00010	0.00006
131072	-0.00050	-0.00049	-0.00034	-0.00037	-0.00048	-0.00022	-0.00370	-0.00046	-0.00376	-0.00041	-0.00024	-0.00381	-0.00389	-0.00343	-0.00009	-0.00045	
65536	-0.00162	-0.00144	-0.00185	-0.00162	-0.00163	-0.00170	-0.00149	-0.00193	-0.00186	-0.00158	-0.00174	-0.00172	-0.00163	-0.00139	-0.00146		
32768	-0.00399	-0.00411	-0.00420	-0.00395	-0.00411	-0.00060	-0.00395	-0.00075	-0.00453	-0.00390	-0.00404	-0.00394	-0.00413	-0.00351			
16384	-0.00653	-0.00723	-0.00666	-0.00717	-0.00776	-0.00367	-0.00365	-0.00461	-0.00382	-0.00778	-0.00726	-0.00424	-0.00379				
8192	-0.00912	-0.01322	-0.01457	-0.01142	-0.01466	-0.01226	-0.01096	-0.01178	-0.01085	-0.01505	-0.01476	-0.01129					
4096	-0.01980	-0.02089	-0.02101	-0.02256	-0.02197	-0.02616	-0.02574	-0.02645	-0.02551	-0.02627	-0.02331						
2048	-0.03451	-0.04241	-0.04535	-0.04634	-0.04712	-0.05100	-0.04960	-0.05044	-0.04822	-0.04733							
1024	-0.06635	-0.07386	-0.08003	-0.08378	-0.08385	-0.08948	-0.08637	-0.08716	-0.08614								
512	-0.11450	-0.12520	-0.13966	-0.14058	-0.14304	-0.14818	-0.14567	-0.14926									
256	-0.18893	-0.19985	-0.21645	-0.22851	-0.22993	-0.23621	-0.23511										
128	-0.29225	-0.31297	-0.33418	-0.34845	-0.35063	-0.35633											
64	-0.43232	-0.45076	-0.47555	-0.49033	-0.49327												
32	-0.60924	-0.62750	-0.65739	-0.67358													
16	-0.80655	-0.83176	-0.85434														
8	-1.03733	-1.06284															
4	-1.24838																

(f) $\mathcal{P}^{\mathcal{C}}$ for Scene: [**Veach Door**] Algorithm: [**ERPT**] Metric: [**VSNR**] True GT: [**BDPT @ 524288 spp**].

M-ERPT	2	4	8	16	32	64	128	256	512	1024	2048	4096	8192	16384	32768	65536	131072
GT (BDPT)																	
262144	0.00011	-0.00032	-0.00020	-0.00008	-0.00014	-0.00017	-0.00026	-0.00005	-0.00026	-0.00012	0.00015	-0.00018	-0.00026	-0.00023	-0.00032	-0.00024	-0.00029
131072	-0.00026	-0.00054	-0.00038	-0.00028	-0.00024	-0.00388	-0.00061	-0.00050	-0.00033	-0.00040	-0.00022	-0.00048	-0.00065	-0.00038	-0.00040	-0.00057	
65536	-0.00168	0.00199	0.00173	-0.00175	-0.00184	-0.00202	-0.00201	-0.00193	-0.00156	-0.00160	-0.00150	-0.00197	-0.00211	-0.00162	-0.00188		
32768	-0.00396	-0.00060	-0.00039	-0.00080	-0.00442	-0.00441	-0.00462	-0.00448	-0.00456	-0.00416	-0.00398	-0.00423	-0.00472	-0.00457			
16384	0.00119	-0.00054	-0.00420	-0.00103	-0.00815	-0.00840	-0.00804	-0.00726	-0.00448	-0.00815	-0.00784	-0.00825	-0.00793				
8192	-0.00602	-0.00795	-0.01153	-0.00834	-0.01541	-0.01574	-0.01633	-0.01187	-0.00917	-0.01178	-0.01260	-0.01584					
4096	-0.01322	-0.01493	-0.02059	-0.01970	-0.02401	-0.02395	-0.02545	-0.02479	-0.02509	-0.02325	-0.02330						
2048	-0.03324	-0.04057	-0.04626	-0.04113	-0.04928	-0.05061	-0.05041	-0.05026	-0.04745	-0.04937							
1024	-0.06382	-0.07421	-0.08392	-0.08080	-0.08842	-0.08955	-0.08907	-0.08967	-0.08683								
512	-0.10162	-0.12666	-0.13427	-0.13696	-0.14047	-0.14474	-0.14788	-0.14199									
256	-0.18012	-0.20751	-0.21906	-0.22257	-0.22926	-0.23016	-0.23284										
128	-0.28274	-0.31435	-0.33566	-0.33398	-0.34273	-0.34115											
64	-0.41565	-0.45785	-0.47662	-0.48504	-0.49422												
32	-0.59765	-0.63681	-0.65600	-0.66016													
16	-0.78757	-0.82603	-0.85617														
8	-1.01900	-1.06642															
4	-1.23096																

(g) $\mathcal{P}^{\mathcal{C}}$ for Scene: [**Veach Door**] Algorithm: [**M-ERPT**] Metric: [**VSNR**] True GT: [**BDPT @ 524288 spp**].

Table 39

9.5 Sponza

PT	2	4	8	16	32	64	128	256	512	1024	2048	4096	8192
GT (PT)													
16384	-0.01020	-0.00046	-0.00584	0.00174	0.00076	-0.00003	-0.00022	-0.00403	-0.00339	-0.00491	-0.00972	-0.01466	-0.02076
8192	-0.00409	-0.00536	-0.00571	0.00079	-0.00110	-0.00215	-0.00300	-0.00706	-0.01017	-0.01672	-0.02699	-0.03905	
4096	-0.01327	-0.00024	-0.00994	-0.00088	-0.00423	-0.00618	-0.00769	-0.01316	-0.02048	-0.03070	-0.05280		
2048	-0.01604	-0.01435	-0.01431	-0.00811	-0.00814	-0.01247	-0.01839	-0.02574	-0.04041	-0.06247			
1024	-0.02909	-0.01838	-0.02029	-0.01493	-0.01835	-0.02207	-0.03525	-0.05066	-0.07388				
512	-0.03679	-0.02857	-0.03648	-0.02967	-0.03164	-0.03919	-0.06051	-0.08590					
256	-0.05110	-0.03945	-0.04040	-0.04259	-0.05214	-0.06907	-0.09862						
128	-0.05689	-0.04737	-0.05859	-0.06298	-0.08160	-0.11259							
64	-0.07788	-0.06721	-0.07989	-0.09305	-0.13063								
32	-0.06514	-0.07277	-0.10037	-0.14029									
16	-0.07991	-0.10049	-0.15633										
8	-0.06749	-0.14651											
4	-0.09651												

(a) $\mathcal{P}^{\mathcal{C}}$ for Scene: [**Sponza**] Algorithm: [**PT**] Metric: [**VSNR**] True GT: [**PT @ 32768 spp**].

BDPT	2	4	8	16	32	64	128	256	512	1024	2048	4096	8192
GT (PT)													
16384	0.00187	0.00020	-0.00620	0.00216	-0.00418	0.00041	-0.00326	-0.00057	-0.00122	-0.00528	-0.00834	-0.01082	-0.01920
8192	-0.00330	-0.00480	-0.00553	0.00121	-0.00587	-0.00188	-0.00256	-0.00386	-0.00765	-0.01139	-0.02114	-0.03367	
4096	-0.01226	-0.00565	-0.01430	-0.00689	-0.00870	-0.00518	-0.00714	-0.01160	-0.01642	-0.02592	-0.04809		
2048	-0.01235	-0.01178	-0.01810	-0.00637	-0.01154	-0.01071	-0.01772	-0.02163	-0.03612	-0.05601			
1024	-0.02236	-0.02716	-0.02605	-0.01886	-0.02104	-0.02262	-0.02875	-0.03928	-0.06383				
512	-0.03550	-0.03338	-0.03150	-0.03166	-0.03199	-0.03457	-0.05371	-0.07374					
256	-0.04477	-0.03991	-0.04883	-0.04079	-0.05017	-0.05765	-0.08927						
128	-0.06152	-0.05414	-0.05682	-0.05622	-0.07572	-0.09998							
64	-0.06066	-0.06894	-0.07953	-0.08456	-0.11641								
32	-0.05495	-0.07202	-0.09679	-0.12058									
16	-0.05029	-0.08866	-0.14030										
8	-0.02809	-0.11914											
4	-0.03604												

(b) $\mathcal{P}^{\mathcal{C}}$ for Scene: [**Sponza**] Algorithm: [**BDPT**] Metric: [**VSNR**] True GT: [**PT @ 32768 spp**].

PSSMLT	2	4	8	16	32	64	128	256	512	1024	2048	4096	8192
GT (PT)													
16384	-0.01007	-0.00079	-0.00658	0.00235	-0.00473	0.00052	-0.00010	-0.00045	-0.00072	-0.00097	-0.00123	-0.00399	-0.00381
8192	-0.00501	-0.00601	-0.00634	0.00132	-0.00619	-0.00144	-0.00261	-0.00329	-0.00385	-0.00440	-0.00525	-0.01041	
4096	-0.01498	-0.00152	-0.00559	-0.00009	-0.00909	-0.00511	-0.00709	-0.00842	-0.01263	-0.01061	-0.01744		
2048	-0.01021	-0.00998	-0.01081	-0.00741	-0.01210	-0.01000	-0.01355	-0.01631	-0.02184	-0.02704			
1024	-0.02645	-0.02238	-0.02184	-0.01421	-0.02150	-0.02232	-0.02492	-0.03646	-0.04734				
512	-0.03058	-0.02913	-0.03098	-0.02117	-0.03340	-0.03284	-0.04646	-0.06457					
256	-0.05157	-0.03910	-0.04468	-0.03490	-0.04842	-0.05707	-0.07826						
128	-0.05151	-0.05113	-0.05712	-0.05559	-0.07437	-0.09723							
64	-0.06655	-0.06545	-0.08089	-0.09243	-0.12657								
32	-0.06135	-0.07381	-0.09940	-0.13254									
16	-0.08400	-0.11697	-0.16556										
8	-0.09752	-0.15348											
4	-0.14325												

(c) $\mathcal{P}^{\mathcal{C}}$ for Scene: [**Sponza**] Algorithm: [**PSSMLT**] Metric: [**VSNR**] True GT: [**PT @ 32768 spp**].

MLT	2	4	8	16	32	64	128	256	512	1024	2048	4096	8192
GT (PT)													
16384	-0.00073	-0.00135	0.00153	0.00066	0.00019	-0.00001	0.00259	-0.00332	-0.00329	-0.00345	-0.00123	-0.00141	-0.00350
8192	0.00087	-0.00152	0.00007	-0.00107	-0.00184	-0.00224	-0.00312	-0.00351	-0.00970	-0.00987	-0.00550	-0.00556	
4096	-0.00814	-0.00205	-0.00224	-0.00431	-0.00603	-0.00676	-0.00781	-0.01249	-0.01593	-0.01973	-0.01793		
2048	-0.01040	-0.00765	-0.00949	-0.01251	-0.01162	-0.01453	-0.01670	-0.02537	-0.03208	-0.03712			
1024	-0.01632	-0.01903	-0.01715	-0.01871	-0.02371	-0.02885	-0.03898	-0.04906	-0.06118				
512	-0.02852	-0.02913	-0.02594	-0.03127	-0.04070	-0.05074	-0.06562	-0.08698					
256	-0.04309	-0.03700	-0.04083	-0.05293	-0.06549	-0.09130	-0.11471						
128	-0.05339	-0.04948	-0.06245	-0.07857	-0.11058	-0.14873							
64	-0.06475	-0.07745	-0.09159	-0.12919	-0.17485								
32	-0.07069	-0.09801	-0.14495	-0.20017									
16	-0.10248	-0.15671	-0.22590										
8	-0.13093	-0.22566											
4	-0.18710												

(d) $\mathcal{P}^{\mathcal{C}}$ for Scene: [**Sponza**] Algorithm: [**MLT**] Metric: [**VSNR**] True GT: [**PT @ 32768 spp**].

M-MLT	2	4	8	16	32	64	128	256	512	1024	2048	4096	8192
GT (PT)													
16384	-0.00693	-0.00553	0.00141	0.00083	-0.00382	-0.00013	-0.00350	-0.00086	-0.00297	-0.00349	-0.00142	-0.00386	-0.00397
8192	-0.00544	-0.00573	0.00032	-0.00104	-0.00213	-0.00263	-0.00634	-0.00389	-0.00992	-0.00977	-0.00482	-0.00795	
4096	-0.01478	-0.00593	-0.00195	-0.00421	-0.00642	-0.00730	-0.01135	-0.01114	-0.01591	-0.01772	-0.01777		
2048	-0.01677	-0.01166	-0.00990	-0.00872	-0.01202	-0.01745	-0.02222	-0.02536	-0.03229	-0.03690			
1024	-0.02378	-0.01526	-0.01774	-0.01910	-0.02443	-0.03185	-0.03891	-0.05049	-0.06166				
512	-0.03740	-0.02572	-0.02631	-0.03255	-0.04084	-0.05157	-0.07258	-0.08916					
256	-0.04326	-0.04022	-0.04141	-0.04935	-0.06990	-0.09113	-0.11918						
128	-0.05538	-0.05415	-0.05774	-0.08078	-0.11212	-0.14780							
64	-0.07086	-0.07748	-0.09455	-0.13338	-0.18505								
32	-0.08829	-0.10524	-0.14515	-0.20023									
16	-0.12704	-0.16081	-0.23359										
8	-0.17373	-0.23791											
4	-0.24834												

(e) $\mathcal{P}^{\mathcal{C}}$ for Scene: [**Sponza**] Algorithm: [**M-MLT**] Metric: [**VSNR**] True GT: [**PT @ 32768 spp**].

ERPT	2	4	8	16	32	64	128	256	512	1024	2048	4096	8192
GT (PT)													
16384	-0.00359	-0.00346	-0.00035	-0.00057	-0.00077	-0.00079	-0.00045	-0.00058	-0.00075	0.00221	-0.00082	-0.00046	-0.00330
8192	-0.00642	-0.00650	-0.00357	-0.00624	-0.00674	-0.00627	-0.00356	-0.00608	-0.00620	-0.00315	-0.00611	-0.00662	
4096	-0.01411	-0.01104	-0.00838	-0.01191	-0.01148	-0.01177	-0.01111	-0.01188	-0.01176	-0.00852	-0.01148		
2048	-0.02219	-0.02245	-0.02301	-0.02045	-0.02059	-0.02065	-0.01999	-0.02071	-0.02353	-0.02041			
1024	-0.04147	-0.04289	-0.04073	-0.04398	-0.04436	-0.04435	-0.04439	-0.04328	-0.04318				
512	-0.07170	-0.07505	-0.07415	-0.07651	-0.07704	-0.07858	-0.07714	-0.07703					
256	-0.12344	-0.12647	-0.12655	-0.13108	-0.13189	-0.13156	-0.12830						
128	-0.19821	-0.20675	-0.20541	-0.20732	-0.20710	-0.20775							
64	-0.29923	-0.30689	-0.30897	-0.31329	-0.31275								
32	-0.42589	-0.43504	-0.43211	-0.44249									
16	-0.58368	-0.59024	-0.59116										
8	-0.75003	-0.75512											
4	-0.90512												

(f) $\mathcal{P}^{\mathcal{C}}$ for Scene: [**Sponza**] Algorithm: [**ERPT**] Metric: [**VSNR**] True GT: [**PT @ 32768 spp**].

M-ERPT	2	4	8	16	32	64	128	256	512	1024	2048	4096	8192
GT (PT)													
16384	-0.00349	-0.00346	0.00245	-0.00054	-0.00358	-0.00330	0.00232	-0.00028	-0.00057	-0.00044	-0.00098	-0.00059	-0.00046
8192	-0.00355	-0.00632	-0.00354	-0.00604	-0.00685	-0.00656	-0.00355	-0.00371	-0.00320	-0.00648	-0.00643	-0.00624	
4096	-0.00855	-0.01176	-0.00934	-0.01199	-0.01199	-0.01438	-0.00906	-0.01110	-0.01139	-0.01163	-0.01189		
2048	-0.02266	-0.02314	-0.02076	-0.02003	-0.02366	-0.02351	-0.01711	-0.02101	-0.02102	-0.02065			
1024	-0.04069	-0.03972	-0.04114	-0.04285	-0.04394	-0.04373	-0.04154	-0.04529	-0.04427				
512	-0.07142	-0.07445	-0.07519	-0.07702	-0.07762	-0.08049	-0.07504	-0.07760					
256	-0.11758	-0.12821	-0.12630	-0.12435	-0.12985	-0.12997	-0.12305						
128	-0.19179	-0.20258	-0.20400	-0.20375	-0.21038	-0.21107							
64	-0.29773	-0.30441	-0.30391	-0.30849	-0.30868								
32	-0.41902	-0.43130	-0.43327	-0.43510									
16	-0.57703	-0.58630	-0.59281										
8	-0.74342	-0.75725											
4	-0.89848												

(g) $\mathcal{P}^{\mathcal{C}}$ for Scene: [**Sponza**] Algorithm: [**M-ERPT**] Metric: [**VSNR**] True GT: [**PT @ 32768 spp**].

Table 40

10 FSIM

10.1 Cornell Box

PT	2	4	8	16	32	64	128	256	512	1024	2048	4096	8192
**GT (PT)**													
16384	0.00118	0.00115	0.00099	0.00080	0.00069	0.00049	0.00040	0.00026	0.00012	0.00006	0.00003	-0.00001	-0.00005
8192	0.00380	0.00347	0.00307	0.00247	0.00207	0.00156	0.00109	0.00071	0.00040	0.00018	0.00005	-0.00006	
4096	0.00830	0.00742	0.00661	0.00537	0.00435	0.00331	0.00224	0.00144	0.00079	0.00033	-0.00002		
2048	0.01575	0.01428	0.01243	0.01019	0.00793	0.00597	0.00413	0.00244	0.00125	0.00028			
1024	0.02705	0.02412	0.02084	0.01674	0.01285	0.00923	0.00614	0.00328	0.00130				
512	0.04361	0.03849	0.03315	0.02607	0.01958	0.01345	0.00838	0.00390					
256	0.06487	0.05671	0.04830	0.03713	0.02692	0.01743	0.00915						
128	0.09381	0.08081	0.06647	0.04904	0.03304	0.01936							
64	0.12743	0.10796	0.08621	0.06067	0.03718								
32	0.16646	0.13585	0.10372	0.06609									
16	0.20486	0.16116	0.11414										
8	0.23829	0.17613											
4	0.25917												

(a) $\mathcal{P}^{\mathcal{C}}$ for Scene: [**Cornell Box**] Algorithm: [**PT**] Metric: [**FSIM**] True GT: [**PT @ 32768 spp**].

BDPT	2	4	8	16	32	64	128	256	512	1024	2048	4096	8192
**GT (PT)**													
16384	0.00109	0.00096	0.00076	0.00057	0.00046	0.00029	0.00020	0.00012	0.00004	-0.00000	-0.00002	-0.00005	-0.00006
8192	0.00344	0.00298	0.00238	0.00181	0.00133	0.00090	0.00059	0.00031	0.00011	-0.00001	-0.00011	-0.00016	
4096	0.00747	0.00643	0.00511	0.00399	0.00286	0.00193	0.00118	0.00059	0.00018	-0.00014	-0.00031		
2048	0.01407	0.01197	0.00945	0.00720	0.00509	0.00322	0.00193	0.00083	-0.00000	-0.00052			
1024	0.02357	0.01988	0.01531	0.01149	0.00781	0.00489	0.00248	0.00054	-0.00065				
512	0.03776	0.03142	0.02390	0.01728	0.01119	0.00626	0.00239	-0.00064					
256	0.05534	0.04474	0.03288	0.02262	0.01350	0.00600	0.00021						
128	0.07673	0.06026	0.04210	0.02669	0.01276	0.00189							
64	0.10124	0.07657	0.04901	0.02664	0.00741								
32	0.12569	0.08775	0.04981	0.01838									
16	0.14434	0.09125	0.03847										
8	0.15188	0.07809											
4	0.13867												

(b) $\mathcal{P}^{\mathcal{C}}$ for Scene: [**Cornell Box**] Algorithm: [**BDPT**] Metric: [**FSIM**] True GT: [**PT @ 32768 spp**].

PSSMLT	2	4	8	16	32	64	128	256	512	1024	2048	4096	8192
**GT (PT)**													
16384	0.00101	0.00094	0.00083	0.00066	0.00049	0.00033	0.00023	0.00014	0.00008	0.00003	0.00000	-0.00001	-0.00002
8192	0.00318	0.00273	0.00234	0.00184	0.00135	0.00092	0.00061	0.00038	0.00020	0.00006	-0.00002	-0.00007	
4096	0.00694	0.00604	0.00503	0.00399	0.00294	0.00201	0.00124	0.00077	0.00034	0.00006	-0.00011		
2048	0.01311	0.01126	0.00918	0.00723	0.00526	0.00346	0.00206	0.00111	0.00033	-0.00017			
1024	0.02195	0.01884	0.01516	0.01151	0.00812	0.00500	0.00262	0.00111	-0.00018				
512	0.03518	0.02957	0.02344	0.01732	0.01162	0.00656	0.00275	0.00024					
256	0.05179	0.04211	0.03242	0.02279	0.01395	0.00650	0.00076						
128	0.07184	0.05683	0.04178	0.02708	0.01391	0.00311							
64	0.09394	0.07162	0.04893	0.02757	0.00847								
32	0.11761	0.08315	0.05008	0.02039									
16	0.13329	0.08535	0.03935										
8	0.13839	0.07163											
4	0.12045												

(c) $\mathcal{P}^{\mathcal{C}}$ for Scene: [**Cornell Box**] Algorithm: [**PSSMLT**] Metric: [**FSIM**] True GT: [**PT @ 32768 spp**].

MLT	2	4	8	16	32	64	128	256	512	1024	2048	4096	8192
**GT (PT)**													
16384	0.00122	0.00115	0.00106	0.00092	0.00080	0.00064	0.00052	0.00036	0.00022	0.00013	0.00007	0.00003	0.00000
8192	0.00381	0.00354	0.00330	0.00291	0.00253	0.00206	0.00150	0.00103	0.00069	0.00040	0.00020	0.00007	
4096	0.00837	0.00793	0.00730	0.00639	0.00540	0.00433	0.00319	0.00220	0.00141	0.00084	0.00037		
2048	0.01614	0.01494	0.01371	0.01198	0.00998	0.00790	0.00575	0.00389	0.00246	0.00137			
1024	0.02779	0.02571	0.02326	0.01989	0.01632	0.01266	0.00899	0.00582	0.00335				
512	0.04526	0.04153	0.03726	0.03141	0.02516	0.01919	0.01295	0.00777					
256	0.06859	0.06262	0.05521	0.04572	0.03570	0.02610	0.01652						
128	0.10072	0.09068	0.07873	0.06377	0.04771	0.03258							
64	0.13850	0.12328	0.10480	0.08162	0.05803								
32	0.18727	0.16247	0.13407	0.09933									
16	0.23911	0.20047	0.15852										
8	0.29035	0.23543											
4	0.33738												

(d) $\mathcal{P}^{\mathcal{C}}$ for Scene: [**Cornell Box**] Algorithm: [**MLT**] Metric: [**FSIM**] True GT: [**PT @ 32768 spp**].

M-MLT	2	4	8	16	32	64	128	256	512	1024	2048	4096	8192
**GT (PT)**													
16384	0.00121	0.00116	0.00102	0.00090	0.00077	0.00061	0.00050	0.00032	0.00023	0.00013	0.00006	0.00002	0.00001
8192	0.00375	0.00359	0.00329	0.00283	0.00244	0.00200	0.00149	0.00103	0.00064	0.00038	0.00018	0.00009	
4096	0.00840	0.00775	0.00709	0.00614	0.00510	0.00409	0.00303	0.00210	0.00135	0.00076	0.00034		
2048	0.01585	0.01477	0.01329	0.01141	0.00959	0.00762	0.00556	0.00375	0.00227	0.00113			
1024	0.02750	0.02544	0.02259	0.01909	0.01559	0.01205	0.00846	0.00542	0.00294				
512	0.04471	0.04099	0.03617	0.03024	0.02464	0.01849	0.01234	0.00746					
256	0.06745	0.06158	0.05356	0.04411	0.03458	0.02468	0.01520						
128	0.09823	0.08911	0.07530	0.06020	0.04534	0.02991							
64	0.13602	0.12145	0.10024	0.07702	0.05499								
32	0.18085	0.15984	0.12708	0.09268									
16	0.22942	0.19652	0.14963										
8	0.27564	0.22866											
4	0.31796												

(e) $\mathcal{P}^{\mathcal{C}}$ for Scene: [**Cornell Box**] Algorithm: [**M-MLT**] Metric: [**FSIM**] True GT: [**PT @ 32768 spp**].

ERPT	2	4	8	16	32	64	128	256	512	1024	2048	4096	8192
**GT (PT)**													
16384	0.00043	0.00042	0.00045	0.00037	0.00048	0.00049	0.00044	0.00044	0.00047	0.00046	0.00045	0.00042	0.00044
8192	0.00133	0.00131	0.00136	0.00132	0.00141	0.00139	0.00139	0.00137	0.00142	0.00134	0.00135	0.00140	
4096	0.00289	0.00275	0.00298	0.00291	0.00291	0.00290	0.00293	0.00298	0.00306	0.00284	0.00291		
2048	0.00519	0.00501	0.00542	0.00530	0.00523	0.00524	0.00535	0.00535	0.00560	0.00513			
1024	0.00813	0.00795	0.00825	0.00820	0.00805	0.00801	0.00821	0.00811	0.00837				
512	0.01172	0.01122	0.01197	0.01180	0.01141	0.01155	0.01209	0.01171					
256	0.01425	0.01377	0.01462	0.01451	0.01398	0.01419	0.01484						
128	0.01479	0.01391	0.01486	0.01500	0.01438	0.01432							
64	0.01006	0.00894	0.01052	0.01031	0.01022								
32	-0.00395	-0.00540	-0.00373	-0.00366									
16	-0.03324	-0.03494	-0.03282										
8	-0.08313	-0.08616											
4	-0.15899												

(f) $\mathcal{P}^{\mathcal{C}}$ for Scene: [**Cornell Box**] Algorithm: [**ERPT**] Metric: [**FSIM**] True GT: [**PT @ 32768 spp**].

M-ERPT	2	4	8	16	32	64	128	256	512	1024	2048	4096	8192
**GT (PT)**													
16384	0.00044	0.00039	0.00046	0.00044	0.00042	0.00041	0.00046	0.00046	0.00040	0.00042	0.00052	0.00044	0.00045
8192	0.00140	0.00129	0.00139	0.00140	0.00132	0.00141	0.00136	0.00142	0.00138	0.00143	0.00139	0.00142	
4096	0.00292	0.00288	0.00298	0.00305	0.00285	0.00301	0.00295	0.00299	0.00298	0.00302	0.00302		
2048	0.00539	0.00519	0.00543	0.00542	0.00514	0.00549	0.00545	0.00535	0.00541	0.00551			
1024	0.00835	0.00803	0.00840	0.00845	0.00799	0.00845	0.00836	0.00824	0.00827				
512	0.01183	0.01170	0.01217	0.01236	0.01163	0.01227	0.01186	0.01187					
256	0.01473	0.01430	0.01498	0.01552	0.01410	0.01500	0.01462						
128	0.01518	0.01456	0.01559	0.01588	0.01434	0.01575							
64	0.01052	0.01004	0.01114	0.01191	0.01003								
32	-0.00336	-0.00367	-0.00255	-0.00204									
16	-0.03178	-0.03291	-0.03045										
8	-0.08233	-0.08330											
4	-0.15771												

(g) $\mathcal{P}^{\mathcal{C}}$ for Scene: [**Cornell Box**] Algorithm: [**M-ERPT**] Metric: [**FSIM**] True GT: [**PT @ 32768 spp**].

Table 41

10.2 Torus

PT	2	4	8	16	32	64	128	256	512	1024	2048	4096	8192	16384	32768	65536	131072
GT (BDPT)																	
262144	0.00668	0.00554	0.00392	0.00299	0.00218	0.00164	0.00133	0.00173	0.00194	0.00220	0.00253	0.00265	0.00255	0.00219	0.00154	0.00063	-0.00012
131072	0.02030	0.01537	0.01147	0.00793	0.00625	0.00434	0.00367	0.00344	0.00538	0.00690	0.00840	0.00814	0.00726	0.00503	0.00307	0.00083	
65536	0.03663	0.02670	0.01858	0.01297	0.00885	0.00666	0.00510	0.00566	0.00888	0.01246	0.01339	0.01328	0.01029	0.00714	0.00285		
32768	0.05892	0.04214	0.02845	0.01793	0.01221	0.00902	0.00731	0.00852	0.01387	0.01938	0.02063	0.01927	0.01415	0.00677			
16384	0.08076	0.05569	0.03501	0.01945	0.01244	0.00762	0.00637	0.01037	0.01876	0.02481	0.02739	0.02261	0.01350				
8192	0.10205	0.06646	0.03725	0.01673	0.00702	0.00326	0.00618	0.01273	0.02389	0.03201	0.03109	0.02060					
4096	0.11469	0.06971	0.03295	0.01034	0.00144	-0.00358	0.00517	0.01617	0.02973	0.03626	0.02893						
2048	0.11489	0.06230	0.02011	-0.00435	-0.01136	-0.01032	-0.00090	0.01505	0.03236	0.03289							
1024	0.11100	0.05262	0.00455	-0.01786	-0.02358	-0.02020	-0.00537	0.00949	0.02256								
512	0.09705	0.04034	-0.00449	-0.02373	-0.02677	-0.02165	-0.00812	0.00570									
256	0.08610	0.03638	-0.00625	-0.01709	-0.02134	-0.01973	-0.00998										
128	0.09351	0.04654	0.01199	-0.00001	-0.01044	-0.01261											
64	0.10736	0.06201	0.02789	0.01124	-0.00359												
32	0.13379	0.08792	0.04996	0.02543													
16	0.17845	0.11732	0.07699														
8	0.21319	0.13732															
4	0.24722																

(a) $\mathcal{P}^{\mathcal{C}}$ for Scene: [**Torus**] Algorithm: [**PT**] Metric: [**FSIM**] True GT: [**BDPT @ 524288 spp**].

BDPT	2	4	8	16	32	64	128	256	512	1024	2048	4096	8192	16384	32768	65536	131072
GT (BDPT)																	
262144	0.00694	0.00497	0.00388	0.00285	0.00224	0.00182	0.00142	0.00121	0.00202	0.00227	0.00262	0.00303	0.00256	0.00200	0.00141	0.00054	-0.00009
131072	0.02049	0.01510	0.01147	0.00824	0.00598	0.00421	0.00341	0.00350	0.00553	0.00660	0.00762	0.00819	0.00732	0.00552	0.00345	0.00096	
65536	0.03736	0.02741	0.01941	0.01330	0.00883	0.00622	0.00546	0.00597	0.00870	0.01201	0.01365	0.01382	0.01141	0.00749	0.00347		
32768	0.05977	0.04271	0.02953	0.01893	0.01219	0.00859	0.00768	0.00807	0.01436	0.01812	0.02000	0.01941	0.01432	0.00752			
16384	0.08321	0.05824	0.03633	0.02166	0.01182	0.00708	0.00647	0.01087	0.01880	0.02354	0.02530	0.02254	0.01472				
8192	0.10693	0.07090	0.03970	0.02075	0.00809	0.00418	0.00432	0.01324	0.02526	0.03169	0.02971	0.02192					
4096	0.12292	0.07686	0.03907	0.01593	0.00101	-0.00045	0.00277	0.01453	0.02947	0.03308	0.02759						
2048	0.12560	0.06982	0.02518	0.00212	-0.01067	-0.00954	0.00185	0.01728	0.03077	0.03020							
1024	0.12208	0.06125	0.01383	-0.01124	-0.02303	-0.01793	-0.00693	0.01118	0.02303								
512	0.10929	0.04773	0.00269	-0.01961	-0.02753	-0.01826	-0.00701	0.00818									
256	0.09274	0.03875	0.00103	-0.01388	-0.02056	-0.01964	-0.00673										
128	0.09851	0.04992	0.01485	0.00158	-0.00561	-0.00927											
64	0.11237	0.06716	0.03103	0.01255	0.00004												
32	0.13895	0.08967	0.04942	0.02750													
16	0.18653	0.12571	0.07086														
8	0.22624	0.14942															
4	0.26008																

(b) $\mathcal{P}^{\mathcal{C}}$ for Scene: [**Torus**] Algorithm: [**BDPT**] Metric: [**FSIM**] True GT: [**BDPT @ 524288 spp**].

PSSMLT	2	4	8	16	32	64	128	256	512	1024	2048	4096	8192	16384
GT (BDPT)														
262144	0.00131	0.00186	0.00167	0.00174	0.00183	0.00221	0.00226	0.00252	0.00238	0.00259	0.00266	0.00265	0.00238	0.00202
131072	0.00366	0.00478	0.00516	0.00500	0.00536	0.00614	0.00663	0.00726	0.00747	0.00754	0.00742	0.00691	0.00615	0.00583
65536	0.00615	0.00819	0.00804	0.00764	0.00885	0.00980	0.01092	0.01218	0.01237	0.01223	0.01183	0.01076	0.00909	0.00781
32768	0.01095	0.01345	0.01233	0.01161	0.01366	0.01626	0.01769	0.01833	0.01748	0.01673	0.01485	0.01187	0.00883	0.00678
16384	0.01882	0.01856	0.01592	0.01397	0.01742	0.01910	0.02114	0.02046	0.01902	0.01698	0.01288	0.00774	0.00251	
8192	0.02964	0.02639	0.02030	0.01596	0.01980	0.02244	0.02279	0.02138	0.01514	0.01065	0.00273	-0.00625		
4096	0.03654	0.02703	0.02015	0.01572	0.02085	0.02262	0.02191	0.01648	0.00676	-0.00080	-0.01320			
2048	0.03408	0.01798	0.00818	0.00595	0.01444	0.01692	0.01523	0.00416	-0.01089	-0.02538				
1024	0.02810	0.00517	-0.00540	-0.00730	0.00091	0.00497	-0.00247	-0.01712	-0.03698					
512	0.00921	-0.00712	-0.01458	-0.01358	-0.00458	-0.00790	-0.01779	-0.03488						
256	-0.00112	-0.02008	-0.02630	-0.02495	-0.01904	-0.02306	-0.03363							
128	-0.01136	-0.02053	-0.02364	-0.02163	-0.01860	-0.02446								
64	-0.01564	-0.01820	-0.02061	-0.02171	-0.02321									
32	-0.00771	-0.00883	-0.01664	-0.01799										
16	0.00895	0.00213	-0.01382											
8	0.02198	0.00696												
4	0.02341													

(c) $\mathcal{P}^{\mathcal{C}}$ for Scene: [**Torus**] Algorithm: [**PSSMLT**] Metric: [**FSIM**] True GT: [**BDPT @ 524288 spp**].

MLT	2	4	8	16	32	64	128	256	512	1024	2048	4096	8192	16384
GT (BDPT)														
262144	-0.00142	-0.00041	0.00101	0.00151	0.00197	0.00196	0.00146	0.00192	0.00169	0.00160	0.00188	0.00154	0.00170	0.00183
131072	-0.00446	-0.00160	0.00273	0.00321	0.00508	0.00518	0.00440	0.00495	0.00403	0.00444	0.00425	0.00419	0.00403	0.00400
65536	-0.00771	-0.00250	0.00518	0.00677	0.00825	0.00769	0.00679	0.00700	0.00526	0.00561	0.00522	0.00483	0.00477	0.00413
32768	-0.00708	-0.00106	0.00895	0.01071	0.01169	0.00989	0.00770	0.00662	0.00396	0.00443	0.00307	0.00265	0.00237	0.00118
16384	-0.00540	0.00301	0.01411	0.01469	0.01385	0.00966	0.00555	0.00369	-0.00084	-0.00093	-0.00342	-0.00465	-0.00542	
8192	0.00139	0.01262	0.02190	0.01772	0.01339	0.00640	-0.00201	-0.00719	-0.01484	-0.01636	-0.01872	-0.02161		
4096	0.00348	0.01481	0.02338	0.01320	0.00461	-0.00437	-0.01702	-0.02535	-0.03486	-0.03671	-0.04081			
2048	0.00441	0.01303	0.01523	-0.00175	-0.01454	-0.02647	-0.04065	-0.04942	-0.06176	-0.06446				
1024	-0.00171	0.00725	0.00003	-0.02472	-0.03956	-0.05337	-0.07034	-0.08041	-0.09336					
512	-0.01404	-0.00651	-0.01786	-0.04671	-0.05949	-0.07388	-0.09167	-0.10268						
256	-0.02455	-0.02191	-0.03236	-0.06105	-0.07372	-0.08407	-0.10197							
128	-0.03039	-0.03106	-0.04072	-0.06591	-0.07212	-0.08033								
64	-0.03022	-0.03652	-0.04506	-0.07122	-0.06876									
32	-0.02848	-0.03337	-0.03508	-0.05903										
16	-0.01824	-0.02337	-0.01915											
8	-0.02269	-0.02540												
4	-0.03609													

(d) $\mathcal{P}^{\mathcal{C}}$ for Scene: [**Torus**] Algorithm: [**MLT**] Metric: [**FSIM**] True GT: [**BDPT @ 524288 spp**].

M-MLT	2	4	8	16	32	64	128	256	512	1024	2048	4096	8192	16384
GT (BDPT)														
262144	-0.00102	0.00033	0.00147	0.00147	0.00206	0.00186	0.00151	0.00165	0.00171	0.00145	0.00162	0.00152	0.00162	0.00152
131072	-0.00426	0.00112	0.00477	0.00412	0.00621	0.00503	0.00491	0.00542	0.00428	0.00423	0.00434	0.00426	0.00410	0.00390
65536	-0.00669	0.00159	0.00893	0.00643	0.00978	0.00719	0.00776	0.00757	0.00549	0.00572	0.00573	0.00524	0.00525	0.00453
32768	-0.00767	0.00469	0.01525	0.01040	0.01453	0.00878	0.00966	0.00781	0.00352	0.00402	0.00319	0.00280	0.00228	0.00121
16384	-0.00609	0.01032	0.02227	0.01458	0.01787	0.00705	0.00767	0.00422	-0.00256	-0.00419	-0.00517	-0.00560	-0.00714	
8192	-0.00089	0.01929	0.02954	0.01488	0.01785	-0.00077	-0.00140	-0.00922	-0.01969	-0.01933	-0.02305	-0.02405		
4096	-0.00193	0.02332	0.02962	0.00983	0.01167	-0.01759	-0.01780	-0.02842	-0.04076	-0.04300	-0.04648			
2048	0.00015	0.02155	0.01722	-0.00705	-0.00832	-0.04354	-0.04518	-0.05686	-0.07280	-0.07464				
1024	-0.00089	0.01186	-0.00347	-0.03182	-0.03182	-0.07939	-0.08068	-0.09265	-0.10907					
512	-0.01275	-0.00773	-0.02946	-0.05809	-0.05563	-0.10282	-0.10391	-0.11721						
256	-0.01377	-0.01937	-0.04613	-0.07532	-0.06780	-0.11514	-0.11598							
128	-0.01650	-0.03160	-0.06134	-0.07917	-0.06919	-0.11713								
64	-0.01821	-0.04008	-0.06530	-0.08061	-0.06964									
32	-0.02110	-0.03548	-0.05750	-0.07389										
16	-0.01988	-0.01686	-0.04226											
8	-0.03857	-0.00602												
4	-0.06735													

(e) $\mathcal{P}^{\mathcal{C}}$ for Scene: [**Torus**] Algorithm: [**M-MLT**] Metric: [**FSIM**] True GT: [**BDPT @ 524288 spp**].

ERPT	2	4	8	16	32	64	128	256	512	1024	2048	4096	8192	16384	32768	65536	131072
GT (BDPT)																	
262144	0.00207	0.00229	0.00233	0.00234	0.00176	0.00227	0.00221	0.00222	0.00265	0.00251	0.00264	0.00271	0.00265	0.00254	0.00253	0.00247	0.00251
131072	0.00467	0.00650	0.00562	0.00599	0.00595	0.00659	0.00653	0.00696	0.00693	0.00722	0.00729	0.00694	0.00720	0.00669	0.00697	0.00692	
65536	0.00584	0.00930	0.00843	0.00893	0.00897	0.00972	0.00993	0.01111	0.01088	0.01092	0.01085	0.01033	0.01062	0.01031	0.01038		
32768	0.00575	0.01128	0.01002	0.00999	0.01060	0.01222	0.01183	0.01367	0.01291	0.01286	0.01231	0.01160	0.01177	0.01225			
16384	0.00187	0.00937	0.00700	0.00695	0.00792	0.01009	0.00909	0.01153	0.01027	0.01001	0.00933	0.00741	0.00754				
8192	-0.01071	-0.00146	-0.00394	-0.00397	-0.00226	-0.00081	-0.00205	0.00149	-0.00115	-0.00215	-0.00301	-0.00524					
4096	-0.02698	-0.01681	-0.02008	-0.02143	-0.01903	-0.01754	-0.02008	-0.01615	-0.02035	-0.02059	-0.02532						
2048	-0.05537	-0.04734	-0.04889	-0.05292	-0.04914	-0.04520	-0.05098	-0.04726	-0.05202	-0.05525							
1024	-0.08317	-0.08156	-0.08174	-0.08676	-0.08465	-0.07875	-0.08449	-0.07991	-0.08808								
512	-0.09846	-0.10110	-0.10229	-0.11012	-0.10892	-0.10461	-0.11110	-0.10761									
256	-0.10334	-0.10889	-0.11133	-0.11785	-0.11766	-0.11342	-0.12413										
128	-0.09083	-0.09744	-0.10146	-0.11367	-0.11630	-0.11122											
64	-0.08211	-0.08738	-0.09681	-0.10580	-0.11023												
32	-0.07063	-0.08106	-0.08651	-0.09555													
16	-0.06605	-0.07860	-0.08728														
8	-0.08044	-0.09170															
4	-0.13255																

(f) $\mathcal{P}^{\mathcal{C}}$ for Scene: [**Torus**] Algorithm: [**ERPT**] Metric: [**FSIM**] True GT: [**BDPT @ 524288 spp**].

M-ERPT	2	4	8	16	32	64	128	256	512	1024	2048	4096	8192	16384	32768	65536	131072
GT (BDPT)																	
262144	0.00194	0.00149	0.00201	0.00213	0.00249	0.00225	0.00233	0.00227	0.00250	0.00251	0.00250	0.00247	0.00235	0.00249	0.00246	0.00257	0.00249
131072	0.00518	0.00434	0.00575	0.00646	0.00694	0.00703	0.00696	0.00695	0.00690	0.00724	0.00702	0.00745	0.00742	0.00725	0.00738	0.00719	
65536	0.00812	0.00663	0.00767	0.00917	0.01004	0.01024	0.01093	0.01027	0.01043	0.01073	0.01117	0.01143	0.01050	0.01079	0.01053		
32768	0.01044	0.00829	0.00994	0.01160	0.01289	0.01071	0.01191	0.01185	0.01150	0.01218	0.01147	0.01267	0.01216	0.01194			
16384	0.00910	0.00619	0.00628	0.00936	0.01053	0.00765	0.00897	0.00837	0.00804	0.00819	0.00775	0.00868	0.00755				
8192	0.00246	-0.00282	-0.00331	-0.00102	0.00088	-0.00418	-0.00484	-0.00500	-0.00582	-0.00517	-0.00674	-0.00444					
4096	-0.01165	-0.01936	-0.02117	-0.01834	-0.01831	-0.02600	-0.02351	-0.02666	-0.02850	-0.02723	-0.02799						
2048	-0.03430	-0.04441	-0.04967	-0.04730	-0.04822	-0.05797	-0.05879	-0.06193	-0.06419	-0.06318							
1024	-0.06464	-0.07340	-0.07780	-0.08018	-0.08329	-0.09455	-0.09779	-0.09905	-0.10235								
512	-0.07977	-0.09256	-0.10063	-0.10481	-0.10889	-0.12029	-0.12305	-0.12869									
256	-0.08806	-0.09718	-0.10864	-0.11353	-0.11676	-0.12917	-0.13440										
128	-0.07911	-0.08968	-0.10226	-0.11005	-0.11206	-0.12448											
64	-0.07226	-0.08294	-0.09484	-0.10458	-0.10264												
32	-0.06503	-0.07709	-0.08631	-0.09561													
16	-0.06178	-0.07438	-0.08036														
8	-0.07404	-0.08275															
4	-0.12204																

(g) $\mathcal{P}^{\mathcal{C}}$ for Scene: [**Torus**] Algorithm: [**M-ERPT**] Metric: [**FSIM**] True GT: [**BDPT @ 524288 spp**].

Table 42

10.3 Veach Bidir

PT	2	4	8	16	32	64	128	256	512	1024	2048	4096	8192	16384	32768	65536	131072
GT (BDPT)																	
262144	0.00018	0.00019	0.00015	0.00017	0.00018	0.00016	0.00015	0.00011	0.00014	0.00011	0.00006	0.00008	0.00001	0.00005	0.00002	0.00004	0.00003
131072	0.00069	0.00057	0.00046	0.00044	0.00042	0.00040	0.00033	0.00028	0.00029	0.00021	0.00014	0.00017	0.00011	0.00010	0.00007	0.00008	
65536	0.00137	0.00122	0.00112	0.00107	0.00099	0.00090	0.00090	0.00076	0.00070	0.00059	0.00061	0.00055	0.00047	0.00040	0.00030		
32768	0.00279	0.00255	0.00241	0.00220	0.00213	0.00196	0.00180	0.00163	0.00149	0.00136	0.00109	0.00105	0.00090	0.00082			
16384	0.00398	0.00362	0.00333	0.00317	0.00289	0.00267	0.00263	0.00229	0.00206	0.00191	0.00160	0.00155	0.00126				
8192	0.00743	0.00685	0.00625	0.00595	0.00548	0.00514	0.00480	0.00431	0.00397	0.00345	0.00305	0.00259					
4096	0.01231	0.01132	0.01023	0.00975	0.00890	0.00796	0.00758	0.00684	0.00604	0.00556	0.00479						
2048	0.02063	0.01900	0.01785	0.01621	0.01496	0.01364	0.01273	0.01148	0.01011	0.00912							
1024	0.03857	0.03560	0.03289	0.03049	0.02761	0.02506	0.02347	0.02089	0.01890								
512	0.06422	0.05950	0.05495	0.04994	0.04539	0.04112	0.03795	0.03330									
256	0.10442	0.09656	0.08812	0.08010	0.07264	0.06492	0.05953										
128	0.15317	0.14193	0.12876	0.11631	0.10425	0.09258											
64	0.21431	0.19784	0.17969	0.16130	0.14202												
32	0.29825	0.27412	0.24622	0.21821													
16	0.38687	0.35311	0.31462														
8	0.49526	0.44712															
4	0.60102																

(a) $\mathcal{P}^{\mathcal{C}}$ for Scene: [**Veach Bidir**] Algorithm: [**PT**] Metric: [**FSIM**] True GT: [**BDPT @ 524288 spp**].

BDPT	2	4	8	16	32	64	128	256	512	1024	2048	4096	8192	16384	32768	65536	131072
GT (BDPT)																	
262144	0.00015	0.00013	0.00007	0.00010	0.00007	0.00003	0.00005	0.00005	0.00001	0.00002	-0.00003	-0.00002	-0.00000	-0.00000	-0.00000	-0.00001	-0.00001
131072	0.00041	0.00034	0.00024	0.00020	0.00014	0.00011	0.00007	0.00005	0.00003	-0.00001	-0.00002	-0.00002	-0.00000	-0.00001	-0.00001	-0.00003	
65536	0.00092	0.00081	0.00070	0.00063	0.00051	0.00037	0.00032	0.00023	0.00015	0.00006	0.00001	-0.00003	-0.00000	-0.00004	-0.00005		
32768	0.00191	0.00166	0.00143	0.00122	0.00104	0.00077	0.00058	0.00043	0.00028	0.00010	0.00002	-0.00009	-0.00009	-0.00010			
16384	0.00281	0.00246	0.00216	0.00177	0.00155	0.00126	0.00098	0.00064	0.00043	0.00014	-0.00002	-0.00019	-0.00021				
8192	0.00506	0.00448	0.00395	0.00322	0.00262	0.00213	0.00156	0.00112	0.00050	0.00017	-0.00017	-0.00034					
4096	0.00817	0.00716	0.00607	0.00504	0.00402	0.00319	0.00239	0.00172	0.00077	0.00018	-0.00023						
2048	0.01397	0.01208	0.01024	0.00841	0.00681	0.00537	0.00396	0.00258	0.00148	0.00021							
1024	0.02567	0.02209	0.01870	0.01515	0.01217	0.00931	0.00660	0.00413	0.00166								
512	0.04239	0.03566	0.02966	0.02368	0.01811	0.01315	0.00863	0.00456									
256	0.06743	0.05642	0.04600	0.03499	0.02595	0.01731	0.00993										
128	0.09791	0.07975	0.06323	0.04615	0.03186	0.01838											
64	0.13338	0.10752	0.08181	0.05596	0.03521												
32	0.17969	0.13991	0.10170	0.06275													
16	0.22224	0.16439	0.11072														
8	0.26308	0.18202															
4	0.28887																

(b) $\mathcal{P}^{\mathcal{C}}$ for Scene: [**Veach Bidir**] Algorithm: [**BDPT**] Metric: [**FSIM**] True GT: [**BDPT @ 524288 spp**].

PSSMLT	2	4	8	16	32	64	128	256	512	1024	2048	4096	8192	16384
GT (BDPT)														
262144	0.00015	0.00011	0.00010	0.00010	0.00008	0.00009	0.00008	0.00003	0.00003	0.00003	0.00001	0.00002	0.00001	0.00001
131072	0.00044	0.00032	0.00027	0.00022	0.00020	0.00016	0.00014	0.00008	0.00006	0.00002	0.00003	-0.00000	-0.00001	-0.00001
65536	0.00096	0.00077	0.00066	0.00057	0.00050	0.00040	0.00034	0.00025	0.00022	0.00014	0.00010	0.00007	0.00003	-0.00001
32768	0.00184	0.00167	0.00137	0.00126	0.00103	0.00084	0.00070	0.00052	0.00040	0.00030	0.00019	0.00005	0.00001	-0.00001
16384	0.00265	0.00235	0.00197	0.00176	0.00151	0.00130	0.00106	0.00075	0.00055	0.00037	0.00016	0.00004	-0.00007	
8192	0.00508	0.00451	0.00373	0.00335	0.00275	0.00222	0.00176	0.00139	0.00105	0.00061	0.00028	0.00011		
4096	0.00832	0.00711	0.00613	0.00521	0.00431	0.00347	0.00276	0.00204	0.00138	0.00077	0.00031			
2048	0.01385	0.01185	0.01016	0.00869	0.00708	0.00571	0.00458	0.00338	0.00228	0.00123				
1024	0.02574	0.02214	0.01882	0.01594	0.01279	0.01018	0.00768	0.00531	0.00335					
512	0.04248	0.03608	0.02974	0.02446	0.01902	0.01455	0.01036	0.00650						
256	0.06820	0.05747	0.04662	0.03726	0.02771	0.01986	0.01301							
128	0.09848	0.08129	0.06408	0.04935	0.03456	0.02262								
64	0.13476	0.10962	0.08430	0.06179	0.03977									
32	0.18072	0.14299	0.10537	0.07026										
16	0.22471	0.17044	0.11726											
8	0.26708	0.19230												
4	0.29515													

(c) $\mathcal{P}^{\mathcal{C}}$ for Scene: [**Veach Bidir**] Algorithm: [**PSSMLT**] Metric: [**FSIM**] True GT: [**BDPT @ 524288 spp**].

MLT	2	4	8	16	32	64	128	256	512	1024	2048	4096	8192	16384
GT (BDPT)														
262144	0.00016	0.00018	0.00018	0.00012	0.00012	0.00011	0.00009	0.00006	0.00005	0.00005	0.00004	0.00002	0.00001	0.00002
131072	0.00053	0.00045	0.00043	0.00034	0.00031	0.00024	0.00018	0.00014	0.00012	0.00011	0.00007	0.00003	0.00001	0.00000
65536	0.00106	0.00096	0.00093	0.00081	0.00072	0.00064	0.00058	0.00048	0.00040	0.00029	0.00022	0.00012	0.00009	0.00006
32768	0.00230	0.00213	0.00195	0.00169	0.00158	0.00132	0.00111	0.00090	0.00076	0.00059	0.00042	0.00023	0.00014	0.00005
16384	0.00333	0.00303	0.00269	0.00244	0.00220	0.00192	0.00170	0.00144	0.00120	0.00093	0.00059	0.00038	0.00025	
8192	0.00622	0.00562	0.00501	0.00433	0.00381	0.00343	0.00302	0.00252	0.00209	0.00163	0.00108	0.00059		
4096	0.01049	0.00921	0.00818	0.00709	0.00609	0.00519	0.00448	0.00372	0.00301	0.00216	0.00144			
2048	0.01749	0.01556	0.01370	0.01200	0.01038	0.00888	0.00752	0.00623	0.00493	0.00343				
1024	0.03234	0.02903	0.02564	0.02238	0.01929	0.01629	0.01353	0.01111	0.00860					
512	0.05388	0.04812	0.04217	0.03652	0.03093	0.02538	0.02041	0.01614						
256	0.08833	0.07886	0.06881	0.05855	0.04901	0.03903	0.03053							
128	0.13098	0.11634	0.10068	0.08522	0.06954	0.05371								
64	0.18400	0.16407	0.14074	0.11636	0.09246									
32	0.25471	0.22618	0.19193	0.15614										
16	0.32671	0.28994	0.24221											
8	0.41314	0.36091												
4	0.49420													

(d) $\mathcal{P}^{\mathcal{C}}$ for Scene: [**Veach Bidir**] Algorithm: [**MLT**] Metric: [**FSIM**] True GT: [**BDPT @ 524288 spp**].

M-MLT	2	4	8	16	32	64	128	256	512	1024	2048	4096	8192	16384
GT (BDPT)														
262144	0.00017	0.00014	0.00013	0.00011	0.00010	0.00008	0.00008	0.00007	0.00007	0.00004	0.00002	0.00002	0.00002	0.00000
131072	0.00057	0.00048	0.00041	0.00033	0.00030	0.00023	0.00014	0.00011	0.00010	0.00008	0.00003	0.00003	0.00000	-0.00000
65536	0.00116	0.00093	0.00085	0.00077	0.00073	0.00061	0.00051	0.00049	0.00041	0.00031	0.00018	0.00013	0.00008	0.00003
32768	0.00234	0.00204	0.00187	0.00161	0.00149	0.00128	0.00103	0.00091	0.00074	0.00050	0.00035	0.00019	0.00009	-0.00001
16384	0.00326	0.00298	0.00263	0.00240	0.00212	0.00188	0.00161	0.00141	0.00115	0.00085	0.00058	0.00041	0.00024	
8192	0.00610	0.00551	0.00491	0.00429	0.00369	0.00341	0.00282	0.00243	0.00189	0.00146	0.00092	0.00050		
4096	0.01030	0.00903	0.00796	0.00686	0.00614	0.00522	0.00433	0.00363	0.00284	0.00215	0.00127			
2048	0.01731	0.01518	0.01351	0.01162	0.01031	0.00890	0.00730	0.00618	0.00481	0.00341				
1024	0.03179	0.02824	0.02505	0.02170	0.01869	0.01595	0.01304	0.01088	0.00821					
512	0.05326	0.04720	0.04139	0.03511	0.03015	0.02501	0.01962	0.01549						
256	0.08707	0.07737	0.06688	0.05629	0.04748	0.03833	0.02938							
128	0.12858	0.11286	0.09865	0.08168	0.06681	0.05164								
64	0.18070	0.15848	0.13675	0.11112	0.08921									
32	0.25002	0.21911	0.18750	0.14897										
16	0.32302	0.28143	0.23594											
8	0.40775	0.34989												
4	0.48722													

(e) $\mathcal{P}^{\mathcal{C}}$ for Scene: [**Veach Bidir**] Algorithm: [**M-MLT**] Metric: [**FSIM**] True GT: [**BDPT @ 524288 spp**].

ERPT	2	4	8	16	32	64	128	256	512	1024	2048	4096	8192	16384	32768	65536	131072
GT (BDPT)																	
262144	0.00009	0.00009	0.00009	0.00005	0.00007	0.00008	0.00008	0.00006	0.00005	0.00006	0.00006	0.00006	0.00008	0.00007	0.00001	0.00006	0.00006
131072	0.00018	0.00020	0.00018	0.00013	0.00016	0.00020	0.00016	0.00018	0.00012	0.00019	0.00018	0.00018	0.00013	0.00016	0.00013	0.00021	
65536	0.00052	0.00052	0.00054	0.00051	0.00051	0.00050	0.00050	0.00049	0.00048	0.00045	0.00047	0.00046	0.00050	0.00050	0.00047		
32768	0.00106	0.00103	0.00101	0.00104	0.00103	0.00097	0.00099	0.00104	0.00097	0.00106	0.00104	0.00099	0.00103	0.00104			
16384	0.00160	0.00161	0.00153	0.00151	0.00152	0.00157	0.00151	0.00142	0.00142	0.00145	0.00150	0.00148	0.00147				
8192	0.00277	0.00277	0.00273	0.00285	0.00275	0.00270	0.00279	0.00281	0.00274	0.00263	0.00264	0.00264					
4096	0.00420	0.00438	0.00429	0.00429	0.00423	0.00420	0.00426	0.00428	0.00418	0.00429	0.00435						
2048	0.00719	0.00705	0.00725	0.00707	0.00729	0.00712	0.00716	0.00706	0.00704	0.00713							
1024	0.01285	0.01276	0.01280	0.01281	0.01287	0.01260	0.01275	0.01230	0.01250								
512	0.01935	0.01917	0.01928	0.01905	0.01912	0.01879	0.01913	0.01843									
256	0.02829	0.02820	0.02797	0.02821	0.02826	0.02806	0.02785										
128	0.03595	0.03592	0.03538	0.03564	0.03549	0.03604											
64	0.04202	0.04169	0.04187	0.04201	0.04182												
32	0.04390	0.04182	0.04225	0.04185													
16	0.03371	0.03131	0.03159														
8	0.00339	0.00021															
4	-0.05264																

(f) $\mathcal{P}^{\mathcal{C}}$ for Scene: [**Veach Bidir**] Algorithm: [**ERPT**] Metric: [**FSIM**] True GT: [**BDPT @ 524288 spp**].

M-ERPT	2	4	8	16	32	64	128	256	512	1024	2048	4096	8192	16384	32768	65536	131072
GT (BDPT)																	
262144	0.00011	0.00008	0.00005	0.00008	0.00007	0.00007	0.00005	0.00009	0.00002	0.00006	0.00008	0.00007	0.00005	0.00005	0.00006	0.00004	0.00006
131072	0.00017	0.00015	0.00012	0.00017	0.00016	0.00020	0.00014	0.00016	0.00017	0.00013	0.00017	0.00015	0.00017	0.00014	0.00017	0.00015	
65536	0.00057	0.00055	0.00052	0.00055	0.00050	0.00054	0.00042	0.00048	0.00047	0.00050	0.00046	0.00051	0.00049	0.00047	0.00050		
32768	0.00103	0.00100	0.00102	0.00107	0.00106	0.00101	0.00092	0.00098	0.00107	0.00107	0.00101	0.00096	0.00105	0.00099			
16384	0.00157	0.00154	0.00144	0.00160	0.00152	0.00147	0.00142	0.00139	0.00153	0.00140	0.00146	0.00144	0.00139				
8192	0.00277	0.00268	0.00275	0.00286	0.00270	0.00275	0.00259	0.00257	0.00266	0.00267	0.00266	0.00275					
4096	0.00429	0.00429	0.00430	0.00441	0.00432	0.00436	0.00410	0.00413	0.00418	0.00432	0.00429						
2048	0.00726	0.00727	0.00721	0.00723	0.00722	0.00710	0.00706	0.00681	0.00722	0.00716							
1024	0.01294	0.01300	0.01273	0.01314	0.01283	0.01253	0.01197	0.01195	0.01252								
512	0.01957	0.02006	0.01902	0.01966	0.01917	0.01888	0.01791	0.01825									
256	0.02891	0.02894	0.02852	0.02849	0.02867	0.02814	0.02683										
128	0.03677	0.03679	0.03557	0.03680	0.03599	0.03618											
64	0.04395	0.04323	0.04287	0.04354	0.04246												
32	0.04556	0.04551	0.04548	0.04586													
16	0.03627	0.03542	0.03574														
8	0.00662	0.00394															
4	-0.04765																

(g) $\mathcal{P}^{\mathcal{C}}$ for Scene: [**Veach Bidir**] Algorithm: [**M-ERPT**] Metric: [**FSIM**] True GT: [**BDPT @ 524288 spp**].

Table 43

10.4 Veach Door

PT	2	4	8	16	32	64	128	256	512	1024	2048	4096	8192	16384	32768	65536	131072
GT (BDPT)																	
262144	0.00028	0.00005	0.00022	0.00045	0.00049	0.00063	0.00064	0.00064	0.00066	0.00059	0.00046	0.00039	0.00021	0.00028	0.00014	0.00005	0.00004
131072	0.00070	0.00038	0.00069	0.00089	0.00140	0.00185	0.00208	0.00212	0.00198	0.00173	0.00158	0.00115	0.00095	0.00081	0.00044	0.00023	
65536	0.00174	0.00201	0.00235	0.00297	0.00389	0.00463	0.00491	0.00489	0.00443	0.00394	0.00340	0.00275	0.00189	0.00140	0.00086		
32768	0.00232	0.00276	0.00387	0.00499	0.00677	0.00827	0.00901	0.00864	0.00822	0.00716	0.00593	0.00459	0.00330	0.00239			
16384	0.00639	0.00688	0.00880	0.00968	0.01281	0.01474	0.01606	0.01540	0.01402	0.01234	0.01028	0.00786	0.00530				
8192	0.00962	0.01159	0.01362	0.01679	0.02084	0.02451	0.02616	0.02482	0.02264	0.01921	0.01552	0.01138					
4096	0.01301	0.01523	0.01975	0.02368	0.03077	0.03672	0.03835	0.03646	0.03317	0.02749	0.02162						
2048	0.01985	0.02538	0.03133	0.03573	0.04595	0.05334	0.05538	0.05206	0.04575	0.03666							
1024	0.03294	0.04286	0.05038	0.05805	0.06977	0.07959	0.07955	0.07290	0.06156								
512	0.05419	0.06508	0.07618	0.08461	0.09685	0.10599	0.10420	0.09132									
256	0.09110	0.10619	0.11512	0.12013	0.13122	0.13711	0.12749										
128	0.14225	0.15641	0.16375	0.16305	0.17001	0.16657											
64	0.20908	0.21978	0.22251	0.21154	0.20321												
32	0.29823	0.30497	0.29012	0.25869													
16	0.39998	0.39538	0.35400														
8	0.50192	0.47789															
4	0.58720																

(a) $\mathcal{P}^{\mathcal{C}}$ for Scene: [**Veach Door**] Algorithm: [**PT**] Metric: [**FSIM**] True GT: [**BDPT @ 524288 spp**].

BDPT	2	4	8	16	32	64	128	256	512	1024	2048	4096	8192	16384	32768	65536	131072
GT (BDPT)																	
262144	0.00013	0.00031	0.00039	0.00036	0.00068	0.00067	0.00070	0.00062	0.00049	0.00036	0.00026	0.00019	0.00006	0.00005	0.00000	-0.00003	-0.00006
131072	0.00049	0.00074	0.00097	0.00132	0.00179	0.00185	0.00195	0.00187	0.00157	0.00111	0.00084	0.00055	0.00029	0.00008	-0.00002	-0.00013	
65536	0.00217	0.00243	0.00324	0.00377	0.00435	0.00473	0.00444	0.00391	0.00327	0.00231	0.00161	0.00099	0.00047	0.00013	-0.00020		
32768	0.00327	0.00427	0.00528	0.00602	0.00805	0.00863	0.00829	0.00698	0.00584	0.00425	0.00280	0.00165	0.00054	-0.00018			
16384	0.00731	0.00810	0.01009	0.01228	0.01454	0.01451	0.01364	0.01184	0.00963	0.00687	0.00433	0.00232	0.00044				
8192	0.01070	0.01288	0.01662	0.02041	0.02355	0.02387	0.02224	0.01861	0.01478	0.01000	0.00564	0.00203					
4096	0.01445	0.01766	0.02337	0.02986	0.03474	0.03474	0.03226	0.02666	0.02018	0.01274	0.00549						
2048	0.02512	0.02831	0.03661	0.04406	0.05055	0.04945	0.04475	0.03554	0.02557	0.01312							
1024	0.04179	0.04717	0.05821	0.06722	0.07386	0.07006	0.05954	0.04335	0.02760								
512	0.06477	0.07152	0.08354	0.09356	0.09857	0.08883	0.07206	0.04725									
256	0.10493	0.10896	0.11979	0.12408	0.12472	0.10583	0.07669										
128	0.15462	0.15591	0.16024	0.15886	0.14691	0.11391											
64	0.21734	0.21322	0.20788	0.18670	0.15860												
32	0.30118	0.28172	0.25069	0.20757													
16	0.38600	0.34462	0.28284														
8	0.47044	0.38959															
4	0.51825																

(b) $\mathcal{P}^{\mathcal{C}}$ for Scene: [**Veach Door**] Algorithm: [**BDPT**] Metric: [**FSIM**] True GT: [**BDPT @ 524288 spp**].

PSSMLT	2	4	8	16	32	64	128	256	512	1024	2048	4096	8192	16384
GT (BDPT)														
262144	0.00027	0.00050	0.00055	0.00057	0.00052	0.00059	0.00048	0.00041	0.00030	0.00022	0.00009	0.00007	0.00002	-0.00004
131072	0.00088	0.00097	0.00134	0.00146	0.00164	0.00176	0.00154	0.00140	0.00109	0.00078	0.00048	0.00027	0.00008	-0.00005
65536	0.00256	0.00281	0.00348	0.00370	0.00389	0.00392	0.00347	0.00297	0.00219	0.00151	0.00085	0.00041	0.00011	-0.00018
32768	0.00445	0.00496	0.00628	0.00678	0.00723	0.00728	0.00623	0.00526	0.00387	0.00265	0.00154	0.00064	-0.00008	-0.00053
16384	0.00903	0.01001	0.01164	0.01208	0.01227	0.01237	0.01039	0.00847	0.00622	0.00407	0.00209	0.00054	-0.00060	
8192	0.01493	0.01672	0.01909	0.01984	0.02019	0.01933	0.01606	0.01277	0.00869	0.00498	0.00186	-0.00067		
4096	0.02201	0.02488	0.02869	0.02880	0.02857	0.02848	0.02289	0.01703	0.01095	0.00498	0.00019			
2048	0.03378	0.03815	0.04233	0.04166	0.04037	0.03842	0.02970	0.02102	0.01090	0.00223				
1024	0.05388	0.05782	0.06338	0.06115	0.05641	0.05101	0.03595	0.02098	0.00589					
512	0.07867	0.08311	0.08755	0.08029	0.07011	0.05834	0.03577	0.01365						
256	0.11394	0.11455	0.11586	0.10045	0.08063	0.06052	0.02759							
128	0.16100	0.15373	0.14240	0.11553	0.08360	0.04928								
64	0.21225	0.19359	0.16513	0.12098	0.07117									
32	0.26655	0.22627	0.17222	0.10733										
16	0.31457	0.24494	0.15747											
8	0.33844	0.23348												
4	0.31761													

(c) $\mathcal{P}^{\mathcal{C}}$ for Scene: [**Veach Door**] Algorithm: [**PSSMLT**] Metric: [**FSIM**] True GT: [**BDPT @ 524288 spp**].

MLT	2	4	8	16	32	64	128	256	512	1024	2048	4096	8192	16384
GT (BDPT)														
262144	0.00023	0.00040	0.00047	0.00054	0.00061	0.00048	0.00052	0.00032	0.00036	0.00025	0.00016	0.00008	0.00001	-0.00001
131072	0.00068	0.00120	0.00139	0.00170	0.00170	0.00162	0.00158	0.00125	0.00103	0.00071	0.00036	0.00020	0.00004	-0.00007
65536	0.00211	0.00318	0.00335	0.00401	0.00392	0.00336	0.00331	0.00247	0.00196	0.00133	0.00080	0.00035	-0.00001	-0.00023
32768	0.00364	0.00515	0.00625	0.00715	0.00692	0.00619	0.00608	0.00471	0.00345	0.00215	0.00113	0.00023	-0.00038	-0.00077
16384	0.00740	0.01009	0.01165	0.01283	0.01232	0.01070	0.00994	0.00738	0.00539	0.00324	0.00126	-0.00003	-0.00103	
8192	0.01209	0.01702	0.01952	0.02056	0.01953	0.01658	0.01553	0.01089	0.00731	0.00381	0.00070	-0.00162		
4096	0.01667	0.02461	0.02740	0.02979	0.02857	0.02358	0.02146	0.01404	0.00866	0.00304	-0.00202			
2048	0.02461	0.03688	0.04095	0.04255	0.03957	0.03130	0.02725	0.01609	0.00762	-0.00092				
1024	0.04042	0.05603	0.06023	0.06093	0.05314	0.03859	0.03182	0.01462	0.00140					
512	0.05999	0.07992	0.08305	0.07849	0.06512	0.04207	0.02935	0.00393						
256	0.09140	0.11068	0.10665	0.09517	0.07201	0.03884	0.01778							
128	0.13036	0.14630	0.12937	0.10520	0.06813	0.02144								
64	0.17739	0.18033	0.14875	0.10615	0.05065									
32	0.22906	0.20965	0.14956	0.08594										
16	0.27566	0.22621	0.13461											
8	0.29643	0.20556												
4	0.28032													

(d) $\mathcal{P}^{\mathcal{C}}$ for Scene: [**Veach Door**] Algorithm: [**MLT**] Metric: [**FSIM**] True GT: [**BDPT @ 524288 spp**].

M-MLT	2	4	8	16	32	64	128	256	512	1024	2048	4096	8192	16384
GT (BDPT)														
262144	0.00032	0.00037	0.00038	0.00047	0.00063	0.00054	0.00047	0.00033	0.00025	0.00019	0.00006	0.00009	-0.00001	-0.00002
131072	0.00098	0.00126	0.00150	0.00143	0.00168	0.00164	0.00148	0.00117	0.00083	0.00065	0.00035	0.00019	-0.00002	-0.00008
65536	0.00271	0.00363	0.00364	0.00360	0.00395	0.00357	0.00316	0.00239	0.00175	0.00122	0.00064	0.00025	-0.00015	-0.00030
32768	0.00469	0.00591	0.00622	0.00643	0.00712	0.00606	0.00558	0.00435	0.00313	0.00181	0.00105	0.00014	-0.00053	-0.00078
16384	0.00985	0.01157	0.01180	0.01193	0.01220	0.01063	0.00933	0.00674	0.00494	0.00273	0.00116	-0.00032	-0.00128	
8192	0.01593	0.01908	0.01983	0.01904	0.01946	0.01692	0.01425	0.00988	0.00652	0.00314	0.00038	-0.00189		
4096	0.02302	0.02805	0.02899	0.02789	0.02802	0.02391	0.01954	0.01302	0.00727	0.00191	-0.00244			
2048	0.03531	0.04132	0.04266	0.04027	0.03911	0.03179	0.02408	0.01447	0.00543	-0.00305				
1024	0.05414	0.06403	0.06236	0.05694	0.05278	0.04014	0.02778	0.01169	-0.00204					
512	0.07875	0.08911	0.08406	0.07129	0.06309	0.04356	0.02329	0.00087						
256	0.11451	0.12151	0.11009	0.08764	0.06844	0.03830	0.01032							
128	0.15802	0.15459	0.13118	0.09709	0.06496	0.02245								
64	0.20620	0.19192	0.14541	0.09520	0.04600									
32	0.25496	0.21670	0.14630	0.07493										
16	0.29883	0.23026	0.12332											
8	0.31433	0.20705												
4	0.29441													

(e) $\mathcal{P}^{\mathcal{C}}$ for Scene: [**Veach Door**] Algorithm: [**M-MLT**] Metric: [**FSIM**] True GT: [**BDPT @ 524288 spp**].

ERPT	2	4	8	16	32	64	128	256	512	1024	2048	4096	8192	16384	32768	65536	131072
GT (BDPT)																	
262144	0.00044	0.00050	0.00051	0.00047	0.00048	0.00043	0.00041	0.00044	0.00042	0.00045	0.00038	0.00043	0.00044	0.00047	0.00042	0.00039	0.00045
131072	0.00134	0.00150	0.00144	0.00147	0.00145	0.00140	0.00136	0.00142	0.00144	0.00136	0.00142	0.00140	0.00141	0.00155	0.00142	0.00141	
65536	0.00276	0.00300	0.00293	0.00296	0.00296	0.00299	0.00288	0.00301	0.00290	0.00287	0.00293	0.00294	0.00296	0.00303	0.00283		
32768	0.00500	0.00546	0.00545	0.00559	0.00543	0.00544	0.00533	0.00536	0.00536	0.00533	0.00545	0.00522	0.00540	0.00565			
16384	0.00855	0.00864	0.00909	0.00893	0.00895	0.00877	0.00873	0.00887	0.00885	0.00856	0.00875	0.00884	0.00919				
8192	0.01306	0.01325	0.01353	0.01374	0.01357	0.01335	0.01304	0.01341	0.01317	0.01277	0.01343	0.01318					
4096	0.01754	0.01821	0.01874	0.01895	0.01863	0.01853	0.01805	0.01852	0.01828	0.01761	0.01780						
2048	0.02097	0.02212	0.02274	0.02240	0.02324	0.02247	0.02198	0.02266	0.02257	0.02043							
1024	0.02270	0.02394	0.02487	0.02439	0.02476	0.02372	0.02374	0.02472	0.02458								
512	0.01720	0.01801	0.01931	0.01863	0.01914	0.01851	0.01770	0.01899									
256	0.00180	0.00354	0.00312	0.00380	0.00403	0.00343	0.00317										
128	-0.02810	-0.02507	-0.02622	-0.02706	-0.02738	-0.02805											
64	-0.07586	-0.07378	-0.07485	-0.07672	-0.07789												
32	-0.14066	-0.14007	-0.14475	-0.14433													
16	-0.22329	-0.22765	-0.23250														
8	-0.33862	-0.33980															
4	-0.49065																

(f) $\mathcal{P}^{\mathcal{C}}$ for Scene: [**Veach Door**] Algorithm: [**ERPT**] Metric: [**FSIM**] True GT: [**BDPT @ 524288 spp**].

M-ERPT	2	4	8	16	32	64	128	256	512	1024	2048	4096	8192	16384	32768	65536	131072
GT (BDPT)																	
262144	0.00053	0.00044	0.00045	0.00046	0.00045	0.00045	0.00050	0.00046	0.00049	0.00047	0.00048	0.00045	0.00045	0.00044	0.00041	0.00044	0.00040
131072	0.00148	0.00143	0.00133	0.00142	0.00147	0.00136	0.00139	0.00148	0.00150	0.00139	0.00150	0.00140	0.00138	0.00151	0.00142	0.00144	
65536	0.00309	0.00295	0.00298	0.00291	0.00296	0.00305	0.00299	0.00298	0.00314	0.00304	0.00311	0.00300	0.00291	0.00312	0.00293		
32768	0.00566	0.00530	0.00555	0.00520	0.00539	0.00550	0.00548	0.00553	0.00565	0.00546	0.00561	0.00559	0.00551	0.00537			
16384	0.00929	0.00894	0.00872	0.00884	0.00891	0.00904	0.00906	0.00908	0.00925	0.00867	0.00913	0.00924	0.00903				
8192	0.01440	0.01382	0.01365	0.01335	0.01345	0.01383	0.01397	0.01388	0.01396	0.01319	0.01402	0.01401					
4096	0.01972	0.01861	0.01832	0.01819	0.01853	0.01891	0.01878	0.01891	0.01917	0.01848	0.01945						
2048	0.02497	0.02299	0.02269	0.02279	0.02289	0.02367	0.02286	0.02355	0.02406	0.02252							
1024	0.02802	0.02515	0.02469	0.02416	0.02439	0.02571	0.02549	0.02598	0.02631								
512	0.02339	0.02035	0.01933	0.01893	0.01953	0.02081	0.01987	0.02099									
256	0.00964	0.00616	0.00449	0.00332	0.00529	0.00510	0.00399										
128	-0.01929	-0.02240	-0.02440	-0.02611	-0.02542	-0.02302											
64	-0.06700	-0.07076	-0.07146	-0.07530	-0.07355												
32	-0.13306	-0.13730	-0.13875	-0.14358													
16	-0.21924	-0.22123	-0.22658														
8	-0.33652	-0.33919															
4	-0.48698																

(g) $\mathcal{P}^{\mathcal{C}}$ for Scene: [**Veach Door**] Algorithm: [**M-ERPT**] Metric: [**FSIM**] True GT: [**BDPT @ 524288 spp**].

Table 44

10.5 Sponza

PT	2	4	8	16	32	64	128	256	512	1024	2048	4096	8192
**GT (PT)**													
16384	0.00365	0.00373	0.00369	0.00309	0.00294	0.00239	0.00175	0.00102	0.00069	0.00028	0.00005	-0.00024	-0.00035
8192	0.00938	0.00881	0.00844	0.00805	0.00691	0.00545	0.00429	0.00281	0.00133	0.00045	-0.00046	-0.00089	
4096	0.02125	0.02187	0.02026	0.01845	0.01633	0.01293	0.00940	0.00568	0.00300	0.00052	-0.00112		
2048	0.03999	0.03948	0.03711	0.03331	0.02884	0.02293	0.01589	0.00932	0.00404	-0.00028			
1024	0.07152	0.06979	0.06519	0.05822	0.04875	0.03765	0.02526	0.01344	0.00390				
512	0.11536	0.11292	0.10417	0.09039	0.07523	0.05566	0.03437	0.01498					
256	0.17510	0.17056	0.15484	0.13279	0.10440	0.07428	0.04130						
128	0.24603	0.23792	0.21085	0.17699	0.13282	0.08470							
64	0.32658	0.30982	0.26799	0.21641	0.14873								
32	0.40975	0.37931	0.31749	0.23923									
16	0.48893	0.43968	0.34980										
8	0.55299	0.47617											
4	0.58729												

(a) $\mathcal{P}^{\mathcal{C}}$ for Scene: [**Sponza**] Algorithm: [**PT**] Metric: [**FSIM**] True GT: [**PT @ 32768 spp**].

BDPT	2	4	8	16	32	64	128	256	512	1024	2048	4096	8192
**GT (PT)**													
16384	0.00350	0.00388	0.00353	0.00341	0.00308	0.00253	0.00197	0.00137	0.00090	0.00038	0.00012	-0.00015	-0.00036
8192	0.00883	0.00903	0.00876	0.00813	0.00749	0.00604	0.00455	0.00315	0.00176	0.00058	-0.00010	-0.00072	
4096	0.02118	0.02160	0.02082	0.01943	0.01677	0.01385	0.01056	0.00721	0.00391	0.00135	-0.00075		
2048	0.03843	0.03998	0.03835	0.03491	0.03080	0.02487	0.01802	0.01178	0.00560	0.00072			
1024	0.06934	0.07037	0.06778	0.06093	0.05266	0.04151	0.02957	0.01707	0.00672				
512	0.11235	0.11328	0.10771	0.09645	0.08138	0.06289	0.04113	0.02091					
256	0.16910	0.17214	0.16022	0.14150	0.11737	0.08463	0.04997						
128	0.23811	0.24176	0.21976	0.19020	0.14986	0.10179							
64	0.32023	0.31794	0.28434	0.23783	0.17676								
32	0.40272	0.38973	0.34067	0.26965									
16	0.48572	0.45677	0.38150										
8	0.55510	0.50109											
4	0.60377												

(b) $\mathcal{P}^{\mathcal{C}}$ for Scene: [**Sponza**] Algorithm: [**BDPT**] Metric: [**FSIM**] True GT: [**PT @ 32768 spp**].

PSSMLT	2	4	8	16	32	64	128	256	512	1024	2048	4096	8192
**GT (PT)**													
16384	0.00353	0.00371	0.00364	0.00321	0.00304	0.00252	0.00194	0.00140	0.00110	0.00068	0.00045	0.00017	0.00010
8192	0.00887	0.00918	0.00860	0.00768	0.00702	0.00603	0.00457	0.00346	0.00235	0.00129	0.00066	0.00015	
4096	0.02054	0.02031	0.01950	0.01796	0.01560	0.01301	0.01013	0.00751	0.00522	0.00326	0.00183		
2048	0.03711	0.03780	0.03501	0.03125	0.02764	0.02306	0.01780	0.01320	0.00836	0.00456			
1024	0.06509	0.06497	0.06058	0.05454	0.04733	0.03880	0.02957	0.02030	0.01165				
512	0.10439	0.10340	0.09508	0.08472	0.07089	0.05708	0.04050	0.02659					
256	0.15444	0.15400	0.14081	0.12124	0.09962	0.07592	0.04998						
128	0.21308	0.21088	0.18985	0.15921	0.12485	0.08949							
64	0.27953	0.27236	0.23833	0.19313	0.14146								
32	0.34397	0.32874	0.27730	0.21236									
16	0.39787	0.36712	0.29047										
8	0.43281	0.38421											
4	0.43361												

(c) $\mathcal{P}^{\mathcal{C}}$ for Scene: [**Sponza**] Algorithm: [**PSSMLT**] Metric: [**FSIM**] True GT: [**PT @ 32768 spp**].

MLT	2	4	8	16	32	64	128	256	512	1024	2048	4096	8192
**GT (PT)**													
16384	0.00377	0.00303	0.00313	0.00274	0.00207	0.00176	0.00124	0.00063	0.00057	0.00021	0.00004	0.00004	-0.00012
8192	0.00901	0.00849	0.00791	0.00680	0.00536	0.00422	0.00275	0.00176	0.00099	0.00041	-0.00004	-0.00020	
4096	0.02075	0.01996	0.01761	0.01521	0.01239	0.00911	0.00623	0.00402	0.00228	0.00118	0.00020		
2048	0.03872	0.03623	0.03236	0.02744	0.02089	0.01570	0.00970	0.00585	0.00277	0.00061			
1024	0.06783	0.06309	0.05626	0.04677	0.03513	0.02477	0.01461	0.00760	0.00195				
512	0.10982	0.10095	0.08714	0.07076	0.05096	0.03407	0.01808	0.00584					
256	0.16297	0.14852	0.12582	0.09864	0.06690	0.04021	0.01479						
128	0.22951	0.20320	0.16551	0.12409	0.07515	0.03549							
64	0.29934	0.25865	0.20150	0.14058	0.06931								
32	0.36723	0.30416	0.22365	0.13597									
16	0.42419	0.33178	0.21885										
8	0.45845	0.33399											
4	0.46240												

(d) $\mathcal{P}^{\mathcal{C}}$ for Scene: [**Sponza**] Algorithm: [**MLT**] Metric: [**FSIM**] True GT: [**PT @ 32768 spp**].

M-MLT	2	4	8	16	32	64	128	256	512	1024	2048	4096	8192
**GT (PT)**													
16384	0.00353	0.00356	0.00305	0.00256	0.00217	0.00154	0.00114	0.00080	0.00068	0.00019	0.00022	0.00011	0.00002
8192	0.00929	0.00848	0.00754	0.00630	0.00504	0.00377	0.00268	0.00150	0.00100	0.00040	0.00002	-0.00016	
4096	0.02094	0.01986	0.01778	0.01497	0.01153	0.00843	0.00597	0.00328	0.00230	0.00084	0.00003		
2048	0.03816	0.03609	0.03184	0.02654	0.02049	0.01463	0.00963	0.00514	0.00247	0.00068			
1024	0.06728	0.06288	0.05441	0.04438	0.03363	0.02303	0.01421	0.00649	0.00170				
512	0.10706	0.09918	0.08483	0.06697	0.04845	0.03179	0.01617	0.00404					
256	0.16104	0.14577	0.12276	0.09345	0.06305	0.03632	0.01226						
128	0.22281	0.19943	0.16048	0.11577	0.07009	0.02815							
64	0.29219	0.25314	0.19240	0.12725	0.06115								
32	0.35446	0.29683	0.21249	0.12008									
16	0.40605	0.32110	0.20133										
8	0.43246	0.31589											
4	0.42420												

(e) $\mathcal{P}^{\mathcal{C}}$ for Scene: [**Sponza**] Algorithm: [**M-MLT**] Metric: [**FSIM**] True GT: [**PT @ 32768 spp**].

ERPT	2	4	8	16	32	64	128	256	512	1024	2048	4096	8192
**GT (PT)**													
16384	0.00100	0.00100	0.00114	0.00101	0.00092	0.00118	0.00094	0.00109	0.00085	0.00097	0.00122	0.00102	0.00120
8192	0.00229	0.00224	0.00240	0.00235	0.00230	0.00263	0.00241	0.00245	0.00237	0.00238	0.00241	0.00225	
4096	0.00523	0.00517	0.00583	0.00544	0.00545	0.00529	0.00530	0.00526	0.00550	0.00522	0.00510		
2048	0.00875	0.00842	0.00879	0.00855	0.00848	0.00844	0.00878	0.00834	0.00828	0.00821			
1024	0.01236	0.01169	0.01234	0.01231	0.01184	0.01182	0.01148	0.01250	0.01214				
512	0.01366	0.01332	0.01321	0.01329	0.01299	0.01263	0.01279	0.01239					
256	0.00935	0.00835	0.00840	0.00798	0.00740	0.00825	0.00852						
128	-0.01140	-0.01236	-0.01113	-0.01302	-0.01238	-0.01261							
64	-0.04742	-0.05175	-0.05099	-0.05287	-0.05176								
32	-0.10988	-0.11451	-0.11267	-0.11486									
16	-0.20323	-0.21077	-0.20669										
8	-0.31819	-0.32478											
4	-0.45150												

(f) $\mathcal{P}^{\mathcal{C}}$ for Scene: [**Sponza**] Algorithm: [**ERPT**] Metric: [**FSIM**] True GT: [**PT @ 32768 spp**].

M-ERPT	2	4	8	16	32	64	128	256	512	1024	2048	4096	8192
**GT (PT)**													
16384	0.00105	0.00104	0.00125	0.00090	0.00093	0.00107	0.00114	0.00108	0.00116	0.00092	0.00103	0.00103	0.00108
8192	0.00248	0.00248	0.00242	0.00250	0.00256	0.00225	0.00246	0.00245	0.00254	0.00231	0.00249	0.00242	
4096	0.00555	0.00548	0.00506	0.00550	0.00538	0.00537	0.00530	0.00557	0.00570	0.00508	0.00537		
2048	0.00898	0.00902	0.00854	0.00881	0.00826	0.00865	0.00899	0.00841	0.00840	0.00842			
1024	0.01249	0.01339	0.01202	0.01308	0.01269	0.01271	0.01210	0.01228	0.01185				
512	0.01446	0.01502	0.01421	0.01472	0.01390	0.01384	0.01385	0.01363					
256	0.00993	0.01004	0.00926	0.01046	0.00849	0.00847	0.00949						
128	-0.00953	-0.00925	-0.01184	-0.00911	-0.01107	-0.01245							
64	-0.04748	-0.04633	-0.05122	-0.04848	-0.04996								
32	-0.10915	-0.10787	-0.11369	-0.11104									
16	-0.20304	-0.20208	-0.20932										
8	-0.31987	-0.31636											
4	-0.45263												

(g) $\mathcal{P}^{\mathcal{C}}$ for Scene: [**Sponza**] Algorithm: [**M-ERPT**] Metric: [**FSIM**] True GT: [**PT @ 32768 spp**].

Table 45

11 FSIMc

11.1 Cornell Box

PT	2	4	8	16	32	64	128	256	512	1024	2048	4096	8192
**GT (PT)**													
16384	0.00118	0.00115	0.00099	0.00080	0.00069	0.00049	0.00040	0.00026	0.00012	0.00006	0.00003	-0.00001	-0.00005
8192	0.00379	0.00347	0.00307	0.00247	0.00207	0.00156	0.00109	0.00071	0.00040	0.00018	0.00005	-0.00006	
4096	0.00829	0.00742	0.00661	0.00537	0.00435	0.00331	0.00224	0.00144	0.00079	0.00033	-0.00002		
2048	0.01574	0.01428	0.01243	0.01019	0.00793	0.00597	0.00413	0.00244	0.00125	0.00028			
1024	0.02703	0.02412	0.02084	0.01674	0.01285	0.00923	0.00613	0.00328	0.00130				
512	0.04357	0.03847	0.03315	0.02607	0.01958	0.01344	0.00838	0.00389					
256	0.06480	0.05668	0.04829	0.03712	0.02691	0.01742	0.00914						
128	0.09369	0.08075	0.06644	0.04902	0.03302	0.01934							
64	0.12722	0.10785	0.08615	0.06062	0.03713								
32	0.16614	0.13566	0.10359	0.06599									
16	0.20434	0.16084	0.11389										
8	0.23745	0.17554											
4	0.25783												

(a) $\mathcal{P}^{\mathcal{C}}$ for Scene: [**Cornell Box**] Algorithm: [**PT**] Metric: [**FSIMc**] True GT: [**PT @ 32768 spp**].

BDPT	2	4	8	16	32	64	128	256	512	1024	2048	4096	8192
**GT (PT)**													
16384	0.00109	0.00096	0.00076	0.00057	0.00046	0.00029	0.00020	0.00012	0.00004	-0.00000	-0.00002	-0.00005	-0.00006
8192	0.00344	0.00298	0.00238	0.00181	0.00133	0.00090	0.00059	0.00031	0.00011	-0.00001	-0.00011	-0.00016	
4096	0.00747	0.00643	0.00511	0.00399	0.00287	0.00193	0.00118	0.00059	0.00018	-0.00014	-0.00032		
2048	0.01407	0.01197	0.00945	0.00720	0.00509	0.00322	0.00193	0.00082	-0.00000	-0.00052			
1024	0.02357	0.01989	0.01532	0.01149	0.00781	0.00489	0.00248	0.00053	-0.00065				
512	0.03775	0.03142	0.02389	0.01727	0.01119	0.00626	0.00238	-0.00065					
256	0.05532	0.04473	0.03287	0.02261	0.01349	0.00599	0.00020						
128	0.07669	0.06024	0.04208	0.02667	0.01273	0.00187							
64	0.10117	0.07651	0.04896	0.02659	0.00735								
32	0.12555	0.08763	0.04970	0.01827									
16	0.14406	0.09102	0.03825										
8	0.15136	0.07764											
4	0.13764												

(b) $\mathcal{P}^{\mathcal{C}}$ for Scene: [**Cornell Box**] Algorithm: [**BDPT**] Metric: [**FSIMc**] True GT: [**PT @ 32768 spp**].

PSSMLT	2	4	8	16	32	64	128	256	512	1024	2048	4096	8192
**GT (PT)**													
16384	0.00101	0.00094	0.00083	0.00066	0.00049	0.00033	0.00023	0.00014	0.00008	0.00003	0.00000	-0.00001	-0.00002
8192	0.00318	0.00273	0.00234	0.00184	0.00135	0.00092	0.00060	0.00038	0.00020	0.00006	-0.00002	-0.00007	
4096	0.00694	0.00604	0.00503	0.00400	0.00294	0.00201	0.00124	0.00077	0.00034	0.00006	-0.00011		
2048	0.01310	0.01126	0.00918	0.00723	0.00526	0.00346	0.00206	0.00111	0.00033	-0.00018			
1024	0.02194	0.01884	0.01516	0.01151	0.00812	0.00500	0.00262	0.00111	-0.00019				
512	0.03516	0.02956	0.02343	0.01731	0.01162	0.00656	0.00275	0.00024					
256	0.05175	0.04210	0.03241	0.02278	0.01394	0.00649	0.00075						
128	0.07178	0.05679	0.04175	0.02706	0.01389	0.00308							
64	0.09383	0.07155	0.04888	0.02752	0.00842								
32	0.11743	0.08303	0.04997	0.02028									
16	0.13297	0.08510	0.03913										
8	0.13783	0.07116											
4	0.11937												

(c) $\mathcal{P}^{\mathcal{C}}$ for Scene: [**Cornell Box**] Algorithm: [**PSSMLT**] Metric: [**FSIMc**] True GT: [**PT @ 32768 spp**].

MLT	2	4	8	16	32	64	128	256	512	1024	2048	4096	8192
**GT (PT)**													
16384	0.00121	0.00115	0.00106	0.00092	0.00080	0.00064	0.00052	0.00036	0.00022	0.00013	0.00007	0.00003	0.00000
8192	0.00380	0.00353	0.00330	0.00290	0.00253	0.00206	0.00149	0.00103	0.00069	0.00040	0.00020	0.00007	
4096	0.00835	0.00791	0.00730	0.00639	0.00540	0.00433	0.00319	0.00220	0.00141	0.00084	0.00037		
2048	0.01610	0.01491	0.01369	0.01197	0.00997	0.00790	0.00575	0.00389	0.00246	0.00137			
1024	0.02773	0.02566	0.02324	0.01988	0.01631	0.01265	0.00899	0.00582	0.00335				
512	0.04513	0.04144	0.03722	0.03139	0.02515	0.01918	0.01294	0.00776					
256	0.06839	0.06250	0.05515	0.04569	0.03568	0.02609	0.01651						
128	0.10038	0.09046	0.07862	0.06371	0.04767	0.03255							
64	0.13798	0.12295	0.10463	0.08152	0.05796								
32	0.18655	0.16200	0.13380	0.09915									
16	0.23799	0.19974	0.15807										
8	0.28879	0.23435											
4	0.33515												

(d) $\mathcal{P}^{\mathcal{C}}$ for Scene: [**Cornell Box**] Algorithm: [**MLT**] Metric: [**FSIMc**] True GT: [**PT @ 32768 spp**].

M-MLT	2	4	8	16	32	64	128	256	512	1024	2048	4096	8192
**GT (PT)**													
16384	0.00120	0.00116	0.00102	0.00090	0.00077	0.00061	0.00050	0.00032	0.00023	0.00013	0.00006	0.00002	0.00001
8192	0.00374	0.00359	0.00329	0.00283	0.00244	0.00200	0.00149	0.00103	0.00064	0.00038	0.00018	0.00009	
4096	0.00838	0.00774	0.00709	0.00614	0.00510	0.00409	0.00303	0.00210	0.00135	0.00076	0.00034		
2048	0.01581	0.01475	0.01328	0.01140	0.00959	0.00761	0.00556	0.00375	0.00227	0.00113			
1024	0.02743	0.02541	0.02257	0.01908	0.01558	0.01204	0.00846	0.00541	0.00293				
512	0.04457	0.04092	0.03614	0.03022	0.02463	0.01848	0.01233	0.00745					
256	0.06723	0.06147	0.05351	0.04408	0.03456	0.02467	0.01519						
128	0.09790	0.08892	0.07519	0.06013	0.04530	0.02988							
64	0.13552	0.12115	0.10007	0.07691	0.05492								
32	0.18014	0.15940	0.12682	0.09250									
16	0.22836	0.19585	0.14920										
8	0.27411	0.22763											
4	0.31577												

(e) $\mathcal{P}^{\mathcal{C}}$ for Scene: [**Cornell Box**] Algorithm: [**M-MLT**] Metric: [**FSIMc**] True GT: [**PT @ 32768 spp**].

ERPT	2	4	8	16	32	64	128	256	512	1024	2048	4096	8192
**GT (PT)**													
16384	0.00043	0.00042	0.00045	0.00037	0.00048	0.00049	0.00044	0.00044	0.00047	0.00046	0.00045	0.00042	0.00044
8192	0.00133	0.00131	0.00136	0.00132	0.00141	0.00139	0.00139	0.00137	0.00142	0.00134	0.00135	0.00140	
4096	0.00289	0.00275	0.00298	0.00291	0.00291	0.00290	0.00293	0.00298	0.00307	0.00284	0.00291		
2048	0.00519	0.00501	0.00542	0.00530	0.00523	0.00523	0.00535	0.00535	0.00560	0.00512			
1024	0.00813	0.00795	0.00825	0.00820	0.00805	0.00801	0.00821	0.00811	0.00837				
512	0.01171	0.01122	0.01196	0.01179	0.01140	0.01155	0.01208	0.01170					
256	0.01423	0.01376	0.01461	0.01450	0.01397	0.01417	0.01483						
128	0.01476	0.01388	0.01483	0.01497	0.01436	0.01429							
64	0.01001	0.00888	0.01046	0.01026	0.01017								
32	-0.00406	-0.00551	-0.00384	-0.00377									
16	-0.03345	-0.03516	-0.03304										
8	-0.08355	-0.08658											
4	-0.15980												

(f) $\mathcal{P}^{\mathcal{C}}$ for Scene: [**Cornell Box**] Algorithm: [**ERPT**] Metric: [**FSIMc**] True GT: [**PT @ 32768 spp**].

M-ERPT	2	4	8	16	32	64	128	256	512	1024	2048	4096	8192
**GT (PT)**													
16384	0.00044	0.00039	0.00046	0.00044	0.00042	0.00041	0.00046	0.00046	0.00040	0.00042	0.00052	0.00044	0.00045
8192	0.00140	0.00129	0.00139	0.00140	0.00132	0.00141	0.00136	0.00142	0.00138	0.00143	0.00139	0.00142	
4096	0.00292	0.00288	0.00298	0.00305	0.00285	0.00301	0.00295	0.00299	0.00298	0.00302	0.00302		
2048	0.00539	0.00518	0.00543	0.00542	0.00514	0.00549	0.00544	0.00535	0.00541	0.00551			
1024	0.00835	0.00803	0.00840	0.00844	0.00799	0.00845	0.00836	0.00824	0.00827				
512	0.01183	0.01170	0.01216	0.01235	0.01163	0.01227	0.01186	0.01187					
256	0.01472	0.01428	0.01497	0.01551	0.01409	0.01499	0.01460						
128	0.01516	0.01453	0.01556	0.01585	0.01431	0.01572							
64	0.01046	0.00998	0.01108	0.01186	0.00997								
32	-0.00347	-0.00378	-0.00267	-0.00215									
16	-0.03200	-0.03312	-0.03066										
8	-0.08275	-0.08371											
4	-0.15851												

(g) $\mathcal{P}^{\mathcal{C}}$ for Scene: [**Cornell Box**] Algorithm: [**M-ERPT**] Metric: [**FSIMc**] True GT: [**PT @ 32768 spp**].

Table 46

11.2 Torus

PT	2	4	8	16	32	64	128	256	512	1024	2048	4096	8192	16384	32768	65536	131072
GT (BDPT)																	
262144	0.00663	0.00550	0.00389	0.00296	0.00215	0.00161	0.00131	0.00171	0.00192	0.00219	0.00252	0.00265	0.00254	0.00219	0.00154	0.00062	-0.00012
131072	0.02016	0.01526	0.01137	0.00784	0.00617	0.00426	0.00359	0.00337	0.00532	0.00686	0.00837	0.00812	0.00725	0.00502	0.00306	0.00082	
65536	0.03638	0.02649	0.01840	0.01281	0.00870	0.00650	0.00495	0.00553	0.00877	0.01238	0.01333	0.01324	0.01026	0.00712	0.00283		
32768	0.05853	0.04182	0.02818	0.01767	0.01196	0.00878	0.00707	0.00832	0.01369	0.01924	0.02052	0.01919	0.01409	0.00672			
16384	0.08023	0.05528	0.03464	0.01911	0.01210	0.00728	0.00602	0.01007	0.01849	0.02458	0.02722	0.02248	0.01339				
8192	0.10138	0.06595	0.03685	0.01633	0.00662	0.00285	0.00576	0.01233	0.02351	0.03168	0.03083	0.02037					
4096	0.11407	0.06924	0.03257	0.00995	0.00103	-0.00403	0.00467	0.01568	0.02923	0.03581	0.02854						
2048	0.11435	0.06192	0.01984	-0.00468	-0.01173	-0.01077	-0.00145	0.01447	0.03171	0.03228							
1024	0.11075	0.05242	0.00446	-0.01808	-0.02386	-0.02061	-0.00594	0.00882	0.02178								
512	0.09712	0.04041	-0.00436	-0.02375	-0.02701	-0.02207	-0.00875	0.00497									
256	0.08659	0.03679	-0.00581	-0.01688	-0.02128	-0.01993	-0.01045										
128	0.09449	0.04735	0.01279	0.00048	-0.01019	-0.01261											
64	0.10859	0.06299	0.02890	0.01193	-0.00311												
32	0.13519	0.08908	0.05110	0.02623													
16	0.18004	0.11861	0.07819														
8	0.21481	0.13859															
4	0.24866																

(a) $\mathcal{P}^{\mathcal{C}}$ for Scene: [**Torus**] Algorithm: [**PT**] Metric: [**FSIMc**] True GT: [**BDPT @ 524288 spp**].

BDPT	2	4	8	16	32	64	128	256	512	1024	2048	4096	8192	16384	32768	65536	131072
GT (BDPT)																	
262144	0.00689	0.00493	0.00385	0.00281	0.00221	0.00180	0.00140	0.00119	0.00200	0.00225	0.00261	0.00303	0.00255	0.00199	0.00141	0.00053	-0.00010
131072	0.02035	0.01499	0.01137	0.00814	0.00590	0.00412	0.00333	0.00343	0.00547	0.00655	0.00759	0.00817	0.00730	0.00551	0.00344	0.00095	
65536	0.03711	0.02718	0.01921	0.01313	0.00867	0.00607	0.00532	0.00584	0.00859	0.01192	0.01358	0.01377	0.01137	0.00747	0.00345		
32768	0.05934	0.04235	0.02923	0.01866	0.01194	0.00835	0.00745	0.00786	0.01418	0.01797	0.01989	0.01933	0.01426	0.00747			
16384	0.08263	0.05776	0.03593	0.02129	0.01147	0.00674	0.00615	0.01056	0.01852	0.02331	0.02512	0.02241	0.01462				
8192	0.10625	0.07034	0.03925	0.02033	0.00767	0.00374	0.00390	0.01285	0.02489	0.03135	0.02945	0.02170					
4096	0.12226	0.07630	0.03863	0.01548	0.00057	-0.00093	0.00229	0.01403	0.02897	0.03264	0.02720						
2048	0.12499	0.06937	0.02482	0.00177	-0.01111	-0.01002	0.00132	0.01671	0.03015	0.02960							
1024	0.12170	0.06104	0.01362	-0.01147	-0.02340	-0.01836	-0.00748	0.01051	0.02226								
512	0.10926	0.04780	0.00271	-0.01965	-0.02777	-0.01869	-0.00755	0.00747									
256	0.09316	0.03918	0.00134	-0.01362	-0.02060	-0.01987	-0.00714										
128	0.09940	0.05076	0.01553	0.00210	-0.00535	-0.00923											
64	0.11353	0.06820	0.03192	0.01326	0.00044												
32	0.14033	0.09094	0.05048	0.02836													
16	0.18807	0.12710	0.07204														
8	0.22785	0.15081															
4	0.26152																

(b) $\mathcal{P}^{\mathcal{C}}$ for Scene: [**Torus**] Algorithm: [**BDPT**] Metric: [**FSIMc**] True GT: [**BDPT @ 524288 spp**].

PSSMLT	2	4	8	16	32	64	128	256	512	1024	2048	4096	8192	16384
GT (BDPT)														
262144	0.00126	0.00182	0.00164	0.00172	0.00181	0.00219	0.00224	0.00251	0.00238	0.00259	0.00266	0.00264	0.00238	0.00201
131072	0.00351	0.00466	0.00507	0.00492	0.00530	0.00609	0.00659	0.00723	0.00745	0.00752	0.00741	0.00690	0.00614	0.00582
65536	0.00587	0.00797	0.00785	0.00749	0.00872	0.00970	0.01084	0.01211	0.01233	0.01219	0.01180	0.01073	0.00907	0.00779
32768	0.01049	0.01311	0.01204	0.01137	0.01347	0.01610	0.01755	0.01822	0.01740	0.01666	0.01479	0.01182	0.00878	0.00673
16384	0.01815	0.01806	0.01550	0.01361	0.01712	0.01885	0.02092	0.02029	0.01888	0.01685	0.01277	0.00764	0.00241	
8192	0.02885	0.02580	0.01978	0.01552	0.01940	0.02210	0.02249	0.02111	0.01490	0.01044	0.00253	-0.00644		
4096	0.03565	0.02638	0.01960	0.01521	0.02039	0.02221	0.02151	0.01611	0.00641	-0.00113	-0.01352			
2048	0.03319	0.01730	0.00756	0.00537	0.01386	0.01635	0.01468	0.00361	-0.01143	-0.02590				
1024	0.02732	0.00454	-0.00601	-0.00790	0.00024	0.00427	-0.00318	-0.01784	-0.03772					
512	0.00861	-0.00766	-0.01516	-0.01417	-0.00535	-0.00873	-0.01870	-0.03586						
256	-0.00143	-0.02037	-0.02672	-0.02543	-0.01980	-0.02397	-0.03467							
128	-0.01128	-0.02062	-0.02385	-0.02196	-0.01934	-0.02538								
64	-0.01536	-0.01818	-0.02073	-0.02192	-0.02393									
32	-0.00725	-0.00872	-0.01666	-0.01815										
16	0.00950	0.00231	-0.01384											
8	0.02255	0.00706												
4	0.02370													

(c) $\mathcal{P}^{\mathcal{C}}$ for Scene: [**Torus**] Algorithm: [**PSSMLT**] Metric: [**FSIMc**] True GT: [**BDPT @ 524288 spp**].

MLT	2	4	8	16	32	64	128	256	512	1024	2048	4096	8192	16384
GT (BDPT)														
262144	-0.00153	-0.00052	0.00094	0.00148	0.00195	0.00194	0.00144	0.00191	0.00168	0.00159	0.00187	0.00153	0.00169	0.00182
131072	-0.00485	-0.00198	0.00247	0.00310	0.00500	0.00511	0.00434	0.00491	0.00399	0.00441	0.00421	0.00416	0.00400	0.00398
65536	-0.00842	-0.00320	0.00468	0.00655	0.00811	0.00756	0.00669	0.00693	0.00518	0.00555	0.00516	0.00477	0.00472	0.00408
32768	-0.00820	-0.00215	0.00819	0.01035	0.01144	0.00966	0.00752	0.00648	0.00382	0.00431	0.00296	0.00253	0.00226	0.00109
16384	-0.00682	0.00159	0.01304	0.01419	0.01349	0.00933	0.00530	0.00348	-0.00106	-0.00112	-0.00360	-0.00483	-0.00559	
8192	-0.00036	0.01076	0.02050	0.01700	0.01288	0.00587	-0.00241	-0.00753	-0.01518	-0.01667	-0.01902	-0.02191		
4096	0.00182	0.01294	0.02189	0.01226	0.00396	-0.00501	-0.01757	-0.02586	-0.03536	-0.03719	-0.04127			
2048	0.00279	0.01112	0.01370	-0.00294	-0.01538	-0.02733	-0.04142	-0.05014	-0.06248	-0.06513				
1024	-0.00307	0.00552	-0.00133	-0.02614	-0.04065	-0.05446	-0.07141	-0.08141	-0.09437					
512	-0.01487	-0.00789	-0.01901	-0.04841	-0.06069	-0.07514	-0.09300	-0.10394						
256	-0.02483	-0.02280	-0.03321	-0.06286	-0.07507	-0.08554	-0.10352							
128	-0.03013	-0.03150	-0.04128	-0.06785	-0.07354	-0.08188								
64	-0.02979	-0.03697	-0.04563	-0.07344	-0.07042									
32	-0.02781	-0.03360	-0.03556	-0.06147										
16	-0.01759	-0.02355	-0.01966											
8	-0.02206	-0.02575												
4	-0.03605													

(d) $\mathcal{P}^{\mathcal{C}}$ for Scene: [**Torus**] Algorithm: [**MLT**] Metric: [**FSIMc**] True GT: [**BDPT @ 524288 spp**].

M-MLT	2	4	8	16	32	64	128	256	512	1024	2048	4096	8192	16384
GT (BDPT)														
262144	-0.00110	0.00026	0.00142	0.00145	0.00204	0.00184	0.00150	0.00164	0.00170	0.00145	0.00161	0.00151	0.00161	0.00152
131072	-0.00448	0.00089	0.00464	0.00400	0.00614	0.00496	0.00486	0.00539	0.00425	0.00420	0.00432	0.00423	0.00408	0.00388
65536	-0.00708	0.00113	0.00868	0.00621	0.00966	0.00704	0.00767	0.00750	0.00543	0.00566	0.00568	0.00519	0.00520	0.00450
32768	-0.00825	0.00397	0.01484	0.01003	0.01432	0.00854	0.00952	0.00769	0.00341	0.00391	0.00309	0.00270	0.00219	0.00113
16384	-0.00683	0.00931	0.02168	0.01407	0.01758	0.00669	0.00746	0.00402	-0.00273	-0.00436	-0.00532	-0.00576	-0.00728	
8192	-0.00178	0.01796	0.02876	0.01411	0.01739	-0.00132	-0.00176	-0.00955	-0.02001	-0.01963	-0.02334	-0.02434		
4096	-0.00272	0.02187	0.02868	0.00894	0.01107	-0.01831	-0.01832	-0.02892	-0.04122	-0.04346	-0.04693			
2048	-0.00061	0.02002	0.01602	-0.00815	-0.00918	-0.04455	-0.04597	-0.05759	-0.07349	-0.07534				
1024	-0.00164	0.01040	-0.00476	-0.03313	-0.03292	-0.08068	-0.08183	-0.09367	-0.11004					
512	-0.01337	-0.00916	-0.03102	-0.05961	-0.05693	-0.10432	-0.10536	-0.11856						
256	-0.01434	-0.02059	-0.04770	-0.07690	-0.06921	-0.11692	-0.11768							
128	-0.01684	-0.03264	-0.06311	-0.08086	-0.07081	-0.11913								
64	-0.01839	-0.04119	-0.06747	-0.08258	-0.07156									
32	-0.02124	-0.03667	-0.05991	-0.07609										
16	-0.02014	-0.01820	-0.04487											
8	-0.03908	-0.00753												
4	-0.06841													

(e) $\mathcal{P}^{\mathcal{C}}$ for Scene: [**Torus**] Algorithm: [**M-MLT**] Metric: [**FSIMc**] True GT: [**BDPT @ 524288 spp**].

ERPT	2	4	8	16	32	64	128	256	512	1024	2048	4096	8192	16384	32768	65536	131072
GT (BDPT)																	
262144	0.00205	0.00227	0.00232	0.00233	0.00175	0.00227	0.00221	0.00222	0.00264	0.00250	0.00263	0.00270	0.00265	0.00254	0.00253	0.00247	0.00251
131072	0.00462	0.00646	0.00559	0.00596	0.00592	0.00657	0.00652	0.00694	0.00691	0.00720	0.00727	0.00692	0.00719	0.00668	0.00696	0.00690	
65536	0.00576	0.00923	0.00837	0.00887	0.00892	0.00967	0.00990	0.01107	0.01084	0.01089	0.01082	0.01030	0.01059	0.01029	0.01035		
32768	0.00561	0.01117	0.00992	0.00989	0.01051	0.01214	0.01176	0.01360	0.01285	0.01280	0.01225	0.01154	0.01171	0.01220			
16384	0.00167	0.00919	0.00683	0.00680	0.00777	0.00996	0.00896	0.01141	0.01015	0.00990	0.00922	0.00730	0.00743				
8192	-0.01097	-0.00172	-0.00422	-0.00422	-0.00252	-0.00104	-0.00228	0.00128	-0.00137	-0.00236	-0.00321	-0.00544					
4096	-0.02733	-0.01716	-0.02049	-0.02180	-0.01945	-0.01791	-0.02043	-0.01650	-0.02071	-0.02094	-0.02566						
2048	-0.05581	-0.04786	-0.04950	-0.05349	-0.04978	-0.04576	-0.05156	-0.04784	-0.05261	-0.05582							
1024	-0.08376	-0.08228	-0.08254	-0.08756	-0.08557	-0.07957	-0.08536	-0.08075	-0.08894								
512	-0.09913	-0.10202	-0.10337	-0.11115	-0.11013	-0.10569	-0.11224	-0.10874									
256	-0.10413	-0.10996	-0.11259	-0.11905	-0.11909	-0.11471	-0.12550										
128	-0.09161	-0.09865	-0.10285	-0.11508	-0.11795	-0.11267											
64	-0.08300	-0.08880	-0.09840	-0.10744	-0.11210												
32	-0.07165	-0.08268	-0.08826	-0.09734													
16	-0.06733	-0.08049	-0.08930														
8	-0.08192	-0.09387															
4	-0.13444																

(f) $\mathcal{P}^{\mathcal{C}}$ for Scene: [**Torus**] Algorithm: [**ERPT**] Metric: [**FSIMc**] True GT: [**BDPT @ 524288 spp**].

M-ERPT	2	4	8	16	32	64	128	256	512	1024	2048	4096	8192	16384	32768	65536	131072
GT (BDPT)																	
262144	0.00192	0.00147	0.00200	0.00212	0.00248	0.00225	0.00232	0.00227	0.00250	0.00250	0.00249	0.00247	0.00234	0.00249	0.00245	0.00257	0.00249
131072	0.00511	0.00428	0.00571	0.00643	0.00691	0.00701	0.00694	0.00693	0.00689	0.00722	0.00700	0.00743	0.00740	0.00723	0.00737	0.00718	
65536	0.00799	0.00651	0.00759	0.00910	0.00998	0.01020	0.01089	0.01024	0.01040	0.01070	0.01114	0.01140	0.01047	0.01076	0.01051		
32768	0.01024	0.00810	0.00982	0.01148	0.01280	0.01063	0.01184	0.01179	0.01144	0.01212	0.01141	0.01261	0.01210	0.01189			
16384	0.00882	0.00591	0.00610	0.00917	0.01040	0.00753	0.00885	0.00825	0.00793	0.00808	0.00764	0.00857	0.00744				
8192	0.00208	-0.00319	-0.00359	-0.00134	0.00065	-0.00439	-0.00507	-0.00522	-0.00604	-0.00538	-0.00694	-0.00464					
4096	-0.01213	-0.01985	-0.02156	-0.01879	-0.01868	-0.02634	-0.02388	-0.02703	-0.02886	-0.02758	-0.02834						
2048	-0.03490	-0.04498	-0.05021	-0.04798	-0.04877	-0.05850	-0.05941	-0.06251	-0.06477	-0.06374							
1024	-0.06542	-0.07416	-0.07855	-0.08111	-0.08408	-0.09531	-0.09870	-0.09991	-0.10321								
512	-0.08068	-0.09347	-0.10154	-0.10603	-0.10993	-0.12129	-0.12425	-0.12986									
256	-0.08907	-0.09817	-0.10967	-0.11487	-0.11797	-0.13035	-0.13582										
128	-0.08019	-0.09070	-0.10337	-0.11164	-0.11344	-0.12584											
64	-0.07345	-0.08406	-0.09608	-0.10642	-0.10423												
32	-0.06632	-0.07832	-0.08768	-0.09759													
16	-0.06324	-0.07578	-0.08192														
8	-0.07577	-0.08443															
4	-0.12427																

(g) $\mathcal{P}^{\mathcal{C}}$ for Scene: [**Torus**] Algorithm: [**M-ERPT**] Metric: [**FSIMc**] True GT: [**BDPT @ 524288 spp**].

Table 47

11.3 Veach Bidir

PT	2	4	8	16	32	64	128	256	512	1024	2048	4096	8192	16384	32768	65536	131072
GT (BDPT)																	
262144	0.00018	0.00019	0.00015	0.00017	0.00018	0.00016	0.00015	0.00011	0.00014	0.00011	0.00006	0.00008	0.00001	0.00005	0.00002	0.00004	0.00003
131072	0.00069	0.00057	0.00046	0.00044	0.00042	0.00040	0.00033	0.00028	0.00029	0.00021	0.00014	0.00017	0.00011	0.00010	0.00007	0.00008	
65536	0.00137	0.00122	0.00112	0.00107	0.00099	0.00090	0.00090	0.00076	0.00070	0.00059	0.00061	0.00055	0.00047	0.00040	0.00030		
32768	0.00279	0.00255	0.00241	0.00221	0.00213	0.00197	0.00180	0.00163	0.00149	0.00136	0.00109	0.00105	0.00090	0.00082			
16384	0.00398	0.00362	0.00333	0.00318	0.00290	0.00267	0.00263	0.00229	0.00206	0.00191	0.00160	0.00155	0.00126				
8192	0.00744	0.00686	0.00626	0.00595	0.00548	0.00515	0.00480	0.00431	0.00397	0.00345	0.00305	0.00259					
4096	0.01231	0.01132	0.01024	0.00976	0.00891	0.00796	0.00758	0.00684	0.00604	0.00556	0.00479						
2048	0.02064	0.01901	0.01786	0.01622	0.01497	0.01364	0.01273	0.01148	0.01011	0.00912							
1024	0.03860	0.03562	0.03292	0.03051	0.02763	0.02507	0.02348	0.02089	0.01890								
512	0.06426	0.05954	0.05499	0.04996	0.04541	0.04114	0.03796	0.03330									
256	0.10448	0.09660	0.08817	0.08013	0.07266	0.06495	0.05954										
128	0.15325	0.14199	0.12882	0.11636	0.10427	0.09261											
64	0.21439	0.19790	0.17974	0.16134	0.14204												
32	0.29832	0.27417	0.24627	0.21824													
16	0.38689	0.35312	0.31462														
8	0.49520	0.44705															
4	0.60084																

(a) $\mathcal{P}^{\mathcal{C}}$ for Scene: [**Veach Bidir**] Algorithm: [**PT**] Metric: [**FSIMc**] True GT: [**BDPT @ 524288 spp**].

BDPT	2	4	8	16	32	64	128	256	512	1024	2048	4096	8192	16384	32768	65536	131072
GT (BDPT)																	
262144	0.00015	0.00013	0.00007	0.00010	0.00007	0.00003	0.00006	0.00005	0.00001	0.00002	-0.00003	-0.00002	-0.00000	-0.00000	-0.00000	-0.00001	-0.00001
131072	0.00041	0.00034	0.00024	0.00020	0.00014	0.00011	0.00007	0.00005	0.00003	-0.00001	-0.00002	-0.00002	-0.00000	-0.00001	-0.00001	-0.00003	
65536	0.00093	0.00082	0.00070	0.00063	0.00051	0.00037	0.00032	0.00023	0.00015	0.00006	0.00001	-0.00003	-0.00000	-0.00004	-0.00005		
32768	0.00191	0.00166	0.00143	0.00122	0.00104	0.00077	0.00058	0.00043	0.00028	0.00010	0.00002	-0.00009	-0.00009	-0.00010			
16384	0.00281	0.00246	0.00216	0.00177	0.00155	0.00126	0.00098	0.00064	0.00043	0.00014	-0.00002	-0.00020	-0.00021				
8192	0.00506	0.00449	0.00395	0.00322	0.00262	0.00213	0.00156	0.00112	0.00050	0.00017	-0.00017	-0.00034					
4096	0.00817	0.00716	0.00607	0.00505	0.00402	0.00320	0.00239	0.00172	0.00077	0.00018	-0.00023						
2048	0.01398	0.01208	0.01025	0.00841	0.00681	0.00537	0.00396	0.00258	0.00148	0.00020							
1024	0.02568	0.02210	0.01870	0.01516	0.01217	0.00931	0.00660	0.00413	0.00166								
512	0.04241	0.03567	0.02967	0.02369	0.01811	0.01316	0.00864	0.00456									
256	0.06745	0.05643	0.04601	0.03500	0.02595	0.01732	0.00993										
128	0.09794	0.07977	0.06324	0.04615	0.03187	0.01838											
64	0.13341	0.10754	0.08182	0.05596	0.03520												
32	0.17971	0.13992	0.10169	0.06273													
16	0.22223	0.16436	0.11067														
8	0.26300	0.18192															
4	0.28867																

(b) $\mathcal{P}^{\mathcal{C}}$ for Scene: [**Veach Bidir**] Algorithm: [**BDPT**] Metric: [**FSIMc**] True GT: [**BDPT @ 524288 spp**].

PSSMLT	2	4	8	16	32	64	128	256	512	1024	2048	4096	8192	16384
GT (BDPT)														
262144	0.00015	0.00011	0.00010	0.00010	0.00008	0.00009	0.00008	0.00003	0.00003	0.00003	0.00001	0.00002	0.00001	0.00001
131072	0.00044	0.00032	0.00027	0.00022	0.00020	0.00016	0.00014	0.00008	0.00006	0.00002	0.00003	-0.00000	-0.00001	-0.00001
65536	0.00096	0.00077	0.00066	0.00057	0.00050	0.00040	0.00034	0.00025	0.00022	0.00014	0.00010	0.00007	0.00003	-0.00001
32768	0.00184	0.00168	0.00137	0.00126	0.00103	0.00084	0.00071	0.00052	0.00040	0.00030	0.00019	0.00005	0.00001	-0.00001
16384	0.00265	0.00235	0.00197	0.00176	0.00151	0.00130	0.00106	0.00075	0.00055	0.00037	0.00016	0.00004	-0.00007	
8192	0.00508	0.00452	0.00373	0.00335	0.00276	0.00222	0.00177	0.00139	0.00105	0.00061	0.00028	0.00011		
4096	0.00832	0.00711	0.00614	0.00522	0.00431	0.00347	0.00276	0.00204	0.00138	0.00077	0.00031			
2048	0.01386	0.01186	0.01016	0.00869	0.00708	0.00571	0.00458	0.00338	0.00228	0.00123				
1024	0.02575	0.02215	0.01883	0.01595	0.01280	0.01018	0.00768	0.00531	0.00335					
512	0.04250	0.03609	0.02975	0.02447	0.01903	0.01456	0.01036	0.00650						
256	0.06822	0.05749	0.04663	0.03726	0.02771	0.01986	0.01301							
128	0.09850	0.08131	0.06409	0.04935	0.03456	0.02262								
64	0.13478	0.10964	0.08431	0.06179	0.03976									
32	0.18073	0.14299	0.10536	0.07024										
16	0.22467	0.17040	0.11721											
8	0.26697	0.19219												
4	0.29491													

(c) $\mathcal{P}^{\mathcal{C}}$ for Scene: [**Veach Bidir**] Algorithm: [**PSSMLT**] Metric: [**FSIMc**] True GT: [**BDPT @ 524288 spp**].

MLT	2	4	8	16	32	64	128	256	512	1024	2048	4096	8192	16384
GT (BDPT)														
262144	0.00016	0.00018	0.00018	0.00012	0.00012	0.00011	0.00009	0.00006	0.00005	0.00005	0.00004	0.00002	0.00001	0.00002
131072	0.00053	0.00045	0.00044	0.00034	0.00031	0.00024	0.00018	0.00014	0.00012	0.00011	0.00007	0.00003	0.00001	0.00000
65536	0.00107	0.00097	0.00093	0.00081	0.00072	0.00064	0.00058	0.00048	0.00040	0.00029	0.00022	0.00012	0.00009	0.00006
32768	0.00230	0.00213	0.00195	0.00169	0.00158	0.00132	0.00111	0.00090	0.00076	0.00059	0.00042	0.00023	0.00014	0.00005
16384	0.00334	0.00303	0.00269	0.00244	0.00220	0.00192	0.00170	0.00144	0.00120	0.00093	0.00059	0.00038	0.00025	
8192	0.00622	0.00562	0.00502	0.00433	0.00381	0.00343	0.00302	0.00253	0.00209	0.00164	0.00108	0.00059		
4096	0.01050	0.00921	0.00818	0.00709	0.00610	0.00519	0.00448	0.00372	0.00301	0.00216	0.00144			
2048	0.01749	0.01556	0.01370	0.01200	0.01038	0.00888	0.00752	0.00623	0.00493	0.00343				
1024	0.03235	0.02904	0.02565	0.02239	0.01929	0.01629	0.01354	0.01112	0.00860					
512	0.05390	0.04814	0.04218	0.03653	0.03093	0.02539	0.02041	0.01614						
256	0.08835	0.07887	0.06882	0.05855	0.04901	0.03904	0.03054							
128	0.13099	0.11635	0.10069	0.08523	0.06954	0.05372								
64	0.18400	0.16406	0.14074	0.11636	0.09245									
32	0.25469	0.22615	0.19191	0.15612										
16	0.32661	0.28985	0.24213											
8	0.41296	0.36074												
4	0.49386													

(d) $\mathcal{P}^{\mathcal{C}}$ for Scene: [**Veach Bidir**] Algorithm: [**MLT**] Metric: [**FSIMc**] True GT: [**BDPT @ 524288 spp**].

M-MLT	2	4	8	16	32	64	128	256	512	1024	2048	4096	8192	16384
GT (BDPT)														
262144	0.00017	0.00014	0.00013	0.00011	0.00010	0.00008	0.00008	0.00007	0.00007	0.00004	0.00002	0.00002	0.00002	0.00000
131072	0.00057	0.00048	0.00041	0.00033	0.00030	0.00023	0.00014	0.00011	0.00010	0.00008	0.00003	0.00003	0.00000	-0.00000
65536	0.00116	0.00093	0.00085	0.00077	0.00073	0.00061	0.00051	0.00049	0.00041	0.00031	0.00018	0.00013	0.00008	0.00003
32768	0.00234	0.00204	0.00187	0.00161	0.00149	0.00128	0.00103	0.00091	0.00074	0.00050	0.00035	0.00019	0.00009	-0.00001
16384	0.00327	0.00298	0.00263	0.00240	0.00212	0.00188	0.00161	0.00141	0.00116	0.00085	0.00059	0.00041	0.00024	
8192	0.00611	0.00551	0.00491	0.00429	0.00369	0.00341	0.00282	0.00243	0.00189	0.00146	0.00093	0.00050		
4096	0.01030	0.00903	0.00796	0.00686	0.00614	0.00522	0.00433	0.00363	0.00284	0.00215	0.00127			
2048	0.01731	0.01518	0.01351	0.01162	0.01032	0.00890	0.00730	0.00618	0.00481	0.00341				
1024	0.03180	0.02825	0.02505	0.02171	0.01869	0.01595	0.01304	0.01088	0.00821					
512	0.05327	0.04722	0.04140	0.03512	0.03016	0.02501	0.01963	0.01550						
256	0.08708	0.07738	0.06688	0.05629	0.04748	0.03833	0.02938							
128	0.12860	0.11288	0.09865	0.08168	0.06682	0.05165								
64	0.18071	0.15849	0.13675	0.11112	0.08921									
32	0.25002	0.21909	0.18747	0.14895										
16	0.32294	0.28135	0.23586											
8	0.40756	0.34973												
4	0.48688													

(e) $\mathcal{P}^{\mathcal{C}}$ for Scene: [**Veach Bidir**] Algorithm: [**M-MLT**] Metric: [**FSIMc**] True GT: [**BDPT @ 524288 spp**].

ERPT	2	4	8	16	32	64	128	256	512	1024	2048	4096	8192	16384	32768	65536	131072
GT (BDPT)																	
262144	0.00009	0.00009	0.00009	0.00005	0.00007	0.00008	0.00008	0.00006	0.00005	0.00006	0.00006	0.00006	0.00008	0.00007	0.00001	0.00006	0.00006
131072	0.00018	0.00020	0.00018	0.00013	0.00016	0.00020	0.00016	0.00018	0.00012	0.00019	0.00018	0.00018	0.00013	0.00016	0.00013	0.00021	
65536	0.00052	0.00052	0.00054	0.00052	0.00051	0.00050	0.00050	0.00049	0.00048	0.00045	0.00047	0.00046	0.00050	0.00050	0.00047		
32768	0.00106	0.00103	0.00101	0.00104	0.00103	0.00097	0.00099	0.00104	0.00097	0.00106	0.00104	0.00099	0.00103	0.00104			
16384	0.00160	0.00161	0.00153	0.00152	0.00152	0.00157	0.00151	0.00142	0.00142	0.00145	0.00150	0.00148	0.00147				
8192	0.00277	0.00277	0.00273	0.00285	0.00275	0.00270	0.00279	0.00281	0.00274	0.00263	0.00264	0.00264					
4096	0.00420	0.00438	0.00429	0.00429	0.00423	0.00420	0.00426	0.00428	0.00418	0.00429	0.00435						
2048	0.00719	0.00705	0.00725	0.00707	0.00729	0.00712	0.00716	0.00706	0.00704	0.00713							
1024	0.01285	0.01276	0.01280	0.01282	0.01287	0.01260	0.01276	0.01230	0.01249								
512	0.01936	0.01918	0.01928	0.01905	0.01912	0.01878	0.01913	0.01843									
256	0.02829	0.02820	0.02797	0.02821	0.02826	0.02806	0.02785										
128	0.03596	0.03592	0.03538	0.03565	0.03549	0.03603											
64	0.04202	0.04168	0.04186	0.04201	0.04180												
32	0.04388	0.04179	0.04222	0.04182													
16	0.03364	0.03125	0.03152														
8	0.00325	0.00008															
4	-0.05291																

(f) $\mathcal{P}^{\mathcal{C}}$ for Scene: [**Veach Bidir**] Algorithm: [**ERPT**] Metric: [**FSIMc**] True GT: [**BDPT @ 524288 spp**].

M-ERPT	2	4	8	16	32	64	128	256	512	1024	2048	4096	8192	16384	32768	65536	131072
GT (BDPT)																	
262144	0.00011	0.00008	0.00005	0.00008	0.00007	0.00007	0.00005	0.00009	0.00002	0.00006	0.00008	0.00007	0.00005	0.00005	0.00006	0.00004	0.00006
131072	0.00017	0.00015	0.00012	0.00017	0.00016	0.00020	0.00014	0.00016	0.00017	0.00013	0.00017	0.00015	0.00017	0.00014	0.00017	0.00015	
65536	0.00057	0.00055	0.00052	0.00055	0.00050	0.00054	0.00042	0.00048	0.00047	0.00050	0.00046	0.00051	0.00049	0.00047	0.00050		
32768	0.00103	0.00100	0.00102	0.00107	0.00106	0.00101	0.00092	0.00098	0.00107	0.00107	0.00101	0.00096	0.00105	0.00099			
16384	0.00157	0.00154	0.00144	0.00160	0.00152	0.00147	0.00142	0.00140	0.00154	0.00140	0.00146	0.00144	0.00139				
8192	0.00277	0.00268	0.00275	0.00286	0.00271	0.00275	0.00259	0.00258	0.00266	0.00268	0.00266	0.00275					
4096	0.00429	0.00429	0.00430	0.00440	0.00432	0.00436	0.00410	0.00413	0.00418	0.00432	0.00429						
2048	0.00727	0.00727	0.00721	0.00723	0.00722	0.00710	0.00706	0.00681	0.00722	0.00716							
1024	0.01294	0.01301	0.01273	0.01314	0.01283	0.01252	0.01196	0.01195	0.01252								
512	0.01957	0.02006	0.01902	0.01966	0.01917	0.01888	0.01790	0.01825									
256	0.02891	0.02895	0.02852	0.02848	0.02866	0.02813	0.02683										
128	0.03677	0.03680	0.03555	0.03678	0.03597	0.03616											
64	0.04394	0.04323	0.04284	0.04350	0.04243												
32	0.04553	0.04548	0.04543	0.04580													
16	0.03620	0.03535	0.03565														
8	0.00648	0.00381															
4	-0.04792																

(g) $\mathcal{P}^{\mathcal{C}}$ for Scene: [**Veach Bidir**] Algorithm: [**M-ERPT**] Metric: [**FSIMc**] True GT: [**BDPT @ 524288 spp**].

Table 48

11.4 Veach Door

PT	2	4	8	16	32	64	128	256	512	1024	2048	4096	8192	16384	32768	65536	131072
GT (BDPT)																	
262144	0.00029	0.00005	0.00022	0.00045	0.00049	0.00063	0.00064	0.00064	0.00066	0.00059	0.00046	0.00039	0.00021	0.00028	0.00014	0.00005	0.00004
131072	0.00071	0.00038	0.00069	0.00088	0.00140	0.00185	0.00209	0.00212	0.00198	0.00173	0.00158	0.00115	0.00095	0.00081	0.00044	0.00023	
65536	0.00175	0.00201	0.00235	0.00297	0.00389	0.00463	0.00492	0.00489	0.00443	0.00394	0.00340	0.00275	0.00189	0.00140	0.00086		
32768	0.00232	0.00275	0.00387	0.00498	0.00677	0.00827	0.00901	0.00864	0.00822	0.00716	0.00593	0.00459	0.00330	0.00239			
16384	0.00639	0.00687	0.00879	0.00967	0.01281	0.01474	0.01606	0.01539	0.01402	0.01234	0.01028	0.00786	0.00529				
8192	0.00960	0.01155	0.01359	0.01677	0.02083	0.02451	0.02615	0.02481	0.02263	0.01921	0.01551	0.01138					
4096	0.01295	0.01518	0.01970	0.02364	0.03075	0.03670	0.03833	0.03644	0.03316	0.02748	0.02161						
2048	0.01975	0.02529	0.03124	0.03566	0.04590	0.05330	0.05536	0.05203	0.04573	0.03663							
1024	0.03276	0.04270	0.05024	0.05795	0.06969	0.07953	0.07949	0.07284	0.06151								
512	0.05392	0.06479	0.07594	0.08441	0.09670	0.10588	0.10409	0.09121									
256	0.09073	0.10584	0.11475	0.11984	0.13097	0.13691	0.12728										
128	0.14177	0.15583	0.16320	0.16251	0.16955	0.16619											
64	0.20829	0.21894	0.22161	0.21075	0.20247												
32	0.29695	0.30356	0.28864	0.25729													
16	0.39801	0.39317	0.35167														
8	0.49900	0.47466															
4	0.58300																

(a) $\mathcal{P}^{\mathcal{C}}$ for Scene: [**Veach Door**] Algorithm: [**PT**] Metric: [**FSIMc**] True GT: [**BDPT @ 524288 spp**].

BDPT	2	4	8	16	32	64	128	256	512	1024	2048	4096	8192	16384	32768	65536	131072
GT (BDPT)																	
262144	0.00013	0.00031	0.00039	0.00036	0.00068	0.00067	0.00070	0.00062	0.00049	0.00036	0.00026	0.00019	0.00006	0.00005	0.00000	-0.00003	-0.00006
131072	0.00049	0.00074	0.00097	0.00132	0.00179	0.00185	0.00195	0.00187	0.00157	0.00111	0.00084	0.00055	0.00029	0.00008	-0.00002	-0.00013	
65536	0.00217	0.00243	0.00324	0.00377	0.00435	0.00473	0.00444	0.00391	0.00327	0.00231	0.00161	0.00099	0.00047	0.00013	-0.00020		
32768	0.00327	0.00427	0.00528	0.00603	0.00805	0.00863	0.00830	0.00698	0.00584	0.00425	0.00280	0.00165	0.00053	-0.00019			
16384	0.00730	0.00809	0.01008	0.01229	0.01454	0.01450	0.01363	0.01184	0.00963	0.00687	0.00432	0.00231	0.00044				
8192	0.01068	0.01286	0.01660	0.02040	0.02354	0.02386	0.02224	0.01861	0.01477	0.00999	0.00564	0.00203					
4096	0.01440	0.01762	0.02333	0.02983	0.03472	0.03472	0.03224	0.02665	0.02016	0.01272	0.00547						
2048	0.02502	0.02822	0.03654	0.04402	0.05053	0.04942	0.04473	0.03551	0.02554	0.01309							
1024	0.04167	0.04705	0.05811	0.06715	0.07380	0.07001	0.05949	0.04330	0.02755								
512	0.06454	0.07130	0.08338	0.09341	0.09846	0.08874	0.07197	0.04714									
256	0.10457	0.10864	0.11950	0.12385	0.12453	0.10565	0.07650										
128	0.15412	0.15541	0.15978	0.15847	0.14655	0.11355											
64	0.21662	0.21239	0.20712	0.18604	0.15797												
32	0.29989	0.28049	0.24946	0.20642													
16	0.38411	0.34254	0.28079														
8	0.46737	0.38638															
4	0.51394																

(b) $\mathcal{P}^{\mathcal{C}}$ for Scene: [**Veach Door**] Algorithm: [**BDPT**] Metric: [**FSIMc**] True GT: [**BDPT @ 524288 spp**].

PSSMLT	2	4	8	16	32	64	128	256	512	1024	2048	4096	8192	16384
GT (BDPT)														
262144	0.00028	0.00050	0.00055	0.00057	0.00052	0.00059	0.00048	0.00041	0.00030	0.00022	0.00009	0.00007	0.00001	-0.00004
131072	0.00088	0.00097	0.00135	0.00146	0.00164	0.00176	0.00154	0.00140	0.00109	0.00078	0.00048	0.00027	0.00008	-0.00005
65536	0.00255	0.00281	0.00348	0.00370	0.00389	0.00392	0.00347	0.00297	0.00219	0.00151	0.00085	0.00041	0.00011	-0.00018
32768	0.00445	0.00496	0.00627	0.00678	0.00723	0.00728	0.00623	0.00526	0.00387	0.00264	0.00153	0.00063	-0.00008	-0.00053
16384	0.00902	0.01000	0.01164	0.01208	0.01227	0.01237	0.01039	0.00847	0.00621	0.00407	0.00209	0.00054	-0.00060	
8192	0.01491	0.01670	0.01908	0.01983	0.02019	0.01932	0.01605	0.01276	0.00868	0.00498	0.00186	-0.00068		
4096	0.02196	0.02484	0.02868	0.02878	0.02856	0.02846	0.02287	0.01701	0.01094	0.00497	0.00017			
2048	0.03369	0.03808	0.04230	0.04163	0.04035	0.03839	0.02967	0.02099	0.01087	0.00220				
1024	0.05372	0.05771	0.06331	0.06109	0.05636	0.05095	0.03589	0.02093	0.00582					
512	0.07842	0.08292	0.08742	0.08018	0.07001	0.05824	0.03567	0.01354						
256	0.11357	0.11425	0.11566	0.10026	0.08044	0.06032	0.02739							
128	0.16038	0.15322	0.14200	0.11516	0.08324	0.04892								
64	0.21133	0.19281	0.16447	0.12034	0.07056									
32	0.26512	0.22501	0.17110	0.10628										
16	0.31218	0.24281	0.15565											
8	0.33487	0.23034												
4	0.31293													

(c) $\mathcal{P}^{\mathcal{C}}$ for Scene: [**Veach Door**] Algorithm: [**PSSMLT**] Metric: [**FSIMc**] True GT: [**BDPT @ 524288 spp**].

MLT	2	4	8	16	32	64	128	256	512	1024	2048	4096	8192	16384
GT (BDPT)														
262144	0.00024	0.00040	0.00047	0.00054	0.00061	0.00048	0.00052	0.00032	0.00036	0.00025	0.00016	0.00008	0.00001	-0.00001
131072	0.00068	0.00120	0.00139	0.00170	0.00170	0.00163	0.00158	0.00125	0.00103	0.00071	0.00036	0.00020	0.00004	-0.00007
65536	0.00211	0.00318	0.00335	0.00401	0.00393	0.00336	0.00331	0.00247	0.00196	0.00133	0.00080	0.00035	-0.00001	-0.00023
32768	0.00363	0.00515	0.00625	0.00715	0.00692	0.00619	0.00608	0.00471	0.00345	0.00215	0.00113	0.00023	-0.00038	-0.00077
16384	0.00739	0.01009	0.01165	0.01284	0.01232	0.01070	0.00994	0.00737	0.00539	0.00324	0.00126	-0.00004	-0.00103	
8192	0.01207	0.01700	0.01951	0.02055	0.01953	0.01658	0.01552	0.01088	0.00731	0.00381	0.00069	-0.00163		
4096	0.01663	0.02459	0.02739	0.02978	0.02856	0.02357	0.02145	0.01402	0.00864	0.00302	-0.00204			
2048	0.02453	0.03682	0.04092	0.04253	0.03954	0.03128	0.02722	0.01606	0.00759	-0.00095				
1024	0.04029	0.05594	0.06017	0.06089	0.05309	0.03854	0.03177	0.01456	0.00134					
512	0.05975	0.07974	0.08293	0.07838	0.06503	0.04197	0.02924	0.00381						
256	0.09106	0.11039	0.10643	0.09498	0.07182	0.03864	0.01757							
128	0.12984	0.14585	0.12898	0.10485	0.06777	0.02110								
64	0.17652	0.17954	0.14805	0.10552	0.05002									
32	0.22761	0.20835	0.14843	0.08485										
16	0.27353	0.22415	0.13279											
8	0.29300	0.20245												
4	0.27598													

(d) $\mathcal{P}^{\mathcal{C}}$ for Scene: [**Veach Door**] Algorithm: [**MLT**] Metric: [**FSIMc**] True GT: [**BDPT @ 524288 spp**].

M-MLT	2	4	8	16	32	64	128	256	512	1024	2048	4096	8192	16384
GT (BDPT)														
262144	0.00032	0.00037	0.00038	0.00047	0.00063	0.00054	0.00047	0.00033	0.00025	0.00019	0.00006	0.00009	-0.00001	-0.00002
131072	0.00098	0.00126	0.00150	0.00143	0.00168	0.00164	0.00148	0.00117	0.00083	0.00065	0.00035	0.00019	-0.00002	-0.00008
65536	0.00270	0.00363	0.00364	0.00360	0.00395	0.00357	0.00316	0.00239	0.00175	0.00122	0.00064	0.00025	-0.00015	-0.00030
32768	0.00468	0.00591	0.00622	0.00644	0.00712	0.00606	0.00558	0.00435	0.00313	0.00180	0.00104	0.00014	-0.00053	-0.00078
16384	0.00984	0.01157	0.01180	0.01193	0.01220	0.01063	0.00933	0.00673	0.00494	0.00273	0.00115	-0.00033	-0.00128	
8192	0.01591	0.01907	0.01982	0.01903	0.01946	0.01691	0.01424	0.00988	0.00651	0.00313	0.00037	-0.00190		
4096	0.02297	0.02802	0.02897	0.02787	0.02800	0.02390	0.01952	0.01301	0.00725	0.00189	-0.00245			
2048	0.03522	0.04127	0.04263	0.04024	0.03909	0.03177	0.02405	0.01444	0.00540	-0.00308				
1024	0.05400	0.06393	0.06230	0.05688	0.05273	0.04008	0.02773	0.01164	-0.00210					
512	0.07851	0.08893	0.08395	0.07117	0.06300	0.04345	0.02318	0.00076						
256	0.11410	0.12123	0.10988	0.08743	0.06824	0.03810	0.01012							
128	0.15745	0.15407	0.13079	0.09669	0.06460	0.02208								
64	0.20530	0.19108	0.14476	0.09453	0.04536									
32	0.25344	0.21536	0.14517	0.07380										
16	0.29655	0.22810	0.12146											
8	0.31099	0.20385												
4	0.28992													

(e) $\mathcal{P}^{\mathcal{C}}$ for Scene: [**Veach Door**] Algorithm: [**M-MLT**] Metric: [**FSIMc**] True GT: [**BDPT @ 524288 spp**].

ERPT	2	4	8	16	32	64	128	256	512	1024	2048	4096	8192	16384	32768	65536	131072
GT (BDPT)																	
262144	0.00044	0.00050	0.00051	0.00047	0.00048	0.00043	0.00041	0.00044	0.00042	0.00045	0.00039	0.00043	0.00044	0.00046	0.00042	0.00040	0.00045
131072	0.00134	0.00150	0.00144	0.00147	0.00145	0.00140	0.00136	0.00142	0.00144	0.00136	0.00142	0.00140	0.00141	0.00155	0.00142	0.00141	
65536	0.00276	0.00300	0.00293	0.00296	0.00296	0.00298	0.00288	0.00301	0.00290	0.00287	0.00293	0.00294	0.00296	0.00303	0.00283		
32768	0.00500	0.00546	0.00545	0.00559	0.00543	0.00544	0.00533	0.00536	0.00536	0.00533	0.00545	0.00522	0.00539	0.00565			
16384	0.00855	0.00864	0.00909	0.00893	0.00895	0.00877	0.00873	0.00887	0.00885	0.00856	0.00875	0.00884	0.00919				
8192	0.01306	0.01324	0.01352	0.01373	0.01356	0.01334	0.01303	0.01341	0.01316	0.01276	0.01342	0.01318					
4096	0.01753	0.01820	0.01873	0.01893	0.01862	0.01852	0.01804	0.01850	0.01827	0.01759	0.01778						
2048	0.02095	0.02209	0.02271	0.02237	0.02321	0.02244	0.02196	0.02263	0.02255	0.02040							
1024	0.02265	0.02388	0.02481	0.02434	0.02470	0.02366	0.02369	0.02466	0.02453								
512	0.01709	0.01791	0.01919	0.01852	0.01903	0.01840	0.01759	0.01887									
256	0.00160	0.00334	0.00291	0.00359	0.00382	0.00322	0.00297										
128	-0.02847	-0.02544	-0.02659	-0.02743	-0.02776	-0.02843											
64	-0.07651	-0.07442	-0.07550	-0.07739	-0.07855												
32	-0.14173	-0.14113	-0.14582	-0.14540													
16	-0.22499	-0.22932	-0.23418														
8	-0.34109	-0.34224															
4	-0.49396																

(f) $\mathcal{P}^{\mathcal{C}}$ for Scene: [**Veach Door**] Algorithm: [**ERPT**] Metric: [**FSIMc**] True GT: [**BDPT @ 524288 spp**].

M-ERPT	2	4	8	16	32	64	128	256	512	1024	2048	4096	8192	16384	32768	65536	131072
GT (BDPT)																	
262144	0.00053	0.00044	0.00045	0.00046	0.00045	0.00045	0.00050	0.00046	0.00049	0.00047	0.00048	0.00045	0.00045	0.00044	0.00041	0.00044	0.00040
131072	0.00148	0.00143	0.00133	0.00142	0.00147	0.00136	0.00139	0.00148	0.00150	0.00139	0.00150	0.00140	0.00138	0.00151	0.00142	0.00144	
65536	0.00309	0.00295	0.00298	0.00291	0.00296	0.00304	0.00299	0.00298	0.00314	0.00304	0.00311	0.00300	0.00291	0.00312	0.00293		
32768	0.00566	0.00529	0.00554	0.00520	0.00539	0.00550	0.00548	0.00553	0.00565	0.00546	0.00561	0.00558	0.00551	0.00537			
16384	0.00928	0.00894	0.00872	0.00884	0.00891	0.00904	0.00906	0.00908	0.00925	0.00867	0.00913	0.00924	0.00903				
8192	0.01439	0.01381	0.01364	0.01334	0.01345	0.01382	0.01396	0.01387	0.01396	0.01318	0.01401	0.01400					
4096	0.01971	0.01860	0.01831	0.01818	0.01851	0.01890	0.01876	0.01890	0.01916	0.01846	0.01944						
2048	0.02494	0.02296	0.02266	0.02276	0.02286	0.02364	0.02283	0.02352	0.02403	0.02249							
1024	0.02796	0.02510	0.02464	0.02410	0.02433	0.02566	0.02543	0.02593	0.02625								
512	0.02328	0.02024	0.01923	0.01882	0.01942	0.02070	0.01976	0.02088									
256	0.00943	0.00595	0.00429	0.00312	0.00509	0.00490	0.00378										
128	-0.01965	-0.02277	-0.02476	-0.02649	-0.02579	-0.02339											
64	-0.06765	-0.07141	-0.07211	-0.07595	-0.07420												
32	-0.13413	-0.13836	-0.13982	-0.14465													
16	-0.22094	-0.22290	-0.22826														
8	-0.33901	-0.34165															
4	-0.49032																

(g) $\mathcal{P}^{\mathcal{C}}$ for Scene: [**Veach Door**] Algorithm: [**M-ERPT**] Metric: [**FSIMc**] True GT: [**BDPT @ 524288 spp**].

Table 49

11.5 Sponza

PT	2	4	8	16	32	64	128	256	512	1024	2048	4096	8192
GT (PT)													
16384	0.00364	0.00374	0.00368	0.00310	0.00294	0.00239	0.00174	0.00102	0.00068	0.00028	0.00005	-0.00024	-0.00035
8192	0.00930	0.00879	0.00841	0.00805	0.00690	0.00544	0.00429	0.00281	0.00133	0.00044	-0.00046	-0.00090	
4096	0.02123	0.02183	0.02024	0.01845	0.01632	0.01292	0.00939	0.00567	0.00299	0.00051	-0.00113		
2048	0.03988	0.03941	0.03705	0.03328	0.02883	0.02291	0.01586	0.00930	0.00401	-0.00031			
1024	0.07135	0.06964	0.06510	0.05818	0.04871	0.03762	0.02521	0.01339	0.00385				
512	0.11512	0.11278	0.10406	0.09031	0.07516	0.05559	0.03428	0.01489					
256	0.17483	0.17036	0.15465	0.13261	0.10425	0.07410	0.04110						
128	0.24572	0.23763	0.21048	0.17664	0.13247	0.08432							
64	0.32622	0.30932	0.26726	0.21571	0.14800								
32	0.40923	0.37842	0.31612	0.23776									
16	0.48787	0.43799	0.34727										
8	0.55101	0.47281											
4	0.58406												

(a) $\mathcal{P}^{\mathcal{C}}$ for Scene: [**Sponza**] Algorithm: [**PT**] Metric: [**FSIMc**] True GT: [**PT @ 32768 spp**].

BDPT	2	4	8	16	32	64	128	256	512	1024	2048	4096	8192
GT (PT)													
16384	0.00349	0.00386	0.00352	0.00342	0.00308	0.00253	0.00197	0.00137	0.00090	0.00038	0.00012	-0.00015	-0.00036
8192	0.00881	0.00901	0.00872	0.00812	0.00748	0.00603	0.00455	0.00314	0.00175	0.00057	-0.00010	-0.00072	
4096	0.02117	0.02157	0.02077	0.01942	0.01676	0.01384	0.01056	0.00720	0.00390	0.00134	-0.00076		
2048	0.03835	0.03991	0.03828	0.03487	0.03077	0.02485	0.01801	0.01176	0.00558	0.00069			
1024	0.06928	0.07032	0.06769	0.06087	0.05262	0.04148	0.02953	0.01703	0.00667				
512	0.11235	0.11316	0.10757	0.09634	0.08128	0.06281	0.04104	0.02082					
256	0.16902	0.17204	0.16002	0.14130	0.11721	0.08447	0.04979						
128	0.23825	0.24150	0.21941	0.18984	0.14951	0.10142							
64	0.32017	0.31750	0.28381	0.23708	0.17606								
32	0.40273	0.38893	0.33948	0.26814									
16	0.48527	0.45538	0.37946										
8	0.55397	0.49829											
4	0.60159												

(b) $\mathcal{P}^{\mathcal{C}}$ for Scene: [**Sponza**] Algorithm: [**BDPT**] Metric: [**FSIMc**] True GT: [**PT @ 32768 spp**].

PSSMLT	2	4	8	16	32	64	128	256	512	1024	2048	4096	8192
GT (PT)													
16384	0.00353	0.00371	0.00363	0.00321	0.00304	0.00252	0.00194	0.00140	0.00109	0.00068	0.00045	0.00017	0.00010
8192	0.00884	0.00915	0.00860	0.00767	0.00701	0.00603	0.00456	0.00345	0.00234	0.00128	0.00065	0.00015	
4096	0.02050	0.02030	0.01949	0.01795	0.01560	0.01301	0.01013	0.00750	0.00521	0.00325	0.00182		
2048	0.03704	0.03772	0.03497	0.03122	0.02762	0.02304	0.01778	0.01317	0.00834	0.00453			
1024	0.06499	0.06488	0.06052	0.05449	0.04730	0.03876	0.02953	0.02025	0.01160				
512	0.10422	0.10318	0.09496	0.08462	0.07080	0.05700	0.04040	0.02649					
256	0.15424	0.15375	0.14059	0.12104	0.09942	0.07573	0.04979						
128	0.21283	0.21039	0.18939	0.15879	0.12445	0.08909							
64	0.27922	0.27178	0.23757	0.19231	0.14065								
32	0.34331	0.32754	0.27575	0.21078									
16	0.39662	0.36491	0.28766										
8	0.43051	0.38024											
4	0.42970												

(c) $\mathcal{P}^{\mathcal{C}}$ for Scene: [**Sponza**] Algorithm: [**PSSMLT**] Metric: [**FSIMc**] True GT: [**PT @ 32768 spp**].

MLT	2	4	8	16	32	64	128	256	512	1024	2048	4096	8192
GT (PT)													
16384	0.00379	0.00303	0.00313	0.00273	0.00207	0.00176	0.00124	0.00063	0.00057	0.00021	0.00004	0.00004	-0.00012
8192	0.00901	0.00848	0.00790	0.00679	0.00536	0.00422	0.00275	0.00176	0.00098	0.00040	-0.00004	-0.00020	
4096	0.02080	0.01995	0.01760	0.01520	0.01238	0.00910	0.00622	0.00401	0.00227	0.00116	0.00019		
2048	0.03871	0.03619	0.03234	0.02742	0.02087	0.01568	0.00968	0.00583	0.00274	0.00058			
1024	0.06781	0.06304	0.05622	0.04673	0.03509	0.02472	0.01457	0.00755	0.00189				
512	0.10974	0.10082	0.08704	0.07066	0.05086	0.03398	0.01799	0.00573					
256	0.16289	0.14832	0.12564	0.09845	0.06669	0.04000	0.01458						
128	0.22934	0.20278	0.16512	0.12368	0.07472	0.03508							
64	0.29901	0.25792	0.20068	0.13974	0.06844								
32	0.36637	0.30267	0.22201	0.13425									
16	0.42247	0.32899	0.21572										
8	0.45501	0.32892											
4	0.45697												

(d) $\mathcal{P}^{\mathcal{C}}$ for Scene: [**Sponza**] Algorithm: [**MLT**] Metric: [**FSIMc**] True GT: [**PT @ 32768 spp**].

M-MLT	2	4	8	16	32	64	128	256	512	1024	2048	4096	8192
GT (PT)													
16384	0.00351	0.00357	0.00305	0.00256	0.00217	0.00154	0.00113	0.00080	0.00068	0.00019	0.00022	0.00011	0.00002
8192	0.00929	0.00848	0.00754	0.00630	0.00503	0.00376	0.00268	0.00149	0.00100	0.00039	0.00002	-0.00017	
4096	0.02090	0.01986	0.01778	0.01497	0.01152	0.00842	0.00596	0.00326	0.00228	0.00083	0.00001		
2048	0.03811	0.03606	0.03181	0.02652	0.02047	0.01461	0.00961	0.00512	0.00244	0.00066			
1024	0.06724	0.06282	0.05438	0.04434	0.03359	0.02299	0.01416	0.00644	0.00164				
512	0.10693	0.09912	0.08473	0.06687	0.04836	0.03169	0.01606	0.00394					
256	0.16080	0.14557	0.12252	0.09324	0.06285	0.03611	0.01205						
128	0.22253	0.19900	0.15998	0.11536	0.06968	0.02771							
64	0.29177	0.25241	0.19143	0.12641	0.06028								
32	0.35348	0.29521	0.21057	0.11834									
16	0.40435	0.31799	0.19769										
8	0.42905	0.31020											
4	0.41871												

(e) $\mathcal{P}^{\mathcal{C}}$ for Scene: [**Sponza**] Algorithm: [**M-MLT**] Metric: [**FSIMc**] True GT: [**PT @ 32768 spp**].

ERPT	2	4	8	16	32	64	128	256	512	1024	2048	4096	8192
GT (PT)													
16384	0.00099	0.00100	0.00114	0.00101	0.00092	0.00118	0.00094	0.00108	0.00085	0.00097	0.00122	0.00102	0.00120
8192	0.00229	0.00224	0.00240	0.00235	0.00229	0.00262	0.00241	0.00245	0.00237	0.00237	0.00240	0.00224	
4096	0.00522	0.00516	0.00583	0.00543	0.00544	0.00528	0.00528	0.00525	0.00549	0.00522	0.00509		
2048	0.00873	0.00839	0.00877	0.00853	0.00846	0.00842	0.00876	0.00832	0.00826	0.00819			
1024	0.01231	0.01164	0.01229	0.01226	0.01179	0.01177	0.01143	0.01245	0.01209				
512	0.01356	0.01321	0.01310	0.01318	0.01288	0.01252	0.01269	0.01229					
256	0.00913	0.00814	0.00818	0.00777	0.00719	0.00804	0.00831						
128	-0.01184	-0.01280	-0.01156	-0.01346	-0.01282	-0.01306							
64	-0.04830	-0.05262	-0.05186	-0.05374	-0.05263								
32	-0.11163	-0.11623	-0.11439	-0.11660									
16	-0.20648	-0.21399	-0.20992										
8	-0.32417	-0.33080											
4	-0.46108												

(f) $\mathcal{P}^{\mathcal{C}}$ for Scene: [**Sponza**] Algorithm: [**ERPT**] Metric: [**FSIMc**] True GT: [**PT @ 32768 spp**].

M-ERPT	2	4	8	16	32	64	128	256	512	1024	2048	4096	8192
GT (PT)													
16384	0.00105	0.00104	0.00125	0.00090	0.00093	0.00107	0.00114	0.00108	0.00115	0.00092	0.00103	0.00103	0.00108
8192	0.00248	0.00248	0.00242	0.00250	0.00255	0.00225	0.00246	0.00245	0.00253	0.00231	0.00249	0.00241	
4096	0.00554	0.00547	0.00505	0.00549	0.00537	0.00536	0.00529	0.00556	0.00569	0.00507	0.00536		
2048	0.00895	0.00900	0.00851	0.00878	0.00824	0.00863	0.00897	0.00839	0.00838	0.00840			
1024	0.01243	0.01335	0.01197	0.01303	0.01264	0.01266	0.01206	0.01223	0.01180				
512	0.01436	0.01492	0.01411	0.01462	0.01380	0.01373	0.01374	0.01353					
256	0.00971	0.00981	0.00905	0.01025	0.00827	0.00826	0.00928						
128	-0.00999	-0.00968	-0.01229	-0.00955	-0.01152	-0.01288							
64	-0.04834	-0.04723	-0.05211	-0.04935	-0.05085								
32	-0.11088	-0.10964	-0.11543	-0.11277									
16	-0.20630	-0.20533	-0.21260										
8	-0.32590	-0.32245											
4	-0.46225												

(g) $\mathcal{P}^{\mathcal{C}}$ for Scene: [**Sponza**] Algorithm: [**M-ERPT**] Metric: [**FSIMc**] True GT: [**PT @ 32768 spp**].

Table 50

12 HDR-VDP-2

12.1 Cornell Box

PT	2	4	8	16	32	64	128	256	512	1024	2048	4096	8192
**GT (PT)**													
16384	0.00023	-0.00081	0.00033	0.00004	-0.00001	-0.00010	-0.00028	0.00011	-0.00170	-0.00191	-0.00273	-0.00518	-0.01059
8192	0.00116	0.00063	0.00089	0.00157	0.00063	0.00046	0.00005	-0.00060	-0.00240	-0.00603	-0.00905	-0.01549	
4096	0.00346	0.00255	0.00202	0.00250	0.00125	0.00043	-0.00077	-0.00204	-0.00570	-0.01203	-0.02242		
2048	0.00788	0.00731	0.00789	0.00584	0.00360	0.00251	-0.00081	-0.00364	-0.01151	-0.02488			
1024	0.01732	0.01257	0.01346	0.01238	0.00822	0.00477	-0.00276	-0.01038	-0.02501				
512	0.03282	0.02849	0.02696	0.02069	0.01594	0.00863	-0.00422	-0.01592					
256	0.05488	0.04622	0.04615	0.03689	0.02577	0.01296	-0.00818						
128	0.07530	0.06802	0.06318	0.05067	0.03340	0.01483							
64	0.10945	0.09322	0.08512	0.06829	0.04191								
32	0.13999	0.11534	0.09895	0.07141									
16	0.15820	0.13259	0.10893										
8	0.18178	0.14276											
4	0.19135												

(a) $\mathcal{P}^{\mathcal{C}}$ for Scene: [**Cornell Box**] Algorithm: [**PT**] Metric: [**HDR-VDP-2**] True GT: [**PT @ 32768 spp**].

BDPT	2	4	8	16	32	64	128	256	512	1024	2048	4096	8192
**GT (PT)**													
16384	0.00069	0.00061	0.00023	0.00017	0.00070	-0.00122	-0.00049	-0.00019	-0.00438	-0.00663	-0.00472	-0.01012	-0.01582
8192	0.00135	0.00077	0.00099	0.00059	0.00003	-0.00055	-0.00104	-0.00320	-0.00575	-0.00867	-0.01418	-0.02252	
4096	0.00301	0.00112	0.00253	0.00150	0.00180	-0.00101	-0.00394	-0.00516	-0.01265	-0.02330	-0.03381		
2048	0.00523	0.00486	0.00434	0.00206	0.00025	-0.00148	-0.00711	-0.01443	-0.02351	-0.03825			
1024	0.00934	0.01054	0.00967	0.00628	0.00390	-0.00147	-0.01202	-0.02456	-0.04561				
512	0.02234	0.01974	0.01844	0.01213	0.00482	-0.00383	-0.02187	-0.04315					
256	0.03743	0.03500	0.02745	0.02212	0.00886	-0.01341	-0.04229						
128	0.06009	0.05272	0.04354	0.02893	0.00325	-0.02340							
64	0.08230	0.06972	0.05534	0.03304	-0.00370								
32	0.10520	0.08631	0.06176	0.02416									
16	0.12428	0.09279	0.05239										
8	0.13101	0.08409											
4	0.12523												

(b) $\mathcal{P}^{\mathcal{C}}$ for Scene: [**Cornell Box**] Algorithm: [**BDPT**] Metric: [**HDR-VDP-2**] True GT: [**PT @ 32768 spp**].

PSSMLT	2	4	8	16	32	64	128	256	512	1024	2048	4096	8192
**GT (PT)**													
16384	0.00045	0.00020	0.00015	0.00072	0.00026	0.00075	0.00050	0.00112	-0.00007	-0.00193	-0.00076	-0.00115	-0.00331
8192	0.00131	0.00058	0.00042	0.00062	0.00030	-0.00013	0.00077	-0.00002	-0.00001	-0.00117	-0.00194	-0.00259	
4096	0.00311	0.00241	0.00155	0.00140	0.00081	-0.00022	-0.00071	-0.00208	-0.00423	-0.01098	-0.00919		
2048	0.00641	0.00419	0.00292	0.00234	0.00099	-0.00011	-0.00367	-0.00372	-0.00714	-0.01533			
1024	0.01201	0.01035	0.00846	0.00698	0.00314	-0.00063	-0.00447	-0.00944	-0.01773				
512	0.02786	0.02206	0.01522	0.01215	0.00393	-0.00240	-0.01584	-0.02476					
256	0.04748	0.03805	0.02911	0.02011	0.00648	-0.00819	-0.03176						
128	0.06394	0.05341	0.04325	0.03028	0.00446	-0.02174							
64	0.09057	0.07737	0.05719	0.03183	-0.00477								
32	0.11525	0.09290	0.06281	0.02343									
16	0.13246	0.10310	0.05765										
8	0.14218	0.09820											
4	0.13823												

(c) $\mathcal{P}^{\mathcal{C}}$ for Scene: [**Cornell Box**] Algorithm: [**PSSMLT**] Metric: [**HDR-VDP-2**] True GT: [**PT @ 32768 spp**].

MLT	2	4	8	16	32	64	128	256	512	1024	2048	4096	8192
**GT (PT)**													
16384	0.00064	0.00084	0.00063	0.00083	0.00111	0.00090	0.00038	0.00016	-0.00043	-0.00066	-0.00086	-0.00267	0.00033
8192	0.00087	0.00110	0.00109	0.00097	0.00139	0.00090	0.00015	-0.00024	-0.00170	-0.00039	-0.00239	-0.00335	
4096	0.00348	0.00271	0.00232	0.00158	0.00238	0.00129	0.00115	-0.00068	-0.00425	-0.00366	-0.00911		
2048	0.00696	0.00714	0.00606	0.00590	0.00558	0.00566	0.00262	-0.00087	-0.00556	-0.00606			
1024	0.01741	0.01423	0.01168	0.01041	0.00883	0.00665	0.00289	-0.00287	-0.00935				
512	0.02725	0.02684	0.02306	0.01966	0.01613	0.01086	0.00412	-0.00557					
256	0.04638	0.04123	0.03314	0.03182	0.02416	0.01855	0.00504						
128	0.06935	0.06467	0.05571	0.04793	0.03372	0.02150							
64	0.09643	0.08689	0.07509	0.06218	0.04453								
32	0.12288	0.11081	0.09441	0.07682									
16	0.14791	0.13228	0.11045										
8	0.17214	0.15325											
4	0.19206												

(d) $\mathcal{P}^{\mathcal{C}}$ for Scene: [**Cornell Box**] Algorithm: [**MLT**] Metric: [**HDR-VDP-2**] True GT: [**PT @ 32768 spp**].

M-MLT	2	4	8	16	32	64	128	256	512	1024	2048	4096	8192
**GT (PT)**													
16384	0.00107	0.00092	-0.00008	0.00049	0.00030	0.00062	-0.00012	0.00044	0.00040	-0.00162	-0.00227	-0.00057	-0.00058
8192	0.00135	0.00128	0.00169	0.00157	0.00089	0.00072	0.00084	0.00020	-0.00002	-0.00126	-0.00166	-0.00388	
4096	0.00363	0.00311	0.00342	0.00290	0.00214	0.00127	-0.00017	-0.00087	-0.00165	-0.00289	-0.00505		
2048	0.00640	0.00528	0.00300	0.00540	0.00377	0.00239	0.00113	-0.00243	-0.00620	-0.01236			
1024	0.01391	0.01527	0.01233	0.01136	0.00838	0.00489	0.00206	-0.00086	-0.00628				
512	0.02743	0.02682	0.02565	0.01943	0.01493	0.00933	0.00240	-0.00493					
256	0.04533	0.04496	0.03788	0.03222	0.02569	0.02013	0.00598						
128	0.06496	0.06582	0.05719	0.04851	0.03598	0.02545							
64	0.09402	0.08982	0.07614	0.06774	0.04592								
32	0.11824	0.10878	0.09188	0.07657									
16	0.14594	0.13406	0.11409										
8	0.16538	0.15155											
4	0.18964												

(e) $\mathcal{P}^{\mathcal{C}}$ for Scene: [**Cornell Box**] Algorithm: [**M-MLT**] Metric: [**HDR-VDP-2**] True GT: [**PT @ 32768 spp**].

ERPT	2	4	8	16	32	64	128	256	512	1024	2048	4096	8192
**GT (PT)**													
16384	-0.00016	-0.00016	0.00068	0.00023	0.00011	0.00016	0.00080	0.00135	0.00060	-0.00031	0.00070	0.00060	-0.00020
8192	-0.00013	0.00050	-0.00076	0.00008	0.00041	-0.00082	0.00001	0.00098	0.00083	0.00048	-0.00012	0.00081	
4096	0.00113	0.00047	0.00135	0.00052	0.00026	-0.00083	0.00076	0.00019	0.00044	0.00013	-0.00003		
2048	0.00106	0.00070	0.00104	0.00053	-0.00040	0.00070	0.00135	0.00254	0.00119	-0.00035			
1024	0.00290	0.00181	0.00277	0.00104	0.00304	0.00026	0.00424	0.00337	0.00063				
512	0.00446	0.00754	0.00380	0.00142	-0.00064	0.00117	0.00312	0.00540					
256	0.00485	0.00374	0.00304	0.00095	0.00412	0.00207	0.00594						
128	0.00220	-0.00227	-0.00016	-0.00248	-0.00067	-0.00468							
64	-0.01026	-0.01262	-0.01398	-0.01884	-0.01375								
32	-0.03530	-0.03752	-0.04104	-0.04704									
16	-0.07251	-0.07529	-0.07735										
8	-0.11816	-0.11779											
4	-0.16854												

(f) $\mathcal{P}^{\mathcal{C}}$ for Scene: [**Cornell Box**] Algorithm: [**ERPT**] Metric: [**HDR-VDP-2**] True GT: [**PT @ 32768 spp**].

M-ERPT	2	4	8	16	32	64	128	256	512	1024	2048	4096	8192
**GT (PT)**													
16384	0.00043	0.00008	0.00022	0.00109	-0.00035	-0.00008	0.00040	-0.00032	-0.00009	0.00043	0.00059	-0.00022	0.00002
8192	0.00059	0.00084	0.00009	0.00020	0.00025	-0.00001	0.00031	0.00086	0.00106	0.00064	0.00121	-0.00001	
4096	-0.00000	0.00065	0.00003	0.00063	-0.00069	0.00082	0.00162	0.00119	0.00101	0.00006	0.00080		
2048	0.00116	0.00131	-0.00062	0.00383	0.00048	0.00112	0.00014	0.00232	0.00148	0.00129			
1024	0.00298	0.00221	0.00200	0.00390	0.00076	0.00346	0.00233	0.00297	-0.00290				
512	0.00568	0.00445	0.00224	0.00470	0.00667	0.00596	0.00453	0.00481					
256	0.00325	0.00677	0.00427	0.00556	0.00325	0.00665	-0.00092						
128	0.00026	0.00437	0.00032	0.00175	0.00052	0.00242							
64	-0.01392	-0.00980	-0.01325	-0.01043	-0.01459								
32	-0.03989	-0.03413	-0.03872	-0.03293									
16	-0.07440	-0.06844	-0.07455										
8	-0.11938	-0.11000											
4	-0.16748												

(g) $\mathcal{P}^{\mathcal{C}}$ for Scene: [**Cornell Box**] Algorithm: [**M-ERPT**] Metric: [**HDR-VDP-2**] True GT: [**PT @ 32768 spp**].

Table 51

12.2 Torus

PT	2	4	8	16	32	64	128	256	512	1024	2048	4096	8192	16384	32768	65536	131072
GT (BDPT)																	
262144	0.00673	0.00467	0.00648	0.00471	0.00864	0.00604	0.00567	0.00746	0.00888	0.00672	0.00581	0.00513	0.00310	0.00058	0.00210	-0.00262	-0.01462
131072	0.01580	0.01422	0.01471	0.01620	0.02701	0.01771	0.01842	0.02241	0.01982	0.01825	0.01722	0.01562	0.01154	0.00552	0.00295	-0.01093	
65536	0.02639	0.02394	0.02482	0.02571	0.03301	0.02527	0.03361	0.03470	0.03216	0.03139	0.02937	0.02902	0.02195	0.00978	0.00270		
32768	0.05265	0.03551	0.03982	0.04039	0.04682	0.04565	0.05100	0.05888	0.05253	0.04853	0.04724	0.04833	0.03273	0.00861			
16384	0.06707	0.06091	0.05607	0.05265	0.07152	0.05629	0.06012	0.07767	0.06799	0.06110	0.06322	0.05752	0.03593				
8192	0.09100	0.07407	0.06784	0.05705	0.09240	0.06927	0.06087	0.08958	0.08110	0.07144	0.07315	0.06362					
4096	0.09726	0.07903	0.08140	0.06650	0.09047	0.07284	0.07992	0.10244	0.08725	0.07199	0.07742						
2048	0.10313	0.10107	0.06546	0.05907	0.06630	0.07320	0.07285	0.10580	0.07438	0.06851							
1024	0.09369	0.09109	0.11488	0.06314	0.09827	0.07251	0.07523	0.08171	0.08050								
512	0.06106	0.09323	0.06595	0.05766	0.08160	0.06003	0.07509	0.06100									
256	0.08615	0.07224	0.09912	0.06351	0.09936	0.06277	0.07908										
128	0.09918	0.07113	0.05807	0.06977	0.07733	0.05855											
64	0.10528	0.09752	0.07884	0.09310	0.05182												
32	0.10465	0.10403	0.09161	0.09640													
16	0.14232	0.12448	0.11203														
8	0.16331	0.13811															
4	0.18797																

(a) $\mathcal{P}^{\mathcal{C}}$ for Scene: [**Torus**] Algorithm: [**PT**] Metric: [**HDR-VDP-2**] True GT: [**BDPT @ 524288 spp**].

BDPT	2	4	8	16	32	64	128	256	512	1024	2048	4096	8192	16384	32768	65536	131072
GT (BDPT)																	
262144	0.00406	0.00791	0.00819	0.00748	0.00437	0.00464	0.00589	0.00809	0.00792	0.00655	0.00689	0.00787	0.00389	0.00333	-0.00005	-0.00797	-0.01080
131072	0.00830	0.01883	0.01781	0.02178	0.01533	0.01604	0.01635	0.01918	0.02381	0.01837	0.01826	0.01583	0.01254	0.00746	0.00237	-0.00505	
65536	0.02146	0.02816	0.03067	0.03201	0.03057	0.03186	0.02827	0.03641	0.04273	0.03315	0.03230	0.02890	0.02108	0.01347	0.00081		
32768	0.03480	0.03883	0.04353	0.05040	0.04922	0.04751	0.04916	0.05485	0.06461	0.05285	0.05236	0.04464	0.03281	0.01347			
16384	0.04268	0.04672	0.06729	0.06692	0.06042	0.07050	0.06155	0.07091	0.07626	0.06698	0.06700	0.05619	0.03785				
8192	0.05099	0.05202	0.06657	0.08061	0.07600	0.07434	0.06543	0.08154	0.09306	0.08012	0.08105	0.06054					
4096	0.05047	0.08144	0.07597	0.08039	0.07709	0.08751	0.08060	0.10052	0.09902	0.08464	0.08134						
2048	0.05070	0.08699	0.07417	0.06742	0.07465	0.07293	0.08314	0.09222	0.08771	0.07953							
1024	0.07108	0.09175	0.09943	0.08066	0.08186	0.07346	0.06967	0.08713	0.07945								
512	0.04433	0.05250	0.08269	0.07876	0.08223	0.08442	0.07037	0.07331									
256	0.04491	0.06287	0.07443	0.08492	0.07544	0.07040	0.06133										
128	0.07590	0.07735	0.07380	0.08743	0.08188	0.07112											
64	0.08553	0.09725	0.08417	0.09228	0.07313												
32	0.11725	0.12316	0.09709	0.10118													
16	0.14589	0.14216	0.13133														
8	0.16920	0.14852															
4	0.17832																

(b) $\mathcal{P}^{\mathcal{C}}$ for Scene: [**Torus**] Algorithm: [**BDPT**] Metric: [**HDR-VDP-2**] True GT: [**BDPT @ 524288 spp**].

PSSMLT	2	4	8	16	32	64	128	256	512	1024	2048	4096	8192	16384
GT (BDPT)														
262144	0.01293	0.00486	0.01142	0.00498	0.00679	0.00430	0.00372	0.00201	0.00174	0.00255	0.00075	0.00185	0.00550	0.00087
131072	0.02623	0.01176	0.02243	0.01326	0.01358	0.01196	0.01135	0.00922	0.00790	0.00890	0.01484	0.01546	0.01580	0.01041
65536	0.04891	0.02591	0.04098	0.02591	0.02330	0.02340	0.02035	0.02052	0.01487	0.01737	0.02688	0.02529	0.02925	0.02212
32768	0.06664	0.04648	0.06946	0.03894	0.03601	0.03185	0.02894	0.02705	0.02399	0.02575	0.03553	0.03558	0.03389	0.01890
16384	0.08548	0.06419	0.09403	0.06304	0.05501	0.04614	0.04928	0.04135	0.03424	0.03517	0.03808	0.03383	0.02147	
8192	0.11596	0.08586	0.12150	0.07803	0.05874	0.05342	0.05808	0.05659	0.05278	0.04701	0.03899	0.01995		
4096	0.11874	0.10554	0.15046	0.10266	0.08591	0.07877	0.07156	0.06757	0.05004	0.04276	0.02005			
2048	0.12301	0.10336	0.13466	0.04370	0.03957	0.03793	0.03756	0.02835	0.02842	0.01139				
1024	0.11470	0.10045	0.11797	0.09095	0.09013	0.08155	0.05677	0.04550	0.03321					
512	0.11583	0.13579	0.13627	0.12128	0.06220	0.05533	0.05085	0.04004						
256	0.11356	0.09620	0.12749	0.09264	0.07422	0.06280	0.03303							
128	0.09740	0.09199	0.08686	0.08073	0.07658	0.05021								
64	0.09628	0.09103	0.07281	0.07790	0.07151									
32	0.08353	0.11087	0.08054	0.07809										
16	0.08830	0.11223	0.08742											
8	0.08425	0.10394												
4	0.11238													

(c) $\mathcal{P}^{\mathcal{C}}$ for Scene: [**Torus**] Algorithm: [**PSSMLT**] Metric: [**HDR-VDP-2**] True GT: [**BDPT @ 524288 spp**].

MLT	2	4	8	16	32	64	128	256	512	1024	2048	4096	8192	16384
GT (BDPT)														
262144	0.00360	0.00194	0.00737	0.00783	0.00086	0.01120	0.00299	0.00284	0.00291	0.00716	0.00574	0.00623	0.00571	0.00383
131072	0.00925	0.01174	0.01509	0.01724	0.00887	0.01723	0.00689	0.00281	0.00255	0.01236	0.00981	0.00591	0.00643	0.00214
65536	0.01334	0.01500	0.02226	0.02807	0.01859	0.03977	0.01276	0.00512	0.00805	0.01813	0.01108	0.00849	0.00888	0.00220
32768	0.01938	0.02738	0.03378	0.03523	0.02707	0.05198	0.01634	0.00998	0.01665	0.02496	0.01918	0.01296	0.01217	0.00221
16384	0.02697	0.03416	0.03533	0.04476	0.02210	0.06229	0.02239	0.01302	0.01279	0.01786	0.01688	0.00712	0.00293	
8192	0.02887	0.03870	0.05056	0.04765	0.03430	0.06821	0.02148	0.01461	0.00470	0.00864	-0.00146	-0.00797		
4096	0.03279	0.04339	0.04300	0.04404	0.04232	0.06102	0.01189	-0.00322	-0.02362	-0.02245	-0.03446			
2048	0.02785	0.03837	0.04045	0.04232	0.03477	0.04120	-0.00967	-0.03180	-0.05865	-0.06200				
1024	0.04013	0.03293	0.04299	0.03635	0.01306	0.01360	-0.05014	-0.06666	-0.09802					
512	0.03870	0.03365	0.03185	0.02030	-0.01399	-0.02321	-0.08602	-0.10651						
256	0.03999	0.02671	0.03245	-0.00103	-0.03802	-0.05786	-0.11457							
128	0.03632	0.02293	0.02001	-0.01260	-0.06570	-0.08876								
64	0.03176	0.02639	0.01526	-0.02216	-0.07217									
32	0.03818	0.01737	0.01925	-0.02164										
16	0.05480	0.01289	0.02552											
8	0.03172	0.00824												
4	0.06343													

(d) $\mathcal{P}^{\mathcal{C}}$ for Scene: [**Torus**] Algorithm: [**MLT**] Metric: [**HDR-VDP-2**] True GT: [**BDPT @ 524288 spp**].

M-MLT	2	4	8	16	32	64	128	256	512	1024	2048	4096	8192	16384
GT (BDPT)														
262144	0.00806	0.00576	0.00127	0.00528	0.00557	0.00278	0.00108	0.00140	0.00015	0.00710	0.00721	0.00618	0.00090	0.00107
131072	0.01565	0.01227	0.00693	0.00997	0.01280	0.00920	0.01124	0.00636	-0.00048	0.01106	0.01197	0.00736	0.00029	0.00310
65536	0.02684	0.01589	0.01444	0.01923	0.02225	0.01878	0.01546	0.00373	0.00292	0.01665	0.01672	0.00870	0.00198	0.00368
32768	0.03614	0.02445	0.02203	0.03001	0.03651	0.03328	0.02499	0.01728	0.00329	0.02520	0.02511	0.01833	-0.00028	0.00563
16384	0.03883	0.03398	0.02385	0.03737	0.04256	0.04639	0.02914	0.01666	0.00812	0.01778	0.01581	0.00619	0.00269	
8192	0.04877	0.04031	0.02888	0.04150	0.04809	0.05499	0.03828	0.03095	0.00670	0.00857	-0.00340	-0.01327		
4096	0.04908	0.04475	0.03200	0.04353	0.05218	0.06156	0.04058	0.02418	-0.00910	-0.02483	-0.03872			
2048	0.03958	0.04669	0.04654	0.04035	0.04551	0.05234	0.02063	-0.00618	-0.02928	-0.06550				
1024	0.04872	0.02244	0.02999	0.03182	0.02907	0.01618	0.00372	-0.03946	-0.06637					
512	0.03911	0.01787	0.01433	0.01995	0.02008	-0.00013	-0.01985	-0.07685						
256	0.01699	0.03054	0.01815	0.00667	-0.00471	-0.02823	-0.04922							
128	0.00010	0.04266	0.02130	-0.01531	-0.01969	-0.05257								
64	-0.00880	0.03120	0.02188	-0.01166	-0.02363									
32	-0.01535	0.04947	0.04435	-0.00162										
16	0.00475	0.04621	0.03719											
8	-0.02331	0.06108												
4	0.00503													

(e) $\mathcal{P}^{\mathcal{C}}$ for Scene: [**Torus**] Algorithm: [**M-MLT**] Metric: [**HDR-VDP-2**] True GT: [**BDPT @ 524288 spp**].

ERPT	2	4	8	16	32	64	128	256	512	1024	2048	4096	8192	16384	32768	65536	131072
GT (BDPT)																	
262144	0.00850	0.01042	0.00608	0.01092	0.00385	0.00744	0.00864	0.01101	0.00158	0.00540	0.00393	0.00002	0.00029	-0.00004	0.00049	0.00036	0.00072
131072	0.02137	0.01035	0.02050	0.01578	0.01253	0.02165	0.01298	0.02088	0.01013	0.01048	0.00747	0.00255	0.00337	0.00159	0.00196	-0.00027	
65536	0.04903	0.02387	0.03032	0.02199	0.02389	0.03581	0.02802	0.03256	0.01931	0.01950	0.01224	0.00255	0.00003	0.00096	0.00210		
32768	0.06267	0.04760	0.02862	0.03941	0.03861	0.04225	0.04087	0.04488	0.02344	0.02367	0.01589	-0.00031	-0.00212	-0.00059			
16384	0.10087	0.06132	0.04833	0.05348	0.05632	0.05945	0.04612	0.05301	0.02931	0.02326	0.01022	-0.00727	-0.00778				
8192	0.10224	0.08486	0.04928	0.04914	0.06886	0.05806	0.05253	0.05547	0.02653	0.01257	-0.00546	-0.02616					
4096	0.10715	0.06576	0.06265	0.03493	0.05963	0.05106	0.04443	0.03522	0.01602	-0.01064	-0.02942						
2048	0.08949	0.05390	0.04248	0.00206	0.04402	0.02898	0.02942	0.00116	-0.01643	-0.04842							
1024	0.05560	0.01384	0.01178	-0.04053	0.00832	0.00088	-0.00793	-0.03557	-0.05094								
512	0.01716	-0.02485	-0.01333	-0.07323	-0.02934	-0.03502	-0.04440	-0.07589									
256	-0.00592	-0.05347	-0.03783	-0.10580	-0.05659	-0.06240	-0.07215										
128	-0.03455	-0.08716	-0.06037	-0.13324	-0.08693	-0.09356											
64	-0.03867	-0.08953	-0.06839	-0.13703	-0.10159												
32	-0.04207	-0.09618	-0.07628	-0.13842													
16	-0.03836	-0.09275	-0.06422														
8	-0.04460	-0.08961															
4	-0.03101																

(f) $\mathcal{P}^{\mathcal{C}}$ for Scene: [**Torus**] Algorithm: [**ERPT**] Metric: [**HDR-VDP-2**] True GT: [**BDPT @ 524288 spp**].

M-ERPT	2	4	8	16	32	64	128	256	512	1024	2048	4096	8192	16384	32768	65536	131072
GT (BDPT)																	
262144	0.00246	-0.00434	0.00182	0.00089	0.00315	-0.00104	0.00316	0.00193	0.00103	0.00067	0.00072	0.00036	-0.00087	-0.00095	-0.00058	0.00111	0.00070
131072	0.00598	0.00617	0.00383	0.00411	0.00681	0.00284	0.00452	0.00467	0.00355	0.00915	0.00427	0.00009	0.00174	0.00128	0.00357	0.00271	
65536	0.00606	0.00800	0.00699	0.00639	0.01325	0.00809	0.00590	0.00966	0.01107	0.00920	0.00373	0.00013	0.00057	0.00141	0.00398		
32768	0.00913	0.01580	0.01011	0.00272	0.01944	0.00915	0.02725	0.02482	0.01362	0.02010	0.00393	-0.00157	0.00037	-0.00150			
16384	0.00995	0.04055	0.01543	0.00526	0.02074	0.01224	0.02559	0.02782	0.00948	0.01381	-0.00155	-0.00591	-0.00320				
8192	0.00509	0.04452	0.02114	0.01068	0.01902	0.00415	0.02253	0.01701	-0.00416	-0.00084	-0.01885	-0.02198					
4096	-0.00807	0.04153	0.00310	0.00312	-0.00241	-0.01199	0.00957	0.00684	-0.02658	-0.02544	-0.04372						
2048	-0.03359	0.03127	-0.01553	-0.00906	-0.03420	-0.03686	-0.02674	-0.02420	-0.06460	-0.06358							
1024	-0.06402	0.00145	-0.05324	-0.04225	-0.07236	-0.07183	-0.06373	-0.06469	-0.10525								
512	-0.09933	-0.03135	-0.09035	-0.06584	-0.10379	-0.10652	-0.09455	-0.09621									
256	-0.12569	-0.06548	-0.11534	-0.09279	-0.13454	-0.13625	-0.12496										
128	-0.15477	-0.07445	-0.13813	-0.12052	-0.15534	-0.16414											
64	-0.16600	-0.08429	-0.14700	-0.13081	-0.16630												
32	-0.16167	-0.07813	-0.15163	-0.13084													
16	-0.16312	-0.08417	-0.14548														
8	-0.16337	-0.08065															
4	-0.16573																

(g) $\mathcal{P}^{\mathcal{C}}$ for Scene: [**Torus**] Algorithm: [**M-ERPT**] Metric: [**HDR-VDP-2**] True GT: [**BDPT @ 524288 spp**].

Table 52

12.3 Veach Bidir

PT	2	4	8	16	32	64	128	256	512	1024	2048	4096	8192	16384	32768	65536	131072
GT (BDPT)																	
262144	-0.00021	0.00013	0.00001	0.00018	0.00053	-0.00053	-0.00027	-0.00077	0.00011	0.00032	-0.00089	0.00008	-0.00010	0.00010	0.00009	-0.00023	0.00002
131072	-0.00012	0.00024	0.00029	0.00026	0.00059	0.00015	0.00023	-0.00002	-0.00037	0.00028	-0.00009	0.00032	0.00035	0.00066	0.00044	0.00031	
65536	0.00048	0.00010	0.00085	0.00041	0.00098	0.00047	-0.00022	0.00032	-0.00028	0.00061	0.00052	-0.00019	-0.00001	0.00001	-0.00008		
32768	0.00041	0.00093	0.00188	0.00044	0.00172	-0.00036	-0.00018	-0.00079	0.00051	0.00116	0.00159	0.00038	0.00053	0.00096			
16384	0.00077	0.00121	0.00139	0.00090	0.00359	0.00040	0.00057	0.00016	0.00072	0.00100	-0.00036	0.00152	0.00123				
8192	0.00230	0.00346	0.00315	0.00160	0.00341	0.00121	0.00226	0.00156	0.00032	0.00309	0.00570	0.00268					
4096	0.00379	0.00374	0.00461	0.00172	0.00545	0.00257	0.00311	0.00347	0.00228	0.00490	0.00748						
2048	0.00748	0.00746	0.01026	0.00606	0.01106	0.00659	0.00698	0.00454	0.00404	0.00721							
1024	0.01444	0.01396	0.01725	0.01120	0.01752	0.01317	0.01469	0.01218	0.01171								
512	0.02645	0.02718	0.02793	0.01865	0.03288	0.02181	0.02389	0.02247									
256	0.04915	0.04427	0.04451	0.04096	0.04672	0.03953	0.04041										
128	0.07146	0.06648	0.06988	0.05936	0.07129	0.06101											
64	0.10469	0.09660	0.10398	0.08834	0.09732												
32	0.14042	0.13201	0.13520	0.12292													
16	0.17818	0.16798	0.16830														
8	0.21454	0.20069															
4	0.24558																

(a) $\mathcal{P}^{\mathcal{C}}$ for Scene: [**Veach Bidir**] Algorithm: [**PT**] Metric: [**HDR-VDP-2**] True GT: [**BDPT @ 524288 spp**].

BDPT	2	4	8	16	32	64	128	256	512	1024	2048	4096	8192	16384	32768	65536	131072
GT (BDPT)																	
262144	0.00010	0.00036	-0.00011	0.00021	0.00021	0.00060	0.01228	-0.00095	-0.00244	0.00600	-0.00329	-0.00309	0.00520	0.01362	0.00806	0.00227	-0.01092
131072	0.00024	-0.00021	0.00021	0.00014	0.00066	0.00043	0.01062	0.00349	0.00220	-0.00158	-0.00220	-0.00586	0.00804	0.00670	0.00409	-0.00945	
65536	0.00002	0.00017	0.00008	-0.00016	0.00015	-0.00024	0.00082	-0.00044	0.00169	-0.00275	-0.00736	0.00759	0.01308	0.00540	-0.00236		
32768	0.00066	0.00182	0.00072	0.00037	0.00279	-0.00014	0.04816	-0.00709	0.00168	-0.01394	0.00052	-0.01501	0.00767	-0.00215			
16384	0.00115	0.00193	-0.00142	0.00078	-0.00009	-0.00032	0.04973	-0.00447	-0.00386	-0.02131	-0.01993	-0.03783	0.00194				
8192	0.00148	0.00154	0.00101	0.00073	0.00255	-0.00058	-0.00361	-0.00868	-0.03355	-0.07184	-0.08495	-0.07527					
4096	0.00399	0.00283	0.00211	0.00334	0.00212	-0.00142	0.01481	0.00169	-0.01386	-0.03002	-0.04667						
2048	0.00592	0.00485	-0.00167	0.00321	0.00384	-0.00796	0.01556	-0.01207	-0.00737	-0.04589							
1024	0.01298	0.01261	0.00543	0.00964	0.01041	-0.00207	0.00652	-0.00486	-0.03786								
512	0.01966	0.02435	0.01418	0.02107	0.02008	0.00015	0.03410	-0.00884									
256	0.03568	0.03477	0.03103	0.03161	0.02506	0.00578	0.05617										
128	0.05566	0.05618	0.04584	0.04607	0.02634	0.00391											
64	0.07507	0.07251	0.06213	0.06336	0.03533												
32	0.09853	0.09432	0.07244	0.06680													
16	0.12241	0.11080	0.08342														
8	0.14227	0.11852															
4	0.15332																

(b) $\mathcal{P}^{\mathcal{C}}$ for Scene: [**Veach Bidir**] Algorithm: [**BDPT**] Metric: [**HDR-VDP-2**] True GT: [**BDPT @ 524288 spp**].

PSSMLT	2	4	8	16	32	64	128	256	512	1024	2048	4096	8192	16384
GT (BDPT)														
262144	0.00010	0.00006	0.00055	0.00031	0.00048	0.00028	-0.00034	-0.00064	-0.00048	0.00442	0.00197	0.00242	0.00064	0.00033
131072	0.00016	0.00034	0.00048	0.00051	0.00047	0.00014	-0.00095	-0.00251	-0.00061	0.00549	-0.00012	-0.00157	-0.00140	-0.00068
65536	-0.00005	0.00010	0.00015	0.00038	0.00091	0.00047	0.00124	0.00377	0.00105	0.00477	-0.00197	-0.00067	0.00005	0.00048
32768	0.00101	0.00035	0.00087	0.00086	0.00080	0.00103	0.00366	0.00836	-0.00201	0.02053	0.00051	-0.00624	-0.00425	-0.00284
16384	0.00040	0.00054	-0.00006	0.00053	0.00032	-0.00124	-0.00474	-0.00885	-0.01249	-0.00821	-0.01041	-0.01555	-0.01805	
8192	0.00310	0.00177	0.00206	0.00103	0.00217	-0.00054	-0.00547	-0.01017	-0.03501	-0.02803	-0.04002	-0.05244		
4096	0.00377	0.00279	0.00520	0.00473	0.00502	0.00108	-0.00218	-0.00693	-0.01623	-0.00851	-0.01176			
2048	0.00599	0.00466	0.00721	0.00700	0.00423	0.00196	0.00376	0.01306	-0.02389	-0.00685				
1024	0.01440	0.01365	0.01566	0.01365	0.00724	-0.00875	-0.00295	0.00097	-0.01671					
512	0.02355	0.02194	0.02250	0.02369	0.01566	0.00348	0.00113	-0.00056						
256	0.03834	0.03634	0.03917	0.03435	0.02535	0.00898	0.00797							
128	0.05480	0.05301	0.05449	0.05101	0.03176	-0.02211								
64	0.07708	0.07098	0.07133	0.06206	0.04062									
32	0.10429	0.09407	0.08806	0.07256										
16	0.12586	0.11053	0.09873											
8	0.14935	0.12587												
4	0.16234													

(c) $\mathcal{P}^{\mathcal{C}}$ for Scene: [**Veach Bidir**] Algorithm: [**PSSMLT**] Metric: [**HDR-VDP-2**] True GT: [**BDPT @ 524288 spp**].

MLT	2	4	8	16	32	64	128	256	512	1024	2048	4096	8192	16384
GT (BDPT)														
262144	-0.00012	-0.00008	-0.00000	0.00092	0.00245	0.00539	0.00845	0.00928	0.00793	0.00529	0.00541	0.00324	0.00251	0.00442
131072	-0.00004	0.00009	0.00005	0.00081	0.00048	-0.00032	0.00068	0.00197	0.00153	0.00056	0.00257	0.00233	0.00197	0.00278
65536	0.00041	0.00010	0.00023	0.00184	0.00577	0.01108	0.01711	0.02196	0.02389	0.02401	0.02680	0.02262	0.00762	0.00209
32768	0.00058	0.00065	0.00063	0.00325	0.01129	0.02738	0.04538	0.05766	0.06022	0.05468	0.04883	0.02520	0.00441	0.00023
16384	0.00031	0.00070	0.00088	0.00274	0.00647	0.01357	0.02432	0.02854	0.02730	0.02149	0.01443	0.01088	-0.00075	
8192	0.00143	0.00116	0.00173	0.00419	0.01343	0.02775	0.05333	0.07146	0.07547	0.07364	0.05312	0.02961		
4096	0.00314	0.00264	0.00206	0.00306	0.00436	0.00759	0.00951	0.01015	0.01114	0.00756	0.00392			
2048	0.00915	0.00693	0.00585	0.00516	0.00413	0.00440	0.00305	0.00294	0.00076	-0.00228				
1024	0.01283	0.01078	0.00904	0.00694	0.00586	0.00359	0.00086	-0.00054	0.00027					
512	0.02442	0.02243	0.01915	0.01710	0.02061	0.02103	0.02492	0.02534						
256	0.04433	0.03760	0.03396	0.03515	0.03688	0.03516	0.04001							
128	0.06244	0.05922	0.05101	0.05161	0.05871	0.05929								
64	0.09122	0.08508	0.07209	0.07066	0.07230									
32	0.12237	0.11263	0.09846	0.09067										
16	0.15579	0.14202	0.12290											
8	0.18858	0.16875												
4	0.21624													

(d) $\mathcal{P}^{\mathcal{C}}$ for Scene: [**Veach Bidir**] Algorithm: [**MLT**] Metric: [**HDR-VDP-2**] True GT: [**BDPT @ 524288 spp**].

M-MLT	2	4	8	16	32	64	128	256	512	1024	2048	4096	8192	16384
GT (BDPT)														
262144	0.00022	0.00020	0.00020	0.00062	0.00060	0.00034	0.00002	-0.00012	-0.00011	0.00028	-0.00085	0.00200	0.00057	0.00126
131072	0.00035	-0.00002	0.00015	0.00033	0.00015	0.00023	0.00009	-0.00010	-0.00053	0.00057	-0.00094	0.00315	0.00181	0.00166
65536	0.00037	0.00026	0.00033	0.00109	0.00114	0.00074	0.00017	0.00012	0.00228	-0.00043	0.00465	0.00045	-0.00050	-0.00022
32768	0.00094	0.00068	0.00037	0.00071	0.00025	0.00034	0.00030	0.00054	0.00391	-0.00124	0.01227	-0.00097	-0.00272	-0.00058
16384	0.00180	0.00086	0.00117	0.00139	0.00141	0.00093	0.00045	-0.00031	-0.00344	-0.00117	-0.00624	-0.00413	-0.00888	
8192	0.00329	0.00260	0.00227	0.00117	0.00082	0.00074	0.00139	0.00045	0.00047	-0.01268	-0.00871	-0.02304		
4096	0.00382	0.00385	0.00299	0.00238	0.00286	0.00194	0.00183	0.00005	-0.00040	-0.01120	-0.00417			
2048	0.00696	0.00677	0.00661	0.00348	0.00378	0.00169	0.00166	0.00282	0.00518	-0.01203				
1024	0.01519	0.01229	0.01044	0.01084	0.00897	0.00638	0.00519	0.00300	0.00315					
512	0.02493	0.02212	0.01974	0.02093	0.01912	0.01577	0.01007	0.00603						
256	0.04466	0.03982	0.03348	0.03436	0.03062	0.02524	0.01980							
128	0.06635	0.06039	0.05257	0.04696	0.04497	0.03613								
64	0.09201	0.08415	0.07248	0.06969	0.06108									
32	0.12401	0.11013	0.09574	0.08874										
16	0.15693	0.13914	0.12026											
8	0.18603	0.16468												
4	0.21613													

(e) $\mathcal{P}^{\mathcal{C}}$ for Scene: [**Veach Bidir**] Algorithm: [**M-MLT**] Metric: [**HDR-VDP-2**] True GT: [**BDPT @ 524288 spp**].

ERPT	2	4	8	16	32	64	128	256	512	1024	2048	4096	8192	16384	32768	65536	131072
GT (BDPT)																	
262144	-0.00012	-0.00016	0.00020	-0.00005	0.00101	0.00026	-0.00019	-0.00076	0.00010	0.00002	0.00123	0.00049	0.00019	-0.00066	0.00007	-0.00006	-0.00030
131072	-0.00001	-0.00002	0.00012	0.00007	0.00063	0.00063	-0.00016	0.00007	-0.00076	0.00057	0.00501	-0.00002	0.00063	-0.00004	-0.00007	-0.00009	
65536	-0.00003	0.00018	0.00042	0.00044	0.00048	0.00057	0.00007	-0.00015	-0.00033	0.00026	0.00390	-0.00016	0.00013	-0.00006	0.00048		
32768	-0.00030	0.00039	0.00053	0.00032	0.00095	0.00011	0.00026	0.00040	-0.00123	0.00019	0.00834	0.00086	-0.00034	-0.00062			
16384	0.00014	-0.00010	0.00062	0.00062	0.00076	-0.00153	-0.00002	-0.00203	-0.00369	0.00363	0.00336	-0.00047	-0.00109				
8192	0.00059	0.00064	0.00104	0.00046	0.00268	-0.00398	0.00153	-0.00400	-0.00399	0.00084	0.00735	-0.00082					
4096	0.00106	0.00028	0.00120	0.00103	0.00218	-0.00387	0.00146	-0.00554	-0.00725	-0.00053	-0.00105						
2048	0.00060	0.00021	0.00069	0.00121	0.00262	-0.00485	0.00188	-0.00571	-0.01112	-0.00222							
1024	0.00158	0.00179	0.00244	0.00232	0.00685	-0.00600	0.00518	-0.00172	-0.00551								
512	0.00883	0.00994	0.01104	0.01049	0.01293	-0.00172	0.00947	0.00005									
256	0.01755	0.01736	0.02111	0.01905	0.02266	0.00802	0.01503										
128	0.02486	0.02381	0.02592	0.02132	0.01476	0.00766											
64	0.03325	0.03539	0.03429	0.03247	0.03389												
32	0.02947	0.03150	0.03247	0.02621													
16	0.01943	0.02610	0.02569														
8	-0.00354	-0.00160															
4	-0.03852																

(f) $\mathcal{P}^{\mathcal{C}}$ for Scene: [**Veach Bidir**] Algorithm: [**ERPT**] Metric: [**HDR-VDP-2**] True GT: [**BDPT @ 524288 spp**].

M-ERPT	2	4	8	16	32	64	128	256	512	1024	2048	4096	8192	16384	32768	65536	131072
GT (BDPT)																	
262144	0.00001	0.00008	0.00005	-0.00003	-0.00004	0.00013	0.00006	0.00030	-0.00036	0.00003	0.00007	0.00004	0.00011	0.00009	0.00011	0.00013	-0.00004
131072	0.00007	0.00032	0.00016	0.00010	-0.00014	-0.00016	0.00046	0.00009	-0.00036	0.00000	0.00009	-0.00000	-0.00014	-0.00024	0.00004	0.00017	
65536	0.00021	0.00038	0.00014	-0.00049	-0.00000	0.00118	0.00075	-0.00110	0.00036	0.00037	0.00004	0.00038	0.00013	0.00023	0.00018		
32768	0.00018	0.00046	0.00043	-0.00003	-0.00005	0.00219	-0.00045	-0.00022	-0.00218	0.00033	0.00018	-0.00021	0.00004	0.00010			
16384	0.00091	0.00051	-0.00038	-0.00046	-0.00160	-0.00243	-0.00278	-0.00173	-0.00229	-0.00084	-0.00169	-0.00003	-0.00057				
8192	0.00086	0.00167	-0.00513	-0.00628	-0.00388	-0.00344	-0.00631	-0.00448	-0.00541	-0.00328	-0.00428	-0.00178					
4096	0.00154	0.00196	-0.00447	-0.00305	-0.00499	-0.00272	-0.00451	-0.00374	-0.00536	-0.00531	-0.00417						
2048	0.00241	0.00188	-0.00557	-0.00449	-0.00726	-0.00473	-0.00452	-0.00565	-0.00828	-0.00905							
1024	0.00335	0.00257	-0.00375	-0.00332	-0.00380	-0.00045	-0.00325	-0.00469	-0.00689								
512	0.01267	0.01329	-0.00401	0.00038	-0.00105	0.00152	0.00159	-0.00166									
256	0.02081	0.01785	0.00133	0.00598	0.00241	0.00552	0.00667										
128	0.02610	0.02049	0.00034	0.00454	0.00093	0.00485											
64	0.03561	0.03023	0.02022	0.02395	0.01863												
32	0.03026	0.03086	0.01828	0.01907													
16	0.02696	0.02308	0.00956														
8	0.00138	0.00389															
4	-0.02948																

(g) $\mathcal{P}^{\mathcal{C}}$ for Scene: [**Veach Bidir**] Algorithm: [**M-ERPT**] Metric: [**HDR-VDP-2**] True GT: [**BDPT @ 524288 spp**].

Table 53

12.4 Veach Door

PT	2	4	8	16	32	64	128	256	512	1024	2048	4096	8192	16384	32768	65536	131072
GT (BDPT)																	
262144	0.00129	0.00124	0.00112	0.00099	0.00123	0.00073	0.00115	0.00075	0.00040	0.00051	0.00093	0.00048	0.00019	0.00001	-0.00564	-0.02362	-0.04776
131072	0.00375	0.00392	0.00357	0.00290	0.00337	0.00275	0.00326	0.00278	0.00343	0.00201	0.00216	0.00173	0.00143	0.00119	-0.00149	-0.00535	
65536	0.00871	0.00857	0.00842	0.00721	0.00703	0.00728	0.00693	0.00562	0.00654	0.00674	0.00535	0.00475	0.00373	0.00232	-0.00321		
32768	0.02029	0.01887	0.01916	0.01731	0.01666	0.01637	0.01564	0.01457	0.01484	0.01190	0.01112	0.00944	0.00808	0.00569			
16384	0.03646	0.03715	0.03513	0.03438	0.03102	0.03025	0.02802	0.02961	0.02680	0.02494	0.02124	0.01816	0.01426				
8192	0.06349	0.06389	0.06053	0.05898	0.05620	0.05092	0.04990	0.04874	0.04774	0.04183	0.03434	0.03049					
4096	0.10298	0.10468	0.09761	0.09598	0.09073	0.08594	0.08150	0.08153	0.07590	0.06856	0.05649						
2048	0.14850	0.14878	0.14220	0.13590	0.13142	0.12393	0.11341	0.11590	0.11097	0.09608							
1024	0.19884	0.19639	0.19241	0.18375	0.17267	0.16481	0.15331	0.15163	0.14239								
512	0.24520	0.24202	0.23336	0.21980	0.21010	0.20211	0.18527	0.17977									
256	0.29081	0.28885	0.27567	0.26124	0.24725	0.23588	0.21565										
128	0.33259	0.33027	0.31211	0.29293	0.27869	0.26333											
64	0.36919	0.36492	0.34061	0.32451	0.29877												
32	0.40528	0.39645	0.36735	0.33014													
16	0.41913	0.40951	0.38004														
8	0.43458	0.41362															
4	0.41851																

(a) $\mathcal{P}^{\mathcal{C}}$ for Scene: [**Veach Door**] Algorithm: [**PT**] Metric: [**HDR-VDP-2**] True GT: [**BDPT @ 524288 spp**].

BDPT	2	4	8	16	32	64	128	256	512	1024	2048	4096	8192	16384	32768	65536	131072
GT (BDPT)																	
262144	0.00111	0.00115	0.00132	0.00130	0.00076	0.00097	0.00102	0.00085	0.00060	0.00048	0.00036	-0.00479	-0.01450	-0.04925	-0.06422	-0.08349	-0.05729
131072	0.00350	0.00294	0.00279	0.00336	0.00290	0.00257	0.00164	0.00208	0.00165	0.00208	0.00018	0.00011	-0.00395	-0.01789	-0.01939	-0.03267	
65536	0.00874	0.00794	0.00803	0.00710	0.00655	0.00588	0.00569	0.00468	0.00378	0.00477	0.00142	0.00006	-0.00502	-0.01654	-0.02428		
32768	0.01852	0.01887	0.01911	0.01680	0.01574	0.01402	0.01176	0.00940	0.00985	0.00778	0.00359	-0.00013	-0.00449	-0.02015			
16384	0.03652	0.03426	0.03404	0.03148	0.02927	0.02532	0.02326	0.01991	0.01660	0.01318	0.00552	0.00110	-0.00953				
8192	0.06540	0.06194	0.06095	0.05281	0.04799	0.04467	0.04292	0.03396	0.03052	0.02384	0.01047	-0.00155					
4096	0.10388	0.10177	0.09735	0.08636	0.08229	0.07177	0.06492	0.05680	0.04479	0.03285	0.00331						
2048	0.14489	0.14127	0.13821	0.12330	0.11433	0.10149	0.09042	0.07567	0.06352	0.04371							
1024	0.19892	0.19137	0.18192	0.16508	0.15485	0.13377	0.11808	0.09389	0.07001								
512	0.24384	0.23650	0.22450	0.19910	0.18486	0.16109	0.13637	0.10673									
256	0.28563	0.27521	0.26090	0.23029	0.21274	0.18215	0.15487										
128	0.31235	0.31102	0.29022	0.25342	0.23359	0.19044											
64	0.35440	0.33933	0.31659	0.27080	0.24064												
32	0.36430	0.36215	0.32956	0.28225													
16	0.35474	0.35823	0.33334														
8	0.37107	0.35280															
4	0.39498																

(b) $\mathcal{P}^{\mathcal{C}}$ for Scene: [**Veach Door**] Algorithm: [**BDPT**] Metric: [**HDR-VDP-2**] True GT: [**BDPT @ 524288 spp**].

PSSMLT	2	4	8	16	32	64	128	256	512	1024	2048	4096	8192	16384
GT (BDPT)														
262144	0.00068	0.00111	0.00130	0.00056	0.00102	0.00114	0.00091	0.00087	0.00063	-0.00030	-0.00370	-0.02236	-0.05149	-0.00243
131072	0.00254	0.00266	0.00302	0.00189	0.00250	0.00281	0.00220	0.00234	0.00116	0.00026	-0.00111	-0.00408	-0.01668	0.00261
65536	0.00766	0.00799	0.00712	0.00688	0.00599	0.00594	0.00493	0.00425	0.00307	0.00129	-0.00209	-0.00589	-0.01934	0.00396
32768	0.01624	0.01559	0.01692	0.01721	0.01427	0.01221	0.01114	0.00997	0.00855	0.00319	-0.00190	-0.00984	-0.02617	0.01696
16384	0.03169	0.02861	0.03089	0.02771	0.02476	0.02403	0.02065	0.01764	0.01500	0.00616	-0.00274	-0.01563	-0.03760	
8192	0.05793	0.05531	0.05100	0.04871	0.04410	0.03966	0.03334	0.02995	0.02360	0.01115	-0.00708	-0.03346		
4096	0.09017	0.09146	0.08515	0.07915	0.07130	0.06419	0.05584	0.04830	0.03262	0.00557	-0.03073			
2048	0.12787	0.12098	0.11072	0.10330	0.09302	0.08861	0.07551	0.06024	0.03992	0.00310				
1024	0.18024	0.17134	0.15936	0.14700	0.12832	0.10995	0.09021	0.06953	0.03102					
512	0.21849	0.20496	0.18698	0.17626	0.14878	0.13138	0.10225	0.06865						
256	0.24936	0.22973	0.20491	0.19395	0.16575	0.13799	0.10190							
128	0.27526	0.24815	0.22462	0.20755	0.17480	0.13041								
64	0.32743	0.29480	0.25679	0.22652	0.17899									
32	0.33295	0.30138	0.25785	0.21568										
16	0.32676	0.28763	0.23896											
8	0.30938	0.27261												
4	0.29722													

(c) $\mathcal{P}^{\mathcal{C}}$ for Scene: [**Veach Door**] Algorithm: [**PSSMLT**] Metric: [**HDR-VDP-2**] True GT: [**BDPT @ 524288 spp**].

MLT	2	4	8	16	32	64	128	256	512	1024	2048	4096	8192	16384
GT (BDPT)														
262144	0.00132	0.00108	0.00088	0.00118	0.00116	0.00165	0.00118	0.00060	0.00126	0.00035	0.00046	-0.01491	-0.04929	-0.08106
131072	0.00398	0.00254	0.00262	0.00272	0.00233	0.00323	0.00259	0.00155	0.00072	0.00186	-0.00118	-0.00469	-0.00750	-0.02471
65536	0.00783	0.00806	0.00731	0.00736	0.00730	0.00526	0.00534	0.00372	0.00385	0.00242	0.00294	-0.00278	-0.00893	-0.02610
32768	0.01778	0.01605	0.01479	0.01479	0.01486	0.01360	0.01072	0.00919	0.00837	0.00457	0.00147	-0.00949	-0.01608	-0.03482
16384	0.03535	0.02907	0.02740	0.02662	0.02447	0.02252	0.01838	0.01303	0.01420	0.00445	0.00060	-0.02022	-0.03101	
8192	0.05726	0.05327	0.04986	0.04466	0.04350	0.03868	0.03336	0.02283	0.01701	0.00702	0.00319	-0.03413		
4096	0.09589	0.08656	0.08001	0.07187	0.06778	0.05873	0.04868	0.03520	0.02494	-0.00108	-0.00325			
2048	0.13503	0.12316	0.11049	0.09601	0.08829	0.07345	0.06055	0.03865	0.02541	-0.01145				
1024	0.17990	0.16144	0.14279	0.12685	0.11334	0.09554	0.06744	0.03694	0.00924					
512	0.21500	0.19275	0.16890	0.15078	0.12823	0.10788	0.07429	0.02329						
256	0.25273	0.22544	0.19358	0.16836	0.14267	0.11112	0.06551							
128	0.28735	0.25490	0.19633	0.18357	0.15155	0.10108								
64	0.30744	0.27394	0.20797	0.18281	0.13512									
32	0.32936	0.28393	0.20464	0.17445										
16	0.32969	0.26883	0.18666											
8	0.31859	0.26094												
4	0.31596													

(d) $\mathcal{P}^{\mathcal{C}}$ for Scene: [**Veach Door**] Algorithm: [**MLT**] Metric: [**HDR-VDP-2**] True GT: [**BDPT @ 524288 spp**].

M-MLT	2	4	8	16	32	64	128	256	512	1024	2048	4096	8192	16384
GT (BDPT)														
262144	0.00170	0.00142	0.00034	0.00136	0.00088	0.00122	0.00142	0.00005	0.00061	-0.00110	-0.00044	-0.02645	-0.02638	-0.02026
131072	0.00427	0.00320	0.00218	0.00254	0.00237	0.00268	0.00278	-0.00083	0.00099	0.00159	-0.00133	-0.00415	-0.00889	-0.00201
65536	0.00865	0.00755	0.00732	0.00793	0.00665	0.00512	0.00512	0.00520	0.00431	0.00615	0.00269	-0.00403	-0.00616	0.00362
32768	0.01965	0.01808	0.01743	0.01505	0.01220	0.01075	0.01012	0.01345	0.01082	0.00967	0.00436	-0.00827	-0.01422	-0.00393
16384	0.03163	0.03221	0.02877	0.02806	0.02378	0.02152	0.02028	0.02025	0.01478	0.02090	0.01534	-0.01907	-0.02482	
8192	0.05517	0.05289	0.04831	0.04211	0.04162	0.03407	0.03157	0.04374	0.03237	0.02224	0.01990	-0.03848		
4096	0.09197	0.08996	0.07517	0.06513	0.06567	0.05574	0.04875	0.05450	0.04452	0.02767	0.01758			
2048	0.12359	0.12227	0.10686	0.10137	0.08780	0.07603	0.06193	0.06663	0.05295	0.01838				
1024	0.16691	0.15969	0.13598	0.12798	0.11364	0.09251	0.07421	0.06835	0.03871					
512	0.20126	0.19459	0.16958	0.15601	0.13064	0.10395	0.07342	0.05783						
256	0.22869	0.22237	0.19024	0.17717	0.14272	0.10645	0.06301							
128	0.25244	0.24278	0.19443	0.19437	0.14765	0.09005								
64	0.28602	0.26365	0.21995	0.18482	0.12379									
32	0.28104	0.26053	0.21495	0.17642										
16	0.28202	0.26446	0.19289											
8	0.27919	0.25346												
4	0.25925													

(e) $\mathcal{P}^{\mathcal{C}}$ for Scene: [**Veach Door**] Algorithm: [**M-MLT**] Metric: [**HDR-VDP-2**] True GT: [**BDPT @ 524288 spp**].

ERPT	2	4	8	16	32	64	128	256	512	1024	2048	4096	8192	16384	32768	65536	131072
GT (BDPT)																	
262144	0.00104	0.00076	0.00101	0.00091	0.00058	0.00150	0.00126	0.00037	0.00043	0.00021	0.00040	0.00125	0.00119	0.00132	0.00084	0.00054	0.00087
131072	0.00210	0.00246	0.00313	0.00191	0.00268	0.00188	0.00188	0.00182	0.00211	0.00184	0.00062	0.00202	0.00203	0.00225	0.00227	0.00216	
65536	0.00485	0.00753	0.00487	0.00473	0.00474	0.00566	0.00459	0.00395	0.00400	0.00555	0.00380	0.00570	0.00592	0.00461	0.00676		
32768	0.00872	0.01269	0.01092	0.00967	0.00982	0.01143	0.01023	0.00918	0.00927	0.00921	0.00959	0.00971	0.00809	0.00792			
16384	0.01616	0.01935	0.01873	0.01791	0.01757	0.01850	0.01791	0.01697	0.01818	0.01527	0.01927	0.01862	0.01834				
8192	0.02698	0.03472	0.02895	0.02900	0.02830	0.02933	0.02789	0.02902	0.03031	0.02950	0.03066	0.03103					
4096	0.03780	0.04606	0.04178	0.04590	0.04130	0.04512	0.04118	0.03976	0.04356	0.04259	0.04861						
2048	0.05309	0.06222	0.05854	0.05411	0.05321	0.05841	0.05441	0.05438	0.05320	0.05707							
1024	0.06345	0.07037	0.06696	0.06154	0.05364	0.06256	0.05465	0.06088	0.05849								
512	0.06485	0.07400	0.06212	0.06141	0.05954	0.06078	0.05634	0.05539									
256	0.05187	0.06481	0.04861	0.04539	0.04125	0.04402	0.03603										
128	0.02076	0.02833	0.00992	0.00804	0.00404	0.00353											
64	-0.01823	-0.00739	-0.02678	-0.03170	-0.03489												
32	-0.06405	-0.05702	-0.07391	-0.07994													
16	-0.11210	-0.10649	-0.12344														
8	-0.16033	-0.15293															
4	-0.20728																

(f) $\mathcal{P}^{\mathcal{C}}$ for Scene: [**Veach Door**] Algorithm: [**ERPT**] Metric: [**HDR-VDP-2**] True GT: [**BDPT @ 524288 spp**].

M-ERPT	2	4	8	16	32	64	128	256	512	1024	2048	4096	8192	16384	32768	65536	131072
GT (BDPT)																	
262144	0.00195	0.00040	0.00095	0.00119	0.00095	0.00095	0.00059	0.00067	0.00054	0.00107	0.00143	0.00070	0.00178	0.00032	0.00079	-0.00023	0.00092
131072	0.00174	0.00160	0.00164	0.00225	0.00235	0.00215	0.00185	0.00154	0.00149	0.00184	0.00239	0.00230	0.00166	0.00154	0.00204	0.00186	
65536	0.00702	0.00413	0.00385	0.00469	0.00453	0.00412	0.00407	0.00351	0.00351	0.00409	0.00432	0.00617	0.00400	0.00476	0.00485		
32768	0.00785	0.00950	0.01018	0.00823	0.00937	0.00962	0.00871	0.01013	0.01000	0.00938	0.01023	0.01095	0.00979	0.00982			
16384	0.02324	0.01814	0.01649	0.01746	0.01787	0.01720	0.01766	0.01923	0.01840	0.01582	0.01685	0.01874	0.01850				
8192	0.02874	0.03604	0.03008	0.02762	0.03072	0.03065	0.02939	0.03130	0.02916	0.02712	0.02929	0.03151					
4096	0.05183	0.04451	0.04362	0.04799	0.04368	0.04234	0.04140	0.04506	0.04330	0.04249	0.04279						
2048	0.07020	0.06211	0.05550	0.06285	0.05934	0.05722	0.05206	0.06156	0.05873	0.05583							
1024	0.08487	0.07699	0.06402	0.07050	0.06271	0.06055	0.06016	0.06801	0.06639								
512	0.09076	0.07504	0.06547	0.07035	0.06283	0.06188	0.06137	0.06693									
256	0.07791	0.06328	0.05088	0.05419	0.04596	0.04637	0.04464										
128	0.04234	0.02982	0.01280	0.01617	0.00785	0.00770											
64	0.00623	-0.00914	-0.02270	-0.01829	-0.02688												
32	-0.03740	-0.05696	-0.06855	-0.06129													
16	-0.08241	-0.10294	-0.11452														
8	-0.13314	-0.15025															
4	-0.17915																

(g) $\mathcal{P}^{\mathcal{C}}$ for Scene: [**Veach Door**] Algorithm: [**M-ERPT**] Metric: [**HDR-VDP-2**] True GT: [**BDPT @ 524288 spp**].

Table 54

12.5 Sponza

PT	2	4	8	16	32	64	128	256	512	1024	2048	4096	8192
GT (PT)													
16384	0.01218	0.00977	0.00998	0.00706	0.00691	0.00768	0.00850	0.00635	0.00392	0.00071	-0.00070	-0.00512	-0.00909
8192	0.03137	0.02453	0.02407	0.02127	0.02000	0.01899	0.01705	0.01307	0.00949	0.00666	-0.00178	-0.01150	
4096	0.05730	0.05013	0.04671	0.04455	0.04015	0.03728	0.03475	0.02830	0.01991	0.00902	-0.00507		
2048	0.08311	0.07622	0.07080	0.06180	0.05793	0.05450	0.04971	0.03960	0.02140	0.00568			
1024	0.12034	0.10688	0.10060	0.09174	0.07946	0.07523	0.06219	0.04855	0.02347				
512	0.14982	0.13734	0.12889	0.11673	0.09966	0.09436	0.07107	0.05193					
256	0.18786	0.17113	0.16248	0.14281	0.12149	0.10926	0.07871						
128	0.22115	0.20461	0.19169	0.16668	0.13938	0.11718							
64	0.25050	0.23228	0.21539	0.18749	0.14716								
32	0.28157	0.25652	0.23530	0.19649									
16	0.30446	0.27403	0.24453										
8	0.31378	0.27895											
4	0.31614												

(a) $\mathcal{P}^{\mathcal{C}}$ for Scene: [**Sponza**] Algorithm: [**PT**] Metric: [**HDR-VDP-2**] True GT: [**PT @ 32768 spp**].

BDPT	2	4	8	16	32	64	128	256	512	1024	2048	4096	8192
GT (PT)													
16384	0.01045	0.00928	0.00989	0.01026	0.00612	0.00505	0.00622	0.00530	0.00353	0.00075	0.00173	-0.00231	-0.00982
8192	0.02654	0.02394	0.02291	0.02305	0.02002	0.01842	0.01470	0.01293	0.00883	0.00391	0.00218	-0.00811	
4096	0.05653	0.05070	0.05447	0.04747	0.04088	0.03792	0.03163	0.02299	0.02055	0.00702	0.00153		
2048	0.08340	0.07765	0.07589	0.06895	0.06455	0.05314	0.04735	0.03498	0.02599	0.00719			
1024	0.12236	0.10996	0.10813	0.08926	0.09003	0.07563	0.06235	0.04425	0.02645				
512	0.15495	0.13920	0.13698	0.11884	0.11250	0.09024	0.07538	0.04990					
256	0.18913	0.17500	0.17176	0.14378	0.13666	0.10862	0.08495						
128	0.22442	0.20707	0.20204	0.17236	0.15817	0.12100							
64	0.25361	0.23714	0.23145	0.19373	0.17075								
32	0.28339	0.26156	0.24956	0.20609									
16	0.30732	0.28115	0.26512										
8	0.32363	0.28959											
4	0.32732												

(b) $\mathcal{P}^{\mathcal{C}}$ for Scene: [**Sponza**] Algorithm: [**BDPT**] Metric: [**HDR-VDP-2**] True GT: [**PT @ 32768 spp**].

PSSMLT	2	4	8	16	32	64	128	256	512	1024	2048	4096	8192
GT (PT)													
16384	0.00962	0.00708	0.00964	0.01059	0.01034	0.00660	0.00534	0.00398	0.00201	0.00252	0.00004	0.00253	0.00299
8192	0.03099	0.02669	0.02422	0.02350	0.02093	0.02073	0.01583	0.01164	0.00648	0.00356	0.00207	0.00496	
4096	0.06167	0.05330	0.05015	0.04363	0.04027	0.03392	0.02857	0.02608	0.01450	0.00997	0.00628		
2048	0.08292	0.07151	0.07310	0.06209	0.05528	0.05081	0.04080	0.03413	0.01953	0.01846			
1024	0.11666	0.10625	0.09855	0.08649	0.07937	0.07080	0.05411	0.04231	0.02855				
512	0.14763	0.13676	0.12649	0.11018	0.10009	0.08893	0.06839	0.05496					
256	0.18132	0.16960	0.15589	0.13306	0.11962	0.10791	0.07964						
128	0.21478	0.20009	0.18519	0.16015	0.14073	0.12049							
64	0.24271	0.22665	0.19932	0.17758	0.15200								
32	0.26990	0.24958	0.22650	0.19206									
16	0.28379	0.26083	0.23354										
8	0.28732	0.26071											
4	0.27312												

(c) $\mathcal{P}^{\mathcal{C}}$ for Scene: [**Sponza**] Algorithm: [**PSSMLT**] Metric: [**HDR-VDP-2**] True GT: [**PT @ 32768 spp**].

MLT	2	4	8	16	32	64	128	256	512	1024	2048	4096	8192
GT (PT)													
16384	0.00979	0.00954	0.00754	0.00732	0.00661	0.00515	0.00411	0.00214	0.00243	0.00117	0.00201	0.00275	0.00123
8192	0.02314	0.02286	0.02092	0.01947	0.01544	0.01227	0.00581	0.00540	0.00284	0.00176	0.00102	0.00299	
4096	0.04427	0.04175	0.03566	0.03370	0.02810	0.02251	0.01625	0.01251	0.00822	0.00947	0.00735		
2048	0.06896	0.06729	0.05687	0.05232	0.03903	0.02829	0.02271	0.01783	0.01406	0.01365			
1024	0.09569	0.08783	0.07189	0.07091	0.05622	0.04444	0.03192	0.02718	0.02135				
512	0.12297	0.11305	0.09554	0.08475	0.06655	0.05378	0.03884	0.03009					
256	0.15192	0.13684	0.11691	0.10327	0.08259	0.05881	0.03814						
128	0.18011	0.16401	0.13617	0.12010	0.08781	0.05574							
64	0.20820	0.18514	0.14781	0.12350	0.08609								
32	0.23015	0.20080	0.15978	0.12388									
16	0.23301	0.20741	0.15581										
8	0.23643	0.20349											
4	0.21886												

(d) $\mathcal{P}^{\mathcal{C}}$ for Scene: [**Sponza**] Algorithm: [**MLT**] Metric: [**HDR-VDP-2**] True GT: [**PT @ 32768 spp**].

M-MLT	2	4	8	16	32	64	128	256	512	1024	2048	4096	8192
GT (PT)													
16384	0.00834	0.00504	0.00704	0.00789	0.00562	0.00319	0.00199	0.00224	0.00238	0.00119	-0.00115	0.00531	0.00164
8192	0.02389	0.01986	0.02142	0.02047	0.01243	0.01154	0.00602	0.00229	0.00237	0.00292	0.00114	0.00260	
4096	0.04745	0.04233	0.03504	0.03863	0.02799	0.02275	0.01305	0.00970	0.01102	0.00705	0.00411		
2048	0.07359	0.06287	0.05866	0.05168	0.04022	0.03136	0.01817	0.01589	0.01740	0.01063			
1024	0.09704	0.08275	0.07746	0.06631	0.05473	0.04402	0.02827	0.02393	0.02310				
512	0.12340	0.10478	0.09536	0.08983	0.06683	0.05484	0.03641	0.02832					
256	0.15256	0.13116	0.11877	0.11477	0.07931	0.06114	0.03803						
128	0.18080	0.15641	0.13801	0.12954	0.08231	0.05848							
64	0.20643	0.17696	0.15057	0.13705	0.07438								
32	0.22568	0.19088	0.15597	0.13167									
16	0.24094	0.20011	0.15194										
8	0.24174	0.19191											
4	0.23430												

(e) $\mathcal{P}^{\mathcal{C}}$ for Scene: [**Sponza**] Algorithm: [**M-MLT**] Metric: [**HDR-VDP-2**] True GT: [**PT @ 32768 spp**].

ERPT	2	4	8	16	32	64	128	256	512	1024	2048	4096	8192
GT (PT)													
16384	0.00439	0.00276	0.00221	0.00378	0.00147	0.00303	0.00207	0.00076	0.00180	0.00139	0.00400	0.00227	0.00271
8192	0.00855	0.00935	0.00775	0.00467	0.00642	0.00741	0.00654	0.00415	0.00574	0.00524	0.00782	0.00491	
4096	0.01215	0.01427	0.01370	0.01193	0.01106	0.01341	0.01122	0.00982	0.01083	0.01162	0.01416		
2048	0.02126	0.02233	0.01739	0.02132	0.01628	0.01958	0.02035	0.01812	0.01908	0.01744			
1024	0.03148	0.03384	0.03119	0.02745	0.02390	0.02911	0.02799	0.02568	0.02676				
512	0.03975	0.03923	0.03606	0.03447	0.02738	0.03391	0.03174	0.03256					
256	0.03775	0.03356	0.03435	0.03034	0.02582	0.03010	0.02845						
128	0.01708	0.01823	0.01305	0.00914	0.00476	0.00973							
64	-0.01997	-0.01663	-0.02400	-0.02673	-0.03265								
32	-0.06026	-0.05839	-0.06482	-0.06791									
16	-0.11322	-0.11067	-0.11758										
8	-0.15831	-0.15784											
4	-0.20048												

(f) $\mathcal{P}^{\mathcal{C}}$ for Scene: [**Sponza**] Algorithm: [**ERPT**] Metric: [**HDR-VDP-2**] True GT: [**PT @ 32768 spp**].

M-ERPT	2	4	8	16	32	64	128	256	512	1024	2048	4096	8192
GT (PT)													
16384	0.00217	0.00150	0.00439	0.00341	0.00187	0.00220	0.00200	0.00335	0.00242	0.00201	0.00160	0.00292	0.00458
8192	0.00608	0.00675	0.00582	0.00346	0.00638	0.00469	0.00645	0.00579	0.00447	0.00512	0.00603	0.00389	
4096	0.01464	0.01148	0.01591	0.01280	0.01461	0.01404	0.01229	0.01251	0.01245	0.01088	0.00925		
2048	0.02128	0.01946	0.02360	0.02454	0.02242	0.02069	0.02192	0.01969	0.01975	0.01909			
1024	0.03345	0.02968	0.03028	0.03257	0.03216	0.02837	0.02855	0.02682	0.02805				
512	0.04077	0.03625	0.03506	0.03810	0.03647	0.03452	0.03497	0.03529					
256	0.03759	0.03462	0.03542	0.03791	0.03411	0.03363	0.03311						
128	0.01992	0.01494	0.01547	0.01920	0.01418	0.01408							
64	-0.01471	-0.02283	-0.01977	-0.01696	-0.02197								
32	-0.05472	-0.06240	-0.06279	-0.05821									
16	-0.10644	-0.11423	-0.11204										
8	-0.15012	-0.15783											
4	-0.19203												

(g) $\mathcal{P}^{\mathcal{C}}$ for Scene: [**Sponza**] Algorithm: [**M-ERPT**] Metric: [**HDR-VDP-2**] True GT: [**PT @ 32768 spp**].

Table 55

13 SC-QI

13.1 Cornell Box

**PT**	2	4	8	16	32	64	128	256	512	1024	2048	4096	8192
**GT (PT)**													
16384	0.00001	0.00001	0.00001	0.00000	0.00000	0.00000	0.00000	0.00000	0.00000	0.00000	0.00000	-0.00000	-0.00000
8192	0.00002	0.00002	0.00001	0.00001	0.00001	0.00001	0.00000	0.00000	0.00000	0.00000	0.00000	-0.00000	
4096	0.00004	0.00004	0.00003	0.00002	0.00002	0.00001	0.00001	0.00000	0.00000	0.00000	-0.00000		
2048	0.00007	0.00006	0.00005	0.00004	0.00003	0.00002	0.00001	0.00001	0.00000	0.00000			
1024	0.00012	0.00009	0.00008	0.00006	0.00004	0.00003	0.00002	0.00001	0.00000				
512	0.00018	0.00014	0.00011	0.00008	0.00006	0.00004	0.00002	0.00001					
256	0.00026	0.00020	0.00015	0.00011	0.00007	0.00004	0.00001						
128	0.00035	0.00027	0.00020	0.00013	0.00008	0.00003							
64	0.00047	0.00035	0.00024	0.00015	0.00007								
32	0.00058	0.00041	0.00027	0.00013									
16	0.00068	0.00045	0.00025										
8	0.00074	0.00042											
4	0.00069												

(a) $\mathcal{P}^{\mathcal{C}}$ for Scene: [**Cornell Box**] Algorithm: [**PT**] Metric: [**SC-QI**] True GT: [**PT @ 32768 spp**].

**BDPT**	2	4	8	16	32	64	128	256	512	1024	2048	4096	8192
**GT (PT)**													
16384	0.00001	0.00000	0.00000	0.00000	0.00000	0.00000	0.00000	0.00000	0.00000	-0.00000	-0.00000	-0.00000	-0.00000
8192	0.00001	0.00001	0.00001	0.00001	0.00000	0.00000	0.00000	0.00000	0.00000	-0.00000	-0.00000	-0.00000	
4096	0.00003	0.00002	0.00002	0.00001	0.00001	0.00001	0.00000	0.00000	-0.00000	-0.00000	-0.00000		
2048	0.00005	0.00004	0.00003	0.00002	0.00001	0.00001	0.00000	0.00000	-0.00000	-0.00000			
1024	0.00008	0.00006	0.00004	0.00003	0.00002	0.00001	0.00000	-0.00000	-0.00001				
512	0.00011	0.00009	0.00006	0.00004	0.00002	0.00001	-0.00000	-0.00001					
256	0.00016	0.00012	0.00008	0.00005	0.00002	0.00000	-0.00001						
128	0.00022	0.00015	0.00009	0.00005	0.00001	-0.00002							
64	0.00027	0.00017	0.00009	0.00003	-0.00003								
32	0.00032	0.00017	0.00006	-0.00003									
16	0.00032	0.00013	-0.00004										
8	0.00024	-0.00003											
4	0.00002												

(b) $\mathcal{P}^{\mathcal{C}}$ for Scene: [**Cornell Box**] Algorithm: [**BDPT**] Metric: [**SC-QI**] True GT: [**PT @ 32768 spp**].

**PSSMLT**	2	4	8	16	32	64	128	256	512	1024	2048	4096	8192
**GT (PT)**													
16384	0.00000	0.00000	0.00000	0.00000	0.00000	0.00000	0.00000	0.00000	0.00000	0.00000	-0.00000	-0.00000	-0.00000
8192	0.00001	0.00001	0.00001	0.00001	0.00000	0.00000	0.00000	0.00000	0.00000	0.00000	-0.00000	-0.00000	
4096	0.00002	0.00002	0.00001	0.00001	0.00001	0.00001	0.00000	0.00000	0.00000	-0.00000	-0.00000		
2048	0.00004	0.00003	0.00002	0.00002	0.00001	0.00001	0.00000	0.00000	-0.00000	-0.00000			
1024	0.00006	0.00005	0.00004	0.00002	0.00002	0.00001	0.00000	-0.00000	-0.00000				
512	0.00009	0.00007	0.00005	0.00003	0.00002	0.00001	-0.00000	-0.00001					
256	0.00014	0.00009	0.00006	0.00004	0.00002	-0.00000	-0.00001						
128	0.00018	0.00012	0.00007	0.00003	0.00000	-0.00003							
64	0.00022	0.00014	0.00006	0.00001	-0.00004								
32	0.00026	0.00013	0.00002	-0.00006									
16	0.00024	0.00007	-0.00008										
8	0.00016	-0.00009											
4	-0.00009												

(c) $\mathcal{P}^{\mathcal{C}}$ for Scene: [**Cornell Box**] Algorithm: [**PSSMLT**] Metric: [**SC-QI**] True GT: [**PT @ 32768 spp**].

**MLT**	2	4	8	16	32	64	128	256	512	1024	2048	4096	8192
**GT (PT)**													
16384	0.00001	0.00001	0.00001	0.00000	0.00000	0.00000	0.00000	0.00000	0.00000	0.00000	0.00000	0.00000	0.00000
8192	0.00002	0.00001	0.00001	0.00001	0.00001	0.00001	0.00000	0.00000	0.00000	0.00000	0.00000	0.00000	
4096	0.00003	0.00003	0.00002	0.00002	0.00002	0.00001	0.00001	0.00001	0.00000	0.00000	0.00000		
2048	0.00006	0.00005	0.00004	0.00003	0.00003	0.00002	0.00001	0.00001	0.00000	0.00000			
1024	0.00009	0.00008	0.00007	0.00005	0.00004	0.00003	0.00002	0.00001	0.00000				
512	0.00015	0.00012	0.00010	0.00007	0.00005	0.00004	0.00002	0.00001					
256	0.00022	0.00018	0.00014	0.00010	0.00007	0.00004	0.00002						
128	0.00030	0.00024	0.00019	0.00013	0.00008	0.00004							
64	0.00042	0.00032	0.00024	0.00014	0.00008								
32	0.00054	0.00040	0.00028	0.00015									
16	0.00067	0.00047	0.00029										
8	0.00076	0.00048											
4	0.00077												

(d) $\mathcal{P}^{\mathcal{C}}$ for Scene: [**Cornell Box**] Algorithm: [**MLT**] Metric: [**SC-QI**] True GT: [**PT @ 32768 spp**].

**M-MLT**	2	4	8	16	32	64	128	256	512	1024	2048	4096	8192
**GT (PT)**													
16384	0.00001	0.00001	0.00000	0.00000	0.00000	0.00000	0.00000	0.00000	0.00000	0.00000	0.00000	0.00000	-0.00000
8192	0.00002	0.00002	0.00001	0.00001	0.00001	0.00001	0.00000	0.00000	0.00000	0.00000	0.00000	0.00000	
4096	0.00003	0.00003	0.00002	0.00002	0.00001	0.00001	0.00001	0.00001	0.00000	0.00000	0.00000		
2048	0.00006	0.00005	0.00004	0.00003	0.00002	0.00002	0.00001	0.00001	0.00000	0.00000			
1024	0.00009	0.00008	0.00006	0.00005	0.00004	0.00002	0.00002	0.00001	0.00000				
512	0.00015	0.00012	0.00009	0.00007	0.00005	0.00003	0.00002	0.00001					
256	0.00022	0.00018	0.00014	0.00010	0.00007	0.00004	0.00002						
128	0.00031	0.00024	0.00018	0.00012	0.00008	0.00004							
64	0.00042	0.00032	0.00023	0.00014	0.00007								
32	0.00053	0.00040	0.00026	0.00014									
16	0.00065	0.00046	0.00026										
8	0.00072	0.00045											
4	0.00073												

(e) $\mathcal{P}^{\mathcal{C}}$ for Scene: [**Cornell Box**] Algorithm: [**M-MLT**] Metric: [**SC-QI**] True GT: [**PT @ 32768 spp**].

**ERPT**	2	4	8	16	32	64	128	256	512	1024	2048	4096	8192
**GT (PT)**													
16384	0.00000	0.00000	0.00000	0.00000	0.00000	0.00000	0.00000	0.00000	0.00000	0.00000	0.00000	0.00000	0.00000
8192	0.00000	0.00000	0.00000	0.00000	0.00000	0.00000	0.00000	0.00000	0.00000	0.00000	0.00000	0.00000	
4096	0.00001	0.00001	0.00001	0.00001	0.00001	0.00001	0.00001	0.00001	0.00001	0.00001	0.00001		
2048	0.00001	0.00001	0.00001	0.00001	0.00001	0.00001	0.00001	0.00001	0.00001	0.00001			
1024	0.00002	0.00002	0.00002	0.00002	0.00002	0.00001	0.00001	0.00002	0.00001				
512	0.00002	0.00002	0.00002	0.00002	0.00002	0.00002	0.00002	0.00002					
256	0.00001	0.00001	0.00002	0.00002	0.00002	0.00001	0.00001						
128	-0.00000	-0.00000	-0.00000	-0.00000	-0.00000	-0.00000							
64	-0.00004	-0.00004	-0.00004	-0.00004	-0.00004								
32	-0.00013	-0.00013	-0.00013	-0.00012									
16	-0.00029	-0.00029	-0.00029										
8	-0.00058	-0.00058											
4	-0.00105												

(f) $\mathcal{P}^{\mathcal{C}}$ for Scene: [**Cornell Box**] Algorithm: [**ERPT**] Metric: [**SC-QI**] True GT: [**PT @ 32768 spp**].

**M-ERPT**	2	4	8	16	32	64	128	256	512	1024	2048	4096	8192
**GT (PT)**													
16384	0.00000	0.00000	0.00000	0.00000	0.00000	0.00000	0.00000	0.00000	0.00000	0.00000	0.00000	0.00000	0.00000
8192	0.00000	0.00000	0.00000	0.00000	0.00000	0.00000	0.00000	0.00000	0.00000	0.00000	0.00000	0.00000	
4096	0.00001	0.00001	0.00001	0.00001	0.00001	0.00001	0.00001	0.00001	0.00001	0.00001	0.00001		
2048	0.00001	0.00001	0.00001	0.00001	0.00001	0.00001	0.00001	0.00001	0.00001	0.00001			
1024	0.00002	0.00002	0.00002	0.00002	0.00002	0.00002	0.00002	0.00002	0.00002				
512	0.00002	0.00002	0.00002	0.00002	0.00002	0.00002	0.00002	0.00002					
256	0.00002	0.00002	0.00002	0.00002	0.00002	0.00002	0.00002						
128	0.00000	0.00000	0.00000	0.00000	0.00000	0.00000							
64	-0.00004	-0.00004	-0.00004	-0.00004	-0.00004								
32	-0.00012	-0.00012	-0.00012	-0.00012									
16	-0.00028	-0.00028	-0.00029										
8	-0.00058	-0.00057											
4	-0.00105												

(g) $\mathcal{P}^{\mathcal{C}}$ for Scene: [**Cornell Box**] Algorithm: [**M-ERPT**] Metric: [**SC-QI**] True GT: [**PT @ 32768 spp**].

Table 56

13.2 Torus

PT	2	4	8	16	32	64	128	256	512	1024	2048	4096	8192	16384	32768	65536	131072
GT (BDPT)																	
262144	0.00003	0.00003	0.00002	0.00002	0.00002	0.00002	0.00003	0.00003	0.00003	0.00003	0.00002	0.00002	0.00001	0.00001	0.00000	0.00000	-0.00000
131072	0.00007	0.00006	0.00005	0.00005	0.00005	0.00005	0.00006	0.00007	0.00007	0.00006	0.00005	0.00004	0.00003	0.00001	0.00001	-0.00000	
65536	0.00013	0.00010	0.00008	0.00008	0.00008	0.00009	0.00010	0.00012	0.00012	0.00010	0.00008	0.00006	0.00004	0.00002	0.00000		
32768	0.00020	0.00016	0.00013	0.00012	0.00013	0.00014	0.00016	0.00018	0.00018	0.00015	0.00012	0.00008	0.00004	0.00001			
16384	0.00028	0.00023	0.00018	0.00016	0.00018	0.00020	0.00022	0.00026	0.00025	0.00021	0.00017	0.00010	0.00004				
8192	0.00034	0.00027	0.00020	0.00018	0.00020	0.00023	0.00028	0.00031	0.00030	0.00025	0.00018	0.00008					
4096	0.00039	0.00029	0.00021	0.00019	0.00022	0.00025	0.00033	0.00036	0.00034	0.00027	0.00017						
2048	0.00041	0.00029	0.00017	0.00016	0.00019	0.00026	0.00032	0.00036	0.00035	0.00025							
1024	0.00041	0.00026	0.00013	0.00012	0.00016	0.00021	0.00029	0.00036	0.00032								
512	0.00038	0.00023	0.00008	0.00006	0.00011	0.00016	0.00025	0.00030									
256	0.00036	0.00021	0.00005	0.00004	0.00009	0.00013	0.00019										
128	0.00042	0.00027	0.00013	0.00011	0.00012	0.00015											
64	0.00049	0.00033	0.00016	0.00012	0.00013												
32	0.00060	0.00042	0.00024	0.00017													
16	0.00071	0.00049	0.00033														
8	0.00078	0.00049															
4	0.00079																

(a) $\mathcal{P}^{\mathcal{C}}$ for Scene: [**Torus**] Algorithm: [**PT**] Metric: [**SC-QI**] True GT: [**BDPT @ 524288 spp**].

BDPT	2	4	8	16	32	64	128	256	512	1024	2048	4096	8192	16384	32768	65536	131072
GT (BDPT)																	
262144	0.00003	0.00003	0.00002	0.00002	0.00002	0.00003	0.00003	0.00003	0.00003	0.00003	0.00002	0.00002	0.00001	0.00001	0.00001	0.00000	-0.00000
131072	0.00008	0.00007	0.00006	0.00005	0.00005	0.00006	0.00006	0.00006	0.00007	0.00006	0.00005	0.00004	0.00002	0.00002	0.00001	-0.00000	
65536	0.00013	0.00011	0.00009	0.00008	0.00009	0.00009	0.00011	0.00012	0.00012	0.00010	0.00008	0.00006	0.00004	0.00002	0.00000		
32768	0.00021	0.00018	0.00014	0.00013	0.00013	0.00014	0.00017	0.00018	0.00018	0.00016	0.00012	0.00008	0.00005	0.00002			
16384	0.00030	0.00025	0.00019	0.00018	0.00018	0.00020	0.00023	0.00026	0.00026	0.00022	0.00016	0.00009	0.00005				
8192	0.00037	0.00029	0.00022	0.00020	0.00022	0.00024	0.00028	0.00031	0.00030	0.00026	0.00018	0.00009					
4096	0.00044	0.00033	0.00024	0.00023	0.00024	0.00028	0.00032	0.00036	0.00035	0.00029	0.00017						
2048	0.00046	0.00033	0.00021	0.00020	0.00022	0.00027	0.00034	0.00037	0.00035	0.00025							
1024	0.00045	0.00031	0.00018	0.00018	0.00018	0.00024	0.00032	0.00036	0.00032								
512	0.00043	0.00027	0.00014	0.00012	0.00013	0.00019	0.00028	0.00031									
256	0.00041	0.00025	0.00012	0.00010	0.00012	0.00014	0.00024										
128	0.00046	0.00031	0.00016	0.00014	0.00014	0.00017											
64	0.00052	0.00037	0.00020	0.00017	0.00016												
32	0.00063	0.00047	0.00028	0.00025													
16	0.00077	0.00054	0.00032														
8	0.00083	0.00056															
4	0.00083																

(b) $\mathcal{P}^{\mathcal{C}}$ for Scene: [**Torus**] Algorithm: [**BDPT**] Metric: [**SC-QI**] True GT: [**BDPT @ 524288 spp**].

PSSMLT	2	4	8	16	32	64	128	256	512	1024	2048	4096	8192	16384
GT (BDPT)														
262144	0.00003	0.00002	0.00002	0.00002	0.00002	0.00002	0.00002	0.00001	0.00001	0.00001	0.00001	0.00000	0.00000	0.00000
131072	0.00007	0.00006	0.00005	0.00005	0.00005	0.00004	0.00004	0.00003	0.00002	0.00002	0.00001	0.00001	0.00000	-0.00000
65536	0.00011	0.00009	0.00008	0.00007	0.00007	0.00007	0.00006	0.00005	0.00004	0.00003	0.00002	0.00001	-0.00000	-0.00001
32768	0.00018	0.00014	0.00012	0.00012	0.00012	0.00011	0.00009	0.00007	0.00005	0.00003	0.00001	-0.00000	-0.00002	-0.00003
16384	0.00025	0.00019	0.00016	0.00015	0.00015	0.00013	0.00011	0.00007	0.00005	0.00002	-0.00001	-0.00004	-0.00007	
8192	0.00034	0.00027	0.00022	0.00020	0.00018	0.00015	0.00011	0.00007	0.00002	-0.00002	-0.00007	-0.00011		
4096	0.00042	0.00031	0.00025	0.00023	0.00019	0.00016	0.00011	0.00003	-0.00004	-0.00009	-0.00016			
2048	0.00045	0.00029	0.00022	0.00019	0.00017	0.00011	0.00002	-0.00007	-0.00016	-0.00024				
1024	0.00046	0.00027	0.00019	0.00016	0.00011	0.00004	-0.00007	-0.00017	-0.00030					
512	0.00040	0.00021	0.00011	0.00008	0.00002	-0.00008	-0.00020	-0.00033						
256	0.00033	0.00012	0.00004	-0.00000	-0.00007	-0.00017	-0.00029							
128	0.00032	0.00014	0.00002	0.00000	-0.00007	-0.00018								
64	0.00024	0.00008	-0.00002	-0.00005	-0.00016									
32	0.00025	0.00008	-0.00005	-0.00007										
16	0.00023	0.00006	-0.00012											
8	0.00021	0.00002												
4	0.00007													

(c) $\mathcal{P}^{\mathcal{C}}$ for Scene: [**Torus**] Algorithm: [**PSSMLT**] Metric: [**SC-QI**] True GT: [**BDPT @ 524288 spp**].

MLT	2	4	8	16	32	64	128	256	512	1024	2048	4096	8192	16384
GT (BDPT)														
262144	0.00003	0.00002	0.00002	0.00001	0.00001	0.00001	0.00000	0.00001	0.00000	0.00001	0.00000	0.00000	0.00000	0.00000
131072	0.00007	0.00004	0.00004	0.00002	0.00002	0.00002	0.00001	0.00001	0.00000	0.00001	0.00000	0.00000	-0.00000	-0.00000
65536	0.00012	0.00009	0.00008	0.00005	0.00003	0.00002	0.00001	0.00001	0.00000	0.00000	0.00000	-0.00000	-0.00001	-0.00001
32768	0.00020	0.00013	0.00011	0.00006	0.00004	0.00002	0.00001	-0.00000	-0.00001	-0.00001	-0.00002	-0.00002	-0.00003	-0.00004
16384	0.00029	0.00023	0.00017	0.00009	0.00005	0.00002	-0.00001	-0.00002	-0.00004	-0.00004	-0.00005	-0.00006	-0.00007	
8192	0.00044	0.00031	0.00021	0.00010	0.00004	0.00001	-0.00005	-0.00006	-0.00009	-0.00010	-0.00012	-0.00013		
4096	0.00054	0.00038	0.00025	0.00008	0.00001	-0.00004	-0.00012	-0.00016	-0.00020	-0.00021	-0.00023			
2048	0.00066	0.00046	0.00025	0.00005	-0.00008	-0.00016	-0.00024	-0.00030	-0.00034	-0.00037				
1024	0.00071	0.00045	0.00020	-0.00006	-0.00022	-0.00032	-0.00043	-0.00047	-0.00053					
512	0.00071	0.00041	0.00014	-0.00019	-0.00035	-0.00046	-0.00058	-0.00068						
256	0.00065	0.00036	0.00008	-0.00026	-0.00046	-0.00053	-0.00071							
128	0.00057	0.00030	0.00005	-0.00033	-0.00048	-0.00058								
64	0.00050	0.00023	0.00001	-0.00035	-0.00050									
32	0.00051	0.00016	0.00002	-0.00038										
16	0.00059	0.00019	0.00004											
8	0.00049	0.00010												
4	0.00051													

(d) $\mathcal{P}^{\mathcal{C}}$ for Scene: [**Torus**] Algorithm: [**MLT**] Metric: [**SC-QI**] True GT: [**BDPT @ 524288 spp**].

M-MLT	2	4	8	16	32	64	128	256	512	1024	2048	4096	8192	16384
GT (BDPT)														
262144	0.00002	0.00002	0.00001	0.00001	0.00001	0.00001	0.00000	0.00001	0.00001	0.00000	0.00000	0.00000	0.00000	-0.00000
131072	0.00005	0.00005	0.00004	0.00003	0.00003	0.00001	0.00001	0.00001	0.00001	-0.00000	-0.00000	-0.00000	-0.00000	-0.00001
65536	0.00010	0.00010	0.00006	0.00004	0.00005	0.00002	0.00001	0.00001	0.00000	-0.00000	-0.00001	-0.00001	-0.00001	-0.00002
32768	0.00015	0.00015	0.00009	0.00006	0.00006	0.00001	0.00000	0.00001	-0.00001	-0.00002	-0.00002	-0.00003	-0.00004	-0.00004
16384	0.00023	0.00022	0.00013	0.00008	0.00007	0.00000	-0.00001	-0.00001	-0.00003	-0.00006	-0.00006	-0.00007	-0.00008	
8192	0.00034	0.00030	0.00016	0.00009	0.00007	-0.00003	-0.00004	-0.00007	-0.00010	-0.00012	-0.00014	-0.00015		
4096	0.00042	0.00038	0.00016	0.00006	0.00004	-0.00010	-0.00012	-0.00014	-0.00019	-0.00023	-0.00026			
2048	0.00050	0.00041	0.00012	0.00001	-0.00005	-0.00021	-0.00025	-0.00030	-0.00036	-0.00040				
1024	0.00055	0.00041	0.00003	-0.00011	-0.00015	-0.00039	-0.00045	-0.00050	-0.00058					
512	0.00047	0.00035	-0.00007	-0.00025	-0.00030	-0.00052	-0.00061	-0.00069						
256	0.00044	0.00033	-0.00018	-0.00034	-0.00039	-0.00064	-0.00072							
128	0.00036	0.00025	-0.00025	-0.00038	-0.00041	-0.00069								
64	0.00029	0.00025	-0.00025	-0.00041	-0.00048									
32	0.00027	0.00025	-0.00027	-0.00043										
16	0.00026	0.00027	-0.00030											
8	0.00011	0.00022												
4	0.00013													

(e) $\mathcal{P}^{\mathcal{C}}$ for Scene: [**Torus**] Algorithm: [**M-MLT**] Metric: [**SC-QI**] True GT: [**BDPT @ 524288 spp**].

ERPT	2	4	8	16	32	64	128	256	512	1024	2048	4096	8192	16384	32768	65536	131072
GT (BDPT)																	
262144	0.00001	0.00001	0.00001	0.00000	-0.00000	0.00000	0.00000	0.00001	0.00001	0.00000	0.00000	0.00000	0.00000	0.00000	0.00000	0.00000	0.00000
131072	0.00003	0.00001	0.00001	0.00001	0.00001	0.00001	0.00001	0.00001	0.00001	0.00001	0.00001	0.00001	0.00001	0.00001	0.00001	0.00001	
65536	0.00003	0.00001	0.00001	0.00001	0.00001	0.00001	0.00001	0.00002	0.00002	0.00001	0.00001	0.00001	0.00001	0.00001	0.00001		
32768	0.00003	0.00000	0.00001	-0.00000	-0.00000	0.00001	0.00001	0.00002	0.00001	0.00001	0.00000	-0.00000	-0.00000	-0.00000			
16384	0.00004	-0.00002	-0.00001	-0.00002	-0.00002	-0.00002	-0.00001	-0.00001	-0.00002	-0.00002	-0.00002	-0.00003	-0.00003				
8192	-0.00000	-0.00007	-0.00005	-0.00008	-0.00007	-0.00007	-0.00007	-0.00006	-0.00007	-0.00007	-0.00009	-0.00009					
4096	-0.00007	-0.00014	-0.00013	-0.00016	-0.00015	-0.00016	-0.00015	-0.00015	-0.00017	-0.00017	-0.00019						
2048	-0.00018	-0.00028	-0.00027	-0.00032	-0.00031	-0.00030	-0.00031	-0.00031	-0.00033	-0.00034							
1024	-0.00031	-0.00047	-0.00042	-0.00050	-0.00051	-0.00049	-0.00051	-0.00050	-0.00053								
512	-0.00046	-0.00063	-0.00061	-0.00067	-0.00067	-0.00068	-0.00068	-0.00069									
256	-0.00054	-0.00071	-0.00070	-0.00078	-0.00078	-0.00078	-0.00082										
128	-0.00051	-0.00069	-0.00070	-0.00079	-0.00084	-0.00083											
64	-0.00050	-0.00066	-0.00069	-0.00080	-0.00081												
32	-0.00048	-0.00068	-0.00069	-0.00078													
16	-0.00048	-0.00070	-0.00072														
8	-0.00053	-0.00076															
4	-0.00083																

(f) $\mathcal{P}^{\mathcal{C}}$ for Scene: [**Torus**] Algorithm: [**ERPT**] Metric: [**SC-QI**] True GT: [**BDPT @ 524288 spp**].

M-ERPT	2	4	8	16	32	64	128	256	512	1024	2048	4096	8192	16384	32768	65536	131072
GT (BDPT)																	
262144	0.00000	0.00001	0.00001	0.00000	0.00000	0.00001	0.00000	0.00000	0.00001	0.00000	0.00000	0.00000	0.00000	0.00000	0.00001	0.00000	0.00000
131072	0.00001	0.00001	0.00001	0.00001	0.00001	0.00001	0.00001	0.00001	0.00001	0.00001	0.00001	0.00001	0.00001	0.00001	0.00001	0.00001	
65536	0.00002	0.00001	0.00001	0.00001	0.00002	0.00002	0.00001	0.00001	0.00001	0.00001	0.00001	0.00001	0.00001	0.00001	0.00001		
32768	0.00002	0.00001	0.00002	-0.00000	0.00001	0.00001	0.00001	0.00001	0.00000	0.00000	0.00000	0.00000	0.00000	-0.00000			
16384	0.00001	0.00000	0.00001	-0.00002	0.00000	-0.00001	-0.00002	-0.00002	-0.00002	-0.00002	-0.00003	-0.00003	-0.00003				
8192	-0.00001	-0.00002	-0.00004	-0.00008	-0.00005	-0.00006	-0.00008	-0.00008	-0.00009	-0.00009	-0.00010	-0.00009					
4096	-0.00009	-0.00008	-0.00010	-0.00017	-0.00014	-0.00017	-0.00016	-0.00017	-0.00018	-0.00019	-0.00019						
2048	-0.00018	-0.00019	-0.00023	-0.00032	-0.00027	-0.00032	-0.00033	-0.00033	-0.00035	-0.00036							
1024	-0.00036	-0.00034	-0.00036	-0.00049	-0.00046	-0.00052	-0.00053	-0.00054	-0.00056								
512	-0.00048	-0.00047	-0.00052	-0.00066	-0.00063	-0.00070	-0.00071	-0.00073									
256	-0.00056	-0.00056	-0.00060	-0.00077	-0.00074	-0.00082	-0.00084										
128	-0.00054	-0.00057	-0.00062	-0.00080	-0.00076	-0.00086											
64	-0.00054	-0.00053	-0.00060	-0.00081	-0.00074												
32	-0.00056	-0.00055	-0.00059	-0.00078													
16	-0.00056	-0.00054	-0.00057														
8	-0.00061	-0.00059															
4	-0.00092																

(g) $\mathcal{P}^{\mathcal{C}}$ for Scene: [**Torus**] Algorithm: [**M-ERPT**] Metric: [**SC-QI**] True GT: [**BDPT @ 524288 spp**].

Table 57

13.3 Veach Bidir

PT	2	4	8	16	32	64	128	256	512	1024	2048	4096	8192	16384	32768	65536	131072
GT (BDPT)																	
262144	0.00000	0.00000	0.00000	0.00000	0.00000	0.00000	0.00000	0.00000	0.00000	0.00000	0.00000	0.00000	0.00000	0.00000	0.00000	0.00000	0.00000
131072	0.00000	0.00000	0.00000	0.00000	0.00000	0.00000	0.00000	0.00000	0.00000	0.00000	0.00000	0.00000	0.00000	0.00000	0.00000	0.00000	
65536	0.00001	0.00001	0.00001	0.00001	0.00001	0.00001	0.00001	0.00000	0.00000	0.00000	0.00000	0.00000	0.00000	0.00000	0.00000		
32768	0.00002	0.00002	0.00002	0.00001	0.00001	0.00001	0.00001	0.00001	0.00001	0.00001	0.00001	0.00001	0.00000	0.00000			
16384	0.00003	0.00003	0.00003	0.00003	0.00002	0.00002	0.00002	0.00002	0.00002	0.00001	0.00001	0.00001	0.00001				
8192	0.00005	0.00005	0.00005	0.00005	0.00004	0.00004	0.00003	0.00003	0.00003	0.00002	0.00002	0.00001					
4096	0.00009	0.00008	0.00008	0.00007	0.00006	0.00006	0.00005	0.00005	0.00004	0.00003	0.00003						
2048	0.00015	0.00014	0.00013	0.00012	0.00010	0.00010	0.00009	0.00008	0.00007	0.00006							
1024	0.00023	0.00022	0.00020	0.00018	0.00017	0.00015	0.00013	0.00012	0.00010								
512	0.00034	0.00034	0.00031	0.00027	0.00024	0.00021	0.00019	0.00017									
256	0.00051	0.00049	0.00043	0.00039	0.00035	0.00030	0.00027										
128	0.00076	0.00069	0.00064	0.00055	0.00049	0.00042											
64	0.00106	0.00099	0.00089	0.00076	0.00065												
32	0.00147	0.00133	0.00117	0.00099													
16	0.00196	0.00171	0.00146														
8	0.00246	0.00216															
4	0.00293																

(a) $\mathcal{P}^{\mathcal{C}}$ for Scene: [**Veach Bidir**] Algorithm: [**PT**] Metric: [**SC-QI**] True GT: [**BDPT @ 524288 spp**].

BDPT	2	4	8	16	32	64	128	256	512	1024	2048	4096	8192	16384	32768	65536	131072
GT (BDPT)																	
262144	0.00000	0.00000	0.00000	0.00000	0.00000	0.00000	0.00000	0.00000	0.00000	0.00000	0.00000	-0.00000	0.00000	0.00000	0.00000	-0.00000	-0.00000
131072	0.00000	0.00000	0.00000	0.00000	0.00000	0.00000	0.00000	0.00000	0.00000	-0.00000	-0.00000	-0.00000	0.00000	-0.00000	0.00000	-0.00000	
65536	0.00001	0.00001	0.00000	0.00000	0.00000	0.00000	0.00000	0.00000	0.00000	0.00000	0.00000	-0.00000	0.00000	-0.00000	-0.00000		
32768	0.00001	0.00001	0.00001	0.00001	0.00000	0.00000	0.00000	0.00000	0.00000	-0.00000	-0.00000	-0.00000	-0.00000	-0.00000			
16384	0.00002	0.00002	0.00002	0.00001	0.00001	0.00001	0.00001	0.00000	0.00000	0.00000	0.00000	-0.00000	-0.00000				
8192	0.00004	0.00003	0.00003	0.00002	0.00002	0.00001	0.00001	0.00001	0.00000	0.00000	-0.00000	-0.00000					
4096	0.00006	0.00005	0.00004	0.00003	0.00002	0.00002	0.00001	0.00001	0.00000	0.00000	-0.00000						
2048	0.00011	0.00009	0.00007	0.00005	0.00004	0.00003	0.00002	0.00001	0.00001	0.00000							
1024	0.00016	0.00013	0.00010	0.00009	0.00006	0.00004	0.00003	0.00002	0.00001								
512	0.00024	0.00019	0.00015	0.00012	0.00008	0.00005	0.00003	0.00001									
256	0.00034	0.00027	0.00020	0.00015	0.00010	0.00005	0.00002										
128	0.00048	0.00037	0.00027	0.00018	0.00011	0.00005											
64	0.00063	0.00048	0.00033	0.00020	0.00010												
32	0.00081	0.00056	0.00036	0.00017													
16	0.00093	0.00060	0.00031														
8	0.00099	0.00054															
4	0.00092																

(b) $\mathcal{P}^{\mathcal{C}}$ for Scene: [**Veach Bidir**] Algorithm: [**BDPT**] Metric: [**SC-QI**] True GT: [**BDPT @ 524288 spp**].

PSSMLT	2	4	8	16	32	64	128	256	512	1024	2048	4096	8192	16384
GT (BDPT)														
262144	0.00000	0.00000	0.00000	0.00000	0.00000	0.00000	0.00000	0.00000	0.00000	0.00000	0.00000	0.00000	0.00000	0.00000
131072	0.00000	0.00000	0.00000	0.00000	0.00000	0.00000	-0.00000	-0.00000	-0.00000	-0.00000	0.00000	-0.00000	-0.00000	-0.00000
65536	0.00001	0.00000	0.00000	0.00000	0.00000	0.00000	0.00000	0.00000	0.00000	0.00000	0.00000	0.00000	-0.00000	-0.00000
32768	0.00001	0.00001	0.00001	0.00001	0.00000	0.00000	0.00000	0.00000	0.00000	0.00000	0.00000	-0.00000	-0.00000	-0.00000
16384	0.00002	0.00002	0.00001	0.00001	0.00001	0.00001	0.00001	0.00000	0.00000	0.00000	-0.00000	-0.00000	-0.00000	
8192	0.00003	0.00003	0.00002	0.00002	0.00002	0.00001	0.00001	0.00001	0.00000	0.00000	0.00000	-0.00000		
4096	0.00005	0.00004	0.00004	0.00003	0.00002	0.00002	0.00001	0.00001	0.00001	0.00000	0.00000			
2048	0.00009	0.00007	0.00006	0.00005	0.00004	0.00003	0.00002	0.00002	0.00001	0.00001				
1024	0.00014	0.00012	0.00009	0.00007	0.00006	0.00004	0.00003	0.00002	0.00001					
512	0.00021	0.00017	0.00013	0.00010	0.00007	0.00005	0.00003	0.00002						
256	0.00029	0.00023	0.00018	0.00013	0.00009	0.00006	0.00003							
128	0.00040	0.00032	0.00023	0.00016	0.00010	0.00005								
64	0.00055	0.00042	0.00030	0.00020	0.00010									
32	0.00069	0.00049	0.00033	0.00016										
16	0.00082	0.00053	0.00027											
8	0.00089	0.00048												
4	0.00079													

(c) $\mathcal{P}^{\mathcal{C}}$ for Scene: [**Veach Bidir**] Algorithm: [**PSSMLT**] Metric: [**SC-QI**] True GT: [**BDPT @ 524288 spp**].

MLT	2	4	8	16	32	64	128	256	512	1024	2048	4096	8192	16384
GT (BDPT)														
262144	0.00000	0.00000	0.00000	0.00000	0.00000	0.00000	0.00000	0.00000	0.00000	0.00000	0.00000	0.00000	0.00000	0.00000
131072	0.00000	0.00000	0.00000	0.00000	0.00000	0.00000	0.00000	0.00000	0.00000	0.00000	0.00000	0.00000	-0.00000	-0.00000
65536	0.00001	0.00001	0.00001	0.00000	0.00000	0.00000	0.00000	0.00000	0.00000	0.00000	0.00000	0.00000	0.00000	0.00000
32768	0.00001	0.00001	0.00001	0.00001	0.00001	0.00001	0.00000	0.00000	0.00000	0.00000	0.00000	-0.00000	-0.00000	-0.00000
16384	0.00002	0.00002	0.00002	0.00001	0.00001	0.00001	0.00001	0.00001	0.00001	0.00000	0.00000	0.00000	0.00000	
8192	0.00004	0.00004	0.00003	0.00003	0.00002	0.00002	0.00002	0.00001	0.00001	0.00001	0.00001	0.00000		
4096	0.00006	0.00006	0.00005	0.00004	0.00004	0.00003	0.00002	0.00002	0.00001	0.00001	0.00001			
2048	0.00011	0.00010	0.00009	0.00007	0.00006	0.00005	0.00004	0.00003	0.00002	0.00002				
1024	0.00017	0.00016	0.00014	0.00011	0.00009	0.00007	0.00006	0.00004	0.00003					
512	0.00026	0.00024	0.00020	0.00017	0.00013	0.00011	0.00008	0.00005						
256	0.00040	0.00035	0.00029	0.00024	0.00019	0.00014	0.00010							
128	0.00059	0.00052	0.00042	0.00034	0.00026	0.00018								
64	0.00084	0.00072	0.00058	0.00045	0.00032									
32	0.00114	0.00095	0.00075	0.00055										
16	0.00153	0.00125	0.00091											
8	0.00191	0.00147												
4	0.00224													

(d) $\mathcal{P}^{\mathcal{C}}$ for Scene: [**Veach Bidir**] Algorithm: [**MLT**] Metric: [**SC-QI**] True GT: [**BDPT @ 524288 spp**].

M-MLT	2	4	8	16	32	64	128	256	512	1024	2048	4096	8192	16384
GT (BDPT)														
262144	0.00000	0.00000	0.00000	0.00000	0.00000	0.00000	0.00000	0.00000	0.00000	0.00000	0.00000	0.00000	0.00000	0.00000
131072	0.00000	0.00000	0.00000	0.00000	0.00000	0.00000	0.00000	0.00000	-0.00000	-0.00000	-0.00000	-0.00000	-0.00000	-0.00000
65536	0.00001	0.00001	0.00000	0.00000	0.00000	0.00000	0.00000	0.00000	0.00000	0.00000	0.00000	0.00000	0.00000	0.00000
32768	0.00001	0.00001	0.00001	0.00001	0.00001	0.00001	0.00000	0.00000	0.00000	0.00000	-0.00000	-0.00000	-0.00000	-0.00000
16384	0.00002	0.00002	0.00002	0.00002	0.00001	0.00001	0.00001	0.00001	0.00001	0.00000	0.00000	0.00000	0.00000	
8192	0.00004	0.00003	0.00003	0.00003	0.00002	0.00002	0.00001	0.00001	0.00001	0.00000	0.00000	0.00000		
4096	0.00006	0.00006	0.00005	0.00004	0.00004	0.00003	0.00002	0.00002	0.00001	0.00001	0.00001			
2048	0.00011	0.00010	0.00009	0.00008	0.00006	0.00005	0.00004	0.00003	0.00002	0.00001				
1024	0.00017	0.00016	0.00013	0.00011	0.00009	0.00007	0.00006	0.00004	0.00003					
512	0.00026	0.00024	0.00020	0.00017	0.00013	0.00010	0.00008	0.00005						
256	0.00038	0.00035	0.00029	0.00024	0.00018	0.00014	0.00010							
128	0.00058	0.00050	0.00041	0.00033	0.00025	0.00018								
64	0.00082	0.00070	0.00056	0.00043	0.00031									
32	0.00113	0.00095	0.00075	0.00054										
16	0.00146	0.00122	0.00090											
8	0.00190	0.00148												
4	0.00221													

(e) $\mathcal{P}^{\mathcal{C}}$ for Scene: [**Veach Bidir**] Algorithm: [**M-MLT**] Metric: [**SC-QI**] True GT: [**BDPT @ 524288 spp**].

ERPT	2	4	8	16	32	64	128	256	512	1024	2048	4096	8192	16384	32768	65536	131072
GT (BDPT)																	
262144	0.00000	0.00000	0.00000	0.00000	0.00000	0.00000	0.00000	0.00000	0.00000	0.00000	0.00000	0.00000	0.00000	0.00000	0.00000	0.00000	0.00000
131072	0.00000	0.00000	0.00000	0.00000	0.00000	0.00000	0.00000	0.00000	0.00000	0.00000	0.00000	0.00000	0.00000	0.00000	0.00000	0.00000	
65536	0.00000	0.00000	0.00000	0.00000	0.00000	0.00000	0.00000	0.00000	0.00000	0.00000	0.00000	0.00000	0.00000	0.00000	0.00000		
32768	0.00000	0.00000	0.00000	0.00000	0.00000	0.00000	0.00000	0.00000	0.00000	0.00000	0.00000	0.00000	0.00000	0.00000			
16384	0.00001	0.00001	0.00001	0.00001	0.00001	0.00001	0.00001	0.00001	0.00001	0.00001	0.00001	0.00001	0.00001				
8192	0.00001	0.00001	0.00002	0.00001	0.00001	0.00001	0.00001	0.00001	0.00001	0.00001	0.00001	0.00001					
4096	0.00002	0.00002	0.00002	0.00002	0.00002	0.00002	0.00002	0.00002	0.00002	0.00002	0.00002						
2048	0.00004	0.00004	0.00004	0.00004	0.00004	0.00004	0.00004	0.00003	0.00003	0.00003							
1024	0.00005	0.00005	0.00005	0.00005	0.00006	0.00005	0.00005	0.00005	0.00005								
512	0.00007	0.00007	0.00007	0.00007	0.00008	0.00007	0.00007	0.00007									
256	0.00008	0.00009	0.00009	0.00009	0.00009	0.00009	0.00009										
128	0.00010	0.00010	0.00010	0.00010	0.00010	0.00010											
64	0.00009	0.00010	0.00010	0.00010	0.00009												
32	0.00004	0.00003	0.00003	0.00003													
16	-0.00013	-0.00012	-0.00012														
8	-0.00045	-0.00045															
4	-0.00103																

(f) $\mathcal{P}^{\mathcal{C}}$ for Scene: [**Veach Bidir**] Algorithm: [**ERPT**] Metric: [**SC-QI**] True GT: [**BDPT @ 524288 spp**].

M-ERPT	2	4	8	16	32	64	128	256	512	1024	2048	4096	8192	16384	32768	65536	131072
GT (BDPT)																	
262144	0.00000	0.00000	0.00000	0.00000	0.00000	0.00000	-0.00000	0.00000	0.00000	0.00000	0.00000	0.00000	0.00000	0.00000	0.00000	0.00000	0.00000
131072	0.00000	0.00000	-0.00000	0.00000	0.00000	0.00000	-0.00000	0.00000	0.00000	0.00000	0.00000	0.00000	0.00000	0.00000	0.00000	0.00000	
65536	0.00000	0.00000	0.00000	0.00000	0.00000	0.00000	0.00000	0.00000	0.00000	0.00000	0.00000	0.00000	0.00000	0.00000	0.00000		
32768	0.00000	0.00000	0.00000	0.00001	0.00000	0.00000	0.00000	0.00000	0.00000	0.00000	0.00000	0.00000	0.00000	0.00000			
16384	0.00001	0.00001	0.00001	0.00001	0.00001	0.00001	0.00001	0.00001	0.00001	0.00001	0.00000	0.00001	0.00001				
8192	0.00001	0.00001	0.00001	0.00001	0.00001	0.00001	0.00001	0.00001	0.00001	0.00001	0.00001	0.00001					
4096	0.00002	0.00002	0.00002	0.00002	0.00002	0.00002	0.00002	0.00002	0.00002	0.00002	0.00002						
2048	0.00004	0.00004	0.00003	0.00004	0.00004	0.00003	0.00004	0.00004	0.00004	0.00003							
1024	0.00006	0.00006	0.00005	0.00006	0.00006	0.00005	0.00005	0.00005	0.00005								
512	0.00007	0.00008	0.00007	0.00007	0.00008	0.00007	0.00007	0.00007									
256	0.00009	0.00009	0.00009	0.00009	0.00010	0.00009	0.00009										
128	0.00011	0.00011	0.00009	0.00010	0.00010	0.00010											
64	0.00012	0.00012	0.00009	0.00010	0.00011												
32	0.00005	0.00006	0.00004	0.00005													
16	-0.00010	-0.00009	-0.00012														
8	-0.00042	-0.00041															
4	-0.00098																

(g) $\mathcal{P}^{\mathcal{C}}$ for Scene: [**Veach Bidir**] Algorithm: [**M-ERPT**] Metric: [**SC-QI**] True GT: [**BDPT @ 524288 spp**].

Table 58

13.4 Veach Door

PT	2	4	8	16	32	64	128	256	512	1024	2048	4096	8192	16384	32768	65536	131072
GT (BDPT)																	
262144	0.00002	0.00002	0.00002	0.00002	0.00002	0.00002	0.00001	0.00001	0.00001	0.00001	0.00001	0.00000	0.00000	0.00000	0.00000	0.00000	0.00000
131072	0.00004	0.00005	0.00005	0.00005	0.00004	0.00004	0.00003	0.00003	0.00003	0.00002	0.00002	0.00001	0.00001	0.00001	0.00000	0.00000	
65536	0.00009	0.00011	0.00011	0.00011	0.00009	0.00008	0.00007	0.00007	0.00006	0.00005	0.00004	0.00003	0.00002	0.00002	0.00001		
32768	0.00017	0.00020	0.00021	0.00019	0.00017	0.00015	0.00013	0.00011	0.00010	0.00008	0.00006	0.00005	0.00004	0.00002			
16384	0.00029	0.00032	0.00034	0.00032	0.00028	0.00024	0.00021	0.00019	0.00016	0.00013	0.00010	0.00007	0.00005				
8192	0.00050	0.00055	0.00056	0.00050	0.00044	0.00038	0.00034	0.00029	0.00024	0.00019	0.00014	0.00010					
4096	0.00079	0.00084	0.00084	0.00076	0.00067	0.00056	0.00049	0.00041	0.00034	0.00027	0.00019						
2048	0.00119	0.00126	0.00123	0.00110	0.00094	0.00081	0.00068	0.00057	0.00045	0.00034							
1024	0.00177	0.00183	0.00177	0.00156	0.00132	0.00110	0.00092	0.00073	0.00057								
512	0.00250	0.00250	0.00235	0.00202	0.00169	0.00138	0.00116	0.00087									
256	0.00327	0.00326	0.00301	0.00258	0.00210	0.00168	0.00130										
128	0.00406	0.00399	0.00361	0.00301	0.00231	0.00179											
64	0.00498	0.00488	0.00419	0.00335	0.00249												
32	0.00583	0.00557	0.00466	0.00345													
16	0.00674	0.00616	0.00481														
8	0.00739	0.00654															
4	0.00789																

(a) $\mathcal{P}^{\mathcal{C}}$ for Scene: [**Veach Door**] Algorithm: [**PT**] Metric: [**SC-QI**] True GT: [**BDPT @ 524288 spp**].

BDPT	2	4	8	16	32	64	128	256	512	1024	2048	4096	8192	16384	32768	65536	131072
GT (BDPT)																	
262144	0.00002	0.00002	0.00002	0.00002	0.00001	0.00001	0.00001	0.00001	0.00001	0.00000	0.00000	0.00000	0.00000	0.00000	-0.00000	-0.00000	-0.00000
131072	0.00005	0.00005	0.00005	0.00005	0.00004	0.00003	0.00002	0.00002	0.00001	0.00001	0.00001	0.00001	0.00000	0.00000	-0.00000	-0.00000	
65536	0.00011	0.00011	0.00011	0.00010	0.00008	0.00007	0.00006	0.00004	0.00003	0.00002	0.00002	0.00001	0.00000	0.00000	-0.00000		
32768	0.00021	0.00021	0.00020	0.00017	0.00014	0.00012	0.00009	0.00007	0.00005	0.00004	0.00002	0.00001	0.00000	-0.00000			
16384	0.00034	0.00034	0.00031	0.00028	0.00023	0.00019	0.00015	0.00011	0.00008	0.00005	0.00003	0.00001	0.00000				
8192	0.00056	0.00055	0.00049	0.00044	0.00036	0.00029	0.00022	0.00016	0.00011	0.00007	0.00003	0.00000					
4096	0.00085	0.00084	0.00075	0.00065	0.00053	0.00042	0.00031	0.00022	0.00014	0.00007	0.00002						
2048	0.00128	0.00121	0.00109	0.00091	0.00074	0.00058	0.00040	0.00027	0.00016	0.00006							
1024	0.00185	0.00172	0.00151	0.00125	0.00100	0.00073	0.00048	0.00029	0.00012								
512	0.00249	0.00230	0.00200	0.00158	0.00124	0.00085	0.00051	0.00021									
256	0.00330	0.00293	0.00246	0.00192	0.00140	0.00089	0.00041										
128	0.00404	0.00349	0.00291	0.00213	0.00138	0.00071											
64	0.00475	0.00406	0.00317	0.00218	0.00123												
32	0.00544	0.00446	0.00327	0.00184													
16	0.00593	0.00459	0.00307														
8	0.00628	0.00434															
4	0.00599																

(b) $\mathcal{P}^{\mathcal{C}}$ for Scene: [**Veach Door**] Algorithm: [**BDPT**] Metric: [**SC-QI**] True GT: [**BDPT @ 524288 spp**].

PSSMLT	2	4	8	16	32	64	128	256	512	1024	2048	4096	8192	16384
GT (BDPT)														
262144	0.00001	0.00001	0.00001	0.00001	0.00001	0.00001	0.00001	0.00000	0.00000	0.00000	0.00000	0.00000	-0.00000	-0.00000
131072	0.00004	0.00003	0.00003	0.00002	0.00002	0.00002	0.00001	0.00001	0.00001	0.00000	0.00000	0.00000	-0.00000	-0.00000
65536	0.00008	0.00007	0.00006	0.00005	0.00004	0.00004	0.00003	0.00002	0.00002	0.00001	0.00001	0.00000	-0.00000	-0.00000
32768	0.00015	0.00013	0.00012	0.00010	0.00008	0.00006	0.00005	0.00004	0.00002	0.00001	0.00001	-0.00000	-0.00001	-0.00001
16384	0.00025	0.00022	0.00019	0.00015	0.00013	0.00010	0.00007	0.00005	0.00003	0.00002	0.00000	-0.00001	-0.00002	
8192	0.00041	0.00036	0.00030	0.00024	0.00019	0.00015	0.00011	0.00007	0.00004	0.00001	-0.00001	-0.00003		
4096	0.00064	0.00056	0.00046	0.00036	0.00029	0.00022	0.00015	0.00010	0.00004	-0.00001	-0.00004			
2048	0.00095	0.00081	0.00066	0.00052	0.00039	0.00028	0.00018	0.00009	0.00001	-0.00006				
1024	0.00141	0.00116	0.00091	0.00070	0.00050	0.00033	0.00018	0.00004	-0.00009					
512	0.00188	0.00150	0.00116	0.00084	0.00055	0.00030	0.00006	-0.00013						
256	0.00235	0.00185	0.00138	0.00093	0.00051	0.00016	-0.00016							
128	0.00280	0.00208	0.00141	0.00084	0.00029	-0.00022								
64	0.00321	0.00224	0.00139	0.00056	-0.00021									
32	0.00346	0.00217	0.00098	-0.00012										
16	0.00341	0.00161	-0.00005											
8	0.00300	0.00062												
4	0.00193													

(c) $\mathcal{P}^{\mathcal{C}}$ for Scene: [**Veach Door**] Algorithm: [**PSSMLT**] Metric: [**SC-QI**] True GT: [**BDPT @ 524288 spp**].

MLT	2	4	8	16	32	64	128	256	512	1024	2048	4096	8192	16384
GT (BDPT)														
262144	0.00002	0.00001	0.00001	0.00001	0.00001	0.00001	0.00000	0.00000	0.00000	0.00000	0.00000	0.00000	-0.00000	-0.00000
131072	0.00004	0.00004	0.00003	0.00003	0.00002	0.00001	0.00001	0.00001	0.00001	0.00000	0.00000	0.00000	-0.00000	-0.00000
65536	0.00009	0.00008	0.00006	0.00005	0.00004	0.00003	0.00002	0.00002	0.00001	0.00001	0.00000	-0.00000	-0.00000	-0.00001
32768	0.00016	0.00013	0.00011	0.00009	0.00006	0.00005	0.00004	0.00003	0.00002	0.00001	0.00000	-0.00001	-0.00001	-0.00001
16384	0.00027	0.00023	0.00018	0.00015	0.00011	0.00008	0.00006	0.00004	0.00002	0.00001	-0.00000	-0.00001	-0.00002	
8192	0.00044	0.00037	0.00029	0.00023	0.00017	0.00011	0.00008	0.00005	0.00002	-0.00000	-0.00002	-0.00004		
4096	0.00066	0.00055	0.00043	0.00033	0.00023	0.00015	0.00010	0.00004	0.00000	-0.00003	-0.00006			
2048	0.00097	0.00078	0.00061	0.00045	0.00030	0.00019	0.00010	0.00002	-0.00005	-0.00010				
1024	0.00138	0.00110	0.00081	0.00060	0.00037	0.00019	0.00005	-0.00007	-0.00016					
512	0.00181	0.00141	0.00103	0.00066	0.00034	0.00010	-0.00012	-0.00029						
256	0.00227	0.00173	0.00112	0.00068	0.00025	-0.00011	-0.00039							
128	0.00263	0.00192	0.00111	0.00049	-0.00009	-0.00056								
64	0.00296	0.00195	0.00094	0.00008	-0.00070									
32	0.00313	0.00174	0.00036	-0.00078										
16	0.00304	0.00121	-0.00068											
8	0.00246	0.00003												
4	0.00122													

(d) $\mathcal{P}^{\mathcal{C}}$ for Scene: [**Veach Door**] Algorithm: [**MLT**] Metric: [**SC-QI**] True GT: [**BDPT @ 524288 spp**].

M-MLT	2	4	8	16	32	64	128	256	512	1024	2048	4096	8192	16384
GT (BDPT)														
262144	0.00001	0.00001	0.00001	0.00001	0.00001	0.00001	0.00000	0.00000	0.00000	0.00000	0.00000	0.00000	-0.00000	-0.00000
131072	0.00004	0.00003	0.00003	0.00002	0.00002	0.00001	0.00001	0.00001	0.00001	0.00000	0.00000	0.00000	-0.00000	-0.00000
65536	0.00009	0.00007	0.00006	0.00005	0.00004	0.00003	0.00002	0.00002	0.00001	0.00001	0.00000	-0.00000	-0.00000	-0.00001
32768	0.00016	0.00013	0.00010	0.00009	0.00007	0.00005	0.00003	0.00002	0.00002	0.00001	0.00000	-0.00001	-0.00001	-0.00001
16384	0.00027	0.00022	0.00018	0.00014	0.00011	0.00008	0.00006	0.00004	0.00002	0.00001	-0.00000	-0.00001	-0.00002	
8192	0.00044	0.00036	0.00028	0.00022	0.00016	0.00012	0.00008	0.00005	0.00002	-0.00000	-0.00002	-0.00004		
4096	0.00067	0.00055	0.00042	0.00033	0.00024	0.00016	0.00010	0.00005	0.00000	-0.00003	-0.00006			
2048	0.00099	0.00078	0.00057	0.00045	0.00031	0.00020	0.00010	0.00002	-0.00004	-0.00010				
1024	0.00138	0.00110	0.00081	0.00057	0.00037	0.00020	0.00006	-0.00007	-0.00016					
512	0.00183	0.00139	0.00096	0.00066	0.00035	0.00012	-0.00009	-0.00028						
256	0.00228	0.00167	0.00113	0.00066	0.00023	-0.00008	-0.00039							
128	0.00266	0.00184	0.00112	0.00046	-0.00008	-0.00056								
64	0.00299	0.00190	0.00085	0.00006	-0.00075									
32	0.00301	0.00161	0.00033	-0.00078										
16	0.00293	0.00103	-0.00079											
8	0.00223	-0.00017												
4	0.00088													

(e) $\mathcal{P}^{\mathcal{C}}$ for Scene: [**Veach Door**] Algorithm: [**M-MLT**] Metric: [**SC-QI**] True GT: [**BDPT @ 524288 spp**].

ERPT	2	4	8	16	32	64	128	256	512	1024	2048	4096	8192	16384	32768	65536	131072
GT (BDPT)																	
262144	0.00000	0.00000	0.00000	0.00000	0.00000	0.00000	0.00000	0.00000	0.00000	0.00000	0.00000	0.00000	0.00000	0.00000	0.00000	0.00000	0.00000
131072	0.00001	0.00001	0.00001	0.00001	0.00001	0.00001	0.00001	0.00001	0.00001	0.00001	0.00001	0.00001	0.00001	0.00001	0.00001	0.00001	
65536	0.00002	0.00002	0.00002	0.00002	0.00002	0.00002	0.00002	0.00002	0.00002	0.00002	0.00002	0.00002	0.00002	0.00002	0.00002		
32768	0.00003	0.00003	0.00003	0.00003	0.00003	0.00003	0.00003	0.00003	0.00003	0.00003	0.00003	0.00003	0.00003	0.00003			
16384	0.00004	0.00005	0.00005	0.00005	0.00005	0.00005	0.00005	0.00005	0.00005	0.00005	0.00005	0.00005	0.00005				
8192	0.00006	0.00006	0.00006	0.00006	0.00007	0.00007	0.00007	0.00007	0.00007	0.00007	0.00007	0.00007					
4096	0.00007	0.00007	0.00007	0.00008	0.00008	0.00008	0.00008	0.00008	0.00007	0.00008	0.00008						
2048	0.00005	0.00006	0.00006	0.00007	0.00007	0.00007	0.00007	0.00007	0.00006	0.00007							
1024	-0.00000	-0.00000	0.00000	0.00000	0.00002	0.00001	0.00002	0.00001	0.00001								
512	-0.00017	-0.00018	-0.00016	-0.00015	-0.00015	-0.00017	-0.00016	-0.00016									
256	-0.00050	-0.00048	-0.00049	-0.00046	-0.00047	-0.00047	-0.00048										
128	-0.00110	-0.00111	-0.00110	-0.00109	-0.00108	-0.00110											
64	-0.00206	-0.00203	-0.00205	-0.00206	-0.00204												
32	-0.00353	-0.00352	-0.00354	-0.00353													
16	-0.00550	-0.00555	-0.00556														
8	-0.00822	-0.00825															
4	-0.01160																

(f) $\mathcal{P}^{\mathcal{C}}$ for Scene: [**Veach Door**] Algorithm: [**ERPT**] Metric: [**SC-QI**] True GT: [**BDPT @ 524288 spp**].

M-ERPT	2	4	8	16	32	64	128	256	512	1024	2048	4096	8192	16384	32768	65536	131072
GT (BDPT)																	
262144	0.00000	0.00000	0.00000	0.00000	0.00000	0.00000	0.00000	0.00000	0.00000	0.00000	0.00000	0.00000	0.00000	0.00000	0.00000	0.00000	0.00000
131072	0.00001	0.00001	0.00001	0.00001	0.00001	0.00001	0.00001	0.00001	0.00001	0.00001	0.00001	0.00001	0.00001	0.00001	0.00001	0.00001	
65536	0.00002	0.00002	0.00002	0.00002	0.00002	0.00002	0.00002	0.00002	0.00002	0.00002	0.00002	0.00002	0.00002	0.00002	0.00002		
32768	0.00004	0.00004	0.00003	0.00004	0.00004	0.00004	0.00004	0.00004	0.00004	0.00004	0.00004	0.00004	0.00004	0.00004			
16384	0.00005	0.00005	0.00006	0.00005	0.00006	0.00005	0.00006	0.00006	0.00006	0.00005	0.00006	0.00006	0.00005				
8192	0.00007	0.00008	0.00008	0.00007	0.00008	0.00008	0.00008	0.00008	0.00008	0.00007	0.00007	0.00008					
4096	0.00009	0.00009	0.00009	0.00009	0.00009	0.00010	0.00009	0.00009	0.00009	0.00010	0.00010						
2048	0.00009	0.00009	0.00009	0.00009	0.00009	0.00009	0.00009	0.00009	0.00009	0.00009							
1024	0.00004	0.00004	0.00003	0.00005	0.00003	0.00004	0.00004	0.00004	0.00005								
512	-0.00012	-0.00012	-0.00011	-0.00011	-0.00011	-0.00011	-0.00011	-0.00012									
256	-0.00042	-0.00042	-0.00042	-0.00040	-0.00042	-0.00041	-0.00042										
128	-0.00101	-0.00100	-0.00102	-0.00102	-0.00102	-0.00102											
64	-0.00193	-0.00194	-0.00195	-0.00195	-0.00197												
32	-0.00336	-0.00340	-0.00342	-0.00340													
16	-0.00532	-0.00535	-0.00539														
8	-0.00804	-0.00810															
4	-0.01141																

(g) $\mathcal{P}^{\mathcal{C}}$ for Scene: [**Veach Door**] Algorithm: [**M-ERPT**] Metric: [**SC-QI**] True GT: [**BDPT @ 524288 spp**].

Table 59

13.5 Sponza

PT	2	4	8	16	32	64	128	256	512	1024	2048	4096	8192
GT (PT)													
16384	0.00004	0.00004	0.00004	0.00003	0.00002	0.00002	0.00001	0.00001	0.00000	0.00000	-0.00000	-0.00000	-0.00000
8192	0.00011	0.00010	0.00009	0.00008	0.00006	0.00004	0.00003	0.00002	0.00001	0.00000	-0.00000	-0.00001	
4096	0.00021	0.00020	0.00018	0.00014	0.00011	0.00008	0.00006	0.00003	0.00001	-0.00000	-0.00001		
2048	0.00036	0.00034	0.00031	0.00025	0.00018	0.00013	0.00008	0.00004	0.00001	-0.00002			
1024	0.00057	0.00054	0.00048	0.00039	0.00027	0.00018	0.00010	0.00004	-0.00002				
512	0.00090	0.00083	0.00072	0.00057	0.00039	0.00025	0.00011	0.00001					
256	0.00124	0.00116	0.00097	0.00073	0.00046	0.00025	0.00007						
128	0.00175	0.00157	0.00129	0.00091	0.00051	0.00017							
64	0.00224	0.00193	0.00149	0.00094	0.00036								
32	0.00260	0.00212	0.00151	0.00078									
16	0.00289	0.00219	0.00131										
8	0.00284	0.00188											
4	0.00252												

(a) $\mathcal{P}^{\mathcal{C}}$ for Scene: [**Sponza**] Algorithm: [**PT**] Metric: [**SC-QI**] True GT: [**PT @ 32768 spp**].

BDPT	2	4	8	16	32	64	128	256	512	1024	2048	4096	8192
GT (PT)													
16384	0.00004	0.00005	0.00004	0.00003	0.00003	0.00002	0.00001	0.00001	0.00001	0.00000	0.00000	-0.00000	-0.00000
8192	0.00010	0.00011	0.00010	0.00008	0.00007	0.00005	0.00004	0.00002	0.00001	0.00000	-0.00000	-0.00001	
4096	0.00020	0.00022	0.00019	0.00017	0.00013	0.00009	0.00006	0.00004	0.00002	0.00000	-0.00001		
2048	0.00035	0.00037	0.00032	0.00028	0.00021	0.00015	0.00010	0.00005	0.00002	-0.00001			
1024	0.00056	0.00059	0.00053	0.00043	0.00032	0.00022	0.00013	0.00006	-0.00000				
512	0.00089	0.00088	0.00080	0.00065	0.00047	0.00030	0.00015	0.00003					
256	0.00125	0.00124	0.00107	0.00083	0.00058	0.00033	0.00012						
128	0.00174	0.00169	0.00142	0.00107	0.00068	0.00029							
64	0.00225	0.00212	0.00167	0.00118	0.00060								
32	0.00265	0.00241	0.00181	0.00111									
16	0.00297	0.00249	0.00172										
8	0.00306	0.00231											
4	0.00281												

(b) $\mathcal{P}^{\mathcal{C}}$ for Scene: [**Sponza**] Algorithm: [**BDPT**] Metric: [**SC-QI**] True GT: [**PT @ 32768 spp**].

PSSMLT	2	4	8	16	32	64	128	256	512	1024	2048	4096	8192
GT (PT)													
16384	0.00003	0.00003	0.00002	0.00002	0.00002	0.00002	0.00001	0.00001	0.00001	0.00000	0.00000	0.00000	0.00000
8192	0.00008	0.00008	0.00007	0.00006	0.00005	0.00004	0.00003	0.00002	0.00001	0.00001	0.00000	-0.00000	
4096	0.00016	0.00015	0.00013	0.00011	0.00009	0.00007	0.00006	0.00004	0.00002	0.00001	0.00000		
2048	0.00026	0.00026	0.00022	0.00019	0.00015	0.00012	0.00009	0.00006	0.00003	0.00001			
1024	0.00043	0.00041	0.00035	0.00029	0.00023	0.00018	0.00012	0.00007	0.00002				
512	0.00066	0.00062	0.00053	0.00043	0.00033	0.00024	0.00014	0.00006					
256	0.00092	0.00086	0.00071	0.00055	0.00039	0.00024	0.00010						
128	0.00129	0.00113	0.00092	0.00066	0.00039	0.00018							
64	0.00161	0.00136	0.00102	0.00065	0.00029								
32	0.00181	0.00144	0.00095	0.00044									
16	0.00186	0.00131	0.00051										
8	0.00159	0.00085											
4	0.00090												

(c) $\mathcal{P}^{\mathcal{C}}$ for Scene: [**Sponza**] Algorithm: [**PSSMLT**] Metric: [**SC-QI**] True GT: [**PT @ 32768 spp**].

MLT	2	4	8	16	32	64	128	256	512	1024	2048	4096	8192
GT (PT)													
16384	0.00003	0.00003	0.00002	0.00002	0.00001	0.00001	0.00001	0.00000	0.00000	0.00000	-0.00000	-0.00000	-0.00000
8192	0.00009	0.00007	0.00006	0.00005	0.00003	0.00002	0.00002	0.00001	0.00000	0.00000	-0.00000	-0.00000	
4096	0.00016	0.00014	0.00011	0.00009	0.00006	0.00005	0.00003	0.00002	0.00001	-0.00000	-0.00000		
2048	0.00027	0.00023	0.00019	0.00014	0.00010	0.00007	0.00004	0.00002	0.00000	-0.00001			
1024	0.00045	0.00037	0.00030	0.00022	0.00014	0.00009	0.00003	0.00001	-0.00002				
512	0.00069	0.00055	0.00044	0.00030	0.00019	0.00010	0.00002	-0.00003					
256	0.00094	0.00074	0.00056	0.00035	0.00018	0.00004	-0.00007						
128	0.00130	0.00098	0.00068	0.00037	0.00010	-0.00011							
64	0.00160	0.00114	0.00065	0.00025	-0.00017								
32	0.00177	0.00107	0.00044	-0.00013									
16	0.00172	0.00076	-0.00017										
8	0.00142	0.00005											
4	0.00059												

(d) $\mathcal{P}^{\mathcal{C}}$ for Scene: [**Sponza**] Algorithm: [**MLT**] Metric: [**SC-QI**] True GT: [**PT @ 32768 spp**].

M-MLT	2	4	8	16	32	64	128	256	512	1024	2048	4096	8192
GT (PT)													
16384	0.00003	0.00003	0.00002	0.00002	0.00001	0.00001	0.00001	0.00000	0.00000	0.00000	-0.00000	0.00000	-0.00000
8192	0.00008	0.00007	0.00006	0.00005	0.00003	0.00003	0.00002	0.00001	0.00001	0.00000	-0.00000	-0.00000	
4096	0.00016	0.00014	0.00012	0.00009	0.00006	0.00004	0.00003	0.00001	0.00001	-0.00000	-0.00001		
2048	0.00028	0.00023	0.00019	0.00014	0.00010	0.00006	0.00004	0.00001	0.00000	-0.00001			
1024	0.00045	0.00038	0.00028	0.00021	0.00014	0.00008	0.00004	0.00000	-0.00002				
512	0.00068	0.00056	0.00042	0.00029	0.00018	0.00009	0.00002	-0.00004					
256	0.00093	0.00072	0.00053	0.00032	0.00017	0.00003	-0.00007						
128	0.00126	0.00095	0.00065	0.00033	0.00009	-0.00013							
64	0.00155	0.00106	0.00059	0.00016	-0.00021								
32	0.00165	0.00099	0.00038	-0.00026									
16	0.00163	0.00068	-0.00029										
8	0.00116	-0.00012											
4	0.00030												

(e) $\mathcal{P}^{\mathcal{C}}$ for Scene: [**Sponza**] Algorithm: [**M-MLT**] Metric: [**SC-QI**] True GT: [**PT @ 32768 spp**].

ERPT	2	4	8	16	32	64	128	256	512	1024	2048	4096	8192
GT (PT)													
16384	0.00001	0.00001	0.00001	0.00001	0.00001	0.00001	0.00001	0.00001	0.00000	0.00001	0.00000	0.00001	0.00001
8192	0.00001	0.00001	0.00002	0.00001	0.00001	0.00002	0.00002	0.00001	0.00001	0.00001	0.00001	0.00001	
4096	0.00002	0.00002	0.00003	0.00002	0.00002	0.00002	0.00002	0.00002	0.00002	0.00002	0.00002		
2048	0.00003	0.00003	0.00003	0.00003	0.00003	0.00003	0.00003	0.00003	0.00003	0.00003			
1024	0.00002	0.00002	0.00002	0.00002	0.00002	0.00002	0.00002	0.00002	0.00003				
512	-0.00000	-0.00000	-0.00001	-0.00000	-0.00001	-0.00001	-0.00001	-0.00000					
256	-0.00009	-0.00011	-0.00011	-0.00011	-0.00012	-0.00011	-0.00011						
128	-0.00034	-0.00036	-0.00034	-0.00035	-0.00035	-0.00035							
64	-0.00080	-0.00081	-0.00081	-0.00083	-0.00081								
32	-0.00157	-0.00159	-0.00158	-0.00159									
16	-0.00286	-0.00293	-0.00288										
8	-0.00460	-0.00464											
4	-0.00670												

(f) $\mathcal{P}^{\mathcal{C}}$ for Scene: [**Sponza**] Algorithm: [**ERPT**] Metric: [**SC-QI**] True GT: [**PT @ 32768 spp**].

M-ERPT	2	4	8	16	32	64	128	256	512	1024	2048	4096	8192
GT (PT)													
16384	0.00001	0.00001	0.00001	0.00001	0.00001	0.00001	0.00001	0.00001	0.00001	0.00000	0.00000	0.00001	0.00001
8192	0.00001	0.00002	0.00001	0.00001	0.00001	0.00001	0.00001	0.00002	0.00002	0.00002	0.00001	0.00001	
4096	0.00002	0.00003	0.00002	0.00002	0.00003	0.00003	0.00002	0.00003	0.00003	0.00003	0.00003		
2048	0.00003	0.00004	0.00003	0.00003	0.00003	0.00003	0.00004	0.00003	0.00003	0.00003			
1024	0.00003	0.00004	0.00003	0.00003	0.00003	0.00003	0.00003	0.00003	0.00003				
512	0.00000	0.00000	0.00000	0.00000	-0.00000	-0.00000	0.00000	0.00000					
256	-0.00009	-0.00009	-0.00010	-0.00009	-0.00010	-0.00009	-0.00010						
128	-0.00032	-0.00033	-0.00034	-0.00033	-0.00032	-0.00033							
64	-0.00077	-0.00078	-0.00078	-0.00080	-0.00078								
32	-0.00152	-0.00154	-0.00155	-0.00157									
16	-0.00282	-0.00284	-0.00283										
8	-0.00455	-0.00458											
4	-0.00665												

(g) $\mathcal{P}^{\mathcal{C}}$ for Scene: [**Sponza**] Algorithm: [**M-ERPT**] Metric: [**SC-QI**] True GT: [**PT @ 32768 spp**].

Table 60

14 MS-SSIM

14.1 Cornell Box

PT	2	4	8	16	32	64	128	256	512	1024	2048	4096	8192
**GT (PT)**													
16384	-0.00008	-0.00006	-0.00007	0.00003	-0.00006	-0.00005	-0.00004	-0.00004	-0.00005	-0.00005	-0.00005	-0.00005	-0.00005
8192	-0.00018	-0.00021	-0.00008	-0.00010	-0.00008	-0.00011	-0.00015	-0.00014	-0.00015	-0.00015	-0.00015	-0.00015	
4096	-0.00025	-0.00021	-0.00020	-0.00017	-0.00025	-0.00024	-0.00035	-0.00038	-0.00035	-0.00034	-0.00036		
2048	-0.00046	-0.00033	-0.00044	-0.00044	-0.00061	-0.00062	-0.00066	-0.00077	-0.00075	-0.00075			
1024	-0.00027	-0.00046	-0.00090	-0.00097	-0.00112	-0.00137	-0.00152	-0.00149	-0.00156				
512	-0.00119	-0.00103	-0.00131	-0.00202	-0.00244	-0.00256	-0.00287	-0.00294					
256	-0.00170	-0.00231	-0.00261	-0.00396	-0.00453	-0.00533	-0.00606						
128	-0.00364	-0.00475	-0.00604	-0.00768	-0.00925	-0.01059							
64	-0.00734	-0.00786	-0.01231	-0.01444	-0.01750								
32	-0.01386	-0.01716	-0.02212	-0.02846									
16	-0.02573	-0.03467	-0.04221										
8	-0.04582	-0.06503											
4	-0.08065												

(a) $\mathcal{P}^{\mathcal{C}}$ for Scene: [**Cornell Box**] Algorithm: [**PT**] Metric: [**MS-SSIM**] True GT: [**PT @ 32768 spp**].

BDPT	2	4	8	16	32	64	128	256	512	1024	2048	4096	8192
**GT (PT)**													
16384	-0.00010	-0.00000	-0.00003	-0.00006	-0.00003	-0.00008	-0.00005	-0.00005	-0.00006	-0.00005	-0.00005	-0.00005	-0.00006
8192	-0.00012	-0.00004	-0.00015	-0.00014	-0.00013	-0.00012	-0.00015	-0.00016	-0.00015	-0.00015	-0.00016	-0.00016	
4096	-0.00007	-0.00025	-0.00025	-0.00027	-0.00026	-0.00035	-0.00034	-0.00036	-0.00035	-0.00037	-0.00037		
2048	-0.00010	-0.00033	-0.00059	-0.00063	-0.00069	-0.00076	-0.00073	-0.00076	-0.00075	-0.00076			
1024	-0.00104	-0.00105	-0.00119	-0.00120	-0.00141	-0.00133	-0.00148	-0.00155	-0.00152				
512	-0.00179	-0.00159	-0.00252	-0.00266	-0.00279	-0.00294	-0.00305	-0.00310					
256	-0.00306	-0.00351	-0.00468	-0.00539	-0.00561	-0.00583	-0.00620						
128	-0.00628	-0.00800	-0.00909	-0.01011	-0.01146	-0.01181							
64	-0.01118	-0.01467	-0.01825	-0.02007	-0.02134								
32	-0.02254	-0.02859	-0.03358	-0.03739									
16	-0.04154	-0.05122	-0.05902										
8	-0.07407	-0.08862											
4	-0.12584												

(b) $\mathcal{P}^{\mathcal{C}}$ for Scene: [**Cornell Box**] Algorithm: [**BDPT**] Metric: [**MS-SSIM**] True GT: [**PT @ 32768 spp**].

PSSMLT	2	4	8	16	32	64	128	256	512	1024	2048	4096	8192
**GT (PT)**													
16384	-0.00005	0.00001	0.00002	0.00003	-0.00003	-0.00006	-0.00006	-0.00005	-0.00005	-0.00006	-0.00005	-0.00005	-0.00005
8192	-0.00012	-0.00012	-0.00009	-0.00012	-0.00014	-0.00017	-0.00014	-0.00014	-0.00014	-0.00015	-0.00015	-0.00015	
4096	-0.00016	-0.00018	-0.00025	-0.00022	-0.00029	-0.00034	-0.00034	-0.00034	-0.00036	-0.00037	-0.00037		
2048	-0.00032	-0.00039	-0.00053	-0.00055	-0.00071	-0.00071	-0.00071	-0.00073	-0.00075	-0.00077			
1024	-0.00064	-0.00091	-0.00112	-0.00114	-0.00121	-0.00140	-0.00143	-0.00150	-0.00153				
512	-0.00152	-0.00165	-0.00198	-0.00239	-0.00276	-0.00297	-0.00308	-0.00311					
256	-0.00285	-0.00388	-0.00464	-0.00516	-0.00555	-0.00592	-0.00612						
128	-0.00599	-0.00751	-0.00894	-0.01046	-0.01143	-0.01188							
64	-0.01150	-0.01449	-0.01697	-0.01949	-0.02135								
32	-0.02148	-0.02820	-0.03207	-0.03627									
16	-0.03846	-0.04870	-0.05810										
8	-0.07024	-0.08706											
4	-0.12475												

(c) $\mathcal{P}^{\mathcal{C}}$ for Scene: [**Cornell Box**] Algorithm: [**PSSMLT**] Metric: [**MS-SSIM**] True GT: [**PT @ 32768 spp**].

MLT	2	4	8	16	32	64	128	256	512	1024	2048	4096	8192
**GT (PT)**													
16384	-0.00005	-0.00002	-0.00001	0.00004	0.00005	-0.00001	-0.00002	-0.00004	-0.00005	-0.00005	-0.00004	-0.00005	-0.00005
8192	-0.00010	-0.00009	-0.00002	-0.00003	-0.00004	-0.00007	-0.00011	-0.00012	-0.00016	-0.00016	-0.00016	-0.00016	
4096	0.00000	-0.00010	-0.00007	-0.00009	-0.00022	-0.00030	-0.00036	-0.00036	-0.00038	-0.00035	-0.00035		
2048	-0.00011	-0.00003	-0.00001	-0.00002	-0.00032	-0.00052	-0.00066	-0.00074	-0.00076	-0.00075			
1024	-0.00064	-0.00074	-0.00067	-0.00092	-0.00109	-0.00130	-0.00136	-0.00144	-0.00152				
512	-0.00140	-0.00103	-0.00141	-0.00190	-0.00227	-0.00268	-0.00298	-0.00306					
256	-0.00107	-0.00234	-0.00322	-0.00408	-0.00477	-0.00510	-0.00562						
128	-0.00240	-0.00327	-0.00445	-0.00653	-0.00803	-0.00974							
64	-0.00574	-0.00828	-0.01108	-0.01433	-0.01703								
32	-0.01168	-0.01534	-0.01920	-0.02610									
16	-0.01987	-0.02820	-0.03821										
8	-0.03547	-0.05431											
4	-0.07169												

(d) $\mathcal{P}^{\mathcal{C}}$ for Scene: [**Cornell Box**] Algorithm: [**MLT**] Metric: [**MS-SSIM**] True GT: [**PT @ 32768 spp**].

M-MLT	2	4	8	16	32	64	128	256	512	1024	2048	4096	8192
**GT (PT)**													
16384	-0.00002	0.00012	0.00005	0.00004	0.00001	-0.00003	-0.00005	-0.00006	-0.00004	-0.00005	-0.00005	-0.00005	-0.00005
8192	-0.00001	0.00003	0.00001	-0.00003	-0.00006	-0.00012	-0.00010	-0.00014	-0.00017	-0.00015	-0.00015	-0.00014	
4096	-0.00009	-0.00005	-0.00008	-0.00010	-0.00022	-0.00033	-0.00036	-0.00035	-0.00034	-0.00035	-0.00036		
2048	-0.00023	-0.00017	-0.00028	-0.00043	-0.00054	-0.00058	-0.00063	-0.00074	-0.00076	-0.00078			
1024	-0.00058	-0.00034	-0.00072	-0.00106	-0.00137	-0.00145	-0.00158	-0.00160	-0.00157				
512	-0.00061	-0.00104	-0.00161	-0.00212	-0.00241	-0.00275	-0.00290	-0.00307					
256	-0.00168	-0.00214	-0.00306	-0.00394	-0.00460	-0.00531	-0.00590						
128	-0.00299	-0.00333	-0.00557	-0.00742	-0.00915	-0.01044							
64	-0.00330	-0.00754	-0.01059	-0.01399	-0.01675								
32	-0.01142	-0.01803	-0.02266	-0.02845									
16	-0.01961	-0.03067	-0.04046										
8	-0.04324	-0.05496											
4	-0.06912												

(e) $\mathcal{P}^{\mathcal{C}}$ for Scene: [**Cornell Box**] Algorithm: [**M-MLT**] Metric: [**MS-SSIM**] True GT: [**PT @ 32768 spp**].

ERPT	2	4	8	16	32	64	128	256	512	1024	2048	4096	8192
**GT (PT)**													
16384	-0.00005	-0.00007	-0.00000	-0.00005	-0.00003	-0.00002	-0.00005	-0.00005	-0.00006	-0.00003	-0.00002	-0.00005	-0.00007
8192	-0.00016	-0.00015	-0.00014	-0.00012	-0.00013	-0.00014	-0.00014	-0.00017	-0.00015	-0.00013	-0.00014	-0.00017	
4096	-0.00028	-0.00034	-0.00029	-0.00028	-0.00030	-0.00035	-0.00029	-0.00033	-0.00030	-0.00031	-0.00034		
2048	-0.00071	-0.00078	-0.00065	-0.00065	-0.00071	-0.00072	-0.00065	-0.00066	-0.00066	-0.00070			
1024	-0.00143	-0.00135	-0.00131	-0.00132	-0.00130	-0.00141	-0.00143	-0.00141	-0.00148				
512	-0.00282	-0.00276	-0.00284	-0.00281	-0.00287	-0.00303	-0.00287	-0.00295					
256	-0.00570	-0.00586	-0.00577	-0.00572	-0.00581	-0.00578	-0.00574						
128	-0.01162	-0.01148	-0.01148	-0.01132	-0.01113	-0.01157							
64	-0.02176	-0.02149	-0.02161	-0.02153	-0.02102								
32	-0.04017	-0.03966	-0.04026	-0.04012									
16	-0.07032	-0.07113	-0.07163										
8	-0.12070	-0.12012											
4	-0.19144												

(f) $\mathcal{P}^{\mathcal{C}}$ for Scene: [**Cornell Box**] Algorithm: [**ERPT**] Metric: [**MS-SSIM**] True GT: [**PT @ 32768 spp**].

M-ERPT	2	4	8	16	32	64	128	256	512	1024	2048	4096	8192
**GT (PT)**													
16384	-0.00002	-0.00006	-0.00006	-0.00003	-0.00006	-0.00009	-0.00007	-0.00007	-0.00007	-0.00003	-0.00002	-0.00006	-0.00004
8192	-0.00016	-0.00015	-0.00012	-0.00018	-0.00012	-0.00014	-0.00015	-0.00015	-0.00010	-0.00011	-0.00016	-0.00015	
4096	-0.00034	-0.00028	-0.00031	-0.00033	-0.00033	-0.00029	-0.00030	-0.00029	-0.00031	-0.00031	-0.00029		
2048	-0.00068	-0.00070	-0.00072	-0.00064	-0.00073	-0.00069	-0.00068	-0.00063	-0.00057	-0.00065			
1024	-0.00136	-0.00130	-0.00144	-0.00134	-0.00145	-0.00135	-0.00126	-0.00147	-0.00140				
512	-0.00279	-0.00274	-0.00278	-0.00288	-0.00277	-0.00292	-0.00286	-0.00279					
256	-0.00561	-0.00567	-0.00580	-0.00557	-0.00573	-0.00579	-0.00601						
128	-0.01114	-0.01120	-0.01143	-0.01135	-0.01129	-0.01130							
64	-0.02188	-0.02167	-0.02198	-0.02108	-0.02183								
32	-0.03987	-0.04024	-0.03941	-0.03954									
16	-0.07018	-0.07090	-0.07056										
8	-0.11924	-0.11978											
4	-0.19115												

(g) $\mathcal{P}^{\mathcal{C}}$ for Scene: [**Cornell Box**] Algorithm: [**M-ERPT**] Metric: [**MS-SSIM**] True GT: [**PT @ 32768 spp**].

Table 61

14.2 Torus

PT	2	4	8	16	32	64	128	256	512	1024	2048	4096	8192	16384	32768	65536	131072
GT (BDPT)																	
262144	0.00044	0.00064	0.00036	0.00001	0.00046	0.00003	0.00018	0.00009	-0.00018	-0.00014	-0.00030	-0.00024	-0.00037	-0.00034	-0.00041	-0.00055	-0.00056
131072	0.00004	0.00009	-0.00022	-0.00061	0.00027	-0.00056	-0.00041	-0.00063	-0.00061	-0.00060	-0.00045	-0.00108	-0.00121	-0.00152	-0.00166	-0.00176	
65536	-0.00048	-0.00090	-0.00112	-0.00125	-0.00075	-0.00063	-0.00036	-0.00053	-0.00106	-0.00079	-0.00181	-0.00227	-0.00289	-0.00326	-0.00329		
32768	-0.00147	-0.00227	-0.00316	-0.00367	-0.00237	-0.00239	-0.00161	-0.00241	-0.00267	-0.00280	-0.00350	-0.00490	-0.00580	-0.00672			
16384	-0.00388	-0.00652	-0.00754	-0.00799	-0.00625	-0.00622	-0.00503	-0.00554	-0.00522	-0.00659	-0.00649	-0.00958	-0.01091				
8192	-0.00893	-0.01000	-0.01191	-0.01236	-0.01125	-0.01065	-0.00887	-0.01077	-0.01008	-0.01109	-0.01268	-0.01703					
4096	-0.01553	-0.01742	-0.01932	-0.02038	-0.01524	-0.01796	-0.01598	-0.01554	-0.01602	-0.01958	-0.02275						
2048	-0.01860	-0.02433	-0.03086	-0.03068	-0.02477	-0.02588	-0.02586	-0.02548	-0.02824	-0.03132							
1024	-0.02267	-0.03026	-0.03456	-0.03563	-0.02980	-0.03329	-0.03184	-0.03524	-0.04203								
512	-0.02500	-0.03452	-0.03763	-0.04142	-0.03811	-0.03843	-0.03968	-0.04551									
256	-0.02073	-0.02928	-0.03548	-0.03345	-0.03221	-0.03844	-0.04373										
128	-0.00381	-0.01682	-0.02044	-0.02315	-0.02774	-0.03364											
64	0.00933	-0.00852	-0.01126	-0.01735	-0.02295												
32	0.02036	0.00482	-0.00094	-0.01522													
16	0.02721	0.00402	0.00015														
8	0.01980	-0.00778															
4	-0.01400																

(a) $\mathcal{P}^{\mathcal{C}}$ for Scene: [**Torus**] Algorithm: [**PT**] Metric: [**MS-SSIM**] True GT: [**BDPT @ 524288 spp**].

BDPT	2	4	8	16	32	64	128	256	512	1024	2048	4096	8192	16384	32768	65536	131072
GT (BDPT)																	
262144	0.00075	0.00054	0.00036	0.00011	-0.00020	0.00018	-0.00004	0.00018	0.00002	-0.00005	-0.00030	0.00007	-0.00035	-0.00038	-0.00040	-0.00058	-0.00058
131072	0.00026	0.00041	-0.00012	-0.00026	0.00010	-0.00020	-0.00056	-0.00017	-0.00020	-0.00054	-0.00103	-0.00088	-0.00112	-0.00135	-0.00138	-0.00166	
65536	0.00051	-0.00005	-0.00007	-0.00057	-0.00099	-0.00089	-0.00103	-0.00097	-0.00082	-0.00136	-0.00206	-0.00196	-0.00275	-0.00313	-0.00327		
32768	-0.00134	-0.00232	-0.00304	-0.00363	-0.00246	-0.00241	-0.00244	-0.00232	-0.00187	-0.00327	-0.00362	-0.00502	-0.00579	-0.00625			
16384	-0.00264	-0.00524	-0.00619	-0.00753	-0.00648	-0.00615	-0.00501	-0.00479	-0.00543	-0.00649	-0.00820	-0.00917	-0.01038				
8192	-0.00618	-0.00927	-0.01076	-0.01111	-0.00921	-0.01014	-0.01031	-0.00782	-0.00986	-0.01058	-0.01275	-0.01609					
4096	-0.01057	-0.01611	-0.01780	-0.01973	-0.01663	-0.01613	-0.01527	-0.01554	-0.01532	-0.01706	-0.02331						
2048	-0.01926	-0.02446	-0.02711	-0.02556	-0.02531	-0.02547	-0.02306	-0.02330	-0.02496	-0.03067							
1024	-0.02290	-0.02821	-0.03252	-0.03266	-0.03386	-0.03166	-0.03274	-0.03293	-0.03964								
512	-0.02370	-0.03203	-0.03945	-0.03795	-0.03904	-0.03692	-0.03888	-0.04223									
256	-0.01353	-0.02580	-0.03213	-0.03185	-0.03550	-0.04049	-0.04131										
128	-0.00316	-0.01360	-0.01969	-0.01932	-0.02477	-0.03468											
64	0.00763	0.00058	-0.00856	-0.01489	-0.02171												
32	0.02104	0.01377	-0.00237	-0.00680													
16	0.02754	0.01260	-0.00405														
8	0.02055	0.00091															
4	-0.01477																

(b) $\mathcal{P}^{\mathcal{C}}$ for Scene: [**Torus**] Algorithm: [**BDPT**] Metric: [**MS-SSIM**] True GT: [**BDPT @ 524288 spp**].

PSSMLT	2	4	8	16	32	64	128	256	512	1024	2048	4096	8192	16384
GT (BDPT)														
262144	0.00050	0.00032	0.00019	0.00034	0.00013	0.00018	0.00003	-0.00007	-0.00024	-0.00043	-0.00027	-0.00041	-0.00050	-0.00043
131072	-0.00006	0.00035	0.00018	0.00007	-0.00016	-0.00020	-0.00041	-0.00081	-0.00066	-0.00092	-0.00111	-0.00128	-0.00149	-0.00149
65536	0.00011	-0.00025	-0.00095	-0.00068	-0.00129	-0.00099	-0.00124	-0.00183	-0.00210	-0.00246	-0.00284	-0.00323	-0.00358	-0.00372
32768	-0.00077	-0.00167	-0.00216	-0.00249	-0.00269	-0.00250	-0.00287	-0.00349	-0.00472	-0.00529	-0.00593	-0.00655	-0.00718	-0.00748
16384	-0.00355	-0.00479	-0.00564	-0.00684	-0.00667	-0.00675	-0.00767	-0.00859	-0.00947	-0.01091	-0.01209	-0.01309	-0.01383	
8192	-0.00503	-0.00631	-0.00797	-0.00870	-0.01060	-0.01201	-0.01336	-0.01496	-0.01765	-0.01948	-0.02222	-0.02421		
4096	-0.00811	-0.01236	-0.01302	-0.01479	-0.01756	-0.01926	-0.02184	-0.02447	-0.02852	-0.03125	-0.03482			
2048	-0.01820	-0.02227	-0.02559	-0.02866	-0.03066	-0.03250	-0.03441	-0.04039	-0.04573	-0.05096				
1024	-0.01806	-0.02662	-0.03340	-0.03673	-0.04115	-0.04318	-0.05204	-0.05805	-0.06664					
512	-0.02681	-0.03416	-0.04211	-0.04412	-0.05042	-0.05712	-0.06663	-0.07419						
256	-0.02625	-0.03490	-0.04573	-0.05226	-0.06167	-0.07216	-0.08367							
128	-0.02437	-0.03066	-0.04450	-0.05192	-0.06603	-0.07728								
64	-0.02229	-0.02768	-0.04376	-0.05380	-0.07259									
32	-0.01705	-0.02325	-0.04538	-0.05592										
16	-0.02914	-0.03863	-0.06168											
8	-0.05328	-0.06208												
4	-0.11050													

(c) $\mathcal{P}^{\mathcal{C}}$ for Scene: [**Torus**] Algorithm: [**PSSMLT**] Metric: [**MS-SSIM**] True GT: [**BDPT @ 524288 spp**].

MLT	2	4	8	16	32	64	128	256	512	1024	2048	4096	8192	16384
GT (BDPT)														
262144	0.00045	0.00011	0.00013	-0.00006	-0.00006	-0.00004	-0.00028	-0.00045	-0.00037	-0.00041	-0.00039	-0.00043	-0.00043	-0.00028
131072	0.00028	-0.00002	-0.00073	-0.00098	-0.00096	-0.00105	-0.00101	-0.00142	-0.00141	-0.00133	-0.00146	-0.00150	-0.00149	-0.00151
65536	0.00032	-0.00094	-0.00057	-0.00163	-0.00168	-0.00182	-0.00243	-0.00284	-0.00316	-0.00332	-0.00327	-0.00338	-0.00342	-0.00357
32768	-0.00235	-0.00202	-0.00239	-0.00419	-0.00429	-0.00434	-0.00521	-0.00698	-0.00688	-0.00700	-0.00719	-0.00715	-0.00749	-0.00756
16384	-0.00266	-0.00312	-0.00358	-0.00737	-0.00810	-0.00835	-0.00959	-0.01082	-0.01140	-0.01227	-0.01242	-0.01275	-0.01288	
8192	-0.00856	-0.00862	-0.00996	-0.01358	-0.01509	-0.01642	-0.01992	-0.02103	-0.02261	-0.02321	-0.02376	-0.02406		
4096	-0.00788	-0.01162	-0.01296	-0.02448	-0.02553	-0.02680	-0.03207	-0.03557	-0.03692	-0.03771	-0.03796			
2048	-0.01695	-0.02355	-0.02416	-0.03807	-0.03999	-0.04305	-0.04774	-0.05212	-0.05482	-0.05541				
1024	-0.02463	-0.03651	-0.03340	-0.05540	-0.05773	-0.06212	-0.07161	-0.07393	-0.07794					
512	-0.03073	-0.04150	-0.04153	-0.06957	-0.07212	-0.07610	-0.08743	-0.09297						
256	-0.03334	-0.05553	-0.04986	-0.08396	-0.08267	-0.09215	-0.10585							
128	-0.02636	-0.05081	-0.04964	-0.08894	-0.08294	-0.09272								
64	-0.02554	-0.06117	-0.04947	-0.09151	-0.09347									
32	-0.01935	-0.06074	-0.04725	-0.09732										
16	-0.02202	-0.07146	-0.05723											
8	-0.06293	-0.10487												
4	-0.11471													

(d) $\mathcal{P}^{\mathcal{C}}$ for Scene: [**Torus**] Algorithm: [**MLT**] Metric: [**MS-SSIM**] True GT: [**BDPT @ 524288 spp**].

M-MLT	2	4	8	16	32	64	128	256	512	1024	2048	4096	8192	16384
GT (BDPT)														
262144	0.00027	-0.00000	-0.00035	0.00009	0.00009	-0.00033	-0.00059	-0.00077	-0.00061	-0.00058	-0.00060	-0.00070	-0.00058	-0.00054
131072	-0.00013	-0.00029	-0.00081	-0.00041	-0.00015	-0.00121	-0.00134	-0.00132	-0.00160	-0.00169	-0.00160	-0.00186	-0.00180	-0.00174
65536	-0.00009	-0.00057	-0.00180	-0.00231	-0.00142	-0.00262	-0.00319	-0.00384	-0.00361	-0.00364	-0.00374	-0.00387	-0.00395	-0.00410
32768	-0.00233	-0.00200	-0.00413	-0.00506	-0.00479	-0.00627	-0.00683	-0.00742	-0.00790	-0.00781	-0.00802	-0.00823	-0.00827	-0.00847
16384	-0.00374	-0.00359	-0.00601	-0.00632	-0.00786	-0.01068	-0.01148	-0.01280	-0.01363	-0.01376	-0.01398	-0.01418	-0.01437	
8192	-0.00823	-0.00742	-0.01130	-0.01557	-0.01568	-0.02116	-0.02135	-0.02427	-0.02687	-0.02579	-0.02700	-0.02753		
4096	-0.01316	-0.01259	-0.02220	-0.02291	-0.02395	-0.03397	-0.03521	-0.03815	-0.04090	-0.04092	-0.04169			
2048	-0.01968	-0.02055	-0.03417	-0.04013	-0.04268	-0.05333	-0.05230	-0.05763	-0.06226	-0.06110				
1024	-0.03066	-0.03648	-0.05324	-0.05906	-0.05857	-0.07673	-0.07754	-0.08193	-0.08595					
512	-0.03668	-0.04399	-0.07667	-0.07692	-0.07005	-0.09423	-0.09619	-0.10026						
256	-0.04237	-0.05604	-0.09357	-0.09421	-0.08351	-0.11580	-0.11850							
128	-0.03796	-0.05195	-0.10583	-0.09912	-0.08875	-0.12454								
64	-0.03952	-0.05292	-0.12563	-0.11206	-0.10026									
32	-0.03876	-0.05799	-0.13646	-0.11416										
16	-0.05168	-0.07647	-0.16065											
8	-0.08695	-0.10509												
4	-0.13805													

(e) $\mathcal{P}^{\mathcal{C}}$ for Scene: [**Torus**] Algorithm: [**M-MLT**] Metric: [**MS-SSIM**] True GT: [**BDPT @ 524288 spp**].

ERPT	2	4	8	16	32	64	128	256	512	1024	2048	4096	8192	16384	32768	65536	131072
GT (BDPT)																	
262144	-0.00014	-0.00026	-0.00021	-0.00032	-0.00058	-0.00046	-0.00047	-0.00039	-0.00046	-0.00034	-0.00045	-0.00042	-0.00045	-0.00046	-0.00049	-0.00049	-0.00040
131072	-0.00125	-0.00129	-0.00115	-0.00158	-0.00124	-0.00141	-0.00136	-0.00130	-0.00147	-0.00149	-0.00135	-0.00166	-0.00156	-0.00157	-0.00161	-0.00165	
65536	-0.00265	-0.00292	-0.00294	-0.00287	-0.00317	-0.00294	-0.00310	-0.00277	-0.00305	-0.00307	-0.00341	-0.00363	-0.00353	-0.00363	-0.00350		
32768	-0.00635	-0.00590	-0.00576	-0.00673	-0.00703	-0.00627	-0.00669	-0.00632	-0.00621	-0.00661	-0.00715	-0.00738	-0.00740	-0.00683			
16384	-0.01140	-0.01198	-0.01144	-0.01218	-0.01173	-0.01212	-0.01236	-0.01229	-0.01212	-0.01281	-0.01334	-0.01386	-0.01361				
8192	-0.02044	-0.02088	-0.02188	-0.02193	-0.02123	-0.02198	-0.02313	-0.02295	-0.02293	-0.02355	-0.02466	-0.02464					
4096	-0.03225	-0.03230	-0.03334	-0.03520	-0.03580	-0.03654	-0.03605	-0.03690	-0.03681	-0.03750	-0.03866						
2048	-0.04923	-0.05117	-0.05388	-0.05419	-0.05544	-0.05326	-0.05690	-0.05770	-0.05748	-0.05867							
1024	-0.06409	-0.07352	-0.07047	-0.07432	-0.07761	-0.07511	-0.08113	-0.08024	-0.08128								
512	-0.07594	-0.08704	-0.08785	-0.09543	-0.09766	-0.09482	-0.09839	-0.10003									
256	-0.08768	-0.10534	-0.10801	-0.10667	-0.11601	-0.11195	-0.12158										
128	-0.08953	-0.10666	-0.10862	-0.11793	-0.12725	-0.12365											
64	-0.09126	-0.11000	-0.11904	-0.12596	-0.13635												
32	-0.09554	-0.12144	-0.12488	-0.13221													
16	-0.10963	-0.14103	-0.14770														
8	-0.13299	-0.16782															
4	-0.19974																

(f) $\mathcal{P}^{\mathcal{C}}$ for Scene: [**Torus**] Algorithm: [**ERPT**] Metric: [**MS-SSIM**] True GT: [**BDPT @ 524288 spp**].

M-ERPT	2	4	8	16	32	64	128	256	512	1024	2048	4096	8192	16384	32768	65536	131072
GT (BDPT)																	
262144	0.00016	-0.00053	-0.00031	-0.00047	-0.00044	-0.00055	-0.00058	-0.00042	-0.00040	-0.00048	-0.00055	-0.00056	-0.00056	-0.00062	-0.00054	-0.00052	-0.00059
131072	-0.00078	-0.00162	-0.00080	-0.00099	-0.00132	-0.00121	-0.00152	-0.00161	-0.00156	-0.00144	-0.00148	-0.00154	-0.00154	-0.00157	-0.00160	-0.00156	
65536	-0.00212	-0.00262	-0.00275	-0.00307	-0.00376	-0.00289	-0.00332	-0.00347	-0.00357	-0.00341	-0.00335	-0.00352	-0.00360	-0.00371	-0.00357		
32768	-0.00472	-0.00642	-0.00527	-0.00595	-0.00645	-0.00704	-0.00700	-0.00743	-0.00722	-0.00718	-0.00742	-0.00732	-0.00744	-0.00755			
16384	-0.00933	-0.01059	-0.01187	-0.01142	-0.01137	-0.01299	-0.01329	-0.01347	-0.01323	-0.01374	-0.01375	-0.01403	-0.01427				
8192	-0.01637	-0.01876	-0.02035	-0.02110	-0.02161	-0.02274	-0.02486	-0.02400	-0.02542	-0.02488	-0.02517	-0.02460					
4096	-0.02660	-0.03089	-0.03311	-0.03543	-0.03470	-0.03712	-0.03767	-0.03861	-0.03844	-0.03914	-0.03944						
2048	-0.04129	-0.04550	-0.04958	-0.05246	-0.05366	-0.05665	-0.05937	-0.05875	-0.06009	-0.06010							
1024	-0.06101	-0.06505	-0.06866	-0.07391	-0.07634	-0.07878	-0.08510	-0.08143	-0.08441								
512	-0.06999	-0.08021	-0.08116	-0.09219	-0.09315	-0.09761	-0.10241	-0.10280									
256	-0.08130	-0.09442	-0.09783	-0.10518	-0.10599	-0.11405	-0.12288										
128	-0.08408	-0.09257	-0.10442	-0.11599	-0.11753	-0.12480											
64	-0.08784	-0.09752	-0.10285	-0.12764	-0.12052												
32	-0.08628	-0.10549	-0.11213	-0.13094													
16	-0.09728	-0.12578	-0.12784														
8	-0.11801	-0.14529															
4	-0.18923																

(g) $\mathcal{P}^{\mathcal{C}}$ for Scene: [**Torus**] Algorithm: [**M-ERPT**] Metric: [**MS-SSIM**] True GT: [**BDPT @ 524288 spp**].

Table 62

14.3 Veach Bidir

PT	2	4	8	16	32	64	128	256	512	1024	2048	4096	8192	16384	32768	65536	131072
GT (BDPT)																	
262144	0.00009	0.00005	0.00010	0.00011	0.00001	0.00003	0.00009	-0.00001	0.00005	0.00003	-0.00002	0.00004	-0.00003	0.00001	-0.00001	-0.00000	-0.00001
131072	0.00027	0.00014	0.00019	0.00009	0.00009	0.00010	0.00006	0.00002	0.00002	-0.00001	-0.00001	-0.00001	-0.00002	-0.00001	-0.00002	-0.00003	
65536	0.00013	-0.00001	0.00006	0.00006	0.00006	-0.00002	0.00011	-0.00008	-0.00006	-0.00001	0.00002	-0.00001	-0.00002	-0.00006	-0.00005		
32768	0.00027	-0.00007	0.00022	0.00017	-0.00001	0.00009	0.00006	-0.00003	-0.00008	-0.00006	-0.00005	-0.00005	-0.00013	-0.00013			
16384	0.00053	0.00064	0.00086	0.00057	0.00035	0.00032	0.00013	0.00007	0.00008	-0.00004	-0.00009	-0.00010	-0.00019				
8192	0.00089	0.00006	0.00076	0.00056	-0.00006	0.00041	0.00016	-0.00013	-0.00000	-0.00009	-0.00035	-0.00040					
4096	-0.00008	0.00048	0.00034	0.00044	0.00002	-0.00006	-0.00020	-0.00035	-0.00013	-0.00013	-0.00039						
2048	0.00118	0.00071	0.00064	0.00042	0.00039	0.00068	-0.00008	-0.00042	-0.00048	-0.00060							
1024	0.00139	0.00124	0.00169	0.00163	0.00117	0.00120	0.00015	-0.00047	-0.00015								
512	0.00265	0.00205	0.00221	0.00133	0.00084	0.00130	-0.00047	-0.00175									
256	0.00360	0.00295	0.00251	0.00196	-0.00037	0.00051	-0.00266										
128	0.00512	0.00445	0.00563	0.00141	-0.00203	-0.00052											
64	0.00472	0.00409	0.00370	-0.00012	-0.00427												
32	0.00699	0.00087	-0.00191	-0.00584													
16	0.00616	0.00002	-0.00888														
8	-0.00548	-0.01262															
4	-0.01650																

(a) $\mathcal{P}^{\mathcal{C}}$ for Scene: [**Veach Bidir**] Algorithm: [**PT**] Metric: [**MS-SSIM**] True GT: [**BDPT @ 524288 spp**].

BDPT	2	4	8	16	32	64	128	256	512	1024	2048	4096	8192	16384	32768	65536	131072
GT (BDPT)																	
262144	0.00005	0.00003	0.00003	0.00003	0.00003	0.00003	0.00005	0.00002	-0.00002	0.00003	-0.00004	-0.00003	0.00001	-0.00000	-0.00000	-0.00001	-0.00001
131072	0.00012	0.00012	0.00000	-0.00002	0.00001	0.00002	0.00001	-0.00003	-0.00003	-0.00005	-0.00005	-0.00003	-0.00001	-0.00002	-0.00002	-0.00002	
65536	0.00017	0.00008	-0.00003	-0.00006	-0.00005	-0.00010	-0.00012	-0.00008	-0.00004	-0.00008	-0.00007	-0.00004	-0.00003	-0.00008	-0.00005		
32768	0.00018	0.00003	-0.00011	-0.00012	-0.00007	-0.00020	-0.00016	-0.00012	-0.00004	-0.00011	-0.00008	-0.00015	-0.00016	-0.00013			
16384	0.00047	0.00032	0.00032	0.00011	0.00017	0.00019	0.00022	0.00001	0.00001	-0.00009	-0.00014	-0.00024	-0.00024				
8192	0.00040	0.00023	0.00056	0.00011	0.00006	0.00007	-0.00000	-0.00014	-0.00039	-0.00030	-0.00050	-0.00050					
4096	0.00002	0.00001	-0.00019	-0.00027	-0.00039	-0.00043	-0.00047	-0.00043	-0.00050	-0.00053	-0.00063						
2048	0.00045	0.00037	-0.00017	-0.00045	-0.00034	-0.00039	-0.00053	-0.00066	-0.00037	-0.00093							
1024	0.00142	0.00073	0.00087	0.00027	0.00002	0.00012	-0.00011	-0.00085	-0.00101								
512	0.00201	0.00116	0.00063	-0.00047	-0.00061	-0.00078	-0.00158	-0.00260									
256	0.00109	0.00111	-0.00029	-0.00205	-0.00225	-0.00238	-0.00338										
128	0.00161	-0.00077	-0.00305	-0.00715	-0.00658	-0.00647											
64	-0.00095	-0.00477	-0.00937	-0.01500	-0.01498												
32	-0.00751	-0.01511	-0.02255	-0.03010													
16	-0.02164	-0.03144	-0.04462														
8	-0.04541	-0.06718															
4	-0.08775																

(b) $\mathcal{P}^{\mathcal{C}}$ for Scene: [**Veach Bidir**] Algorithm: [**BDPT**] Metric: [**MS-SSIM**] True GT: [**BDPT @ 524288 spp**].

PSSMLT	2	4	8	16	32	64	128	256	512	1024	2048	4096	8192	16384
GT (BDPT)														
262144	0.00009	0.00009	0.00008	0.00007	0.00005	0.00002	0.00002	0.00001	0.00001	0.00002	0.00001	0.00002	0.00001	0.00001
131072	0.00021	0.00010	0.00006	0.00000	0.00002	0.00000	-0.00000	-0.00002	-0.00003	-0.00002	-0.00001	-0.00001	-0.00002	-0.00002
65536	0.00010	0.00004	-0.00006	-0.00004	-0.00004	-0.00009	-0.00010	-0.00012	-0.00004	-0.00003	-0.00004	-0.00005	-0.00005	-0.00006
32768	0.00007	-0.00004	-0.00008	-0.00007	-0.00015	-0.00014	-0.00012	-0.00012	-0.00011	-0.00006	-0.00007	-0.00012	-0.00013	-0.00014
16384	0.00032	0.00040	0.00025	0.00015	0.00011	0.00013	0.00011	0.00003	-0.00017	-0.00017	-0.00022	-0.00020	-0.00021	
8192	0.00048	0.00035	0.00029	0.00027	0.00019	-0.00001	0.00002	-0.00010	-0.00018	-0.00024	-0.00035	-0.00032		
4096	0.00007	-0.00019	-0.00027	-0.00036	-0.00035	-0.00042	-0.00043	-0.00052	-0.00055	-0.00049	-0.00060			
2048	0.00034	0.00030	0.00008	-0.00020	-0.00037	-0.00037	-0.00042	-0.00060	-0.00097	-0.00100				
1024	0.00139	0.00088	0.00085	0.00052	0.00025	0.00004	-0.00004	-0.00060	-0.00125					
512	0.00171	0.00122	0.00099	0.00027	-0.00040	-0.00057	-0.00077	-0.00234						
256	0.00184	0.00086	-0.00032	-0.00143	-0.00239	-0.00296	-0.00301							
128	0.00091	-0.00086	-0.00308	-0.00492	-0.00646	-0.00747								
64	-0.00123	-0.00408	-0.00746	-0.01175	-0.01460									
32	-0.01020	-0.01655	-0.02142	-0.02844										
16	-0.02330	-0.03360	-0.04455											
8	-0.04403	-0.06545												
4	-0.08926													

(c) $\mathcal{P}^{\mathcal{C}}$ for Scene: [**Veach Bidir**] Algorithm: [**PSSMLT**] Metric: [**MS-SSIM**] True GT: [**BDPT @ 524288 spp**].

MLT	2	4	8	16	32	64	128	256	512	1024	2048	4096	8192	16384
GT (BDPT)														
262144	0.00016	0.00011	0.00010	0.00007	0.00003	0.00005	0.00005	0.00004	0.00002	0.00002	0.00005	0.00005	0.00005	0.00004
131072	0.00044	0.00031	0.00024	0.00017	0.00012	0.00010	0.00005	0.00004	0.00000	-0.00001	0.00001	0.00001	0.00000	-0.00001
65536	0.00048	0.00034	0.00022	0.00023	0.00012	0.00007	-0.00003	-0.00002	-0.00006	-0.00006	-0.00003	-0.00005	-0.00005	-0.00008
32768	0.00020	0.00016	0.00015	0.00012	0.00008	0.00005	-0.00004	-0.00009	-0.00008	-0.00007	-0.00006	-0.00011	-0.00010	-0.00013
16384	0.00064	0.00061	0.00045	0.00055	0.00045	0.00034	0.00026	0.00021	0.00016	0.00015	0.00007	0.00009	0.00008	
8192	0.00034	0.00028	0.00047	0.00056	0.00029	0.00018	0.00024	0.00025	0.00019	0.00017	0.00018	0.00013		
4096	0.00062	0.00054	0.00042	0.00038	0.00019	-0.00010	-0.00029	-0.00023	-0.00035	-0.00041	-0.00046			
2048	0.00112	0.00107	0.00080	0.00044	0.00026	-0.00011	-0.00027	-0.00042	-0.00045	-0.00040				
1024	0.00126	0.00172	0.00206	0.00160	0.00062	0.00045	0.00029	0.00035	0.00004					
512	0.00368	0.00300	0.00292	0.00193	0.00052	0.00008	-0.00075	-0.00073						
256	0.00172	0.00190	0.00225	0.00151	-0.00037	-0.00121	-0.00181							
128	0.00310	0.00188	-0.00000	-0.00090	-0.00282	-0.00447								
64	0.00554	0.00288	-0.00012	-0.00489	-0.00862									
32	-0.00033	-0.00038	-0.00479	-0.01278										
16	-0.00665	-0.01213	-0.01965											
8	-0.01506	-0.02314												
4	-0.02639													

(d) $\mathcal{P}^{\mathcal{C}}$ for Scene: [**Veach Bidir**] Algorithm: [**MLT**] Metric: [**MS-SSIM**] True GT: [**BDPT @ 524288 spp**].

M-MLT	2	4	8	16	32	64	128	256	512	1024	2048	4096	8192	16384
GT (BDPT)														
262144	0.00023	0.00013	0.00015	0.00011	0.00012	0.00007	0.00010	0.00007	0.00006	0.00004	0.00002	0.00003	0.00004	0.00004
131072	0.00045	0.00033	0.00030	0.00021	0.00015	0.00006	0.00006	0.00002	0.00001	0.00000	-0.00002	-0.00000	-0.00001	-0.00000
65536	0.00022	0.00010	0.00016	0.00008	0.00011	-0.00001	0.00000	-0.00003	-0.00005	-0.00008	-0.00010	-0.00009	-0.00010	-0.00009
32768	0.00041	0.00014	0.00022	0.00004	0.00008	0.00002	-0.00001	-0.00007	-0.00010	-0.00019	-0.00013	-0.00017	-0.00016	-0.00017
16384	0.00085	0.00031	0.00024	0.00049	0.00027	0.00021	0.00023	0.00020	0.00015	0.00015	0.00009	0.00012	0.00013	
8192	0.00109	0.00087	0.00056	0.00061	0.00045	0.00044	0.00031	0.00016	0.00017	0.00003	-0.00006	0.00001		
4096	0.00058	-0.00007	-0.00000	0.00002	-0.00012	-0.00020	-0.00023	-0.00031	-0.00040	-0.00043	-0.00049			
2048	0.00060	-0.00004	0.00019	0.00044	0.00027	0.00003	-0.00025	-0.00025	-0.00034	-0.00054				
1024	0.00198	0.00195	0.00050	0.00130	0.00077	0.00072	0.00063	0.00038	0.00021					
512	0.00221	0.00255	0.00093	0.00129	0.00039	0.00010	-0.00016	-0.00075						
256	0.00301	0.00328	0.00128	0.00116	-0.00012	-0.00117	-0.00179							
128	0.00477	0.00360	0.00130	0.00025	-0.00244	-0.00429								
64	0.00495	0.00291	-0.00061	-0.00207	-0.00675									
32	0.00183	-0.00279	-0.00740	-0.01024										
16	-0.00482	-0.00583	-0.01801											
8	-0.01336	-0.01858												
4	-0.02927													

(e) $\mathcal{P}^{\mathcal{C}}$ for Scene: [**Veach Bidir**] Algorithm: [**M-MLT**] Metric: [**MS-SSIM**] True GT: [**BDPT @ 524288 spp**].

ERPT	2	4	8	16	32	64	128	256	512	1024	2048	4096	8192	16384	32768	65536	131072
GT (BDPT)																	
262144	0.00004	0.00003	0.00007	0.00001	0.00003	0.00003	0.00006	0.00002	0.00003	-0.00002	0.00000	-0.00000	-0.00001	-0.00002	-0.00001	-0.00000	0.00001
131072	0.00003	0.00004	0.00004	-0.00001	0.00001	0.00005	0.00001	0.00002	-0.00002	0.00001	0.00004	-0.00001	0.00002	-0.00001	-0.00001	-0.00000	
65536	-0.00008	-0.00006	-0.00006	-0.00011	-0.00011	-0.00002	-0.00003	-0.00003	-0.00004	-0.00006	-0.00006	-0.00004	-0.00005	-0.00003	0.00000		
32768	-0.00011	-0.00010	-0.00011	-0.00007	-0.00012	-0.00012	-0.00009	-0.00003	-0.00005	-0.00005	-0.00010	-0.00010	-0.00011	-0.00007			
16384	0.00014	0.00016	0.00019	0.00015	0.00017	-0.00013	0.00009	-0.00018	-0.00026	-0.00013	-0.00013	-0.00025	-0.00012				
8192	0.00026	0.00014	0.00013	0.00004	0.00003	-0.00025	-0.00001	-0.00002	-0.00019	-0.00041	-0.00032	-0.00039					
4096	-0.00052	-0.00029	-0.00042	-0.00032	-0.00046	-0.00033	-0.00035	-0.00037	-0.00061	-0.00042	-0.00033						
2048	-0.00035	-0.00048	-0.00015	-0.00026	-0.00020	-0.00088	-0.00053	-0.00086	-0.00098	-0.00063							
1024	0.00046	0.00030	0.00043	0.00032	0.00010	-0.00147	-0.00007	-0.00106	-0.00152								
512	-0.00015	-0.00023	-0.00038	-0.00066	-0.00131	-0.00353	-0.00149	-0.00256									
256	-0.00165	-0.00181	-0.00213	-0.00211	-0.00285	-0.00547	-0.00358										
128	-0.00620	-0.00561	-0.00660	-0.00593	-0.00752	-0.01195											
64	-0.01422	-0.01442	-0.01490	-0.01454	-0.01663												
32	-0.03233	-0.03170	-0.03348	-0.03426													
16	-0.06326	-0.06315	-0.06368														
8	-0.11638	-0.11752															
4	-0.19823																

(f) $\mathcal{P}^{\mathcal{C}}$ for Scene: [**Veach Bidir**] Algorithm: [**ERPT**] Metric: [**MS-SSIM**] True GT: [**BDPT @ 524288 spp**].

M-ERPT	2	4	8	16	32	64	128	256	512	1024	2048	4096	8192	16384	32768	65536	131072
GT (BDPT)																	
262144	0.00003	0.00007	0.00002	0.00003	0.00003	0.00001	-0.00004	0.00003	-0.00004	-0.00002	-0.00001	0.00004	-0.00001	-0.00002	-0.00004	-0.00002	0.00003
131072	0.00002	0.00001	0.00000	-0.00002	0.00000	0.00002	0.00001	0.00001	-0.00002	-0.00003	0.00001	0.00002	0.00003	-0.00001	-0.00004	-0.00001	
65536	-0.00006	-0.00003	-0.00000	-0.00005	-0.00003	-0.00004	-0.00008	-0.00004	-0.00008	-0.00003	-0.00006	-0.00001	-0.00004	-0.00004	-0.00005		
32768	-0.00003	-0.00012	-0.00008	-0.00003	-0.00008	-0.00012	-0.00015	-0.00007	-0.00008	-0.00010	-0.00009	-0.00014	-0.00009	-0.00011			
16384	0.00020	0.00016	-0.00016	-0.00019	-0.00004	-0.00008	-0.00007	-0.00023	-0.00011	-0.00029	-0.00020	-0.00021	-0.00023				
8192	0.00006	0.00017	-0.00022	-0.00029	-0.00011	-0.00018	-0.00055	-0.00023	-0.00043	-0.00038	-0.00046	-0.00031					
4096	-0.00040	-0.00033	-0.00057	-0.00057	-0.00037	-0.00049	-0.00047	-0.00062	-0.00058	-0.00051	-0.00054						
2048	-0.00003	-0.00030	-0.00090	-0.00109	-0.00076	-0.00098	-0.00050	-0.00090	-0.00046	-0.00096							
1024	0.00035	0.00060	-0.00132	-0.00100	-0.00066	-0.00150	-0.00208	-0.00169	-0.00170								
512	-0.00044	-0.00018	-0.00381	-0.00352	-0.00277	-0.00392	-0.00367	-0.00399									
256	-0.00129	-0.00161	-0.00571	-0.00657	-0.00491	-0.00650	-0.00645										
128	-0.00591	-0.00556	-0.01341	-0.01346	-0.01185	-0.01341											
64	-0.01309	-0.01471	-0.02285	-0.02347	-0.02173												
32	-0.03131	-0.03189	-0.04074	-0.04088													
16	-0.06310	-0.06272	-0.07050														
8	-0.11526	-0.11506															
4	-0.19494																

(g) $\mathcal{P}^{\mathcal{C}}$ for Scene: [**Veach Bidir**] Algorithm: [**M-ERPT**] Metric: [**MS-SSIM**] True GT: [**BDPT @ 524288 spp**].

Table 63

14.4 Veach Door

PT	2	4	8	16	32	64	128	256	512	1024	2048	4096	8192	16384	32768	65536	131072
GT (BDPT)																	
262144	0.00006	-0.00002	-0.00010	0.00004	-0.00023	0.00018	-0.00004	-0.00003	0.00010	-0.00009	-0.00006	-0.00006	-0.00008	-0.00006	-0.00006	-0.00013	-0.00009
131072	-0.00009	-0.00016	-0.00007	-0.00009	-0.00026	0.00014	-0.00023	-0.00014	-0.00015	-0.00006	-0.00011	-0.00013	-0.00014	-0.00017	-0.00021	-0.00024	
65536	-0.00019	0.00004	-0.00021	0.00022	-0.00023	0.00001	-0.00018	-0.00011	-0.00012	-0.00028	-0.00044	-0.00035	-0.00035	-0.00053	-0.00051		
32768	-0.00094	-0.00083	-0.00010	-0.00025	-0.00030	-0.00030	-0.00029	-0.00040	-0.00048	-0.00030	-0.00065	-0.00093	-0.00096	-0.00107			
16384	-0.00056	-0.00075	-0.00066	-0.00041	-0.00032	-0.00015	-0.00020	-0.00065	-0.00104	-0.00145	-0.00148	-0.00188	-0.00215				
8192	-0.00181	-0.00091	-0.00098	0.00004	-0.00077	-0.00085	-0.00050	-0.00063	-0.00175	-0.00263	-0.00337	-0.00384					
4096	-0.00436	-0.00248	-0.00169	-0.00221	-0.00195	-0.00142	-0.00095	-0.00205	-0.00397	-0.00442	-0.00634						
2048	-0.00603	-0.00327	-0.00173	-0.00213	-0.00093	-0.00218	-0.00331	-0.00442	-0.00678	-0.01045							
1024	-0.01204	-0.00403	-0.00276	-0.00202	-0.00349	-0.00336	-0.00701	-0.01219	-0.01446								
512	-0.01313	-0.01037	-0.00726	-0.00842	-0.00491	-0.00831	-0.01242	-0.01853									
256	-0.01907	-0.01065	-0.00717	-0.00956	-0.01184	-0.02239	-0.02584										
128	-0.02120	-0.01719	-0.01040	-0.01753	-0.01980	-0.03759											
64	-0.03287	-0.02234	-0.01779	-0.02751	-0.04421												
32	-0.04121	-0.03035	-0.02655	-0.04968													
16	-0.04869	-0.02574	-0.06935														
8	-0.08893	-0.08664															
4	-0.16451																

(a) $\mathcal{P}^{\mathcal{C}}$ for Scene: [**Veach Door**] Algorithm: [**PT**] Metric: [**MS-SSIM**] True GT: [**BDPT @ 524288 spp**].

BDPT	2	4	8	16	32	64	128	256	512	1024	2048	4096	8192	16384	32768	65536	131072
GT (BDPT)																	
262144	0.00005	0.00001	-0.00002	0.00009	0.00004	-0.00011	0.00005	-0.00001	-0.00001	-0.00008	-0.00008	-0.00013	-0.00009	-0.00011	-0.00012	-0.00011	-0.00011
131072	-0.00015	0.00001	0.00017	0.00011	0.00004	-0.00013	-0.00028	-0.00025	-0.00020	-0.00017	-0.00020	-0.00020	-0.00021	-0.00023	-0.00022	-0.00023	
65536	0.00009	-0.00005	0.00014	-0.00022	-0.00024	-0.00034	-0.00027	-0.00038	-0.00041	-0.00044	-0.00047	-0.00055	-0.00056	-0.00055	-0.00058		
32768	-0.00047	0.00005	-0.00002	-0.00071	-0.00002	-0.00032	-0.00045	-0.00077	-0.00086	-0.00098	-0.00104	-0.00115	-0.00121	-0.00123			
16384	-0.00045	-0.00021	-0.00064	0.00003	-0.00035	-0.00077	-0.00121	-0.00158	-0.00178	-0.00197	-0.00225	-0.00232	-0.00245				
8192	-0.00134	-0.00020	-0.00006	-0.00065	-0.00074	-0.00179	-0.00209	-0.00292	-0.00374	-0.00409	-0.00448	-0.00480					
4096	-0.00055	-0.00202	-0.00197	-0.00178	-0.00116	-0.00306	-0.00458	-0.00581	-0.00755	-0.00819	-0.00900						
2048	-0.00246	-0.00270	-0.00248	-0.00254	-0.00244	-0.00561	-0.00851	-0.01136	-0.01406	-0.01618							
1024	-0.00371	-0.00537	-0.00474	-0.00524	-0.00829	-0.01194	-0.01547	-0.02293	-0.02788								
512	-0.00984	-0.00620	-0.00964	-0.00987	-0.01291	-0.02187	-0.03095	-0.04128									
256	-0.01232	-0.00860	-0.01002	-0.01387	-0.02271	-0.03855	-0.05828										
128	-0.01657	-0.01109	-0.02199	-0.02612	-0.04573	-0.07335											
64	-0.01189	-0.01214	-0.02809	-0.04558	-0.08593												
32	-0.02292	-0.02947	-0.05139	-0.09022													
16	-0.04329	-0.07229	-0.08807														
8	-0.07103	-0.10853															
4	-0.17096																

(b) $\mathcal{P}^{\mathcal{C}}$ for Scene: [**Veach Door**] Algorithm: [**BDPT**] Metric: [**MS-SSIM**] True GT: [**BDPT @ 524288 spp**].

PSSMLT	2	4	8	16	32	64	128	256	512	1024	2048	4096	8192	16384
GT (BDPT)														
262144	0.00015	0.00005	0.00012	0.00015	0.00005	0.00001	-0.00006	-0.00003	-0.00011	-0.00012	-0.00010	-0.00011	-0.00011	-0.00012
131072	0.00003	0.00013	0.00009	0.00014	-0.00020	-0.00016	-0.00025	-0.00014	-0.00018	-0.00023	-0.00022	-0.00021	-0.00021	-0.00022
65536	-0.00009	-0.00008	-0.00004	0.00009	-0.00026	-0.00021	-0.00034	-0.00036	-0.00046	-0.00050	-0.00054	-0.00054	-0.00053	-0.00056
32768	-0.00055	-0.00050	-0.00006	-0.00036	-0.00051	-0.00081	-0.00091	-0.00098	-0.00114	-0.00123	-0.00128	-0.00127	-0.00126	-0.00125
16384	-0.00064	-0.00078	-0.00086	-0.00086	-0.00113	-0.00084	-0.00148	-0.00176	-0.00220	-0.00231	-0.00243	-0.00248	-0.00250	
8192	-0.00099	-0.00078	-0.00071	-0.00152	-0.00167	-0.00268	-0.00338	-0.00366	-0.00408	-0.00432	-0.00476	-0.00486		
4096	-0.00186	-0.00093	-0.00112	-0.00210	-0.00306	-0.00431	-0.00600	-0.00776	-0.00861	-0.00960	-0.00981			
2048	-0.00203	-0.00262	-0.00344	-0.00537	-0.00612	-0.00920	-0.01233	-0.01397	-0.01653	-0.01780				
1024	-0.00386	-0.00471	-0.00623	-0.00889	-0.01371	-0.01840	-0.02369	-0.02838	-0.03190					
512	-0.00645	-0.00862	-0.01041	-0.01805	-0.02499	-0.03453	-0.04436	-0.05309						
256	-0.01006	-0.01202	-0.01863	-0.03221	-0.04571	-0.06296	-0.08074							
128	-0.01501	-0.02310	-0.02757	-0.05060	-0.07501	-0.10862								
64	-0.02748	-0.03671	-0.05888	-0.09531	-0.13929									
32	-0.03605	-0.07020	-0.10864	-0.16154										
16	-0.06257	-0.11032	-0.18130											
8	-0.10465	-0.18977												
4	-0.25807													

(c) $\mathcal{P}^{\mathcal{C}}$ for Scene: [**Veach Door**] Algorithm: [**PSSMLT**] Metric: [**MS-SSIM**] True GT: [**BDPT @ 524288 spp**].

MLT	2	4	8	16	32	64	128	256	512	1024	2048	4096	8192	16384
GT (BDPT)														
262144	0.00009	0.00014	0.00007	-0.00010	-0.00000	-0.00006	0.00001	-0.00012	-0.00008	-0.00010	-0.00010	-0.00009	-0.00012	-0.00010
131072	0.00008	0.00008	-0.00005	-0.00001	-0.00014	-0.00021	-0.00007	-0.00020	-0.00014	-0.00019	-0.00020	-0.00020	-0.00021	-0.00021
65536	-0.00023	-0.00020	-0.00027	-0.00020	-0.00030	-0.00056	-0.00036	-0.00054	-0.00051	-0.00051	-0.00048	-0.00054	-0.00053	-0.00055
32768	-0.00009	-0.00010	-0.00049	0.00009	-0.00056	-0.00067	-0.00090	-0.00093	-0.00101	-0.00111	-0.00114	-0.00121	-0.00123	-0.00123
16384	-0.00047	-0.00057	-0.00041	-0.00081	-0.00107	-0.00136	-0.00200	-0.00208	-0.00239	-0.00237	-0.00246	-0.00244	-0.00248	
8192	-0.00054	-0.00047	-0.00144	-0.00176	-0.00274	-0.00286	-0.00399	-0.00422	-0.00481	-0.00489	-0.00498	-0.00501		
4096	-0.00194	-0.00153	-0.00156	-0.00290	-0.00446	-0.00559	-0.00733	-0.00858	-0.00900	-0.00954	-0.00991			
2048	-0.00357	-0.00171	-0.00334	-0.00636	-0.00846	-0.01165	-0.01404	-0.01646	-0.01741	-0.01837				
1024	-0.00522	-0.00619	-0.00967	-0.01162	-0.01806	-0.02422	-0.02782	-0.03117	-0.03437					
512	-0.01019	-0.00634	-0.01215	-0.01969	-0.03218	-0.04183	-0.05290	-0.05801						
256	-0.01305	-0.01582	-0.02802	-0.04168	-0.05946	-0.07772	-0.09180							
128	-0.00951	-0.02448	-0.04735	-0.06756	-0.09985	-0.13015								
64	-0.02673	-0.03445	-0.07679	-0.12143	-0.17200									
32	-0.04438	-0.08320	-0.13758	-0.19983										
16	-0.05663	-0.12699	-0.20727											
8	-0.12146	-0.21361												
4	-0.27646													

(d) $\mathcal{P}^{\mathcal{C}}$ for Scene: [**Veach Door**] Algorithm: [**MLT**] Metric: [**MS-SSIM**] True GT: [**BDPT @ 524288 spp**].

M-MLT	2	4	8	16	32	64	128	256	512	1024	2048	4096	8192	16384
GT (BDPT)														
262144	-0.00013	0.00021	0.00003	-0.00003	0.00000	-0.00001	-0.00005	-0.00006	-0.00009	-0.00009	-0.00012	-0.00010	-0.00010	-0.00011
131072	-0.00034	0.00015	0.00016	-0.00017	-0.00015	-0.00017	-0.00023	-0.00011	-0.00018	-0.00019	-0.00021	-0.00022	-0.00023	-0.00022
65536	-0.00016	-0.00004	0.00014	-0.00015	-0.00036	-0.00028	-0.00044	-0.00047	-0.00051	-0.00055	-0.00056	-0.00055	-0.00056	-0.00057
32768	-0.00047	-0.00003	-0.00019	-0.00009	-0.00052	-0.00089	-0.00103	-0.00108	-0.00104	-0.00115	-0.00117	-0.00119	-0.00123	-0.00121
16384	-0.00044	0.00016	-0.00036	-0.00105	-0.00129	-0.00132	-0.00174	-0.00194	-0.00215	-0.00237	-0.00241	-0.00252	-0.00248	
8192	-0.00061	-0.00069	-0.00048	-0.00185	-0.00232	-0.00293	-0.00367	-0.00415	-0.00438	-0.00477	-0.00497	-0.00498		
4096	-0.00212	-0.00182	-0.00161	-0.00308	-0.00434	-0.00603	-0.00762	-0.00869	-0.00921	-0.00954	-0.01004			
2048	-0.00157	-0.00224	-0.00361	-0.00605	-0.00890	-0.01215	-0.01446	-0.01596	-0.01738	-0.01853				
1024	-0.00400	-0.00374	-0.00756	-0.01038	-0.01683	-0.02196	-0.02706	-0.03186	-0.03380					
512	-0.00415	-0.00833	-0.01368	-0.01957	-0.03433	-0.04244	-0.05184	-0.05836						
256	-0.00806	-0.01423	-0.02605	-0.03757	-0.05819	-0.07711	-0.09010							
128	-0.01745	-0.02506	-0.04155	-0.07346	-0.10244	-0.13291								
64	-0.02506	-0.04593	-0.07945	-0.12655	-0.16923									
32	-0.04905	-0.08012	-0.14841	-0.19708										
16	-0.08164	-0.13128	-0.22749											
8	-0.14352	-0.22739												
4	-0.26454													

(e) $\mathcal{P}^{\mathcal{C}}$ for Scene: [**Veach Door**] Algorithm: [**M-MLT**] Metric: [**MS-SSIM**] True GT: [**BDPT @ 524288 spp**].

ERPT	2	4	8	16	32	64	128	256	512	1024	2048	4096	8192	16384	32768	65536	131072
GT (BDPT)																	
262144	-0.00003	-0.00012	-0.00011	-0.00005	-0.00013	-0.00002	-0.00008	-0.00010	-0.00013	0.00002	-0.00010	-0.00002	-0.00005	-0.00009	-0.00006	-0.00011	-0.00005
131072	-0.00017	-0.00020	-0.00025	-0.00015	-0.00023	-0.00020	-0.00020	-0.00014	-0.00025	-0.00016	-0.00019	-0.00016	-0.00019	-0.00016	-0.00016	-0.00019	
65536	-0.00056	-0.00041	-0.00042	-0.00050	-0.00055	-0.00053	-0.00041	-0.00050	-0.00045	-0.00043	-0.00048	-0.00048	-0.00046	-0.00048	-0.00047		
32768	-0.00111	-0.00101	-0.00087	-0.00105	-0.00110	-0.00086	-0.00096	-0.00087	-0.00103	-0.00089	-0.00088	-0.00087	-0.00096	-0.00098			
16384	-0.00179	-0.00213	-0.00192	-0.00221	-0.00196	-0.00203	-0.00197	-0.00210	-0.00201	-0.00219	-0.00197	-0.00206	-0.00194				
8192	-0.00352	-0.00350	-0.00392	-0.00388	-0.00369	-0.00397	-0.00393	-0.00360	-0.00357	-0.00436	-0.00397	-0.00376					
4096	-0.00781	-0.00827	-0.00792	-0.00815	-0.00755	-0.00799	-0.00821	-0.00803	-0.00803	-0.00758	-0.00796						
2048	-0.01504	-0.01486	-0.01497	-0.01573	-0.01501	-0.01500	-0.01433	-0.01463	-0.01467	-0.01438							
1024	-0.02898	-0.02914	-0.02964	-0.02991	-0.02976	-0.02970	-0.02921	-0.02955	-0.02938								
512	-0.05454	-0.05395	-0.05500	-0.05427	-0.05407	-0.05587	-0.05409	-0.05478									
256	-0.09423	-0.09493	-0.09419	-0.09467	-0.09585	-0.09625	-0.09674										
128	-0.15676	-0.16176	-0.16117	-0.16171	-0.16262	-0.15936											
64	-0.24455	-0.24286	-0.25074	-0.24869	-0.25011												
32	-0.35649	-0.36080	-0.36149	-0.36439													
16	-0.48547	-0.49121	-0.49738														
8	-0.64860	-0.65719															
4	-0.85489																

(f) $\mathcal{P}^{\mathcal{C}}$ for Scene: [**Veach Door**] Algorithm: [**ERPT**] Metric: [**MS-SSIM**] True GT: [**BDPT @ 524288 spp**].

M-ERPT	2	4	8	16	32	64	128	256	512	1024	2048	4096	8192	16384	32768	65536	131072
GT (BDPT)																	
262144	-0.00002	-0.00012	-0.00003	-0.00003	0.00000	-0.00000	-0.00016	-0.00001	-0.00013	-0.00007	-0.00001	-0.00005	-0.00008	-0.00004	-0.00007	-0.00011	-0.00012
131072	-0.00024	-0.00018	-0.00024	-0.00012	0.00005	-0.00016	-0.00013	-0.00010	-0.00021	-0.00021	0.00000	-0.00021	-0.00018	-0.00020	-0.00018	-0.00022	
65536	-0.00047	-0.00042	-0.00058	-0.00037	-0.00038	-0.00033	-0.00051	-0.00046	-0.00036	-0.00044	-0.00042	-0.00052	-0.00057	-0.00043	-0.00052		
32768	-0.00099	-0.00100	-0.00086	-0.00089	-0.00106	-0.00100	-0.00099	-0.00083	-0.00095	-0.00090	-0.00087	-0.00096	-0.00095	-0.00087			
16384	-0.00163	-0.00210	-0.00181	-0.00213	-0.00179	-0.00202	-0.00186	-0.00190	-0.00201	-0.00199	-0.00194	-0.00212	-0.00186				
8192	-0.00362	-0.00336	-0.00355	-0.00375	-0.00386	-0.00363	-0.00383	-0.00393	-0.00392	-0.00388	-0.00396	-0.00432					
4096	-0.00737	-0.00782	-0.00777	-0.00767	-0.00753	-0.00812	-0.00795	-0.00766	-0.00774	-0.00790	-0.00787						
2048	-0.01461	-0.01467	-0.01512	-0.01384	-0.01450	-0.01486	-0.01489	-0.01495	-0.01410	-0.01488							
1024	-0.02815	-0.02819	-0.02938	-0.02909	-0.02931	-0.02936	-0.02867	-0.02946	-0.02894								
512	-0.05207	-0.05232	-0.05196	-0.05353	-0.05230	-0.05397	-0.05295	-0.05300									
256	-0.09447	-0.09256	-0.09324	-0.09366	-0.09253	-0.09297	-0.09449										
128	-0.15461	-0.15559	-0.15519	-0.15648	-0.15707	-0.15677											
64	-0.24117	-0.24127	-0.24405	-0.24391	-0.24502												
32	-0.35803	-0.35923	-0.35390	-0.35808													
16	-0.48709	-0.48894	-0.48969														
8	-0.64794	-0.65559															
4	-0.85857																

(g) $\mathcal{P}^{\mathcal{C}}$ for Scene: [**Veach Door**] Algorithm: [**M-ERPT**] Metric: [**MS-SSIM**] True GT: [**BDPT @ 524288 spp**].

Table 64

14.5 Sponza

PT	2	4	8	16	32	64	128	256	512	1024	2048	4096	8192
GT (PT)													
16384	-0.00020	-0.00046	0.00020	-0.00015	-0.00005	-0.00025	-0.00023	-0.00098	-0.00059	-0.00058	-0.00062	-0.00067	-0.00063
8192	0.00078	-0.00008	-0.00035	-0.00026	-0.00067	-0.00098	-0.00098	-0.00156	-0.00193	-0.00172	-0.00184	-0.00188	
4096	-0.00005	-0.00069	-0.00159	-0.00111	-0.00190	-0.00198	-0.00235	-0.00359	-0.00400	-0.00418	-0.00427		
2048	-0.00054	0.00005	-0.00002	-0.00171	-0.00289	-0.00435	-0.00503	-0.00752	-0.00801	-0.00851			
1024	-0.00163	-0.00317	-0.00119	-0.00245	-0.00606	-0.00926	-0.01162	-0.01434	-0.01607				
512	-0.00360	-0.00617	-0.00436	-0.00711	-0.01126	-0.01652	-0.02372	-0.02671					
256	-0.00483	-0.00525	-0.00511	-0.01249	-0.02239	-0.03242	-0.04354						
128	-0.00836	-0.01038	-0.01424	-0.02334	-0.04594	-0.06474							
64	-0.00766	-0.01767	-0.03055	-0.05926	-0.08268								
32	-0.01778	-0.03539	-0.05216	-0.09740									
16	-0.04133	-0.06629	-0.09236										
8	-0.07393	-0.12274											
4	-0.13800												

(a) $\mathcal{P}^{\mathcal{C}}$ for Scene: **[Sponza]** Algorithm: **[PT]** Metric: **[MS-SSIM]** True GT: **[PT @ 32768 spp]**.

BDPT	2	4	8	16	32	64	128	256	512	1024	2048	4096	8192
GT (PT)													
16384	0.00002	0.00061	-0.00008	-0.00069	-0.00014	-0.00027	-0.00030	-0.00040	-0.00048	-0.00069	-0.00057	-0.00063	-0.00062
8192	-0.00024	0.00058	-0.00053	-0.00022	-0.00051	-0.00078	-0.00098	-0.00137	-0.00159	-0.00174	-0.00181	-0.00188	
4096	0.00017	-0.00005	-0.00006	-0.00090	-0.00226	-0.00171	-0.00230	-0.00295	-0.00360	-0.00412	-0.00444		
2048	-0.00153	-0.00064	-0.00175	-0.00116	-0.00293	-0.00366	-0.00554	-0.00702	-0.00777	-0.00870			
1024	-0.00197	0.00064	-0.00291	-0.00282	-0.00425	-0.00698	-0.01003	-0.01297	-0.01585				
512	-0.00208	-0.00163	-0.00252	-0.00792	-0.00948	-0.01449	-0.02038	-0.02600					
256	-0.00178	-0.00422	-0.00725	-0.01025	-0.01990	-0.02571	-0.03988						
128	-0.01061	-0.00843	-0.02060	-0.01913	-0.03522	-0.05712							
64	-0.00332	-0.01453	-0.02488	-0.04251	-0.07100								
32	-0.01292	-0.03339	-0.04390	-0.08277									
16	-0.04275	-0.04653	-0.08596										
8	-0.06387	-0.08496											
4	-0.10572												

(b) $\mathcal{P}^{\mathcal{C}}$ for Scene: **[Sponza]** Algorithm: **[BDPT]** Metric: **[MS-SSIM]** True GT: **[PT @ 32768 spp]**.

PSSMLT	2	4	8	16	32	64	128	256	512	1024	2048	4096	8192
GT (PT)													
16384	-0.00039	-0.00079	-0.00034	-0.00029	-0.00031	-0.00005	-0.00031	-0.00046	-0.00053	-0.00049	-0.00054	-0.00066	-0.00065
8192	-0.00093	-0.00079	-0.00076	-0.00093	-0.00082	-0.00057	-0.00115	-0.00127	-0.00138	-0.00148	-0.00168	-0.00185	
4096	-0.00102	-0.00103	-0.00077	-0.00136	-0.00179	-0.00207	-0.00275	-0.00315	-0.00358	-0.00381	-0.00397		
2048	-0.00041	-0.00105	-0.00064	-0.00179	-0.00264	-0.00330	-0.00540	-0.00662	-0.00723	-0.00795			
1024	-0.00088	-0.00048	-0.00201	-0.00391	-0.00426	-0.00753	-0.01049	-0.01287	-0.01532				
512	-0.00231	-0.00457	-0.00596	-0.00790	-0.01179	-0.01614	-0.02153	-0.02647					
256	-0.00419	-0.00515	-0.00896	-0.01621	-0.02460	-0.03137	-0.04097						
128	-0.01451	-0.01119	-0.01844	-0.02962	-0.04604	-0.06097							
64	-0.02002	-0.02714	-0.04480	-0.06161	-0.08502								
32	-0.02873	-0.04271	-0.06408	-0.10106									
16	-0.04174	-0.07505	-0.12421										
8	-0.10380	-0.13738											
4	-0.16456												

(c) $\mathcal{P}^{\mathcal{C}}$ for Scene: **[Sponza]** Algorithm: **[PSSMLT]** Metric: **[MS-SSIM]** True GT: **[PT @ 32768 spp]**.

MLT	2	4	8	16	32	64	128	256	512	1024	2048	4096	8192
GT (PT)													
16384	-0.00043	-0.00052	-0.00023	-0.00023	-0.00047	-0.00010	-0.00031	-0.00053	-0.00056	-0.00057	-0.00064	-0.00061	-0.00049
8192	0.00040	0.00017	-0.00072	0.00004	-0.00087	-0.00088	-0.00149	-0.00149	-0.00174	-0.00178	-0.00197	-0.00184	
4096	-0.00023	-0.00120	-0.00220	-0.00154	-0.00233	-0.00262	-0.00349	-0.00389	-0.00395	-0.00431	-0.00429		
2048	-0.00207	-0.00193	-0.00237	-0.00254	-0.00479	-0.00636	-0.00741	-0.00778	-0.00882	-0.00898			
1024	-0.00194	-0.00373	-0.00403	-0.00642	-0.01030	-0.01307	-0.01514	-0.01613	-0.01748				
512	-0.00441	-0.00613	-0.00824	-0.01179	-0.02132	-0.02296	-0.02912	-0.03189					
256	-0.00841	-0.01326	-0.01673	-0.03320	-0.04067	-0.04880	-0.05363						
128	-0.00927	-0.01812	-0.03083	-0.05015	-0.07025	-0.08833							
64	-0.01660	-0.03600	-0.06219	-0.09143	-0.12011								
32	-0.02898	-0.06087	-0.10714	-0.15316									
16	-0.05404	-0.11262	-0.17529										
8	-0.09758	-0.19291											
4	-0.19995												

(d) $\mathcal{P}^{\mathcal{C}}$ for Scene: **[Sponza]** Algorithm: **[MLT]** Metric: **[MS-SSIM]** True GT: **[PT @ 32768 spp]**.

M-MLT	2	4	8	16	32	64	128	256	512	1024	2048	4096	8192
GT (PT)													
16384	-0.00070	-0.00035	-0.00050	0.00006	-0.00034	-0.00038	-0.00042	-0.00051	-0.00049	-0.00063	-0.00058	-0.00060	-0.00059
8192	0.00012	0.00026	-0.00041	-0.00061	-0.00118	-0.00118	-0.00155	-0.00163	-0.00179	-0.00167	-0.00175	-0.00176	
4096	-0.00093	-0.00030	-0.00072	-0.00115	-0.00285	-0.00314	-0.00351	-0.00405	-0.00410	-0.00428	-0.00435		
2048	0.00012	-0.00129	-0.00250	-0.00376	-0.00562	-0.00677	-0.00764	-0.00810	-0.00854	-0.00868			
1024	-0.00308	-0.00289	-0.00466	-0.00658	-0.00950	-0.01341	-0.01455	-0.01652	-0.01761				
512	-0.00471	-0.00443	-0.00637	-0.01300	-0.02001	-0.02532	-0.02978	-0.03226					
256	-0.00938	-0.00904	-0.01678	-0.02648	-0.03670	-0.04817	-0.05539						
128	-0.01294	-0.01775	-0.03522	-0.05021	-0.06944	-0.08795							
64	-0.02697	-0.03415	-0.06331	-0.09421	-0.13120								
32	-0.03346	-0.06495	-0.10159	-0.15611									
16	-0.07251	-0.12458	-0.19237										
8	-0.13702	-0.20816											
4	-0.22983												

(e) $\mathcal{P}^{\mathcal{C}}$ for Scene: **[Sponza]** Algorithm: **[M-MLT]** Metric: **[MS-SSIM]** True GT: **[PT @ 32768 spp]**.

ERPT	2	4	8	16	32	64	128	256	512	1024	2048	4096	8192
GT (PT)													
16384	-0.00059	-0.00050	-0.00038	-0.00046	-0.00057	-0.00058	-0.00047	-0.00045	-0.00055	-0.00049	-0.00056	-0.00051	-0.00054
8192	-0.00160	-0.00154	-0.00147	-0.00139	-0.00182	-0.00181	-0.00139	-0.00143	-0.00154	-0.00143	-0.00154	-0.00180	
4096	-0.00378	-0.00337	-0.00343	-0.00353	-0.00349	-0.00371	-0.00345	-0.00351	-0.00369	-0.00391	-0.00345		
2048	-0.00743	-0.00758	-0.00768	-0.00747	-0.00738	-0.00793	-0.00722	-0.00784	-0.00824	-0.00760			
1024	-0.01571	-0.01561	-0.01599	-0.01583	-0.01631	-0.01597	-0.01567	-0.01490	-0.01553				
512	-0.03042	-0.03039	-0.03008	-0.02960	-0.03056	-0.03086	-0.03070	-0.03016					
256	-0.05639	-0.05665	-0.05730	-0.05589	-0.05799	-0.05716	-0.05633						
128	-0.10437	-0.10471	-0.10435	-0.10447	-0.10540	-0.10375							
64	-0.17497	-0.17388	-0.17622	-0.17507	-0.17449								
32	-0.27247	-0.26750	-0.26793	-0.26876									
16	-0.39946	-0.39989	-0.39765										
8	-0.55230	-0.55167											
4	-0.71796												

(f) $\mathcal{P}^{\mathcal{C}}$ for Scene: **[Sponza]** Algorithm: **[ERPT]** Metric: **[MS-SSIM]** True GT: **[PT @ 32768 spp]**.

M-ERPT	2	4	8	16	32	64	128	256	512	1024	2048	4096	8192
GT (PT)													
16384	-0.00045	-0.00055	-0.00048	-0.00071	-0.00055	-0.00027	-0.00044	-0.00037	-0.00032	-0.00050	-0.00062	-0.00039	-0.00033
8192	-0.00162	-0.00147	-0.00164	-0.00164	-0.00165	-0.00170	-0.00157	-0.00159	-0.00129	-0.00155	-0.00163	-0.00163	
4096	-0.00388	-0.00363	-0.00387	-0.00369	-0.00375	-0.00354	-0.00374	-0.00326	-0.00306	-0.00350	-0.00368		
2048	-0.00766	-0.00756	-0.00772	-0.00731	-0.00762	-0.00745	-0.00713	-0.00731	-0.00780	-0.00732			
1024	-0.01579	-0.01494	-0.01595	-0.01523	-0.01506	-0.01540	-0.01558	-0.01651	-0.01549				
512	-0.02942	-0.02983	-0.02977	-0.03021	-0.03018	-0.03021	-0.02976	-0.02931					
256	-0.05422	-0.05535	-0.05681	-0.05577	-0.05746	-0.05676	-0.05551						
128	-0.10235	-0.10380	-0.10222	-0.09966	-0.10371	-0.10293							
64	-0.17183	-0.17165	-0.17216	-0.17275	-0.17100								
32	-0.26468	-0.26821	-0.26539	-0.26727									
16	-0.39290	-0.39529	-0.39463										
8	-0.55191	-0.54973											
4	-0.71333												

(g) $\mathcal{P}^{\mathcal{C}}$ for Scene: **[Sponza]** Algorithm: **[M-ERPT]** Metric: **[MS-SSIM]** True GT: **[PT @ 32768 spp]**.

Table 65

15 IW-SSIM

15.1 Cornell Box

PT	2	4	8	16	32	64	128	256	512	1024	2048	4096	8192
GT (PT)													
16384	-0.00039	-0.00032	-0.00040	-0.00015	-0.00049	-0.00011	-0.00014	-0.00012	-0.00013	-0.00009	-0.00008	-0.00007	-0.00007
8192	-0.00142	-0.00127	-0.00120	-0.00096	-0.00079	-0.00055	-0.00046	-0.00037	-0.00032	-0.00026	-0.00023	-0.00021	
4096	-0.00290	-0.00299	-0.00265	-0.00186	-0.00173	-0.00132	-0.00107	-0.00088	-0.00071	-0.00059	-0.00053		
2048	-0.00598	-0.00529	-0.00448	-0.00381	-0.00315	-0.00253	-0.00189	-0.00170	-0.00141	-0.00125			
1024	-0.00918	-0.00810	-0.00754	-0.00642	-0.00503	-0.00442	-0.00384	-0.00318	-0.00289				
512	-0.01383	-0.01267	-0.01081	-0.00982	-0.00839	-0.00683	-0.00630	-0.00565					
256	-0.01852	-0.01745	-0.01547	-0.01468	-0.01250	-0.01261	-0.01263						
128	-0.02481	-0.02353	-0.02329	-0.02182	-0.02214	-0.02168							
64	-0.03397	-0.03028	-0.03556	-0.03370	-0.03619								
32	-0.04672	-0.04640	-0.05016	-0.05456									
16	-0.06598	-0.07357	-0.07973										
8	-0.09309	-0.11714											
4	-0.14044												

(a) $\mathcal{P}^{\mathcal{C}}$ for Scene: [**Cornell Box**] Algorithm: [**PT**] Metric: [**IW-SSIM**] True GT: [**PT @ 32768 spp**].

BDPT	2	4	8	16	32	64	128	256	512	1024	2048	4096	8192
GT (PT)													
16384	-0.00027	-0.00016	-0.00019	-0.00014	-0.00008	-0.00016	-0.00009	-0.00008	-0.00009	-0.00007	-0.00006	-0.00006	-0.00006
8192	-0.00083	-0.00081	-0.00072	-0.00055	-0.00041	-0.00035	-0.00028	-0.00026	-0.00023	-0.00022	-0.00020	-0.00020	
4096	-0.00163	-0.00198	-0.00145	-0.00111	-0.00089	-0.00084	-0.00069	-0.00061	-0.00053	-0.00052	-0.00049		
2048	-0.00332	-0.00322	-0.00271	-0.00240	-0.00196	-0.00176	-0.00142	-0.00125	-0.00115	-0.00109			
1024	-0.00690	-0.00607	-0.00515	-0.00406	-0.00350	-0.00285	-0.00279	-0.00263	-0.00237				
512	-0.01080	-0.00888	-0.00845	-0.00715	-0.00622	-0.00567	-0.00531	-0.00516					
256	-0.01537	-0.01417	-0.01380	-0.01250	-0.01156	-0.01067	-0.01070						
128	-0.02220	-0.02254	-0.02113	-0.02028	-0.02162	-0.02071							
64	-0.03238	-0.03556	-0.03628	-0.03771	-0.03787								
32	-0.05154	-0.05555	-0.05928	-0.06483									
16	-0.07653	-0.08847	-0.09696										
8	-0.12223	-0.13947											
4	-0.19195												

(b) $\mathcal{P}^{\mathcal{C}}$ for Scene: [**Cornell Box**] Algorithm: [**BDPT**] Metric: [**IW-SSIM**] True GT: [**PT @ 32768 spp**].

PSSMLT	2	4	8	16	32	64	128	256	512	1024	2048	4096	8192
GT (PT)													
16384	-0.00038	-0.00023	-0.00005	-0.00002	-0.00008	-0.00016	-0.00010	-0.00006	-0.00005	-0.00006	-0.00005	-0.00004	-0.00004
8192	-0.00113	-0.00104	-0.00076	-0.00062	-0.00050	-0.00044	-0.00028	-0.00021	-0.00019	-0.00016	-0.00016	-0.00015	
4096	-0.00250	-0.00221	-0.00182	-0.00125	-0.00104	-0.00089	-0.00067	-0.00056	-0.00051	-0.00046	-0.00043		
2048	-0.00472	-0.00404	-0.00339	-0.00252	-0.00212	-0.00174	-0.00145	-0.00127	-0.00113	-0.00105			
1024	-0.00793	-0.00715	-0.00603	-0.00477	-0.00373	-0.00336	-0.00279	-0.00255	-0.00231				
512	-0.01243	-0.01066	-0.00907	-0.00769	-0.00672	-0.00618	-0.00562	-0.00520					
256	-0.01714	-0.01599	-0.01483	-0.01364	-0.01204	-0.01151	-0.01094						
128	-0.02497	-0.02390	-0.02298	-0.02272	-0.02238	-0.02168							
64	-0.03621	-0.03667	-0.03661	-0.03815	-0.03937								
32	-0.05171	-0.05881	-0.05978	-0.06348									
16	-0.07700	-0.08826	-0.09983										
8	-0.11459	-0.13641											
4	-0.18986												

(c) $\mathcal{P}^{\mathcal{C}}$ for Scene: [**Cornell Box**] Algorithm: [**PSSMLT**] Metric: [**IW-SSIM**] True GT: [**PT @ 32768 spp**].

MLT	2	4	8	16	32	64	128	256	512	1024	2048	4096	8192
GT (PT)													
16384	-0.00052	-0.00053	-0.00046	-0.00030	-0.00017	-0.00015	-0.00011	-0.00013	-0.00011	-0.00009	-0.00006	-0.00006	-0.00005
8192	-0.00128	-0.00133	-0.00109	-0.00090	-0.00061	-0.00046	-0.00037	-0.00033	-0.00032	-0.00027	-0.00021	-0.00017	
4096	-0.00239	-0.00266	-0.00227	-0.00175	-0.00146	-0.00114	-0.00096	-0.00081	-0.00070	-0.00055	-0.00048		
2048	-0.00498	-0.00461	-0.00371	-0.00270	-0.00240	-0.00199	-0.00178	-0.00161	-0.00141	-0.00118			
1024	-0.00868	-0.00818	-0.00707	-0.00616	-0.00483	-0.00398	-0.00324	-0.00285	-0.00270				
512	-0.01303	-0.01161	-0.01054	-0.00904	-0.00804	-0.00696	-0.00646	-0.00585					
256	-0.01695	-0.01682	-0.01609	-0.01438	-0.01316	-0.01172	-0.01122						
128	-0.02288	-0.02130	-0.01972	-0.01878	-0.01873	-0.01952							
64	-0.03267	-0.03331	-0.03320	-0.03400	-0.03431								
32	-0.04331	-0.04450	-0.04430	-0.04958									
16	-0.05303	-0.06051	-0.07098										
8	-0.07546	-0.09926											
4	-0.12528												

(d) $\mathcal{P}^{\mathcal{C}}$ for Scene: [**Cornell Box**] Algorithm: [**MLT**] Metric: [**IW-SSIM**] True GT: [**PT @ 32768 spp**].

M-MLT	2	4	8	16	32	64	128	256	512	1024	2048	4096	8192
GT (PT)													
16384	-0.00036	-0.00019	-0.00025	-0.00009	-0.00009	-0.00011	-0.00014	-0.00015	-0.00008	-0.00008	-0.00008	-0.00006	-0.00005
8192	-0.00097	-0.00092	-0.00089	-0.00077	-0.00062	-0.00052	-0.00036	-0.00035	-0.00031	-0.00023	-0.00020	-0.00015	
4096	-0.00255	-0.00249	-0.00196	-0.00166	-0.00144	-0.00116	-0.00100	-0.00077	-0.00060	-0.00053	-0.00047		
2048	-0.00508	-0.00452	-0.00404	-0.00330	-0.00265	-0.00191	-0.00162	-0.00149	-0.00132	-0.00118			
1024	-0.00878	-0.00772	-0.00692	-0.00621	-0.00533	-0.00416	-0.00352	-0.00314	-0.00272				
512	-0.01118	-0.01153	-0.01038	-0.00948	-0.00789	-0.00682	-0.00616	-0.00554					
256	-0.01701	-0.01641	-0.01523	-0.01384	-0.01263	-0.01138	-0.01130						
128	-0.02327	-0.02187	-0.02114	-0.02084	-0.02042	-0.02029							
64	-0.02672	-0.02996	-0.03088	-0.03137	-0.03344								
32	-0.04340	-0.04804	-0.04966	-0.05423									
16	-0.05076	-0.06430	-0.07291										
8	-0.08889	-0.09639											
4	-0.11306												

(e) $\mathcal{P}^{\mathcal{C}}$ for Scene: [**Cornell Box**] Algorithm: [**M-MLT**] Metric: [**IW-SSIM**] True GT: [**PT @ 32768 spp**].

ERPT	2	4	8	16	32	64	128	256	512	1024	2048	4096	8192
GT (PT)													
16384	-0.00017	-0.00016	-0.00008	-0.00013	-0.00009	-0.00010	-0.00012	-0.00012	-0.00014	-0.00012	-0.00009	-0.00009	-0.00020
8192	-0.00044	-0.00037	-0.00037	-0.00034	-0.00034	-0.00036	-0.00040	-0.00050	-0.00041	-0.00037	-0.00038	-0.00038	
4096	-0.00081	-0.00089	-0.00085	-0.00080	-0.00082	-0.00093	-0.00076	-0.00092	-0.00082	-0.00090	-0.00085		
2048	-0.00172	-0.00190	-0.00161	-0.00161	-0.00171	-0.00167	-0.00159	-0.00165	-0.00152	-0.00173			
1024	-0.00318	-0.00281	-0.00298	-0.00309	-0.00290	-0.00309	-0.00312	-0.00327	-0.00331				
512	-0.00567	-0.00566	-0.00575	-0.00573	-0.00578	-0.00602	-0.00571	-0.00614					
256	-0.01124	-0.01101	-0.01114	-0.01069	-0.01085	-0.01094	-0.01068						
128	-0.02023	-0.01989	-0.02019	-0.02009	-0.01945	-0.02041							
64	-0.03671	-0.03656	-0.03639	-0.03740	-0.03546								
32	-0.06520	-0.06389	-0.06565	-0.06539									
16	-0.11112	-0.11269	-0.11391										
8	-0.18100	-0.17972											
4	-0.27513												

(f) $\mathcal{P}^{\mathcal{C}}$ for Scene: [**Cornell Box**] Algorithm: [**ERPT**] Metric: [**IW-SSIM**] True GT: [**PT @ 32768 spp**].

M-ERPT	2	4	8	16	32	64	128	256	512	1024	2048	4096	8192
GT (PT)													
16384	-0.00012	-0.00019	-0.00013	-0.00013	-0.00020	-0.00019	-0.00021	-0.00016	-0.00021	-0.00015	-0.00001	-0.00013	-0.00014
8192	-0.00043	-0.00045	-0.00035	-0.00049	-0.00037	-0.00040	-0.00039	-0.00040	-0.00030	-0.00033	-0.00042	-0.00037	
4096	-0.00094	-0.00081	-0.00088	-0.00092	-0.00087	-0.00080	-0.00084	-0.00073	-0.00078	-0.00077	-0.00083		
2048	-0.00166	-0.00182	-0.00183	-0.00167	-0.00171	-0.00158	-0.00168	-0.00154	-0.00133	-0.00168			
1024	-0.00310	-0.00310	-0.00329	-0.00309	-0.00322	-0.00315	-0.00292	-0.00311	-0.00298				
512	-0.00570	-0.00585	-0.00571	-0.00569	-0.00544	-0.00597	-0.00586	-0.00557					
256	-0.01048	-0.01095	-0.01113	-0.01049	-0.01082	-0.01077	-0.01123						
128	-0.02014	-0.02017	-0.02012	-0.01986	-0.01965	-0.01998							
64	-0.03764	-0.03700	-0.03781	-0.03629	-0.03719								
32	-0.06491	-0.06657	-0.06433	-0.06480									
16	-0.11029	-0.11275	-0.11107										
8	-0.17845	-0.17820											
4	-0.27502												

(g) $\mathcal{P}^{\mathcal{C}}$ for Scene: [**Cornell Box**] Algorithm: [**M-ERPT**] Metric: [**IW-SSIM**] True GT: [**PT @ 32768 spp**].

Table 66

15.2 Torus

PT	2	4	8	16	32	64	128	256	512	1024	2048	4096	8192	16384	32768	65536	131072
GT (BDPT)																	
262144	-0.00015	0.00107	0.00003	-0.00045	-0.00012	-0.00161	-0.00087	-0.00156	-0.00165	-0.00134	-0.00168	-0.00165	-0.00141	-0.00129	-0.00145	-0.00159	-0.00156
131072	-0.00087	-0.00099	-0.00089	-0.00247	-0.00081	-0.00335	-0.00279	-0.00404	-0.00414	-0.00293	-0.00311	-0.00447	-0.00406	-0.00465	-0.00481	-0.00477	
65536	-0.00277	-0.00314	-0.00233	-0.00412	-0.00366	-0.00358	-0.00353	-0.00293	-0.00355	-0.00397	-0.00681	-0.00690	-0.00827	-0.00885	-0.00883		
32768	-0.00587	-0.00592	-0.00681	-0.00907	-0.00709	-0.00582	-0.00634	-0.00623	-0.00618	-0.00700	-0.01066	-0.01352	-0.01604	-0.01844			
16384	-0.00985	-0.01311	-0.01417	-0.01620	-0.01461	-0.01407	-0.01263	-0.01183	-0.01270	-0.01704	-0.01605	-0.02407	-0.02879				
8192	-0.02108	-0.02011	-0.02180	-0.02576	-0.02658	-0.02516	-0.02275	-0.02477	-0.02319	-0.02908	-0.03434	-0.04663					
4096	-0.03089	-0.03292	-0.03574	-0.04308	-0.03134	-0.03888	-0.03978	-0.03654	-0.03905	-0.04934	-0.05905						
2048	-0.04078	-0.04873	-0.05839	-0.06474	-0.05690	-0.05657	-0.06435	-0.06556	-0.06864	-0.07237							
1024	-0.04395	-0.06155	-0.06817	-0.07584	-0.06481	-0.07352	-0.08152	-0.08934	-0.10516								
512	-0.05084	-0.06796	-0.07657	-0.08805	-0.07913	-0.08872	-0.09859	-0.10374									
256	-0.04633	-0.05693	-0.07487	-0.07448	-0.06864	-0.08702	-0.10193										
128	-0.02452	-0.04435	-0.04611	-0.05364	-0.06261	-0.07390											
64	-0.00359	-0.02766	-0.02796	-0.03762	-0.03923												
32	0.02139	0.00145	-0.00092	-0.02026													
16	0.03357	0.00784	0.01058														
8	0.02870	-0.00083															
4	-0.00847																

(a) $\mathcal{P}^{\mathcal{C}}$ for Scene: [**Torus**] Algorithm: [**PT**] Metric: [**IW-SSIM**] True GT: [**BDPT @ 524288 spp**].

BDPT	2	4	8	16	32	64	128	256	512	1024	2048	4096	8192	16384	32768	65536	131072
GT (BDPT)																	
262144	0.00047	0.00051	0.00033	-0.00088	-0.00175	-0.00036	-0.00152	-0.00110	-0.00118	-0.00148	-0.00169	-0.00058	-0.00158	-0.00157	-0.00130	-0.00178	-0.00161
131072	-0.00098	-0.00047	-0.00106	-0.00194	-0.00152	-0.00241	-0.00443	-0.00279	-0.00305	-0.00414	-0.00436	-0.00382	-0.00400	-0.00469	-0.00404	-0.00459	
65536	-0.00168	-0.00190	-0.00139	-0.00301	-0.00422	-0.00443	-0.00524	-0.00518	-0.00457	-0.00515	-0.00607	-0.00614	-0.00748	-0.00905	-0.00902		
32768	-0.00621	-0.00780	-0.00730	-0.01009	-0.00774	-0.00755	-0.00825	-0.00772	-0.00553	-0.00935	-0.01069	-0.01462	-0.01652	-0.01684			
16384	-0.00788	-0.01247	-0.01310	-0.01688	-0.01477	-0.01469	-0.01458	-0.01341	-0.01315	-0.01597	-0.02118	-0.02408	-0.02672				
8192	-0.01674	-0.02020	-0.02285	-0.02555	-0.02083	-0.02480	-0.02751	-0.01964	-0.02588	-0.02897	-0.03171	-0.04079					
4096	-0.02073	-0.03330	-0.03471	-0.04401	-0.03451	-0.04005	-0.03621	-0.04099	-0.03808	-0.03932	-0.05864						
2048	-0.04325	-0.05226	-0.05109	-0.05515	-0.05704	-0.05985	-0.05690	-0.05696	-0.06225	-0.07607							
1024	-0.04611	-0.05929	-0.06744	-0.07006	-0.07606	-0.06680	-0.08277	-0.08059	-0.09725								
512	-0.04873	-0.06748	-0.07946	-0.08018	-0.08794	-0.08036	-0.09985	-0.09441									
256	-0.03389	-0.05621	-0.06592	-0.06913	-0.07633	-0.09044	-0.09404										
128	-0.01928	-0.04042	-0.04714	-0.04234	-0.05463	-0.06918											
64	-0.00162	-0.01458	-0.02471	-0.03190	-0.03559												
32	0.02711	0.01579	-0.00184	-0.00402													
16	0.03909	0.02082	0.00685														
8	0.03774	0.01505															
4	-0.00490																

(b) $\mathcal{P}^{\mathcal{C}}$ for Scene: [**Torus**] Algorithm: [**BDPT**] Metric: [**IW-SSIM**] True GT: [**BDPT @ 524288 spp**].

PSSMLT	2	4	8	16	32	64	128	256	512	1024	2048	4096	8192	16384
GT (BDPT)														
262144	-0.00022	-0.00057	-0.00047	0.00002	-0.00053	-0.00032	-0.00055	-0.00065	-0.00088	-0.00139	-0.00081	-0.00105	-0.00116	-0.00091
131072	-0.00249	-0.00094	-0.00109	-0.00144	-0.00193	-0.00198	-0.00241	-0.00291	-0.00207	-0.00289	-0.00292	-0.00309	-0.00331	-0.00294
65536	-0.00245	-0.00296	-0.00402	-0.00325	-0.00525	-0.00415	-0.00431	-0.00567	-0.00531	-0.00582	-0.00679	-0.00692	-0.00755	-0.00757
32768	-0.00490	-0.00559	-0.00676	-0.00682	-0.00776	-0.00721	-0.00758	-0.00881	-0.01096	-0.01257	-0.01274	-0.01429	-0.01517	-0.01518
16384	-0.00786	-0.01114	-0.01257	-0.01635	-0.01667	-0.01675	-0.01751	-0.01884	-0.01988	-0.02313	-0.02554	-0.02853	-0.02938	
8192	-0.01297	-0.01425	-0.01797	-0.01971	-0.02591	-0.02863	-0.03100	-0.03403	-0.03886	-0.04291	-0.04839	-0.05306		
4096	-0.02055	-0.02843	-0.02830	-0.02939	-0.03625	-0.03981	-0.04677	-0.05243	-0.06171	-0.06831	-0.07592			
2048	-0.04747	-0.05763	-0.06277	-0.06579	-0.07086	-0.07228	-0.07509	-0.08822	-0.09827	-0.11144				
1024	-0.05521	-0.07185	-0.07907	-0.08385	-0.09214	-0.09165	-0.11246	-0.12513	-0.14455					
512	-0.06011	-0.07679	-0.08711	-0.08910	-0.10222	-0.11421	-0.13314	-0.15039						
256	-0.05466	-0.07352	-0.09691	-0.10537	-0.12372	-0.14403	-0.17060							
128	-0.04403	-0.05733	-0.08027	-0.09078	-0.11774	-0.14132								
64	-0.02939	-0.04215	-0.06946	-0.08393	-0.12115									
32	0.00915	-0.01052	-0.05083	-0.06750										
16	0.02190	-0.00269	-0.04593											
8	0.00714	-0.01832												
4	-0.04013													

(c) $\mathcal{P}^{\mathcal{C}}$ for Scene: [**Torus**] Algorithm: [**PSSMLT**] Metric: [**IW-SSIM**] True GT: [**BDPT @ 524288 spp**].

MLT	2	4	8	16	32	64	128	256	512	1024	2048	4096	8192	16384
GT (BDPT)														
262144	0.00140	0.00002	-0.00027	0.00064	-0.00034	-0.00010	-0.00033	-0.00058	-0.00026	-0.00049	-0.00032	-0.00018	-0.00027	0.00008
131072	0.00249	0.00013	-0.00253	-0.00109	-0.00173	-0.00107	-0.00087	-0.00172	-0.00128	-0.00141	-0.00171	-0.00151	-0.00143	-0.00146
65536	0.00899	0.00155	-0.00101	0.00037	-0.00252	-0.00153	-0.00275	-0.00336	-0.00388	-0.00471	-0.00448	-0.00441	-0.00427	-0.00454
32768	0.00854	0.00280	-0.00236	-0.00033	-0.00577	-0.00443	-0.00697	-0.01120	-0.00889	-0.00993	-0.01051	-0.00982	-0.01056	-0.01072
16384	0.01688	0.00522	-0.00254	-0.00417	-0.01024	-0.00980	-0.01331	-0.01562	-0.01545	-0.01831	-0.01862	-0.01928	-0.01976	
8192	0.00729	-0.00249	-0.01588	-0.01745	-0.02530	-0.02555	-0.03418	-0.03586	-0.03722	-0.03958	-0.04150	-0.04200		
4096	0.01680	-0.00641	-0.01913	-0.03782	-0.04460	-0.04300	-0.05946	-0.06527	-0.06654	-0.06997	-0.07063			
2048	0.00198	-0.03282	-0.04484	-0.06161	-0.07376	-0.07853	-0.09122	-0.10025	-0.10351	-0.10666				
1024	-0.01373	-0.05826	-0.06196	-0.09808	-0.11362	-0.11916	-0.14186	-0.14603	-0.15196					
512	-0.02387	-0.06125	-0.07472	-0.12009	-0.13706	-0.14570	-0.17176	-0.18517						
256	-0.01419	-0.07819	-0.08570	-0.14850	-0.15374	-0.17163	-0.20365							
128	0.01641	-0.05936	-0.08051	-0.13804	-0.14268	-0.15919								
64	0.03372	-0.06476	-0.06121	-0.13471	-0.15124									
32	0.08370	-0.03441	-0.03599	-0.11354										
16	0.11925	-0.01577	-0.02240											
8	0.09000	-0.03304												
4	0.06097													

(d) $\mathcal{P}^{\mathcal{C}}$ for Scene: [**Torus**] Algorithm: [**MLT**] Metric: [**IW-SSIM**] True GT: [**BDPT @ 524288 spp**].

M-MLT	2	4	8	16	32	64	128	256	512	1024	2048	4096	8192	16384
GT (BDPT)														
262144	0.00063	-0.00059	-0.00095	0.00019	-0.00035	-0.00032	-0.00125	-0.00116	-0.00085	-0.00055	-0.00086	-0.00089	-0.00061	-0.00049
131072	0.00011	-0.00135	-0.00252	-0.00043	-0.00076	-0.00147	-0.00212	-0.00174	-0.00216	-0.00211	-0.00209	-0.00237	-0.00238	-0.00227
65536	0.00486	-0.00092	-0.00349	-0.00252	-0.00254	-0.00269	-0.00447	-0.00542	-0.00447	-0.00439	-0.00507	-0.00500	-0.00527	-0.00582
32768	0.00562	-0.00057	-0.00712	-0.00554	-0.00844	-0.00818	-0.01059	-0.01105	-0.01151	-0.01129	-0.01225	-0.01231	-0.01242	-0.01310
16384	0.01094	-0.00110	-0.00861	-0.00350	-0.01202	-0.01514	-0.01776	-0.02088	-0.02165	-0.02212	-0.02331	-0.02305	-0.02366	
8192	0.00438	-0.00790	-0.01645	-0.02114	-0.02940	-0.03591	-0.03796	-0.04380	-0.04779	-0.04551	-0.04911	-0.04921		
4096	-0.00153	-0.02011	-0.03762	-0.03571	-0.04553	-0.06321	-0.06531	-0.07127	-0.07603	-0.07573	-0.07859			
2048	-0.01669	-0.03188	-0.05715	-0.07100	-0.08558	-0.10096	-0.09885	-0.11200	-0.11970	-0.11858				
1024	-0.04062	-0.07169	-0.10480	-0.10863	-0.11605	-0.15016	-0.15456	-0.16441	-0.17248					
512	-0.04707	-0.07337	-0.13487	-0.14353	-0.13565	-0.17777	-0.18632	-0.19784						
256	-0.05125	-0.09666	-0.15989	-0.16444	-0.16341	-0.21368	-0.22868							
128	-0.01937	-0.07678	-0.17083	-0.15627	-0.16048	-0.21394								
64	-0.01150	-0.06412	-0.19407	-0.17121	-0.16679									
32	0.02804	-0.04358	-0.19208	-0.14594										
16	0.04182	-0.04944	-0.20352											
8	0.03176	-0.04899												
4	-0.00121													

(e) $\mathcal{P}^{\mathcal{C}}$ for Scene: [**Torus**] Algorithm: [**M-MLT**] Metric: [**IW-SSIM**] True GT: [**BDPT @ 524288 spp**].

ERPT	2	4	8	16	32	64	128	256	512	1024	2048	4096	8192	16384	32768	65536	131072
GT (BDPT)																	
262144	-0.00011	-0.00040	-0.00059	-0.00075	-0.00161	-0.00146	-0.00123	-0.00106	-0.00089	-0.00074	-0.00099	-0.00088	-0.00105	-0.00111	-0.00107	-0.00116	-0.00088
131072	-0.00171	-0.00203	-0.00235	-0.00307	-0.00258	-0.00346	-0.00267	-0.00276	-0.00353	-0.00339	-0.00322	-0.00370	-0.00330	-0.00333	-0.00335	-0.00356	
65536	-0.00415	-0.00506	-0.00560	-0.00501	-0.00551	-0.00588	-0.00621	-0.00555	-0.00567	-0.00614	-0.00722	-0.00748	-0.00751	-0.00789	-0.00721		
32768	-0.01078	-0.00922	-0.00944	-0.01260	-0.01412	-0.01257	-0.01320	-0.01250	-0.01237	-0.01375	-0.01475	-0.01561	-0.01580	-0.01402			
16384	-0.02009	-0.02145	-0.02135	-0.02302	-0.02275	-0.02334	-0.02350	-0.02394	-0.02412	-0.02643	-0.02787	-0.02911	-0.02815				
8192	-0.03693	-0.04076	-0.04272	-0.04296	-0.04191	-0.04486	-0.04617	-0.04728	-0.04719	-0.05029	-0.05234	-0.05210					
4096	-0.06136	-0.06257	-0.06720	-0.07037	-0.07373	-0.07418	-0.07229	-0.07573	-0.07626	-0.07830	-0.08181						
2048	-0.09858	-0.10316	-0.11255	-0.10886	-0.11233	-0.10846	-0.11684	-0.11815	-0.11901	-0.12286							
1024	-0.12986	-0.15099	-0.14577	-0.15205	-0.16198	-0.15577	-0.16777	-0.16961	-0.17148								
512	-0.15138	-0.17622	-0.17692	-0.19391	-0.19829	-0.19089	-0.19732	-0.20587									
256	-0.16994	-0.20908	-0.21122	-0.20964	-0.23030	-0.22115	-0.24133										
128	-0.16219	-0.19556	-0.19977	-0.21899	-0.24064	-0.23316											
64	-0.15742	-0.19579	-0.21103	-0.22337	-0.24250												
32	-0.14717	-0.19449	-0.20368	-0.21548													
16	-0.14470	-0.19950	-0.21328														
8	-0.15655	-0.21964															
4	-0.22364																

(f) $\mathcal{P}^{\mathcal{C}}$ for Scene: [**Torus**] Algorithm: [**ERPT**] Metric: [**IW-SSIM**] True GT: [**BDPT @ 524288 spp**].

M-ERPT	2	4	8	16	32	64	128	256	512	1024	2048	4096	8192	16384	32768	65536	131072
GT (BDPT)																	
262144	-0.00000	-0.00121	-0.00089	-0.00124	-0.00105	-0.00146	-0.00130	-0.00128	-0.00080	-0.00121	-0.00133	-0.00135	-0.00133	-0.00129	-0.00135	-0.00108	-0.00138
131072	-0.00153	-0.00319	-0.00180	-0.00208	-0.00308	-0.00255	-0.00299	-0.00361	-0.00333	-0.00307	-0.00329	-0.00334	-0.00343	-0.00346	-0.00360	-0.00340	
65536	-0.00288	-0.00464	-0.00581	-0.00596	-0.00776	-0.00592	-0.00648	-0.00715	-0.00735	-0.00699	-0.00696	-0.00745	-0.00729	-0.00766	-0.00745		
32768	-0.00901	-0.01090	-0.00966	-0.01172	-0.01315	-0.01466	-0.01461	-0.01543	-0.01469	-0.01516	-0.01549	-0.01545	-0.01576	-0.01593			
16384	-0.01724	-0.02097	-0.02317	-0.02243	-0.02314	-0.02618	-0.02692	-0.02808	-0.02730	-0.02838	-0.02843	-0.02983	-0.02986				
8192	-0.03187	-0.03879	-0.04152	-0.04368	-0.04450	-0.04709	-0.05188	-0.05008	-0.05285	-0.05296	-0.05388	-0.05168					
4096	-0.05504	-0.06410	-0.06749	-0.07334	-0.07319	-0.07864	-0.07744	-0.08100	-0.08076	-0.08193	-0.08293						
2048	-0.08662	-0.09329	-0.10570	-0.10943	-0.11112	-0.11847	-0.12519	-0.12241	-0.12489	-0.12662							
1024	-0.12953	-0.13900	-0.14475	-0.15709	-0.16140	-0.16963	-0.17944	-0.17232	-0.17809								
512	-0.14748	-0.16629	-0.16770	-0.19228	-0.19211	-0.20204	-0.21091	-0.21321									
256	-0.16275	-0.19200	-0.19862	-0.21423	-0.21489	-0.23093	-0.24607										
128	-0.15410	-0.17386	-0.19784	-0.22220	-0.22200	-0.24028											
64	-0.15111	-0.17343	-0.18304	-0.23543	-0.21622												
32	-0.12918	-0.16388	-0.18047	-0.21992													
16	-0.11771	-0.17014	-0.18428														
8	-0.12687	-0.17768															
4	-0.19697																

(g) $\mathcal{P}^{\mathcal{C}}$ for Scene: [**Torus**] Algorithm: [**M-ERPT**] Metric: [**IW-SSIM**] True GT: [**BDPT @ 524288 spp**].

Table 67

15.3 Veach Bidir

PT	2	4	8	16	32	64	128	256	512	1024	2048	4096	8192	16384	32768	65536	131072
GT (BDPT)																	
262144	0.00050	0.00037	0.00049	0.00052	0.00002	0.00024	0.00023	0.00002	0.00009	0.00022	-0.00008	0.00004	0.00001	0.00001	-0.00003	0.00001	-0.00002
131072	0.00078	0.00108	0.00083	0.00075	0.00060	0.00061	0.00060	0.00036	0.00025	0.00028	0.00010	0.00005	-0.00000	0.00008	-0.00004	-0.00002	
65536	0.00106	0.00106	0.00088	0.00119	0.00088	0.00076	0.00086	0.00032	0.00015	0.00042	0.00024	0.00001	0.00003	-0.00001	-0.00011		
32768	0.00147	0.00120	0.00160	0.00138	0.00121	0.00111	0.00103	0.00063	0.00031	0.00032	0.00006	-0.00010	-0.00024	-0.00023			
16384	0.00233	0.00243	0.00274	0.00204	0.00153	0.00142	0.00115	0.00111	0.00083	0.00067	0.00004	-0.00001	-0.00040				
8192	0.00184	0.00093	0.00218	0.00181	0.00095	0.00125	0.00105	0.00055	0.00043	0.00022	-0.00042	-0.00073					
4096	-0.00106	0.00029	0.00042	0.00095	0.00033	0.00026	-0.00018	-0.00029	0.00002	-0.00026	-0.00105						
2048	-0.00110	-0.00195	-0.00097	-0.00151	-0.00021	0.00042	-0.00109	-0.00117	-0.00121	-0.00107							
1024	-0.00452	-0.00404	-0.00230	-0.00125	-0.00140	-0.00061	-0.00235	-0.00208	-0.00129								
512	-0.00662	-0.00664	-0.00502	-0.00546	-0.00485	-0.00079	-0.00598	-0.00570									
256	-0.01367	-0.01308	-0.01028	-0.00933	-0.01206	-0.00717	-0.01280										
128	-0.01906	-0.01704	-0.01021	-0.01654	-0.01964	-0.01323											
64	-0.02591	-0.02518	-0.02093	-0.02298	-0.03033												
32	-0.03122	-0.03822	-0.03431	-0.03634													
16	-0.03947	-0.04539	-0.05489														
8	-0.06490	-0.06856															
4	-0.08263																

(a) $\mathcal{P}^{\mathcal{C}}$ for Scene: [**Veach Bidir**] Algorithm: [**PT**] Metric: [**IW-SSIM**] True GT: [**BDPT @ 524288 spp**].

BDPT	2	4	8	16	32	64	128	256	512	1024	2048	4096	8192	16384	32768	65536	131072
GT (BDPT)																	
262144	0.00027	0.00006	0.00014	0.00016	0.00007	0.00006	0.00013	0.00003	-0.00007	0.00010	-0.00012	-0.00015	0.00003	-0.00003	-0.00003	-0.00002	-0.00003
131072	0.00060	0.00053	0.00036	0.00015	0.00007	0.00006	0.00002	-0.00006	-0.00010	-0.00013	-0.00015	-0.00013	-0.00003	-0.00006	-0.00006	-0.00007	
65536	0.00097	0.00073	0.00048	0.00027	0.00006	-0.00012	-0.00017	-0.00008	-0.00012	-0.00015	-0.00016	-0.00014	-0.00013	-0.00021	-0.00013		
32768	0.00126	0.00066	0.00049	0.00003	-0.00005	-0.00037	-0.00037	-0.00029	-0.00004	-0.00038	-0.00016	-0.00057	-0.00062	-0.00040			
16384	0.00196	0.00137	0.00122	0.00049	0.00055	0.00055	0.00053	0.00011	0.00025	-0.00016	-0.00026	-0.00080	-0.00065				
8192	0.00156	0.00124	0.00159	0.00054	0.00034	0.00043	0.00025	-0.00008	-0.00086	-0.00046	-0.00108	-0.00129					
4096	0.00019	0.00011	-0.00032	-0.00032	-0.00064	-0.00060	-0.00071	-0.00070	-0.00108	-0.00097	-0.00146						
2048	-0.00066	-0.00010	-0.00096	-0.00094	-0.00031	-0.00046	-0.00042	-0.00052	0.00045	-0.00139							
1024	-0.00081	-0.00040	0.00106	0.00073	0.00099	0.00206	0.00200	0.00074	0.00031								
512	-0.00152	-0.00055	-0.00010	-0.00051	0.00083	0.00206	0.00075	-0.00150									
256	-0.00762	-0.00402	-0.00338	-0.00394	-0.00243	-0.00059	-0.00207										
128	-0.00942	-0.00879	-0.00953	-0.01367	-0.00936	-0.00542											
64	-0.02049	-0.01848	-0.02090	-0.02581	-0.02250												
32	-0.03233	-0.03411	-0.04073	-0.04956													
16	-0.05605	-0.05955	-0.07468														
8	-0.08894	-0.10910															
4	-0.14469																

(b) $\mathcal{P}^{\mathcal{C}}$ for Scene: [**Veach Bidir**] Algorithm: [**BDPT**] Metric: [**IW-SSIM**] True GT: [**BDPT @ 524288 spp**].

PSSMLT	2	4	8	16	32	64	128	256	512	1024	2048	4096	8192	16384
GT (BDPT)														
262144	0.00020	0.00024	0.00025	0.00027	0.00021	0.00010	0.00004	0.00005	0.00006	0.00005	0.00005	0.00009	0.00004	0.00003
131072	0.00090	0.00044	0.00037	0.00025	0.00015	0.00011	0.00004	0.00003	-0.00001	-0.00004	0.00002	-0.00001	-0.00004	-0.00004
65536	0.00089	0.00060	0.00033	0.00031	0.00023	0.00001	-0.00008	-0.00016	-0.00002	-0.00005	-0.00009	-0.00003	-0.00007	-0.00012
32768	0.00123	0.00087	0.00057	0.00033	0.00005	-0.00001	-0.00009	-0.00012	-0.00014	-0.00008	-0.00009	-0.00027	-0.00026	-0.00025
16384	0.00163	0.00158	0.00119	0.00090	0.00060	0.00058	0.00037	0.00035	-0.00015	-0.00024	-0.00044	-0.00036	-0.00035	
8192	0.00176	0.00137	0.00111	0.00107	0.00094	0.00043	0.00040	0.00036	0.00009	-0.00020	-0.00055	-0.00031		
4096	0.00045	0.00004	-0.00008	-0.00026	-0.00024	-0.00036	-0.00051	-0.00075	-0.00080	-0.00067	-0.00092			
2048	-0.00084	-0.00047	-0.00023	-0.00025	-0.00037	-0.00007	0.00006	0.00005	-0.00099	-0.00097				
1024	-0.00089	-0.00057	0.00037	0.00105	0.00173	0.00193	0.00214	0.00123	-0.00088					
512	-0.00236	-0.00129	0.00060	0.00118	0.00137	0.00220	0.00274	-0.00058						
256	-0.00621	-0.00433	-0.00351	-0.00256	-0.00239	-0.00184	-0.00054							
128	-0.01280	-0.01063	-0.00978	-0.00927	-0.00920	-0.00907								
64	-0.01976	-0.01751	-0.01754	-0.01964	-0.02178									
32	-0.03572	-0.03790	-0.03770	-0.04498										
16	-0.05926	-0.06489	-0.07372											
8	-0.08551	-0.10667												
4	-0.14075													

(c) $\mathcal{P}^{\mathcal{C}}$ for Scene: [**Veach Bidir**] Algorithm: [**PSSMLT**] Metric: [**IW-SSIM**] True GT: [**BDPT @ 524288 spp**].

MLT	2	4	8	16	32	64	128	256	512	1024	2048	4096	8192	16384
GT (BDPT)														
262144	0.00039	0.00025	0.00015	0.00012	0.00015	0.00012	0.00005	0.00006	0.00001	0.00003	0.00010	0.00012	0.00012	0.00012
131072	0.00165	0.00099	0.00083	0.00055	0.00037	0.00026	0.00013	0.00009	-0.00002	-0.00002	-0.00002	0.00002	-0.00000	-0.00001
65536	0.00171	0.00135	0.00089	0.00083	0.00058	0.00041	0.00008	0.00008	-0.00005	-0.00006	0.00004	-0.00000	0.00001	-0.00002
32768	0.00131	0.00087	0.00079	0.00070	0.00066	0.00051	0.00010	-0.00007	-0.00011	-0.00009	-0.00009	-0.00023	-0.00017	-0.00021
16384	0.00212	0.00162	0.00161	0.00144	0.00117	0.00093	0.00057	0.00046	0.00038	0.00036	0.00010	0.00027	0.00032	
8192	0.00140	0.00118	0.00140	0.00153	0.00109	0.00075	0.00079	0.00080	0.00063	0.00071	0.00084	0.00086		
4096	0.00020	-0.00001	0.00019	0.00033	0.00042	0.00003	-0.00036	-0.00016	-0.00031	-0.00041	-0.00041			
2048	-0.00057	-0.00076	-0.00034	-0.00067	-0.00017	-0.00025	-0.00019	-0.00029	-0.00002	0.00034				
1024	-0.00332	-0.00195	0.00012	0.00055	0.00074	0.00152	0.00192	0.00258	0.00239					
512	-0.00342	-0.00343	-0.00096	-0.00048	-0.00056	0.00128	0.00115	0.00224						
256	-0.01212	-0.00988	-0.00581	-0.00332	-0.00335	-0.00205	-0.00071							
128	-0.01573	-0.01468	-0.01354	-0.00980	-0.00861	-0.00756								
64	-0.01326	-0.01496	-0.01489	-0.01628	-0.01838									
32	-0.03128	-0.02771	-0.02715	-0.03225										
16	-0.04411	-0.04768	-0.04943											
8	-0.05113	-0.05928												
4	-0.06699													

(d) $\mathcal{P}^{\mathcal{C}}$ for Scene: [**Veach Bidir**] Algorithm: [**MLT**] Metric: [**IW-SSIM**] True GT: [**BDPT @ 524288 spp**].

M-MLT	2	4	8	16	32	64	128	256	512	1024	2048	4096	8192	16384
GT (BDPT)														
262144	0.00059	0.00008	0.00036	0.00017	0.00017	0.00007	0.00009	0.00008	0.00007	0.00008	0.00005	0.00007	0.00009	0.00010
131072	0.00132	0.00081	0.00077	0.00073	0.00045	0.00016	0.00016	0.00010	0.00006	0.00002	-0.00008	0.00000	-0.00004	-0.00001
65536	0.00116	0.00069	0.00076	0.00050	0.00040	0.00009	0.00013	0.00010	-0.00000	-0.00010	-0.00019	-0.00009	-0.00011	-0.00006
32768	0.00127	0.00066	0.00100	0.00046	0.00038	0.00020	-0.00001	-0.00004	-0.00013	-0.00039	-0.00023	-0.00034	-0.00029	-0.00034
16384	0.00198	0.00093	0.00138	0.00155	0.00092	0.00063	0.00044	0.00046	0.00031	0.00042	0.00030	0.00042	0.00056	
8192	0.00233	0.00189	0.00151	0.00148	0.00114	0.00095	0.00074	0.00058	0.00064	0.00044	0.00030	0.00063		
4096	0.00062	-0.00077	-0.00014	0.00001	-0.00024	-0.00025	-0.00030	-0.00029	-0.00051	-0.00043	-0.00062			
2048	-0.00121	-0.00255	-0.00091	0.00004	0.00018	-0.00003	-0.00023	0.00022	0.00007	-0.00019				
1024	-0.00226	-0.00170	-0.00287	0.00021	0.00026	0.00163	0.00219	0.00267	0.00272					
512	-0.00514	-0.00438	-0.00442	-0.00104	-0.00030	0.00054	0.00177	0.00220						
256	-0.00907	-0.00711	-0.00747	-0.00460	-0.00254	-0.00189	-0.00063							
128	-0.01324	-0.01271	-0.01040	-0.00702	-0.00610	-0.00600								
64	-0.01664	-0.01665	-0.01854	-0.01294	-0.01483									
32	-0.02763	-0.02976	-0.03247	-0.02647										
16	-0.03963	-0.03555	-0.04871											
8	-0.05134	-0.05377												
4	-0.07286													

(e) $\mathcal{P}^{\mathcal{C}}$ for Scene: [**Veach Bidir**] Algorithm: [**M-MLT**] Metric: [**IW-SSIM**] True GT: [**BDPT @ 524288 spp**].

ERPT	2	4	8	16	32	64	128	256	512	1024	2048	4096	8192	16384	32768	65536	131072
GT (BDPT)																	
262144	0.00015	0.00008	0.00015	0.00002	0.00008	0.00004	0.00004	0.00002	0.00007	-0.00002	0.00002	0.00006	0.00003	-0.00005	-0.00001	-0.00004	0.00009
131072	0.00022	0.00020	0.00012	0.00001	0.00006	0.00016	0.00008	0.00013	0.00001	0.00011	0.00008	0.00012	0.00011	0.00003	0.00010	0.00007	
65536	0.00007	0.00006	0.00004	-0.00005	-0.00005	-0.00000	0.00011	0.00008	0.00001	-0.00006	-0.00007	-0.00001	0.00002	0.00003	0.00009		
32768	0.00001	0.00001	-0.00005	0.00004	-0.00007	-0.00023	-0.00001	0.00008	-0.00001	0.00007	-0.00017	-0.00009	-0.00009	-0.00001			
16384	0.00053	0.00058	0.00057	0.00036	0.00054	-0.00008	0.00037	-0.00012	-0.00033	-0.00010	-0.00021	-0.00025	-0.00007				
8192	0.00090	0.00069	0.00070	0.00042	0.00033	-0.00016	0.00024	0.00033	-0.00013	-0.00054	-0.00046	-0.00044					
4096	-0.00053	-0.00017	-0.00041	-0.00032	-0.00054	-0.00063	-0.00027	-0.00041	-0.00089	-0.00054	-0.00056						
2048	-0.00008	-0.00050	0.00044	0.00001	0.00022	-0.00116	-0.00075	-0.00117	-0.00171	-0.00108							
1024	0.00221	0.00172	0.00232	0.00217	0.00185	-0.00252	0.00097	-0.00173	-0.00300								
512	0.00233	0.00233	0.00191	0.00160	0.00089	-0.00693	-0.00111	-0.00476									
256	-0.00020	-0.00001	-0.00093	-0.00113	-0.00246	-0.01018	-0.00452										
128	-0.00633	-0.00589	-0.00748	-0.00609	-0.00966	-0.02116											
64	-0.01784	-0.01840	-0.02001	-0.01845	-0.02336												
32	-0.04542	-0.04449	-0.04720	-0.04907													
16	-0.08978	-0.09070	-0.09082														
8	-0.16240	-0.16816															
4	-0.27420																

(f) $\mathcal{P}^{\mathcal{C}}$ for Scene: [**Veach Bidir**] Algorithm: [**ERPT**] Metric: [**IW-SSIM**] True GT: [**BDPT @ 524288 spp**].

M-ERPT	2	4	8	16	32	64	128	256	512	1024	2048	4096	8192	16384	32768	65536	131072
GT (BDPT)																	
262144	0.00007	0.00011	0.00003	0.00009	0.00005	-0.00001	-0.00010	0.00004	-0.00008	-0.00001	-0.00004	0.00006	-0.00003	-0.00001	-0.00005	-0.00003	0.00011
131072	0.00012	0.00013	0.00012	0.00006	0.00005	0.00010	0.00007	0.00013	0.00003	0.00002	0.00015	0.00014	0.00012	0.00011	0.00008	0.00007	
65536	0.00004	0.00012	0.00014	0.00000	0.00010	0.00006	-0.00011	0.00005	-0.00008	0.00012	0.00006	0.00014	0.00008	0.00009	0.00009		
32768	0.00010	-0.00018	-0.00004	-0.00005	-0.00001	-0.00018	-0.00025	0.00011	0.00004	0.00004	0.00006	-0.00011	0.00001	0.00003			
16384	0.00058	0.00044	-0.00009	-0.00009	0.00019	0.00001	0.00009	-0.00019	-0.00001	-0.00031	-0.00013	-0.00026	-0.00016				
8192	0.00035	0.00072	-0.00010	-0.00026	0.00023	-0.00009	-0.00098	-0.00012	-0.00060	-0.00051	-0.00058	-0.00030					
4096	-0.00062	-0.00030	-0.00087	-0.00089	-0.00053	-0.00071	-0.00084	-0.00087	-0.00090	-0.00070	-0.00089						
2048	0.00044	-0.00010	-0.00127	-0.00176	-0.00076	-0.00176	-0.00093	-0.00127	-0.00063	-0.00151							
1024	0.00179	0.00255	-0.00326	-0.00201	-0.00089	-0.00323	-0.00436	-0.00349	-0.00328								
512	0.00212	0.00207	-0.00860	-0.00777	-0.00483	-0.00919	-0.00825	-0.00865									
256	0.00020	0.00004	-0.01173	-0.01296	-0.00819	-0.01336	-0.01280										
128	-0.00582	-0.00617	-0.02625	-0.02526	-0.02063	-0.02632											
64	-0.01700	-0.01910	-0.04190	-0.04099	-0.03750												
32	-0.04368	-0.04556	-0.06699	-0.06664													
16	-0.08939	-0.08915	-0.11050														
8	-0.16207	-0.16269															
4	-0.26881																

(g) $\mathcal{P}^{\mathcal{C}}$ for Scene: [**Veach Bidir**] Algorithm: [**M-ERPT**] Metric: [**IW-SSIM**] True GT: [**BDPT @ 524288 spp**].

Table 68

15.4 Veach Door

PT	2	4	8	16	32	64	128	256	512	1024	2048	4096	8192	16384	32768	65536	131072
GT (BDPT)																	
262144	-0.00028	-0.00055	-0.00065	-0.00041	-0.00070	-0.00057	-0.00067	-0.00056	-0.00044	-0.00048	-0.00045	-0.00031	-0.00025	-0.00020	-0.00013	-0.00018	-0.00012
131072	-0.00117	-0.00160	-0.00170	-0.00169	-0.00202	-0.00164	-0.00200	-0.00180	-0.00163	-0.00117	-0.00111	-0.00080	-0.00056	-0.00044	-0.00037	-0.00032	
65536	-0.00278	-0.00332	-0.00395	-0.00386	-0.00418	-0.00443	-0.00454	-0.00402	-0.00376	-0.00319	-0.00279	-0.00183	-0.00143	-0.00118	-0.00087		
32768	-0.00614	-0.00731	-0.00755	-0.00777	-0.00833	-0.00846	-0.00838	-0.00778	-0.00668	-0.00578	-0.00492	-0.00398	-0.00291	-0.00214			
16384	-0.01030	-0.01180	-0.01314	-0.01385	-0.01400	-0.01399	-0.01383	-0.01274	-0.01188	-0.01064	-0.00861	-0.00683	-0.00563				
8192	-0.01678	-0.01765	-0.02012	-0.02068	-0.02115	-0.02103	-0.02060	-0.01838	-0.01722	-0.01536	-0.01338	-0.01115					
4096	-0.02512	-0.02703	-0.02824	-0.03074	-0.03095	-0.02951	-0.02880	-0.02749	-0.02581	-0.02324	-0.02053						
2048	-0.03482	-0.03588	-0.03768	-0.04082	-0.04023	-0.04123	-0.03943	-0.03715	-0.03448	-0.03379							
1024	-0.04861	-0.04535	-0.04829	-0.05122	-0.05262	-0.05231	-0.05129	-0.05197	-0.05115								
512	-0.06002	-0.06129	-0.06407	-0.06734	-0.06530	-0.06516	-0.06541	-0.06593									
256	-0.07446	-0.07035	-0.07619	-0.07691	-0.08243	-0.09087	-0.08874										
128	-0.08800	-0.08754	-0.08832	-0.09315	-0.09807	-0.11072											
64	-0.11489	-0.10331	-0.09972	-0.11609	-0.12711												
32	-0.12740	-0.12757	-0.12874	-0.14820													
16	-0.14764	-0.13348	-0.17737														
8	-0.21738	-0.21138															
4	-0.28235																

(a) $\mathcal{P}^{\mathcal{C}}$ for Scene: [**Veach Door**] Algorithm: [**PT**] Metric: [**IW-SSIM**] True GT: [**BDPT @ 524288 spp**].

BDPT	2	4	8	16	32	64	128	256	512	1024	2048	4096	8192	16384	32768	65536	131072
GT (BDPT)																	
262144	-0.00035	-0.00058	-0.00065	-0.00052	-0.00054	-0.00078	-0.00037	-0.00038	-0.00030	-0.00031	-0.00019	-0.00019	-0.00014	-0.00013	-0.00013	-0.00012	-0.00010
131072	-0.00156	-0.00159	-0.00175	-0.00173	-0.00187	-0.00193	-0.00169	-0.00117	-0.00104	-0.00060	-0.00050	-0.00036	-0.00031	-0.00027	-0.00022	-0.00021	
65536	-0.00329	-0.00384	-0.00437	-0.00458	-0.00450	-0.00419	-0.00334	-0.00291	-0.00206	-0.00151	-0.00115	-0.00093	-0.00073	-0.00060	-0.00056		
32768	-0.00679	-0.00744	-0.00782	-0.00881	-0.00805	-0.00752	-0.00610	-0.00521	-0.00390	-0.00301	-0.00223	-0.00183	-0.00149	-0.00133			
16384	-0.01212	-0.01285	-0.01443	-0.01391	-0.01378	-0.01258	-0.01095	-0.00923	-0.00674	-0.00501	-0.00419	-0.00323	-0.00296				
8192	-0.01887	-0.01935	-0.02027	-0.02115	-0.02063	-0.01873	-0.01591	-0.01366	-0.01093	-0.00842	-0.00725	-0.00644					
4096	-0.02482	-0.02885	-0.03097	-0.02998	-0.02887	-0.02635	-0.02448	-0.02097	-0.01764	-0.01517	-0.01366						
2048	-0.03573	-0.03863	-0.04002	-0.04112	-0.03819	-0.03773	-0.03518	-0.03129	-0.02843	-0.02550							
1024	-0.04596	-0.05009	-0.05354	-0.05247	-0.05241	-0.04988	-0.04578	-0.04894	-0.04734								
512	-0.06150	-0.06175	-0.06600	-0.06869	-0.06649	-0.06945	-0.06880	-0.07350									
256	-0.07419	-0.07432	-0.07721	-0.07909	-0.08283	-0.09297	-0.10310										
128	-0.09173	-0.08729	-0.09941	-0.10189	-0.11427	-0.13469											
64	-0.10119	-0.09684	-0.11919	-0.12983	-0.16120												
32	-0.11967	-0.12022	-0.14832	-0.18215													
16	-0.15944	-0.19262	-0.18991														
8	-0.19564	-0.23152															
4	-0.29790																

(b) $\mathcal{P}^{\mathcal{C}}$ for Scene: [**Veach Door**] Algorithm: [**BDPT**] Metric: [**IW-SSIM**] True GT: [**BDPT @ 524288 spp**].

PSSMLT	2	4	8	16	32	64	128	256	512	1024	2048	4096	8192	16384
GT (BDPT)														
262144	-0.00057	-0.00058	-0.00055	-0.00042	-0.00051	-0.00041	-0.00036	-0.00028	-0.00036	-0.00026	-0.00018	-0.00017	-0.00011	-0.00014
131072	-0.00164	-0.00155	-0.00171	-0.00154	-0.00178	-0.00151	-0.00123	-0.00077	-0.00063	-0.00049	-0.00035	-0.00025	-0.00022	-0.00022
65536	-0.00387	-0.00409	-0.00400	-0.00361	-0.00359	-0.00286	-0.00241	-0.00184	-0.00143	-0.00104	-0.00087	-0.00066	-0.00055	-0.00055
32768	-0.00793	-0.00815	-0.00782	-0.00753	-0.00693	-0.00609	-0.00492	-0.00374	-0.00287	-0.00220	-0.00184	-0.00149	-0.00127	-0.00115
16384	-0.01313	-0.01342	-0.01354	-0.01271	-0.01190	-0.00962	-0.00825	-0.00637	-0.00532	-0.00412	-0.00353	-0.00301	-0.00264	
8192	-0.01998	-0.01971	-0.01947	-0.01886	-0.01730	-0.01557	-0.01298	-0.01027	-0.00844	-0.00686	-0.00629	-0.00556		
4096	-0.02854	-0.02777	-0.02735	-0.02586	-0.02381	-0.02088	-0.01844	-0.01675	-0.01452	-0.01371	-0.01207			
2048	-0.03771	-0.03817	-0.03781	-0.03648	-0.03363	-0.03151	-0.02970	-0.02706	-0.02628	-0.02428				
1024	-0.04807	-0.04975	-0.05069	-0.04918	-0.04891	-0.04775	-0.04564	-0.04587	-0.04499					
512	-0.06126	-0.06386	-0.06391	-0.06703	-0.06599	-0.06694	-0.06966	-0.07337						
256	-0.07451	-0.07575	-0.07763	-0.08619	-0.09363	-0.10160	-0.11378							
128	-0.08858	-0.09715	-0.09432	-0.10926	-0.12181	-0.14851								
64	-0.11805	-0.12233	-0.13510	-0.15859	-0.19077									
32	-0.12327	-0.15746	-0.18640	-0.22524										
16	-0.17175	-0.21339	-0.27100											
8	-0.22414	-0.29497												
4	-0.39348													

(c) $\mathcal{P}^{\mathcal{C}}$ for Scene: [**Veach Door**] Algorithm: [**PSSMLT**] Metric: [**IW-SSIM**] True GT: [**BDPT @ 524288 spp**].

MLT	2	4	8	16	32	64	128	256	512	1024	2048	4096	8192	16384
GT (BDPT)														
262144	-0.00045	-0.00033	-0.00042	-0.00064	-0.00037	-0.00032	-0.00019	-0.00033	-0.00013	-0.00014	-0.00012	-0.00009	-0.00012	-0.00009
131072	-0.00149	-0.00158	-0.00162	-0.00144	-0.00124	-0.00087	-0.00051	-0.00065	-0.00033	-0.00030	-0.00023	-0.00019	-0.00016	-0.00016
65536	-0.00393	-0.00404	-0.00387	-0.00335	-0.00283	-0.00244	-0.00135	-0.00126	-0.00085	-0.00059	-0.00052	-0.00051	-0.00047	-0.00044
32768	-0.00719	-0.00753	-0.00744	-0.00609	-0.00544	-0.00418	-0.00314	-0.00232	-0.00170	-0.00143	-0.00117	-0.00120	-0.00108	-0.00101
16384	-0.01264	-0.01295	-0.01207	-0.01128	-0.00914	-0.00717	-0.00559	-0.00425	-0.00369	-0.00290	-0.00264	-0.00242	-0.00222	
8192	-0.01893	-0.01886	-0.01930	-0.01696	-0.01509	-0.01199	-0.00975	-0.00787	-0.00702	-0.00591	-0.00528	-0.00492		
4096	-0.02783	-0.02715	-0.02586	-0.02383	-0.02072	-0.01766	-0.01500	-0.01355	-0.01188	-0.01113	-0.01079			
2048	-0.03758	-0.03621	-0.03618	-0.03481	-0.03029	-0.02769	-0.02509	-0.02442	-0.02272	-0.02134				
1024	-0.04793	-0.04914	-0.05005	-0.04441	-0.04558	-0.04605	-0.04195	-0.04196	-0.04244					
512	-0.06016	-0.05962	-0.05984	-0.05988	-0.06448	-0.06470	-0.07307	-0.07495						
256	-0.07579	-0.07496	-0.08346	-0.08705	-0.09786	-0.10839	-0.11791							
128	-0.08054	-0.09761	-0.10806	-0.11596	-0.13914	-0.16272								
64	-0.11510	-0.11267	-0.14214	-0.17130	-0.21642									
32	-0.13230	-0.16718	-0.20331	-0.25154										
16	-0.15753	-0.20556	-0.26660											
8	-0.23697	-0.30321												
4	-0.39797													

(d) $\mathcal{P}^{\mathcal{C}}$ for Scene: [**Veach Door**] Algorithm: [**MLT**] Metric: [**IW-SSIM**] True GT: [**BDPT @ 524288 spp**].

M-MLT	2	4	8	16	32	64	128	256	512	1024	2048	4096	8192	16384
GT (BDPT)														
262144	-0.00072	-0.00039	-0.00063	-0.00049	-0.00036	-0.00031	-0.00021	-0.00016	-0.00019	-0.00012	-0.00017	-0.00012	-0.00010	-0.00010
131072	-0.00202	-0.00148	-0.00136	-0.00156	-0.00126	-0.00092	-0.00073	-0.00036	-0.00039	-0.00023	-0.00020	-0.00023	-0.00020	-0.00018
65536	-0.00424	-0.00372	-0.00363	-0.00338	-0.00298	-0.00231	-0.00181	-0.00124	-0.00093	-0.00073	-0.00059	-0.00053	-0.00053	-0.00046
32768	-0.00801	-0.00754	-0.00735	-0.00616	-0.00538	-0.00442	-0.00363	-0.00251	-0.00187	-0.00150	-0.00127	-0.00114	-0.00112	-0.00099
16384	-0.01336	-0.01231	-0.01182	-0.01112	-0.00961	-0.00686	-0.00564	-0.00420	-0.00346	-0.00295	-0.00265	-0.00245	-0.00223	
8192	-0.01969	-0.01970	-0.01784	-0.01712	-0.01432	-0.01142	-0.00920	-0.00787	-0.00663	-0.00558	-0.00554	-0.00493		
4096	-0.02943	-0.02805	-0.02579	-0.02494	-0.02103	-0.01790	-0.01523	-0.01392	-0.01256	-0.01128	-0.01105			
2048	-0.03715	-0.03650	-0.03610	-0.03475	-0.03110	-0.02785	-0.02614	-0.02335	-0.02272	-0.02158				
1024	-0.04847	-0.04659	-0.04498	-0.04463	-0.04416	-0.04143	-0.04090	-0.04279	-0.04205					
512	-0.05904	-0.06124	-0.05926	-0.06028	-0.06740	-0.06785	-0.07166	-0.07356						
256	-0.07203	-0.07383	-0.08025	-0.08319	-0.09311	-0.10934	-0.11473							
128	-0.09186	-0.09661	-0.10043	-0.12471	-0.14274	-0.17034								
64	-0.10832	-0.12085	-0.13892	-0.17802	-0.21026									
32	-0.14126	-0.15907	-0.21536	-0.24939										
16	-0.17862	-0.20931	-0.28958											
8	-0.25120	-0.32064												
4	-0.38213													

(e) $\mathcal{P}^{\mathcal{C}}$ for Scene: [**Veach Door**] Algorithm: [**M-MLT**] Metric: [**IW-SSIM**] True GT: [**BDPT @ 524288 spp**].

ERPT	2	4	8	16	32	64	128	256	512	1024	2048	4096	8192	16384	32768	65536	131072
GT (BDPT)																	
262144	-0.00036	-0.00035	-0.00037	-0.00028	-0.00033	-0.00017	-0.00016	-0.00033	-0.00044	-0.00020	-0.00026	-0.00015	-0.00018	-0.00033	-0.00023	-0.00030	-0.00026
131072	-0.00077	-0.00065	-0.00081	-0.00060	-0.00079	-0.00072	-0.00063	-0.00059	-0.00080	-0.00072	-0.00065	-0.00057	-0.00061	-0.00072	-0.00056	-0.00073	
65536	-0.00176	-0.00144	-0.00143	-0.00176	-0.00174	-0.00155	-0.00154	-0.00171	-0.00153	-0.00150	-0.00148	-0.00155	-0.00168	-0.00163	-0.00137		
32768	-0.00332	-0.00300	-0.00304	-0.00314	-0.00325	-0.00285	-0.00288	-0.00302	-0.00319	-0.00300	-0.00294	-0.00271	-0.00315	-0.00307			
16384	-0.00498	-0.00547	-0.00529	-0.00563	-0.00534	-0.00563	-0.00540	-0.00557	-0.00524	-0.00591	-0.00536	-0.00561	-0.00539				
8192	-0.00843	-0.00818	-0.00910	-0.00910	-0.00879	-0.00904	-0.00900	-0.00832	-0.00822	-0.00988	-0.00888	-0.00906					
4096	-0.01477	-0.01512	-0.01522	-0.01525	-0.01425	-0.01489	-0.01515	-0.01520	-0.01526	-0.01486	-0.01515						
2048	-0.02473	-0.02372	-0.02535	-0.02603	-0.02488	-0.02565	-0.02457	-0.02388	-0.02363	-0.02439							
1024	-0.04208	-0.04209	-0.04252	-0.04338	-0.04438	-0.04406	-0.04216	-0.04240	-0.04292								
512	-0.07247	-0.07073	-0.07182	-0.07324	-0.07392	-0.07457	-0.07317	-0.07392									
256	-0.11571	-0.11930	-0.11857	-0.11724	-0.11930	-0.12094	-0.12007										
128	-0.18319	-0.19272	-0.18859	-0.19102	-0.19465	-0.18762											
64	-0.27118	-0.26975	-0.28192	-0.27910	-0.28011												
32	-0.37688	-0.38294	-0.38495	-0.39113													
16	-0.49097	-0.50020	-0.50520														
8	-0.65382	-0.66504															
4	-0.85528																

(f) $\mathcal{P}^{\mathcal{C}}$ for Scene: [**Veach Door**] Algorithm: [**ERPT**] Metric: [**IW-SSIM**] True GT: [**BDPT @ 524288 spp**].

M-ERPT	2	4	8	16	32	64	128	256	512	1024	2048	4096	8192	16384	32768	65536	131072
GT (BDPT)																	
262144	-0.00017	-0.00036	-0.00022	-0.00024	-0.00015	-0.00021	-0.00043	-0.00021	-0.00034	-0.00027	-0.00014	-0.00017	-0.00035	-0.00036	-0.00027	-0.00044	-0.00038
131072	-0.00078	-0.00077	-0.00078	-0.00057	-0.00035	-0.00070	-0.00072	-0.00050	-0.00079	-0.00087	-0.00046	-0.00078	-0.00079	-0.00082	-0.00068	-0.00090	
65536	-0.00166	-0.00174	-0.00186	-0.00152	-0.00148	-0.00151	-0.00182	-0.00184	-0.00142	-0.00173	-0.00162	-0.00185	-0.00203	-0.00158	-0.00170		
32768	-0.00311	-0.00327	-0.00299	-0.00308	-0.00330	-0.00335	-0.00353	-0.00293	-0.00302	-0.00316	-0.00300	-0.00326	-0.00338	-0.00302			
16384	-0.00497	-0.00612	-0.00594	-0.00598	-0.00546	-0.00598	-0.00583	-0.00591	-0.00582	-0.00557	-0.00571	-0.00596	-0.00578				
8192	-0.00919	-0.00858	-0.00928	-0.00969	-0.00958	-0.00920	-0.01004	-0.00913	-0.01005	-0.00978	-0.00976	-0.01033					
4096	-0.01479	-0.01549	-0.01656	-0.01608	-0.01539	-0.01594	-0.01636	-0.01551	-0.01602	-0.01589	-0.01606						
2048	-0.02496	-0.02535	-0.02615	-0.02360	-0.02577	-0.02503	-0.02583	-0.02674	-0.02551	-0.02641							
1024	-0.04246	-0.04187	-0.04431	-0.04483	-0.04470	-0.04372	-0.04344	-0.04404	-0.04460								
512	-0.06917	-0.07123	-0.07133	-0.07400	-0.07161	-0.07464	-0.07493	-0.07263									
256	-0.11747	-0.11549	-0.11863	-0.11934	-0.11465	-0.11846	-0.11945										
128	-0.18198	-0.18490	-0.18347	-0.18570	-0.18471	-0.18699											
64	-0.27074	-0.27168	-0.27593	-0.27406	-0.27749												
32	-0.38301	-0.38493	-0.37863	-0.38416													
16	-0.49638	-0.49937	-0.50251														
8	-0.65651	-0.66800															
4	-0.86341																

(g) $\mathcal{P}^{\mathcal{C}}$ for Scene: [**Veach Door**] Algorithm: [**M-ERPT**] Metric: [**IW-SSIM**] True GT: [**BDPT @ 524288 spp**].

Table 69

15.5 Sponza

PT	2	4	8	16	32	64	128	256	512	1024	2048	4096	8192
GT (PT)													
16384	-0.00194	-0.00189	-0.00108	-0.00156	-0.00121	-0.00125	-0.00088	-0.00158	-0.00087	-0.00086	-0.00080	-0.00082	-0.00073
8192	-0.00224	-0.00387	-0.00386	-0.00360	-0.00346	-0.00282	-0.00274	-0.00296	-0.00294	-0.00244	-0.00218	-0.00220	
4096	-0.00683	-0.00788	-0.00850	-0.00754	-0.00751	-0.00652	-0.00575	-0.00614	-0.00558	-0.00545	-0.00516		
2048	-0.01176	-0.01182	-0.00991	-0.01160	-0.01206	-0.01160	-0.01093	-0.01238	-0.01137	-0.01117			
1024	-0.01859	-0.02009	-0.01784	-0.01649	-0.01874	-0.01990	-0.02077	-0.02199	-0.02204				
512	-0.02843	-0.03054	-0.02695	-0.02951	-0.02786	-0.03130	-0.03755	-0.03791					
256	-0.03623	-0.03563	-0.03556	-0.04087	-0.04695	-0.05573	-0.06197						
128	-0.05129	-0.04837	-0.05141	-0.05930	-0.08086	-0.09688							
64	-0.05964	-0.06530	-0.07420	-0.10897	-0.12541								
32	-0.07937	-0.09112	-0.10492	-0.15185									
16	-0.12190	-0.14358	-0.16242										
8	-0.15159	-0.21511											
4	-0.23480												

(a) $\mathcal{P}^{\mathcal{C}}$ for Scene: [**Sponza**] Algorithm: [**PT**] Metric: [**IW-SSIM**] True GT: [**PT @ 32768 spp**].

BDPT	2	4	8	16	32	64	128	256	512	1024	2048	4096	8192
GT (PT)													
16384	-0.00128	-0.00082	-0.00125	-0.00232	-0.00104	-0.00121	-0.00090	-0.00107	-0.00079	-0.00101	-0.00080	-0.00078	-0.00070
8192	-0.00393	-0.00247	-0.00402	-0.00326	-0.00367	-0.00346	-0.00305	-0.00292	-0.00270	-0.00243	-0.00226	-0.00228	
4096	-0.00612	-0.00735	-0.00682	-0.00798	-0.00838	-0.00621	-0.00563	-0.00538	-0.00546	-0.00561	-0.00565		
2048	-0.01238	-0.01193	-0.01329	-0.01179	-0.01306	-0.01171	-0.01229	-0.01142	-0.01134	-0.01161			
1024	-0.01887	-0.01591	-0.02082	-0.01936	-0.01819	-0.01867	-0.01932	-0.02066	-0.02230				
512	-0.02511	-0.02821	-0.02600	-0.03053	-0.02853	-0.03038	-0.03314	-0.03783					
256	-0.03246	-0.03530	-0.03962	-0.03816	-0.04487	-0.04904	-0.06114						
128	-0.05192	-0.04975	-0.06109	-0.05539	-0.06611	-0.09202							
64	-0.04987	-0.06536	-0.07448	-0.09002	-0.11413								
32	-0.06651	-0.09598	-0.10377	-0.14255									
16	-0.11644	-0.10747	-0.15885										
8	-0.14584	-0.16532											
4	-0.18695												

(b) $\mathcal{P}^{\mathcal{C}}$ for Scene: [**Sponza**] Algorithm: [**BDPT**] Metric: [**IW-SSIM**] True GT: [**PT @ 32768 spp**].

PSSMLT	2	4	8	16	32	64	128	256	512	1024	2048	4096	8192
GT (PT)													
16384	-0.00143	-0.00217	-0.00171	-0.00148	-0.00130	-0.00076	-0.00084	-0.00087	-0.00078	-0.00059	-0.00058	-0.00065	-0.00045
8192	-0.00438	-0.00452	-0.00427	-0.00404	-0.00325	-0.00279	-0.00297	-0.00268	-0.00234	-0.00190	-0.00193	-0.00183	
4096	-0.00735	-0.00762	-0.00713	-0.00715	-0.00632	-0.00604	-0.00589	-0.00544	-0.00529	-0.00475	-0.00440		
2048	-0.01074	-0.01183	-0.01138	-0.01122	-0.01098	-0.00995	-0.01076	-0.01017	-0.01003	-0.00983			
1024	-0.01599	-0.01629	-0.01670	-0.01732	-0.01541	-0.01644	-0.01889	-0.01951	-0.02043				
512	-0.02519	-0.02802	-0.02768	-0.02672	-0.02778	-0.03051	-0.03339	-0.03695					
256	-0.03372	-0.03533	-0.03696	-0.04061	-0.04733	-0.05098	-0.05816						
128	-0.05873	-0.05322	-0.05604	-0.06266	-0.07541	-0.08763							
64	-0.06921	-0.07696	-0.09012	-0.10561	-0.12242								
32	-0.08257	-0.10065	-0.11522	-0.14416									
16	-0.10748	-0.14376	-0.19353										
8	-0.18278	-0.22060											
4	-0.25686												

(c) $\mathcal{P}^{\mathcal{C}}$ for Scene: [**Sponza**] Algorithm: [**PSSMLT**] Metric: [**IW-SSIM**] True GT: [**PT @ 32768 spp**].

MLT	2	4	8	16	32	64	128	256	512	1024	2048	4096	8192
GT (PT)													
16384	-0.00153	-0.00166	-0.00093	-0.00104	-0.00085	-0.00042	-0.00033	-0.00073	-0.00053	-0.00055	-0.00049	-0.00037	-0.00003
8192	-0.00281	-0.00258	-0.00321	-0.00221	-0.00228	-0.00193	-0.00195	-0.00170	-0.00171	-0.00148	-0.00165	-0.00135	
4096	-0.00683	-0.00798	-0.00856	-0.00554	-0.00593	-0.00453	-0.00468	-0.00479	-0.00417	-0.00420	-0.00375		
2048	-0.01271	-0.01181	-0.01052	-0.01022	-0.01042	-0.01065	-0.01072	-0.00987	-0.01013	-0.00953			
1024	-0.01834	-0.01892	-0.01612	-0.01684	-0.01809	-0.02016	-0.01997	-0.02001	-0.02033				
512	-0.02825	-0.02701	-0.02534	-0.02559	-0.03542	-0.03265	-0.03733	-0.03845					
256	-0.03954	-0.04279	-0.04215	-0.05437	-0.06045	-0.06571	-0.06724						
128	-0.04726	-0.05408	-0.06123	-0.07425	-0.09478	-0.11164							
64	-0.06321	-0.08362	-0.10276	-0.12818	-0.15636								
32	-0.08474	-0.11332	-0.15408	-0.20086									
16	-0.11235	-0.17324	-0.23124										
8	-0.16208	-0.26495											
4	-0.29313												

(d) $\mathcal{P}^{\mathcal{C}}$ for Scene: [**Sponza**] Algorithm: [**MLT**] Metric: [**IW-SSIM**] True GT: [**PT @ 32768 spp**].

M-MLT	2	4	8	16	32	64	128	256	512	1024	2048	4096	8192
GT (PT)													
16384	-0.00223	-0.00169	-0.00163	-0.00051	-0.00085	-0.00077	-0.00074	-0.00044	-0.00037	-0.00046	-0.00042	-0.00026	-0.00021
8192	-0.00299	-0.00309	-0.00298	-0.00273	-0.00258	-0.00241	-0.00231	-0.00193	-0.00179	-0.00146	-0.00127	-0.00095	
4096	-0.00716	-0.00657	-0.00558	-0.00504	-0.00585	-0.00558	-0.00482	-0.00498	-0.00441	-0.00420	-0.00387		
2048	-0.01056	-0.01141	-0.01104	-0.01080	-0.01121	-0.01159	-0.01048	-0.01014	-0.00935	-0.00900			
1024	-0.01961	-0.01776	-0.01815	-0.01672	-0.01704	-0.02057	-0.01873	-0.02033	-0.02024				
512	-0.02798	-0.02648	-0.02266	-0.02777	-0.03200	-0.03543	-0.03803	-0.03833					
256	-0.04188	-0.03770	-0.04113	-0.04554	-0.05353	-0.06481	-0.06995						
128	-0.05427	-0.05368	-0.06416	-0.07758	-0.09265	-0.11223							
64	-0.08155	-0.08062	-0.10311	-0.13285	-0.16824								
32	-0.09133	-0.11728	-0.14825	-0.20245									
16	-0.14053	-0.19229	-0.25361										
8	-0.21990	-0.28614											
4	-0.32398												

(e) $\mathcal{P}^{\mathcal{C}}$ for Scene: [**Sponza**] Algorithm: [**M-MLT**] Metric: [**IW-SSIM**] True GT: [**PT @ 32768 spp**].

ERPT	2	4	8	16	32	64	128	256	512	1024	2048	4096	8192
GT (PT)													
16384	-0.00084	-0.00076	-0.00066	-0.00062	-0.00086	-0.00086	-0.00058	-0.00073	-0.00076	-0.00055	-0.00068	-0.00072	-0.00077
8192	-0.00219	-0.00195	-0.00197	-0.00204	-0.00274	-0.00242	-0.00187	-0.00182	-0.00214	-0.00183	-0.00239	-0.00275	
4096	-0.00472	-0.00452	-0.00467	-0.00516	-0.00491	-0.00506	-0.00457	-0.00488	-0.00516	-0.00505	-0.00480		
2048	-0.00968	-0.01018	-0.01070	-0.00990	-0.01009	-0.01083	-0.00978	-0.01033	-0.01133	-0.00989			
1024	-0.02001	-0.02042	-0.02039	-0.02077	-0.02032	-0.02043	-0.02028	-0.01929	-0.01992				
512	-0.03733	-0.03696	-0.03875	-0.03743	-0.03832	-0.03897	-0.03915	-0.03810					
256	-0.06877	-0.06982	-0.07028	-0.06878	-0.07215	-0.07066	-0.07004						
128	-0.12777	-0.12826	-0.12726	-0.12771	-0.13060	-0.12778							
64	-0.21127	-0.21143	-0.21321	-0.21200	-0.21016								
32	-0.32361	-0.31709	-0.31638	-0.31767									
16	-0.46731	-0.46510	-0.46409										
8	-0.62840	-0.63038											
4	-0.81883												

(f) $\mathcal{P}^{\mathcal{C}}$ for Scene: [**Sponza**] Algorithm: [**ERPT**] Metric: [**IW-SSIM**] True GT: [**PT @ 32768 spp**].

M-ERPT	2	4	8	16	32	64	128	256	512	1024	2048	4096	8192
GT (PT)													
16384	-0.00077	-0.00086	-0.00071	-0.00121	-0.00067	-0.00053	-0.00049	-0.00047	-0.00030	-0.00079	-0.00084	-0.00054	-0.00042
8192	-0.00251	-0.00213	-0.00228	-0.00214	-0.00242	-0.00252	-0.00219	-0.00221	-0.00166	-0.00218	-0.00208	-0.00254	
4096	-0.00539	-0.00519	-0.00545	-0.00481	-0.00518	-0.00501	-0.00478	-0.00445	-0.00417	-0.00482	-0.00501		
2048	-0.01041	-0.01034	-0.01020	-0.00992	-0.01028	-0.01024	-0.00955	-0.01022	-0.01063	-0.01039			
1024	-0.02071	-0.01871	-0.02042	-0.01952	-0.01957	-0.02059	-0.02018	-0.02213	-0.02037				
512	-0.03783	-0.03781	-0.03789	-0.03906	-0.03916	-0.03804	-0.03858	-0.03718					
256	-0.06707	-0.07020	-0.07033	-0.06932	-0.07177	-0.07024	-0.06904						
128	-0.12617	-0.12855	-0.12496	-0.12228	-0.12673	-0.12726							
64	-0.20788	-0.20662	-0.20736	-0.20701	-0.20533								
32	-0.31539	-0.32005	-0.31588	-0.31654									
16	-0.45960	-0.46078	-0.46295										
8	-0.63069	-0.62941											
4	-0.81684												

(g) $\mathcal{P}^{\mathcal{C}}$ for Scene: [**Sponza**] Algorithm: [**M-ERPT**] Metric: [**IW-SSIM**] True GT: [**PT @ 32768 spp**].

Table 70

16 NQM

16.1 Cornell Box

PT	2	4	8	16	32	64	128	256	512	1024	2048	4096	8192
GT (PT)													
16384	0.00023	-0.00028	-0.00035	-0.00002	-0.00035	0.00007	-0.00000	-0.00194	-0.00233	-0.00462	-0.00358	-0.00819	-0.01073
8192	-0.00010	-0.00034	-0.00074	-0.00041	-0.00036	0.00037	-0.00386	-0.00449	-0.00806	-0.00794	-0.01330	-0.01745	
4096	0.00046	-0.00029	-0.00029	-0.00041	-0.00047	-0.00072	-0.00297	-0.00490	-0.00780	-0.01451	-0.02421		
2048	-0.00072	-0.00060	-0.00075	-0.00151	-0.00155	-0.00396	-0.00451	-0.01279	-0.01644	-0.02861			
1024	0.00039	-0.00029	-0.00100	-0.00266	-0.00429	-0.00648	-0.01241	-0.02346	-0.03804				
512	-0.00058	-0.00104	-0.00160	-0.00528	-0.00850	-0.01258	-0.02152	-0.03482					
256	-0.00087	-0.00282	-0.00402	-0.00929	-0.01449	-0.02615	-0.04742						
128	-0.00283	-0.00507	-0.01020	-0.02093	-0.03360	-0.05607							
64	-0.00587	-0.01184	-0.02595	-0.04099	-0.06497								
32	-0.01073	-0.02333	-0.04891	-0.08213									
16	-0.02235	-0.05211	-0.08382										
8	-0.04723	-0.10430											
4	-0.09422												

(a) $\mathcal{P}^{\mathcal{C}}$ for Scene: [**Cornell Box**] Algorithm: [**PT**] Metric: [**NQM**] True GT: [**PT @ 32768 spp**].

BDPT	2	4	8	16	32	64	128	256	512	1024	2048	4096	8192
GT (PT)													
16384	-0.00018	-0.00008	-0.00069	-0.00063	-0.00080	-0.00143	-0.00319	-0.00179	-0.00544	0.00006	-0.00351	-0.00404	-0.00943
8192	-0.00048	-0.00023	-0.00091	-0.00058	-0.00201	-0.00312	-0.00246	-0.00616	-0.01302	-0.00892	-0.01458	-0.01870	
4096	-0.00024	-0.00049	-0.00106	-0.00144	-0.00289	-0.00591	-0.00788	-0.01246	-0.02085	-0.02721	-0.03961		
2048	-0.00093	-0.00098	-0.00181	-0.00317	-0.00595	-0.00973	-0.01780	-0.02396	-0.04153	-0.05132			
1024	-0.00206	-0.00252	-0.00337	-0.00599	-0.01241	-0.01943	-0.03079	-0.04494	-0.06861				
512	-0.00221	-0.00380	-0.00776	-0.01228	-0.01905	-0.02788	-0.05554	-0.07307					
256	-0.00541	-0.00856	-0.01696	-0.02583	-0.03740	-0.05897	-0.09399						
128	-0.00870	-0.01777	-0.02808	-0.04508	-0.07589	-0.10823							
64	-0.01789	-0.03537	-0.05999	-0.09302	-0.13370								
32	-0.03834	-0.06279	-0.10373	-0.15916									
16	-0.06805	-0.11293	-0.17284										
8	-0.12765	-0.19729											
4	-0.22250												

(b) $\mathcal{P}^{\mathcal{C}}$ for Scene: [**Cornell Box**] Algorithm: [**BDPT**] Metric: [**NQM**] True GT: [**PT @ 32768 spp**].

PSSMLT	2	4	8	16	32	64	128	256	512	1024	2048	4096	8192
GT (PT)													
16384	0.00020	0.00006	-0.00010	0.00031	-0.00069	-0.00082	-0.00080	0.00020	-0.00170	-0.00100	0.00044	-0.00039	0.00175
8192	-0.00034	-0.00033	0.00013	0.00007	-0.00096	-0.00121	-0.00120	-0.00329	-0.00699	-0.00647	-0.00714	-0.00935	
4096	0.00027	-0.00001	-0.00003	-0.00020	-0.00091	-0.00258	-0.00381	-0.00656	-0.01222	-0.01198	-0.01133		
2048	-0.00073	-0.00095	-0.00183	-0.00188	-0.00454	-0.00527	-0.00737	-0.01215	-0.01766	-0.01992			
1024	-0.00001	-0.00120	-0.00155	-0.00217	-0.00636	-0.01135	-0.01858	-0.02351	-0.03826				
512	-0.00056	-0.00203	-0.00434	-0.00739	-0.01530	-0.02291	-0.03157	-0.04476					
256	-0.00321	-0.00698	-0.01221	-0.02146	-0.03589	-0.05314	-0.07205						
128	-0.00579	-0.01333	-0.02276	-0.03615	-0.06375	-0.08606							
64	-0.01549	-0.02968	-0.04541	-0.07601	-0.11755								
32	-0.02872	-0.05419	-0.08797	-0.13557									
16	-0.05548	-0.09944	-0.15386										
8	-0.10265	-0.17295											
4	-0.19865												

(c) $\mathcal{P}^{\mathcal{C}}$ for Scene: [**Cornell Box**] Algorithm: [**PSSMLT**] Metric: [**NQM**] True GT: [**PT @ 32768 spp**].

MLT	2	4	8	16	32	64	128	256	512	1024	2048	4096	8192
GT (PT)													
16384	0.00017	0.00029	-0.00002	0.00005	0.00013	-0.00036	-0.00055	-0.00073	0.00040	-0.00036	0.00062	-0.00229	-0.00075
8192	0.00005	-0.00011	0.00029	-0.00004	-0.00040	0.00009	0.00038	-0.00009	-0.00081	-0.00193	-0.00026	-0.00192	
4096	0.00028	0.00010	-0.00010	-0.00021	-0.00044	-0.00066	-0.00111	-0.00311	-0.00353	-0.00473	-0.00550		
2048	-0.00050	-0.00052	-0.00035	-0.00072	-0.00132	-0.00184	-0.00321	-0.00558	-0.00938	-0.01373			
1024	0.00021	-0.00098	-0.00162	-0.00264	-0.00447	-0.00560	-0.00908	-0.01498	-0.01762				
512	0.00084	-0.00043	-0.00189	-0.00307	-0.00491	-0.00839	-0.01307	-0.02228					
256	0.00099	-0.00103	-0.00282	-0.00641	-0.00898	-0.01620	-0.02423						
128	0.00204	-0.00195	-0.00512	-0.00935	-0.01783	-0.03288							
64	0.00162	-0.00469	-0.01188	-0.02383	-0.03847								
32	0.00053	-0.00988	-0.02655	-0.04792									
16	0.00192	-0.01895	-0.05135										
8	0.01023	-0.03555											
4	-0.00647												

(d) $\mathcal{P}^{\mathcal{C}}$ for Scene: [**Cornell Box**] Algorithm: [**MLT**] Metric: [**NQM**] True GT: [**PT @ 32768 spp**].

M-MLT	2	4	8	16	32	64	128	256	512	1024	2048	4096	8192
GT (PT)													
16384	-0.00008	-0.00004	-0.00001	-0.00006	0.00008	-0.00074	-0.00095	-0.00143	-0.00113	-0.00229	-0.00140	-0.00276	-0.00159
8192	-0.00033	-0.00039	-0.00043	-0.00050	-0.00044	-0.00042	-0.00084	-0.00099	-0.00254	-0.00227	-0.00427	-0.00262	
4096	0.00029	0.00025	-0.00004	-0.00015	-0.00018	-0.00168	-0.00279	-0.00372	-0.00637	-0.00664	-0.00814		
2048	-0.00055	-0.00038	-0.00040	-0.00061	-0.00093	-0.00205	-0.00332	-0.00562	-0.01028	-0.01669			
1024	-0.00045	0.00040	-0.00049	-0.00097	-0.00159	-0.00238	-0.00663	-0.01238	-0.01684				
512	0.00072	-0.00073	-0.00170	-0.00293	-0.00491	-0.00862	-0.01357	-0.02005					
256	-0.00062	-0.00214	-0.00342	-0.00628	-0.00972	-0.01575	-0.02622						
128	0.00451	-0.00110	-0.00470	-0.00872	-0.01694	-0.03304							
64	0.00056	-0.01011	-0.01564	-0.02281	-0.03928								
32	-0.00122	-0.01836	-0.03097	-0.04763									
16	-0.00249	-0.02782	-0.05386										
8	-0.00566	-0.04459											
4	-0.01420												

(e) $\mathcal{P}^{\mathcal{C}}$ for Scene: [**Cornell Box**] Algorithm: [**M-MLT**] Metric: [**NQM**] True GT: [**PT @ 32768 spp**].

ERPT	2	4	8	16	32	64	128	256	512	1024	2048	4096	8192
GT (PT)													
16384	0.00009	-0.00119	-0.00065	-0.00005	0.00022	-0.00023	-0.00069	0.00018	-0.00073	0.00041	-0.00047	-0.00058	-0.00002
8192	-0.00100	-0.00151	-0.00036	0.00016	-0.00074	-0.00039	-0.00051	-0.00034	-0.00122	-0.00010	-0.00107	0.00013	
4096	-0.00057	-0.00205	-0.00217	0.00039	-0.00138	-0.00164	-0.00193	-0.00136	-0.00154	-0.00148	-0.00161		
2048	-0.00245	-0.00498	-0.00506	-0.00268	-0.00278	-0.00517	-0.00425	-0.00424	-0.00537	-0.00216			
1024	-0.00591	-0.00618	-0.00866	-0.00357	-0.00723	-0.00889	-0.00690	-0.00668	-0.00925				
512	-0.01108	-0.01391	-0.01337	-0.01160	-0.01305	-0.01635	-0.01360	-0.01465					
256	-0.02459	-0.02319	-0.02584	-0.02428	-0.02843	-0.02872	-0.02997						
128	-0.04983	-0.05199	-0.05262	-0.05118	-0.05205	-0.05986							
64	-0.09509	-0.09570	-0.09990	-0.09319	-0.09791								
32	-0.16780	-0.16765	-0.17214	-0.16505									
16	-0.26221	-0.26619	-0.26604										
8	-0.39835	-0.39719											
4	-0.55481												

(f) $\mathcal{P}^{\mathcal{C}}$ for Scene: [**Cornell Box**] Algorithm: [**ERPT**] Metric: [**NQM**] True GT: [**PT @ 32768 spp**].

M-ERPT	2	4	8	16	32	64	128	256	512	1024	2048	4096	8192
GT (PT)													
16384	-0.00036	-0.00013	-0.00015	0.00013	-0.00061	0.00022	-0.00086	-0.00050	-0.00040	0.00094	-0.00044	-0.00006	-0.00088
8192	-0.00180	-0.00144	-0.00060	-0.00030	-0.00061	-0.00060	-0.00087	-0.00027	-0.00014	0.00034	-0.00042	-0.00048	
4096	-0.00176	-0.00112	-0.00058	-0.00039	-0.00200	-0.00153	-0.00162	-0.00179	-0.00061	-0.00018	-0.00078		
2048	-0.00217	-0.00323	-0.00312	-0.00274	-0.00456	-0.00186	-0.00443	-0.00287	-0.00070	-0.00162			
1024	-0.00637	-0.00600	-0.00611	-0.00709	-0.00760	-0.00470	-0.00701	-0.00664	-0.00222				
512	-0.01064	-0.01064	-0.01196	-0.01185	-0.01251	-0.01286	-0.01215	-0.01189					
256	-0.02144	-0.02902	-0.02499	-0.02651	-0.02640	-0.02761	-0.02721						
128	-0.04414	-0.05245	-0.04729	-0.05162	-0.05399	-0.04648							
64	-0.09243	-0.09720	-0.09464	-0.09937	-0.09339								
32	-0.15819	-0.16918	-0.15961	-0.16611									
16	-0.25124	-0.26703	-0.25155										
8	-0.38564	-0.39626											
4	-0.54419												

(g) $\mathcal{P}^{\mathcal{C}}$ for Scene: [**Cornell Box**] Algorithm: [**M-ERPT**] Metric: [**NQM**] True GT: [**PT @ 32768 spp**].

Table 71

16.2 Torus

PT	2	4	8	16	32	64	128	256	512	1024	2048	4096	8192	16384	32768	65536	131072
GT (BDPT)																	
262144	0.00535	0.00408	0.00382	0.00444	0.00398	0.00258	0.00340	0.00141	0.00269	0.00041	0.00071	-0.00344	-0.00045	-0.00823	-0.00650	-0.01345	-0.02031
131072	0.01299	0.00886	0.00861	0.00792	0.00977	0.00621	0.00456	0.00219	0.00252	0.00168	0.00228	-0.00389	-0.00704	-0.01555	-0.02389	-0.02673	
65536	0.01694	0.01239	0.01419	0.01047	0.01184	0.01168	0.00844	0.00389	0.00066	0.00082	-0.00418	-0.01146	-0.01492	-0.02764	-0.05258		
32768	0.01792	0.01633	0.01342	0.01217	0.01313	0.01406	0.00376	-0.00013	-0.00528	-0.00518	-0.01093	-0.02333	-0.03939	-0.06889			
16384	0.02848	0.01914	0.01833	0.01705	0.01420	0.01138	0.00784	-0.00595	-0.00632	-0.02266	-0.02378	-0.05009	-0.07729				
8192	0.05299	0.04763	0.04398	0.03574	0.02576	0.02077	0.01567	-0.00566	-0.00783	-0.03071	-0.05234	-0.10414					
4096	0.06812	0.05251	0.04654	0.04378	0.05530	0.02223	-0.00224	-0.01945	-0.02954	-0.07331	-0.10397						
2048	0.11237	0.08737	0.08065	0.06186	0.05227	0.03632	-0.00456	-0.04569	-0.06967	-0.11777							
1024	0.20573	0.13507	0.13035	0.11428	0.08244	0.04637	-0.01163	-0.10195	-0.13682								
512	0.25415	0.18434	0.17987	0.13345	0.09824	0.04091	-0.04377	-0.12892									
256	0.35144	0.25643	0.22141	0.19660	0.15765	0.06454	-0.02626										
128	0.44414	0.34633	0.33338	0.28211	0.19526	0.12884											
64	0.54945	0.42607	0.40468	0.33065	0.22705												
32	0.59309	0.47108	0.45935	0.34635													
16	0.65357	0.52930	0.53049														
8	0.68981	0.54021															
4	0.70500																

(a) $\mathcal{P}^{\mathcal{C}}$ for Scene: [**Torus**] Algorithm: [**PT**] Metric: [**NQM**] True GT: [**BDPT @ 524288 spp**].

BDPT	2	4	8	16	32	64	128	256	512	1024	2048	4096	8192	16384	32768	65536	131072
GT (BDPT)																	
262144	0.00702	0.00522	0.00473	0.00484	0.00277	0.00447	0.00322	0.00192	0.00140	0.00167	-0.00068	0.00117	-0.00413	-0.00315	-0.00144	-0.01583	-0.02305
131072	0.01481	0.01181	0.01069	0.00782	0.01069	0.00941	0.00761	0.00608	0.00531	0.00133	-0.00144	-0.00248	-0.00440	-0.01186	-0.01743	-0.03767	
65536	0.01909	0.01622	0.01549	0.01118	0.01102	0.01116	0.00892	0.00551	0.00240	0.00233	-0.00546	-0.00600	-0.01489	-0.03165	-0.03961		
32768	0.02389	0.01532	0.01553	0.01273	0.01401	0.00944	0.00805	0.00161	0.00253	-0.00701	-0.01553	-0.02939	-0.03644	-0.05962			
16384	0.03256	0.01965	0.01710	0.01312	0.01163	0.00959	0.00584	-0.00132	-0.00345	-0.01568	-0.03549	-0.05195	-0.06943				
8192	0.07166	0.05456	0.04329	0.04166	0.04245	0.01807	0.00779	0.01286	-0.01453	-0.03368	-0.05367	-0.09475					
4096	0.08939	0.06594	0.05508	0.02721	0.02720	0.01050	0.01433	-0.02381	-0.02971	-0.06744	-0.11237						
2048	0.13769	0.09547	0.08496	0.07088	0.04601	0.02126	0.01799	-0.02102	-0.07493	-0.12061							
1024	0.21206	0.17255	0.12711	0.11574	0.06290	0.04204	0.00048	-0.06333	-0.14106								
512	0.27187	0.20933	0.15780	0.13189	0.08611	0.05099	-0.01979	-0.08903									
256	0.38319	0.30035	0.23330	0.20576	0.14390	0.05846	-0.00517										
128	0.50073	0.40604	0.31355	0.28463	0.21420	0.10970											
64	0.55751	0.49246	0.38902	0.33955	0.25704												
32	0.64513	0.56551	0.43112	0.40223													
16	0.70167	0.62625	0.46123														
8	0.72807	0.64168															
4	0.74064																

(b) $\mathcal{P}^{\mathcal{C}}$ for Scene: [**Torus**] Algorithm: [**BDPT**] Metric: [**NQM**] True GT: [**BDPT @ 524288 spp**].

PSSMLT	2	4	8	16	32	64	128	256	512	1024	2048	4096	8192	16384
GT (BDPT)														
262144	0.00700	0.00357	0.00195	0.00338	0.00225	0.00313	0.00133	0.00121	-0.00008	-0.00052	-0.00001	0.00017	-0.00043	-0.00083
131072	0.01291	0.01022	0.01103	0.00989	0.00638	0.00581	0.00527	0.00179	0.00065	0.00080	0.00140	-0.00014	-0.00528	-0.00132
65536	0.02911	0.01438	0.00852	0.00926	0.00636	0.00277	0.00511	0.00272	-0.00006	-0.00455	-0.00672	-0.01083	-0.02176	-0.01431
32768	0.03975	0.01775	0.01026	0.00904	0.00647	0.00317	0.00425	-0.00492	-0.01353	-0.01753	-0.01799	-0.02742	-0.04632	-0.03458
16384	0.03426	0.00983	0.00680	-0.00012	-0.00540	-0.00919	-0.01295	-0.01951	-0.02464	-0.03464	-0.04069	-0.05663	-0.08954	
8192	0.09609	0.05197	0.03130	0.02020	0.00193	-0.00713	-0.02172	-0.03768	-0.06294	-0.08022	-0.09702	-0.11885		
4096	0.14923	0.07001	0.04388	0.04051	-0.00786	-0.02464	-0.04790	-0.07583	-0.10467	-0.13417	-0.16281			
2048	0.17671	0.07877	0.02090	-0.00915	-0.05480	-0.07182	-0.10806	-0.16384	-0.19065	-0.24296				
1024	0.28978	0.11305	0.03729	0.01219	-0.06588	-0.10422	-0.17662	-0.23992	-0.31164					
512	0.36745	0.16544	0.05688	0.01469	-0.11794	-0.16258	-0.25618	-0.34799						
256	0.45981	0.20173	0.06185	-0.00777	-0.16571	-0.24975	-0.35983							
128	0.54747	0.24309	0.09500	0.02469	-0.19029	-0.30248								
64	0.57188	0.26892	0.11146	0.03566	-0.23819									
32	0.63822	0.27241	0.08679	0.00084										
16	0.65518	0.25991	0.06944											
8	0.68905	0.28572												
4	0.68995													

(c) $\mathcal{P}^{\mathcal{C}}$ for Scene: [**Torus**] Algorithm: [**PSSMLT**] Metric: [**NQM**] True GT: [**BDPT @ 524288 spp**].

MLT	2	4	8	16	32	64	128	256	512	1024	2048	4096	8192	16384
GT (BDPT)														
262144	-0.00178	-0.00239	0.02558	0.00919	0.00574	0.00543	0.00167	0.00216	0.00120	0.00146	0.00060	0.00131	0.00184	0.00329
131072	-0.00403	-0.00434	0.03315	-0.00335	0.00290	0.00051	0.00149	0.00199	0.00172	0.00195	-0.00000	0.00069	0.00091	-0.00032
65536	-0.00748	-0.00755	0.07999	-0.00017	0.00836	0.00317	0.00278	0.00217	0.00091	0.00027	-0.00108	-0.00132	-0.00121	-0.00293
32768	-0.00989	-0.01295	0.09016	-0.01833	0.01123	-0.00260	-0.00361	-0.00548	-0.00806	-0.00895	-0.00706	-0.00588	-0.00798	-0.01346
16384	-0.01580	-0.02173	0.15206	0.02603	0.00728	0.00353	0.00017	0.00007	-0.00077	-0.00577	-0.00631	-0.00656	-0.00953	
8192	-0.02660	-0.03046	0.09696	-0.02077	-0.00004	-0.01912	-0.03262	-0.03002	-0.03740	-0.04136	-0.03923	-0.04004		
4096	-0.04940	-0.05783	0.27174	-0.02488	-0.01392	-0.02828	-0.04340	-0.05169	-0.06037	-0.07110	-0.06945			
2048	-0.07674	-0.07762	0.28233	-0.05651	-0.04465	-0.08567	-0.08936	-0.09086	-0.10840	-0.10602				
1024	-0.10566	-0.08972	0.47525	-0.13504	-0.10748	-0.15695	-0.17663	-0.18308	-0.20133					
512	-0.14378	-0.12725	0.43507	-0.18601	-0.16076	-0.21466	-0.23041	-0.24039						
256	-0.17634	-0.13796	0.49602	-0.27835	-0.21416	-0.32051	-0.34187							
128	-0.20511	-0.15631	0.58976	-0.40263	-0.26811	-0.36911								
64	-0.20216	-0.11565	0.52206	-0.53104	-0.38080									
32	-0.20988	-0.12940	0.62785	-0.64428										
16	-0.21702	-0.10263	0.51593											
8	-0.20808	-0.11367												
4	-0.25986													

(d) $\mathcal{P}^{\mathcal{C}}$ for Scene: [**Torus**] Algorithm: [**MLT**] Metric: [**NQM**] True GT: [**BDPT @ 524288 spp**].

M-MLT	2	4	8	16	32	64	128	256	512	1024	2048	4096	8192	16384
GT (BDPT)														
262144	-0.00116	-0.00420	0.01231	0.06727	0.01001	0.00569	0.00129	-0.00086	-0.00012	0.00087	0.00014	-0.00057	-0.00005	0.00346
131072	-0.00273	-0.00738	0.00938	0.08340	0.00816	-0.00134	-0.00187	0.00268	0.00010	-0.00135	0.00010	-0.00044	-0.00126	-0.00111
65536	-0.00532	-0.01565	0.02335	0.09993	0.01185	0.00193	-0.00247	-0.00366	-0.00069	-0.00135	-0.00310	-0.00292	-0.00461	-0.00523
32768	-0.00853	-0.02759	0.01684	0.11864	0.00824	0.00135	-0.00699	-0.00937	-0.00760	-0.01026	-0.00886	-0.00887	-0.01165	-0.02029
16384	-0.01301	-0.03471	0.03291	0.26022	0.01278	0.00188	-0.00919	-0.01211	-0.01319	-0.01220	-0.01761	-0.01732	-0.02156	
8192	-0.02155	-0.05625	0.04375	0.26144	-0.00123	-0.02566	-0.03230	-0.03701	-0.05542	-0.05028	-0.05654	-0.05721		
4096	-0.03614	-0.10056	0.00985	0.52472	-0.00336	-0.03175	-0.05238	-0.06835	-0.08158	-0.08501	-0.09367			
2048	-0.05707	-0.14966	0.00856	0.40989	-0.08402	-0.06632	-0.10244	-0.11761	-0.14061	-0.14838				
1024	-0.07791	-0.19947	-0.07947	0.49388	-0.11214	-0.15377	-0.20195	-0.21645	-0.23668					
512	-0.10759	-0.30206	-0.27077	0.58183	-0.17479	-0.18315	-0.24769	-0.28873						
256	-0.12486	-0.33643	-0.38910	0.50479	-0.24312	-0.29682	-0.37625							
128	-0.14412	-0.41810	-0.66290	0.57386	-0.32922	-0.33943								
64	-0.13944	-0.40654	-1.67389	-0.11521	-0.52986									
32	-0.13880	-0.41653	-2.24398	-0.32003										
16	-0.12757	-0.35074	-2.45935											
8	-0.13120	-0.36328												
4	-0.16212													

(e) $\mathcal{P}^{\mathcal{C}}$ for Scene: [**Torus**] Algorithm: [**M-MLT**] Metric: [**NQM**] True GT: [**BDPT @ 524288 spp**].

ERPT	2	4	8	16	32	64	128	256	512	1024	2048	4096	8192	16384	32768	65536	131072
GT (BDPT)																	
262144	0.00255	0.00250	0.00278	0.00186	-0.00126	-0.00046	-0.00304	-0.00122	-0.00233	-0.00196	-0.00055	-0.00226	-0.00169	-0.00099	-0.00176	-0.00163	-0.00062
131072	0.00252	-0.00001	0.00316	-0.00232	0.00170	-0.00393	-0.00438	-0.00110	-0.00430	-0.00776	-0.00795	-0.00781	-0.00968	-0.00960	-0.01148	-0.00752	
65536	0.00014	0.00025	-0.00118	-0.00429	-0.00386	-0.00217	-0.01131	-0.01022	-0.00851	-0.01458	-0.02025	-0.02758	-0.02263	-0.02505	-0.02292		
32768	-0.00377	-0.00283	-0.00133	-0.00686	-0.01713	-0.01459	-0.02439	-0.02253	-0.02342	-0.03146	-0.03973	-0.04850	-0.04714	-0.04407			
16384	0.00249	-0.00858	-0.01067	-0.00932	-0.01307	-0.02824	-0.04144	-0.04463	-0.04622	-0.06625	-0.08294	-0.09291	-0.08104				
8192	-0.00492	-0.02690	-0.03608	-0.02525	-0.04018	-0.06557	-0.09818	-0.09628	-0.10671	-0.13547	-0.15322	-0.16284					
4096	-0.02418	-0.03982	-0.06415	-0.06192	-0.08467	-0.12924	-0.16111	-0.17701	-0.18058	-0.21839	-0.24393						
2048	-0.04665	-0.10357	-0.14750	-0.13279	-0.16098	-0.21338	-0.26897	-0.29464	-0.28946	-0.34855							
1024	-0.10155	-0.20205	-0.19089	-0.21895	-0.27921	-0.32594	-0.41980	-0.43453	-0.44520								
512	-0.12437	-0.27435	-0.29853	-0.32019	-0.37542	-0.45528	-0.52500	-0.55741									
256	-0.20185	-0.39239	-0.41618	-0.39304	-0.49469	-0.57991	-0.68047										
128	-0.21126	-0.43628	-0.47284	-0.47530	-0.58941	-0.68488											
64	-0.27899	-0.52563	-0.58615	-0.56201	-0.65717												
32	-0.32776	-0.63549	-0.64698	-0.59995													
16	-0.39882	-0.73389	-0.74357														
8	-0.36987	-0.71862															
4	-0.43401																

(f) $\mathcal{P}^{\mathcal{C}}$ for Scene: [**Torus**] Algorithm: [**ERPT**] Metric: [**NQM**] True GT: [**BDPT @ 524288 spp**].

M-ERPT	2	4	8	16	32	64	128	256	512	1024	2048	4096	8192	16384	32768	65536	131072
GT (BDPT)																	
262144	0.00211	-0.00003	0.00078	0.00048	0.00216	0.00083	-0.00440	-0.00066	0.00011	-0.00038	-0.00141	-0.00130	-0.00208	-0.00223	-0.00057	0.00095	-0.00019
131072	0.00303	0.00001	0.00169	0.00464	0.00372	0.00151	-0.00603	-0.00378	-0.00416	-0.00301	-0.00561	-0.00662	-0.00672	-0.00475	-0.00777	-0.00746	
65536	0.00691	0.00168	0.00038	0.00068	-0.00605	-0.00081	-0.01283	-0.01075	-0.01554	-0.01434	-0.01722	-0.01592	-0.01720	-0.01843	-0.01792		
32768	0.00251	-0.01284	-0.00218	-0.00077	-0.00816	-0.01339	-0.03138	-0.02566	-0.03351	-0.03550	-0.04444	-0.03881	-0.04120	-0.04102			
16384	0.01288	-0.00111	-0.01073	-0.00920	-0.01178	-0.01565	-0.04942	-0.04261	-0.06160	-0.06262	-0.08567	-0.07228	-0.08116				
8192	0.01107	0.00888	-0.02428	-0.02889	-0.05097	-0.04098	-0.10493	-0.09908	-0.12994	-0.13262	-0.16252	-0.14270					
4096	0.00518	-0.01683	-0.04725	-0.07472	-0.08819	-0.09474	-0.17902	-0.17783	-0.20266	-0.21356	-0.25589						
2048	-0.03290	-0.00073	-0.09153	-0.12611	-0.16924	-0.18280	-0.28953	-0.29062	-0.32710	-0.34423							
1024	-0.12147	-0.06951	-0.17147	-0.21434	-0.29696	-0.29408	-0.44707	-0.42801	-0.48394								
512	-0.12488	-0.10711	-0.19726	-0.30162	-0.37960	-0.40352	-0.56733	-0.56200									
256	-0.27189	-0.14158	-0.29394	-0.40054	-0.49045	-0.51064	-0.70531										
128	-0.35517	-0.14804	-0.33853	-0.49706	-0.58822	-0.59717											
64	-0.48801	-0.17820	-0.36113	-0.57549	-0.66220												
32	-0.58006	-0.26230	-0.42972	-0.61168													
16	-0.65171	-0.32674	-0.48489														
8	-0.59197	-0.31810															
4	-0.73549																

(g) $\mathcal{P}^{\mathcal{C}}$ for Scene: [**Torus**] Algorithm: [**M-ERPT**] Metric: [**NQM**] True GT: [**BDPT @ 524288 spp**].

Table 72

16.3 Veach Bidir

PT	2	4	8	16	32	64	128	256	512	1024	2048	4096	8192	16384	32768	65536	131072
GT (BDPT)																	
262144	0.05999	0.00131	0.00085	0.00058	0.00038	0.00016	0.00045	0.00028	0.00011	0.00025	0.00010	0.00037	0.00010	0.00014	0.00080	0.00054	-0.00028
131072	0.15381	0.00328	0.00168	0.00117	0.00095	0.00069	0.00076	0.00057	0.00037	0.00050	0.00056	0.00041	0.00003	-0.00004	-0.00007	0.00033	
65536	0.13940	0.00311	0.00180	0.00146	0.00099	0.00090	0.00106	0.00041	0.00000	0.00049	0.00050	0.00063	0.00024	0.00004	0.00023		
32768	0.14219	0.00336	0.00194	0.00166	0.00094	0.00081	0.00098	0.00080	0.00018	0.00014	0.00036	0.00041	-0.00014	-0.00078			
16384	0.16618	0.00466	0.00304	0.00236	0.00173	0.00191	0.00161	0.00044	0.00100	0.00125	0.00108	0.00040	-0.00005				
8192	0.14078	0.00298	0.00239	0.00172	0.00090	0.00187	0.00111	0.00129	0.00026	0.00028	-0.00022	-0.00156					
4096	0.13718	0.00387	0.00201	0.00179	0.00048	0.00152	0.00027	-0.00022	0.00017	0.00005	-0.00039						
2048	0.17301	0.00482	0.00355	0.00177	0.00262	0.00261	0.00122	-0.00010	0.00096	-0.00122							
1024	0.24251	0.00751	0.00745	0.00677	0.00389	0.00876	0.00220	0.00314	0.00335								
512	0.28303	0.00997	0.01131	0.00861	0.00519	0.01304	0.00171	0.00483									
256	0.34381	0.01138	0.01081	0.00867	0.00066	0.01298	0.00100										
128	0.46297	0.01637	0.01725	0.01086	-0.00033	0.01372											
64	0.61046	0.01736	0.01690	0.01094	-0.00161												
32	0.78886	0.02422	0.01663	0.00667													
16	0.97462	0.02304	0.00810														
8	1.18965	0.02668															
4	1.39488																

(a) $\mathcal{P}^{\mathcal{C}}$ for Scene: [**Veach Bidir**] Algorithm: [**PT**] Metric: [**NQM**] True GT: [**BDPT @ 524288 spp**].

BDPT	2	4	8	16	32	64	128	256	512	1024	2048	4096	8192	16384	32768	65536	131072
GT (BDPT)																	
262144	0.00035	0.00033	0.00053	0.00041	0.00052	0.00067	0.00127	0.00062	0.00092	0.00254	-0.00098	-0.00375	0.00012	-0.00038	0.00543	0.00104	-0.00015
131072	0.00066	0.00077	0.00074	0.00013	0.00082	0.00006	0.00069	-0.00051	-0.00026	-0.00132	-0.00159	-0.00026	-0.00078	0.00340	0.00493	0.00042	
65536	0.00091	0.00072	0.00096	0.00110	0.00047	0.00086	0.00028	0.00133	0.00036	-0.00273	-0.00675	-0.00676	-0.00803	-0.00941	-0.00579		
32768	0.00096	0.00047	0.00086	0.00119	0.00084	-0.00004	0.00133	0.00064	-0.00023	-0.00397	-0.00813	-0.01888	-0.01756	-0.00939			
16384	0.00235	0.00348	0.00419	0.00137	0.00909	0.00814	0.01269	0.00731	0.00472	0.00838	-0.00756	-0.04068	-0.04159				
8192	0.00268	0.00340	0.00503	0.00479	0.00557	0.00878	0.00686	0.00764	0.00230	-0.00275	-0.01903	-0.03930					
4096	0.00057	0.00053	0.00154	0.00046	0.00072	0.00032	0.00156	-0.00410	-0.00720	-0.01450	-0.03729						
2048	0.00305	0.00468	0.00555	-0.00038	0.01193	0.00899	0.01622	-0.00441	0.00554	-0.01119							
1024	0.01162	0.01386	0.02088	0.02218	0.03171	0.04801	0.04771	0.02626	0.03010								
512	0.02000	0.02499	0.03368	0.03189	0.04489	0.07473	0.05970	0.01368									
256	0.01868	0.02658	0.03668	0.03762	0.04883	0.08089	0.06519										
128	0.02323	0.02849	0.03974	0.01418	0.05674	0.09462											
64	0.02325	0.03141	0.03264	0.00809	0.03007												
32	0.01508	0.01837	0.01043	-0.03691													
16	-0.00574	-0.01523	-0.03758														
8	-0.03421	-0.05657															
4	-0.09111																

(b) $\mathcal{P}^{\mathcal{C}}$ for Scene: [**Veach Bidir**] Algorithm: [**BDPT**] Metric: [**NQM**] True GT: [**BDPT @ 524288 spp**].

PSSMLT	2	4	8	16	32	64	128	256	512	1024	2048	4096	8192	16384
GT (BDPT)														
262144	0.00065	0.00069	0.00072	0.00074	0.00087	0.00081	0.00072	0.00055	0.00083	0.00081	0.00074	0.00140	0.00073	0.00009
131072	0.00132	0.00100	0.00080	0.00055	0.00035	0.00063	0.00057	0.00049	0.00051	0.00024	0.00019	0.00111	0.00055	0.00020
65536	0.00103	0.00096	0.00065	0.00073	0.00099	0.00118	0.00100	0.00027	-0.00002	-0.00053	-0.00070	0.00048	-0.00047	-0.00091
32768	0.00123	0.00100	0.00093	0.00076	0.00070	0.00100	0.00108	-0.00189	-0.00125	-0.00200	-0.00291	-0.00252	-0.00376	-0.00360
16384	0.00289	0.00322	0.00384	0.00456	0.00582	0.00804	0.00859	0.00440	-0.00120	-0.00046	-0.00466	-0.00033	-0.00638	
8192	0.00235	0.00301	0.00392	0.00558	0.00620	0.00712	0.00884	0.00662	0.00213	-0.00061	-0.00473	-0.00463		
4096	0.00096	0.00056	-0.00007	0.00054	0.00150	0.00220	0.00172	-0.00526	-0.00857	-0.00949	-0.01531			
2048	0.00335	0.00378	0.00513	0.00710	0.00618	0.01014	0.01180	0.00654	-0.01885	-0.01410				
1024	0.00900	0.01229	0.01758	0.02668	0.03447	0.04538	0.05285	0.02725	-0.01351					
512	0.01402	0.01885	0.02915	0.04128	0.05297	0.07199	0.08769	0.03171						
256	0.01532	0.02099	0.03073	0.04403	0.05321	0.07027	0.08736							
128	0.01975	0.02416	0.03455	0.04749	0.05746	0.07346								
64	0.01743	0.02318	0.03425	0.04592	0.05096									
32	0.01112	0.01185	0.01728	0.01190										
16	-0.00650	-0.01782	-0.02597											
8	-0.02297	-0.04873												
4	-0.06559													

(c) $\mathcal{P}^{\mathcal{C}}$ for Scene: [**Veach Bidir**] Algorithm: [**PSSMLT**] Metric: [**NQM**] True GT: [**BDPT @ 524288 spp**].

MLT	2	4	8	16	32	64	128	256	512	1024	2048	4096	8192	16384
GT (BDPT)														
262144	0.00311	0.00090	0.00060	0.00063	0.00052	0.00077	0.00079	0.00068	0.00079	0.00123	0.00099	0.00106	0.00117	0.00102
131072	0.00992	0.00234	0.00141	0.00108	0.00069	0.00109	0.00086	0.00057	0.00018	0.00015	0.00008	0.00002	0.00013	0.00012
65536	0.00910	0.00259	0.00158	0.00131	0.00128	0.00165	0.00145	0.00127	0.00131	0.00155	0.00162	0.00153	0.00171	0.00143
32768	0.00970	0.00245	0.00145	0.00140	0.00179	0.00209	0.00179	0.00127	0.00153	0.00110	0.00099	0.00121	0.00109	0.00109
16384	0.01136	0.00356	0.00281	0.00287	0.00228	0.00377	0.00491	0.00575	0.00715	0.00844	0.00845	0.00912	0.00966	
8192	0.01242	0.00328	0.00309	0.00381	0.00341	0.00434	0.00644	0.00774	0.00858	0.00980	0.01020	0.01045		
4096	0.01031	0.00246	0.00151	0.00147	0.00144	0.00170	0.00105	0.00293	0.00153	0.00132	0.00263			
2048	0.01477	0.00534	0.00496	0.00386	0.00469	0.00524	0.00746	0.00788	0.01004	0.01344				
1024	0.01958	0.00847	0.00884	0.01123	0.01159	0.01720	0.02749	0.03790	0.04716					
512	0.03465	0.01513	0.01579	0.01781	0.01664	0.02677	0.04023	0.05897						
256	0.03210	0.01524	0.01566	0.01924	0.01918	0.02919	0.04755							
128	0.05076	0.01941	0.01802	0.01901	0.02025	0.03380								
64	0.06107	0.02262	0.01993	0.01919	0.01493									
32	0.06934	0.02560	0.02117	0.01259										
16	0.07318	0.01330	0.00400											
8	0.12066	0.01915												
4	0.20135													

(d) $\mathcal{P}^{\mathcal{C}}$ for Scene: [**Veach Bidir**] Algorithm: [**MLT**] Metric: [**NQM**] True GT: [**BDPT @ 524288 spp**].

M-MLT	2	4	8	16	32	64	128	256	512	1024	2048	4096	8192	16384
GT (BDPT)														
262144	0.00509	0.00110	0.00072	0.00052	0.00040	0.00029	0.00048	0.00040	0.00055	0.00047	0.00056	0.00073	0.00075	0.00081
131072	0.01210	0.00240	0.00167	0.00114	0.00094	0.00082	0.00099	0.00012	0.00054	0.00012	0.00010	0.00039	0.00033	-0.00024
65536	0.01088	0.00215	0.00133	0.00062	0.00093	0.00049	0.00100	0.00091	0.00094	0.00084	0.00089	0.00120	0.00108	0.00106
32768	0.01091	0.00235	0.00157	0.00084	0.00058	0.00052	0.00106	0.00077	0.00068	0.00036	0.00140	0.00088	0.00114	0.00025
16384	0.01361	0.00311	0.00234	0.00283	0.00283	0.00358	0.00464	0.00464	0.00634	0.00788	0.00913	0.00957	0.01042	
8192	0.01560	0.00381	0.00330	0.00362	0.00418	0.00560	0.00644	0.00708	0.00880	0.00863	0.01004	0.01062		
4096	0.01251	0.00163	0.00143	0.00068	0.00045	-0.00005	0.00063	0.00109	0.00049	-0.00006	-0.00068			
2048	0.01061	0.00288	0.00239	0.00364	0.00453	0.00504	0.00609	0.00816	0.00999	0.01205				
1024	0.02689	0.01084	0.00796	0.01130	0.01483	0.02032	0.02724	0.03728	0.04485					
512	0.03762	0.01808	0.01408	0.01856	0.02472	0.03420	0.04271	0.05916						
256	0.03954	0.01505	0.01451	0.01806	0.02595	0.03698	0.04674							
128	0.06122	0.02406	0.01922	0.02368	0.02949	0.03864								
64	0.07830	0.03243	0.02323	0.02719	0.03276									
32	0.11489	0.03004	0.02035	0.01985										
16	0.10441	0.02208	0.00757											
8	0.16994	0.03481												
4	0.24928													

(e) $\mathcal{P}^{\mathcal{C}}$ for Scene: [**Veach Bidir**] Algorithm: [**M-MLT**] Metric: [**NQM**] True GT: [**BDPT @ 524288 spp**].

ERPT	2	4	8	16	32	64	128	256	512	1024	2048	4096	8192	16384	32768	65536	131072
GT (BDPT)																	
262144	0.00035	0.00060	0.00058	0.00054	0.00085	0.00044	0.00083	0.00014	0.00042	0.00027	0.00016	-0.00003	-0.00017	-0.00037	0.00042	0.00041	0.00036
131072	0.00025	0.00045	0.00059	0.00037	0.00092	0.00073	0.00080	0.00047	0.00030	0.00101	0.00071	0.00050	0.00023	0.00005	0.00062	0.00063	
65536	0.00052	0.00094	0.00081	0.00077	0.00045	0.00098	0.00058	-0.00015	0.00005	0.00024	-0.00011	0.00032	-0.00022	-0.00009	0.00025		
32768	0.00059	0.00099	0.00102	0.00115	0.00055	-0.00010	0.00021	0.00034	-0.00001	0.00070	-0.00017	0.00004	-0.00011	-0.00012			
16384	0.00546	0.00505	0.00564	0.00522	0.00674	0.00060	0.00430	-0.00101	-0.00125	-0.00030	-0.00033	-0.00253	-0.00081				
8192	0.00631	0.00565	0.00590	0.00518	0.00369	-0.00054	0.00294	0.00100	0.00043	-0.00034	-0.00014	-0.00100					
4096	-0.00006	0.00135	0.00007	0.00180	-0.00025	-0.00081	0.00058	0.00019	-0.00202	-0.00130	-0.00061						
2048	0.00735	0.00764	0.00909	0.00664	0.01072	0.00007	0.00696	-0.00373	-0.00315	-0.00387							
1024	0.02902	0.02999	0.03312	0.03095	0.02008	-0.00513	0.01909	-0.00192	-0.00824								
512	0.04993	0.04955	0.05105	0.04579	0.02947	-0.01608	0.02260	-0.01380									
256	0.05134	0.05058	0.05206	0.05000	0.02367	-0.02531	0.01145										
128	0.05864	0.05637	0.05600	0.05939	0.02689	-0.06239											
64	0.05253	0.04830	0.04325	0.04577	0.00423												
32	0.01718	0.00300	-0.00092	-0.00303													
16	-0.05972	-0.05498	-0.06756														
8	-0.13279	-0.13983															
4	-0.25980																

(f) $\mathcal{P}^{\mathcal{C}}$ for Scene: [**Veach Bidir**] Algorithm: [**ERPT**] Metric: [**NQM**] True GT: [**BDPT @ 524288 spp**].

M-ERPT	2	4	8	16	32	64	128	256	512	1024	2048	4096	8192	16384	32768	65536	131072
GT (BDPT)																	
262144	0.00022	0.00055	0.00026	0.00032	0.00028	0.00011	-0.00025	0.00045	-0.00005	0.00013	-0.00007	0.00025	0.00036	0.00041	0.00045	0.00022	0.00040
131072	0.00038	0.00022	0.00019	0.00000	0.00046	0.00030	-0.00006	0.00049	0.00054	0.00034	0.00041	0.00045	0.00091	0.00076	0.00049	0.00080	
65536	0.00049	0.00083	0.00014	0.00033	0.00002	0.00042	-0.00021	0.00002	-0.00031	0.00065	-0.00002	-0.00006	0.00032	0.00058	0.00044		
32768	0.00088	0.00033	-0.00012	-0.00026	0.00050	0.00026	0.00018	0.00000	0.00008	0.00013	0.00019	-0.00008	0.00039	0.00034			
16384	0.00584	0.00490	-0.00010	-0.00038	0.00094	-0.00165	-0.00023	-0.00109	-0.00031	-0.00187	-0.00122	-0.00130	-0.00113				
8192	0.00628	0.00641	-0.00007	-0.00016	0.00122	0.00050	-0.00315	0.00017	-0.00122	-0.00072	-0.00153	-0.00114					
4096	0.00027	0.00096	-0.00305	-0.00117	-0.00100	-0.00183	-0.00208	-0.00172	-0.00279	-0.00167	-0.00301						
2048	0.01054	0.00789	-0.00319	-0.00599	0.00048	-0.00480	-0.00101	-0.00479	-0.00100	-0.00563							
1024	0.03101	0.03337	-0.01064	-0.00416	0.00196	-0.01069	-0.01357	-0.01231	-0.00913								
512	0.04625	0.04722	-0.02932	-0.01783	-0.00801	-0.02882	-0.02448	-0.02937									
256	0.05167	0.05829	-0.03842	-0.02860	-0.01287	-0.03683	-0.03853										
128	0.06072	0.06201	-0.08277	-0.06631	-0.05152	-0.08066											
64	0.05116	0.05613	-0.11188	-0.09291	-0.07596												
32	0.01515	0.01085	-0.12780	-0.10985													
16	-0.05513	-0.04906	-0.17919														
8	-0.12487	-0.13127															
4	-0.25546																

(g) $\mathcal{P}^{\mathcal{C}}$ for Scene: [**Veach Bidir**] Algorithm: [**M-ERPT**] Metric: [**NQM**] True GT: [**BDPT @ 524288 spp**].

Table 73

16.4 Veach Door

PT	2	4	8	16	32	64	128	256	512	1024	2048	4096	8192	16384	32768	65536	131072
GT (BDPT)																	
262144	-0.00065	-0.00117	0.00045	0.00022	-0.00009	0.00026	-0.00002	0.00009	-0.00014	0.00026	0.00020	-0.00009	-0.00044	-0.00072	-0.00034	-0.00155	-0.00390
131072	-0.00044	-0.00009	0.00061	0.00013	0.00007	-0.00010	-0.00026	-0.00031	-0.00021	-0.00019	-0.00038	-0.00047	-0.00070	-0.00127	-0.00360	-0.00447	
65536	-0.00076	-0.00110	0.00062	0.00065	-0.00006	0.00007	-0.00027	-0.00035	-0.00023	-0.00057	-0.00026	-0.00099	-0.00231	-0.00351	-0.00632		
32768	-0.00066	-0.00185	0.00178	0.00047	0.00014	0.00018	-0.00034	-0.00046	-0.00055	-0.00079	-0.00186	-0.00262	-0.00449	-0.00753			
16384	-0.00269	-0.00688	0.00406	0.00117	0.00127	0.00091	-0.00005	0.00014	-0.00124	-0.00173	-0.00256	-0.00521	-0.00908				
8192	-0.00459	-0.01270	0.00890	0.00243	0.00195	0.00176	-0.00068	-0.00004	-0.00115	-0.00218	-0.00533	-0.01002					
4096	-0.00672	-0.01650	0.01153	0.00320	0.00111	0.00112	-0.00076	-0.00196	-0.00388	-0.00684	-0.01313						
2048	-0.01264	-0.04291	0.02653	0.00808	0.00505	0.00139	0.00187	-0.00278	-0.00783	-0.01214							
1024	-0.02040	-0.06836	0.04227	0.01328	0.00581	-0.00031	-0.00364	-0.00942	-0.01702								
512	-0.04341	-0.12068	0.06392	0.02118	0.01161	0.00079	-0.00669	-0.01816									
256	-0.07262	-0.22355	0.09708	0.03260	0.00999	-0.01084	-0.02047										
128	-0.11659	-0.34470	0.14846	0.03975	0.00760	-0.03032											
64	-0.18058	-0.58573	0.21448	0.04261	-0.01399												
32	-0.28836	-1.03752	0.27233	0.04409													
16	-0.41710	-1.82714	0.24498														
8	-0.67509	-2.93403															
4	-0.96434																

(a) $\mathcal{P}^{\mathcal{C}}$ for Scene: [**Veach Door**] Algorithm: [**PT**] Metric: [**NQM**] True GT: [**BDPT @ 524288 spp**].

BDPT	2	4	8	16	32	64	128	256	512	1024	2048	4096	8192	16384	32768	65536	131072
GT (BDPT)																	
262144	-0.00270	0.00076	0.00006	0.00041	0.00010	0.00006	-0.00002	0.00027	-0.00000	-0.00053	-0.00113	-0.00096	-0.00145	-0.00349	-0.00294	-0.00518	-0.01083
131072	-0.00061	0.00081	0.00035	0.00011	-0.00003	-0.00008	-0.00007	-0.00009	-0.00070	-0.00131	-0.00179	-0.00103	-0.00401	-0.00802	-0.00686	-0.01499	
65536	-0.00299	0.00110	0.00041	0.00014	-0.00025	-0.00015	-0.00037	-0.00029	-0.00104	-0.00197	-0.00355	-0.00562	-0.01032	-0.01717	-0.02221		
32768	-0.00429	0.00206	0.00097	-0.00000	0.00000	-0.00003	-0.00084	-0.00050	-0.00168	-0.00398	-0.00569	-0.01081	-0.01859	-0.03498			
16384	-0.00934	0.00396	0.00132	0.00065	0.00050	-0.00015	-0.00023	-0.00105	-0.00413	-0.00681	-0.01271	-0.02052	-0.03737				
8192	-0.02193	0.00819	0.00249	0.00180	0.00152	-0.00065	-0.00112	-0.00400	-0.00707	-0.01494	-0.02442	-0.04167					
4096	-0.03460	0.00950	0.00330	0.00218	0.00005	-0.00154	-0.00415	-0.00627	-0.01796	-0.02888	-0.05106						
2048	-0.07181	0.01978	0.00854	0.00432	0.00152	-0.00165	-0.00887	-0.01623	-0.02852	-0.05320							
1024	-0.11586	0.03852	0.01175	0.00475	-0.00072	-0.00870	-0.01848	-0.03494	-0.06191								
512	-0.22473	0.05843	0.02005	0.00830	-0.00203	-0.01682	-0.03717	-0.06680									
256	-0.38152	0.09514	0.03292	0.00908	-0.01351	-0.04265	-0.07332										
128	-0.61899	0.12978	0.03858	-0.00159	-0.03717	-0.08670											
64	-1.31986	0.18505	0.03968	-0.02253	-0.08226												
32	-2.74131	0.23824	0.02601	-0.07136													
16	-1.33625	0.19279	-0.05581														
8	-0.34590	0.17459															
4	-0.11344																

(b) $\mathcal{P}^{\mathcal{C}}$ for Scene: [**Veach Door**] Algorithm: [**BDPT**] Metric: [**NQM**] True GT: [**BDPT @ 524288 spp**].

PSSMLT	2	4	8	16	32	64	128	256	512	1024	2048	4096	8192	16384
GT (BDPT)														
262144	0.00218	0.00007	-0.00002	-0.00001	-0.00003	-0.00012	-0.00029	-0.00052	-0.00057	-0.00112	-0.00078	-0.00181	-0.00252	-0.00317
131072	0.00077	0.00016	0.00021	0.00016	-0.00009	-0.00007	-0.00042	-0.00070	-0.00083	-0.00149	-0.00225	-0.00343	-0.00516	-0.00665
65536	0.00104	0.00035	0.00023	0.00010	-0.00027	-0.00052	-0.00071	-0.00104	-0.00135	-0.00296	-0.00482	-0.00650	-0.01147	-0.01608
32768	0.00384	0.00093	0.00033	0.00002	-0.00012	-0.00065	-0.00119	-0.00220	-0.00347	-0.00566	-0.01099	-0.01614	-0.02416	-0.03261
16384	0.00903	0.00131	0.00046	0.00049	-0.00026	-0.00080	-0.00158	-0.00244	-0.00551	-0.00803	-0.01492	-0.02472	-0.03854	
8192	0.01165	0.00278	0.00092	0.00020	-0.00070	-0.00235	-0.00378	-0.00746	-0.01164	-0.01838	-0.03224	-0.05002		
4096	0.01562	0.00533	0.00308	0.00118	-0.00110	-0.00253	-0.00593	-0.01113	-0.02032	-0.03934	-0.06009			
2048	0.03812	0.00687	0.00270	-0.00068	-0.00361	-0.00629	-0.01395	-0.02453	-0.04088	-0.06634				
1024	0.05658	0.01119	0.00382	-0.00131	-0.00842	-0.01548	-0.02786	-0.05115	-0.08164					
512	0.11124	0.02213	0.00648	-0.00332	-0.01562	-0.03109	-0.05803	-0.09650						
256	0.16050	0.02708	0.00630	-0.01444	-0.03347	-0.06226	-0.10542							
128	0.23130	0.03948	0.00233	-0.03256	-0.06996	-0.12195								
64	0.30215	0.04025	-0.01712	-0.07464	-0.13934									
32	0.40887	0.03185	-0.06685	-0.15214										
16	0.44009	-0.03081	-0.17884											
8	0.43526	-0.15127												
4	0.22695													

(c) $\mathcal{P}^{\mathcal{C}}$ for Scene: [**Veach Door**] Algorithm: [**PSSMLT**] Metric: [**NQM**] True GT: [**BDPT @ 524288 spp**].

MLT	2	4	8	16	32	64	128	256	512	1024	2048	4096	8192	16384
GT (BDPT)														
262144	0.00117	0.00039	0.00032	0.00012	-0.00007	-0.00000	-0.00012	-0.00054	-0.00012	0.00008	-0.00030	-0.00199	-0.00293	-0.00456
131072	0.00103	0.00011	0.00006	0.00007	-0.00011	-0.00010	-0.00056	-0.00051	-0.00026	-0.00092	-0.00238	-0.00432	-0.00431	-0.00726
65536	0.00073	0.00047	-0.00034	-0.00035	-0.00051	-0.00094	-0.00078	-0.00145	-0.00141	-0.00248	-0.00459	-0.00899	-0.01123	-0.01709
32768	0.00108	0.00041	-0.00017	0.00025	-0.00057	-0.00082	-0.00101	-0.00184	-0.00313	-0.00439	-0.00810	-0.01748	-0.02368	-0.03352
16384	0.00409	0.00109	0.00048	0.00071	-0.00032	-0.00018	-0.00086	-0.00311	-0.00524	-0.00849	-0.01914	-0.02873	-0.04194	
8192	0.00738	0.00215	0.00081	0.00047	-0.00099	-0.00051	-0.00389	-0.00736	-0.01252	-0.02292	-0.03603	-0.05590		
4096	0.00993	0.00327	0.00113	0.00107	-0.00096	-0.00257	-0.00781	-0.01632	-0.02416	-0.04115	-0.06715			
2048	0.02249	0.00938	0.00469	-0.00015	-0.00149	-0.00354	-0.01341	-0.02555	-0.04451	-0.06840				
1024	0.03503	0.00927	0.00337	-0.00207	-0.00703	-0.01552	-0.02836	-0.05410	-0.08353					
512	0.06150	0.01983	0.00791	-0.00619	-0.01488	-0.02763	-0.06207	-0.10121						
256	0.09817	0.02571	-0.00117	-0.01822	-0.03415	-0.06210	-0.11472							
128	0.14837	0.03663	-0.00538	-0.04097	-0.07505	-0.11951								
64	0.18269	0.03838	-0.03329	-0.09412	-0.14556									
32	0.23971	0.01055	-0.09744	-0.18647										
16	0.25146	-0.06534	-0.22151											
8	0.21719	-0.20379												
4	0.04607													

(d) $\mathcal{P}^{\mathcal{C}}$ for Scene: [**Veach Door**] Algorithm: [**MLT**] Metric: [**NQM**] True GT: [**BDPT @ 524288 spp**].

M-MLT	2	4	8	16	32	64	128	256	512	1024	2048	4096	8192	16384
GT (BDPT)														
262144	0.00047	0.00020	0.00009	0.00020	0.00030	-0.00013	-0.00001	0.00003	-0.00023	-0.00003	-0.00101	-0.00116	-0.00219	-0.00128
131072	0.00047	0.00040	0.00012	0.00015	0.00007	-0.00047	-0.00037	-0.00028	-0.00058	-0.00011	-0.00177	-0.00276	-0.00506	-0.00679
65536	-0.00030	0.00052	0.00010	0.00006	0.00006	-0.00083	-0.00079	-0.00108	-0.00179	-0.00205	-0.00412	-0.00699	-0.01179	-0.01439
32768	0.00178	0.00055	-0.00019	0.00075	0.00014	-0.00117	-0.00069	-0.00127	-0.00278	-0.00476	-0.01063	-0.01277	-0.02255	-0.02538
16384	0.00350	0.00179	0.00072	-0.00027	-0.00029	-0.00111	-0.00110	-0.00314	-0.00459	-0.00843	-0.01817	-0.02452	-0.04002	
8192	0.00507	0.00196	0.00102	0.00046	0.00007	-0.00193	-0.00212	-0.00674	-0.01085	-0.01880	-0.03674	-0.04480		
4096	0.00636	0.00336	0.00094	0.00036	-0.00138	-0.00387	-0.00887	-0.01442	-0.02450	-0.03904	-0.06801			
2048	0.01516	0.00608	0.00269	0.00106	-0.00128	-0.00645	-0.01533	-0.02771	-0.04392	-0.06749				
1024	0.02504	0.00956	0.00133	0.00016	-0.00956	-0.01672	-0.03050	-0.05244	-0.08323					
512	0.04968	0.01627	0.00489	-0.00255	-0.01778	-0.03531	-0.06330	-0.09589						
256	0.06599	0.02397	0.00154	-0.01358	-0.03849	-0.07149	-0.11490							
128	0.10239	0.02375	-0.00750	-0.03896	-0.07725	-0.13391								
64	0.12945	0.01714	-0.03310	-0.09075	-0.15505									
32	0.16211	0.00180	-0.09523	-0.18886										
16	0.12905	-0.07432	-0.22350											
8	0.08274	-0.20493												
4	-0.10853													

(e) $\mathcal{P}^{\mathcal{C}}$ for Scene: [**Veach Door**] Algorithm: [**M-MLT**] Metric: [**NQM**] True GT: [**BDPT @ 524288 spp**].

ERPT	2	4	8	16	32	64	128	256	512	1024	2048	4096	8192	16384	32768	65536	131072
GT (BDPT)																	
262144	-0.00023	-0.00004	-0.00000	0.00012	-0.00039	0.00005	-0.00015	-0.00047	-0.00045	-0.00034	-0.00024	-0.00006	-0.00039	-0.00041	-0.00039	0.00008	-0.00045
131072	-0.00075	-0.00058	-0.00022	-0.00062	-0.00052	-0.00066	-0.00066	-0.00004	-0.00094	-0.00074	-0.00033	-0.00058	-0.00049	-0.00054	-0.00052	-0.00072	
65536	-0.00078	-0.00104	-0.00078	-0.00115	-0.00121	-0.00126	-0.00072	-0.00092	-0.00139	-0.00092	-0.00057	-0.00140	-0.00103	-0.00119	-0.00070		
32768	-0.00174	-0.00176	-0.00177	-0.00134	-0.00180	-0.00165	-0.00162	-0.00169	-0.00239	-0.00181	-0.00209	-0.00197	-0.00202	-0.00178			
16384	-0.00135	-0.00224	-0.00176	-0.00297	-0.00414	-0.00288	-0.00287	-0.00387	-0.00308	-0.00316	-0.00365	-0.00469	-0.00237				
8192	-0.00384	-0.00298	-0.00402	-0.00614	-0.00452	-0.00541	-0.00516	-0.00692	-0.00624	-0.00706	-0.00518	-0.00502					
4096	-0.00979	-0.01282	-0.00946	-0.01286	-0.01273	-0.01254	-0.01324	-0.01509	-0.01251	-0.01477	-0.01402						
2048	-0.01754	-0.01722	-0.02019	-0.02544	-0.02250	-0.02308	-0.02354	-0.02401	-0.02392	-0.02476							
1024	-0.03452	-0.03741	-0.04171	-0.05266	-0.04679	-0.05099	-0.04839	-0.05086	-0.04806								
512	-0.06641	-0.07140	-0.08376	-0.09361	-0.09200	-0.09290	-0.09287	-0.09388									
256	-0.12223	-0.12723	-0.14174	-0.16385	-0.15851	-0.16122	-0.16184										
128	-0.20336	-0.22087	-0.23905	-0.26683	-0.26034	-0.25905											
64	-0.32725	-0.33974	-0.36821	-0.39652	-0.38896												
32	-0.49620	-0.51367	-0.54209	-0.57615													
16	-0.71855	-0.73942	-0.76861														
8	-0.99599	-1.02109															
4	-1.34572																

(f) $\mathcal{P}^{\mathcal{C}}$ for Scene: [**Veach Door**] Algorithm: [**ERPT**] Metric: [**NQM**] True GT: [**BDPT @ 524288 spp**].

M-ERPT	2	4	8	16	32	64	128	256	512	1024	2048	4096	8192	16384	32768	65536	131072
GT (BDPT)																	
262144	0.00005	-0.00029	-0.00011	0.00011	-0.00019	-0.00011	-0.00033	-0.00036	-0.00044	-0.00027	0.00005	0.00005	-0.00012	-0.00009	0.00007	-0.00040	-0.00047
131072	-0.00037	-0.00067	-0.00019	-0.00050	-0.00034	-0.00052	-0.00060	-0.00046	-0.00002	-0.00069	-0.00082	-0.00075	-0.00026	-0.00024	-0.00027	-0.00058	
65536	-0.00065	-0.00107	-0.00042	-0.00133	-0.00129	-0.00067	-0.00172	-0.00166	-0.00077	-0.00098	-0.00102	-0.00037	-0.00099	-0.00120	-0.00112		
32768	-0.00097	-0.00199	-0.00095	-0.00187	-0.00218	-0.00176	-0.00154	-0.00189	-0.00204	-0.00175	-0.00184	-0.00198	-0.00195	-0.00199			
16384	-0.00185	-0.00323	-0.00180	-0.00376	-0.00236	-0.00311	-0.00276	-0.00196	-0.00152	-0.00317	-0.00344	-0.00369	-0.00266				
8192	-0.00284	-0.00461	-0.00481	-0.00473	-0.00564	-0.00321	-0.00537	-0.00533	-0.00604	-0.00494	-0.00651	-0.00571					
4096	-0.00744	-0.00926	-0.01189	-0.00968	-0.01169	-0.00933	-0.01185	-0.01231	-0.01075	-0.01183	-0.01189						
2048	-0.01338	-0.02004	-0.02070	-0.02011	-0.02258	-0.01892	-0.02067	-0.02054	-0.02292	-0.02100							
1024	-0.02998	-0.04218	-0.04254	-0.04361	-0.04845	-0.04311	-0.04627	-0.04800	-0.04478								
512	-0.05872	-0.07374	-0.07511	-0.07820	-0.08753	-0.08125	-0.08290	-0.08860									
256	-0.11163	-0.13530	-0.14185	-0.14544	-0.15179	-0.14810	-0.15245										
128	-0.18953	-0.22143	-0.23943	-0.24258	-0.25316	-0.23696											
64	-0.30761	-0.35240	-0.36204	-0.37115	-0.38288												
32	-0.47480	-0.52159	-0.53923	-0.54736													
16	-0.69631	-0.74674	-0.76750														
8	-0.96750	-1.03026															
4	-1.31301																

(g) $\mathcal{P}^{\mathcal{C}}$ for Scene: [**Veach Door**] Algorithm: [**M-ERPT**] Metric: [**NQM**] True GT: [**BDPT @ 524288 spp**].

Table 74

16.5 Sponza

PT	2	4	8	16	32	64	128	256	512	1024	2048	4096	8192
GT (PT)													
16384	0.00077	-0.00014	0.00031	0.00020	0.00020	-0.00048	-0.00010	-0.00137	-0.00219	-0.00477	-0.00618	-0.00846	-0.01514
8192	0.00096	-0.00001	-0.00007	-0.00012	-0.00076	-0.00217	-0.00236	-0.00327	-0.00807	-0.01218	-0.01723	-0.02740	
4096	-0.00012	-0.00058	-0.00042	-0.00148	-0.00113	-0.00367	-0.00501	-0.00831	-0.01448	-0.02513	-0.03707		
2048	0.00261	0.00078	0.00036	-0.00071	-0.00197	-0.00731	-0.00996	-0.02032	-0.03079	-0.04888			
1024	0.00532	0.00111	-0.00016	-0.00249	-0.00533	-0.01416	-0.02281	-0.03587	-0.05919				
512	0.00488	-0.00156	-0.00171	-0.00944	-0.01346	-0.02700	-0.04464	-0.06779					
256	0.01838	0.00549	-0.00037	-0.01428	-0.02845	-0.04991	-0.08194						
128	0.02310	0.00549	-0.01011	-0.02888	-0.05716	-0.09840							
64	0.03931	-0.00100	-0.01959	-0.06121	-0.10675								
32	0.06604	0.00653	-0.04051	-0.11667									
16	0.08807	-0.01262	-0.10346										
8	0.14464	-0.04368											
4	0.14505												

(a) $\mathcal{P}^{\mathcal{C}}$ for Scene: [**Sponza**] Algorithm: [**PT**] Metric: [**NQM**] True GT: [**PT @ 32768 spp**].

BDPT	2	4	8	16	32	64	128	256	512	1024	2048	4096	8192
GT (PT)													
16384	0.00070	0.00122	0.00044	0.00023	-0.00017	-0.00031	-0.00025	-0.00024	-0.00182	-0.00376	-0.00352	-0.00726	-0.01018
8192	-0.00065	0.00085	-0.00019	-0.00012	-0.00030	-0.00108	-0.00153	-0.00261	-0.00518	-0.00947	-0.01271	-0.02211	
4096	0.00066	0.00025	-0.00038	-0.00117	-0.00229	-0.00232	-0.00413	-0.00764	-0.01065	-0.01896	-0.03274		
2048	0.00162	0.00149	-0.00034	-0.00151	-0.00322	-0.00507	-0.00924	-0.01564	-0.02585	-0.04092			
1024	0.00912	0.00478	0.00046	-0.00064	-0.00406	-0.00983	-0.01805	-0.03190	-0.04942				
512	0.00825	0.00144	-0.00163	-0.00682	-0.01352	-0.02017	-0.03479	-0.05925					
256	0.02962	0.01244	-0.00037	-0.00725	-0.02105	-0.03563	-0.06558						
128	0.03654	0.01327	-0.00762	-0.01737	-0.04426	-0.07827							
64	0.07406	0.01944	-0.01018	-0.04195	-0.08759								
32	0.12541	0.03250	-0.02358	-0.08499									
16	0.15739	0.01897	-0.07312										
8	0.28349	0.03117											
4	0.32253												

(b) $\mathcal{P}^{\mathcal{C}}$ for Scene: [**Sponza**] Algorithm: [**BDPT**] Metric: [**NQM**] True GT: [**PT @ 32768 spp**].

PSSMLT	2	4	8	16	32	64	128	256	512	1024	2048	4096	8192
GT (PT)													
16384	0.00019	0.00036	0.00023	-0.00020	-0.00014	-0.00012	-0.00067	-0.00046	-0.00086	-0.00134	-0.00134	-0.00252	-0.00168
8192	0.00008	0.00004	-0.00015	-0.00073	-0.00064	-0.00065	-0.00191	-0.00225	-0.00262	-0.00314	-0.00429	-0.00655	
4096	0.00061	-0.00005	-0.00072	-0.00145	-0.00163	-0.00206	-0.00364	-0.00410	-0.00663	-0.00765	-0.01145		
2048	0.00357	-0.00026	-0.00109	-0.00195	-0.00354	-0.00525	-0.00826	-0.01197	-0.01634	-0.02063			
1024	0.00802	0.00270	-0.00015	-0.00393	-0.00538	-0.00892	-0.01564	-0.02203	-0.03290				
512	0.00701	-0.00096	-0.00463	-0.00800	-0.01343	-0.01795	-0.03004	-0.04418					
256	0.02522	0.00935	-0.00084	-0.01116	-0.02244	-0.03462	-0.05665						
128	0.03959	0.00846	-0.00798	-0.02389	-0.04660	-0.07266							
64	0.06908	0.01251	-0.01907	-0.04698	-0.08773								
32	0.12792	0.02844	-0.03171	-0.08958									
16	0.17100	0.00578	-0.09603										
8	0.25690	0.00291											
4	0.30120												

(c) $\mathcal{P}^{\mathcal{C}}$ for Scene: [**Sponza**] Algorithm: [**PSSMLT**] Metric: [**NQM**] True GT: [**PT @ 32768 spp**].

MLT	2	4	8	16	32	64	128	256	512	1024	2048	4096	8192
GT (PT)													
16384	0.00030	-0.00004	-0.00035	-0.00005	-0.00057	0.00010	-0.00095	-0.00129	-0.00065	-0.00121	-0.00171	-0.00194	-0.00225
8192	0.00080	0.00040	-0.00004	-0.00014	-0.00054	-0.00018	-0.00328	-0.00254	-0.00380	-0.00423	-0.00532	-0.00531	
4096	-0.00078	-0.00175	-0.00181	-0.00166	-0.00287	-0.00353	-0.00553	-0.00732	-0.01001	-0.01182	-0.01219		
2048	0.00001	-0.00098	-0.00133	-0.00222	-0.00555	-0.01030	-0.01407	-0.01692	-0.02345	-0.02722			
1024	0.00216	-0.00195	-0.00225	-0.00609	-0.01260	-0.01841	-0.02836	-0.03513	-0.04269				
512	0.00027	-0.00247	-0.00734	-0.01311	-0.02530	-0.03569	-0.05326	-0.06706					
256	0.00680	-0.00428	-0.01308	-0.02723	-0.04275	-0.06912	-0.09536						
128	0.00778	-0.00412	-0.02519	-0.05131	-0.08507	-0.13009							
64	0.01588	-0.02325	-0.05833	-0.10308	-0.15044								
32	0.01974	-0.03554	-0.10846	-0.18218									
16	0.02131	-0.09099	-0.19296										
8	0.01119	-0.15867											
4	-0.04313												

(d) $\mathcal{P}^{\mathcal{C}}$ for Scene: [**Sponza**] Algorithm: [**MLT**] Metric: [**NQM**] True GT: [**PT @ 32768 spp**].

M-MLT	2	4	8	16	32	64	128	256	512	1024	2048	4096	8192
GT (PT)													
16384	0.00029	0.00001	0.00022	0.00042	-0.00040	-0.00073	-0.00027	-0.00079	-0.00101	-0.00050	-0.00073	-0.00196	-0.00268
8192	0.00046	0.00005	-0.00030	-0.00023	-0.00113	-0.00188	-0.00264	-0.00334	-0.00506	-0.00502	-0.00506	-0.00588	
4096	-0.00051	-0.00058	-0.00092	-0.00105	-0.00360	-0.00462	-0.00643	-0.00862	-0.01116	-0.01236	-0.01410		
2048	0.00106	-0.00039	-0.00164	-0.00317	-0.00658	-0.00871	-0.01441	-0.01869	-0.02127	-0.02728			
1024	0.00159	-0.00040	-0.00282	-0.00738	-0.01144	-0.01963	-0.02658	-0.03716	-0.04716				
512	0.00116	-0.00284	-0.00656	-0.01460	-0.02535	-0.03509	-0.05356	-0.06890					
256	0.00376	0.00314	-0.01302	-0.02562	-0.04381	-0.06661	-0.09540						
128	0.00319	-0.00346	-0.02369	-0.05149	-0.08186	-0.12286							
64	0.00018	-0.01163	-0.05281	-0.10343	-0.15797								
32	0.00742	-0.02985	-0.09443	-0.17822									
16	-0.01450	-0.07777	-0.19602										
8	-0.04372	-0.13859											
4	-0.10301												

(e) $\mathcal{P}^{\mathcal{C}}$ for Scene: [**Sponza**] Algorithm: [**M-MLT**] Metric: [**NQM**] True GT: [**PT @ 32768 spp**].

ERPT	2	4	8	16	32	64	128	256	512	1024	2048	4096	8192
GT (PT)													
16384	-0.00157	-0.00128	-0.00044	-0.00026	-0.00131	-0.00044	-0.00146	-0.00066	-0.00063	-0.00011	-0.00085	-0.00190	-0.00113
8192	-0.00244	-0.00239	-0.00311	-0.00309	-0.00377	-0.00250	-0.00348	-0.00260	-0.00277	-0.00351	-0.00298	-0.00350	
4096	-0.00560	-0.00582	-0.00688	-0.00684	-0.00716	-0.00797	-0.00648	-0.00725	-0.00775	-0.00697	-0.00789		
2048	-0.01298	-0.01500	-0.01655	-0.01503	-0.01468	-0.01539	-0.01619	-0.01562	-0.01624	-0.01524			
1024	-0.02682	-0.03081	-0.02997	-0.03256	-0.03287	-0.03241	-0.03226	-0.03080	-0.03063				
512	-0.05316	-0.05502	-0.05720	-0.06057	-0.06062	-0.06250	-0.06173	-0.05904					
256	-0.09377	-0.10200	-0.10541	-0.10853	-0.11347	-0.10881	-0.10517						
128	-0.17374	-0.18519	-0.18864	-0.19642	-0.19303	-0.19204							
64	-0.28264	-0.29670	-0.30190	-0.30953	-0.30247								
32	-0.43570	-0.44712	-0.45108	-0.46383									
16	-0.62285	-0.64066	-0.64353										
8	-0.84385	-0.85895											
4	-1.05902												

(f) $\mathcal{P}^{\mathcal{C}}$ for Scene: [**Sponza**] Algorithm: [**ERPT**] Metric: [**NQM**] True GT: [**PT @ 32768 spp**].

M-ERPT	2	4	8	16	32	64	128	256	512	1024	2048	4096	8192
GT (PT)													
16384	-0.00058	-0.00111	-0.00048	-0.00113	-0.00103	-0.00104	-0.00101	-0.00019	-0.00042	-0.00076	-0.00140	-0.00039	-0.00045
8192	-0.00274	-0.00330	-0.00171	-0.00303	-0.00258	-0.00266	-0.00270	-0.00246	-0.00214	-0.00356	-0.00338	-0.00266	
4096	-0.00713	-0.00688	-0.00642	-0.00691	-0.00701	-0.00657	-0.00642	-0.00589	-0.00514	-0.00661	-0.00707		
2048	-0.01417	-0.01374	-0.01600	-0.01574	-0.01522	-0.01457	-0.01432	-0.01553	-0.01517	-0.01565			
1024	-0.02843	-0.02536	-0.03036	-0.02908	-0.03189	-0.02955	-0.03045	-0.03443	-0.02999				
512	-0.05242	-0.05420	-0.05675	-0.05578	-0.05699	-0.05914	-0.05801	-0.05940					
256	-0.09434	-0.10570	-0.10403	-0.10177	-0.10911	-0.10493	-0.10342						
128	-0.17477	-0.18588	-0.18529	-0.18067	-0.18837	-0.18919							
64	-0.28177	-0.28773	-0.29635	-0.29501	-0.29722								
32	-0.43203	-0.44657	-0.45121	-0.45022									
16	-0.62217	-0.63868	-0.64201										
8	-0.84383	-0.85660											
4	-1.05921												

(g) $\mathcal{P}^{\mathcal{C}}$ for Scene: [**Sponza**] Algorithm: [**M-ERPT**] Metric: [**NQM**] True GT: [**PT @ 32768 spp**].

Table 75

17 SSIM

17.1 Cornell Box

PT	2	4	8	16	32	64	128	256	512	1024	2048	4096	8192
**GT (PT)**													
16384	-0.00006	-0.00020	-0.00008	0.00001	-0.00011	-0.00021	-0.00019	-0.00016	-0.00023	-0.00026	-0.00028	-0.00026	-0.00029
8192	-0.00035	-0.00052	-0.00008	-0.00024	-0.00020	-0.00047	-0.00064	-0.00065	-0.00067	-0.00072	-0.00079	-0.00078	
4096	-0.00068	-0.00060	-0.00038	-0.00081	-0.00094	-0.00097	-0.00158	-0.00170	-0.00169	-0.00173	-0.00175		
2048	-0.00140	-0.00066	-0.00119	-0.00159	-0.00201	-0.00279	-0.00310	-0.00352	-0.00363	-0.00369			
1024	-0.00179	-0.00156	-0.00276	-0.00361	-0.00450	-0.00549	-0.00644	-0.00698	-0.00740				
512	-0.00285	-0.00400	-0.00480	-0.00677	-0.00967	-0.01104	-0.01298	-0.01370					
256	-0.00494	-0.00670	-0.00887	-0.01393	-0.01727	-0.02259	-0.02593						
128	-0.01061	-0.01528	-0.02090	-0.02783	-0.03487	-0.04364							
64	-0.02180	-0.02678	-0.03820	-0.05347	-0.06700								
32	-0.03730	-0.05348	-0.07462	-0.09814									
16	-0.07348	-0.10329	-0.13547										
8	-0.13598	-0.18733											
4	-0.22886												

(a) $\mathcal{P}^{\mathcal{C}}$ for Scene: [**Cornell Box**] Algorithm: [**PT**] Metric: [**SSIM**] True GT: [**PT @ 32768 spp**].

BDPT	2	4	8	16	32	64	128	256	512	1024	2048	4096	8192
**GT (PT)**													
16384	-0.00011	-0.00023	0.00000	-0.00018	-0.00019	-0.00025	-0.00025	-0.00024	-0.00026	-0.00025	-0.00028	-0.00029	-0.00031
8192	-0.00038	-0.00026	-0.00050	-0.00056	-0.00057	-0.00065	-0.00072	-0.00074	-0.00075	-0.00077	-0.00080	-0.00083	
4096	-0.00051	-0.00102	-0.00104	-0.00129	-0.00136	-0.00159	-0.00165	-0.00176	-0.00174	-0.00180	-0.00187		
2048	-0.00118	-0.00132	-0.00208	-0.00263	-0.00318	-0.00348	-0.00351	-0.00363	-0.00369	-0.00375			
1024	-0.00324	-0.00378	-0.00465	-0.00544	-0.00619	-0.00665	-0.00710	-0.00747	-0.00748				
512	-0.00504	-0.00770	-0.00951	-0.01126	-0.01245	-0.01371	-0.01440	-0.01473					
256	-0.00978	-0.01326	-0.01755	-0.02249	-0.02494	-0.02698	-0.02891						
128	-0.01995	-0.02826	-0.03618	-0.04208	-0.04943	-0.05262							
64	-0.03628	-0.05164	-0.06887	-0.07989	-0.09051								
32	-0.07269	-0.09939	-0.12349	-0.14332									
16	-0.13064	-0.17316	-0.21030										
8	-0.22227	-0.28910											
4	-0.35663												

(b) $\mathcal{P}^{\mathcal{C}}$ for Scene: [**Cornell Box**] Algorithm: [**BDPT**] Metric: [**SSIM**] True GT: [**PT @ 32768 spp**].

PSSMLT	2	4	8	16	32	64	128	256	512	1024	2048	4096	8192
**GT (PT)**													
16384	-0.00022	-0.00009	-0.00008	-0.00007	-0.00016	-0.00028	-0.00026	-0.00024	-0.00025	-0.00026	-0.00026	-0.00027	-0.00026
8192	-0.00020	-0.00025	-0.00021	-0.00037	-0.00052	-0.00067	-0.00065	-0.00073	-0.00071	-0.00074	-0.00076	-0.00077	
4096	-0.00064	-0.00066	-0.00110	-0.00120	-0.00142	-0.00162	-0.00171	-0.00172	-0.00177	-0.00178	-0.00181		
2048	-0.00140	-0.00157	-0.00215	-0.00251	-0.00311	-0.00335	-0.00349	-0.00360	-0.00368	-0.00375			
1024	-0.00246	-0.00344	-0.00452	-0.00533	-0.00597	-0.00667	-0.00705	-0.00736	-0.00748				
512	-0.00485	-0.00657	-0.00847	-0.01067	-0.01251	-0.01370	-0.01438	-0.01492					
256	-0.00999	-0.01445	-0.01869	-0.02196	-0.02495	-0.02741	-0.02856						
128	-0.02101	-0.02811	-0.03567	-0.04271	-0.04825	-0.05225							
64	-0.04002	-0.05226	-0.06665	-0.07984	-0.08990								
32	-0.07329	-0.09869	-0.12039	-0.14145									
16	-0.13129	-0.17241	-0.20987										
8	-0.23253	-0.29005											
4	-0.37796												

(c) $\mathcal{P}^{\mathcal{C}}$ for Scene: [**Cornell Box**] Algorithm: [**PSSMLT**] Metric: [**SSIM**] True GT: [**PT @ 32768 spp**].

MLT	2	4	8	16	32	64	128	256	512	1024	2048	4096	8192
**GT (PT)**													
16384	0.00006	-0.00016	-0.00003	-0.00004	-0.00011	-0.00020	-0.00015	-0.00023	-0.00025	-0.00024	-0.00023	-0.00026	-0.00026
8192	-0.00010	-0.00025	-0.00016	-0.00026	-0.00034	-0.00058	-0.00063	-0.00071	-0.00070	-0.00073	-0.00074	-0.00075	
4096	0.00001	-0.00041	-0.00041	-0.00081	-0.00138	-0.00162	-0.00164	-0.00166	-0.00176	-0.00172	-0.00176		
2048	-0.00004	-0.00016	-0.00035	-0.00078	-0.00181	-0.00246	-0.00297	-0.00339	-0.00349	-0.00353			
1024	-0.00131	-0.00168	-0.00207	-0.00301	-0.00447	-0.00558	-0.00631	-0.00693	-0.00726				
512	-0.00403	-0.00354	-0.00522	-0.00723	-0.00942	-0.01148	-0.01295	-0.01400					
256	-0.00511	-0.00797	-0.01055	-0.01431	-0.01876	-0.02245	-0.02524						
128	-0.00914	-0.01165	-0.01655	-0.02528	-0.03381	-0.04162							
64	-0.01572	-0.02523	-0.03783	-0.05135	-0.06516								
32	-0.03534	-0.05036	-0.06773	-0.09258									
16	-0.06606	-0.08981	-0.12591										
8	-0.11356	-0.16090											
4	-0.20799												

(d) $\mathcal{P}^{\mathcal{C}}$ for Scene: [**Cornell Box**] Algorithm: [**MLT**] Metric: [**SSIM**] True GT: [**PT @ 32768 spp**].

M-MLT	2	4	8	16	32	64	128	256	512	1024	2048	4096	8192
**GT (PT)**													
16384	-0.00024	0.00018	0.00011	0.00008	0.00010	-0.00008	-0.00018	-0.00022	-0.00022	-0.00027	-0.00024	-0.00024	-0.00025
8192	-0.00024	-0.00007	-0.00019	-0.00033	-0.00043	-0.00062	-0.00059	-0.00069	-0.00077	-0.00080	-0.00079	-0.00075	
4096	-0.00054	-0.00031	-0.00067	-0.00086	-0.00103	-0.00139	-0.00149	-0.00166	-0.00169	-0.00176	-0.00177		
2048	-0.00078	-0.00083	-0.00106	-0.00174	-0.00219	-0.00265	-0.00302	-0.00335	-0.00350	-0.00362			
1024	-0.00110	-0.00101	-0.00201	-0.00343	-0.00469	-0.00584	-0.00663	-0.00717	-0.00732				
512	-0.00173	-0.00304	-0.00503	-0.00713	-0.00873	-0.01106	-0.01255	-0.01384					
256	-0.00558	-0.00836	-0.01087	-0.01477	-0.01851	-0.02278	-0.02592						
128	-0.01066	-0.01231	-0.01940	-0.02770	-0.03597	-0.04354							
64	-0.01309	-0.02435	-0.03655	-0.05102	-0.06507								
32	-0.03640	-0.05025	-0.07196	-0.09729									
16	-0.06790	-0.09905	-0.13249										
8	-0.12840	-0.16652											
4	-0.21320												

(e) $\mathcal{P}^{\mathcal{C}}$ for Scene: [**Cornell Box**] Algorithm: [**M-MLT**] Metric: [**SSIM**] True GT: [**PT @ 32768 spp**].

ERPT	2	4	8	16	32	64	128	256	512	1024	2048	4096	8192
**GT (PT)**													
16384	-0.00020	-0.00029	-0.00018	-0.00029	-0.00019	-0.00025	-0.00026	-0.00023	-0.00033	-0.00024	-0.00016	-0.00023	-0.00032
8192	-0.00069	-0.00068	-0.00069	-0.00062	-0.00066	-0.00066	-0.00063	-0.00072	-0.00066	-0.00066	-0.00062	-0.00069	
4096	-0.00149	-0.00160	-0.00140	-0.00142	-0.00148	-0.00164	-0.00150	-0.00151	-0.00153	-0.00149	-0.00157		
2048	-0.00322	-0.00326	-0.00324	-0.00313	-0.00313	-0.00329	-0.00312	-0.00302	-0.00314	-0.00325			
1024	-0.00657	-0.00636	-0.00627	-0.00648	-0.00639	-0.00672	-0.00651	-0.00643	-0.00663				
512	-0.01305	-0.01269	-0.01285	-0.01300	-0.01302	-0.01353	-0.01299	-0.01324					
256	-0.02582	-0.02637	-0.02570	-0.02573	-0.02593	-0.02615	-0.02544						
128	-0.05005	-0.05005	-0.05031	-0.04969	-0.04936	-0.04996							
64	-0.09232	-0.09194	-0.09245	-0.09125	-0.09052								
32	-0.16266	-0.16232	-0.16317	-0.16245									
16	-0.26900	-0.27250	-0.27275										
8	-0.42485	-0.42660											
4	-0.62254												

(f) $\mathcal{P}^{\mathcal{C}}$ for Scene: [**Cornell Box**] Algorithm: [**ERPT**] Metric: [**SSIM**] True GT: [**PT @ 32768 spp**].

M-ERPT	2	4	8	16	32	64	128	256	512	1024	2048	4096	8192
**GT (PT)**													
16384	-0.00015	-0.00022	-0.00027	-0.00028	-0.00023	-0.00033	-0.00028	-0.00027	-0.00028	-0.00016	-0.00017	-0.00025	-0.00020
8192	-0.00071	-0.00061	-0.00061	-0.00083	-0.00066	-0.00059	-0.00059	-0.00067	-0.00057	-0.00062	-0.00061	-0.00069	
4096	-0.00145	-0.00140	-0.00149	-0.00156	-0.00147	-0.00152	-0.00153	-0.00142	-0.00152	-0.00152	-0.00145		
2048	-0.00315	-0.00313	-0.00318	-0.00314	-0.00324	-0.00329	-0.00295	-0.00291	-0.00308	-0.00310			
1024	-0.00626	-0.00623	-0.00671	-0.00656	-0.00625	-0.00639	-0.00623	-0.00685	-0.00631				
512	-0.01259	-0.01283	-0.01302	-0.01312	-0.01272	-0.01261	-0.01283	-0.01251					
256	-0.02555	-0.02538	-0.02607	-0.02530	-0.02560	-0.02575	-0.02631						
128	-0.04885	-0.04874	-0.04965	-0.04962	-0.04914	-0.04966							
64	-0.09277	-0.09216	-0.09182	-0.09060	-0.09158								
32	-0.16137	-0.16122	-0.16170	-0.16096									
16	-0.26916	-0.26881	-0.26961										
8	-0.42268	-0.42332											
4	-0.62071												

(g) $\mathcal{P}^{\mathcal{C}}$ for Scene: [**Cornell Box**] Algorithm: [**M-ERPT**] Metric: [**SSIM**] True GT: [**PT @ 32768 spp**].

Table 76

17.2 Torus

PT	2	4	8	16	32	64	128	256	512	1024	2048	4096	8192	16384	32768	65536	131072
GT (BDPT)																	
262144	-0.00052	-0.00056	-0.00128	-0.00220	-0.00172	-0.00156	-0.00137	-0.00113	-0.00072	-0.00079	-0.00078	-0.00099	-0.00149	-0.00172	-0.00185	-0.00249	-0.00272
131072	-0.00253	-0.00371	-0.00461	-0.00545	-0.00501	-0.00489	-0.00372	-0.00283	-0.00248	-0.00221	-0.00207	-0.00352	-0.00432	-0.00560	-0.00684	-0.00782	
65536	-0.00475	-0.00869	-0.01065	-0.01182	-0.01139	-0.01042	-0.00860	-0.00661	-0.00563	-0.00518	-0.00577	-0.00707	-0.00974	-0.01226	-0.01392		
32768	-0.01025	-0.01718	-0.02128	-0.02425	-0.02328	-0.02138	-0.01670	-0.01452	-0.01183	-0.01103	-0.01133	-0.01437	-0.01966	-0.02489			
16384	-0.01972	-0.03051	-0.03883	-0.04313	-0.03996	-0.03879	-0.03095	-0.02677	-0.02103	-0.02037	-0.02117	-0.02807	-0.03585				
8192	-0.03954	-0.05073	-0.06236	-0.06908	-0.06667	-0.06199	-0.05190	-0.04552	-0.03600	-0.03615	-0.03710	-0.04717					
4096	-0.06243	-0.07805	-0.09086	-0.09866	-0.09353	-0.08820	-0.07495	-0.06423	-0.05384	-0.05271	-0.05801						
2048	-0.08357	-0.10504	-0.12223	-0.12670	-0.11983	-0.11192	-0.09622	-0.08228	-0.07236	-0.07123							
1024	-0.09405	-0.12119	-0.13940	-0.14550	-0.13552	-0.12741	-0.10869	-0.09524	-0.08638								
512	-0.09622	-0.12165	-0.13933	-0.14359	-0.13417	-0.12723	-0.10865	-0.09694									
256	-0.09143	-0.10882	-0.12468	-0.12415	-0.11610	-0.11146	-0.09809										
128	-0.07133	-0.08854	-0.10116	-0.10442	-0.10077	-0.09802											
64	-0.05897	-0.07776	-0.09183	-0.09448	-0.09475												
32	-0.05784	-0.08083	-0.09777	-0.11169													
16	-0.07159	-0.11112	-0.14426														
8	-0.13584	-0.19643															
4	-0.25712																

(a) $\mathcal{P}^{\mathcal{C}}$ for Scene: [**Torus**] Algorithm: [**PT**] Metric: [**SSIM**] True GT: [**BDPT @ 524288 spp**].

BDPT	2	4	8	16	32	64	128	256	512	1024	2048	4096	8192	16384	32768	65536	131072
GT (BDPT)																	
262144	-0.00021	-0.00034	-0.00132	-0.00134	-0.00202	-0.00121	-0.00140	-0.00087	-0.00067	-0.00076	-0.00100	-0.00043	-0.00129	-0.00153	-0.00216	-0.00265	-0.00291
131072	-0.00212	-0.00214	-0.00396	-0.00426	-0.00465	-0.00418	-0.00421	-0.00293	-0.00264	-0.00230	-0.00261	-0.00306	-0.00418	-0.00534	-0.00635	-0.00797	
65536	-0.00430	-0.00616	-0.00810	-0.01075	-0.01128	-0.00958	-0.00899	-0.00738	-0.00550	-0.00538	-0.00613	-0.00632	-0.00908	-0.01167	-0.01407		
32768	-0.00944	-0.01468	-0.01919	-0.02223	-0.02159	-0.02038	-0.01790	-0.01538	-0.01057	-0.01151	-0.01257	-0.01444	-0.01815	-0.02300			
16384	-0.01506	-0.02615	-0.03371	-0.03868	-0.03999	-0.03764	-0.03201	-0.02567	-0.02224	-0.02158	-0.02324	-0.02679	-0.03324				
8192	-0.03008	-0.04366	-0.05849	-0.06372	-0.06318	-0.06016	-0.05328	-0.04259	-0.03865	-0.03471	-0.03637	-0.04473					
4096	-0.04920	-0.06960	-0.08362	-0.09247	-0.09163	-0.08597	-0.07426	-0.06460	-0.05413	-0.05155	-0.05699						
2048	-0.07112	-0.09450	-0.10886	-0.11819	-0.11690	-0.10846	-0.09537	-0.08046	-0.07142	-0.06908							
1024	-0.08739	-0.10989	-0.13111	-0.13700	-0.13465	-0.12368	-0.11008	-0.09213	-0.08620								
512	-0.08742	-0.11222	-0.12871	-0.13602	-0.13289	-0.12211	-0.10805	-0.09171									
256	-0.07552	-0.10282	-0.11532	-0.11914	-0.11579	-0.10967	-0.09408										
128	-0.06558	-0.08191	-0.09484	-0.09976	-0.09663	-0.09181											
64	-0.05281	-0.07122	-0.08450	-0.09308	-0.09307												
32	-0.05005	-0.07091	-0.09534	-0.10573													
16	-0.06768	-0.10877	-0.13813														
8	-0.12379	-0.18501															
4	-0.24591																

(b) $\mathcal{P}^{\mathcal{C}}$ for Scene: [**Torus**] Algorithm: [**BDPT**] Metric: [**SSIM**] True GT: [**BDPT @ 524288 spp**].

PSSMLT	2	4	8	16	32	64	128	256	512	1024	2048	4096	8192	16384
GT (BDPT)														
262144	-0.00060	-0.00068	-0.00067	-0.00089	-0.00128	-0.00110	-0.00130	-0.00102	-0.00162	-0.00183	-0.00194	-0.00211	-0.00244	-0.00259
131072	-0.00158	-0.00191	-0.00286	-0.00334	-0.00358	-0.00351	-0.00379	-0.00410	-0.00422	-0.00516	-0.00598	-0.00663	-0.00769	-0.00811
65536	-0.00432	-0.00527	-0.00683	-0.00735	-0.00835	-0.00761	-0.00732	-0.00852	-0.00970	-0.01148	-0.01328	-0.01533	-0.01692	-0.01838
32768	-0.00755	-0.01054	-0.01380	-0.01494	-0.01605	-0.01526	-0.01588	-0.01740	-0.02063	-0.02412	-0.02692	-0.02984	-0.03322	-0.03565
16384	-0.01538	-0.02037	-0.02742	-0.03036	-0.03067	-0.02918	-0.03113	-0.03275	-0.03719	-0.04181	-0.04715	-0.05297	-0.05804	
8192	-0.02689	-0.03558	-0.04377	-0.04820	-0.04832	-0.04924	-0.05198	-0.05442	-0.06103	-0.06758	-0.07776	-0.08580		
4096	-0.04005	-0.05497	-0.06486	-0.07049	-0.07087	-0.07071	-0.07531	-0.08048	-0.08964	-0.10016	-0.11212			
2048	-0.06013	-0.07928	-0.09174	-0.09829	-0.09698	-0.09710	-0.09991	-0.10798	-0.11888	-0.13239				
1024	-0.07419	-0.09365	-0.10792	-0.11177	-0.11171	-0.11247	-0.12067	-0.13141	-0.14467					
512	-0.08092	-0.09972	-0.11101	-0.11575	-0.11580	-0.11923	-0.12498	-0.13453						
256	-0.07642	-0.09267	-0.10383	-0.10679	-0.10747	-0.11117	-0.11793							
128	-0.06924	-0.07991	-0.08976	-0.09322	-0.09437	-0.09832								
64	-0.07475	-0.08395	-0.09145	-0.09392	-0.09892									
32	-0.09840	-0.10543	-0.11382	-0.11739										
16	-0.15879	-0.16381	-0.17509											
8	-0.27504	-0.28203												
4	-0.47939													

(c) $\mathcal{P}^{\mathcal{C}}$ for Scene: [**Torus**] Algorithm: [**PSSMLT**] Metric: [**SSIM**] True GT: [**BDPT @ 524288 spp**].

MLT	2	4	8	16	32	64	128	256	512	1024	2048	4096	8192	16384
GT (BDPT)														
262144	-0.00084	-0.00063	-0.00028	-0.00127	-0.00149	-0.00173	-0.00213	-0.00210	-0.00222	-0.00245	-0.00260	-0.00248	-0.00269	-0.00261
131072	-0.00250	-0.00199	-0.00268	-0.00360	-0.00446	-0.00601	-0.00607	-0.00664	-0.00720	-0.00759	-0.00799	-0.00799	-0.00814	-0.00850
65536	-0.00546	-0.00546	-0.00540	-0.00770	-0.01016	-0.01204	-0.01366	-0.01491	-0.01606	-0.01692	-0.01719	-0.01771	-0.01785	-0.01851
32768	-0.01236	-0.01107	-0.01140	-0.01536	-0.02095	-0.02361	-0.02716	-0.03023	-0.03147	-0.03262	-0.03385	-0.03433	-0.03536	-0.03606
16384	-0.02122	-0.02090	-0.02030	-0.02888	-0.03615	-0.04192	-0.04800	-0.05219	-0.05528	-0.05816	-0.05944	-0.06076	-0.06166	
8192	-0.03657	-0.03443	-0.03699	-0.04811	-0.06063	-0.06914	-0.07852	-0.08377	-0.08925	-0.09190	-0.09457	-0.09564		
4096	-0.05559	-0.05355	-0.05684	-0.07470	-0.08989	-0.10181	-0.11410	-0.12195	-0.12691	-0.13026	-0.13333			
2048	-0.07605	-0.07658	-0.08070	-0.10124	-0.12030	-0.13404	-0.14731	-0.15439	-0.16189	-0.16588				
1024	-0.09511	-0.09325	-0.09993	-0.12358	-0.14472	-0.15793	-0.17232	-0.17897	-0.18656					
512	-0.10100	-0.10226	-0.10629	-0.13223	-0.15110	-0.16358	-0.17846	-0.18614						
256	-0.09035	-0.09236	-0.09973	-0.12379	-0.14154	-0.15440	-0.16737							
128	-0.08354	-0.08649	-0.09362	-0.11504	-0.12794	-0.13887								
64	-0.08369	-0.08792	-0.09273	-0.11535	-0.12498									
32	-0.10616	-0.11333	-0.11514	-0.13282										
16	-0.16969	-0.17669	-0.17742											
8	-0.29962	-0.30502												
4	-0.52782													

(d) $\mathcal{P}^{\mathcal{C}}$ for Scene: [**Torus**] Algorithm: [**MLT**] Metric: [**SSIM**] True GT: [**BDPT @ 524288 spp**].

M-MLT	2	4	8	16	32	64	128	256	512	1024	2048	4096	8192	16384
GT (BDPT)														
262144	-0.00051	-0.00092	-0.00092	-0.00083	-0.00115	-0.00203	-0.00227	-0.00259	-0.00266	-0.00272	-0.00267	-0.00307	-0.00280	-0.00298
131072	-0.00295	-0.00168	-0.00223	-0.00278	-0.00305	-0.00483	-0.00572	-0.00651	-0.00800	-0.00799	-0.00812	-0.00873	-0.00873	-0.00896
65536	-0.00572	-0.00409	-0.00573	-0.00795	-0.00851	-0.01221	-0.01385	-0.01612	-0.01729	-0.01766	-0.01790	-0.01868	-0.01884	-0.01960
32768	-0.01216	-0.00925	-0.01148	-0.01501	-0.01829	-0.02456	-0.02700	-0.03092	-0.03392	-0.03430	-0.03607	-0.03677	-0.03746	-0.03818
16384	-0.02223	-0.01779	-0.02133	-0.02666	-0.03224	-0.04309	-0.04802	-0.05399	-0.05997	-0.06157	-0.06265	-0.06397	-0.06496	
8192	-0.03670	-0.02901	-0.03620	-0.04687	-0.05673	-0.07017	-0.07794	-0.08697	-0.09464	-0.09530	-0.09955	-0.10114		
4096	-0.05459	-0.04661	-0.05821	-0.06809	-0.08284	-0.10489	-0.11338	-0.12428	-0.13303	-0.13457	-0.13884			
2048	-0.07302	-0.06423	-0.07813	-0.09819	-0.11453	-0.13630	-0.14521	-0.15858	-0.16925	-0.17077				
1024	-0.09032	-0.08627	-0.10189	-0.12015	-0.13697	-0.16228	-0.17204	-0.18377	-0.19442					
512	-0.09601	-0.09001	-0.11372	-0.12914	-0.13968	-0.16713	-0.17932	-0.18941						
256	-0.08693	-0.08779	-0.10736	-0.12254	-0.13306	-0.15894	-0.16916							
128	-0.07972	-0.07903	-0.10249	-0.11212	-0.12245	-0.14692								
64	-0.08171	-0.08257	-0.10430	-0.11361	-0.12084									
32	-0.10400	-0.10423	-0.12610	-0.13402										
16	-0.17275	-0.16971	-0.18576											
8	-0.29961	-0.28388												
4	-0.52751													

(e) $\mathcal{P}^{\mathcal{C}}$ for Scene: [**Torus**] Algorithm: [**M-MLT**] Metric: [**SSIM**] True GT: [**BDPT @ 524288 spp**].

ERPT	2	4	8	16	32	64	128	256	512	1024	2048	4096	8192	16384	32768	65536	131072
GT (BDPT)																	
262144	-0.00212	-0.00224	-0.00249	-0.00238	-0.00246	-0.00237	-0.00202	-0.00212	-0.00201	-0.00221	-0.00217	-0.00228	-0.00244	-0.00244	-0.00219	-0.00209	-0.00210
131072	-0.00671	-0.00678	-0.00717	-0.00726	-0.00683	-0.00651	-0.00634	-0.00618	-0.00630	-0.00669	-0.00633	-0.00714	-0.00697	-0.00699	-0.00722	-0.00705	
65536	-0.01467	-0.01501	-0.01516	-0.01454	-0.01434	-0.01467	-0.01471	-0.01377	-0.01400	-0.01418	-0.01450	-0.01532	-0.01546	-0.01576	-0.01550		
32768	-0.03055	-0.02949	-0.02942	-0.03058	-0.02944	-0.02889	-0.02917	-0.02794	-0.02757	-0.02884	-0.02966	-0.03133	-0.03113	-0.02976			
16384	-0.05331	-0.05243	-0.05313	-0.05350	-0.05228	-0.05156	-0.05224	-0.05095	-0.05082	-0.05110	-0.05270	-0.05464	-0.05500				
8192	-0.08446	-0.08464	-0.08479	-0.08548	-0.08328	-0.08254	-0.08411	-0.08318	-0.08221	-0.08318	-0.08595	-0.08754					
4096	-0.12015	-0.12046	-0.12145	-0.12324	-0.12118	-0.12091	-0.12005	-0.11829	-0.11823	-0.12079	-0.12323						
2048	-0.15214	-0.15395	-0.15540	-0.15610	-0.15517	-0.15218	-0.15414	-0.15473	-0.15264	-0.15584							
1024	-0.17483	-0.17957	-0.17906	-0.17984	-0.18040	-0.17838	-0.18017	-0.18103	-0.18015								
512	-0.17748	-0.18297	-0.18354	-0.18609	-0.18562	-0.18391	-0.18625	-0.18673									
256	-0.16421	-0.16963	-0.17141	-0.17166	-0.17474	-0.17099	-0.17651										
128	-0.14242	-0.14906	-0.15073	-0.15544	-0.15816	-0.15614											
64	-0.13585	-0.14180	-0.14633	-0.14932	-0.15141												
32	-0.15286	-0.16198	-0.16506	-0.16834													
16	-0.21474	-0.22529	-0.23071														
8	-0.34333	-0.35328															
4	-0.57729																

(f) $\mathcal{P}^{\mathcal{C}}$ for Scene: [**Torus**] Algorithm: [**ERPT**] Metric: [**SSIM**] True GT: [**BDPT @ 524288 spp**].

M-ERPT	2	4	8	16	32	64	128	256	512	1024	2048	4096	8192	16384	32768	65536	131072
GT (BDPT)																	
262144	-0.00177	-0.00248	-0.00199	-0.00215	-0.00227	-0.00233	-0.00238	-0.00236	-0.00215	-0.00226	-0.00289	-0.00238	-0.00258	-0.00259	-0.00239	-0.00209	-0.00240
131072	-0.00591	-0.00660	-0.00621	-0.00604	-0.00690	-0.00675	-0.00626	-0.00720	-0.00706	-0.00712	-0.00692	-0.00706	-0.00692	-0.00737	-0.00723	-0.00696	
65536	-0.01271	-0.01342	-0.01385	-0.01427	-0.01468	-0.01438	-0.01471	-0.01500	-0.01525	-0.01495	-0.01536	-0.01531	-0.01523	-0.01574	-0.01575		
32768	-0.02577	-0.02667	-0.02691	-0.02801	-0.02857	-0.02933	-0.02966	-0.03059	-0.02978	-0.03110	-0.03111	-0.03095	-0.03091	-0.03047			
16384	-0.04603	-0.04860	-0.04966	-0.05026	-0.05038	-0.05340	-0.05260	-0.05371	-0.05279	-0.05475	-0.05497	-0.05415	-0.05503				
8192	-0.07299	-0.07678	-0.07941	-0.07995	-0.08036	-0.08391	-0.08609	-0.08587	-0.08771	-0.08741	-0.08811	-0.08688					
4096	-0.10758	-0.11032	-0.11468	-0.11660	-0.11807	-0.12040	-0.12074	-0.12367	-0.12342	-0.12466	-0.12526						
2048	-0.13824	-0.14321	-0.14696	-0.14947	-0.15150	-0.15459	-0.15718	-0.15686	-0.15807	-0.16048							
1024	-0.16100	-0.16478	-0.16994	-0.17472	-0.17703	-0.18078	-0.18378	-0.18409	-0.18556								
512	-0.16536	-0.16894	-0.17423	-0.18049	-0.18067	-0.18683	-0.18966	-0.19180									
256	-0.15072	-0.15630	-0.16103	-0.16758	-0.16820	-0.17536	-0.17960										
128	-0.13225	-0.13695	-0.14385	-0.15211	-0.15305	-0.15846											
64	-0.12633	-0.13016	-0.13674	-0.14773	-0.14440												
32	-0.14430	-0.15027	-0.15686	-0.16567													
16	-0.20524	-0.21231	-0.21793														
8	-0.33071	-0.33795															
4	-0.56011																

(g) $\mathcal{P}^{\mathcal{C}}$ for Scene: [**Torus**] Algorithm: [**M-ERPT**] Metric: [**SSIM**] True GT: [**BDPT @ 524288 spp**].

Table 77

17.3 Veach Bidir

PT	2	4	8	16	32	64	128	256	512	1024	2048	4096	8192	16384	32768	65536	131072
GT (BDPT)																	
262144	0.00011	0.00011	0.00008	0.00006	0.00003	-0.00008	0.00008	-0.00001	0.00005	0.00001	-0.00004	0.00004	-0.00007	-0.00005	-0.00005	-0.00004	-0.00007
131072	0.00024	-0.00008	0.00043	0.00012	0.00003	0.00006	0.00019	0.00011	-0.00001	-0.00010	-0.00006	-0.00004	-0.00009	-0.00005	-0.00013	-0.00011	
65536	-0.00003	-0.00035	0.00023	0.00007	0.00013	0.00007	0.00024	-0.00008	-0.00017	-0.00015	-0.00009	0.00005	-0.00016	-0.00028	-0.00023		
32768	0.00069	-0.00071	0.00024	0.00048	-0.00028	-0.00000	0.00031	0.00006	-0.00025	-0.00021	-0.00038	-0.00026	-0.00051	-0.00057			
16384	0.00045	-0.00005	0.00083	0.00057	0.00022	-0.00022	0.00003	-0.00028	-0.00039	-0.00044	-0.00047	-0.00031	-0.00062				
8192	0.00051	-0.00042	0.00038	0.00009	-0.00038	0.00030	-0.00015	-0.00062	-0.00046	-0.00080	-0.00115	-0.00084					
4096	-0.00050	0.00050	0.00064	0.00051	-0.00029	-0.00045	-0.00055	-0.00098	-0.00079	-0.00112	-0.00148						
2048	0.00117	-0.00037	-0.00014	0.00059	-0.00066	-0.00045	-0.00144	-0.00230	-0.00225	-0.00251							
1024	-0.00076	-0.00217	-0.00098	0.00009	-0.00143	-0.00166	-0.00243	-0.00333	-0.00350								
512	-0.00049	-0.00221	-0.00049	-0.00369	-0.00354	-0.00288	-0.00518	-0.00721									
256	0.00031	-0.00155	-0.00138	-0.00329	-0.00496	-0.00732	-0.01191										
128	-0.00035	-0.00711	-0.00149	-0.00742	-0.01204	-0.01463											
64	-0.00504	-0.00411	-0.00509	-0.01303	-0.01847												
32	-0.00428	-0.01301	-0.01676	-0.03140													
16	-0.01318	-0.02888	-0.04166														
8	-0.04264	-0.07687															
4	-0.09033																

(a) $\mathcal{P}^{\mathcal{C}}$ for Scene: [**Veach Bidir**] Algorithm: [**PT**] Metric: [**SSIM**] True GT: [**BDPT @ 524288 spp**].

BDPT	2	4	8	16	32	64	128	256	512	1024	2048	4096	8192	16384	32768	65536	131072
GT (BDPT)																	
262144	0.00013	0.00004	0.00002	0.00001	-0.00002	-0.00004	-0.00002	-0.00001	-0.00005	-0.00002	-0.00005	-0.00011	-0.00003	-0.00004	-0.00001	-0.00002	-0.00003
131072	0.00014	0.00023	0.00007	-0.00009	-0.00002	-0.00005	-0.00008	-0.00009	-0.00008	-0.00012	-0.00015	-0.00013	-0.00011	-0.00011	-0.00006	-0.00010	
65536	0.00037	0.00003	0.00004	-0.00012	-0.00013	-0.00017	-0.00020	-0.00019	-0.00020	-0.00027	-0.00022	-0.00024	-0.00020	-0.00028	-0.00025		
32768	0.00007	-0.00012	-0.00009	-0.00032	-0.00026	-0.00053	-0.00052	-0.00050	-0.00050	-0.00058	-0.00055	-0.00069	-0.00068	-0.00067			
16384	0.00027	0.00010	0.00024	-0.00030	-0.00043	-0.00040	-0.00045	-0.00058	-0.00067	-0.00070	-0.00077	-0.00088	-0.00084				
8192	0.00002	0.00011	0.00018	-0.00054	-0.00081	-0.00091	-0.00102	-0.00129	-0.00135	-0.00141	-0.00145	-0.00152					
4096	-0.00003	-0.00050	-0.00083	-0.00104	-0.00156	-0.00188	-0.00197	-0.00204	-0.00222	-0.00220	-0.00234						
2048	0.00024	-0.00047	-0.00167	-0.00229	-0.00247	-0.00313	-0.00349	-0.00368	-0.00355	-0.00408							
1024	-0.00129	-0.00202	-0.00293	-0.00380	-0.00480	-0.00582	-0.00630	-0.00700	-0.00718								
512	-0.00231	-0.00346	-0.00517	-0.00819	-0.00980	-0.01199	-0.01339	-0.01402									
256	-0.00437	-0.00618	-0.01204	-0.01691	-0.02028	-0.02348	-0.02593										
128	-0.00822	-0.01552	-0.02464	-0.03304	-0.03952	-0.04613											
64	-0.01792	-0.03243	-0.04729	-0.06439	-0.07706												
32	-0.03632	-0.06661	-0.09020	-0.11891													
16	-0.07085	-0.11097	-0.16161														
8	-0.12636	-0.20842															
4	-0.22868																

(b) $\mathcal{P}^{\mathcal{C}}$ for Scene: [**Veach Bidir**] Algorithm: [**BDPT**] Metric: [**SSIM**] True GT: [**BDPT @ 524288 spp**].

PSSMLT	2	4	8	16	32	64	128	256	512	1024	2048	4096	8192	16384
GT (BDPT)														
262144	0.00007	0.00008	-0.00001	-0.00005	-0.00002	-0.00006	-0.00006	-0.00005	-0.00005	-0.00003	-0.00002	-0.00002	-0.00002	-0.00002
131072	0.00026	0.00008	0.00006	-0.00003	-0.00001	-0.00007	-0.00009	-0.00008	-0.00010	-0.00009	-0.00009	-0.00009	-0.00012	-0.00011
65536	0.00011	0.00005	-0.00016	-0.00015	-0.00016	-0.00021	-0.00026	-0.00030	-0.00024	-0.00024	-0.00025	-0.00024	-0.00024	-0.00026
32768	0.00006	-0.00013	-0.00034	-0.00040	-0.00056	-0.00057	-0.00061	-0.00059	-0.00060	-0.00049	-0.00048	-0.00055	-0.00062	-0.00065
16384	-0.00002	-0.00008	-0.00027	-0.00029	-0.00042	-0.00049	-0.00063	-0.00067	-0.00080	-0.00083	-0.00086	-0.00078	-0.00086	
8192	0.00005	-0.00034	-0.00021	-0.00048	-0.00084	-0.00116	-0.00120	-0.00125	-0.00136	-0.00141	-0.00142	-0.00143		
4096	0.00038	-0.00047	-0.00073	-0.00109	-0.00148	-0.00173	-0.00202	-0.00206	-0.00217	-0.00227	-0.00242			
2048	-0.00033	-0.00062	-0.00136	-0.00213	-0.00261	-0.00330	-0.00362	-0.00384	-0.00412	-0.00417				
1024	-0.00078	-0.00196	-0.00244	-0.00396	-0.00533	-0.00602	-0.00673	-0.00690	-0.00733					
512	-0.00250	-0.00343	-0.00519	-0.00745	-0.01020	-0.01210	-0.01335	-0.01427						
256	-0.00528	-0.00791	-0.01224	-0.01725	-0.02156	-0.02475	-0.02670							
128	-0.01157	-0.01634	-0.02411	-0.03327	-0.04175	-0.04723								
64	-0.02113	-0.03113	-0.04603	-0.06287	-0.07782									
32	-0.04836	-0.07010	-0.09452	-0.12339										
16	-0.08636	-0.12526	-0.16963											
8	-0.15966	-0.22086												
4	-0.29425													

(c) $\mathcal{P}^{\mathcal{C}}$ for Scene: [**Veach Bidir**] Algorithm: [**PSSMLT**] Metric: [**SSIM**] True GT: [**BDPT @ 524288 spp**].

MLT	2	4	8	16	32	64	128	256	512	1024	2048	4096	8192	16384
GT (BDPT)														
262144	-0.00010	-0.00006	-0.00010	-0.00002	0.00008	0.00013	0.00011	0.00005	-0.00001	0.00000	-0.00000	-0.00001	-0.00001	-0.00000
131072	0.00017	0.00018	0.00019	0.00014	0.00017	0.00018	0.00008	0.00001	-0.00002	-0.00005	-0.00001	-0.00004	-0.00006	-0.00008
65536	0.00023	0.00035	0.00029	0.00031	0.00022	0.00009	-0.00011	-0.00013	-0.00022	-0.00019	-0.00015	-0.00019	-0.00019	-0.00021
32768	-0.00033	-0.00015	-0.00005	-0.00004	-0.00005	0.00002	-0.00018	-0.00025	-0.00033	-0.00039	-0.00041	-0.00046	-0.00049	-0.00055
16384	0.00019	0.00003	0.00013	0.00018	0.00004	-0.00012	-0.00027	-0.00048	-0.00048	-0.00052	-0.00062	-0.00065	-0.00067	
8192	-0.00011	-0.00037	0.00000	-0.00019	-0.00054	-0.00093	-0.00084	-0.00091	-0.00100	-0.00106	-0.00108	-0.00114		
4096	0.00074	0.00057	0.00059	0.00018	-0.00019	-0.00100	-0.00151	-0.00169	-0.00194	-0.00207	-0.00211			
2048	0.00039	0.00018	0.00005	-0.00080	-0.00163	-0.00200	-0.00277	-0.00316	-0.00345	-0.00359				
1024	-0.00113	0.00005	0.00014	-0.00122	-0.00270	-0.00359	-0.00477	-0.00540	-0.00612					
512	0.00056	-0.00048	-0.00042	-0.00271	-0.00511	-0.00803	-0.01061	-0.01235						
256	-0.00403	-0.00471	-0.00359	-0.00521	-0.00977	-0.01551	-0.01972							
128	-0.00471	-0.00787	-0.01064	-0.01572	-0.02380	-0.03164								
64	-0.01254	-0.01694	-0.02396	-0.03552	-0.04731									
32	-0.02644	-0.03050	-0.04009	-0.05926										
16	-0.05313	-0.06360	-0.08545											
8	-0.10352	-0.11539												
4	-0.16654													

(d) $\mathcal{P}^{\mathcal{C}}$ for Scene: [**Veach Bidir**] Algorithm: [**MLT**] Metric: [**SSIM**] True GT: [**BDPT @ 524288 spp**].

M-MLT	2	4	8	16	32	64	128	256	512	1024	2048	4096	8192	16384
GT (BDPT)														
262144	0.00009	0.00001	0.00001	0.00006	0.00002	-0.00003	0.00008	0.00005	0.00004	-0.00002	-0.00003	-0.00004	-0.00001	-0.00002
131072	0.00033	0.00021	0.00015	0.00036	0.00009	0.00009	0.00003	-0.00004	-0.00001	-0.00003	-0.00009	-0.00007	-0.00010	-0.00009
65536	0.00002	0.00002	0.00029	0.00010	0.00007	-0.00001	-0.00005	-0.00011	-0.00014	-0.00017	-0.00028	-0.00022	-0.00025	-0.00023
32768	0.00017	-0.00012	-0.00007	-0.00010	-0.00017	-0.00009	-0.00028	-0.00039	-0.00050	-0.00061	-0.00059	-0.00062	-0.00060	-0.00062
16384	0.00016	-0.00026	-0.00017	0.00045	-0.00014	-0.00015	-0.00017	-0.00042	-0.00051	-0.00049	-0.00063	-0.00064	-0.00065	
8192	0.00021	0.00057	0.00028	0.00026	-0.00040	-0.00054	-0.00055	-0.00089	-0.00099	-0.00114	-0.00126	-0.00129		
4096	0.00047	-0.00066	-0.00034	-0.00001	-0.00103	-0.00112	-0.00143	-0.00167	-0.00188	-0.00194	-0.00226			
2048	-0.00023	-0.00182	-0.00086	-0.00009	-0.00077	-0.00167	-0.00255	-0.00303	-0.00330	-0.00373				
1024	-0.00163	-0.00093	-0.00154	-0.00181	-0.00318	-0.00401	-0.00484	-0.00572	-0.00639					
512	-0.00143	-0.00338	-0.00385	-0.00530	-0.00675	-0.00845	-0.01063	-0.01228						
256	-0.00170	-0.00267	-0.00518	-0.00735	-0.01220	-0.01632	-0.02036							
128	-0.00796	-0.00781	-0.01276	-0.01538	-0.02319	-0.03237								
64	-0.01168	-0.01485	-0.02102	-0.02936	-0.04527									
32	-0.02546	-0.03262	-0.04280	-0.05928										
16	-0.04449	-0.05782	-0.08204											
8	-0.09494	-0.11791												
4	-0.16825													

(e) $\mathcal{P}^{\mathcal{C}}$ for Scene: [**Veach Bidir**] Algorithm: [**M-MLT**] Metric: [**SSIM**] True GT: [**BDPT @ 524288 spp**].

ERPT	2	4	8	16	32	64	128	256	512	1024	2048	4096	8192	16384	32768	65536	131072
GT (BDPT)																	
262144	0.00004	-0.00005	0.00006	-0.00007	0.00002	0.00002	0.00005	-0.00001	-0.00002	-0.00006	-0.00005	-0.00006	0.00000	-0.00010	-0.00007	-0.00004	-0.00003
131072	0.00009	0.00002	-0.00004	-0.00007	-0.00005	-0.00001	-0.00004	0.00001	-0.00007	-0.00008	0.00001	-0.00006	0.00005	-0.00013	-0.00002	-0.00009	
65536	-0.00018	-0.00013	-0.00017	-0.00024	-0.00013	-0.00018	-0.00019	-0.00021	-0.00027	-0.00031	-0.00034	-0.00023	-0.00020	-0.00020	-0.00018		
32768	-0.00048	-0.00038	-0.00051	-0.00041	-0.00040	-0.00035	-0.00037	-0.00037	-0.00032	-0.00047	-0.00049	-0.00042	-0.00052	-0.00046			
16384	-0.00057	-0.00053	-0.00034	-0.00055	-0.00040	-0.00069	-0.00056	-0.00075	-0.00086	-0.00072	-0.00050	-0.00079	-0.00066				
8192	-0.00077	-0.00092	-0.00088	-0.00102	-0.00100	-0.00124	-0.00115	-0.00081	-0.00110	-0.00124	-0.00133	-0.00128					
4096	-0.00184	-0.00135	-0.00196	-0.00167	-0.00171	-0.00172	-0.00166	-0.00170	-0.00216	-0.00169	-0.00167						
2048	-0.00301	-0.00319	-0.00256	-0.00313	-0.00284	-0.00310	-0.00314	-0.00314	-0.00313	-0.00303							
1024	-0.00505	-0.00523	-0.00503	-0.00516	-0.00537	-0.00604	-0.00534	-0.00603	-0.00571								
512	-0.01024	-0.01016	-0.01018	-0.01096	-0.01108	-0.01217	-0.01147	-0.01084									
256	-0.02108	-0.02144	-0.02185	-0.02164	-0.02187	-0.02137	-0.02206										
128	-0.04243	-0.04158	-0.04326	-0.04137	-0.04237	-0.04295											
64	-0.08024	-0.08014	-0.07838	-0.07901	-0.08154												
32	-0.15137	-0.14953	-0.15095	-0.15150													
16	-0.26451	-0.26401	-0.26577														
8	-0.44349	-0.44401															
4	-0.69227																

(f) $\mathcal{P}^{\mathcal{C}}$ for Scene: [**Veach Bidir**] Algorithm: [**ERPT**] Metric: [**SSIM**] True GT: [**BDPT @ 524288 spp**].

M-ERPT	2	4	8	16	32	64	128	256	512	1024	2048	4096	8192	16384	32768	65536	131072
GT (BDPT)																	
262144	-0.00006	0.00010	0.00006	-0.00001	-0.00002	-0.00006	0.00001	-0.00009	-0.00015	-0.00007	-0.00006	0.00003	-0.00005	-0.00002	-0.00011	-0.00011	0.00006
131072	-0.00006	-0.00006	-0.00005	-0.00005	0.00001	-0.00004	0.00000	-0.00006	-0.00009	-0.00008	-0.00005	-0.00004	0.00001	-0.00001	-0.00007	-0.00013	
65536	-0.00010	-0.00002	-0.00008	-0.00023	-0.00011	-0.00013	-0.00019	-0.00020	-0.00036	-0.00023	-0.00029	-0.00019	-0.00020	-0.00026	-0.00014		
32768	-0.00024	-0.00041	-0.00041	-0.00046	-0.00039	-0.00041	-0.00064	-0.00045	-0.00055	-0.00053	-0.00049	-0.00049	-0.00043	-0.00053			
16384	-0.00049	-0.00034	-0.00070	-0.00067	-0.00042	-0.00057	-0.00051	-0.00068	-0.00062	-0.00070	-0.00075	-0.00078	-0.00083				
8192	-0.00105	-0.00080	-0.00112	-0.00121	-0.00101	-0.00105	-0.00126	-0.00112	-0.00138	-0.00129	-0.00133	-0.00117					
4096	-0.00178	-0.00162	-0.00198	-0.00181	-0.00151	-0.00189	-0.00175	-0.00202	-0.00191	-0.00186	-0.00182						
2048	-0.00246	-0.00264	-0.00313	-0.00335	-0.00337	-0.00331	-0.00281	-0.00318	-0.00276	-0.00310							
1024	-0.00523	-0.00484	-0.00579	-0.00531	-0.00508	-0.00585	-0.00591	-0.00579	-0.00598								
512	-0.01074	-0.00996	-0.01141	-0.01151	-0.01104	-0.01144	-0.01207	-0.01195									
256	-0.02012	-0.02065	-0.02133	-0.02222	-0.02166	-0.02209	-0.02186										
128	-0.04175	-0.04024	-0.04209	-0.04189	-0.04313	-0.04230											
64	-0.07759	-0.07907	-0.07957	-0.07989	-0.08021												
32	-0.14573	-0.14559	-0.14941	-0.14813													
16	-0.26049	-0.25994	-0.26168														
8	-0.43463	-0.43440															
4	-0.67963																

(g) $\mathcal{P}^{\mathcal{C}}$ for Scene: [**Veach Bidir**] Algorithm: [**M-ERPT**] Metric: [**SSIM**] True GT: [**BDPT @ 524288 spp**].

Table 78

17.4 Veach Door

PT	2	4	8	16	32	64	128	256	512	1024	2048	4096	8192	16384	32768	65536	131072
GT (BDPT)																	
262144	-0.00008	-0.00047	-0.00043	-0.00040	-0.00055	-0.00022	-0.00039	-0.00014	-0.00008	-0.00039	-0.00013	-0.00021	-0.00037	-0.00034	-0.00042	-0.00054	-0.00045
131072	-0.00052	-0.00104	-0.00048	-0.00074	-0.00045	0.00009	-0.00048	-0.00014	-0.00044	-0.00049	-0.00070	-0.00076	-0.00094	-0.00099	-0.00117	-0.00125	
65536	0.00052	0.00013	0.00036	0.00051	-0.00029	0.00004	-0.00005	-0.00064	-0.00065	-0.00095	-0.00155	-0.00171	-0.00215	-0.00262	-0.00263		
32768	-0.00145	-0.00163	-0.00028	-0.00082	-0.00090	-0.00049	-0.00107	-0.00178	-0.00186	-0.00220	-0.00291	-0.00416	-0.00473	-0.00544			
16384	-0.00165	-0.00142	-0.00124	-0.00125	-0.00101	-0.00149	-0.00163	-0.00235	-0.00474	-0.00497	-0.00568	-0.00815	-0.01012				
8192	-0.00121	-0.00031	-0.00057	0.00069	0.00018	-0.00189	-0.00391	-0.00338	-0.00736	-0.00992	-0.01285	-0.01680					
4096	-0.00284	-0.00246	-0.00263	-0.00361	-0.00427	-0.00637	-0.00370	-0.00827	-0.01243	-0.01942	-0.02623						
2048	-0.00392	-0.00562	0.00008	-0.00931	-0.00547	-0.00615	-0.01018	-0.01846	-0.02919	-0.03599							
1024	-0.01393	-0.00938	-0.00764	-0.00878	-0.00897	-0.01462	-0.02453	-0.03975	-0.04974								
512	-0.00755	-0.00467	-0.00892	-0.00822	-0.01675	-0.02456	-0.04018	-0.06453									
256	0.01728	0.02039	0.00399	-0.00123	-0.02333	-0.05191	-0.07838										
128	0.04687	0.03939	0.00659	-0.01133	-0.03892	-0.07500											
64	0.11053	0.06620	0.04729	-0.00006	-0.07223												
32	0.17040	0.09657	0.01563	-0.03414													
16	0.23209	0.14397	0.01479														
8	0.25579	0.12961															
4	0.24851																

(a) $\mathcal{P}^{\mathcal{C}}$ for Scene: [**Veach Door**] Algorithm: [**PT**] Metric: [**SSIM**] True GT: [**BDPT @ 524288 spp**].

BDPT	2	4	8	16	32	64	128	256	512	1024	2048	4096	8192	16384	32768	65536	131072
GT (BDPT)																	
262144	-0.00049	-0.00006	-0.00017	-0.00020	-0.00017	-0.00021	0.00001	-0.00013	-0.00028	-0.00031	-0.00043	-0.00046	-0.00037	-0.00045	-0.00049	-0.00048	-0.00049
131072	-0.00105	-0.00027	0.00005	-0.00042	0.00008	0.00016	-0.00056	-0.00056	-0.00076	-0.00091	-0.00111	-0.00110	-0.00116	-0.00124	-0.00125	-0.00127	
65536	0.00058	0.00083	0.00021	0.00019	-0.00033	-0.00053	-0.00049	-0.00113	-0.00166	-0.00212	-0.00237	-0.00269	-0.00275	-0.00293	-0.00301		
32768	-0.00026	-0.00049	-0.00053	-0.00102	-0.00091	-0.00085	-0.00126	-0.00294	-0.00402	-0.00451	-0.00550	-0.00595	-0.00627	-0.00648			
16384	-0.00177	-0.00112	-0.00106	-0.00071	-0.00093	-0.00260	-0.00400	-0.00493	-0.00753	-0.00906	-0.01065	-0.01204	-0.01267				
8192	-0.00098	0.00028	-0.00038	-0.00215	-0.00241	-0.00613	-0.00708	-0.01069	-0.01511	-0.01881	-0.02177	-0.02431					
4096	-0.00100	-0.00336	-0.00422	-0.00564	-0.00543	-0.01030	-0.01486	-0.02069	-0.02982	-0.03633	-0.04248						
2048	-0.00457	-0.00705	-0.00816	-0.00792	-0.00991	-0.01916	-0.02720	-0.04188	-0.05612	-0.07076							
1024	-0.00902	-0.01146	-0.00751	-0.01384	-0.02147	-0.03174	-0.05321	-0.07868	-0.10833								
512	-0.00360	-0.00773	-0.01598	-0.01842	-0.03403	-0.06017	-0.10094	-0.14240									
256	-0.00044	0.00019	-0.00755	-0.02588	-0.05378	-0.10706	-0.16593										
128	0.01882	0.01456	-0.01687	-0.05254	-0.10101	-0.17695											
64	0.06636	0.02789	-0.02460	-0.07966	-0.16532												
32	0.08996	0.05400	-0.03062	-0.14854													
16	0.10965	-0.00265	-0.08111														
8	0.10816	-0.03146															
4	0.06422																

(b) $\mathcal{P}^{\mathcal{C}}$ for Scene: [**Veach Door**] Algorithm: [**BDPT**] Metric: [**SSIM**] True GT: [**BDPT @ 524288 spp**].

PSSMLT	2	4	8	16	32	64	128	256	512	1024	2048	4096	8192	16384
GT (BDPT)														
262144	-0.00037	-0.00023	0.00026	0.00010	-0.00014	-0.00010	-0.00030	-0.00018	-0.00031	-0.00036	-0.00044	-0.00044	-0.00046	-0.00049
131072	-0.00057	-0.00032	-0.00044	0.00002	-0.00030	-0.00045	-0.00076	-0.00075	-0.00084	-0.00099	-0.00119	-0.00121	-0.00120	-0.00126
65536	0.00012	-0.00019	-0.00027	0.00014	-0.00075	-0.00084	-0.00134	-0.00161	-0.00210	-0.00241	-0.00271	-0.00281	-0.00287	-0.00295
32768	-0.00131	-0.00137	-0.00101	-0.00117	-0.00204	-0.00287	-0.00364	-0.00445	-0.00532	-0.00586	-0.00624	-0.00641	-0.00655	-0.00666
16384	-0.00076	-0.00120	-0.00158	-0.00216	-0.00349	-0.00426	-0.00639	-0.00806	-0.01016	-0.01118	-0.01214	-0.01270	-0.01304	
8192	-0.00186	-0.00187	-0.00232	-0.00327	-0.00551	-0.00867	-0.01339	-0.01629	-0.01992	-0.02192	-0.02428	-0.02532		
4096	-0.00458	-0.00480	-0.00473	-0.00878	-0.01381	-0.01864	-0.02632	-0.03408	-0.03993	-0.04531	-0.04777			
2048	-0.00423	-0.00494	-0.00995	-0.01601	-0.02128	-0.03521	-0.05048	-0.06231	-0.07524	-0.08353				
1024	-0.00966	-0.01387	-0.01783	-0.03022	-0.04748	-0.07105	-0.09592	-0.12052	-0.14134					
512	-0.01738	-0.01974	-0.03322	-0.05399	-0.08531	-0.12842	-0.17295	-0.21435						
256	-0.01293	-0.02581	-0.05222	-0.09602	-0.15041	-0.21701	-0.28927							
128	-0.02356	-0.05017	-0.08747	-0.15086	-0.23716	-0.34984								
64	-0.04607	-0.07518	-0.14729	-0.25282	-0.38214									
32	-0.05241	-0.14569	-0.26614	-0.38776										
16	-0.09766	-0.19510	-0.35761											
8	-0.15570	-0.30251												
4	-0.28868													

(c) $\mathcal{P}^{\mathcal{C}}$ for Scene: [**Veach Door**] Algorithm: [**PSSMLT**] Metric: [**SSIM**] True GT: [**BDPT @ 524288 spp**].

MLT	2	4	8	16	32	64	128	256	512	1024	2048	4096	8192	16384
GT (BDPT)														
262144	0.00027	-0.00007	-0.00041	-0.00016	-0.00027	-0.00032	-0.00019	-0.00045	-0.00029	-0.00047	-0.00039	-0.00045	-0.00049	-0.00046
131072	0.00024	-0.00004	-0.00022	0.00004	-0.00056	-0.00059	-0.00063	-0.00087	-0.00105	-0.00115	-0.00124	-0.00127	-0.00125	-0.00128
65536	0.00092	0.00009	-0.00003	-0.00003	-0.00101	-0.00160	-0.00177	-0.00235	-0.00247	-0.00267	-0.00272	-0.00290	-0.00289	-0.00298
32768	0.00011	-0.00070	-0.00098	-0.00050	-0.00229	-0.00289	-0.00445	-0.00493	-0.00533	-0.00578	-0.00619	-0.00650	-0.00660	-0.00662
16384	-0.00071	-0.00056	-0.00165	-0.00276	-0.00407	-0.00617	-0.00858	-0.01011	-0.01130	-0.01222	-0.01280	-0.01299	-0.01318	
8192	-0.00111	-0.00095	-0.00266	-0.00478	-0.00851	-0.01167	-0.01707	-0.01945	-0.02262	-0.02441	-0.02527	-0.02597		
4096	-0.00329	-0.00458	-0.00512	-0.00941	-0.01644	-0.02207	-0.03159	-0.03894	-0.04340	-0.04686	-0.04944			
2048	-0.00875	-0.00834	-0.01106	-0.02097	-0.03026	-0.04612	-0.05976	-0.07331	-0.08238	-0.08893				
1024	-0.01234	-0.01570	-0.02106	-0.03822	-0.06383	-0.08897	-0.11550	-0.13619	-0.15312					
512	-0.01555	-0.02579	-0.03607	-0.06461	-0.10896	-0.15583	-0.20422	-0.23859						
256	-0.01564	-0.03598	-0.06440	-0.11077	-0.18619	-0.26206	-0.33658							
128	-0.00955	-0.05420	-0.11427	-0.19095	-0.30536	-0.41304								
64	-0.03915	-0.06894	-0.17058	-0.29336	-0.45895									
32	-0.04149	-0.14786	-0.27681	-0.44159										
16	-0.07032	-0.22079	-0.40698											
8	-0.13172	-0.33766												
4	-0.26189													

(d) $\mathcal{P}^{\mathcal{C}}$ for Scene: [**Veach Door**] Algorithm: [**MLT**] Metric: [**SSIM**] True GT: [**BDPT @ 524288 spp**].

M-MLT	2	4	8	16	32	64	128	256	512	1024	2048	4096	8192	16384
GT (BDPT)														
262144	-0.00033	0.00012	-0.00024	-0.00012	0.00011	-0.00017	-0.00022	-0.00041	-0.00051	-0.00040	-0.00047	-0.00047	-0.00046	-0.00049
131072	-0.00048	-0.00036	0.00002	-0.00046	-0.00026	-0.00063	-0.00091	-0.00082	-0.00115	-0.00111	-0.00123	-0.00125	-0.00131	-0.00125
65536	-0.00030	0.00003	0.00059	-0.00028	-0.00063	-0.00123	-0.00167	-0.00227	-0.00239	-0.00278	-0.00286	-0.00290	-0.00299	-0.00301
32768	-0.00074	-0.00084	-0.00056	-0.00126	-0.00159	-0.00266	-0.00380	-0.00458	-0.00541	-0.00587	-0.00627	-0.00643	-0.00658	-0.00665
16384	-0.00194	-0.00051	-0.00169	-0.00229	-0.00448	-0.00499	-0.00752	-0.00977	-0.01066	-0.01199	-0.01251	-0.01307	-0.01325	
8192	-0.00116	-0.00159	-0.00202	-0.00594	-0.00797	-0.01180	-0.01547	-0.01942	-0.02221	-0.02419	-0.02519	-0.02572		
4096	-0.00286	-0.00310	-0.00526	-0.00854	-0.01723	-0.02282	-0.03281	-0.03828	-0.04367	-0.04695	-0.04965			
2048	-0.00673	-0.00745	-0.01238	-0.01678	-0.02984	-0.04511	-0.06121	-0.07335	-0.08180	-0.08935				
1024	-0.01032	-0.01715	-0.02358	-0.03457	-0.06054	-0.08658	-0.11217	-0.13607	-0.15260					
512	-0.01280	-0.02032	-0.03699	-0.06509	-0.11114	-0.15494	-0.20224	-0.23908						
256	-0.01051	-0.03397	-0.06869	-0.11890	-0.18791	-0.26117	-0.33150							
128	-0.02699	-0.04689	-0.10903	-0.19535	-0.30600	-0.41311								
64	-0.01650	-0.07213	-0.17970	-0.30070	-0.46018									
32	-0.05966	-0.15045	-0.30157	-0.43898										
16	-0.08191	-0.22153	-0.43172											
8	-0.15337	-0.36371												
4	-0.29122													

(e) $\mathcal{P}^{\mathcal{C}}$ for Scene: [**Veach Door**] Algorithm: [**M-MLT**] Metric: [**SSIM**] True GT: [**BDPT @ 524288 spp**].

ERPT	2	4	8	16	32	64	128	256	512	1024	2048	4096	8192	16384	32768	65536	131072
GT (BDPT)																	
262144	-0.00013	-0.00022	-0.00042	-0.00016	-0.00041	-0.00023	-0.00042	-0.00024	-0.00021	-0.00013	-0.00029	-0.00028	-0.00041	-0.00035	-0.00025	-0.00041	-0.00036
131072	-0.00078	-0.00070	-0.00114	-0.00083	-0.00094	-0.00104	-0.00109	-0.00088	-0.00098	-0.00096	-0.00078	-0.00095	-0.00097	-0.00088	-0.00064	-0.00088	
65536	-0.00208	-0.00184	-0.00225	-0.00189	-0.00206	-0.00227	-0.00188	-0.00210	-0.00223	-0.00200	-0.00176	-0.00219	-0.00206	-0.00186	-0.00208		
32768	-0.00461	-0.00441	-0.00453	-0.00468	-0.00483	-0.00451	-0.00434	-0.00438	-0.00453	-0.00453	-0.00403	-0.00438	-0.00447	-0.00450			
16384	-0.00854	-0.00921	-0.00916	-0.00910	-0.00913	-0.00884	-0.00879	-0.00872	-0.00874	-0.00929	-0.00919	-0.00891	-0.00892				
8192	-0.01671	-0.01672	-0.01834	-0.01802	-0.01771	-0.01828	-0.01823	-0.01776	-0.01722	-0.01819	-0.01811	-0.01759					
4096	-0.03420	-0.03407	-0.03469	-0.03521	-0.03437	-0.03508	-0.03546	-0.03431	-0.03565	-0.03428	-0.03476						
2048	-0.06666	-0.06633	-0.06683	-0.06821	-0.06679	-0.06670	-0.06582	-0.06680	-0.06604	-0.06543							
1024	-0.12358	-0.12508	-0.12483	-0.12534	-0.12528	-0.12556	-0.12507	-0.12425	-0.12455								
512	-0.21744	-0.21881	-0.22223	-0.21881	-0.21906	-0.22193	-0.21662	-0.22092									
256	-0.35455	-0.35740	-0.35959	-0.35820	-0.36051	-0.36148	-0.36183										
128	-0.54206	-0.54824	-0.55422	-0.55214	-0.55536	-0.55292											
64	-0.75893	-0.76178	-0.77281	-0.76888	-0.77473												
32	-1.00121	-1.00597	-1.01848	-1.01367													
16	-1.24727	-1.26099	-1.27762														
8	-1.50634	-1.51730															
4	-1.78499																

(f) $\mathcal{P}^{\mathcal{C}}$ for Scene: [**Veach Door**] Algorithm: [**ERPT**] Metric: [**SSIM**] True GT: [**BDPT @ 524288 spp**].

M-ERPT	2	4	8	16	32	64	128	256	512	1024	2048	4096	8192	16384	32768	65536	131072
GT (BDPT)																	
262144	-0.00011	-0.00036	-0.00032	-0.00018	-0.00039	-0.00018	-0.00052	-0.00018	-0.00042	-0.00041	-0.00023	-0.00031	-0.00022	-0.00008	-0.00045	-0.00021	-0.00038
131072	-0.00078	-0.00075	-0.00091	-0.00076	-0.00059	-0.00079	-0.00096	-0.00085	-0.00068	-0.00087	-0.00062	-0.00081	-0.00099	-0.00079	-0.00086	-0.00075	
65536	-0.00186	-0.00181	-0.00173	-0.00165	-0.00207	-0.00172	-0.00209	-0.00193	-0.00169	-0.00188	-0.00167	-0.00185	-0.00218	-0.00199	-0.00193		
32768	-0.00412	-0.00432	-0.00404	-0.00397	-0.00440	-0.00439	-0.00413	-0.00450	-0.00444	-0.00395	-0.00407	-0.00390	-0.00448	-0.00396			
16384	-0.00782	-0.00824	-0.00806	-0.00878	-0.00835	-0.00848	-0.00834	-0.00827	-0.00834	-0.00828	-0.00851	-0.00894	-0.00854				
8192	-0.01609	-0.01624	-0.01597	-0.01641	-0.01700	-0.01690	-0.01656	-0.01705	-0.01711	-0.01667	-0.01710	-0.01811					
4096	-0.03223	-0.03428	-0.03253	-0.03280	-0.03287	-0.03382	-0.03351	-0.03335	-0.03385	-0.03324	-0.03270						
2048	-0.06358	-0.06448	-0.06463	-0.06349	-0.06262	-0.06481	-0.06422	-0.06415	-0.06376	-0.06454							
1024	-0.11772	-0.11858	-0.12212	-0.11864	-0.11810	-0.12083	-0.12016	-0.12081	-0.11703								
512	-0.20732	-0.20857	-0.20689	-0.21011	-0.21033	-0.21276	-0.20901	-0.21163									
256	-0.34782	-0.34416	-0.34397	-0.34466	-0.34451	-0.34501	-0.34814										
128	-0.52976	-0.52776	-0.52951	-0.53048	-0.53287	-0.53008											
64	-0.74650	-0.74340	-0.74512	-0.74941	-0.74699												
32	-0.99386	-0.99053	-0.98513	-0.98872													
16	-1.24151	-1.23008	-1.24204														
8	-1.50067	-1.49727															
4	-1.78842																

(g) $\mathcal{P}^{\mathcal{C}}$ for Scene: [**Veach Door**] Algorithm: [**M-ERPT**] Metric: [**SSIM**] True GT: [**BDPT @ 524288 spp**].

Table 79

17.5 Sponza

PT	2	4	8	16	32	64	128	256	512	1024	2048	4096	8192
GT (PT)													
16384	-0.00132	-0.00076	-0.00007	-0.00126	-0.00095	-0.00053	-0.00087	-0.00236	-0.00234	-0.00248	-0.00287	-0.00296	-0.00299
8192	0.00094	-0.00029	-0.00017	-0.00077	-0.00260	-0.00257	-0.00308	-0.00553	-0.00720	-0.00778	-0.00848	-0.00880	
4096	-0.00079	-0.00183	-0.00229	-0.00267	-0.00551	-0.00605	-0.00811	-0.01276	-0.01695	-0.01784	-0.01970		
2048	-0.00018	-0.00062	-0.00069	-0.00344	-0.00843	-0.01168	-0.01817	-0.02681	-0.03270	-0.03719			
1024	-0.00192	-0.00430	-0.00321	-0.01011	-0.01645	-0.02663	-0.03977	-0.05297	-0.06519				
512	-0.00581	-0.01274	-0.00784	-0.01715	-0.02861	-0.04785	-0.07713	-0.09908					
256	-0.00952	-0.01421	-0.01462	-0.03311	-0.05618	-0.09344	-0.13925						
128	-0.01238	-0.02198	-0.02897	-0.06254	-0.10968	-0.17603							
64	-0.01986	-0.03398	-0.06852	-0.12534	-0.20461								
32	-0.03791	-0.06935	-0.10605	-0.20454									
16	-0.07628	-0.12860	-0.19050										
8	-0.11790	-0.22081											
4	-0.18060												

(a) $\mathcal{P}^{\mathcal{C}}$ for Scene: [**Sponza**] Algorithm: [**PT**] Metric: [**SSIM**] True GT: [**PT @ 32768 spp**].

BDPT	2	4	8	16	32	64	128	256	512	1024	2048	4096	8192
GT (PT)													
16384	0.00070	0.00041	-0.00019	0.00013	-0.00028	-0.00015	-0.00094	-0.00150	-0.00229	-0.00283	-0.00268	-0.00288	-0.00304
8192	-0.00063	-0.00037	-0.00153	-0.00020	-0.00189	-0.00247	-0.00290	-0.00492	-0.00665	-0.00779	-0.00794	-0.00869	
4096	-0.00055	0.00106	-0.00139	-0.00058	-0.00469	-0.00494	-0.00806	-0.01136	-0.01471	-0.01774	-0.01951		
2048	-0.00204	-0.00234	-0.00308	-0.00219	-0.00656	-0.00995	-0.01722	-0.02500	-0.03120	-0.03690			
1024	-0.00013	0.00162	-0.00712	-0.00709	-0.01203	-0.02124	-0.03239	-0.04677	-0.06232				
512	-0.00021	-0.00510	-0.00677	-0.01547	-0.02365	-0.04015	-0.06734	-0.09435					
256	-0.00271	-0.00898	-0.01378	-0.02604	-0.04923	-0.07314	-0.12489						
128	-0.00886	-0.01842	-0.03380	-0.04469	-0.09314	-0.15076							
64	-0.00403	-0.02792	-0.04984	-0.09664	-0.16714								
32	-0.00672	-0.08211	-0.09524	-0.17152									
16	-0.05988	-0.06673	-0.17603										
8	-0.09221	-0.14752											
4	-0.15826												

(b) $\mathcal{P}^{\mathcal{C}}$ for Scene: [**Sponza**] Algorithm: [**BDPT**] Metric: [**SSIM**] True GT: [**PT @ 32768 spp**].

PSSMLT	2	4	8	16	32	64	128	256	512	1024	2048	4096	8192
GT (PT)													
16384	-0.00064	-0.00059	0.00022	0.00016	-0.00031	-0.00017	-0.00111	-0.00124	-0.00209	-0.00237	-0.00257	-0.00276	-0.00293
8192	-0.00159	-0.00095	-0.00138	-0.00178	-0.00202	-0.00218	-0.00385	-0.00449	-0.00626	-0.00690	-0.00775	-0.00840	
4096	-0.00134	-0.00148	-0.00198	-0.00263	-0.00539	-0.00674	-0.00958	-0.01215	-0.01477	-0.01647	-0.01793		
2048	-0.00014	0.00023	-0.00194	-0.00546	-0.00982	-0.01218	-0.01890	-0.02499	-0.03017	-0.03464			
1024	-0.00173	-0.00287	-0.00682	-0.01108	-0.01493	-0.02704	-0.03749	-0.04972	-0.06126				
512	-0.00625	-0.00947	-0.01438	-0.02167	-0.03917	-0.05412	-0.07466	-0.09795					
256	-0.01145	-0.01484	-0.02561	-0.04630	-0.07223	-0.10236	-0.13642						
128	-0.02894	-0.03566	-0.05472	-0.08952	-0.13763	-0.18755							
64	-0.05032	-0.07830	-0.12014	-0.17503	-0.23704								
32	-0.08296	-0.12504	-0.17811	-0.28115									
16	-0.16172	-0.23288	-0.32425										
8	-0.23734	-0.34259											
4	-0.35905												

(c) $\mathcal{P}^{\mathcal{C}}$ for Scene: [**Sponza**] Algorithm: [**PSSMLT**] Metric: [**SSIM**] True GT: [**PT @ 32768 spp**].

MLT	2	4	8	16	32	64	128	256	512	1024	2048	4096	8192
GT (PT)													
16384	-0.00091	-0.00081	-0.00056	-0.00079	-0.00116	-0.00119	-0.00160	-0.00220	-0.00247	-0.00276	-0.00294	-0.00296	-0.00288
8192	-0.00064	0.00038	-0.00279	-0.00182	-0.00300	-0.00399	-0.00641	-0.00688	-0.00775	-0.00850	-0.00897	-0.00860	
4096	-0.00059	-0.00175	-0.00335	-0.00568	-0.00746	-0.01061	-0.01384	-0.01641	-0.01799	-0.01966	-0.01992		
2048	-0.00271	-0.00366	-0.00673	-0.00882	-0.01648	-0.02254	-0.02910	-0.03402	-0.03749	-0.03972			
1024	-0.00497	-0.00770	-0.01016	-0.02203	-0.03422	-0.04744	-0.06028	-0.06850	-0.07494				
512	-0.00967	-0.01497	-0.02261	-0.03941	-0.06584	-0.08730	-0.11375	-0.12938					
256	-0.01494	-0.02953	-0.04554	-0.08733	-0.12567	-0.16622	-0.20298						
128	-0.02804	-0.05248	-0.09135	-0.14373	-0.21980	-0.28910							
64	-0.04775	-0.09540	-0.14962	-0.24497	-0.35176								
32	-0.08324	-0.14562	-0.27011	-0.39201									
16	-0.14239	-0.26074	-0.39851										
8	-0.22779	-0.41193											
4	-0.39500												

(d) $\mathcal{P}^{\mathcal{C}}$ for Scene: [**Sponza**] Algorithm: [**MLT**] Metric: [**SSIM**] True GT: [**PT @ 32768 spp**].

M-MLT	2	4	8	16	32	64	128	256	512	1024	2048	4096	8192
GT (PT)													
16384	-0.00143	-0.00009	-0.00082	-0.00059	-0.00108	-0.00094	-0.00170	-0.00265	-0.00248	-0.00277	-0.00275	-0.00284	-0.00297
8192	-0.00001	-0.00002	-0.00037	-0.00188	-0.00379	-0.00484	-0.00606	-0.00693	-0.00816	-0.00801	-0.00835	-0.00830	
4096	-0.00208	-0.00125	-0.00251	-0.00463	-0.00823	-0.01156	-0.01377	-0.01716	-0.01788	-0.01935	-0.01953		
2048	-0.00172	-0.00126	-0.00508	-0.01113	-0.01777	-0.02321	-0.02990	-0.03427	-0.03833	-0.03936			
1024	-0.00402	-0.00695	-0.01176	-0.02072	-0.03323	-0.04890	-0.05875	-0.06923	-0.07609				
512	-0.01009	-0.01304	-0.02187	-0.04020	-0.06596	-0.09016	-0.11493	-0.13097					
256	-0.02274	-0.02089	-0.04634	-0.08179	-0.12031	-0.16671	-0.20456						
128	-0.03412	-0.05227	-0.09829	-0.14771	-0.22169	-0.29025							
64	-0.05678	-0.08870	-0.16562	-0.25259	-0.36673								
32	-0.08589	-0.16031	-0.27236	-0.40654									
16	-0.16649	-0.27537	-0.44566										
8	-0.24676	-0.41288											
4	-0.41292												

(e) $\mathcal{P}^{\mathcal{C}}$ for Scene: [**Sponza**] Algorithm: [**M-MLT**] Metric: [**SSIM**] True GT: [**PT @ 32768 spp**].

ERPT	2	4	8	16	32	64	128	256	512	1024	2048	4096	8192
GT (PT)													
16384	-0.00253	-0.00229	-0.00203	-0.00201	-0.00230	-0.00246	-0.00237	-0.00211	-0.00237	-0.00269	-0.00259	-0.00250	-0.00216
8192	-0.00686	-0.00675	-0.00688	-0.00628	-0.00659	-0.00692	-0.00636	-0.00643	-0.00665	-0.00625	-0.00637	-0.00696	
4096	-0.01577	-0.01505	-0.01515	-0.01556	-0.01542	-0.01607	-0.01533	-0.01511	-0.01545	-0.01611	-0.01513		
2048	-0.03192	-0.03188	-0.03188	-0.03159	-0.03152	-0.03297	-0.03136	-0.03284	-0.03304	-0.03172			
1024	-0.06384	-0.06436	-0.06560	-0.06347	-0.06505	-0.06438	-0.06416	-0.06192	-0.06425				
512	-0.12164	-0.12262	-0.12003	-0.12012	-0.12087	-0.12310	-0.12071	-0.12154					
256	-0.21486	-0.21424	-0.21676	-0.21580	-0.21709	-0.21564	-0.21515						
128	-0.36506	-0.36411	-0.36564	-0.36534	-0.36349	-0.36493							
64	-0.55722	-0.55572	-0.56065	-0.55846	-0.56156								
32	-0.79908	-0.79232	-0.78864	-0.79460									
16	-1.06478	-1.06731	-1.06191										
8	-1.34778	-1.34463											
4	-1.63423												

(f) $\mathcal{P}^{\mathcal{C}}$ for Scene: [**Sponza**] Algorithm: [**ERPT**] Metric: [**SSIM**] True GT: [**PT @ 32768 spp**].

M-ERPT	2	4	8	16	32	64	128	256	512	1024	2048	4096	8192
GT (PT)													
16384	-0.00177	-0.00206	-0.00215	-0.00237	-0.00244	-0.00173	-0.00203	-0.00160	-0.00239	-0.00198	-0.00245	-0.00192	-0.00196
8192	-0.00627	-0.00679	-0.00651	-0.00641	-0.00650	-0.00688	-0.00629	-0.00606	-0.00628	-0.00624	-0.00677	-0.00632	
4096	-0.01505	-0.01545	-0.01557	-0.01499	-0.01538	-0.01490	-0.01539	-0.01427	-0.01387	-0.01502	-0.01469		
2048	-0.03071	-0.03141	-0.03107	-0.03024	-0.03195	-0.03109	-0.02990	-0.03008	-0.03170	-0.03036			
1024	-0.06238	-0.06117	-0.06323	-0.06174	-0.06245	-0.06158	-0.06340	-0.06315	-0.06354				
512	-0.11572	-0.11952	-0.11741	-0.11764	-0.11955	-0.11907	-0.11840	-0.11743					
256	-0.20497	-0.21212	-0.21184	-0.20991	-0.21464	-0.21275	-0.20821						
128	-0.35589	-0.35968	-0.35545	-0.35048	-0.36019	-0.35828							
64	-0.54741	-0.54869	-0.54574	-0.54909	-0.55034								
32	-0.78184	-0.78681	-0.78176	-0.78835									
16	-1.05192	-1.05644	-1.05671										
8	-1.33220	-1.33846											
4	-1.62146												

(g) $\mathcal{P}^{\mathcal{C}}$ for Scene: [**Sponza**] Algorithm: [**M-ERPT**] Metric: [**SSIM**] True GT: [**PT @ 32768 spp**].

Table 80

18 IW-PSNR

18.1 Cornell Box

PT	2	4	8	16	32	64	128	256	512	1024	2048	4096	8192
GT (PT)													
16384	-0.00023	-0.00006	-0.00017	-0.00014	-0.00073	0.00057	-0.00034	-0.00125	-0.00163	-0.00159	-0.00352	-0.00582	-0.01207
8192	-0.00079	-0.00048	-0.00062	-0.00063	-0.00074	-0.00078	-0.00232	-0.00164	-0.00538	-0.00778	-0.01095	-0.02004	
4096	-0.00115	-0.00130	-0.00120	-0.00116	-0.00198	-0.00158	-0.00368	-0.00559	-0.00851	-0.01691	-0.02508		
2048	-0.00214	-0.00191	-0.00204	-0.00213	-0.00249	-0.00397	-0.00642	-0.01188	-0.02055	-0.03109			
1024	-0.00261	-0.00253	-0.00298	-0.00349	-0.00399	-0.00788	-0.01396	-0.01694	-0.03895				
512	-0.00406	-0.00377	-0.00414	-0.00658	-0.00850	-0.01207	-0.02039	-0.03474					
256	-0.00414	-0.00532	-0.00585	-0.01074	-0.01453	-0.02513	-0.04835						
128	-0.00561	-0.00531	-0.00762	-0.01294	-0.02386	-0.04202							
64	-0.00754	-0.00878	-0.01546	-0.02110	-0.04314								
32	-0.01216	-0.01489	-0.02622	-0.04081									
16	-0.01447	-0.02245	-0.03337										
8	-0.01818	-0.03568											
4	-0.03062												

(a) $\mathcal{P}^{\mathcal{C}}$ for Scene: **[Cornell Box]** Algorithm: **[PT]** Metric: **[IW-PSNR]** True GT: **[PT @ 32768 spp]**.

BDPT	2	4	8	16	32	64	128	256	512	1024	2048	4096	8192
GT (PT)													
16384	-0.00010	-0.00019	-0.00045	-0.00053	-0.00009	-0.00063	-0.00056	-0.00101	-0.00402	-0.00420	-0.00969	-0.01056	-0.01552
8192	-0.00070	-0.00058	-0.00089	-0.00140	-0.00095	-0.00127	-0.00380	-0.00482	-0.01146	-0.01443	-0.02509	-0.03432	
4096	-0.00090	-0.00127	-0.00153	-0.00133	-0.00205	-0.00335	-0.00569	-0.01207	-0.01986	-0.03334	-0.05098		
2048	-0.00134	-0.00133	-0.00238	-0.00340	-0.00457	-0.00793	-0.01463	-0.02279	-0.03982	-0.05730			
1024	-0.00264	-0.00264	-0.00330	-0.00529	-0.00750	-0.01088	-0.02609	-0.04502	-0.07031				
512	-0.00378	-0.00446	-0.00701	-0.01087	-0.01522	-0.02672	-0.04275	-0.07367					
256	-0.00556	-0.00790	-0.01211	-0.02054	-0.02907	-0.04741	-0.08326						
128	-0.00753	-0.01146	-0.01623	-0.02738	-0.05211	-0.08453							
64	-0.01157	-0.02184	-0.03613	-0.05635	-0.08795								
32	-0.01939	-0.03044	-0.05284	-0.09870									
16	-0.02936	-0.05020	-0.08996										
8	-0.04935	-0.08840											
4	-0.10026												

(b) $\mathcal{P}^{\mathcal{C}}$ for Scene: **[Cornell Box]** Algorithm: **[BDPT]** Metric: **[IW-PSNR]** True GT: **[PT @ 32768 spp]**.

PSSMLT	2	4	8	16	32	64	128	256	512	1024	2048	4096	8192
GT (PT)													
16384	-0.00022	-0.00025	-0.00021	-0.00023	-0.00033	-0.00100	-0.00147	-0.00144	-0.00131	-0.00315	-0.00334	-0.00396	-0.00289
8192	-0.00091	-0.00095	-0.00071	-0.00094	-0.00085	-0.00183	-0.00212	-0.00276	-0.00441	-0.00565	-0.00725	-0.00828	
4096	-0.00125	-0.00141	-0.00147	-0.00127	-0.00162	-0.00270	-0.00380	-0.00503	-0.00767	-0.01160	-0.01522		
2048	-0.00207	-0.00197	-0.00245	-0.00287	-0.00414	-0.00553	-0.00757	-0.01252	-0.01781	-0.02496			
1024	-0.00296	-0.00331	-0.00361	-0.00408	-0.00595	-0.01096	-0.01674	-0.02558	-0.03423				
512	-0.00462	-0.00469	-0.00580	-0.00687	-0.01046	-0.01801	-0.02968	-0.04322					
256	-0.00591	-0.00727	-0.01018	-0.01544	-0.02335	-0.03711	-0.05743						
128	-0.00799	-0.01093	-0.01612	-0.02589	-0.04208	-0.06419							
64	-0.01240	-0.01854	-0.02874	-0.04407	-0.06945								
32	-0.01947	-0.03147	-0.04760	-0.07668									
16	-0.02509	-0.04696	-0.08179										
8	-0.04103	-0.08038											
4	-0.08539												

(c) $\mathcal{P}^{\mathcal{C}}$ for Scene: **[Cornell Box]** Algorithm: **[PSSMLT]** Metric: **[IW-PSNR]** True GT: **[PT @ 32768 spp]**.

MLT	2	4	8	16	32	64	128	256	512	1024	2048	4096	8192
GT (PT)													
16384	-0.00033	-0.00026	-0.00024	-0.00013	-0.00004	0.00010	-0.00004	-0.00052	-0.00045	-0.00045	-0.00012	-0.00090	-0.00065
8192	-0.00070	-0.00061	-0.00049	-0.00049	-0.00040	-0.00025	-0.00050	-0.00088	-0.00185	-0.00316	-0.00338	-0.00417	
4096	-0.00100	-0.00087	-0.00073	-0.00073	-0.00049	-0.00048	-0.00105	-0.00199	-0.00337	-0.00388	-0.00561		
2048	-0.00158	-0.00135	-0.00112	-0.00065	-0.00103	-0.00149	-0.00218	-0.00369	-0.00707	-0.01139			
1024	-0.00196	-0.00159	-0.00137	-0.00163	-0.00205	-0.00322	-0.00495	-0.00779	-0.01261				
512	-0.00220	-0.00200	-0.00216	-0.00249	-0.00340	-0.00466	-0.00880	-0.01531					
256	-0.00217	-0.00224	-0.00299	-0.00398	-0.00684	-0.01061	-0.01884						
128	-0.00235	-0.00244	-0.00254	-0.00336	-0.00700	-0.01422							
64	-0.00267	-0.00346	-0.00559	-0.00958	-0.01864								
32	-0.00457	-0.00593	-0.00969	-0.01653									
16	-0.00427	-0.00838	-0.01637										
8	-0.00666	-0.01657											
4	-0.01180												

(d) $\mathcal{P}^{\mathcal{C}}$ for Scene: **[Cornell Box]** Algorithm: **[MLT]** Metric: **[IW-PSNR]** True GT: **[PT @ 32768 spp]**.

M-MLT	2	4	8	16	32	64	128	256	512	1024	2048	4096	8192
GT (PT)													
16384	-0.00027	-0.00021	-0.00019	-0.00012	-0.00009	-0.00007	-0.00036	-0.00071	-0.00078	-0.00101	-0.00165	-0.00226	-0.00178
8192	-0.00059	-0.00064	-0.00059	-0.00054	-0.00058	-0.00048	-0.00066	-0.00115	-0.00214	-0.00197	-0.00307	-0.00336	
4096	-0.00089	-0.00091	-0.00076	-0.00087	-0.00101	-0.00103	-0.00149	-0.00192	-0.00370	-0.00486	-0.00606		
2048	-0.00135	-0.00127	-0.00119	-0.00128	-0.00143	-0.00127	-0.00186	-0.00406	-0.00706	-0.01080			
1024	-0.00178	-0.00142	-0.00143	-0.00155	-0.00196	-0.00237	-0.00424	-0.00856	-0.01283				
512	-0.00186	-0.00222	-0.00194	-0.00302	-0.00372	-0.00440	-0.00817	-0.01372					
256	-0.00249	-0.00228	-0.00264	-0.00357	-0.00520	-0.00787	-0.01476						
128	-0.00145	-0.00151	-0.00220	-0.00480	-0.00822	-0.01522							
64	-0.00212	-0.00476	-0.00686	-0.01057	-0.01942								
32	-0.00466	-0.00763	-0.01313	-0.02334									
16	-0.00419	-0.00930	-0.01868										
8	-0.01096	-0.01720											
4	-0.01780												

(e) $\mathcal{P}^{\mathcal{C}}$ for Scene: **[Cornell Box]** Algorithm: **[M-MLT]** Metric: **[IW-PSNR]** True GT: **[PT @ 32768 spp]**.

ERPT	2	4	8	16	32	64	128	256	512	1024	2048	4096	8192
GT (PT)													
16384	-0.00041	-0.00036	-0.00021	0.00008	-0.00006	-0.00014	-0.00002	-0.00010	-0.00018	-0.00008	0.00007	0.00007	0.00007
8192	-0.00112	-0.00080	-0.00098	-0.00039	-0.00081	-0.00044	-0.00038	-0.00092	-0.00098	-0.00064	-0.00075	-0.00101	
4096	-0.00063	-0.00161	-0.00085	-0.00067	-0.00103	-0.00177	-0.00102	-0.00109	-0.00061	-0.00095	-0.00061		
2048	-0.00225	-0.00207	-0.00241	-0.00225	-0.00215	-0.00264	-0.00192	-0.00182	-0.00161	-0.00233			
1024	-0.00448	-0.00304	-0.00432	-0.00363	-0.00363	-0.00375	-0.00403	-0.00343	-0.00467				
512	-0.00812	-0.00763	-0.00692	-0.00808	-0.00840	-0.00873	-0.00911	-0.00894					
256	-0.01703	-0.01616	-0.01542	-0.01370	-0.01658	-0.01655	-0.01768						
128	-0.03044	-0.02843	-0.02891	-0.03274	-0.02673	-0.03360							
64	-0.05626	-0.05226	-0.05417	-0.05681	-0.05225								
32	-0.09936	-0.09296	-0.09774	-0.10075									
16	-0.15783	-0.15545	-0.16023										
8	-0.24401	-0.23956											
4	-0.34097												

(f) $\mathcal{P}^{\mathcal{C}}$ for Scene: **[Cornell Box]** Algorithm: **[ERPT]** Metric: **[IW-PSNR]** True GT: **[PT @ 32768 spp]**.

M-ERPT	2	4	8	16	32	64	128	256	512	1024	2048	4096	8192
GT (PT)													
16384	-0.00059	-0.00028	-0.00019	-0.00037	-0.00033	-0.00026	-0.00033	-0.00021	-0.00032	-0.00050	-0.00005	-0.00038	-0.00028
8192	-0.00097	-0.00090	-0.00052	-0.00080	-0.00092	-0.00050	-0.00066	-0.00067	-0.00031	-0.00038	-0.00059	-0.00094	
4096	-0.00157	-0.00079	-0.00111	-0.00103	-0.00109	-0.00098	-0.00073	-0.00090	-0.00145	-0.00075	-0.00133		
2048	-0.00311	-0.00216	-0.00210	-0.00259	-0.00236	-0.00188	-0.00234	-0.00215	-0.00079	-0.00222			
1024	-0.00400	-0.00446	-0.00382	-0.00306	-0.00425	-0.00377	-0.00325	-0.00417	-0.00260				
512	-0.00774	-0.00647	-0.00619	-0.00802	-0.00737	-0.00837	-0.00749	-0.00811					
256	-0.01541	-0.01792	-0.01550	-0.01471	-0.01680	-0.01413	-0.01588						
128	-0.02768	-0.03026	-0.02878	-0.02995	-0.03454	-0.03007							
64	-0.05642	-0.05262	-0.05068	-0.05049	-0.05783								
32	-0.09138	-0.10148	-0.09000	-0.09330									
16	-0.15132	-0.16500	-0.14364										
8	-0.23709	-0.24726											
4	-0.33832												

(g) $\mathcal{P}^{\mathcal{C}}$ for Scene: **[Cornell Box]** Algorithm: **[M-ERPT]** Metric: **[IW-PSNR]** True GT: **[PT @ 32768 spp]**.

Table 81

18.2 Torus

PT	2	4	8	16	32	64	128	256	512	1024	2048	4096	8192	16384	32768	65536	131072
GT (BDPT)																	
262144	0.00046	0.00077	0.00023	0.00064	0.00095	0.00022	0.00086	0.00095	0.00094	0.00091	0.00135	0.00095	0.00019	-0.00107	-0.00254	-0.00620	-0.01385
131072	0.00096	0.00050	0.00056	0.00049	0.00211	0.00077	0.00117	0.00060	0.00070	0.00139	0.00270	-0.00107	-0.00052	-0.00910	-0.01727	-0.02029	
65536	0.00050	0.00019	0.00069	0.00112	0.00165	0.00178	0.00155	0.00203	0.00209	0.00189	0.00154	-0.00147	-0.00559	-0.01557	-0.02821		
32768	-0.00062	-0.00041	-0.00068	0.00024	0.00159	0.00291	0.00223	0.00157	0.00053	0.00064	0.00000	-0.00880	-0.02111	-0.03921			
16384	-0.00030	-0.00056	-0.00077	0.00064	0.00270	0.00158	0.00232	-0.00003	-0.00038	-0.00523	-0.00399	-0.02137	-0.03504				
8192	-0.00444	-0.00368	-0.00348	-0.00194	-0.00077	0.00048	0.00235	-0.00059	-0.00071	-0.00469	-0.00752	-0.03964					
4096	-0.00982	-0.00976	-0.01064	-0.00907	-0.00023	-0.00397	-0.00386	-0.00423	-0.00639	-0.01551	-0.02731						
2048	-0.01557	-0.01415	-0.01982	-0.01542	-0.00840	-0.00803	-0.01017	-0.01217	-0.01516	-0.02954							
1024	-0.01430	-0.01588	-0.02092	-0.01433	-0.00443	-0.00640	-0.00958	-0.01695	-0.02691								
512	-0.02032	-0.02182	-0.02874	-0.02270	-0.01197	-0.01496	-0.01491	-0.01243									
256	-0.00997	-0.00938	-0.02312	-0.00757	0.00501	-0.00158	-0.00062										
128	0.02011	0.02316	0.01162	0.02558	0.03532	0.03847											
64	0.04817	0.04612	0.04118	0.05405	0.06407												
32	0.08762	0.08852	0.08340	0.08841													
16	0.12741	0.13644	0.13404														
8	0.15238	0.14799															
4	0.18467																

(a) $\mathcal{P}^{\mathcal{C}}$ for Scene: [**Torus**] Algorithm: [**PT**] Metric: [**IW-PSNR**] True GT: [**BDPT @ 524288 spp**].

BDPT	2	4	8	16	32	64	128	256	512	1024	2048	4096	8192	16384	32768	65536	131072
GT (BDPT)																	
262144	0.00070	0.00074	0.00081	0.00042	0.00028	0.00092	0.00077	0.00062	0.00078	0.00119	-0.00022	0.00158	-0.00111	-0.00103	0.00114	-0.00462	-0.01172
131072	0.00082	0.00087	0.00113	0.00068	0.00163	0.00130	0.00106	0.00154	0.00170	0.00082	0.00031	0.00121	-0.00264	-0.00310	-0.00873	-0.02066	
65536	0.00077	0.00055	0.00117	0.00131	0.00132	0.00172	0.00201	0.00162	0.00163	0.00216	0.00006	0.00029	-0.00459	-0.01769	-0.02068		
32768	0.00017	-0.00182	-0.00027	-0.00084	0.00174	0.00144	0.00116	0.00130	0.00184	-0.00134	-0.00343	-0.00827	-0.01827	-0.03724			
16384	0.00058	-0.00212	0.00022	-0.00041	0.00175	0.00217	0.00157	0.00034	0.00038	-0.00317	-0.00891	-0.01692	-0.03210				
8192	-0.00187	-0.00541	-0.00145	-0.00102	0.00283	0.00094	-0.00046	0.00297	-0.00083	-0.00484	-0.01133	-0.02850					
4096	-0.00600	-0.01141	-0.00708	-0.01046	-0.00367	-0.00377	-0.00357	-0.00609	-0.00549	-0.01018	-0.03040						
2048	-0.01418	-0.02054	-0.01260	-0.01224	-0.00822	-0.00769	-0.00354	-0.00516	-0.01489	-0.02453							
1024	-0.01206	-0.02001	-0.01449	-0.01021	-0.00813	-0.00263	-0.01322	-0.00890	-0.02124								
512	-0.01572	-0.02843	-0.02436	-0.01943	-0.01408	-0.01066	-0.01411	-0.00450									
256	-0.00425	-0.01724	-0.00832	-0.00651	0.00355	-0.00372	0.00806										
128	0.02754	0.01287	0.01991	0.03075	0.04544	0.03641											
64	0.05092	0.04279	0.05139	0.05486	0.07153												
32	0.09143	0.09072	0.09218	0.09920													
16	0.13891	0.13370	0.12883														
8	0.15663	0.15535															
4	0.19346																

(b) $\mathcal{P}^{\mathcal{C}}$ for Scene: [**Torus**] Algorithm: [**BDPT**] Metric: [**IW-PSNR**] True GT: [**BDPT @ 524288 spp**].

PSSMLT	2	4	8	16	32	64	128	256	512	1024	2048	4096	8192	16384
GT (BDPT)														
262144	0.00034	0.00047	0.00022	0.00049	0.00074	0.00119	0.00108	0.00095	0.00120	0.00065	0.00104	0.00123	0.00256	0.00111
131072	-0.00007	0.00069	0.00086	0.00106	0.00127	0.00146	0.00172	0.00102	0.00151	0.00082	0.00052	0.00070	0.00202	-0.00020
65536	0.00038	0.00042	-0.00036	0.00061	0.00038	0.00101	0.00135	0.00041	0.00232	0.00058	-0.00079	-0.00087	-0.00168	-0.00483
32768	0.00051	0.00014	-0.00006	0.00034	0.00041	0.00125	0.00153	-0.00060	-0.00058	-0.00353	-0.00438	-0.00583	-0.00750	-0.01267
16384	-0.00115	-0.00186	-0.00140	-0.00291	-0.00254	-0.00259	-0.00376	-0.00633	-0.00541	-0.01131	-0.01612	-0.01684	-0.02403	
8192	-0.00186	-0.00141	-0.00239	-0.00075	-0.00306	-0.00345	-0.00488	-0.01141	-0.01627	-0.02503	-0.03825	-0.03977		
4096	-0.00295	-0.00528	-0.00376	-0.00141	-0.00606	-0.00838	-0.01410	-0.02286	-0.03138	-0.04418	-0.05991			
2048	-0.01393	-0.01545	-0.01768	-0.01863	-0.02186	-0.02495	-0.03226	-0.04946	-0.06042	-0.08728				
1024	-0.01622	-0.02037	-0.02222	-0.02346	-0.02852	-0.03219	-0.05185	-0.07175	-0.10376					
512	-0.01866	-0.02295	-0.02370	-0.01940	-0.03344	-0.04367	-0.07239	-0.11480						
256	-0.01795	-0.02192	-0.02728	-0.02839	-0.04994	-0.07814	-0.12207							
128	0.00007	-0.00580	-0.01449	-0.01684	-0.06042	-0.10012								
64	0.00329	-0.00424	-0.01208	-0.01801	-0.07888									
32	0.02511	0.01081	-0.00188	-0.01160										
16	0.04611	0.02670	0.00677											
8	0.05799	0.03853												
4	0.07161													

(c) $\mathcal{P}^{\mathcal{C}}$ for Scene: [**Torus**] Algorithm: [**PSSMLT**] Metric: [**IW-PSNR**] True GT: [**BDPT @ 524288 spp**].

MLT	2	4	8	16	32	64	128	256	512	1024	2048	4096	8192	16384
GT (BDPT)														
262144	0.00039	0.00036	0.00014	0.00066	0.00046	0.00052	0.00048	0.00049	0.00075	0.00047	0.00062	0.00065	0.00073	0.00125
131072	0.00164	0.00050	0.00042	0.00053	0.00066	0.00083	0.00084	0.00104	0.00154	0.00158	0.00134	0.00170	0.00183	0.00177
65536	0.00400	0.00193	0.00164	0.00225	0.00157	0.00197	0.00164	0.00155	0.00175	0.00143	0.00132	0.00151	0.00171	0.00146
32768	0.00515	0.00369	0.00219	0.00264	0.00161	0.00292	0.00163	0.00070	0.00166	0.00172	0.00143	0.00180	0.00111	-0.00030
16384	0.00838	0.00543	0.00392	0.00345	0.00144	0.00324	0.00077	0.00152	0.00147	0.00058	0.00062	-0.00036	-0.00109	
8192	0.00743	0.00265	-0.00096	0.00022	-0.00231	-0.00214	-0.00711	-0.00786	-0.00789	-0.00927	-0.01103	-0.01174		
4096	0.00930	0.00054	-0.00256	-0.00706	-0.01212	-0.01015	-0.02107	-0.02224	-0.02414	-0.02683	-0.03024			
2048	0.01003	-0.00622	-0.01149	-0.01667	-0.02684	-0.02586	-0.03874	-0.04130	-0.04400	-0.04592				
1024	0.00195	-0.02222	-0.02178	-0.03324	-0.05025	-0.05240	-0.07017	-0.07232	-0.07505					
512	-0.00122	-0.02885	-0.03553	-0.04741	-0.07007	-0.07262	-0.09481	-0.10289						
256	0.00189	-0.04138	-0.04906	-0.06735	-0.09390	-0.10092	-0.12791							
128	0.01038	-0.03586	-0.04570	-0.06641	-0.09420	-0.10062								
64	0.01207	-0.04120	-0.04661	-0.07363	-0.11247									
32	0.03313	-0.02566	-0.03452	-0.06566										
16	0.05198	-0.00769	-0.01820											
8	0.06219	0.00295												
4	0.07919													

(d) $\mathcal{P}^{\mathcal{C}}$ for Scene: [**Torus**] Algorithm: [**MLT**] Metric: [**IW-PSNR**] True GT: [**BDPT @ 524288 spp**].

M-MLT	2	4	8	16	32	64	128	256	512	1024	2048	4096	8192	16384
GT (BDPT)														
262144	0.00027	0.00001	0.00034	0.00064	0.00066	0.00093	0.00031	-0.00009	0.00032	0.00019	-0.00011	-0.00003	0.00022	0.00054
131072	0.00092	0.00078	0.00036	0.00149	0.00093	0.00044	0.00023	0.00091	0.00071	0.00019	0.00028	0.00019	0.00038	0.00056
65536	0.00265	0.00146	0.00130	0.00244	0.00141	0.00193	0.00100	0.00053	0.00078	0.00062	-0.00027	0.00032	-0.00008	-0.00044
32768	0.00560	0.00291	0.00069	0.00272	0.00089	0.00181	-0.00045	0.00026	-0.00038	-0.00030	-0.00121	-0.00207	-0.00138	-0.00361
16384	0.00789	0.00387	0.00167	0.00583	0.00109	0.00145	-0.00101	-0.00260	-0.00360	-0.00380	-0.00652	-0.00738	-0.00786	
8192	0.00659	0.00153	-0.00056	0.00251	-0.00480	-0.00624	-0.00835	-0.01171	-0.01745	-0.01629	-0.02247	-0.02360		
4096	0.00387	-0.00090	-0.00907	-0.00048	-0.01186	-0.01754	-0.02274	-0.02864	-0.03585	-0.03814	-0.04614			
2048	0.00033	-0.00507	-0.01813	-0.01169	-0.03236	-0.03323	-0.04156	-0.05210	-0.06430	-0.06698				
1024	-0.00950	-0.01734	-0.03556	-0.02738	-0.04809	-0.06036	-0.07552	-0.08663	-0.10346					
512	-0.01547	-0.02516	-0.05708	-0.04108	-0.06483	-0.08119	-0.10063	-0.11768						
256	-0.02016	-0.03358	-0.07254	-0.05518	-0.09054	-0.11036	-0.13855							
128	-0.01277	-0.02665	-0.07802	-0.05360	-0.08960	-0.11748								
64	-0.01387	-0.02772	-0.09512	-0.06826	-0.10974									
32	0.00449	-0.01817	-0.09211	-0.05771										
16	0.01886	-0.00884	-0.07960											
8	0.03426	0.00466												
4	0.04884													

(e) $\mathcal{P}^{\mathcal{C}}$ for Scene: [**Torus**] Algorithm: [**M-MLT**] Metric: [**IW-PSNR**] True GT: [**BDPT @ 524288 spp**].

ERPT	2	4	8	16	32	64	128	256	512	1024	2048	4096	8192	16384	32768	65536	131072
GT (BDPT)																	
262144	0.00082	0.00090	0.00078	0.00044	-0.00074	0.00005	0.00003	0.00070	-0.00001	0.00134	0.00107	0.00068	0.00075	0.00213	0.00123	0.00151	0.00110
131072	0.00095	0.00044	0.00110	0.00007	-0.00017	-0.00105	-0.00062	-0.00049	-0.00171	-0.00138	-0.00105	-0.00315	-0.00233	-0.00218	0.00007	-0.00058	
65536	0.00057	-0.00009	0.00026	-0.00029	-0.00184	0.00016	-0.00192	-0.00206	-0.00224	-0.00265	-0.00405	-0.00534	-0.00582	-0.00465	-0.00320		
32768	-0.00013	0.00025	0.00196	-0.00180	-0.00615	-0.00296	-0.00311	-0.00605	-0.00613	-0.00997	-0.01162	-0.01256	-0.01447	-0.01048			
16384	-0.00385	-0.00386	-0.00263	-0.00468	-0.00688	-0.00710	-0.00931	-0.01295	-0.01131	-0.01989	-0.02814	-0.03262	-0.03293				
8192	-0.00862	-0.01283	-0.01070	-0.01342	-0.01813	-0.01853	-0.02844	-0.03237	-0.03487	-0.04866	-0.05808	-0.06340					
4096	-0.02100	-0.02087	-0.02361	-0.02871	-0.04086	-0.04181	-0.05245	-0.06498	-0.06868	-0.08649	-0.09340						
2048	-0.03498	-0.04636	-0.05426	-0.05901	-0.07196	-0.07333	-0.09606	-0.11900	-0.12295	-0.15057							
1024	-0.05895	-0.08096	-0.07312	-0.09690	-0.11932	-0.11884	-0.15826	-0.18595	-0.19938								
512	-0.07261	-0.10925	-0.11179	-0.13919	-0.16058	-0.17193	-0.20269	-0.24629									
256	-0.09928	-0.15118	-0.15999	-0.17339	-0.20615	-0.22610	-0.26796										
128	-0.10378	-0.16046	-0.17137	-0.19931	-0.23111	-0.26041											
64	-0.12207	-0.18789	-0.20252	-0.22246	-0.25151												
32	-0.12797	-0.19157	-0.20368	-0.22296													
16	-0.10980	-0.18144	-0.19604														
8	-0.09984	-0.17277															
4	-0.08816																

(f) $\mathcal{P}^{\mathcal{C}}$ for Scene: [**Torus**] Algorithm: [**ERPT**] Metric: [**IW-PSNR**] True GT: [**BDPT @ 524288 spp**].

M-ERPT	2	4	8	16	32	64	128	256	512	1024	2048	4096	8192	16384	32768	65536	131072
GT (BDPT)																	
262144	-0.00004	0.00010	0.00045	0.00006	0.00041	0.00068	-0.00053	-0.00002	0.00017	0.00012	0.00106	0.00029	0.00041	-0.00026	0.00060	0.00043	-0.00023
131072	0.00034	-0.00002	0.00073	0.00131	0.00116	0.00179	-0.00071	-0.00066	-0.00120	-0.00045	-0.00250	-0.00141	-0.00110	-0.00095	-0.00090	-0.00160	
65536	0.00114	0.00090	-0.00033	0.00009	-0.00153	0.00049	-0.00161	-0.00385	-0.00509	-0.00452	-0.00512	-0.00334	-0.00357	-0.00471	-0.00404		
32768	-0.00065	-0.00105	0.00041	-0.00077	-0.00206	-0.00514	-0.00700	-0.00989	-0.00963	-0.01014	-0.01457	-0.01234	-0.01067	-0.01358			
16384	-0.00228	-0.00275	-0.00459	-0.00391	-0.00517	-0.00911	-0.01236	-0.01488	-0.02124	-0.02371	-0.03095	-0.03032	-0.02657				
8192	-0.00870	-0.00889	-0.01383	-0.01373	-0.01789	-0.02234	-0.03222	-0.03620	-0.05002	-0.05243	-0.06411	-0.05866					
4096	-0.02197	-0.02255	-0.02842	-0.03289	-0.03441	-0.04707	-0.05957	-0.06577	-0.08271	-0.08882	-0.10510						
2048	-0.04160	-0.03650	-0.05219	-0.05742	-0.06321	-0.08280	-0.10793	-0.11493	-0.14320	-0.15167							
1024	-0.07454	-0.06449	-0.08382	-0.09380	-0.11118	-0.13671	-0.17082	-0.18056	-0.21914								
512	-0.09626	-0.09185	-0.10502	-0.12781	-0.14613	-0.18072	-0.21823	-0.24155									
256	-0.12436	-0.11268	-0.14101	-0.16163	-0.19042	-0.23620	-0.28014										
128	-0.12929	-0.11612	-0.15484	-0.18273	-0.21584	-0.27117											
64	-0.14594	-0.12952	-0.16286	-0.20545	-0.23344												
32	-0.13724	-0.12437	-0.16666	-0.19835													
16	-0.11228	-0.11001	-0.15349														
8	-0.09764	-0.10297															
4	-0.08610																

(g) $\mathcal{P}^{\mathcal{C}}$ for Scene: [**Torus**] Algorithm: [**M-ERPT**] Metric: [**IW-PSNR**] True GT: [**BDPT @ 524288 spp**].

Table 82

18.3 Veach Bidir

PT	2	4	8	16	32	64	128	256	512	1024	2048	4096	8192	16384	32768	65536	131072
GT (BDPT)																	
262144	0.00010	0.00010	0.00013	0.00017	0.00003	0.00011	0.00018	0.00005	0.00024	0.00042	0.00044	0.00059	0.00053	0.00025	0.00084	0.00057	0.00110
131072	0.00010	0.00021	0.00006	0.00009	0.00009	0.00019	0.00010	0.00006	0.00022	0.00027	0.00039	0.00031	0.00019	0.00079	0.00091	0.00032	
65536	0.00008	0.00014	0.00014	0.00031	0.00016	0.00023	0.00045	-0.00021	0.00008	0.00044	0.00084	0.00087	0.00044	0.00054	0.00045		
32768	0.00005	0.00021	0.00024	0.00028	0.00027	0.00033	0.00027	-0.00005	-0.00005	0.00004	0.00044	0.00038	0.00006	-0.00044			
16384	0.00043	0.00093	0.00123	0.00136	0.00160	0.00193	0.00146	0.00181	0.00227	0.00135	0.00133	0.00032	-0.00039				
8192	0.00044	0.00053	0.00112	0.00134	0.00111	0.00195	0.00134	0.00184	0.00189	0.00100	0.00078	-0.00096					
4096	-0.00023	0.00006	-0.00016	0.00007	-0.00022	0.00018	-0.00051	-0.00114	-0.00016	-0.00012	0.00012						
2048	0.00064	0.00084	0.00177	0.00200	0.00305	0.00399	0.00202	0.00312	0.00286	0.00015							
1024	0.00223	0.00356	0.00638	0.00757	0.00755	0.01197	0.00682	0.00904	0.01069								
512	0.00346	0.00544	0.00908	0.01067	0.00933	0.01818	0.00747	0.00980									
256	0.00335	0.00478	0.00897	0.01040	0.00694	0.01850	0.00571										
128	0.00434	0.00648	0.01356	0.01300	0.00643	0.02320											
64	0.00570	0.00751	0.01326	0.01437	0.00659												
32	0.00647	0.00676	0.01109	0.01325													
16	0.00492	0.00603	0.00869														
8	0.00455	0.00367															
4	0.00093																

(a) $\mathcal{P}^{\mathcal{C}}$ for Scene: [**Veach Bidir**] Algorithm: [**PT**] Metric: [**IW-PSNR**] True GT: [**BDPT @ 524288 spp**].

BDPT	2	4	8	16	32	64	128	256	512	1024	2048	4096	8192	16384	32768	65536	131072
GT (BDPT)																	
262144	0.00025	0.00023	0.00039	0.00058	0.00032	0.00032	0.00065	0.00005	0.00028	0.00367	-0.00481	-0.00385	0.00525	0.00838	0.00169	0.00105	-0.00357
131072	0.00036	0.00043	0.00034	0.00021	-0.00014	-0.00005	-0.00015	0.00019	-0.00106	-0.00208	-0.00514	0.01105	0.00154	-0.00015	-0.00153	0.00929	
65536	0.00044	0.00057	0.00051	0.00115	0.00003	-0.00017	-0.00030	0.00030	0.00093	-0.00059	-0.00764	0.01895	-0.00119	-0.01941	-0.00874		
32768	0.00039	0.00034	0.00027	0.00082	-0.00036	-0.00114	-0.00103	-0.00051	0.00253	0.00079	0.00194	-0.01442	-0.02062	-0.00242			
16384	0.00307	0.00417	0.00530	0.00538	0.00770	0.00755	0.01065	0.01238	0.01395	0.02438	0.02540	-0.07197	-0.04089				
8192	0.00311	0.00449	0.00645	0.00757	0.00745	0.00791	0.00973	0.01235	0.00972	0.01189	-0.00831	-0.05725					
4096	0.00076	0.00062	0.00021	0.00113	-0.00107	-0.00178	-0.00314	-0.00195	-0.00536	-0.01298	-0.04924						
2048	0.00522	0.00856	0.01059	0.01171	0.01557	0.01506	0.01938	0.02037	0.03466	0.01859							
1024	0.01742	0.02525	0.03629	0.04630	0.04807	0.05697	0.07170	0.09797	0.09944								
512	0.02814	0.04128	0.05949	0.07610	0.07826	0.10095	0.11377	0.11677									
256	0.02890	0.04395	0.06640	0.08353	0.09167	0.11697	0.12837										
128	0.03873	0.05504	0.08788	0.07482	0.12008	0.19297											
64	0.04330	0.06842	0.09533	0.07912	0.12647												
32	0.04226	0.06757	0.07974	0.05516													
16	0.03107	0.04838	0.06073														
8	0.02338	0.03380															
4	0.00895																

(b) $\mathcal{P}^{\mathcal{C}}$ for Scene: [**Veach Bidir**] Algorithm: [**BDPT**] Metric: [**IW-PSNR**] True GT: [**BDPT @ 524288 spp**].

PSSMLT	2	4	8	16	32	64	128	256	512	1024	2048	4096	8192	16384
GT (BDPT)														
262144	0.00022	0.00032	0.00036	0.00035	0.00046	0.00028	0.00028	0.00022	0.00221	0.00177	0.00426	0.00565	0.00322	0.00118
131072	0.00036	0.00027	0.00022	0.00011	0.00006	-0.00005	-0.00004	-0.00001	-0.00126	-0.00087	0.00076	0.00020	0.00023	-0.00003
65536	0.00034	0.00034	0.00017	0.00026	0.00053	0.00038	0.00046	-0.00130	0.00288	0.00278	0.00126	0.00116	-0.00005	-0.00220
32768	0.00042	0.00042	0.00028	0.00018	-0.00005	0.00008	0.00007	-0.00045	0.00298	0.00128	0.00377	-0.00219	0.00043	-0.00106
16384	0.00166	0.00285	0.00390	0.00485	0.00529	0.00649	0.00666	0.01366	0.01214	0.01051	0.01071	0.01057	0.00817	
8192	0.00181	0.00295	0.00404	0.00577	0.00686	0.00739	0.00833	0.01483	0.01980	0.01351	-0.00007	0.00797		
4096	0.00001	-0.00033	-0.00057	-0.00089	-0.00071	-0.00155	-0.00210	-0.00801	-0.01341	-0.00678	-0.02454			
2048	0.00324	0.00522	0.00739	0.01004	0.01079	0.01434	0.01596	0.02387	0.00444	0.00523				
1024	0.00926	0.01553	0.02385	0.03465	0.04244	0.05039	0.05588	0.08811	0.05288					
512	0.01353	0.02329	0.03731	0.05618	0.06987	0.08944	0.10068	0.11621						
256	0.01570	0.02662	0.04138	0.06396	0.07965	0.10168	0.11765							
128	0.01934	0.03256	0.05166	0.08175	0.10745	0.14396								
64	0.02078	0.03634	0.06199	0.09174	0.11865									
32	0.01728	0.02934	0.05400	0.07239										
16	0.01136	0.01743	0.03036											
8	0.00496	0.00447												
4	-0.00856													

(c) $\mathcal{P}^{\mathcal{C}}$ for Scene: [**Veach Bidir**] Algorithm: [**PSSMLT**] Metric: [**IW-PSNR**] True GT: [**BDPT @ 524288 spp**].

MLT	2	4	8	16	32	64	128	256	512	1024	2048	4096	8192	16384
GT (BDPT)														
262144	-0.00004	-0.00002	-0.00002	-0.00003	0.00012	0.00015	0.00012	0.00008	0.00014	0.00009	0.00057	0.00068	0.00053	0.00054
131072	0.00008	0.00013	0.00013	0.00015	0.00028	0.00042	0.00029	0.00010	-0.00006	-0.00027	-0.00025	-0.00011	-0.00013	-0.00013
65536	0.00012	0.00012	0.00009	0.00029	0.00048	0.00064	0.00045	0.00047	0.00044	0.00035	0.00072	0.00079	0.00082	0.00078
32768	-0.00012	-0.00012	-0.00017	0.00003	0.00022	0.00040	0.00013	-0.00050	-0.00017	-0.00026	0.00017	-0.00004	0.00021	0.00034
16384	0.00008	0.00043	0.00070	0.00145	0.00238	0.00346	0.00466	0.00485	0.00618	0.00639	0.00610	0.00737	0.00772	
8192	0.00014	0.00045	0.00104	0.00198	0.00274	0.00393	0.00570	0.00660	0.00757	0.00798	0.00902	0.00964		
4096	-0.00020	-0.00013	-0.00025	-0.00011	0.00050	0.00046	0.00005	0.00009	-0.00068	-0.00177	-0.00236			
2048	0.00078	0.00156	0.00278	0.00368	0.00593	0.00804	0.01179	0.01224	0.01489	0.01697				
1024	0.00204	0.00400	0.00642	0.00999	0.01476	0.02315	0.03489	0.04258	0.05197					
512	0.00427	0.00684	0.01071	0.01506	0.02185	0.03769	0.05527	0.06953						
256	0.00472	0.00780	0.01234	0.01779	0.02671	0.04391	0.06723							
128	0.00601	0.00983	0.01407	0.01956	0.03119	0.05477								
64	0.00871	0.01210	0.01796	0.02362	0.03298									
32	0.00859	0.01268	0.01906	0.02222										
16	0.00741	0.00936	0.01319											
8	0.00937	0.01118												
4	0.00878													

(d) $\mathcal{P}^{\mathcal{C}}$ for Scene: [**Veach Bidir**] Algorithm: [**MLT**] Metric: [**IW-PSNR**] True GT: [**BDPT @ 524288 spp**].

M-MLT	2	4	8	16	32	64	128	256	512	1024	2048	4096	8192	16384
GT (BDPT)														
262144	0.00004	0.00010	0.00012	0.00011	0.00027	0.00021	0.00014	0.00023	0.00024	0.00016	0.00027	0.00028	0.00031	0.00053
131072	0.00013	0.00016	0.00021	0.00018	0.00038	0.00013	0.00008	0.00002	-0.00003	-0.00033	-0.00042	-0.00016	-0.00022	-0.00026
65536	0.00003	0.00011	0.00010	0.00004	0.00053	0.00029	0.00066	0.00057	0.00050	-0.00001	0.00002	0.00042	0.00036	0.00059
32768	-0.00005	-0.00002	-0.00002	-0.00017	0.00015	0.00003	0.00010	-0.00001	-0.00027	-0.00095	-0.00003	-0.00026	-0.00007	0.00015
16384	0.00017	0.00048	0.00038	0.00150	0.00171	0.00261	0.00330	0.00504	0.00517	0.00663	0.00703	0.00752	0.00812	
8192	0.00043	0.00100	0.00125	0.00216	0.00310	0.00446	0.00528	0.00672	0.00787	0.00789	0.00817	0.00935		
4096	-0.00027	-0.00036	-0.00015	-0.00049	-0.00022	-0.00006	-0.00091	-0.00053	-0.00151	-0.00241	-0.00209			
2048	0.00036	0.00092	0.00133	0.00332	0.00451	0.00617	0.00825	0.01289	0.01389	0.01600				
1024	0.00250	0.00462	0.00521	0.01072	0.01473	0.02268	0.02941	0.04366	0.04847					
512	0.00433	0.00798	0.00969	0.01773	0.02357	0.03547	0.04627	0.07261						
256	0.00497	0.00823	0.01138	0.01963	0.02763	0.04259	0.05683							
128	0.00722	0.01186	0.01523	0.02640	0.03322	0.05306								
64	0.00973	0.01518	0.01803	0.03214	0.03974									
32	0.01108	0.01574	0.01958	0.03204										
16	0.00895	0.01356	0.01386											
8	0.01080	0.01481												
4	0.01037													

(e) $\mathcal{P}^{\mathcal{C}}$ for Scene: [**Veach Bidir**] Algorithm: [**M-MLT**] Metric: [**IW-PSNR**] True GT: [**BDPT @ 524288 spp**].

ERPT	2	4	8	16	32	64	128	256	512	1024	2048	4096	8192	16384	32768	65536	131072
GT (BDPT)																	
262144	0.00022	0.00021	0.00036	0.00033	0.00040	0.00037	0.00072	0.00024	0.00055	0.00016	0.00010	0.00012	0.00026	-0.00016	0.00024	0.00035	0.00036
131072	-0.00017	0.00014	-0.00003	0.00008	0.00016	0.00059	0.00028	0.00028	0.00015	0.00072	0.00040	0.00041	0.00040	0.00019	0.00052	0.00060	
65536	-0.00005	0.00028	0.00006	0.00026	-0.00018	0.00135	0.00025	0.00028	-0.00015	-0.00002	-0.00014	0.00032	-0.00018	-0.00012	0.00007		
32768	-0.00018	-0.00016	-0.00010	0.00022	0.00015	-0.00051	0.00002	0.00012	-0.00056	0.00014	-0.00070	-0.00008	-0.00029	-0.00012			
16384	0.00402	0.00473	0.00510	0.00485	0.00636	0.00020	0.00466	-0.00006	-0.00075	-0.00041	-0.00081	-0.00243	-0.00074				
8192	0.00599	0.00576	0.00577	0.00548	0.00599	-0.00009	0.00462	0.00393	0.00030	-0.00115	-0.00015	-0.00136					
4096	-0.00166	-0.00035	-0.00103	-0.00017	-0.00190	0.00042	-0.00003	0.00086	-0.00490	-0.00220	-0.00236						
2048	0.00957	0.01068	0.01195	0.01127	0.01357	0.00100	0.00869	-0.00044	-0.00485	-0.00333							
1024	0.03479	0.03607	0.03838	0.03716	0.03668	-0.00369	0.03114	0.00452	-0.01030								
512	0.05776	0.06064	0.06306	0.06026	0.05210	-0.02084	0.03611	-0.00805									
256	0.06762	0.07105	0.07336	0.07024	0.05865	-0.02183	0.03728										
128	0.09449	0.09426	0.09933	0.09910	0.06782	-0.06764											
64	0.10285	0.10618	0.10209	0.10010	0.05791												
32	0.08793	0.07923	0.07718	0.07109													
16	0.04444	0.05226	0.04547														
8	0.01379	0.01480															
4	-0.05409																

(f) $\mathcal{P}^{\mathcal{C}}$ for Scene: [**Veach Bidir**] Algorithm: [**ERPT**] Metric: [**IW-PSNR**] True GT: [**BDPT @ 524288 spp**].

M-ERPT	2	4	8	16	32	64	128	256	512	1024	2048	4096	8192	16384	32768	65536	131072
GT (BDPT)																	
262144	-0.00003	0.00038	0.00032	0.00070	0.00018	0.00011	-0.00059	0.00036	-0.00024	0.00015	0.00004	0.00023	0.00024	0.00016	0.00028	0.00010	0.00026
131072	-0.00006	0.00008	0.00035	-0.00030	-0.00008	0.00017	0.00005	0.00019	-0.00015	0.00023	0.00033	0.00048	0.00050	0.00027	0.00072	0.00044	
65536	-0.00012	0.00029	0.00040	0.00025	0.00008	0.00013	-0.00064	0.00008	-0.00038	0.00046	-0.00015	0.00012	0.00004	0.00004	0.00050		
32768	-0.00009	-0.00011	0.00003	0.00045	-0.00015	-0.00041	-0.00043	0.00022	0.00037	-0.00005	-0.00038	-0.00085	0.00001	-0.00001			
16384	0.00435	0.00442	0.00087	0.00139	0.00280	-0.00019	-0.00058	-0.00087	0.00012	-0.00266	-0.00147	-0.00206	-0.00168				
8192	0.00518	0.00571	0.00088	0.00139	0.00370	0.00100	-0.00404	0.00109	-0.00170	-0.00114	-0.00241	-0.00175					
4096	-0.00134	-0.00023	-0.00403	-0.00445	-0.00171	-0.00402	-0.00362	-0.00402	-0.00425	-0.00355	-0.00314						
2048	0.01215	0.01061	-0.00165	-0.00348	0.00327	-0.00319	-0.00008	-0.00404	0.00080	-0.00722							
1024	0.03376	0.03798	-0.00985	0.00060	0.01233	-0.01240	-0.01757	-0.01377	-0.01175								
512	0.05567	0.05805	-0.03816	-0.01511	-0.00130	-0.04101	-0.03592	-0.04161									
256	0.06653	0.07301	-0.03859	-0.02433	-0.00310	-0.04355	-0.03981										
128	0.09041	0.10020	-0.08687	-0.06377	-0.04358	-0.08996											
64	0.09722	0.10539	-0.10829	-0.08475	-0.06941												
32	0.08133	0.08007	-0.10343	-0.08123													
16	0.04230	0.05235	-0.11230														
8	0.01647	0.02211															
4	-0.04779																

(g) $\mathcal{P}^{\mathcal{C}}$ for Scene: [**Veach Bidir**] Algorithm: [**M-ERPT**] Metric: [**IW-PSNR**] True GT: [**BDPT @ 524288 spp**].

Table 83

18.4 Veach Door

PT	2	4	8	16	32	64	128	256	512	1024	2048	4096	8192	16384	32768	65536	131072
GT (BDPT)																	
262144	-0.00001	-0.00007	-0.00002	-0.00010	-0.00018	-0.00001	-0.00006	-0.00004	-0.00011	-0.00012	-0.00005	-0.00020	-0.00022	-0.00052	-0.00030	-0.00265	-0.00264
131072	-0.00008	-0.00029	-0.00012	-0.00020	-0.00029	-0.00017	-0.00033	-0.00052	-0.00048	-0.00049	-0.00066	-0.00094	-0.00096	-0.00190	-0.00376	-0.00447	
65536	-0.00031	-0.00053	-0.00043	-0.00036	-0.00041	-0.00035	-0.00044	-0.00020	-0.00065	-0.00063	-0.00119	-0.00121	-0.00116	-0.00317	-0.00518		
32768	-0.00079	-0.00108	-0.00078	-0.00062	-0.00053	-0.00050	-0.00059	-0.00060	-0.00056	-0.00081	-0.00115	-0.00187	-0.00289	-0.00602			
16384	-0.00157	-0.00171	-0.00126	-0.00081	-0.00012	0.00027	-0.00016	0.00021	0.00032	-0.00044	-0.00087	-0.00157	-0.00384				
8192	-0.00261	-0.00285	-0.00166	-0.00070	-0.00016	0.00051	0.00073	0.00068	0.00076	-0.00019	-0.00037	-0.00328					
4096	-0.00378	-0.00380	-0.00171	-0.00097	-0.00014	0.00088	0.00059	0.00075	-0.00003	-0.00211	-0.00283						
2048	-0.00637	-0.00570	-0.00234	-0.00059	0.00131	0.00126	0.00205	0.00223	0.00097	-0.00206							
1024	-0.00947	-0.00817	-0.00230	0.00017	0.00187	0.00313	0.00235	0.00087	-0.00047								
512	-0.01282	-0.01209	-0.00255	0.00117	0.00436	0.00430	0.00506	0.00266									
256	-0.02022	-0.01609	-0.00383	0.00213	0.00372	0.00392	0.00138										
128	-0.02830	-0.02415	-0.00441	0.00233	0.00432	0.00085											
64	-0.04211	-0.03445	-0.00957	-0.00714	-0.00354												
32	-0.05923	-0.04943	-0.01656	-0.01135													
16	-0.08815	-0.06943	-0.04097														
8	-0.14452	-0.12747															
4	-0.22201																

(a) $\mathcal{P}^{\mathcal{C}}$ for Scene: [**Veach Door**] Algorithm: [**PT**] Metric: [**IW-PSNR**] True GT: [**BDPT @ 524288 spp**].

BDPT	2	4	8	16	32	64	128	256	512	1024	2048	4096	8192	16384	32768	65536	131072
GT (BDPT)																	
262144	0.00001	-0.00010	-0.00017	-0.00003	-0.00005	-0.00003	0.00001	-0.00005	-0.00036	0.00001	-0.00088	0.00009	-0.00250	-0.00173	-0.00479	-0.00825	-0.01253
131072	-0.00012	-0.00016	-0.00022	-0.00019	-0.00034	-0.00039	-0.00040	-0.00062	-0.00126	-0.00125	-0.00214	-0.00171	-0.00535	-0.00776	-0.01046	-0.01826	
65536	-0.00039	-0.00053	-0.00040	-0.00039	-0.00043	-0.00039	-0.00037	-0.00043	-0.00120	-0.00106	-0.00248	-0.00462	-0.01118	-0.01579	-0.02913		
32768	-0.00080	-0.00061	-0.00050	-0.00061	-0.00032	0.00005	-0.00028	-0.00064	-0.00168	-0.00229	-0.00607	-0.00727	-0.01963	-0.03045			
16384	-0.00125	-0.00082	-0.00052	0.00023	0.00034	0.00040	0.00043	0.00019	-0.00095	-0.00245	-0.00508	-0.01413	-0.02999				
8192	-0.00227	-0.00107	-0.00015	0.00068	0.00111	0.00133	0.00093	0.00011	-0.00242	-0.00607	-0.01316	-0.02952					
4096	-0.00242	-0.00141	-0.00022	0.00120	0.00120	0.00111	-0.00012	-0.00074	-0.00752	-0.01302	-0.02810						
2048	-0.00413	-0.00119	0.00097	0.00258	0.00348	0.00264	0.00073	-0.00241	-0.00833	-0.02271							
1024	-0.00588	-0.00103	0.00191	0.00357	0.00440	0.00320	-0.00055	-0.00690	-0.02048								
512	-0.00902	0.00099	0.00353	0.00618	0.00735	0.00262	-0.00202	-0.01581									
256	-0.01223	0.00015	0.00708	0.00787	0.00788	-0.00109	-0.01113										
128	-0.01848	-0.00004	0.00862	0.01030	0.00515	-0.01195											
64	-0.02558	-0.00079	0.00412	-0.00205	-0.01522												
32	-0.03709	-0.00695	-0.00348	-0.01938													
16	-0.06382	-0.03268	-0.02625														
8	-0.10537	-0.07803															
4	-0.18155																

(b) $\mathcal{P}^{\mathcal{C}}$ for Scene: [**Veach Door**] Algorithm: [**BDPT**] Metric: [**IW-PSNR**] True GT: [**BDPT @ 524288 spp**].

PSSMLT	2	4	8	16	32	64	128	256	512	1024	2048	4096	8192	16384
GT (BDPT)														
262144	-0.00006	-0.00009	-0.00001	0.00006	0.00010	-0.00001	-0.00006	-0.00034	-0.00035	-0.00068	-0.00063	-0.00099	-0.00210	-0.00444
131072	-0.00018	-0.00006	0.00001	0.00005	-0.00004	0.00002	-0.00006	-0.00023	-0.00038	-0.00100	-0.00159	-0.00307	-0.00581	-0.00951
65536	-0.00021	-0.00007	0.00014	0.00028	0.00024	0.00021	0.00025	0.00002	-0.00042	-0.00140	-0.00343	-0.00556	-0.00971	-0.01861
32768	-0.00039	0.00010	0.00052	0.00060	0.00052	0.00017	0.00001	-0.00042	-0.00159	-0.00331	-0.00622	-0.01247	-0.02117	-0.03394
16384	-0.00021	0.00057	0.00115	0.00137	0.00116	0.00096	0.00042	0.00011	-0.00163	-0.00391	-0.00985	-0.01941	-0.03415	
8192	-0.00040	0.00106	0.00196	0.00212	0.00196	0.00139	0.00135	-0.00044	-0.00343	-0.00862	-0.02179	-0.04022		
4096	-0.00026	0.00161	0.00284	0.00283	0.00188	0.00122	-0.00019	-0.00375	-0.01036	-0.02402	-0.04093			
2048	0.00023	0.00247	0.00391	0.00395	0.00278	0.00181	-0.00138	-0.00655	-0.01840	-0.03806				
1024	0.00017	0.00268	0.00419	0.00391	0.00097	-0.00131	-0.00806	-0.02128	-0.04390					
512	0.00076	0.00327	0.00483	0.00292	-0.00147	-0.00741	-0.02105	-0.04663						
256	-0.00220	0.00160	0.00274	-0.00211	-0.01110	-0.02460	-0.04982							
128	-0.00399	-0.00166	-0.00109	-0.01012	-0.02624	-0.05575								
64	-0.01328	-0.01330	-0.01760	-0.03613	-0.06734									
32	-0.02320	-0.03103	-0.04517	-0.07696										
16	-0.05435	-0.07016	-0.10838											
8	-0.11606	-0.15002												
4	-0.24595													

(c) $\mathcal{P}^{\mathcal{C}}$ for Scene: [**Veach Door**] Algorithm: [**PSSMLT**] Metric: [**IW-PSNR**] True GT: [**BDPT @ 524288 spp**].

MLT	2	4	8	16	32	64	128	256	512	1024	2048	4096	8192	16384
GT (BDPT)														
262144	-0.00010	0.00009	0.00008	0.00012	0.00015	0.00014	0.00017	-0.00010	0.00001	0.00043	-0.00032	-0.00058	-0.00132	-0.00137
131072	-0.00006	0.00008	0.00006	0.00017	0.00022	0.00021	0.00017	0.00011	0.00036	0.00002	-0.00094	-0.00137	-0.00035	-0.00455
65536	-0.00002	0.00032	0.00032	0.00044	0.00055	0.00038	0.00046	0.00040	0.00004	-0.00012	-0.00168	-0.00346	-0.00365	-0.00967
32768	0.00039	0.00085	0.00096	0.00138	0.00124	0.00123	0.00130	0.00127	0.00043	0.00025	-0.00315	-0.00819	-0.01404	-0.02269
16384	0.00083	0.00172	0.00204	0.00244	0.00258	0.00291	0.00270	0.00225	0.00096	-0.00032	-0.00831	-0.01486	-0.02496	
8192	0.00177	0.00282	0.00319	0.00376	0.00384	0.00436	0.00353	0.00211	-0.00039	-0.00750	-0.01806	-0.03199		
4096	0.00228	0.00368	0.00427	0.00548	0.00504	0.00462	0.00335	-0.00119	-0.00638	-0.01731	-0.03818			
2048	0.00400	0.00545	0.00658	0.00694	0.00656	0.00633	0.00280	-0.00171	-0.01564	-0.03337				
1024	0.00513	0.00671	0.00752	0.00840	0.00666	0.00504	-0.00051	-0.01407	-0.03360					
512	0.00652	0.00802	0.00918	0.00774	0.00559	0.00253	-0.01360	-0.04014						
256	0.00552	0.00661	0.00606	0.00441	0.00044	-0.00909	-0.03983							
128	0.00629	0.00287	0.00192	-0.00496	-0.01597	-0.03387								
64	-0.00319	-0.00820	-0.01597	-0.03182	-0.05402									
32	-0.01252	-0.02810	-0.04361	-0.07875										
16	-0.04055	-0.07363	-0.10349											
8	-0.10845	-0.16344												
4	-0.24610													

(d) $\mathcal{P}^{\mathcal{C}}$ for Scene: [**Veach Door**] Algorithm: [**MLT**] Metric: [**IW-PSNR**] True GT: [**BDPT @ 524288 spp**].

M-MLT	2	4	8	16	32	64	128	256	512	1024	2048	4096	8192	16384
GT (BDPT)														
262144	-0.00005	0.00004	0.00007	0.00011	0.00028	0.00007	0.00012	0.00011	0.00007	0.00039	-0.00019	-0.00029	-0.00016	-0.00145
131072	-0.00004	0.00019	0.00021	0.00013	0.00017	0.00004	-0.00016	0.00016	0.00006	0.00026	-0.00079	-0.00087	-0.00333	-0.00333
65536	0.00007	0.00043	0.00028	0.00038	0.00061	0.00019	0.00041	-0.00004	-0.00016	-0.00004	-0.00106	-0.00164	-0.00672	-0.00787
32768	0.00044	0.00099	0.00091	0.00106	0.00108	0.00108	0.00052	0.00110	0.00011	-0.00031	-0.00425	-0.00467	-0.01307	-0.01274
16384	0.00105	0.00180	0.00204	0.00197	0.00244	0.00216	0.00245	0.00164	0.00031	-0.00059	-0.00738	-0.00963	-0.02361	
8192	0.00181	0.00297	0.00353	0.00352	0.00383	0.00325	0.00357	0.00140	-0.00060	-0.00585	-0.01669	-0.02221		
4096	0.00218	0.00398	0.00461	0.00460	0.00491	0.00351	0.00070	-0.00233	-0.00571	-0.01613	-0.03474			
2048	0.00367	0.00577	0.00668	0.00664	0.00667	0.00464	0.00067	-0.00580	-0.01366	-0.03149				
1024	0.00477	0.00754	0.00755	0.00778	0.00589	0.00170	-0.00177	-0.01617	-0.03212					
512	0.00591	0.00930	0.00952	0.00856	0.00552	-0.00437	-0.01600	-0.03900						
256	0.00456	0.00828	0.00884	0.00508	-0.00090	-0.02051	-0.03827							
128	0.00278	0.00328	0.00403	-0.00943	-0.01516	-0.04886								
64	-0.00315	-0.00707	-0.01450	-0.03342	-0.05975									
32	-0.02022	-0.02523	-0.04276	-0.08833										
16	-0.05269	-0.06924	-0.10812											
8	-0.12226	-0.15133												
4	-0.25034													

(e) $\mathcal{P}^{\mathcal{C}}$ for Scene: [**Veach Door**] Algorithm: [**M-MLT**] Metric: [**IW-PSNR**] True GT: [**BDPT @ 524288 spp**].

ERPT	2	4	8	16	32	64	128	256	512	1024	2048	4096	8192	16384	32768	65536	131072
GT (BDPT)																	
262144	0.00024	0.00010	0.00020	0.00031	0.00010	0.00010	-0.00006	-0.00017	-0.00014	-0.00005	-0.00010	0.00008	-0.00014	0.00012	-0.00032	0.00001	0.00001
131072	0.00007	-0.00001	0.00005	-0.00011	-0.00018	0.00006	-0.00016	-0.00007	-0.00027	-0.00020	-0.00021	-0.00006	-0.00025	0.00013	0.00008	-0.00030	
65536	0.00049	0.00026	0.00025	0.00010	0.00022	0.00012	0.00031	-0.00002	-0.00019	0.00007	0.00007	-0.00001	0.00004	0.00015	0.00000		
32768	0.00088	0.00088	0.00033	0.00078	0.00048	0.00046	0.00016	0.00044	0.00028	0.00005	0.00023	0.00043	0.00043	0.00040			
16384	0.00254	0.00181	0.00174	0.00118	0.00091	0.00099	0.00117	0.00079	0.00188	0.00074	0.00107	0.00067	0.00172				
8192	0.00298	0.00378	0.00272	0.00171	0.00177	0.00153	0.00224	0.00022	0.00245	0.00034	0.00181	0.00149					
4096	0.00165	0.00067	0.00096	-0.00003	0.00013	-0.00060	-0.00025	-0.00196	0.00056	-0.00174	-0.00043						
2048	-0.00014	0.00080	-0.00050	-0.00375	-0.00343	-0.00433	-0.00253	-0.00425	-0.00229	-0.00435							
1024	-0.00554	-0.00658	-0.00816	-0.01228	-0.00946	-0.01609	-0.00892	-0.01397	-0.00819								
512	-0.02075	-0.01985	-0.02971	-0.03538	-0.03491	-0.03943	-0.03436	-0.03858									
256	-0.04947	-0.04586	-0.05853	-0.07425	-0.07496	-0.08306	-0.07339										
128	-0.09605	-0.10356	-0.12331	-0.14605	-0.14653	-0.14794											
64	-0.18059	-0.18229	-0.21443	-0.24012	-0.24088												
32	-0.28178	-0.28970	-0.33049	-0.35734													
16	-0.41702	-0.42635	-0.46429														
8	-0.60619	-0.62087															
4	-0.82505																

(f) $\mathcal{P}^{\mathcal{C}}$ for Scene: [**Veach Door**] Algorithm: [**ERPT**] Metric: [**IW-PSNR**] True GT: [**BDPT @ 524288 spp**].

M-ERPT	2	4	8	16	32	64	128	256	512	1024	2048	4096	8192	16384	32768	65536	131072
GT (BDPT)																	
262144	0.00042	0.00010	0.00003	0.00019	0.00003	0.00005	0.00011	-0.00004	-0.00003	-0.00012	0.00015	0.00001	0.00002	0.00023	-0.00002	-0.00013	-0.00001
131072	0.00022	0.00022	-0.00007	-0.00010	-0.00011	-0.00021	-0.00009	0.00000	-0.00012	-0.00026	-0.00021	-0.00029	-0.00043	-0.00008	-0.00019	-0.00027	
65536	0.00041	0.00020	0.00030	-0.00004	-0.00033	0.00006	-0.00006	-0.00047	0.00003	-0.00006	0.00009	-0.00020	-0.00046	0.00016	-0.00022		
32768	0.00140	0.00048	0.00053	0.00049	-0.00004	0.00069	-0.00006	0.00060	0.00013	-0.00016	0.00013	-0.00007	0.00038	0.00025			
16384	0.00235	0.00153	0.00103	0.00041	0.00150	0.00099	0.00096	0.00131	0.00192	0.00053	0.00118	0.00040	0.00089				
8192	0.00328	0.00210	0.00176	0.00136	0.00150	0.00245	0.00117	0.00127	0.00124	0.00138	0.00111	0.00151					
4096	0.00235	0.00093	-0.00107	0.00040	-0.00073	0.00035	-0.00207	0.00023	-0.00048	-0.00070	0.00022						
2048	0.00119	-0.00223	-0.00328	-0.00142	-0.00333	-0.00227	-0.00457	-0.00208	-0.00174	-0.00218							
1024	-0.00384	-0.01146	-0.01290	-0.00797	-0.01092	-0.01097	-0.01440	-0.01210	-0.01055								
512	-0.01673	-0.02469	-0.02826	-0.02276	-0.02600	-0.02425	-0.03785	-0.02826									
256	-0.04740	-0.06022	-0.06385	-0.05474	-0.05556	-0.06199	-0.08026										
128	-0.09093	-0.11162	-0.12556	-0.11711	-0.12635	-0.12563											
64	-0.16786	-0.20182	-0.21194	-0.20860	-0.22357												
32	-0.27392	-0.31485	-0.32760	-0.33052													
16	-0.40301	-0.44833	-0.46998														
8	-0.58850	-0.64238															
4	-0.80747																

(g) $\mathcal{P}^{\mathcal{C}}$ for Scene: [**Veach Door**] Algorithm: [**M-ERPT**] Metric: [**IW-PSNR**] True GT: [**BDPT @ 524288 spp**].

Table 84

18.5 Sponza

PT	2	4	8	16	32	64	128	256	512	1024	2048	4096	8192
GT (PT)													
16384	-0.00023	-0.00013	0.00003	-0.00007	-0.00003	-0.00028	-0.00016	-0.00203	-0.00120	-0.00284	-0.00619	-0.01103	-0.01403
8192	-0.00007	-0.00026	-0.00039	-0.00015	-0.00078	-0.00080	-0.00147	-0.00265	-0.00549	-0.00928	-0.01394	-0.02801	
4096	-0.00048	-0.00092	-0.00075	-0.00115	-0.00179	-0.00226	-0.00335	-0.00631	-0.01052	-0.02021	-0.03375		
2048	-0.00075	-0.00059	-0.00073	-0.00118	-0.00282	-0.00307	-0.00535	-0.01180	-0.01970	-0.03703			
1024	-0.00139	-0.00168	-0.00200	-0.00236	-0.00347	-0.00734	-0.01387	-0.02259	-0.04189				
512	-0.00209	-0.00348	-0.00344	-0.00536	-0.00778	-0.01282	-0.02537	-0.04239					
256	-0.00239	-0.00372	-0.00494	-0.00834	-0.01399	-0.02409	-0.04134						
128	-0.00453	-0.00544	-0.00818	-0.01344	-0.02526	-0.04440							
64	-0.00690	-0.01006	-0.01487	-0.02721	-0.04030								
32	-0.01211	-0.01860	-0.02461	-0.04300									
16	-0.02415	-0.03234	-0.04268										
8	-0.03727	-0.04712											
4	-0.05459												

(a) $\mathcal{P}^{\mathcal{C}}$ for Scene: [**Sponza**] Algorithm: [**PT**] Metric: [**IW-PSNR**] True GT: [**PT @ 32768 spp**].

BDPT	2	4	8	16	32	64	128	256	512	1024	2048	4096	8192
GT (PT)													
16384	0.00001	0.00002	-0.00005	-0.00038	0.00012	-0.00021	0.00003	-0.00097	-0.00103	-0.00338	-0.00337	-0.00737	-0.01125
8192	-0.00011	0.00000	-0.00041	-0.00025	-0.00026	-0.00087	-0.00103	-0.00242	-0.00278	-0.00831	-0.01276	-0.01996	
4096	0.00004	-0.00077	-0.00071	-0.00135	-0.00167	-0.00140	-0.00250	-0.00408	-0.00800	-0.01747	-0.02826		
2048	-0.00041	-0.00082	-0.00169	-0.00193	-0.00234	-0.00294	-0.00517	-0.00887	-0.01835	-0.03217			
1024	-0.00053	-0.00118	-0.00237	-0.00221	-0.00319	-0.00522	-0.00946	-0.01908	-0.03446				
512	-0.00066	-0.00203	-0.00334	-0.00573	-0.00627	-0.01162	-0.01703	-0.03614					
256	-0.00054	-0.00314	-0.00588	-0.00557	-0.01009	-0.01873	-0.03519						
128	-0.00300	-0.00562	-0.00890	-0.01116	-0.01810	-0.03683							
64	-0.00307	-0.00895	-0.01483	-0.02148	-0.03268								
32	-0.00831	-0.01383	-0.02333	-0.03644									
16	-0.02016	-0.02724	-0.04192										
8	-0.03118	-0.04191											
4	-0.04368												

(b) $\mathcal{P}^{\mathcal{C}}$ for Scene: [**Sponza**] Algorithm: [**BDPT**] Metric: [**IW-PSNR**] True GT: [**PT @ 32768 spp**].

PSSMLT	2	4	8	16	32	64	128	256	512	1024	2048	4096	8192
GT (PT)													
16384	-0.00005	-0.00016	-0.00017	-0.00016	0.00000	0.00011	0.00000	-0.00011	-0.00044	-0.00057	-0.00061	-0.00162	-0.00131
8192	-0.00019	-0.00027	-0.00031	-0.00055	-0.00025	-0.00033	-0.00102	-0.00155	-0.00206	-0.00199	-0.00299	-0.00336	
4096	-0.00024	-0.00050	-0.00051	-0.00070	-0.00095	-0.00121	-0.00204	-0.00259	-0.00382	-0.00439	-0.00640		
2048	-0.00034	-0.00078	-0.00083	-0.00090	-0.00143	-0.00180	-0.00348	-0.00601	-0.00939	-0.01344			
1024	-0.00027	-0.00080	-0.00110	-0.00196	-0.00283	-0.00396	-0.00819	-0.01249	-0.01967				
512	-0.00145	-0.00258	-0.00310	-0.00387	-0.00602	-0.00894	-0.01613	-0.02649					
256	-0.00182	-0.00296	-0.00443	-0.00677	-0.01175	-0.01703	-0.02943						
128	-0.00514	-0.00695	-0.00879	-0.01201	-0.02036	-0.03184							
64	-0.00647	-0.01011	-0.01636	-0.02350	-0.03756								
32	-0.01198	-0.01838	-0.02551	-0.03888									
16	-0.02318	-0.03585	-0.05345										
8	-0.04448	-0.05648											
4	-0.07451												

(c) $\mathcal{P}^{\mathcal{C}}$ for Scene: [**Sponza**] Algorithm: [**PSSMLT**] Metric: [**IW-PSNR**] True GT: [**PT @ 32768 spp**].

MLT	2	4	8	16	32	64	128	256	512	1024	2048	4096	8192
GT (PT)													
16384	-0.00003	-0.00000	0.00011	0.00018	-0.00023	0.00020	-0.00037	-0.00094	-0.00031	-0.00145	-0.00131	-0.00200	-0.00131
8192	0.00016	0.00018	0.00000	0.00024	-0.00049	-0.00023	-0.00124	-0.00123	-0.00259	-0.00237	-0.00291	-0.00293	
4096	-0.00031	-0.00053	-0.00095	-0.00039	-0.00143	-0.00116	-0.00282	-0.00370	-0.00483	-0.00628	-0.00558		
2048	-0.00036	-0.00072	-0.00076	-0.00135	-0.00270	-0.00396	-0.00778	-0.00981	-0.01420	-0.01575			
1024	-0.00055	-0.00133	-0.00095	-0.00229	-0.00570	-0.01036	-0.01460	-0.01865	-0.02693				
512	-0.00144	-0.00193	-0.00307	-0.00578	-0.01206	-0.01634	-0.02906	-0.03863					
256	-0.00224	-0.00473	-0.00692	-0.01509	-0.02421	-0.03644	-0.05239						
128	-0.00284	-0.00673	-0.01091	-0.02625	-0.04259	-0.06554							
64	-0.00504	-0.01457	-0.02669	-0.05072	-0.07529								
32	-0.01310	-0.02597	-0.04904	-0.09341									
16	-0.02074	-0.05245	-0.09210										
8	-0.04843	-0.10719											
4	-0.10947												

(d) $\mathcal{P}^{\mathcal{C}}$ for Scene: [**Sponza**] Algorithm: [**MLT**] Metric: [**IW-PSNR**] True GT: [**PT @ 32768 spp**].

M-MLT	2	4	8	16	32	64	128	256	512	1024	2048	4096	8192
GT (PT)													
16384	-0.00011	-0.00015	-0.00020	0.00017	0.00001	-0.00006	-0.00099	-0.00047	-0.00020	-0.00130	-0.00137	-0.00091	-0.00077
8192	0.00002	-0.00015	-0.00034	-0.00009	-0.00011	-0.00064	-0.00201	-0.00188	-0.00289	-0.00271	-0.00302	-0.00204	
4096	-0.00038	-0.00044	-0.00021	-0.00023	-0.00103	-0.00233	-0.00312	-0.00485	-0.00718	-0.00520	-0.00723		
2048	-0.00002	-0.00051	-0.00103	-0.00140	-0.00242	-0.00376	-0.00792	-0.01093	-0.01278	-0.01422			
1024	-0.00114	-0.00135	-0.00236	-0.00298	-0.00436	-0.00923	-0.01439	-0.02031	-0.02538				
512	-0.00145	-0.00258	-0.00309	-0.00591	-0.01103	-0.01641	-0.02988	-0.04137					
256	-0.00323	-0.00353	-0.00751	-0.01236	-0.02140	-0.03388	-0.05751						
128	-0.00559	-0.00698	-0.01330	-0.02360	-0.03959	-0.06311							
64	-0.01163	-0.01583	-0.02888	-0.04357	-0.07803								
32	-0.01726	-0.02744	-0.04992	-0.08150									
16	-0.03697	-0.05664	-0.10015										
8	-0.07221	-0.10177											
4	-0.13089												

(e) $\mathcal{P}^{\mathcal{C}}$ for Scene: [**Sponza**] Algorithm: [**M-MLT**] Metric: [**IW-PSNR**] True GT: [**PT @ 32768 spp**].

ERPT	2	4	8	16	32	64	128	256	512	1024	2048	4096	8192
GT (PT)													
16384	-0.00063	-0.00018	-0.00009	0.00015	-0.00067	-0.00084	-0.00010	-0.00043	-0.00026	-0.00002	-0.00067	-0.00018	-0.00045
8192	-0.00125	-0.00149	-0.00106	-0.00125	-0.00265	-0.00282	-0.00079	-0.00116	-0.00171	-0.00134	-0.00227	-0.00216	
4096	-0.00245	-0.00270	-0.00323	-0.00346	-0.00394	-0.00439	-0.00284	-0.00370	-0.00373	-0.00367	-0.00394		
2048	-0.00681	-0.00789	-0.00768	-0.00790	-0.00888	-0.00894	-0.00826	-0.00797	-0.00947	-0.00827			
1024	-0.01531	-0.01488	-0.01544	-0.01657	-0.01779	-0.01887	-0.01677	-0.01609	-0.01709				
512	-0.02948	-0.03107	-0.03254	-0.03531	-0.03376	-0.03717	-0.03325	-0.03311					
256	-0.05382	-0.05652	-0.06062	-0.06269	-0.06462	-0.06728	-0.06121						
128	-0.10173	-0.10371	-0.10832	-0.12020	-0.11782	-0.12021							
64	-0.16910	-0.17623	-0.17883	-0.19334	-0.18847								
32	-0.26805	-0.27098	-0.27704	-0.29212									
16	-0.38891	-0.39868	-0.40534										
8	-0.53749	-0.54776											
4	-0.69814												

(f) $\mathcal{P}^{\mathcal{C}}$ for Scene: [**Sponza**] Algorithm: [**ERPT**] Metric: [**IW-PSNR**] True GT: [**PT @ 32768 spp**].

M-ERPT	2	4	8	16	32	64	128	256	512	1024	2048	4096	8192
GT (PT)													
16384	-0.00073	-0.00045	-0.00003	-0.00072	-0.00064	-0.00054	-0.00044	-0.00064	0.00011	-0.00009	-0.00078	-0.00052	-0.00004
8192	-0.00114	-0.00164	-0.00141	-0.00225	-0.00160	-0.00208	-0.00179	-0.00207	-0.00084	-0.00088	-0.00102	-0.00158	
4096	-0.00345	-0.00405	-0.00428	-0.00426	-0.00426	-0.00422	-0.00415	-0.00270	-0.00317	-0.00308	-0.00410		
2048	-0.00693	-0.00693	-0.00819	-0.00825	-0.00860	-0.00836	-0.00827	-0.00884	-0.00891	-0.00813			
1024	-0.01643	-0.01310	-0.01715	-0.01659	-0.01771	-0.01659	-0.01847	-0.02052	-0.01687				
512	-0.02964	-0.02956	-0.03134	-0.03381	-0.03385	-0.03500	-0.03298	-0.03556					
256	-0.05383	-0.05946	-0.06036	-0.05942	-0.06584	-0.06389	-0.06160						
128	-0.09966	-0.10796	-0.11364	-0.11013	-0.11607	-0.11648							
64	-0.16819	-0.17470	-0.18292	-0.18633	-0.18778								
32	-0.25974	-0.27708	-0.28325	-0.28597									
16	-0.38387	-0.39841	-0.40991										
8	-0.53361	-0.54892											
4	-0.69336												

(g) $\mathcal{P}^{\mathcal{C}}$ for Scene: [**Sponza**] Algorithm: [**M-ERPT**] Metric: [**IW-PSNR**] True GT: [**PT @ 32768 spp**].

Table 85

19 PSNR

19.1 Cornell Box

PT	2	4	8	16	32	64	128	256	512	1024	2048	4096	8192
**GT (PT)**													
16384	-0.00002	-0.00004	-0.00003	-0.00001	-0.00011	-0.00019	-0.00037	-0.00055	-0.00142	-0.00248	-0.00443	-0.00639	-0.01064
8192	-0.00006	-0.00015	-0.00008	-0.00016	-0.00023	-0.00049	-0.00120	-0.00199	-0.00383	-0.00687	-0.01184	-0.01814	
4096	-0.00011	-0.00020	-0.00020	-0.00041	-0.00081	-0.00123	-0.00298	-0.00529	-0.00912	-0.01557	-0.02483		
2048	-0.00030	-0.00030	-0.00056	-0.00095	-0.00171	-0.00327	-0.00591	-0.01096	-0.01862	-0.03071			
1024	-0.00034	-0.00056	-0.00115	-0.00207	-0.00361	-0.00645	-0.01188	-0.02045	-0.03509				
512	-0.00076	-0.00137	-0.00214	-0.00411	-0.00755	-0.01271	-0.02281	-0.03748					
256	-0.00152	-0.00258	-0.00418	-0.00821	-0.01392	-0.02532	-0.04316						
128	-0.00294	-0.00517	-0.00911	-0.01599	-0.02750	-0.04660							
64	-0.00564	-0.00976	-0.01748	-0.03065	-0.05040								
32	-0.01168	-0.01912	-0.03345	-0.05535									
16	-0.02255	-0.03744	-0.06070										
8	-0.04086	-0.06846											
4	-0.07464												

(a) $\mathcal{P}^{\mathcal{C}}$ for Scene: [**Cornell Box**] Algorithm: [**PT**] Metric: [**PSNR**] True GT: [**PT @ 32768 spp**].

BDPT	2	4	8	16	32	64	128	256	512	1024	2048	4096	8192
**GT (PT)**													
16384	-0.00003	-0.00006	-0.00004	-0.00017	-0.00027	-0.00061	-0.00107	-0.00166	-0.00337	-0.00490	-0.00808	-0.01126	-0.01574
8192	-0.00013	-0.00012	-0.00029	-0.00052	-0.00086	-0.00154	-0.00306	-0.00530	-0.00914	-0.01445	-0.02151	-0.02961	
4096	-0.00010	-0.00042	-0.00062	-0.00116	-0.00199	-0.00382	-0.00684	-0.01220	-0.01974	-0.03099	-0.04385		
2048	-0.00032	-0.00059	-0.00129	-0.00232	-0.00456	-0.00831	-0.01407	-0.02395	-0.03828	-0.05584			
1024	-0.00090	-0.00161	-0.00270	-0.00484	-0.00876	-0.01525	-0.02679	-0.04461	-0.06651				
512	-0.00163	-0.00315	-0.00560	-0.01003	-0.01738	-0.03012	-0.04909	-0.07573					
256	-0.00331	-0.00580	-0.01093	-0.01980	-0.03366	-0.05444	-0.08511						
128	-0.00661	-0.01207	-0.02131	-0.03599	-0.06127	-0.09295							
64	-0.01289	-0.02287	-0.04023	-0.06522	-0.10135								
32	-0.02493	-0.04299	-0.07044	-0.10916									
16	-0.04632	-0.07622	-0.11735										
8	-0.08200	-0.12683											
4	-0.13652												

(b) $\mathcal{P}^{\mathcal{C}}$ for Scene: [**Cornell Box**] Algorithm: [**BDPT**] Metric: [**PSNR**] True GT: [**PT @ 32768 spp**].

PSSMLT	2	4	8	16	32	64	128	256	512	1024	2048	4096	8192
**GT (PT)**													
16384	-0.00003	-0.00001	-0.00004	-0.00009	-0.00025	-0.00063	-0.00097	-0.00128	-0.00164	-0.00224	-0.00244	-0.00271	-0.00267
8192	-0.00009	-0.00011	-0.00012	-0.00033	-0.00072	-0.00149	-0.00216	-0.00343	-0.00455	-0.00579	-0.00661	-0.00720	
4096	-0.00017	-0.00029	-0.00062	-0.00103	-0.00192	-0.00348	-0.00556	-0.00817	-0.01095	-0.01348	-0.01529		
2048	-0.00039	-0.00066	-0.00125	-0.00216	-0.00422	-0.00704	-0.01117	-0.01665	-0.02161	-0.02649			
1024	-0.00080	-0.00155	-0.00275	-0.00468	-0.00805	-0.01388	-0.02169	-0.03153	-0.04015				
512	-0.00168	-0.00301	-0.00529	-0.00950	-0.01653	-0.02717	-0.04100	-0.05714					
256	-0.00357	-0.00650	-0.01136	-0.01946	-0.03220	-0.05060	-0.07259						
128	-0.00703	-0.01255	-0.02150	-0.03628	-0.05807	-0.08591							
64	-0.01386	-0.02390	-0.03998	-0.06476	-0.09780								
32	-0.02592	-0.04466	-0.07051	-0.10747									
16	-0.04745	-0.07790	-0.11848										
8	-0.08416	-0.12876											
4	-0.14176												

(c) $\mathcal{P}^{\mathcal{C}}$ for Scene: [**Cornell Box**] Algorithm: [**PSSMLT**] Metric: [**PSNR**] True GT: [**PT @ 32768 spp**].

MLT	2	4	8	16	32	64	128	256	512	1024	2048	4096	8192
**GT (PT)**													
16384	-0.00000	0.00000	-0.00002	0.00001	-0.00003	-0.00009	-0.00016	-0.00045	-0.00074	-0.00108	-0.00128	-0.00204	-0.00227
8192	-0.00001	-0.00004	-0.00005	-0.00009	-0.00015	-0.00037	-0.00068	-0.00125	-0.00205	-0.00315	-0.00421	-0.00541	
4096	-0.00003	-0.00008	-0.00013	-0.00025	-0.00059	-0.00111	-0.00185	-0.00309	-0.00511	-0.00724	-0.01000		
2048	-0.00012	-0.00019	-0.00027	-0.00037	-0.00096	-0.00185	-0.00340	-0.00619	-0.00998	-0.01458			
1024	-0.00023	-0.00038	-0.00069	-0.00122	-0.00231	-0.00422	-0.00731	-0.01234	-0.01956				
512	-0.00059	-0.00088	-0.00161	-0.00274	-0.00488	-0.00855	-0.01464	-0.02423					
256	-0.00101	-0.00193	-0.00345	-0.00571	-0.01002	-0.01703	-0.02805						
128	-0.00183	-0.00310	-0.00554	-0.00974	-0.01739	-0.03009							
64	-0.00331	-0.00621	-0.01180	-0.02024	-0.03425								
32	-0.00739	-0.01241	-0.02199	-0.03683									
16	-0.01410	-0.02350	-0.04164										
8	-0.02502	-0.04305											
4	-0.04991												

(d) $\mathcal{P}^{\mathcal{C}}$ for Scene: [**Cornell Box**] Algorithm: [**MLT**] Metric: [**PSNR**] True GT: [**PT @ 32768 spp**].

M-MLT	2	4	8	16	32	64	128	256	512	1024	2048	4096	8192
**GT (PT)**													
16384	-0.00002	0.00004	0.00002	0.00000	0.00003	-0.00008	-0.00024	-0.00051	-0.00074	-0.00117	-0.00158	-0.00194	-0.00230
8192	-0.00004	-0.00004	-0.00010	-0.00015	-0.00026	-0.00043	-0.00070	-0.00128	-0.00235	-0.00334	-0.00451	-0.00539	
4096	-0.00004	-0.00006	-0.00015	-0.00030	-0.00054	-0.00105	-0.00190	-0.00311	-0.00519	-0.00750	-0.01017		
2048	-0.00017	-0.00021	-0.00039	-0.00071	-0.00116	-0.00206	-0.00355	-0.00635	-0.01021	-0.01514			
1024	-0.00026	-0.00030	-0.00070	-0.00135	-0.00246	-0.00440	-0.00756	-0.01298	-0.02016				
512	-0.00041	-0.00096	-0.00164	-0.00288	-0.00473	-0.00854	-0.01449	-0.02410					
256	-0.00107	-0.00192	-0.00316	-0.00544	-0.00945	-0.01670	-0.02818						
128	-0.00175	-0.00320	-0.00603	-0.01067	-0.01853	-0.03215							
64	-0.00344	-0.00684	-0.01192	-0.02006	-0.03413								
32	-0.00777	-0.01345	-0.02328	-0.03879									
16	-0.01493	-0.02619	-0.04350										
8	-0.02864	-0.04675											
4	-0.05237												

(e) $\mathcal{P}^{\mathcal{C}}$ for Scene: [**Cornell Box**] Algorithm: [**M-MLT**] Metric: [**PSNR**] True GT: [**PT @ 32768 spp**].

ERPT	2	4	8	16	32	64	128	256	512	1024	2048	4096	8192
**GT (PT)**													
16384	-0.00029	-0.00036	-0.00022	-0.00030	-0.00022	-0.00030	-0.00031	-0.00029	-0.00040	-0.00032	-0.00019	-0.00029	-0.00036
8192	-0.00089	-0.00085	-0.00089	-0.00072	-0.00081	-0.00078	-0.00075	-0.00092	-0.00085	-0.00084	-0.00077	-0.00092	
4096	-0.00184	-0.00200	-0.00177	-0.00166	-0.00179	-0.00209	-0.00195	-0.00189	-0.00186	-0.00191	-0.00183		
2048	-0.00398	-0.00403	-0.00402	-0.00388	-0.00381	-0.00414	-0.00389	-0.00375	-0.00375	-0.00406			
1024	-0.00810	-0.00776	-0.00778	-0.00781	-0.00775	-0.00833	-0.00810	-0.00785	-0.00833				
512	-0.01584	-0.01546	-0.01557	-0.01559	-0.01582	-0.01670	-0.01584	-0.01609					
256	-0.03013	-0.03098	-0.03043	-0.03025	-0.03029	-0.03119	-0.03037						
128	-0.05468	-0.05527	-0.05514	-0.05522	-0.05411	-0.05602							
64	-0.09290	-0.09364	-0.09313	-0.09287	-0.09146								
32	-0.14663	-0.14714	-0.14781	-0.14726									
16	-0.21539	-0.21759	-0.21762										
8	-0.29956	-0.30147											
4	-0.39551												

(f) $\mathcal{P}^{\mathcal{C}}$ for Scene: [**Cornell Box**] Algorithm: [**ERPT**] Metric: [**PSNR**] True GT: [**PT @ 32768 spp**].

M-ERPT	2	4	8	16	32	64	128	256	512	1024	2048	4096	8192
**GT (PT)**													
16384	-0.00028	-0.00029	-0.00029	-0.00034	-0.00029	-0.00040	-0.00032	-0.00032	-0.00035	-0.00020	-0.00014	-0.00030	-0.00028
8192	-0.00086	-0.00077	-0.00074	-0.00100	-0.00075	-0.00068	-0.00068	-0.00081	-0.00065	-0.00072	-0.00071	-0.00081	
4096	-0.00172	-0.00167	-0.00173	-0.00181	-0.00181	-0.00174	-0.00173	-0.00166	-0.00175	-0.00168	-0.00179		
2048	-0.00381	-0.00365	-0.00370	-0.00386	-0.00399	-0.00382	-0.00360	-0.00350	-0.00347	-0.00374			
1024	-0.00729	-0.00742	-0.00785	-0.00755	-0.00766	-0.00740	-0.00723	-0.00799	-0.00739				
512	-0.01442	-0.01476	-0.01459	-0.01520	-0.01501	-0.01448	-0.01505	-0.01461					
256	-0.02873	-0.02919	-0.02910	-0.02886	-0.02934	-0.02862	-0.02957						
128	-0.05199	-0.05231	-0.05218	-0.05320	-0.05308	-0.05225							
64	-0.09073	-0.09018	-0.08944	-0.08959	-0.09089								
32	-0.14231	-0.14313	-0.14215	-0.14273									
16	-0.21060	-0.21218	-0.21025										
8	-0.29398	-0.29509											
4	-0.39024												

(g) $\mathcal{P}^{\mathcal{C}}$ for Scene: [**Cornell Box**] Algorithm: [**M-ERPT**] Metric: [**PSNR**] True GT: [**PT @ 32768 spp**].

Table 86

19.2 Torus

PT	2	4	8	16	32	64	128	256	512	1024	2048	4096	8192	16384	32768	65536	131072
GT (BDPT)																	
262144	0.00024	0.00036	0.00026	0.00023	0.00038	0.00022	0.00011	-0.00013	0.00004	-0.00016	-0.00040	-0.00088	-0.00181	-0.00300	-0.00513	-0.00997	-0.01613
131072	0.00039	0.00021	0.00036	0.00021	0.00054	-0.00008	-0.00022	-0.00038	-0.00072	-0.00078	-0.00165	-0.00380	-0.00577	-0.01070	-0.01804	-0.02879	
65536	0.00030	-0.00002	0.00028	0.00002	-0.00013	-0.00015	-0.00081	-0.00103	-0.00170	-0.00254	-0.00451	-0.00786	-0.01378	-0.02227	-0.03578		
32768	-0.00093	-0.00152	-0.00171	-0.00218	-0.00225	-0.00220	-0.00230	-0.00357	-0.00457	-0.00656	-0.00986	-0.01669	-0.02830	-0.04573			
16384	-0.00254	-0.00396	-0.00477	-0.00535	-0.00485	-0.00618	-0.00608	-0.00819	-0.00952	-0.01340	-0.01908	-0.03298	-0.05149				
8192	-0.00554	-0.00738	-0.00839	-0.00954	-0.01043	-0.01143	-0.01192	-0.01582	-0.01706	-0.02552	-0.03568	-0.05795					
4096	-0.01204	-0.01551	-0.01724	-0.02022	-0.01880	-0.02121	-0.02324	-0.02703	-0.03173	-0.04365	-0.06256						
2048	-0.02019	-0.02664	-0.03045	-0.03423	-0.03433	-0.03604	-0.03907	-0.04512	-0.05429	-0.07153							
1024	-0.02719	-0.03785	-0.04336	-0.04850	-0.04847	-0.05211	-0.05648	-0.06557	-0.07938								
512	-0.03488	-0.04806	-0.05579	-0.06145	-0.06340	-0.07000	-0.07413	-0.08570									
256	-0.03641	-0.04931	-0.05784	-0.06294	-0.06564	-0.07439	-0.08208										
128	-0.02121	-0.03263	-0.03825	-0.04797	-0.05629	-0.06551											
64	-0.01065	-0.02531	-0.02601	-0.03692	-0.04666												
32	0.00281	-0.00767	-0.00852	-0.02409													
16	0.01973	0.01174	0.01529														
8	0.01992	0.01098															
4	0.01012																

(a) $\mathcal{P}^{\mathcal{C}}$ for Scene: [**Torus**] Algorithm: [**PT**] Metric: [**PSNR**] True GT: [**BDPT @ 524288 spp**].

BDPT	2	4	8	16	32	64	128	256	512	1024	2048	4096	8192	16384	32768	65536	131072
GT (BDPT)																	
262144	0.00029	0.00040	0.00039	0.00031	-0.00006	0.00023	0.00014	0.00014	-0.00000	-0.00010	-0.00079	-0.00052	-0.00176	-0.00279	-0.00502	-0.00995	-0.01642
131072	0.00028	0.00048	0.00039	0.00024	0.00025	-0.00006	-0.00017	-0.00020	-0.00054	-0.00117	-0.00219	-0.00306	-0.00576	-0.00975	-0.01576	-0.02896	
65536	0.00030	0.00033	0.00030	0.00007	-0.00020	-0.00016	-0.00073	-0.00141	-0.00156	-0.00262	-0.00488	-0.00683	-0.01216	-0.02146	-0.03471		
32768	-0.00074	-0.00154	-0.00176	-0.00242	-0.00180	-0.00261	-0.00301	-0.00412	-0.00372	-0.00677	-0.01087	-0.01671	-0.02610	-0.04234			
16384	-0.00237	-0.00386	-0.00437	-0.00498	-0.00498	-0.00555	-0.00618	-0.00786	-0.00962	-0.01401	-0.02116	-0.03189	-0.04753				
8192	-0.00467	-0.00681	-0.00826	-0.00851	-0.00854	-0.01079	-0.01286	-0.01391	-0.01930	-0.02468	-0.03492	-0.05484					
4096	-0.01005	-0.01433	-0.01651	-0.01960	-0.01911	-0.02075	-0.02236	-0.02709	-0.03193	-0.04184	-0.06198						
2048	-0.01893	-0.02588	-0.02837	-0.03166	-0.03323	-0.03526	-0.03832	-0.04321	-0.05372	-0.06937							
1024	-0.02618	-0.03526	-0.04224	-0.04549	-0.04923	-0.04924	-0.05758	-0.06409	-0.07990								
512	-0.03296	-0.04492	-0.05372	-0.05823	-0.06195	-0.06560	-0.07433	-0.08241									
256	-0.03209	-0.04670	-0.05581	-0.06023	-0.06582	-0.07348	-0.08045										
128	-0.01877	-0.02988	-0.03898	-0.04318	-0.05072	-0.06055											
64	-0.00928	-0.01789	-0.02589	-0.03531	-0.04754												
32	0.00424	0.00126	-0.01081	-0.01932													
16	0.02157	0.01895	0.00878														
8	0.02216	0.01895															
4	0.01132																

(b) $\mathcal{P}^{\mathcal{C}}$ for Scene: [**Torus**] Algorithm: [**BDPT**] Metric: [**PSNR**] True GT: [**BDPT @ 524288 spp**].

PSSMLT	2	4	8	16	32	64	128	256	512	1024	2048	4096	8192	16384
GT (BDPT)														
262144	0.00039	0.00041	0.00032	0.00040	0.00025	0.00030	-0.00006	-0.00015	-0.00039	-0.00076	-0.00100	-0.00127	-0.00241	-0.00174
131072	0.00028	0.00036	0.00033	0.00029	-0.00010	-0.00035	-0.00085	-0.00149	-0.00155	-0.00272	-0.00412	-0.00519	-0.00886	-0.00751
65536	0.00044	0.00003	-0.00052	-0.00041	-0.00107	-0.00144	-0.00210	-0.00365	-0.00468	-0.00726	-0.01020	-0.01323	-0.02046	-0.01858
32768	-0.00021	-0.00122	-0.00170	-0.00205	-0.00327	-0.00441	-0.00596	-0.00897	-0.01204	-0.01729	-0.02263	-0.02800	-0.04048	-0.03788
16384	-0.00257	-0.00453	-0.00591	-0.00757	-0.00921	-0.01120	-0.01486	-0.01930	-0.02464	-0.03349	-0.04352	-0.05331	-0.07297	
8192	-0.00425	-0.00682	-0.00939	-0.01058	-0.01504	-0.02032	-0.02605	-0.03446	-0.04488	-0.05893	-0.07666	-0.09127		
4096	-0.00849	-0.01422	-0.01749	-0.02031	-0.02693	-0.03452	-0.04464	-0.05854	-0.07448	-0.09568	-0.11922			
2048	-0.01853	-0.02755	-0.03385	-0.03912	-0.04822	-0.06036	-0.07421	-0.09463	-0.11539	-0.14452				
1024	-0.02552	-0.03888	-0.04849	-0.05526	-0.06790	-0.08428	-0.10612	-0.13178	-0.16014					
512	-0.03268	-0.04963	-0.06123	-0.06953	-0.08835	-0.10952	-0.13293	-0.16392						
256	-0.03444	-0.05381	-0.06950	-0.08009	-0.10105	-0.12668	-0.15466							
128	-0.02083	-0.04157	-0.05895	-0.07149	-0.09629	-0.12458								
64	-0.01632	-0.03817	-0.05627	-0.06709	-0.09727									
32	-0.00253	-0.02581	-0.04659	-0.05882										
16	0.00950	-0.01427	-0.03612											
8	0.01022	-0.01416												
4	0.00016													

(c) $\mathcal{P}^{\mathcal{C}}$ for Scene: [**Torus**] Algorithm: [**PSSMLT**] Metric: [**PSNR**] True GT: [**BDPT @ 524288 spp**].

MLT	2	4	8	16	32	64	128	256	512	1024	2048	4096	8192	16384
GT (BDPT)														
262144	0.00028	0.00034	0.00013	0.00011	-0.00004	0.00001	-0.00020	-0.00041	-0.00017	-0.00042	-0.00040	-0.00044	-0.00051	0.00005
131072	0.00045	0.00007	-0.00051	-0.00071	-0.00101	-0.00121	-0.00136	-0.00174	-0.00170	-0.00194	-0.00221	-0.00216	-0.00245	-0.00259
65536	0.00087	0.00029	-0.00061	-0.00112	-0.00208	-0.00231	-0.00288	-0.00396	-0.00401	-0.00461	-0.00485	-0.00506	-0.00563	-0.00612
32768	0.00055	0.00002	-0.00204	-0.00304	-0.00491	-0.00504	-0.00619	-0.00906	-0.00869	-0.00983	-0.01034	-0.01054	-0.01205	-0.01372
16384	0.00065	-0.00037	-0.00347	-0.00501	-0.00948	-0.00930	-0.01137	-0.01508	-0.01549	-0.01777	-0.01834	-0.01926	-0.02151	
8192	0.00018	-0.00234	-0.00934	-0.01083	-0.01822	-0.01968	-0.02441	-0.03111	-0.03189	-0.03567	-0.03706	-0.03826		
4096	0.00012	-0.00450	-0.01589	-0.02077	-0.03267	-0.03441	-0.04246	-0.05295	-0.05382	-0.05972	-0.06197			
2048	-0.00033	-0.00904	-0.02765	-0.03452	-0.05442	-0.05749	-0.06775	-0.08239	-0.08417	-0.09176				
1024	-0.00259	-0.01640	-0.04141	-0.05264	-0.08109	-0.08659	-0.10115	-0.11891	-0.12060					
512	-0.00190	-0.02011	-0.05514	-0.06772	-0.10506	-0.11039	-0.12760	-0.15128						
256	0.00121	-0.02433	-0.06587	-0.08013	-0.12208	-0.13059	-0.15055							
128	0.00909	-0.02033	-0.06353	-0.08019	-0.12447	-0.13272								
64	0.01244	-0.02168	-0.06387	-0.08282	-0.13169									
32	0.01959	-0.01578	-0.05870	-0.07855										
16	0.02738	-0.01097	-0.05235											
8	0.02439	-0.01286												
4	0.02610													

(d) $\mathcal{P}^{\mathcal{C}}$ for Scene: [**Torus**] Algorithm: [**MLT**] Metric: [**PSNR**] True GT: [**BDPT @ 524288 spp**].

M-MLT	2	4	8	16	32	64	128	256	512	1024	2048	4096	8192	16384
GT (BDPT)														
262144	0.00014	0.00009	0.00010	0.00011	0.00025	-0.00005	-0.00058	-0.00099	-0.00083	-0.00104	-0.00130	-0.00140	-0.00143	-0.00094
131072	0.00026	0.00012	-0.00034	-0.00010	-0.00044	-0.00141	-0.00197	-0.00202	-0.00263	-0.00328	-0.00349	-0.00379	-0.00432	-0.00460
65536	0.00061	0.00006	-0.00076	-0.00107	-0.00135	-0.00282	-0.00416	-0.00570	-0.00597	-0.00700	-0.00801	-0.00808	-0.00927	-0.01065
32768	0.00055	-0.00037	-0.00268	-0.00322	-0.00448	-0.00648	-0.00923	-0.01143	-0.01276	-0.01427	-0.01610	-0.01664	-0.01849	-0.02246
16384	0.00051	-0.00145	-0.00490	-0.00452	-0.00809	-0.01178	-0.01598	-0.02087	-0.02262	-0.02592	-0.02871	-0.02926	-0.03306	
8192	0.00015	-0.00396	-0.00950	-0.01039	-0.01664	-0.02424	-0.03038	-0.03970	-0.04464	-0.04868	-0.05463	-0.05555		
4096	-0.00117	-0.00732	-0.01875	-0.01723	-0.02852	-0.04189	-0.05301	-0.06559	-0.07112	-0.07931	-0.08673			
2048	-0.00200	-0.01240	-0.03107	-0.03188	-0.05088	-0.06663	-0.08221	-0.10084	-0.10903	-0.11956				
1024	-0.00466	-0.02125	-0.04785	-0.04907	-0.07264	-0.09862	-0.12001	-0.14154	-0.15118					
512	-0.00582	-0.02719	-0.06691	-0.06435	-0.09173	-0.12328	-0.15006	-0.17584						
256	-0.00511	-0.03141	-0.07793	-0.07603	-0.10890	-0.14492	-0.17496							
128	0.00047	-0.02531	-0.08068	-0.07553	-0.11095	-0.15060								
64	0.00276	-0.02400	-0.08883	-0.08162	-0.12006									
32	0.00754	-0.01909	-0.08694	-0.07805										
16	0.00988	-0.01588	-0.08635											
8	0.00934	-0.01719												
4	0.00975													

(e) $\mathcal{P}^{\mathcal{C}}$ for Scene: [**Torus**] Algorithm: [**M-MLT**] Metric: [**PSNR**] True GT: [**BDPT @ 524288 spp**].

ERPT	2	4	8	16	32	64	128	256	512	1024	2048	4096	8192	16384	32768	65536	131072
GT (BDPT)																	
262144	0.00020	-0.00003	-0.00048	-0.00042	-0.00145	-0.00131	-0.00135	-0.00132	-0.00161	-0.00164	-0.00173	-0.00215	-0.00227	-0.00201	-0.00212	-0.00189	-0.00191
131072	-0.00096	-0.00180	-0.00196	-0.00305	-0.00283	-0.00380	-0.00417	-0.00441	-0.00533	-0.00617	-0.00605	-0.00742	-0.00757	-0.00711	-0.00688	-0.00685	
65536	-0.00264	-0.00415	-0.00516	-0.00608	-0.00741	-0.00764	-0.00943	-0.00969	-0.01091	-0.01234	-0.01428	-0.01587	-0.01664	-0.01609	-0.01585		
32768	-0.00663	-0.00908	-0.01056	-0.01392	-0.01588	-0.01700	-0.01910	-0.02089	-0.02250	-0.02648	-0.02970	-0.03326	-0.03366	-0.03134			
16384	-0.01427	-0.01916	-0.02278	-0.02600	-0.02793	-0.03189	-0.03625	-0.04072	-0.04323	-0.04942	-0.05522	-0.05978	-0.06164				
8192	-0.02680	-0.03637	-0.04168	-0.04759	-0.05079	-0.05754	-0.06494	-0.07230	-0.07534	-0.08496	-0.09289	-0.09877					
4096	-0.04756	-0.06001	-0.07029	-0.07939	-0.08578	-0.09427	-0.10173	-0.11320	-0.11850	-0.13015	-0.14005						
2048	-0.07497	-0.09652	-0.11151	-0.12181	-0.12843	-0.13801	-0.15034	-0.16552	-0.17007	-0.18553							
1024	-0.10727	-0.13690	-0.14843	-0.16578	-0.17485	-0.18513	-0.20076	-0.21722	-0.22355								
512	-0.13361	-0.16886	-0.18494	-0.20505	-0.21258	-0.22515	-0.23960	-0.25883									
256	-0.15256	-0.19325	-0.21124	-0.22724	-0.24020	-0.25153	-0.26996										
128	-0.14806	-0.19254	-0.21136	-0.23420	-0.24777	-0.25899											
64	-0.15036	-0.19845	-0.21987	-0.24225	-0.25492												
32	-0.14596	-0.19873	-0.21750	-0.23851													
16	-0.13657	-0.19546	-0.21537														
8	-0.14238	-0.20170															
4	-0.16455																

(f) $\mathcal{P}^{\mathcal{C}}$ for Scene: [**Torus**] Algorithm: [**ERPT**] Metric: [**PSNR**] True GT: [**BDPT @ 524288 spp**].

M-ERPT	2	4	8	16	32	64	128	256	512	1024	2048	4096	8192	16384	32768	65536	131072
GT (BDPT)																	
262144	0.00010	-0.00040	-0.00012	-0.00081	-0.00061	-0.00083	-0.00196	-0.00174	-0.00184	-0.00190	-0.00244	-0.00218	-0.00251	-0.00248	-0.00214	-0.00217	-0.00224
131072	-0.00085	-0.00157	-0.00121	-0.00149	-0.00253	-0.00284	-0.00443	-0.00552	-0.00617	-0.00599	-0.00670	-0.00661	-0.00661	-0.00682	-0.00702	-0.00688	
65536	-0.00163	-0.00294	-0.00409	-0.00520	-0.00721	-0.00790	-0.01044	-0.01226	-0.01360	-0.01374	-0.01505	-0.01468	-0.01468	-0.01520	-0.01554		
32768	-0.00468	-0.00723	-0.00819	-0.01086	-0.01443	-0.01904	-0.02245	-0.02508	-0.02666	-0.02847	-0.03137	-0.03110	-0.03082	-0.03078			
16384	-0.00914	-0.01203	-0.01868	-0.02102	-0.02752	-0.03554	-0.04074	-0.04519	-0.04993	-0.05246	-0.05713	-0.05676	-0.05687				
8192	-0.01722	-0.02309	-0.03404	-0.03925	-0.05105	-0.06304	-0.07281	-0.07869	-0.08704	-0.08961	-0.09578	-0.09484					
4096	-0.03131	-0.04144	-0.05906	-0.06789	-0.08419	-0.10237	-0.11230	-0.12182	-0.13095	-0.13528	-0.14361						
2048	-0.05380	-0.06548	-0.09302	-0.10413	-0.12783	-0.15121	-0.16494	-0.17408	-0.18619	-0.19149							
1024	-0.08169	-0.09702	-0.13127	-0.14566	-0.17571	-0.20184	-0.21642	-0.22765	-0.24101								
512	-0.10361	-0.12373	-0.16062	-0.18076	-0.21196	-0.24084	-0.25724	-0.27127									
256	-0.12293	-0.14218	-0.18346	-0.20391	-0.23671	-0.26745	-0.28676										
128	-0.12376	-0.14065	-0.18472	-0.21024	-0.24267	-0.27313											
64	-0.13045	-0.14275	-0.18611	-0.21802	-0.24567												
32	-0.12654	-0.14059	-0.18275	-0.21370													
16	-0.11995	-0.13631	-0.17786														
8	-0.12260	-0.14196															
4	-0.14310																

(g) $\mathcal{P}^{\mathcal{C}}$ for Scene: [**Torus**] Algorithm: [**M-ERPT**] Metric: [**PSNR**] True GT: [**BDPT @ 524288 spp**].

Table 87

19.3 Veach Bidir

PT	2	4	8	16	32	64	128	256	512	1024	2048	4096	8192	16384	32768	65536	131072
GT (BDPT)																	
262144	0.00003	0.00004	0.00003	0.00003	0.00003	-0.00002	0.00006	0.00004	0.00008	0.00007	0.00002	0.00009	-0.00005	-0.00009	-0.00009	-0.00016	-0.00022
131072	0.00007	0.00006	0.00007	0.00005	0.00004	0.00002	0.00002	-0.00000	-0.00000	-0.00000	0.00006	-0.00007	-0.00005	-0.00005	-0.00022	-0.00035	
65536	0.00006	0.00006	0.00006	0.00009	0.00007	0.00007	0.00010	0.00006	-0.00003	0.00000	0.00009	0.00007	-0.00012	-0.00049	-0.00057		
32768	0.00007	0.00003	0.00006	0.00009	0.00000	0.00002	0.00004	0.00005	-0.00009	-0.00016	-0.00017	-0.00031	-0.00049	-0.00082			
16384	0.00014	0.00017	0.00020	0.00019	0.00020	0.00015	0.00005	0.00002	0.00006	-0.00006	-0.00016	-0.00036	-0.00073				
8192	0.00012	0.00009	0.00015	0.00015	0.00009	0.00023	0.00003	0.00005	0.00000	-0.00033	-0.00056	-0.00107					
4096	-0.00001	0.00004	-0.00003	-0.00004	-0.00020	-0.00024	-0.00049	-0.00074	-0.00070	-0.00082	-0.00089						
2048	0.00016	0.00009	0.00014	0.00008	0.00015	0.00013	-0.00045	-0.00047	-0.00049	-0.00154							
1024	0.00030	0.00031	0.00047	0.00062	0.00052	0.00058	-0.00030	-0.00012	-0.00014								
512	0.00032	0.00047	0.00061	0.00043	0.00033	0.00095	-0.00066	-0.00093									
256	0.00027	0.00027	0.00046	0.00030	-0.00098	0.00039	-0.00249										
128	0.00021	0.00014	0.00065	-0.00004	-0.00158	-0.00045											
64	0.00007	-0.00006	-0.00009	-0.00065	-0.00316												
32	-0.00051	-0.00127	-0.00222	-0.00386													
16	-0.00241	-0.00413	-0.00624														
8	-0.00626	-0.00994															
4	-0.01300																

(a) $\mathcal{P}^{\mathcal{C}}$ for Scene: [**Veach Bidir**] Algorithm: [**PT**] Metric: [**PSNR**] True GT: [**BDPT @ 524288 spp**].

BDPT	2	4	8	16	32	64	128	256	512	1024	2048	4096	8192	16384	32768	65536	131072
GT (BDPT)																	
262144	0.00004	0.00004	0.00006	0.00013	0.00009	0.00011	0.00028	-0.00027	0.00009	0.00187	-0.00038	-0.00566	-0.00067	0.00078	-0.00237	-0.00321	-0.01043
131072	0.00005	0.00007	0.00004	-0.00007	-0.00014	-0.00007	-0.00012	0.00001	-0.00108	-0.00176	-0.00401	-0.00244	-0.00465	-0.00406	-0.00511	-0.00837	
65536	0.00014	0.00012	0.00015	0.00023	0.00000	-0.00003	-0.00020	-0.00047	0.00053	-0.00210	-0.00266	-0.00697	-0.00547	-0.01422	-0.01519		
32768	0.00002	-0.00004	-0.00007	-0.00001	-0.00041	-0.00108	-0.00122	-0.00180	-0.00047	-0.00404	-0.00507	-0.02051	-0.02497	-0.02644			
16384	0.00025	0.00042	0.00052	0.00006	0.00131	0.00177	0.00268	0.00046	0.00205	-0.00290	-0.00679	-0.03276	-0.02652				
8192	0.00021	0.00027	0.00063	0.00039	0.00034	0.00086	0.00126	-0.00329	-0.00349	-0.01157	-0.02214	-0.04562					
4096	-0.00020	-0.00039	-0.00104	-0.00158	-0.00287	-0.00399	-0.00658	-0.00975	-0.01526	-0.02561	-0.03919						
2048	0.00014	0.00026	-0.00017	-0.00176	-0.00002	-0.00162	-0.00258	-0.01407	-0.00379	-0.03078							
1024	0.00085	0.00112	0.00168	0.00069	0.00225	0.00603	0.00646	-0.01123	-0.00902								
512	0.00116	0.00176	0.00233	0.00145	0.00152	0.00579	-0.00019	-0.02646									
256	0.00061	0.00093	0.00101	-0.00200	-0.00175	-0.00037	-0.01497										
128	0.00017	-0.00053	-0.00152	-0.01144	-0.00916	-0.00361											
64	-0.00203	-0.00370	-0.00745	-0.02110	-0.02660												
32	-0.00733	-0.01273	-0.02425	-0.04658													
16	-0.01877	-0.03047	-0.05040														
8	-0.03736	-0.06048															
4	-0.07021																

(b) $\mathcal{P}^{\mathcal{C}}$ for Scene: [**Veach Bidir**] Algorithm: [**BDPT**] Metric: [**PSNR**] True GT: [**BDPT @ 524288 spp**].

PSSMLT	2	4	8	16	32	64	128	256	512	1024	2048	4096	8192	16384
GT (BDPT)														
262144	0.00005	0.00006	0.00006	0.00007	0.00015	0.00011	0.00010	-0.00004	0.00011	0.00018	0.00089	0.00074	0.00015	0.00015
131072	0.00010	0.00006	0.00005	-0.00002	-0.00001	-0.00005	-0.00011	-0.00012	-0.00059	-0.00064	-0.00045	-0.00049	-0.00084	-0.00059
65536	0.00012	0.00012	0.00007	0.00011	0.00032	0.00033	0.00036	-0.00040	0.00045	-0.00032	-0.00069	-0.00095	-0.00136	-0.00179
32768	0.00010	0.00007	-0.00000	-0.00008	-0.00028	-0.00021	-0.00034	-0.00097	-0.00112	-0.00199	-0.00183	-0.00363	-0.00427	-0.00449
16384	0.00021	0.00035	0.00051	0.00072	0.00083	0.00138	0.00159	0.00136	-0.00283	-0.00426	-0.00615	-0.00596	-0.00683	
8192	0.00019	0.00024	0.00044	0.00065	0.00103	0.00116	0.00152	0.00047	-0.00336	-0.00487	-0.00808	-0.00986		
4096	-0.00017	-0.00050	-0.00092	-0.00155	-0.00191	-0.00292	-0.00443	-0.00888	-0.01395	-0.01438	-0.02015			
2048	0.00014	0.00018	0.00013	0.00005	-0.00056	-0.00031	-0.00058	-0.00535	-0.02034	-0.02301				
1024	0.00073	0.00104	0.00192	0.00317	0.00427	0.00609	0.00798	0.00133	-0.01575					
512	0.00077	0.00130	0.00227	0.00386	0.00454	0.00778	0.00983	-0.01119						
256	0.00061	0.00074	0.00096	0.00132	0.00055	0.00036	0.00107							
128	0.00009	-0.00040	-0.00121	-0.00229	-0.00504	-0.00773								
64	-0.00178	-0.00334	-0.00576	-0.01145	-0.01881									
32	-0.00745	-0.01319	-0.02047	-0.03559										
16	-0.01686	-0.02938	-0.04688											
8	-0.03363	-0.05574												
4	-0.06376													

(c) $\mathcal{P}^{\mathcal{C}}$ for Scene: [**Veach Bidir**] Algorithm: [**PSSMLT**] Metric: [**PSNR**] True GT: [**BDPT @ 524288 spp**].

MLT	2	4	8	16	32	64	128	256	512	1024	2048	4096	8192	16384
GT (BDPT)														
262144	0.00005	0.00004	0.00003	0.00003	0.00004	0.00009	0.00012	0.00011	0.00012	0.00011	0.00038	0.00043	0.00037	0.00034
131072	0.00013	0.00013	0.00013	0.00012	0.00012	0.00017	0.00013	0.00009	0.00001	-0.00011	-0.00015	-0.00012	-0.00021	-0.00026
65536	0.00016	0.00016	0.00017	0.00023	0.00025	0.00035	0.00038	0.00044	0.00052	0.00052	0.00069	0.00064	0.00060	0.00046
32768	0.00010	0.00008	0.00007	0.00014	0.00016	0.00022	0.00016	-0.00011	-0.00015	-0.00032	-0.00027	-0.00071	-0.00067	-0.00080
16384	0.00017	0.00019	0.00025	0.00038	0.00048	0.00067	0.00080	0.00098	0.00143	0.00194	0.00148	0.00204	0.00225	
8192	0.00017	0.00018	0.00027	0.00043	0.00036	0.00046	0.00088	0.00131	0.00151	0.00179	0.00217	0.00207		
4096	0.00013	0.00012	-0.00003	-0.00020	-0.00028	-0.00071	-0.00161	-0.00220	-0.00352	-0.00501	-0.00637			
2048	0.00028	0.00035	0.00044	0.00029	0.00026	0.00013	-0.00002	-0.00041	-0.00066	-0.00016				
1024	0.00035	0.00064	0.00093	0.00133	0.00114	0.00196	0.00330	0.00586	0.00766					
512	0.00067	0.00094	0.00145	0.00161	0.00129	0.00196	0.00323	0.00523						
256	0.00041	0.00060	0.00107	0.00142	0.00085	0.00073	0.00132							
128	0.00045	0.00060	0.00046	-0.00012	-0.00155	-0.00260								
64	0.00025	-0.00000	-0.00049	-0.00218	-0.00582									
32	-0.00081	-0.00170	-0.00345	-0.00823										
16	-0.00320	-0.00617	-0.01126											
8	-0.00707	-0.01280												
4	-0.01378													

(d) $\mathcal{P}^{\mathcal{C}}$ for Scene: [**Veach Bidir**] Algorithm: [**MLT**] Metric: [**PSNR**] True GT: [**BDPT @ 524288 spp**].

M-MLT	2	4	8	16	32	64	128	256	512	1024	2048	4096	8192	16384
GT (BDPT)														
262144	0.00007	0.00007	0.00008	0.00007	0.00012	0.00009	0.00011	0.00014	0.00019	0.00011	0.00012	0.00018	0.00017	0.00033
131072	0.00014	0.00013	0.00014	0.00014	0.00017	0.00011	0.00011	0.00004	0.00005	-0.00010	-0.00033	-0.00015	-0.00033	-0.00029
65536	0.00014	0.00013	0.00017	0.00013	0.00029	0.00019	0.00032	0.00038	0.00041	0.00024	0.00012	0.00038	0.00027	0.00037
32768	0.00014	0.00010	0.00012	0.00002	0.00010	0.00001	-0.00000	-0.00012	-0.00034	-0.00098	-0.00062	-0.00095	-0.00096	-0.00094
16384	0.00018	0.00018	0.00020	0.00042	0.00043	0.00063	0.00085	0.00113	0.00143	0.00206	0.00189	0.00259	0.00278	
8192	0.00023	0.00036	0.00035	0.00046	0.00060	0.00077	0.00079	0.00092	0.00158	0.00123	0.00107	0.00172		
4096	0.00008	-0.00008	-0.00010	-0.00034	-0.00061	-0.00096	-0.00170	-0.00237	-0.00384	-0.00526	-0.00642			
2048	0.00013	0.00004	0.00006	0.00018	0.00037	-0.00004	-0.00044	0.00002	-0.00037	-0.00122				
1024	0.00039	0.00068	0.00053	0.00115	0.00173	0.00293	0.00369	0.00585	0.00766					
512	0.00047	0.00090	0.00076	0.00161	0.00227	0.00333	0.00404	0.00633						
256	0.00044	0.00060	0.00043	0.00120	0.00142	0.00278	0.00233							
128	0.00036	0.00073	0.00028	0.00071	0.00015	-0.00024								
64	0.00027	0.00057	-0.00067	-0.00074	-0.00295									
32	-0.00071	-0.00153	-0.00368	-0.00716										
16	-0.00279	-0.00535	-0.01109											
8	-0.00708	-0.01270												
4	-0.01399													

(e) $\mathcal{P}^{\mathcal{C}}$ for Scene: [**Veach Bidir**] Algorithm: [**M-MLT**] Metric: [**PSNR**] True GT: [**BDPT @ 524288 spp**].

ERPT	2	4	8	16	32	64	128	256	512	1024	2048	4096	8192	16384	32768	65536	131072
GT (BDPT)																	
262144	0.00007	0.00006	0.00014	0.00009	0.00017	0.00011	0.00024	0.00005	0.00013	-0.00007	-0.00002	-0.00009	0.00011	-0.00012	-0.00002	0.00010	0.00013
131072	-0.00001	0.00006	-0.00004	-0.00003	0.00007	0.00014	-0.00009	0.00008	-0.00006	0.00004	0.00007	-0.00001	0.00003	-0.00004	0.00004	0.00013	
65536	0.00008	0.00024	0.00010	0.00016	0.00009	0.00016	0.00002	-0.00001	-0.00042	-0.00016	-0.00042	-0.00022	-0.00027	-0.00025	-0.00015		
32768	-0.00023	-0.00022	-0.00020	-0.00001	-0.00013	-0.00043	-0.00026	-0.00018	-0.00046	-0.00060	-0.00057	-0.00055	-0.00049	-0.00047			
16384	0.00062	0.00085	0.00101	0.00084	0.00104	-0.00053	0.00035	-0.00117	-0.00121	-0.00074	-0.00084	-0.00124	-0.00090				
8192	0.00091	0.00080	0.00074	0.00058	0.00027	-0.00166	-0.00025	-0.00022	-0.00112	-0.00140	-0.00156	-0.00164					
4096	-0.00244	-0.00180	-0.00239	-0.00190	-0.00225	-0.00152	-0.00202	-0.00169	-0.00366	-0.00236	-0.00255						
2048	-0.00052	-0.00057	0.00006	-0.00015	0.00011	-0.00278	-0.00157	-0.00392	-0.00466	-0.00443							
1024	0.00364	0.00384	0.00407	0.00388	0.00163	-0.00557	0.00073	-0.00370	-0.00625								
512	0.00406	0.00508	0.00474	0.00438	-0.00038	-0.01196	-0.00295	-0.00946									
256	0.00113	0.00160	0.00094	0.00079	-0.00567	-0.01793	-0.01196										
128	-0.00260	-0.00289	-0.00325	-0.00255	-0.01114	-0.03303											
64	-0.01561	-0.01452	-0.01656	-0.01584	-0.02658												
32	-0.04550	-0.04828	-0.04910	-0.04959													
16	-0.09417	-0.09339	-0.09661														
8	-0.15546	-0.15741															
4	-0.23726																

(f) $\mathcal{P}^{\mathcal{C}}$ for Scene: [**Veach Bidir**] Algorithm: [**ERPT**] Metric: [**PSNR**] True GT: [**BDPT @ 524288 spp**].

M-ERPT	2	4	8	16	32	64	128	256	512	1024	2048	4096	8192	16384	32768	65536	131072
GT (BDPT)																	
262144	0.00004	0.00016	0.00012	0.00016	0.00006	0.00005	-0.00015	0.00003	-0.00015	-0.00003	0.00002	0.00009	0.00001	0.00001	0.00000	-0.00000	0.00021
131072	-0.00003	0.00001	0.00003	-0.00006	0.00000	-0.00004	-0.00006	-0.00003	-0.00011	-0.00001	0.00003	0.00004	0.00010	0.00007	0.00008	0.00005	
65536	0.00013	0.00026	0.00003	0.00012	-0.00005	-0.00004	-0.00028	-0.00014	-0.00025	-0.00008	-0.00023	-0.00017	-0.00017	-0.00017	-0.00002		
32768	-0.00009	-0.00013	-0.00019	-0.00013	-0.00032	-0.00035	-0.00061	-0.00026	-0.00033	-0.00036	-0.00044	-0.00050	-0.00050	-0.00034			
16384	0.00097	0.00076	-0.00039	-0.00041	-0.00007	-0.00058	-0.00068	-0.00080	-0.00057	-0.00108	-0.00096	-0.00095	-0.00100				
8192	0.00063	0.00084	-0.00091	-0.00073	-0.00036	-0.00090	-0.00216	-0.00091	-0.00148	-0.00145	-0.00171	-0.00151					
4096	-0.00213	-0.00173	-0.00294	-0.00273	-0.00219	-0.00298	-0.00256	-0.00295	-0.00266	-0.00264	-0.00262						
2048	0.00070	-0.00008	-0.00373	-0.00384	-0.00316	-0.00429	-0.00309	-0.00408	-0.00280	-0.00490							
1024	0.00381	0.00480	-0.00590	-0.00363	-0.00222	-0.00567	-0.00754	-0.00613	-0.00650								
512	0.00474	0.00420	-0.01381	-0.00965	-0.00808	-0.01361	-0.01367	-0.01425									
256	0.00214	0.00255	-0.01840	-0.01738	-0.01414	-0.01961	-0.01939										
128	-0.00142	-0.00034	-0.03545	-0.03163	-0.02889	-0.03527											
64	-0.01316	-0.01367	-0.05292	-0.04942	-0.04738												
32	-0.04122	-0.04351	-0.07724	-0.07357													
16	-0.08865	-0.08831	-0.11790														
8	-0.14741	-0.14921															
4	-0.22804																

(g) $\mathcal{P}^{\mathcal{C}}$ for Scene: [**Veach Bidir**] Algorithm: [**M-ERPT**] Metric: [**PSNR**] True GT: [**BDPT @ 524288 spp**].

Table 88

19.4 Veach Door

PT	2	4	8	16	32	64	128	256	512	1024	2048	4096	8192	16384	32768	65536	131072
GT (BDPT)																	
262144	0.00004	0.00001	-0.00000	0.00001	-0.00003	-0.00000	-0.00003	-0.00005	-0.00005	-0.00010	-0.00011	-0.00016	-0.00031	-0.00054	-0.00087	-0.00195	-0.00274
131072	0.00007	-0.00000	-0.00000	-0.00002	-0.00006	-0.00002	-0.00008	-0.00014	-0.00015	-0.00019	-0.00029	-0.00054	-0.00078	-0.00136	-0.00268	-0.00433	
65536	0.00003	0.00001	0.00001	0.00002	-0.00006	-0.00009	-0.00011	-0.00023	-0.00030	-0.00043	-0.00074	-0.00107	-0.00188	-0.00340	-0.00573		
32768	-0.00007	-0.00009	-0.00001	-0.00007	-0.00013	-0.00013	-0.00032	-0.00055	-0.00055	-0.00085	-0.00139	-0.00255	-0.00405	-0.00725			
16384	-0.00016	-0.00012	-0.00008	-0.00012	-0.00015	-0.00020	-0.00045	-0.00068	-0.00120	-0.00172	-0.00267	-0.00475	-0.00828				
8192	-0.00042	-0.00023	-0.00014	-0.00010	-0.00021	-0.00051	-0.00087	-0.00119	-0.00210	-0.00355	-0.00569	-0.00972					
4096	-0.00076	-0.00049	-0.00032	-0.00055	-0.00081	-0.00124	-0.00159	-0.00284	-0.00428	-0.00741	-0.01147						
2048	-0.00152	-0.00090	-0.00045	-0.00116	-0.00144	-0.00235	-0.00334	-0.00556	-0.00878	-0.01359							
1024	-0.00319	-0.00185	-0.00154	-0.00201	-0.00310	-0.00492	-0.00748	-0.01196	-0.01727								
512	-0.00570	-0.00392	-0.00314	-0.00395	-0.00641	-0.00967	-0.01442	-0.02241									
256	-0.01020	-0.00668	-0.00629	-0.00771	-0.01294	-0.02004	-0.02862										
128	-0.01669	-0.01260	-0.01242	-0.01654	-0.02552	-0.03822											
64	-0.02766	-0.02342	-0.02227	-0.03261	-0.04973												
32	-0.04525	-0.04080	-0.04583	-0.06342													
16	-0.07528	-0.07170	-0.08657														
8	-0.12750	-0.13099															
4	-0.20376																

(a) $\mathcal{P}^{\mathcal{C}}$ for Scene: [**Veach Door**] Algorithm: [**PT**] Metric: [**PSNR**] True GT: [**BDPT @ 524288 spp**].

BDPT	2	4	8	16	32	64	128	256	512	1024	2048	4096	8192	16384	32768	65536	131072
GT (BDPT)																	
262144	0.00002	0.00001	-0.00001	0.00001	-0.00002	-0.00004	-0.00003	-0.00001	-0.00021	-0.00024	-0.00058	-0.00092	-0.00153	-0.00287	-0.00524	-0.00826	-0.01230
131072	0.00001	-0.00000	-0.00000	-0.00005	-0.00005	-0.00006	-0.00019	-0.00029	-0.00057	-0.00082	-0.00145	-0.00224	-0.00440	-0.00790	-0.01276	-0.02068	
65536	0.00003	0.00001	-0.00003	-0.00008	-0.00016	-0.00016	-0.00033	-0.00052	-0.00092	-0.00167	-0.00317	-0.00575	-0.01006	-0.01739	-0.02856		
32768	-0.00002	-0.00004	-0.00003	-0.00019	-0.00024	-0.00032	-0.00060	-0.00118	-0.00240	-0.00387	-0.00704	-0.01195	-0.02165	-0.03555			
16384	-0.00009	-0.00007	-0.00010	-0.00014	-0.00031	-0.00066	-0.00130	-0.00205	-0.00416	-0.00719	-0.01273	-0.02288	-0.03856				
8192	-0.00023	-0.00009	-0.00008	-0.00033	-0.00060	-0.00135	-0.00242	-0.00446	-0.00816	-0.01446	-0.02536	-0.04362					
4096	-0.00029	-0.00042	-0.00048	-0.00084	-0.00139	-0.00285	-0.00504	-0.00879	-0.01636	-0.02783	-0.04805						
2048	-0.00076	-0.00076	-0.00092	-0.00153	-0.00272	-0.00543	-0.00970	-0.01733	-0.02968	-0.05098							
1024	-0.00168	-0.00157	-0.00192	-0.00325	-0.00599	-0.01102	-0.01957	-0.03349	-0.05604								
512	-0.00357	-0.00285	-0.00430	-0.00665	-0.01170	-0.02164	-0.03729	-0.06128									
256	-0.00692	-0.00616	-0.00782	-0.01324	-0.02266	-0.04036	-0.06574										
128	-0.01263	-0.01203	-0.01667	-0.02759	-0.04545	-0.07512											
64	-0.02160	-0.02340	-0.03398	-0.05337	-0.08486												
32	-0.03952	-0.04457	-0.06394	-0.09935													
16	-0.07363	-0.08921	-0.12048														
8	-0.13097	-0.15972															
4	-0.22327																

(b) $\mathcal{P}^{\mathcal{C}}$ for Scene: [**Veach Door**] Algorithm: [**BDPT**] Metric: [**PSNR**] True GT: [**BDPT @ 524288 spp**].

PSSMLT	2	4	8	16	32	64	128	256	512	1024	2048	4096	8192	16384
GT (BDPT)														
262144	-0.00000	-0.00000	0.00001	0.00000	-0.00001	-0.00006	-0.00015	-0.00022	-0.00035	-0.00064	-0.00098	-0.00162	-0.00258	-0.00411
131072	-0.00001	-0.00002	-0.00007	-0.00006	-0.00015	-0.00020	-0.00033	-0.00052	-0.00076	-0.00132	-0.00255	-0.00435	-0.00696	-0.01014
65536	-0.00003	-0.00005	-0.00009	-0.00007	-0.00019	-0.00030	-0.00053	-0.00095	-0.00171	-0.00309	-0.00561	-0.00932	-0.01470	-0.02185
32768	-0.00006	-0.00010	-0.00013	-0.00025	-0.00047	-0.00085	-0.00138	-0.00237	-0.00416	-0.00703	-0.01196	-0.01953	-0.02997	-0.04182
16384	-0.00004	-0.00007	-0.00016	-0.00038	-0.00072	-0.00127	-0.00240	-0.00401	-0.00722	-0.01263	-0.02180	-0.03531	-0.05258	
8192	-0.00019	-0.00027	-0.00043	-0.00077	-0.00146	-0.00267	-0.00475	-0.00836	-0.01459	-0.02480	-0.04113	-0.06323		
4096	-0.00040	-0.00045	-0.00078	-0.00161	-0.00312	-0.00530	-0.00939	-0.01654	-0.02797	-0.04728	-0.07229			
2048	-0.00061	-0.00093	-0.00186	-0.00352	-0.00585	-0.01050	-0.01838	-0.03034	-0.05043	-0.07964				
1024	-0.00140	-0.00237	-0.00402	-0.00708	-0.01233	-0.02119	-0.03501	-0.05748	-0.08897					
512	-0.00296	-0.00480	-0.00835	-0.01439	-0.02390	-0.04018	-0.06409	-0.09921						
256	-0.00658	-0.01018	-0.01636	-0.02795	-0.04529	-0.07221	-0.10915							
128	-0.01326	-0.02062	-0.03253	-0.05228	-0.08179	-0.12502								
64	-0.02758	-0.04039	-0.06254	-0.09611	-0.14196									
32	-0.05074	-0.07785	-0.11622	-0.16654										
16	-0.09804	-0.14117	-0.20108											
8	-0.17632	-0.24342												
4	-0.29995													

(c) $\mathcal{P}^{\mathcal{C}}$ for Scene: [**Veach Door**] Algorithm: [**PSSMLT**] Metric: [**PSNR**] True GT: [**BDPT @ 524288 spp**].

MLT	2	4	8	16	32	64	128	256	512	1024	2048	4096	8192	16384
GT (BDPT)														
262144	0.00002	0.00002	-0.00001	-0.00003	-0.00003	-0.00009	-0.00011	-0.00031	-0.00031	-0.00057	-0.00107	-0.00194	-0.00310	-0.00403
131072	0.00002	-0.00004	-0.00008	-0.00008	-0.00014	-0.00022	-0.00036	-0.00060	-0.00092	-0.00156	-0.00316	-0.00497	-0.00700	-0.01059
65536	-0.00001	-0.00003	-0.00013	-0.00013	-0.00027	-0.00057	-0.00076	-0.00133	-0.00220	-0.00365	-0.00626	-0.01085	-0.01558	-0.02261
32768	0.00002	-0.00012	-0.00020	-0.00017	-0.00051	-0.00084	-0.00149	-0.00254	-0.00438	-0.00743	-0.01309	-0.02204	-0.03178	-0.04304
16384	-0.00002	-0.00012	-0.00025	-0.00044	-0.00083	-0.00146	-0.00281	-0.00519	-0.00883	-0.01502	-0.02609	-0.04016	-0.05551	
8192	-0.00002	-0.00016	-0.00039	-0.00089	-0.00174	-0.00293	-0.00600	-0.01023	-0.01734	-0.02993	-0.04733	-0.07083		
4096	-0.00020	-0.00063	-0.00110	-0.00182	-0.00356	-0.00615	-0.01121	-0.02069	-0.03322	-0.05322	-0.08223			
2048	-0.00052	-0.00094	-0.00183	-0.00382	-0.00658	-0.01206	-0.02096	-0.03623	-0.05926	-0.08974				
1024	-0.00134	-0.00242	-0.00454	-0.00805	-0.01426	-0.02450	-0.04044	-0.06681	-0.10051					
512	-0.00276	-0.00497	-0.00881	-0.01640	-0.02717	-0.04482	-0.07393	-0.11335						
256	-0.00573	-0.01001	-0.01810	-0.03106	-0.05004	-0.07955	-0.12238							
128	-0.01106	-0.02079	-0.03602	-0.05911	-0.09155	-0.13679								
64	-0.02571	-0.04086	-0.06958	-0.10719	-0.15694									
32	-0.04942	-0.07993	-0.12627	-0.18373										
16	-0.09484	-0.14690	-0.21803											
8	-0.17628	-0.25430												
4	-0.30061													

(d) $\mathcal{P}^{\mathcal{C}}$ for Scene: [**Veach Door**] Algorithm: [**MLT**] Metric: [**PSNR**] True GT: [**BDPT @ 524288 spp**].

M-MLT	2	4	8	16	32	64	128	256	512	1024	2048	4096	8192	16384
GT (BDPT)														
262144	0.00000	-0.00000	-0.00003	0.00000	0.00007	-0.00007	-0.00012	-0.00025	-0.00051	-0.00053	-0.00118	-0.00173	-0.00261	-0.00383
131072	-0.00003	-0.00002	-0.00006	-0.00008	-0.00012	-0.00029	-0.00045	-0.00055	-0.00108	-0.00155	-0.00307	-0.00437	-0.00723	-0.00906
65536	-0.00004	-0.00003	-0.00011	-0.00011	-0.00018	-0.00056	-0.00074	-0.00146	-0.00230	-0.00376	-0.00666	-0.00952	-0.01635	-0.02045
32768	-0.00005	-0.00011	-0.00025	-0.00022	-0.00050	-0.00084	-0.00169	-0.00249	-0.00457	-0.00759	-0.01390	-0.01969	-0.03151	-0.03915
16384	-0.00008	-0.00008	-0.00026	-0.00048	-0.00103	-0.00145	-0.00265	-0.00500	-0.00877	-0.01466	-0.02538	-0.03574	-0.05505	
8192	-0.00007	-0.00023	-0.00045	-0.00098	-0.00180	-0.00322	-0.00520	-0.00985	-0.01727	-0.02823	-0.04737	-0.06434		
4096	-0.00049	-0.00062	-0.00112	-0.00199	-0.00384	-0.00628	-0.01238	-0.01972	-0.03287	-0.05217	-0.08185			
2048	-0.00070	-0.00098	-0.00225	-0.00356	-0.00703	-0.01233	-0.02210	-0.03674	-0.05857	-0.08838				
1024	-0.00150	-0.00266	-0.00503	-0.00790	-0.01512	-0.02493	-0.04128	-0.06605	-0.10118					
512	-0.00305	-0.00505	-0.00947	-0.01616	-0.02834	-0.04659	-0.07507	-0.11239						
256	-0.00674	-0.01077	-0.01884	-0.03197	-0.05262	-0.08229	-0.12384							
128	-0.01385	-0.02233	-0.03717	-0.06050	-0.09462	-0.14003								
64	-0.02706	-0.04366	-0.07160	-0.10938	-0.16137									
32	-0.05520	-0.08367	-0.13149	-0.18713										
16	-0.10373	-0.15104	-0.22398											
8	-0.18679	-0.25982												
4	-0.31393													

(e) $\mathcal{P}^{\mathcal{C}}$ for Scene: [**Veach Door**] Algorithm: [**M-MLT**] Metric: [**PSNR**] True GT: [**BDPT @ 524288 spp**].

ERPT	2	4	8	16	32	64	128	256	512	1024	2048	4096	8192	16384	32768	65536	131072
GT (BDPT)																	
262144	-0.00008	-0.00015	-0.00017	-0.00008	-0.00020	-0.00014	-0.00027	-0.00019	-0.00023	-0.00015	-0.00020	-0.00018	-0.00027	-0.00017	-0.00028	-0.00021	-0.00015
131072	-0.00046	-0.00045	-0.00055	-0.00050	-0.00060	-0.00056	-0.00067	-0.00059	-0.00064	-0.00058	-0.00049	-0.00059	-0.00067	-0.00040	-0.00045	-0.00059	
65536	-0.00087	-0.00090	-0.00121	-0.00105	-0.00105	-0.00119	-0.00113	-0.00119	-0.00116	-0.00109	-0.00093	-0.00122	-0.00110	-0.00103	-0.00109		
32768	-0.00187	-0.00185	-0.00229	-0.00220	-0.00245	-0.00218	-0.00223	-0.00213	-0.00230	-0.00229	-0.00200	-0.00224	-0.00235	-0.00213			
16384	-0.00296	-0.00348	-0.00370	-0.00406	-0.00426	-0.00426	-0.00403	-0.00422	-0.00378	-0.00440	-0.00413	-0.00432	-0.00407				
8192	-0.00600	-0.00592	-0.00767	-0.00813	-0.00791	-0.00849	-0.00830	-0.00863	-0.00768	-0.00857	-0.00824	-0.00817					
4096	-0.01274	-0.01346	-0.01473	-0.01590	-0.01573	-0.01625	-0.01593	-0.01668	-0.01594	-0.01618	-0.01638						
2048	-0.02426	-0.02459	-0.02818	-0.03006	-0.03004	-0.03006	-0.02950	-0.03049	-0.02980	-0.03013							
1024	-0.04507	-0.04722	-0.05206	-0.05564	-0.05505	-0.05647	-0.05483	-0.05589	-0.05448								
512	-0.08033	-0.08279	-0.09236	-0.09547	-0.09599	-0.09719	-0.09533	-0.09782									
256	-0.13155	-0.13471	-0.14579	-0.15274	-0.15326	-0.15469	-0.15381										
128	-0.20553	-0.21177	-0.22580	-0.23334	-0.23404	-0.23472											
64	-0.30117	-0.30606	-0.32272	-0.33144	-0.33169												
32	-0.41970	-0.42585	-0.44333	-0.45255													
16	-0.56199	-0.56901	-0.58627														
8	-0.73179	-0.73954															
4	-0.91919																

(f) $\mathcal{P}^{\mathcal{C}}$ for Scene: [**Veach Door**] Algorithm: [**ERPT**] Metric: [**PSNR**] True GT: [**BDPT @ 524288 spp**].

M-ERPT	2	4	8	16	32	64	128	256	512	1024	2048	4096	8192	16384	32768	65536	131072
GT (BDPT)																	
262144	-0.00004	-0.00015	-0.00016	-0.00014	-0.00019	-0.00017	-0.00022	-0.00013	-0.00018	-0.00026	-0.00007	-0.00014	-0.00018	-0.00012	-0.00024	-0.00021	-0.00015
131072	-0.00033	-0.00035	-0.00050	-0.00045	-0.00038	-0.00049	-0.00051	-0.00048	-0.00042	-0.00057	-0.00044	-0.00052	-0.00056	-0.00042	-0.00044	-0.00053	
65536	-0.00084	-0.00091	-0.00085	-0.00094	-0.00106	-0.00102	-0.00118	-0.00108	-0.00093	-0.00096	-0.00089	-0.00108	-0.00121	-0.00092	-0.00108		
32768	-0.00142	-0.00181	-0.00172	-0.00186	-0.00221	-0.00200	-0.00196	-0.00190	-0.00214	-0.00188	-0.00198	-0.00201	-0.00206	-0.00197			
16384	-0.00260	-0.00321	-0.00334	-0.00378	-0.00365	-0.00389	-0.00372	-0.00369	-0.00346	-0.00370	-0.00367	-0.00392	-0.00389				
8192	-0.00547	-0.00635	-0.00670	-0.00703	-0.00723	-0.00696	-0.00721	-0.00758	-0.00723	-0.00720	-0.00775	-0.00748					
4096	-0.01124	-0.01315	-0.01408	-0.01371	-0.01453	-0.01401	-0.01465	-0.01463	-0.01503	-0.01474	-0.01436						
2048	-0.02185	-0.02504	-0.02665	-0.02617	-0.02680	-0.02703	-0.02739	-0.02788	-0.02761	-0.02725							
1024	-0.04105	-0.04662	-0.04986	-0.04912	-0.05083	-0.05049	-0.05181	-0.05213	-0.05105								
512	-0.07358	-0.08182	-0.08505	-0.08668	-0.08791	-0.08809	-0.08980	-0.08993									
256	-0.12324	-0.13457	-0.13967	-0.14157	-0.14249	-0.14241	-0.14459										
128	-0.19454	-0.20836	-0.21606	-0.21900	-0.22126	-0.21932											
64	-0.28689	-0.30496	-0.31154	-0.31588	-0.31796												
32	-0.40486	-0.42397	-0.43170	-0.43592													
16	-0.54560	-0.56548	-0.57564														
8	-0.71474	-0.73648															
4	-0.90208																

(g) $\mathcal{P}^{\mathcal{C}}$ for Scene: [**Veach Door**] Algorithm: [**M-ERPT**] Metric: [**PSNR**] True GT: [**BDPT @ 524288 spp**].

Table 89

19.5 Sponza

PT	2	4	8	16	32	64	128	256	512	1024	2048	4096	8192
GT (PT)													
16384	-0.00004	-0.00004	-0.00006	-0.00016	-0.00018	-0.00031	-0.00040	-0.00127	-0.00187	-0.00321	-0.00609	-0.01035	-0.01629
8192	-0.00002	-0.00011	-0.00015	-0.00025	-0.00065	-0.00099	-0.00145	-0.00309	-0.00584	-0.00976	-0.01683	-0.02790	
4096	-0.00016	-0.00032	-0.00049	-0.00076	-0.00142	-0.00227	-0.00368	-0.00719	-0.01294	-0.02148	-0.03651		
2048	-0.00031	-0.00049	-0.00080	-0.00130	-0.00275	-0.00457	-0.00790	-0.01480	-0.02522	-0.04235			
1024	-0.00072	-0.00129	-0.00179	-0.00315	-0.00554	-0.00985	-0.01687	-0.02880	-0.04826				
512	-0.00167	-0.00264	-0.00379	-0.00654	-0.01096	-0.01867	-0.03279	-0.05350					
256	-0.00296	-0.00465	-0.00718	-0.01247	-0.02167	-0.03604	-0.05925						
128	-0.00600	-0.00897	-0.01452	-0.02442	-0.04148	-0.06791							
64	-0.01134	-0.01765	-0.02871	-0.04748	-0.07635								
32	-0.02227	-0.03420	-0.05276	-0.08551									
16	-0.04205	-0.06408	-0.09688										
8	-0.07530	-0.11377											
4	-0.12563												

(a) $\mathcal{P}^{\mathcal{C}}$ for Scene: [**Sponza**] Algorithm: [**PT**] Metric: [**PSNR**] True GT: [**PT @ 32768 spp**].

BDPT	2	4	8	16	32	64	128	256	512	1024	2048	4096	8192
GT (PT)													
16384	0.00004	0.00004	-0.00004	-0.00007	-0.00013	-0.00017	-0.00042	-0.00077	-0.00161	-0.00310	-0.00469	-0.00817	-0.01379
8192	-0.00005	-0.00005	-0.00017	-0.00020	-0.00046	-0.00083	-0.00130	-0.00250	-0.00455	-0.00841	-0.01375	-0.02353	
4096	-0.00008	-0.00014	-0.00038	-0.00058	-0.00118	-0.00177	-0.00323	-0.00574	-0.01020	-0.01859	-0.03133		
2048	-0.00035	-0.00049	-0.00083	-0.00123	-0.00220	-0.00379	-0.00693	-0.01227	-0.02143	-0.03672			
1024	-0.00057	-0.00069	-0.00172	-0.00255	-0.00452	-0.00779	-0.01352	-0.02381	-0.04122				
512	-0.00108	-0.00218	-0.00296	-0.00553	-0.00892	-0.01543	-0.02735	-0.04616					
256	-0.00236	-0.00375	-0.00605	-0.01013	-0.01759	-0.02900	-0.05100						
128	-0.00498	-0.00762	-0.01253	-0.01947	-0.03401	-0.05714							
64	-0.00885	-0.01466	-0.02350	-0.03831	-0.06348								
32	-0.01781	-0.02982	-0.04461	-0.07134									
16	-0.03547	-0.05207	-0.08248										
8	-0.06452	-0.09554											
4	-0.10867												

(b) $\mathcal{P}^{\mathcal{C}}$ for Scene: [**Sponza**] Algorithm: [**BDPT**] Metric: [**PSNR**] True GT: [**PT @ 32768 spp**].

PSSMLT	2	4	8	16	32	64	128	256	512	1024	2048	4096	8192
GT (PT)													
16384	-0.00004	-0.00009	-0.00005	-0.00010	-0.00014	-0.00015	-0.00039	-0.00056	-0.00100	-0.00130	-0.00150	-0.00181	-0.00188
8192	-0.00015	-0.00017	-0.00028	-0.00047	-0.00054	-0.00076	-0.00140	-0.00190	-0.00289	-0.00368	-0.00451	-0.00535	
4096	-0.00021	-0.00038	-0.00055	-0.00082	-0.00134	-0.00197	-0.00323	-0.00470	-0.00676	-0.00852	-0.01021		
2048	-0.00044	-0.00070	-0.00104	-0.00167	-0.00270	-0.00394	-0.00655	-0.00992	-0.01403	-0.01821			
1024	-0.00089	-0.00129	-0.00215	-0.00354	-0.00525	-0.00850	-0.01339	-0.01999	-0.02822				
512	-0.00211	-0.00319	-0.00468	-0.00682	-0.01121	-0.01697	-0.02629	-0.03880					
256	-0.00390	-0.00577	-0.00893	-0.01385	-0.02189	-0.03317	-0.04955						
128	-0.00842	-0.01192	-0.01774	-0.02709	-0.04236	-0.06220							
64	-0.01547	-0.02291	-0.03506	-0.05245	-0.07740								
32	-0.02939	-0.04277	-0.06291	-0.09320									
16	-0.05666	-0.08091	-0.11569										
8	-0.09997	-0.13804											
4	-0.16443												

(c) $\mathcal{P}^{\mathcal{C}}$ for Scene: [**Sponza**] Algorithm: [**PSSMLT**] Metric: [**PSNR**] True GT: [**PT @ 32768 spp**].

MLT	2	4	8	16	32	64	128	256	512	1024	2048	4096	8192
GT (PT)													
16384	-0.00007	-0.00010	-0.00011	-0.00015	-0.00033	-0.00041	-0.00063	-0.00109	-0.00134	-0.00162	-0.00197	-0.00194	-0.00183
8192	-0.00010	-0.00013	-0.00045	-0.00057	-0.00095	-0.00141	-0.00263	-0.00320	-0.00429	-0.00491	-0.00566	-0.00541	
4096	-0.00034	-0.00057	-0.00099	-0.00148	-0.00241	-0.00358	-0.00551	-0.00762	-0.00962	-0.01143	-0.01209		
2048	-0.00063	-0.00105	-0.00180	-0.00287	-0.00519	-0.00799	-0.01189	-0.01597	-0.02020	-0.02311			
1024	-0.00126	-0.00230	-0.00356	-0.00623	-0.01075	-0.01678	-0.02436	-0.03196	-0.03952				
512	-0.00266	-0.00448	-0.00729	-0.01233	-0.02093	-0.03128	-0.04581	-0.05903					
256	-0.00493	-0.00896	-0.01444	-0.02547	-0.04000	-0.05951	-0.08116						
128	-0.00987	-0.01676	-0.02847	-0.04654	-0.07320	-0.10489							
64	-0.01827	-0.03214	-0.05316	-0.08385	-0.12399								
32	-0.03524	-0.05898	-0.09639	-0.14316									
16	-0.06468	-0.10745	-0.16285										
8	-0.11397	-0.17995											
4	-0.18668												

(d) $\mathcal{P}^{\mathcal{C}}$ for Scene: [**Sponza**] Algorithm: [**MLT**] Metric: [**PSNR**] True GT: [**PT @ 32768 spp**].

M-MLT	2	4	8	16	32	64	128	256	512	1024	2048	4096	8192
GT (PT)													
16384	-0.00010	-0.00007	-0.00017	-0.00011	-0.00036	-0.00042	-0.00084	-0.00121	-0.00137	-0.00172	-0.00176	-0.00184	-0.00192
8192	-0.00005	-0.00017	-0.00034	-0.00056	-0.00112	-0.00169	-0.00257	-0.00341	-0.00442	-0.00475	-0.00514	-0.00529	
4096	-0.00038	-0.00044	-0.00079	-0.00135	-0.00257	-0.00407	-0.00565	-0.00817	-0.00967	-0.01134	-0.01190		
2048	-0.00064	-0.00094	-0.00184	-0.00320	-0.00525	-0.00813	-0.01234	-0.01661	-0.02027	-0.02282			
1024	-0.00158	-0.00206	-0.00372	-0.00636	-0.01034	-0.01693	-0.02425	-0.03270	-0.03938				
512	-0.00304	-0.00424	-0.00712	-0.01255	-0.02098	-0.03190	-0.04682	-0.06060					
256	-0.00618	-0.00781	-0.01445	-0.02483	-0.03964	-0.05941	-0.08284						
128	-0.01199	-0.01623	-0.02831	-0.04743	-0.07354	-0.10455							
64	-0.02282	-0.03103	-0.05357	-0.08561	-0.12677								
32	-0.04230	-0.05826	-0.09530	-0.14550									
16	-0.07906	-0.10610	-0.16493										
8	-0.13548	-0.17784											
4	-0.21632												

(e) $\mathcal{P}^{\mathcal{C}}$ for Scene: [**Sponza**] Algorithm: [**M-MLT**] Metric: [**PSNR**] True GT: [**PT @ 32768 spp**].

ERPT	2	4	8	16	32	64	128	256	512	1024	2048	4096	8192
GT (PT)													
16384	-0.00112	-0.00101	-0.00086	-0.00077	-0.00102	-0.00107	-0.00114	-0.00098	-0.00100	-0.00104	-0.00117	-0.00098	-0.00090
8192	-0.00281	-0.00284	-0.00292	-0.00277	-0.00305	-0.00303	-0.00291	-0.00280	-0.00289	-0.00270	-0.00287	-0.00302	
4096	-0.00625	-0.00633	-0.00641	-0.00669	-0.00684	-0.00702	-0.00665	-0.00667	-0.00665	-0.00686	-0.00661		
2048	-0.01286	-0.01354	-0.01375	-0.01374	-0.01389	-0.01427	-0.01404	-0.01422	-0.01446	-0.01387			
1024	-0.02600	-0.02715	-0.02773	-0.02750	-0.02828	-0.02827	-0.02822	-0.02740	-0.02773				
512	-0.04909	-0.05093	-0.05106	-0.05176	-0.05201	-0.05296	-0.05222	-0.05233					
256	-0.08633	-0.08885	-0.09055	-0.09118	-0.09187	-0.09176	-0.09165						
128	-0.14505	-0.14857	-0.15006	-0.15169	-0.15180	-0.15221							
64	-0.22186	-0.22658	-0.22909	-0.23050	-0.23069								
32	-0.32066	-0.32465	-0.32650	-0.32888									
16	-0.43795	-0.44375	-0.44542										
8	-0.56994	-0.57522											
4	-0.71026												

(f) $\mathcal{P}^{\mathcal{C}}$ for Scene: [**Sponza**] Algorithm: [**ERPT**] Metric: [**PSNR**] True GT: [**PT @ 32768 spp**].

M-ERPT	2	4	8	16	32	64	128	256	512	1024	2048	4096	8192
GT (PT)													
16384	-0.00091	-0.00090	-0.00085	-0.00100	-0.00106	-0.00079	-0.00088	-0.00079	-0.00094	-0.00078	-0.00099	-0.00086	-0.00082
8192	-0.00262	-0.00293	-0.00275	-0.00284	-0.00281	-0.00283	-0.00267	-0.00269	-0.00265	-0.00270	-0.00277	-0.00271	
4096	-0.00623	-0.00648	-0.00654	-0.00650	-0.00671	-0.00628	-0.00649	-0.00618	-0.00602	-0.00631	-0.00633		
2048	-0.01268	-0.01305	-0.01338	-0.01303	-0.01391	-0.01336	-0.01288	-0.01323	-0.01363	-0.01318			
1024	-0.02588	-0.02528	-0.02656	-0.02623	-0.02691	-0.02660	-0.02706	-0.02751	-0.02702				
512	-0.04748	-0.04904	-0.04950	-0.04972	-0.05107	-0.05049	-0.04996	-0.05009					
256	-0.08346	-0.08710	-0.08779	-0.08747	-0.08989	-0.08910	-0.08740						
128	-0.14163	-0.14555	-0.14591	-0.14515	-0.14896	-0.14784							
64	-0.21824	-0.22165	-0.22338	-0.22460	-0.22656								
32	-0.31536	-0.32031	-0.32196	-0.32285									
16	-0.43278	-0.43818	-0.44043										
8	-0.56464	-0.56978											
4	-0.70530												

(g) $\mathcal{P}^{\mathcal{C}}$ for Scene: [**Sponza**] Algorithm: [**M-ERPT**] Metric: [**PSNR**] True GT: [**PT @ 32768 spp**].

Table 90

20 UQI

20.1 Cornell Box

PT	2	4	8	16	32	64	128	256	512	1024	2048	4096	8192
GT (PT)													
16384	0.00045	-0.00000	-0.00003	0.00101	0.00065	-0.00220	-0.00152	-0.00013	-0.00286	-0.00492	-0.00653	-0.00700	-0.01050
8192	-0.00224	-0.00203	0.00118	-0.00028	0.00066	-0.00142	-0.00354	-0.00495	-0.00797	-0.01128	-0.01802	-0.02226	
4096	-0.00214	-0.00103	0.00055	-0.00078	-0.00150	-0.00223	-0.00882	-0.01442	-0.02135	-0.02892	-0.03688		
2048	-0.00489	0.00179	-0.00123	-0.00214	-0.00463	-0.01120	-0.01769	-0.02873	-0.04304	-0.05899			
1024	-0.00459	0.00004	-0.00494	-0.00771	-0.01309	-0.02426	-0.03555	-0.05424	-0.08314				
512	-0.00422	-0.00254	-0.00473	-0.01143	-0.02971	-0.04681	-0.07198	-0.10245					
256	-0.00221	-0.00434	-0.00681	-0.02633	-0.03947	-0.08650	-0.13709						
128	-0.00973	-0.02194	-0.03682	-0.05850	-0.09301	-0.16489							
64	-0.03318	-0.03267	-0.06184	-0.10788	-0.17302								
32	-0.02107	-0.06130	-0.10794	-0.19288									
16	-0.05924	-0.12770	-0.18952										
8	-0.11275	-0.21423											
4	-0.17198												

(a) $\mathcal{P}^{\mathcal{C}}$ for Scene: **[Cornell Box]** Algorithm: **[PT]** Metric: **[UQI]** True GT: **[PT @ 32768 spp]**.

BDPT	2	4	8	16	32	64	128	256	512	1024	2048	4096	8192
GT (PT)													
16384	-0.00016	-0.00047	0.00102	-0.00101	-0.00062	-0.00239	-0.00299	-0.00358	-0.00561	-0.00638	-0.00856	-0.00961	-0.01241
8192	-0.00128	0.00072	-0.00184	-0.00347	-0.00328	-0.00506	-0.00919	-0.01141	-0.01443	-0.01824	-0.02392	-0.02750	
4096	-0.00058	-0.00278	-0.00242	-0.00308	-0.00782	-0.01366	-0.01787	-0.02669	-0.03290	-0.04273	-0.05302		
2048	-0.00196	0.00037	-0.00419	-0.00950	-0.01626	-0.02709	-0.03664	-0.05200	-0.06645	-0.08044			
1024	-0.00876	-0.00863	-0.01262	-0.01911	-0.03178	-0.04773	-0.07081	-0.09589	-0.11862				
512	-0.00671	-0.01724	-0.02504	-0.04182	-0.06313	-0.09421	-0.12735	-0.16702					
256	-0.01158	-0.01620	-0.03499	-0.07936	-0.11135	-0.16372	-0.23092						
128	-0.02239	-0.05105	-0.07984	-0.12901	-0.21362	-0.29163							
64	-0.02999	-0.07467	-0.16077	-0.22912	-0.33511								
32	-0.08393	-0.16007	-0.25384	-0.36224									
16	-0.14569	-0.25068	-0.37830										
8	-0.22923	-0.38538											
4	-0.34799												

(b) $\mathcal{P}^{\mathcal{C}}$ for Scene: **[Cornell Box]** Algorithm: **[BDPT]** Metric: **[UQI]** True GT: **[PT @ 32768 spp]**.

PSSMLT	2	4	8	16	32	64	128	256	512	1024	2048	4096	8192
GT (PT)													
16384	-0.00203	-0.00093	-0.00011	0.00100	0.00012	-0.00216	-0.00252	-0.00291	-0.00417	-0.00558	-0.00709	-0.00896	-0.00891
8192	-0.00066	-0.00103	0.00035	-0.00006	-0.00186	-0.00443	-0.00642	-0.00994	-0.01230	-0.01636	-0.02000	-0.02304	
4096	-0.00148	-0.00073	-0.00217	-0.00320	-0.00606	-0.01199	-0.01808	-0.02360	-0.03099	-0.03790	-0.04487		
2048	-0.00329	-0.00195	-0.00533	-0.00859	-0.01682	-0.02386	-0.03463	-0.04675	-0.06025	-0.07391			
1024	-0.00493	-0.00680	-0.01261	-0.01887	-0.02804	-0.04615	-0.06651	-0.08832	-0.10937				
512	-0.00996	-0.01054	-0.01803	-0.03633	-0.06138	-0.08955	-0.12358	-0.16021					
256	-0.01374	-0.02921	-0.04883	-0.07513	-0.11783	-0.16915	-0.21850						
128	-0.03938	-0.06316	-0.09298	-0.14912	-0.21323	-0.28897							
64	-0.06167	-0.09497	-0.15676	-0.24739	-0.34778								
32	-0.09743	-0.17093	-0.25287	-0.38379									
16	-0.16537	-0.28268	-0.42135										
8	-0.30267	-0.44423											
4	-0.45073												

(c) $\mathcal{P}^{\mathcal{C}}$ for Scene: **[Cornell Box]** Algorithm: **[PSSMLT]** Metric: **[UQI]** True GT: **[PT @ 32768 spp]**.

MLT	2	4	8	16	32	64	128	256	512	1024	2048	4096	8192
GT (PT)													
16384	0.00085	-0.00041	0.00023	-0.00041	0.00006	-0.00029	-0.00013	-0.00109	-0.00161	-0.00245	-0.00337	-0.00593	-0.00724
8192	-0.00054	-0.00104	-0.00036	-0.00021	-0.00001	-0.00096	-0.00231	-0.00544	-0.00734	-0.01020	-0.01364	-0.01650	
4096	0.00161	-0.00043	-0.00022	-0.00198	-0.00526	-0.00657	-0.00871	-0.01130	-0.01817	-0.02285	-0.03109		
2048	0.00272	0.00275	0.00473	0.00405	-0.00115	-0.00559	-0.01353	-0.02319	-0.03438	-0.04463			
1024	-0.00149	-0.00158	-0.00063	-0.00311	-0.01185	-0.01912	-0.02979	-0.04585	-0.06588				
512	-0.01058	-0.00491	-0.00696	-0.01503	-0.02466	-0.04174	-0.06067	-0.09018					
256	-0.00598	-0.01288	-0.01769	-0.02817	-0.04709	-0.07155	-0.11053						
128	-0.00753	-0.00327	-0.00570	-0.03101	-0.06605	-0.11522							
64	-0.00541	-0.02417	-0.05183	-0.07746	-0.11861								
32	-0.02788	-0.04909	-0.06389	-0.12967									
16	-0.05453	-0.07217	-0.13180										
8	-0.05588	-0.13829											
4	-0.16160												

(d) $\mathcal{P}^{\mathcal{C}}$ for Scene: **[Cornell Box]** Algorithm: **[MLT]** Metric: **[UQI]** True GT: **[PT @ 32768 spp]**.

M-MLT	2	4	8	16	32	64	128	256	512	1024	2048	4096	8192
GT (PT)													
16384	-0.00079	0.00102	0.00124	0.00114	0.00222	-0.00007	-0.00076	-0.00121	-0.00123	-0.00388	-0.00474	-0.00545	-0.00739
8192	-0.00160	0.00066	0.00023	-0.00061	-0.00087	-0.00314	-0.00237	-0.00494	-0.00905	-0.01160	-0.01493	-0.01696	
4096	-0.00231	-0.00083	-0.00210	-0.00245	-0.00246	-0.00602	-0.00747	-0.01244	-0.01725	-0.02446	-0.03258		
2048	-0.00168	-0.00126	-0.00138	-0.00390	-0.00571	-0.00880	-0.01579	-0.02412	-0.03478	-0.04878			
1024	-0.00105	0.00060	-0.00075	-0.00581	-0.01240	-0.02152	-0.03549	-0.05182	-0.06929				
512	0.00223	0.00036	-0.00745	-0.01448	-0.01919	-0.03678	-0.06064	-0.09174					
256	-0.00978	-0.01493	-0.02019	-0.03084	-0.04780	-0.07939	-0.12326						
128	-0.01740	-0.00911	-0.02395	-0.04829	-0.08472	-0.13662							
64	0.01371	-0.01237	-0.03878	-0.07468	-0.12551								
32	-0.02944	-0.04493	-0.07936	-0.14394									
16	-0.05358	-0.11376	-0.15531										
8	-0.11680	-0.14511											
4	-0.16543												

(e) $\mathcal{P}^{\mathcal{C}}$ for Scene: **[Cornell Box]** Algorithm: **[M-MLT]** Metric: **[UQI]** True GT: **[PT @ 32768 spp]**.

ERPT	2	4	8	16	32	64	128	256	512	1024	2048	4096	8192
GT (PT)													
16384	-0.00042	-0.00334	-0.00035	-0.00123	-0.00013	-0.00202	-0.00173	-0.00239	-0.00234	-0.00115	-0.00052	-0.00077	-0.00253
8192	-0.00407	-0.00443	-0.00531	-0.00227	-0.00372	-0.00412	-0.00325	-0.00538	-0.00342	-0.00384	-0.00377	-0.00360	
4096	-0.00861	-0.01161	-0.00809	-0.00646	-0.00785	-0.00949	-0.00946	-0.00892	-0.00829	-0.00801	-0.00974		
2048	-0.01807	-0.01853	-0.01893	-0.01543	-0.01752	-0.01902	-0.01643	-0.01596	-0.01607	-0.01811			
1024	-0.03574	-0.03076	-0.02954	-0.03250	-0.03032	-0.03695	-0.03557	-0.03438	-0.03573				
512	-0.06371	-0.06356	-0.06478	-0.06397	-0.06552	-0.07310	-0.06537	-0.06812					
256	-0.11798	-0.12762	-0.12059	-0.11814	-0.11986	-0.12486	-0.11543						
128	-0.21606	-0.21453	-0.21960	-0.20867	-0.21163	-0.21732							
64	-0.34639	-0.34401	-0.34773	-0.33121	-0.33067								
32	-0.52224	-0.52104	-0.52292	-0.52155									
16	-0.71724	-0.74758	-0.74298										
8	-0.98830	-0.99575											
4	-1.24745												

(f) $\mathcal{P}^{\mathcal{C}}$ for Scene: **[Cornell Box]** Algorithm: **[ERPT]** Metric: **[UQI]** True GT: **[PT @ 32768 spp]**.

M-ERPT	2	4	8	16	32	64	128	256	512	1024	2048	4096	8192
GT (PT)													
16384	-0.00049	-0.00048	-0.00177	-0.00125	-0.00164	-0.00361	-0.00137	-0.00180	-0.00099	0.00023	-0.00076	-0.00214	-0.00130
8192	-0.00460	-0.00335	-0.00241	-0.00562	-0.00398	-0.00409	-0.00234	-0.00443	-0.00308	-0.00325	-0.00360	-0.00388	
4096	-0.00685	-0.00638	-0.00719	-0.00895	-0.00731	-0.01002	-0.00682	-0.00659	-0.00815	-0.00838	-0.00872		
2048	-0.01540	-0.01592	-0.01602	-0.01684	-0.01966	-0.01918	-0.01383	-0.01388	-0.01518	-0.01488			
1024	-0.03122	-0.02915	-0.03404	-0.03334	-0.03139	-0.03475	-0.02787	-0.03735	-0.03177				
512	-0.05994	-0.06507	-0.06367	-0.06595	-0.06313	-0.06117	-0.06375	-0.05730					
256	-0.11510	-0.11338	-0.12011	-0.11187	-0.11385	-0.11710	-0.12002						
128	-0.20142	-0.20343	-0.21289	-0.20437	-0.19891	-0.20852							
64	-0.35184	-0.34083	-0.33073	-0.32758	-0.33791								
32	-0.50252	-0.50703	-0.50218	-0.50386									
16	-0.71709	-0.71761	-0.70885										
8	-0.96195	-0.97264											
4	-1.22621												

(g) $\mathcal{P}^{\mathcal{C}}$ for Scene: **[Cornell Box]** Algorithm: **[M-ERPT]** Metric: **[UQI]** True GT: **[PT @ 32768 spp]**.

Table 91

20.2 Torus

PT	2	4	8	16	32	64	128	256	512	1024	2048	4096	8192	16384	32768	65536	131072
GT (BDPT)																	
262144	0.00130	0.00534	0.00600	-0.00680	-0.00565	-0.00315	-0.01569	-0.00538	0.00287	-0.00580	-0.01519	-0.01566	-0.01263	-0.00615	0.00076	-0.00846	-0.03966
131072	-0.00372	-0.00721	-0.00502	-0.01417	-0.01462	-0.01560	-0.02419	-0.01752	-0.01735	-0.02906	-0.01601	-0.02828	-0.02025	-0.02775	-0.04113	-0.05700	
65536	-0.00015	-0.01620	-0.01842	-0.02341	-0.02496	-0.03589	-0.03043	-0.02288	-0.04166	-0.04511	-0.03722	-0.03977	-0.03359	-0.05911	-0.05911		
32768	-0.00225	-0.02377	-0.02594	-0.05170	-0.05860	-0.06221	-0.06116	-0.05913	-0.06755	-0.05364	-0.05851	-0.06282	-0.07329	-0.09195			
16384	-0.00412	-0.03852	-0.04614	-0.07445	-0.04300	-0.09722	-0.09282	-0.12138	-0.07381	-0.10982	-0.09082	-0.11642	-0.10985				
8192	-0.04630	-0.06206	-0.05018	-0.10955	-0.11834	-0.14667	-0.13005	-0.15904	-0.11063	-0.13196	-0.12889	-0.16123					
4096	-0.07246	-0.10001	-0.08809	-0.14740	-0.15544	-0.16233	-0.16520	-0.17404	-0.15318	-0.16975	-0.17932						
2048	-0.08110	-0.13019	-0.15333	-0.19554	-0.19165	-0.20152	-0.19819	-0.17388	-0.16760	-0.20685							
1024	-0.03879	-0.11650	-0.13878	-0.22346	-0.18950	-0.20462	-0.18648	-0.17789	-0.21822								
512	-0.03021	-0.09634	-0.11317	-0.18567	-0.17349	-0.16575	-0.18074	-0.19237									
256	-0.03305	-0.08103	-0.07961	-0.10796	-0.09801	-0.14203	-0.15899										
128	0.01755	-0.02037	-0.01138	-0.06381	-0.06768	-0.09620											
64	0.02535	-0.01796	-0.01911	-0.03108	-0.04144												
32	0.04949	0.01443	0.02470	-0.08102													
16	0.09591	0.03524	-0.01493														
8	0.02923	-0.02456															
4	-0.05778																

(a) $\mathcal{P}^{\mathcal{C}}$ for Scene: [**Torus**] Algorithm: [**PT**] Metric: [**UQI**] True GT: [**BDPT @ 524288 spp**].

BDPT	2	4	8	16	32	64	128	256	512	1024	2048	4096	8192	16384	32768	65536	131072
GT (BDPT)																	
262144	0.00422	0.00538	-0.00273	-0.00317	-0.01010	0.00186	-0.01473	-0.00394	-0.01859	-0.00369	-0.00816	0.00117	-0.00922	-0.01253	-0.01573	-0.01679	-0.04575
131072	-0.00108	0.00664	-0.00583	-0.00039	0.00136	0.00040	-0.03059	-0.01667	-0.02392	-0.01130	-0.03006	-0.01309	-0.01626	-0.02657	-0.03488	-0.04251	
65536	-0.00073	-0.00297	0.00560	-0.01632	-0.03397	-0.01404	-0.04390	-0.03483	-0.04862	-0.03288	-0.04476	-0.02460	-0.02536	-0.04622	-0.06802		
32768	-0.00777	-0.02153	-0.02847	-0.03921	-0.03431	-0.04945	-0.05014	-0.07134	-0.03702	-0.06595	-0.08103	-0.06716	-0.08091	-0.09545			
16384	0.00854	-0.02815	-0.03429	-0.06100	-0.07875	-0.08188	-0.08764	-0.07764	-0.10836	-0.10463	-0.10005	-0.09741	-0.09257				
8192	-0.01881	-0.03781	-0.08366	-0.09116	-0.09672	-0.10881	-0.13486	-0.09318	-0.15151	-0.11769	-0.12134	-0.10870					
4096	-0.03581	-0.09199	-0.09364	-0.13364	-0.14408	-0.16233	-0.14810	-0.16834	-0.16310	-0.15438	-0.15769						
2048	-0.06364	-0.10146	-0.10596	-0.14338	-0.19226	-0.18090	-0.19550	-0.17274	-0.17286	-0.16828							
1024	-0.06854	-0.09471	-0.16356	-0.17217	-0.18948	-0.18606	-0.18804	-0.16878	-0.22537								
512	-0.03426	-0.08621	-0.11168	-0.14960	-0.15103	-0.17290	-0.17976	-0.15463									
256	0.01051	-0.07446	-0.08212	-0.10015	-0.10606	-0.12561	-0.11755										
128	0.02254	-0.00408	-0.00441	-0.03019	-0.03567	-0.07030											
64	0.03958	0.00366	0.00773	-0.03201	-0.04535												
32	0.08308	0.07015	0.00707	-0.00046													
16	0.09023	0.02233	0.03638														
8	0.07557	0.01418															
4	-0.03320																

(b) $\mathcal{P}^{\mathcal{C}}$ for Scene: [**Torus**] Algorithm: [**BDPT**] Metric: [**UQI**] True GT: [**BDPT @ 524288 spp**].

PSSMLT	2	4	8	16	32	64	128	256	512	1024	2048	4096	8192	16384
GT (BDPT)														
262144	-0.00475	0.00441	-0.00863	-0.01054	-0.01058	-0.00901	-0.00627	-0.00291	-0.00742	-0.01725	-0.01071	-0.01215	-0.01533	-0.02078
131072	-0.00990	0.00931	-0.00864	-0.01118	-0.01428	-0.01867	-0.01579	-0.01442	-0.01912	-0.02609	-0.02940	-0.03249	-0.04659	-0.04977
65536	-0.00890	0.00003	-0.02718	-0.01694	-0.02660	-0.02982	-0.02314	-0.03133	-0.04126	-0.05180	-0.05605	-0.06670	-0.08342	-0.10704
32768	-0.01140	-0.00205	-0.02171	-0.02310	-0.04500	-0.03661	-0.03854	-0.04101	-0.07233	-0.09388	-0.10027	-0.10937	-0.15094	-0.18467
16384	-0.04972	-0.02386	-0.05585	-0.05915	-0.08742	-0.08618	-0.09319	-0.10785	-0.12737	-0.15063	-0.15871	-0.18909	-0.23715	
8192	-0.05039	-0.04925	-0.07348	-0.09091	-0.08235	-0.12276	-0.14080	-0.15366	-0.16923	-0.20460	-0.25192	-0.28268		
4096	-0.02707	-0.07221	-0.08829	-0.09330	-0.13765	-0.14932	-0.18378	-0.18897	-0.23730	-0.28430	-0.33398			
2048	-0.11963	-0.13446	-0.17474	-0.20248	-0.23022	-0.23829	-0.22836	-0.27653	-0.30881	-0.35567				
1024	-0.10553	-0.11683	-0.17596	-0.17677	-0.20915	-0.23949	-0.28081	-0.32908	-0.37608					
512	-0.14084	-0.14782	-0.17452	-0.20089	-0.22237	-0.27980	-0.28611	-0.32569						
256	-0.09591	-0.11511	-0.15751	-0.20308	-0.21333	-0.27759	-0.32118							
128	-0.03349	-0.06702	-0.11148	-0.14195	-0.15830	-0.21106								
64	-0.08321	-0.08213	-0.11412	-0.17156	-0.22046									
32	-0.10652	-0.09907	-0.13627	-0.19805										
16	-0.14064	-0.08468	-0.13863											
8	-0.15168	-0.14206												
4	-0.18236													

(c) $\mathcal{P}^{\mathcal{C}}$ for Scene: [**Torus**] Algorithm: [**PSSMLT**] Metric: [**UQI**] True GT: [**BDPT @ 524288 spp**].

MLT	2	4	8	16	32	64	128	256	512	1024	2048	4096	8192	16384
GT (BDPT)														
262144	-0.01071	-0.01507	0.00583	-0.01183	-0.00489	-0.00826	-0.00785	-0.00498	-0.00941	-0.01708	-0.02224	-0.01935	-0.02057	-0.02657
131072	-0.02572	0.00743	-0.01231	-0.01310	-0.02341	-0.02932	-0.02024	-0.02967	-0.03657	-0.04098	-0.04742	-0.05488	-0.06254	-0.07399
65536	-0.03457	-0.02508	-0.01344	-0.02761	-0.03079	-0.04814	-0.05726	-0.06055	-0.07585	-0.08975	-0.08536	-0.10326	-0.11101	-0.12386
32768	-0.04226	-0.00788	-0.00826	-0.03454	-0.06989	-0.06082	-0.09030	-0.11535	-0.12069	-0.13890	-0.15177	-0.16044	-0.18369	-0.20203
16384	-0.03640	-0.02402	-0.01647	-0.04638	-0.09152	-0.10838	-0.15235	-0.17320	-0.19360	-0.23207	-0.26012	-0.26875	-0.28557	
8192	-0.02538	-0.03647	-0.06251	-0.08940	-0.14963	-0.18589	-0.23392	-0.25035	-0.28447	-0.30683	-0.33021	-0.33742		
4096	-0.03291	-0.04954	-0.08466	-0.15554	-0.18806	-0.25565	-0.31522	-0.35156	-0.37606	-0.39787	-0.43206			
2048	-0.02851	-0.08130	-0.11122	-0.18588	-0.26384	-0.33942	-0.40106	-0.43334	-0.48297	-0.50328				
1024	-0.05965	-0.09759	-0.13368	-0.21825	-0.33209	-0.40098	-0.47661	-0.49626	-0.54615					
512	-0.03857	-0.18779	-0.14666	-0.31933	-0.35356	-0.42930	-0.50827	-0.54387						
256	-0.04388	-0.13409	-0.17486	-0.28017	-0.39626	-0.43371	-0.50502							
128	-0.01843	-0.12104	-0.12011	-0.32502	-0.34332	-0.41245								
64	-0.00011	-0.16022	-0.14862	-0.35754	-0.37618									
32	0.00857	-0.24320	-0.17446	-0.32598										
16	-0.02163	-0.18512	-0.20884											
8	-0.12174	-0.28599												
4	-0.19841													

(d) $\mathcal{P}^{\mathcal{C}}$ for Scene: [**Torus**] Algorithm: [**MLT**] Metric: [**UQI**] True GT: [**BDPT @ 524288 spp**].

M-MLT	2	4	8	16	32	64	128	256	512	1024	2048	4096	8192	16384
GT (BDPT)														
262144	0.01314	-0.01547	-0.00809	0.00377	-0.00357	-0.01061	-0.01091	-0.01774	-0.02423	-0.02245	-0.01966	-0.03278	-0.03089	-0.03570
131072	0.00622	0.00543	-0.00127	0.00979	0.00535	-0.00330	-0.01407	-0.02766	-0.05824	-0.05576	-0.05600	-0.07880	-0.07963	-0.08336
65536	-0.00541	-0.00057	-0.01100	-0.03006	-0.01877	-0.04527	-0.05757	-0.08028	-0.10227	-0.11291	-0.11521	-0.13131	-0.13662	-0.15584
32768	0.00234	-0.02171	-0.01695	-0.02553	-0.03786	-0.09029	-0.08675	-0.12428	-0.16878	-0.17701	-0.19431	-0.20251	-0.22917	-0.25184
16384	-0.00737	-0.03664	-0.05469	-0.05773	-0.06735	-0.13083	-0.15260	-0.18939	-0.26339	-0.28764	-0.28812	-0.30812	-0.32197	
8192	-0.03072	-0.04950	-0.05857	-0.12270	-0.13461	-0.19133	-0.23540	-0.27165	-0.35311	-0.35965	-0.39327	-0.41938		
4096	0.01648	-0.05672	-0.12434	-0.12671	-0.16025	-0.29232	-0.31482	-0.37686	-0.44920	-0.46825	-0.48955			
2048	-0.02732	-0.04235	-0.11648	-0.21404	-0.27201	-0.37518	-0.39173	-0.47112	-0.56774	-0.57785				
1024	-0.04605	-0.13156	-0.21471	-0.25060	-0.32130	-0.44633	-0.47435	-0.53797	-0.61364					
512	-0.03579	-0.09185	-0.27812	-0.33791	-0.31085	-0.47934	-0.52543	-0.55433						
256	-0.01788	-0.15380	-0.28382	-0.35852	-0.34183	-0.52064	-0.58095							
128	-0.01681	-0.06821	-0.30283	-0.28258	-0.34674	-0.48997								
64	-0.10273	-0.15462	-0.35607	-0.36753	-0.34426									
32	-0.03312	-0.09613	-0.41475	-0.32909										
16	-0.11718	-0.23497	-0.42729											
8	-0.12958	-0.21604												
4	-0.23268													

(e) $\mathcal{P}^{\mathcal{C}}$ for Scene: [**Torus**] Algorithm: [**M-MLT**] Metric: [**UQI**] True GT: [**BDPT @ 524288 spp**].

ERPT	2	4	8	16	32	64	128	256	512	1024	2048	4096	8192	16384	32768	65536	131072
GT (BDPT)																	
262144	-0.00387	-0.00818	-0.01448	-0.01187	-0.01725	-0.01091	-0.00875	-0.00754	-0.00746	-0.00766	-0.01056	-0.01138	-0.00960	-0.01581	-0.00471	-0.00722	-0.00933
131072	-0.02775	-0.03076	-0.03492	-0.03688	-0.02788	-0.02445	-0.02636	-0.01614	-0.02023	-0.02613	-0.02626	-0.03246	-0.03057	-0.02785	-0.02909	-0.03791	
65536	-0.03401	-0.06012	-0.06289	-0.04330	-0.05117	-0.05600	-0.05702	-0.05013	-0.05102	-0.05930	-0.05716	-0.06582	-0.05629	-0.06529	-0.05713		
32768	-0.11017	-0.09115	-0.09736	-0.10096	-0.09871	-0.10190	-0.10148	-0.10168	-0.08715	-0.09867	-0.09964	-0.12235	-0.11637	-0.09573			
16384	-0.15794	-0.15058	-0.18455	-0.17235	-0.16921	-0.16487	-0.16341	-0.16918	-0.16926	-0.16093	-0.16880	-0.18673	-0.19233				
8192	-0.23221	-0.22964	-0.25875	-0.24168	-0.24986	-0.23128	-0.25937	-0.26011	-0.25862	-0.25322	-0.28296	-0.28140					
4096	-0.31811	-0.29271	-0.34339	-0.34745	-0.34808	-0.35557	-0.33516	-0.34792	-0.34334	-0.36088	-0.36530						
2048	-0.34829	-0.35493	-0.42972	-0.41129	-0.43819	-0.41410	-0.44039	-0.44915	-0.42991	-0.44911							
1024	-0.40894	-0.44498	-0.48609	-0.47355	-0.52849	-0.50685	-0.51658	-0.55552	-0.52996								
512	-0.43035	-0.46717	-0.48890	-0.50668	-0.55447	-0.52469	-0.57069	-0.57927									
256	-0.43385	-0.43316	-0.48989	-0.48543	-0.57539	-0.54214	-0.58380										
128	-0.36865	-0.39222	-0.45118	-0.48643	-0.55623	-0.50688											
64	-0.35713	-0.41730	-0.50990	-0.48835	-0.54084												
32	-0.35568	-0.43722	-0.49845	-0.50356													
16	-0.41226	-0.49097	-0.56641														
8	-0.49988	-0.55342															
4	-0.64850																

(f) $\mathcal{P}^{\mathcal{C}}$ for Scene: [**Torus**] Algorithm: [**ERPT**] Metric: [**UQI**] True GT: [**BDPT @ 524288 spp**].

M-ERPT	2	4	8	16	32	64	128	256	512	1024	2048	4096	8192	16384	32768	65536	131072
GT (BDPT)																	
262144	-0.00054	-0.01707	-0.00791	-0.00188	-0.01121	-0.01078	-0.01139	-0.01271	-0.00956	-0.01133	-0.02227	-0.01343	-0.01438	-0.01459	-0.00959	-0.00389	-0.01491
131072	-0.02009	-0.03049	-0.02023	-0.02140	-0.02856	-0.02435	-0.01980	-0.03498	-0.03602	-0.03407	-0.03110	-0.03556	-0.03668	-0.04166	-0.02952	-0.02947	
65536	-0.03130	-0.05504	-0.03395	-0.04810	-0.05748	-0.04695	-0.05302	-0.05540	-0.06384	-0.05237	-0.06273	-0.06275	-0.06536	-0.06853	-0.06860		
32768	-0.05828	-0.08185	-0.07064	-0.08181	-0.08674	-0.10040	-0.10537	-0.11817	-0.10181	-0.12140	-0.12673	-0.12201	-0.11642	-0.10380			
16384	-0.10135	-0.14905	-0.13349	-0.14736	-0.14742	-0.17787	-0.18040	-0.18739	-0.16265	-0.19250	-0.19645	-0.18101	-0.19132				
8192	-0.11645	-0.18714	-0.20195	-0.20172	-0.20433	-0.23998	-0.26917	-0.26828	-0.28484	-0.28138	-0.29310	-0.26775					
4096	-0.21807	-0.26402	-0.27934	-0.31146	-0.31756	-0.32603	-0.34754	-0.36076	-0.36596	-0.38073	-0.37371						
2048	-0.26044	-0.33470	-0.35900	-0.38988	-0.39029	-0.40369	-0.44479	-0.44201	-0.44203	-0.47052							
1024	-0.30719	-0.37171	-0.40376	-0.45437	-0.44717	-0.48996	-0.53977	-0.52978	-0.55030								
512	-0.32575	-0.39615	-0.42985	-0.49099	-0.45641	-0.52800	-0.58135	-0.58177									
256	-0.28923	-0.39944	-0.39264	-0.47134	-0.47153	-0.55552	-0.59625										
128	-0.23641	-0.32757	-0.39228	-0.47272	-0.45942	-0.52103											
64	-0.25179	-0.29694	-0.37717	-0.48928	-0.45070												
32	-0.25393	-0.37080	-0.41923	-0.47845													
16	-0.28777	-0.40882	-0.44175														
8	-0.35495	-0.48888															
4	-0.48457																

(g) $\mathcal{P}^{\mathcal{C}}$ for Scene: [**Torus**] Algorithm: [**M-ERPT**] Metric: [**UQI**] True GT: [**BDPT @ 524288 spp**].

Table 92

20.3 Veach Bidir

PT	2	4	8	16	32	64	128	256	512	1024	2048	4096	8192	16384	32768	65536	131072
GT (BDPT)																	
262144	-0.00010	-0.00009	-0.00004	0.00017	-0.00001	-0.00023	0.00015	-0.00008	0.00021	-0.00040	0.00007	0.00093	-0.00027	0.00060	-0.00001	-0.00065	-0.00034
131072	0.00013	0.00002	0.00097	0.00031	-0.00016	0.00026	0.00080	0.00067	0.00022	-0.00083	-0.00005	0.00050	0.00001	0.00087	-0.00021	-0.00181	
65536	-0.00081	-0.00095	0.00018	0.00028	0.00034	0.00042	0.00072	-0.00020	-0.00050	-0.00067	0.00049	0.00143	-0.00004	-0.00019	-0.00035		
32768	0.00123	-0.00146	0.00026	0.00117	-0.00091	-0.00004	0.00126	0.00027	-0.00022	-0.00041	-0.00004	0.00080	-0.00110	-0.00108			
16384	0.00046	-0.00013	0.00206	0.00173	0.00049	-0.00032	0.00072	-0.00035	-0.00037	-0.00041	-0.00050	0.00078	-0.00077				
8192	0.00067	-0.00094	0.00134	0.00090	-0.00116	0.00127	0.00063	-0.00087	-0.00028	-0.00100	-0.00333	0.00021					
4096	-0.00128	0.00186	0.00186	0.00272	0.00041	-0.00037	-0.00021	-0.00052	0.00029	-0.00099	-0.00273						
2048	0.00284	-0.00091	0.00002	0.00192	-0.00077	0.00135	-0.00160	-0.00437	-0.00289	-0.00362							
1024	-0.00146	-0.00485	-0.00051	0.00218	-0.00202	-0.00064	-0.00161	-0.00490	-0.00413								
512	-0.00162	-0.00315	0.00058	-0.00635	-0.00336	-0.00001	-0.00483	-0.00977									
256	0.00254	-0.00075	0.00190	0.00112	-0.00178	-0.00436	-0.01467										
128	0.00337	-0.00853	0.00683	-0.00202	-0.01004	-0.00973											
64	-0.00137	0.00643	0.00690	-0.00204	-0.00762												
32	0.01174	-0.00474	-0.00395	-0.02019													
16	0.00742	-0.01834	-0.03388														
8	-0.02541	-0.08296															
4	-0.07367																

(a) $\mathcal{P}^{\mathcal{C}}$ for Scene: [**Veach Bidir**] Algorithm: [**PT**] Metric: [**UQI**] True GT: [**BDPT @ 524288 spp**].

BDPT	2	4	8	16	32	64	128	256	512	1024	2048	4096	8192	16384	32768	65536	131072
GT (BDPT)																	
262144	0.00023	0.00019	0.00017	-0.00008	-0.00000	-0.00018	-0.00060	-0.00045	-0.00030	-0.00014	-0.00043	-0.00075	-0.00035	0.00004	0.00002	0.00081	0.00016
131072	0.00046	0.00089	0.00065	-0.00040	0.00032	0.00038	-0.00053	-0.00086	-0.00053	-0.00078	-0.00109	-0.00152	-0.00099	-0.00153	-0.00170	-0.00200	
65536	0.00113	0.00040	0.00051	-0.00027	0.00031	0.00053	-0.00097	-0.00053	-0.00128	-0.00249	-0.00304	-0.00366	-0.00368	-0.00546	-0.00721		
32768	0.00045	0.00013	0.00007	-0.00063	-0.00008	-0.00110	-0.00244	-0.00220	-0.00272	-0.00434	-0.00668	-0.00783	-0.00996	-0.01272			
16384	0.00125	0.00088	0.00197	-0.00049	-0.00060	-0.00010	-0.00213	-0.00284	-0.00673	-0.00716	-0.01231	-0.01594	-0.01871				
8192	0.00068	0.00142	0.00268	0.00029	-0.00062	-0.00017	-0.00345	-0.00656	-0.01083	-0.01631	-0.02189	-0.02866					
4096	0.00072	-0.00015	0.00024	0.00011	-0.00174	-0.00512	-0.00810	-0.01332	-0.02022	-0.02925	-0.04388						
2048	0.00201	0.00168	-0.00154	-0.00202	-0.00155	-0.00708	-0.01242	-0.02203	-0.03853	-0.05803							
1024	-0.00050	-0.00132	-0.00167	-0.00214	-0.00597	-0.01480	-0.02471	-0.04590	-0.07074								
512	-0.00026	-0.00015	-0.00119	-0.00749	-0.01176	-0.03052	-0.05367	-0.08285									
256	-0.00123	0.00411	-0.00681	-0.01629	-0.02729	-0.05052	-0.09025										
128	-0.00055	-0.00547	-0.02012	-0.03289	-0.05316	-0.10220											
64	-0.00598	-0.01914	-0.03493	-0.07011	-0.10543												
32	-0.01115	-0.04864	-0.06178	-0.10418													
16	-0.03311	-0.04870	-0.10415														
8	-0.05480	-0.12178															
4	-0.12665																

(b) $\mathcal{P}^{\mathcal{C}}$ for Scene: [**Veach Bidir**] Algorithm: [**BDPT**] Metric: [**UQI**] True GT: [**BDPT @ 524288 spp**].

PSSMLT	2	4	8	16	32	64	128	256	512	1024	2048	4096	8192	16384
GT (BDPT)														
262144	0.00041	0.00023	0.00034	0.00021	0.00015	-0.00028	-0.00028	-0.00048	-0.00055	-0.00068	-0.00114	-0.00098	-0.00085	-0.00068
131072	0.00088	0.00018	0.00059	0.00023	0.00040	-0.00006	-0.00007	-0.00059	-0.00093	-0.00081	-0.00080	-0.00068	-0.00144	-0.00175
65536	0.00044	0.00026	-0.00030	0.00019	-0.00003	-0.00095	-0.00154	-0.00257	-0.00252	-0.00231	-0.00267	-0.00352	-0.00414	-0.00496
32768	0.00067	-0.00031	-0.00039	-0.00034	-0.00150	-0.00235	-0.00328	-0.00380	-0.00445	-0.00482	-0.00635	-0.00785	-0.01041	-0.01234
16384	0.00033	-0.00004	0.00005	0.00083	0.00012	-0.00104	-0.00277	-0.00450	-0.00642	-0.00835	-0.01219	-0.01469	-0.01875	
8192	0.00092	-0.00072	0.00094	0.00074	-0.00083	-0.00377	-0.00509	-0.00696	-0.01136	-0.01583	-0.02188	-0.02859		
4096	0.00229	0.00105	0.00117	0.00069	-0.00163	-0.00391	-0.00739	-0.01180	-0.01898	-0.03042	-0.04196			
2048	0.00051	0.00004	0.00007	-0.00156	-0.00250	-0.00963	-0.01494	-0.02649	-0.03908	-0.05730				
1024	0.00084	-0.00137	0.00051	-0.00322	-0.00945	-0.01406	-0.02730	-0.04243	-0.06923					
512	0.00056	-0.00101	-0.00091	-0.00367	-0.01400	-0.02839	-0.04854	-0.07852						
256	-0.00362	-0.00520	-0.01252	-0.02265	-0.03709	-0.06068	-0.09304							
128	-0.01304	-0.01466	-0.02155	-0.03935	-0.06815	-0.10348								
64	-0.01318	-0.02301	-0.03982	-0.06594	-0.11131									
32	-0.04800	-0.07385	-0.09764	-0.13730										
16	-0.08150	-0.11899	-0.16027											
8	-0.14356	-0.19412												
4	-0.28564													

(c) $\mathcal{P}^{\mathcal{C}}$ for Scene: [**Veach Bidir**] Algorithm: [**PSSMLT**] Metric: [**UQI**] True GT: [**BDPT @ 524288 spp**].

MLT	2	4	8	16	32	64	128	256	512	1024	2048	4096	8192	16384
GT (BDPT)														
262144	0.00005	0.00026	0.00004	-0.00005	0.00040	0.00059	0.00073	0.00061	0.00005	0.00004	-0.00000	-0.00012	-0.00023	-0.00049
131072	0.00037	0.00054	0.00059	0.00069	0.00080	0.00071	0.00053	0.00036	0.00015	-0.00033	0.00040	-0.00001	-0.00041	-0.00099
65536	0.00072	0.00120	0.00120	0.00137	0.00141	0.00064	-0.00007	-0.00032	-0.00145	-0.00132	-0.00131	-0.00188	-0.00258	-0.00321
32768	-0.00076	-0.00012	-0.00003	0.00042	0.00041	0.00029	-0.00022	-0.00029	-0.00102	-0.00210	-0.00224	-0.00336	-0.00441	-0.00702
16384	0.00063	0.00030	0.00074	0.00099	0.00107	0.00022	0.00060	-0.00098	-0.00196	-0.00344	-0.00495	-0.00851	-0.01140	
8192	0.00025	-0.00057	0.00086	0.00055	-0.00020	-0.00172	-0.00106	-0.00226	-0.00317	-0.00626	-0.00981	-0.01508		
4096	0.00181	0.00054	0.00163	0.00182	0.00232	-0.00005	-0.00204	-0.00284	-0.00651	-0.01142	-0.01720			
2048	0.00164	0.00067	0.00187	0.00013	-0.00135	-0.00219	-0.00384	-0.00737	-0.01274	-0.02183				
1024	-0.00140	0.00129	0.00227	0.00218	-0.00102	-0.00246	-0.00434	-0.00800	-0.01972					
512	0.00414	0.00207	0.00382	0.00058	-0.00323	-0.00916	-0.01855	-0.03218						
256	-0.00507	-0.00690	0.00023	0.00035	-0.00585	-0.01585	-0.02523							
128	-0.00344	-0.00936	-0.01077	-0.01728	-0.02692	-0.03572								
64	-0.00939	-0.01898	-0.02378	-0.04228	-0.04895									
32	-0.02783	-0.03264	-0.03723	-0.05426										
16	-0.05479	-0.06492	-0.08725											
8	-0.10338	-0.10703												
4	-0.15123													

(d) $\mathcal{P}^{\mathcal{C}}$ for Scene: [**Veach Bidir**] Algorithm: [**MLT**] Metric: [**UQI**] True GT: [**BDPT @ 524288 spp**].

M-MLT	2	4	8	16	32	64	128	256	512	1024	2048	4096	8192	16384
GT (BDPT)														
262144	0.00004	0.00003	0.00021	0.00002	-0.00006	-0.00024	0.00065	0.00052	0.00043	-0.00007	-0.00049	-0.00110	-0.00046	-0.00050
131072	0.00044	0.00018	0.00046	0.00079	0.00018	0.00040	0.00067	0.00002	-0.00026	-0.00003	-0.00024	-0.00055	-0.00101	-0.00141
65536	-0.00033	-0.00066	0.00079	0.00017	0.00028	0.00053	0.00077	0.00050	0.00009	0.00066	-0.00137	-0.00197	-0.00262	-0.00289
32768	0.00040	-0.00045	0.00002	-0.00027	-0.00041	0.00070	0.00028	-0.00061	-0.00150	-0.00264	-0.00297	-0.00521	-0.00629	-0.00794
16384	0.00076	-0.00054	-0.00026	0.00132	0.00059	0.00025	0.00087	-0.00017	-0.00097	-0.00120	-0.00406	-0.00820	-0.01138	
8192	0.00079	0.00120	0.00097	0.00132	0.00018	-0.00022	0.00174	-0.00024	-0.00228	-0.00552	-0.00944	-0.01609		
4096	0.00229	-0.00105	0.00023	0.00251	-0.00122	-0.00076	-0.00167	-0.00358	-0.00595	-0.00946	-0.02065			
2048	0.00050	-0.00381	-0.00013	0.00253	0.00096	0.00001	-0.00343	-0.00716	-0.01189	-0.02163				
1024	-0.00300	-0.00033	-0.00376	-0.00119	-0.00516	-0.00372	-0.00421	-0.01130	-0.02375					
512	0.00086	-0.00426	-0.00493	-0.00787	-0.00915	-0.01059	-0.01834	-0.03142						
256	-0.00013	0.00197	-0.00513	-0.00673	-0.01476	-0.02123	-0.03348							
128	-0.00992	-0.00766	-0.01304	-0.00870	-0.02124	-0.03714								
64	-0.00729	-0.00614	-0.01862	-0.02250	-0.04347									
32	-0.02448	-0.03285	-0.04416	-0.04328										
16	-0.03359	-0.04418	-0.07825											
8	-0.07873	-0.10612												
4	-0.15337													

(e) $\mathcal{P}^{\mathcal{C}}$ for Scene: [**Veach Bidir**] Algorithm: [**M-MLT**] Metric: [**UQI**] True GT: [**BDPT @ 524288 spp**].

ERPT	2	4	8	16	32	64	128	256	512	1024	2048	4096	8192	16384	32768	65536	131072
GT (BDPT)																	
262144	0.00038	0.00001	0.00030	-0.00034	0.00011	0.00016	0.00030	0.00027	-0.00024	-0.00020	-0.00006	-0.00030	0.00000	-0.00033	-0.00010	-0.00003	-0.00017
131072	0.00078	0.00051	0.00002	-0.00006	0.00006	0.00048	0.00037	0.00082	0.00004	-0.00013	0.00071	0.00004	0.00050	-0.00050	-0.00001	-0.00011	
65536	-0.00027	0.00033	-0.00067	-0.00054	-0.00026	-0.00045	-0.00024	0.00005	-0.00056	-0.00119	-0.00098	-0.00059	0.00011	-0.00029	-0.00012		
32768	-0.00141	-0.00026	-0.00160	-0.00100	-0.00075	-0.00039	-0.00048	0.00049	0.00023	-0.00094	-0.00068	-0.00058	-0.00128	-0.00123			
16384	-0.00078	-0.00072	-0.00019	-0.00104	-0.00080	-0.00176	-0.00125	-0.00132	-0.00301	-0.00137	-0.00031	-0.00182	-0.00141				
8192	-0.00042	-0.00095	-0.00191	-0.00206	-0.00184	-0.00202	-0.00247	0.00053	-0.00187	-0.00279	-0.00368	-0.00315					
4096	-0.00360	-0.00049	-0.00441	-0.00301	-0.00327	-0.00393	-0.00224	-0.00191	-0.00509	-0.00224	-0.00195						
2048	-0.00651	-0.00771	-0.00325	-0.00740	-0.00465	-0.00532	-0.00589	-0.00525	-0.00553	-0.00455							
1024	-0.00759	-0.00929	-0.00658	-0.00883	-0.00867	-0.01329	-0.00931	-0.01172	-0.01026								
512	-0.01095	-0.01578	-0.01355	-0.01889	-0.02004	-0.02280	-0.02098	-0.01573									
256	-0.03330	-0.03607	-0.03776	-0.03724	-0.03903	-0.02913	-0.03884										
128	-0.07280	-0.06756	-0.07546	-0.06253	-0.07096	-0.06789											
64	-0.12184	-0.12006	-0.11201	-0.11391	-0.12850												
32	-0.21031	-0.19557	-0.20834	-0.20625													
16	-0.32059	-0.32216	-0.32477														
8	-0.47812	-0.48700															
4	-0.69804																

(f) $\mathcal{P}^{\mathcal{C}}$ for Scene: [**Veach Bidir**] Algorithm: [**ERPT**] Metric: [**UQI**] True GT: [**BDPT @ 524288 spp**].

M-ERPT	2	4	8	16	32	64	128	256	512	1024	2048	4096	8192	16384	32768	65536	131072
GT (BDPT)																	
262144	-0.00028	0.00061	0.00028	0.00011	0.00013	-0.00033	0.00014	-0.00036	-0.00085	-0.00039	-0.00059	0.00041	-0.00001	0.00009	-0.00046	-0.00062	0.00061
131072	-0.00024	-0.00027	0.00010	0.00045	0.00053	0.00012	0.00060	-0.00005	-0.00014	-0.00007	-0.00011	0.00019	0.00038	0.00034	-0.00005	-0.00031	
65536	-0.00027	0.00053	0.00061	-0.00051	-0.00003	0.00003	-0.00013	-0.00020	-0.00128	-0.00062	-0.00077	-0.00021	-0.00025	-0.00081	0.00020		
32768	0.00004	-0.00090	-0.00090	-0.00043	-0.00030	-0.00065	-0.00200	-0.00107	-0.00200	-0.00118	-0.00096	-0.00113	-0.00045	-0.00147			
16384	-0.00082	0.00017	-0.00149	-0.00129	0.00008	-0.00065	-0.00114	-0.00205	-0.00156	-0.00187	-0.00174	-0.00206	-0.00214				
8192	-0.00258	-0.00048	-0.00192	-0.00230	-0.00207	-0.00105	-0.00237	-0.00282	-0.00297	-0.00210	-0.00228	-0.00177					
4096	-0.00311	-0.00287	-0.00441	-0.00206	-0.00121	-0.00230	-0.00230	-0.00419	-0.00466	-0.00362	-0.00303						
2048	-0.00306	-0.00388	-0.00608	-0.00614	-0.00879	-0.00624	-0.00355	-0.00561	-0.00499	-0.00354							
1024	-0.01021	-0.00802	-0.01118	-0.00654	-0.00772	-0.01004	-0.01039	-0.00870	-0.01000								
512	-0.01954	-0.01233	-0.01935	-0.02106	-0.01813	-0.01888	-0.02309	-0.02374									
256	-0.02724	-0.03141	-0.02904	-0.03844	-0.03991	-0.03598	-0.03411										
128	-0.06991	-0.06192	-0.06920	-0.06860	-0.07574	-0.06533											
64	-0.11055	-0.11904	-0.11764	-0.11769	-0.12381												
32	-0.18746	-0.18434	-0.20052	-0.19790													
16	-0.31563	-0.30809	-0.32002														
8	-0.46144	-0.46041															
4	-0.67006																

(g) $\mathcal{P}^{\mathcal{C}}$ for Scene: [**Veach Bidir**] Algorithm: [**M-ERPT**] Metric: [**UQI**] True GT: [**BDPT @ 524288 spp**].

Table 93

20.4 Veach Door

PT	2	4	8	16	32	64	128	256	512	1024	2048	4096	8192	16384	32768	65536	131072
GT (BDPT)																	
262144	0.00067	-0.00009	-0.00040	-0.00021	-0.00035	0.00003	-0.00037	-0.00006	0.00018	-0.00068	0.00018	0.00005	-0.00013	0.00019	-0.00059	-0.00255	-0.00297
131072	0.00048	-0.00059	-0.00009	-0.00034	-0.00022	0.00052	-0.00056	0.00038	-0.00025	-0.00029	-0.00034	-0.00027	-0.00078	-0.00142	-0.00282	-0.00526	
65536	0.00045	0.00020	0.00015	0.00054	-0.00041	0.00021	0.00020	-0.00017	-0.00010	-0.00035	-0.00135	-0.00154	-0.00223	-0.00530	-0.00593		
32768	-0.00072	-0.00141	0.00005	-0.00079	-0.00087	-0.00007	-0.00049	-0.00145	-0.00068	-0.00085	-0.00121	-0.00343	-0.00457	-0.00803			
16384	0.00060	-0.00051	-0.00068	-0.00063	-0.00037	-0.00037	-0.00025	-0.00126	-0.00346	-0.00353	-0.00250	-0.00682	-0.01241				
8192	-0.00011	0.00075	-0.00006	0.00113	0.00107	-0.00097	-0.00266	-0.00036	-0.00403	-0.00566	-0.00896	-0.01651					
4096	0.00214	-0.00112	-0.00017	-0.00239	-0.00281	-0.00415	0.00090	-0.00284	-0.00518	-0.01100	-0.01944						
2048	0.00510	0.00128	0.00468	-0.00555	-0.00159	-0.00148	-0.00205	-0.00836	-0.01759	-0.01930							
1024	-0.00186	0.00293	0.00011	-0.00450	-0.00355	-0.00581	-0.01153	-0.02389	-0.02390								
512	-0.00168	-0.00211	-0.00517	-0.00574	-0.00847	-0.01013	-0.01934	-0.03200									
256	-0.00708	0.00608	-0.00508	-0.00448	-0.01952	-0.03759	-0.04832										
128	-0.00583	0.00474	-0.01244	-0.02083	-0.03336	-0.04636											
64	0.00831	-0.00090	0.01134	-0.01823	-0.06569												
32	0.06283	0.02812	-0.02453	-0.05446													
16	0.14861	0.08773	-0.02238														
8	0.17838	0.07630															
4	0.12964																

(a) $\mathcal{P}^{\mathcal{C}}$ for Scene: [**Veach Door**] Algorithm: [**PT**] Metric: [**UQI**] True GT: [**BDPT @ 524288 spp**].

BDPT	2	4	8	16	32	64	128	256	512	1024	2048	4096	8192	16384	32768	65536	131072
GT (BDPT)																	
262144	-0.00022	0.00022	-0.00002	0.00002	-0.00024	-0.00022	0.00019	-0.00015	0.00032	0.00016	-0.00140	-0.00210	0.00024	-0.00335	-0.00664	-0.00979	-0.01231
131072	-0.00045	0.00003	0.00037	-0.00000	0.00029	0.00033	-0.00049	-0.00034	-0.00079	-0.00099	-0.00195	-0.00254	-0.00573	-0.00923	-0.01713	-0.02705	
65536	0.00058	0.00082	0.00010	0.00023	-0.00049	-0.00038	0.00039	-0.00083	-0.00136	-0.00187	-0.00250	-0.00668	-0.01047	-0.02257	-0.03910		
32768	0.00012	-0.00005	0.00001	-0.00055	-0.00040	-0.00053	0.00023	-0.00266	-0.00367	-0.00357	-0.00858	-0.01617	-0.02593	-0.04546			
16384	-0.00089	-0.00038	-0.00075	-0.00015	-0.00017	-0.00141	-0.00266	-0.00213	-0.00520	-0.00781	-0.01506	-0.03005	-0.05113				
8192	-0.00069	0.00090	0.00027	-0.00165	-0.00141	-0.00570	-0.00314	-0.00548	-0.01177	-0.01765	-0.03175	-0.05709					
4096	0.00205	-0.00131	-0.00280	-0.00456	-0.00277	-0.00723	-0.00946	-0.01025	-0.02341	-0.03277	-0.05333						
2048	0.00188	-0.00260	-0.00450	-0.00363	-0.00425	-0.01332	-0.01411	-0.02480	-0.03938	-0.06395							
1024	0.00111	-0.00588	-0.00267	-0.00798	-0.01221	-0.01770	-0.02808	-0.04524	-0.07837								
512	-0.00108	-0.00334	-0.01378	-0.01092	-0.01887	-0.03355	-0.06297	-0.08240									
256	-0.01371	-0.00732	-0.01067	-0.01939	-0.03019	-0.06669	-0.09267										
128	-0.01670	-0.00246	-0.02492	-0.04480	-0.06565	-0.10206											
64	0.00579	-0.00231	-0.04461	-0.06447	-0.11304												
32	0.02148	0.01380	-0.04569	-0.12039													
16	0.04192	-0.04837	-0.08052														
8	0.05108	-0.06476															
4	-0.01360																

(b) $\mathcal{P}^{\mathcal{C}}$ for Scene: [**Veach Door**] Algorithm: [**BDPT**] Metric: [**UQI**] True GT: [**BDPT @ 524288 spp**].

PSSMLT	2	4	8	16	32	64	128	256	512	1024	2048	4096	8192	16384
GT (BDPT)														
262144	-0.00010	-0.00008	0.00042	0.00038	0.00014	-0.00003	-0.00048	0.00028	-0.00045	-0.00041	-0.00193	-0.00228	-0.00535	-0.00881
131072	-0.00036	-0.00011	-0.00028	0.00030	-0.00003	-0.00019	-0.00098	0.00023	-0.00009	-0.00118	-0.00422	-0.00746	-0.01260	-0.02236
65536	0.00018	-0.00026	-0.00011	0.00081	-0.00045	-0.00024	-0.00127	-0.00126	-0.00334	-0.00516	-0.01155	-0.01796	-0.02866	-0.04727
32768	-0.00130	-0.00154	-0.00098	-0.00110	-0.00186	-0.00357	-0.00475	-0.00555	-0.00886	-0.01467	-0.02507	-0.03847	-0.06130	-0.09081
16384	0.00046	-0.00030	-0.00074	-0.00134	-0.00241	-0.00131	-0.00363	-0.00529	-0.01456	-0.02361	-0.04022	-0.06613	-0.10216	
8192	-0.00113	-0.00096	-0.00125	-0.00140	-0.00176	-0.00468	-0.01086	-0.01188	-0.02250	-0.03683	-0.07098	-0.10838		
4096	-0.00176	-0.00209	-0.00205	-0.00467	-0.00867	-0.01140	-0.02058	-0.03423	-0.05194	-0.08623	-0.12184			
2048	-0.00039	-0.00103	-0.00599	-0.00994	-0.00626	-0.01700	-0.03290	-0.04787	-0.08165	-0.12211				
1024	-0.00123	-0.00724	-0.00928	-0.01660	-0.02568	-0.04046	-0.06245	-0.09572	-0.14103					
512	-0.01308	-0.01402	-0.02064	-0.03181	-0.04505	-0.07253	-0.11071	-0.16161						
256	-0.01080	-0.02173	-0.03290	-0.05928	-0.08255	-0.12267	-0.18003							
128	-0.02481	-0.04030	-0.04831	-0.07515	-0.11107	-0.18369								
64	-0.05707	-0.06668	-0.09587	-0.14711	-0.21062									
32	-0.04471	-0.11479	-0.17982	-0.23090										
16	-0.06408	-0.13293	-0.23745											
8	-0.09949	-0.21906												
4	-0.24815													

(c) $\mathcal{P}^{\mathcal{C}}$ for Scene: [**Veach Door**] Algorithm: [**PSSMLT**] Metric: [**UQI**] True GT: [**BDPT @ 524288 spp**].

MLT	2	4	8	16	32	64	128	256	512	1024	2048	4096	8192	16384
GT (BDPT)														
262144	0.00044	0.00016	-0.00047	-0.00016	-0.00040	-0.00026	0.00002	-0.00041	0.00089	-0.00194	-0.00202	-0.00388	-0.00706	-0.00826
131072	0.00040	0.00036	-0.00004	0.00048	-0.00037	-0.00028	0.00020	-0.00045	-0.00165	-0.00338	-0.00724	-0.01329	-0.02131	-0.03033
65536	0.00077	-0.00001	0.00011	0.00017	-0.00112	-0.00260	-0.00225	-0.00493	-0.00605	-0.01047	-0.01570	-0.03146	-0.04566	-0.06547
32768	0.00041	-0.00050	-0.00103	0.00082	-0.00169	-0.00136	-0.00527	-0.00635	-0.00761	-0.01647	-0.03206	-0.05833	-0.08884	-0.11873
16384	0.00009	0.00039	-0.00122	-0.00178	-0.00218	-0.00360	-0.00947	-0.01206	-0.02275	-0.03997	-0.06373	-0.09700	-0.14333	
8192	-0.00064	-0.00010	-0.00152	-0.00244	-0.00527	-0.00741	-0.01709	-0.02175	-0.04363	-0.06883	-0.10482	-0.15384		
4096	-0.00144	-0.00171	-0.00099	-0.00447	-0.00850	-0.00891	-0.02365	-0.04416	-0.07100	-0.10970	-0.16239			
2048	-0.00503	-0.00465	-0.00506	-0.01137	-0.01410	-0.02833	-0.04261	-0.07405	-0.10978	-0.16447				
1024	-0.00605	-0.01171	-0.01087	-0.01968	-0.03758	-0.05508	-0.07819	-0.11561	-0.17786					
512	-0.00817	-0.01748	-0.01408	-0.02661	-0.05597	-0.08069	-0.13238	-0.18579						
256	-0.01480	-0.02788	-0.03676	-0.04695	-0.09294	-0.14056	-0.20418							
128	-0.01481	-0.04148	-0.06150	-0.08818	-0.14811	-0.21433								
64	-0.05578	-0.04289	-0.08611	-0.13664	-0.23464									
32	-0.03625	-0.10391	-0.14736	-0.22414										
16	-0.03924	-0.13829	-0.22951											
8	-0.09098	-0.23679												
4	-0.23296													

(d) $\mathcal{P}^{\mathcal{C}}$ for Scene: [**Veach Door**] Algorithm: [**MLT**] Metric: [**UQI**] True GT: [**BDPT @ 524288 spp**].

M-MLT	2	4	8	16	32	64	128	256	512	1024	2048	4096	8192	16384
GT (BDPT)														
262144	-0.00022	0.00028	-0.00030	0.00012	0.00039	0.00036	0.00050	-0.00058	-0.00141	-0.00105	-0.00257	-0.00329	-0.00678	-0.00944
131072	-0.00022	-0.00012	0.00041	-0.00038	-0.00006	-0.00020	-0.00148	-0.00070	-0.00251	-0.00383	-0.00674	-0.01266	-0.02190	-0.02790
65536	-0.00072	0.00019	0.00090	-0.00006	-0.00013	-0.00044	-0.00052	-0.00388	-0.00306	-0.00917	-0.01617	-0.02922	-0.04508	-0.06571
32768	-0.00039	-0.00070	-0.00003	-0.00063	0.00025	-0.00070	-0.00215	-0.00433	-0.00945	-0.01629	-0.03265	-0.05481	-0.08407	-0.11570
16384	-0.00170	0.00061	-0.00065	-0.00091	-0.00286	-0.00053	-0.00463	-0.01013	-0.01525	-0.03650	-0.05791	-0.09807	-0.13680	
8192	-0.00015	-0.00090	-0.00092	-0.00489	-0.00374	-0.00701	-0.01194	-0.02324	-0.03704	-0.06297	-0.10188	-0.14788		
4096	-0.00273	-0.00187	-0.00279	-0.00362	-0.00998	-0.01329	-0.02959	-0.04332	-0.07112	-0.10789	-0.16696			
2048	-0.00398	-0.00468	-0.00744	-0.00483	-0.01350	-0.02329	-0.04798	-0.07375	-0.10856	-0.17070				
1024	-0.00628	-0.00974	-0.01224	-0.01055	-0.03013	-0.04340	-0.07144	-0.11889	-0.17182					
512	-0.00610	-0.01096	-0.01608	-0.02684	-0.05777	-0.07892	-0.13215	-0.18966						
256	-0.01227	-0.02695	-0.03965	-0.06376	-0.09002	-0.13365	-0.19925							
128	-0.03603	-0.02722	-0.05189	-0.10037	-0.14855	-0.20910								
64	-0.02685	-0.04465	-0.09322	-0.15587	-0.23237									
32	-0.06152	-0.09836	-0.17699	-0.23046										
16	-0.05920	-0.13003	-0.25715											
8	-0.11478	-0.25647												
4	-0.26556													

(e) $\mathcal{P}^{\mathcal{C}}$ for Scene: [**Veach Door**] Algorithm: [**M-MLT**] Metric: [**UQI**] True GT: [**BDPT @ 524288 spp**].

ERPT	2	4	8	16	32	64	128	256	512	1024	2048	4096	8192	16384	32768	65536	131072
GT (BDPT)																	
262144	0.00029	0.00098	-0.00094	0.00020	-0.00114	0.00021	-0.00078	-0.00025	0.00031	0.00072	-0.00008	0.00054	-0.00064	-0.00060	0.00020	-0.00070	-0.00077
131072	-0.00059	0.00084	-0.00324	0.00003	-0.00156	-0.00191	-0.00144	-0.00031	-0.00122	-0.00137	-0.00020	-0.00157	-0.00086	-0.00065	0.00009	-0.00064	
65536	-0.00361	-0.00119	-0.00296	-0.00244	-0.00340	-0.00410	-0.00059	-0.00274	-0.00255	-0.00252	-0.00167	-0.00243	-0.00207	-0.00159	-0.00280		
32768	-0.00471	-0.00588	-0.00452	-0.00599	-0.00630	-0.00384	-0.00428	-0.00407	-0.00484	-0.00564	-0.00281	-0.00454	-0.00459	-0.00460			
16384	-0.00624	-0.00970	-0.01062	-0.01052	-0.00827	-0.00816	-0.00844	-0.00881	-0.00762	-0.00995	-0.00937	-0.00756	-0.00861				
8192	-0.00948	-0.01233	-0.01830	-0.01966	-0.01593	-0.01777	-0.01598	-0.01276	-0.01397	-0.02065	-0.01930	-0.01323					
4096	-0.02863	-0.02798	-0.03053	-0.03427	-0.02541	-0.02959	-0.03658	-0.02801	-0.03408	-0.02508	-0.02864						
2048	-0.05429	-0.05309	-0.05433	-0.06008	-0.05261	-0.05443	-0.04702	-0.05221	-0.05076	-0.04941							
1024	-0.08780	-0.09353	-0.08952	-0.09518	-0.09438	-0.09505	-0.09388	-0.09012	-0.09047								
512	-0.14800	-0.14872	-0.15748	-0.14895	-0.14543	-0.16080	-0.14343	-0.15439									
256	-0.22190	-0.22674	-0.22738	-0.22697	-0.23031	-0.23489	-0.23635										
128	-0.31615	-0.32718	-0.33190	-0.33514	-0.33906	-0.33032											
64	-0.43029	-0.43177	-0.44301	-0.44395	-0.44945												
32	-0.56624	-0.57412	-0.57492	-0.58429													
16	-0.72804	-0.74246	-0.74608														
8	-0.93571	-0.94756															
4	-1.19414																

(f) $\mathcal{P}^{\mathcal{C}}$ for Scene: [**Veach Door**] Algorithm: [**ERPT**] Metric: [**UQI**] True GT: [**BDPT @ 524288 spp**].

M-ERPT	2	4	8	16	32	64	128	256	512	1024	2048	4096	8192	16384	32768	65536	131072
GT (BDPT)																	
262144	0.00039	-0.00122	0.00007	0.00084	-0.00028	0.00070	-0.00143	0.00021	-0.00103	-0.00066	0.00027	-0.00077	0.00015	0.00074	-0.00091	0.00033	-0.00110
131072	-0.00115	-0.00083	-0.00060	-0.00042	0.00106	-0.00003	-0.00148	-0.00094	-0.00047	-0.00110	0.00089	-0.00069	-0.00123	-0.00115	-0.00131	0.00029	
65536	-0.00172	-0.00159	-0.00175	-0.00041	-0.00255	-0.00034	-0.00295	-0.00176	-0.00062	-0.00218	-0.00197	-0.00154	-0.00327	-0.00308	-0.00203		
32768	-0.00426	-0.00479	-0.00310	-0.00258	-0.00431	-0.00591	-0.00369	-0.00510	-0.00488	-0.00240	-0.00265	-0.00298	-0.00645	-0.00205			
16384	-0.00470	-0.00729	-0.00666	-0.00953	-0.00667	-0.00658	-0.00571	-0.00617	-0.00711	-0.00616	-0.00797	-0.00906	-0.00695				
8192	-0.00940	-0.01210	-0.00973	-0.01114	-0.01305	-0.01432	-0.01371	-0.01554	-0.01630	-0.01293	-0.01464	-0.02232					
4096	-0.02494	-0.03360	-0.02378	-0.02600	-0.02517	-0.03240	-0.02888	-0.02965	-0.02937	-0.02750	-0.02596						
2048	-0.05180	-0.05071	-0.05389	-0.04733	-0.04387	-0.05432	-0.05211	-0.05047	-0.04974	-0.05477							
1024	-0.07983	-0.08266	-0.09581	-0.08447	-0.08037	-0.09398	-0.08725	-0.08972	-0.07650								
512	-0.13061	-0.13405	-0.12877	-0.14495	-0.13971	-0.15019	-0.13523	-0.14389									
256	-0.22050	-0.21238	-0.21093	-0.21468	-0.21202	-0.21327	-0.21912										
128	-0.30254	-0.30282	-0.30618	-0.31326	-0.31705	-0.31136											
64	-0.41583	-0.41781	-0.42217	-0.43260	-0.42941												
32	-0.55849	-0.56491	-0.55601	-0.56391													
16	-0.71734	-0.71558	-0.72923														
8	-0.92161	-0.93377															
4	-1.18628																

(g) $\mathcal{P}^{\mathcal{C}}$ for Scene: [**Veach Door**] Algorithm: [**M-ERPT**] Metric: [**UQI**] True GT: [**BDPT @ 524288 spp**].

Table 94

20.5 Sponza

PT	2	4	8	16	32	64	128	256	512	1024	2048	4096	8192
**GT (PT)**													
16384	-0.00184	-0.00118	-0.00155	-0.00244	-0.00060	0.00108	0.00045	-0.00360	-0.00471	-0.00377	-0.00820	-0.01399	-0.01690
8192	0.00023	-0.00103	-0.00113	-0.00157	-0.00242	-0.00004	-0.00081	-0.00547	-0.01373	-0.01520	-0.02398	-0.03938	
4096	-0.00194	-0.00185	-0.00297	-0.00368	-0.00567	-0.00426	-0.00561	-0.01474	-0.02681	-0.03560	-0.05734		
2048	-0.00383	0.00037	0.00035	-0.00474	-0.00471	-0.00355	-0.01227	-0.02891	-0.05314	-0.06768			
1024	-0.00351	-0.00347	-0.00054	-0.01321	-0.00899	-0.01482	-0.03035	-0.05204	-0.09882				
512	-0.00897	-0.01323	-0.00583	-0.01289	-0.01805	-0.03195	-0.06942	-0.09798					
256	0.00118	-0.00628	-0.00864	-0.02878	-0.02191	-0.07840	-0.08919						
128	-0.00335	-0.01406	-0.02116	-0.05197	-0.06538	-0.14535							
64	-0.01442	-0.02349	-0.05628	-0.11219	-0.16145								
32	-0.01299	-0.05166	-0.07541	-0.16904									
16	-0.06690	-0.12032	-0.13125										
8	-0.11663	-0.21640											
4	-0.22071												

(a) $\mathcal{P}^{\mathcal{C}}$ for Scene: [**Sponza**] Algorithm: [**PT**] Metric: [**UQI**] True GT: [**PT @ 32768 spp**].

BDPT	2	4	8	16	32	64	128	256	512	1024	2048	4096	8192
**GT (PT)**													
16384	0.00058	0.00089	0.00021	0.00037	-0.00014	0.00061	-0.00014	-0.00150	-0.00293	-0.00689	-0.00782	-0.01227	-0.01707
8192	-0.00141	0.00003	-0.00184	0.00065	-0.00169	-0.00230	-0.00207	-0.00555	-0.00968	-0.01685	-0.02092	-0.03365	
4096	-0.00100	0.00163	-0.00144	0.00033	-0.00566	-0.00334	-0.00671	-0.01079	-0.01977	-0.03358	-0.05407		
2048	-0.00371	-0.00295	-0.00289	0.00021	-0.00587	-0.00742	-0.01491	-0.02755	-0.04157	-0.06679			
1024	0.00162	0.00384	-0.00706	-0.00572	-0.00804	-0.01539	-0.02418	-0.04595	-0.08042				
512	0.00240	-0.00740	-0.00554	-0.01509	-0.01623	-0.02770	-0.05752	-0.09412					
256	0.00498	-0.00755	-0.01060	-0.01727	-0.03729	-0.04612	-0.10211						
128	-0.00379	-0.01946	-0.03523	-0.02858	-0.07184	-0.11846							
64	0.00986	-0.02467	-0.03821	-0.07960	-0.13314								
32	0.01281	-0.09723	-0.08267	-0.14353									
16	-0.06806	-0.04816	-0.16637										
8	-0.10736	-0.15565											
4	-0.21128												

(b) $\mathcal{P}^{\mathcal{C}}$ for Scene: [**Sponza**] Algorithm: [**BDPT**] Metric: [**UQI**] True GT: [**PT @ 32768 spp**].

PSSMLT	2	4	8	16	32	64	128	256	512	1024	2048	4096	8192
**GT (PT)**													
16384	-0.00034	-0.00117	0.00116	-0.00071	0.00016	0.00244	-0.00068	-0.00050	-0.00242	-0.00363	-0.00713	-0.01164	-0.01808
8192	0.00050	-0.00143	-0.00108	-0.00405	-0.00155	0.00127	-0.00354	-0.00300	-0.00793	-0.01220	-0.01986	-0.03124	
4096	-0.00121	-0.00179	-0.00147	-0.00130	-0.00458	-0.00487	-0.00851	-0.01278	-0.01891	-0.02761	-0.04225		
2048	0.00562	0.00328	0.00162	-0.00735	-0.00460	-0.00599	-0.01524	-0.02388	-0.03667	-0.05928			
1024	0.00099	0.00055	-0.00235	-0.01322	0.00084	-0.02637	-0.02850	-0.04552	-0.07338				
512	-0.00259	-0.00582	-0.00864	-0.00898	-0.02636	-0.03704	-0.06050	-0.09641					
256	-0.00786	-0.00057	-0.01279	-0.03431	-0.03502	-0.09462	-0.09917						
128	-0.02997	-0.02013	-0.03385	-0.06644	-0.08505	-0.16056							
64	-0.03836	-0.05777	-0.08579	-0.14337	-0.16831								
32	-0.05340	-0.08572	-0.13388	-0.21136									
16	-0.13792	-0.19634	-0.25714										
8	-0.21720	-0.31203											
4	-0.35192												

(c) $\mathcal{P}^{\mathcal{C}}$ for Scene: [**Sponza**] Algorithm: [**PSSMLT**] Metric: [**UQI**] True GT: [**PT @ 32768 spp**].

MLT	2	4	8	16	32	64	128	256	512	1024	2048	4096	8192
**GT (PT)**													
16384	-0.00178	-0.00091	-0.00040	-0.00139	-0.00043	-0.00062	-0.00159	-0.00224	-0.00486	-0.01059	-0.01430	-0.01694	-0.02089
8192	-0.00063	0.00174	-0.00455	-0.00143	-0.00127	-0.00243	-0.00939	-0.00937	-0.01774	-0.03324	-0.04088	-0.04876	
4096	-0.00043	-0.00134	-0.00201	-0.00349	-0.00389	-0.00760	-0.01348	-0.02515	-0.03964	-0.06317	-0.07845		
2048	-0.00169	-0.00019	-0.00679	-0.00688	-0.00908	-0.02112	-0.03606	-0.04950	-0.07359	-0.11317			
1024	-0.00337	-0.00476	-0.00939	-0.02432	-0.02083	-0.04627	-0.07649	-0.09602	-0.12538				
512	-0.00998	-0.01186	-0.00937	-0.01896	-0.04885	-0.06532	-0.11464	-0.16760					
256	-0.00347	-0.01869	-0.03417	-0.08686	-0.09082	-0.14167	-0.21309						
128	-0.01462	-0.03140	-0.07017	-0.10991	-0.15598	-0.24061							
64	-0.03430	-0.06813	-0.10399	-0.17446	-0.24159								
32	-0.05701	-0.08593	-0.19498	-0.29000									
16	-0.10702	-0.20828	-0.28100										
8	-0.18569	-0.35670											
4	-0.36724												

(d) $\mathcal{P}^{\mathcal{C}}$ for Scene: [**Sponza**] Algorithm: [**MLT**] Metric: [**UQI**] True GT: [**PT @ 32768 spp**].

M-MLT	2	4	8	16	32	64	128	256	512	1024	2048	4096	8192
**GT (PT)**													
16384	-0.00101	-0.00012	-0.00047	0.00019	-0.00087	0.00173	-0.00114	-0.00296	-0.00610	-0.00825	-0.01418	-0.01788	-0.02194
8192	0.00225	0.00062	0.00131	-0.00068	-0.00358	-0.00242	-0.00677	-0.00853	-0.02190	-0.02720	-0.04014	-0.04866	
4096	-0.00261	-0.00021	-0.00078	-0.00187	-0.00557	-0.01018	-0.01478	-0.02909	-0.03999	-0.06196	-0.08093		
2048	-0.00112	0.00107	-0.00132	-0.00573	-0.01504	-0.01482	-0.03390	-0.04637	-0.08638	-0.11083			
1024	-0.00599	-0.00558	-0.00345	-0.00881	-0.02419	-0.03811	-0.06136	-0.08802	-0.15027				
512	-0.00996	-0.00838	-0.00914	-0.02217	-0.04525	-0.07713	-0.12228	-0.17314					
256	-0.02261	-0.01144	-0.01799	-0.04645	-0.07641	-0.14998	-0.19963						
128	-0.03018	-0.03865	-0.05647	-0.08606	-0.15719	-0.24711							
64	-0.05156	-0.06450	-0.11421	-0.17159	-0.27702								
32	-0.04873	-0.11321	-0.16483	-0.26600									
16	-0.14467	-0.22938	-0.36226										
8	-0.21866	-0.36525											
4	-0.41085												

(e) $\mathcal{P}^{\mathcal{C}}$ for Scene: [**Sponza**] Algorithm: [**M-MLT**] Metric: [**UQI**] True GT: [**PT @ 32768 spp**].

ERPT	2	4	8	16	32	64	128	256	512	1024	2048	4096	8192
**GT (PT)**													
16384	-0.00419	-0.00417	-0.00291	-0.00266	-0.00439	-0.00474	-0.00437	-0.00357	-0.00480	-0.00575	-0.00463	-0.00425	-0.00311
8192	-0.00997	-0.01127	-0.01140	-0.00787	-0.01064	-0.01221	-0.00908	-0.01027	-0.01006	-0.00984	-0.00911	-0.01074	
4096	-0.02262	-0.01814	-0.01808	-0.02026	-0.01996	-0.02362	-0.01823	-0.01741	-0.02082	-0.02247	-0.01898		
2048	-0.04093	-0.04702	-0.04426	-0.04062	-0.04283	-0.04863	-0.04409	-0.05055	-0.04968	-0.04614			
1024	-0.08136	-0.08976	-0.09122	-0.08063	-0.08573	-0.08382	-0.08666	-0.07795	-0.08647				
512	-0.13939	-0.13827	-0.13183	-0.13508	-0.13842	-0.14554	-0.13612	-0.13506					
256	-0.22369	-0.24312	-0.23782	-0.22844	-0.23762	-0.22871	-0.23975						
128	-0.35925	-0.37663	-0.37058	-0.36315	-0.36472	-0.36316							
64	-0.50971	-0.51157	-0.51990	-0.51594	-0.52223								
32	-0.68599	-0.70122	-0.68530	-0.68344									
16	-0.88991	-0.91101	-0.89078										
8	-1.14120	-1.15175											
4	-1.40768												

(f) $\mathcal{P}^{\mathcal{C}}$ for Scene: [**Sponza**] Algorithm: [**ERPT**] Metric: [**UQI**] True GT: [**PT @ 32768 spp**].

M-ERPT	2	4	8	16	32	64	128	256	512	1024	2048	4096	8192
**GT (PT)**													
16384	-0.00083	-0.00391	-0.00294	-0.00568	-0.00427	-0.00136	-0.00234	-0.00019	-0.00408	-0.00260	-0.00350	-0.00164	-0.00065
8192	-0.00769	-0.01080	-0.01018	-0.01025	-0.01044	-0.01210	-0.00882	-0.00718	-0.00631	-0.00779	-0.00974	-0.00756	
4096	-0.01963	-0.01989	-0.02092	-0.01733	-0.02032	-0.01804	-0.01973	-0.01547	-0.01422	-0.02021	-0.01683		
2048	-0.03809	-0.04402	-0.04039	-0.04054	-0.04186	-0.04137	-0.03421	-0.03195	-0.03980	-0.03440			
1024	-0.07620	-0.07768	-0.08183	-0.08126	-0.07436	-0.07987	-0.08010	-0.07739	-0.07507				
512	-0.12355	-0.13303	-0.12821	-0.12730	-0.13479	-0.13293	-0.13148	-0.12941					
256	-0.19769	-0.23307	-0.22525	-0.22998	-0.22448	-0.23263	-0.21550						
128	-0.34182	-0.36451	-0.34557	-0.34415	-0.35109	-0.35931							
64	-0.49602	-0.49900	-0.49410	-0.50057	-0.50070								
32	-0.65568	-0.68973	-0.67035	-0.69650									
16	-0.87365	-0.89036	-0.88422										
8	-1.12275	-1.14142											
4	-1.39471												

(g) $\mathcal{P}^{\mathcal{C}}$ for Scene: [**Sponza**] Algorithm: [**M-ERPT**] Metric: [**UQI**] True GT: [**PT @ 32768 spp**].

Table 95

21 IFC

21.1 Cornell Box

PT	2	4	8	16	32	64	128	256	512	1024	2048	4096	8192
GT (PT)													
16384	-0.01803	-0.02639	-0.02448	-0.00054	0.00240	-0.01761	0.00180	0.00692	-0.00609	-0.00729	-0.02568	-0.03299	-0.04785
8192	-0.03129	-0.05735	0.00459	0.00941	0.00427	0.00105	0.00480	0.00560	-0.01026	-0.01187	-0.04259	-0.07046	
4096	-0.02864	-0.04242	-0.00152	0.01217	-0.00347	0.01001	0.00479	-0.01165	-0.03180	-0.04324	-0.08914		
2048	-0.05128	-0.03683	-0.01865	-0.00326	-0.00376	-0.00102	0.00618	-0.02218	-0.04771	-0.09027			
1024	-0.06840	-0.04840	-0.03399	-0.01737	-0.02237	-0.02195	-0.01129	-0.04609	-0.09364				
512	-0.11948	-0.10169	-0.04907	-0.04814	-0.04209	-0.04829	-0.04380	-0.07935					
256	-0.13830	-0.13420	-0.09317	-0.07294	-0.06493	-0.07153	-0.11629						
128	-0.20599	-0.19968	-0.17101	-0.12095	-0.12319	-0.15263							
64	-0.29086	-0.25755	-0.21562	-0.16895	-0.19672								
32	-0.34629	-0.32023	-0.27725	-0.27522									
16	-0.41771	-0.39501	-0.37040										
8	-0.47984	-0.48401											
4	-0.53881												

(a) $\mathcal{P}^{\mathcal{C}}$ for Scene: [**Cornell Box**] Algorithm: [**PT**] Metric: [**IFC**] True GT: [**PT @ 32768 spp**].

BDPT	2	4	8	16	32	64	128	256	512	1024	2048	4096	8192
GT (PT)													
16384	-0.00319	-0.01020	0.00960	-0.00720	0.00205	-0.00382	-0.00449	-0.00329	-0.01952	-0.02709	-0.03163	-0.05261	-0.07140
8192	0.00000	0.00703	0.00062	-0.00256	0.00944	0.01098	-0.01494	-0.01258	-0.03196	-0.05238	-0.08415	-0.12197	
4096	0.00930	-0.02193	-0.00001	0.01282	-0.00120	-0.00557	-0.01532	-0.03795	-0.06579	-0.11457	-0.16721		
2048	-0.00342	0.00089	-0.00614	-0.01012	-0.00967	-0.01564	-0.04268	-0.05154	-0.11784	-0.17716			
1024	-0.05665	-0.03760	-0.02083	-0.00943	-0.01786	-0.02879	-0.07778	-0.10916	-0.19133				
512	-0.06410	-0.04893	-0.05190	-0.04940	-0.05462	-0.07441	-0.12887	-0.19090					
256	-0.09373	-0.07727	-0.06556	-0.09034	-0.10134	-0.13569	-0.23416						
128	-0.15710	-0.14671	-0.13981	-0.14979	-0.20378	-0.26507							
64	-0.22303	-0.20070	-0.22766	-0.24081	-0.28766								
32	-0.29973	-0.28969	-0.31365	-0.35583									
16	-0.37953	-0.37311	-0.41908										
8	-0.48467	-0.49669											
4	-0.56088												

(b) $\mathcal{P}^{\mathcal{C}}$ for Scene: [**Cornell Box**] Algorithm: [**BDPT**] Metric: [**IFC**] True GT: [**PT @ 32768 spp**].

PSSMLT	2	4	8	16	32	64	128	256	512	1024	2048	4096	8192
GT (PT)													
16384	-0.03082	-0.01829	-0.00155	-0.00222	-0.00504	-0.01510	-0.01691	-0.00944	-0.00797	-0.01730	-0.02324	-0.02573	-0.03959
8192	-0.02434	-0.02415	0.01512	0.00318	-0.01323	-0.01750	-0.00726	-0.01819	-0.01421	-0.03339	-0.04608	-0.06199	
4096	-0.02344	-0.01083	0.00467	0.00524	-0.00963	-0.00916	-0.01417	-0.02915	-0.03940	-0.06659	-0.08665		
2048	-0.03882	-0.01792	-0.00169	0.00360	-0.01700	-0.01669	-0.02890	-0.04570	-0.07073	-0.10881			
1024	-0.07888	-0.06358	-0.03022	-0.01795	-0.02304	-0.03520	-0.06207	-0.08531	-0.12070				
512	-0.11304	-0.09738	-0.03797	-0.03504	-0.05570	-0.07832	-0.11141	-0.16128					
256	-0.16860	-0.14397	-0.08544	-0.07864	-0.10554	-0.14267	-0.19918						
128	-0.24649	-0.19123	-0.13827	-0.14774	-0.19175	-0.24868							
64	-0.29514	-0.25648	-0.21396	-0.23079	-0.29088								
32	-0.35525	-0.33129	-0.30134	-0.33058									
16	-0.42224	-0.41119	-0.40106										
8	-0.49880	-0.51766											
4	-0.60465												

(c) $\mathcal{P}^{\mathcal{C}}$ for Scene: [**Cornell Box**] Algorithm: [**PSSMLT**] Metric: [**IFC**] True GT: [**PT @ 32768 spp**].

MLT	2	4	8	16	32	64	128	256	512	1024	2048	4096	8192
GT (PT)													
16384	-0.06861	-0.03183	0.00709	0.00390	-0.00735	0.00058	-0.00234	0.00055	-0.00322	-0.00147	-0.00129	-0.00751	-0.01204
8192	-0.03882	-0.00599	0.00676	0.00052	-0.00347	0.01386	-0.00224	-0.00761	-0.01240	-0.01113	-0.02067	-0.03374	
4096	-0.03738	-0.02542	0.00613	0.01484	-0.01539	-0.00352	-0.00586	-0.00119	-0.01883	-0.01893	-0.02461		
2048	-0.05634	-0.02565	0.00342	0.00633	-0.00832	0.00125	-0.00669	-0.00618	-0.02651	-0.03518			
1024	-0.08568	-0.05522	-0.02724	-0.01943	-0.03426	-0.01750	-0.01577	-0.01352	-0.03804				
512	-0.19564	-0.11374	-0.06213	-0.05788	-0.06079	-0.05218	-0.04273	-0.05478					
256	-0.22937	-0.19849	-0.14852	-0.10433	-0.10706	-0.08332	-0.08260						
128	-0.28781	-0.23022	-0.17608	-0.14327	-0.13786	-0.13244							
64	-0.37880	-0.32849	-0.26826	-0.23027	-0.20556								
32	-0.45524	-0.38931	-0.31448	-0.28371									
16	-0.51407	-0.45549	-0.37866										
8	-0.57750	-0.53562											
4	-0.65610												

(d) $\mathcal{P}^{\mathcal{C}}$ for Scene: [**Cornell Box**] Algorithm: [**MLT**] Metric: [**IFC**] True GT: [**PT @ 32768 spp**].

M-MLT	2	4	8	16	32	64	128	256	512	1024	2048	4096	8192
GT (PT)													
16384	-0.03832	-0.00286	0.00058	0.00898	0.02483	0.00338	0.00542	-0.00247	-0.00021	-0.01056	-0.01120	-0.01158	-0.01725
8192	-0.02608	-0.00597	0.00304	0.01016	0.02745	0.00704	0.01312	0.00643	-0.01190	-0.00577	-0.02520	-0.01898	
4096	-0.08660	-0.04025	-0.03434	-0.01340	0.00640	-0.01170	-0.01086	-0.00937	-0.01428	-0.02709	-0.04739		
2048	-0.07201	-0.05373	-0.04796	-0.03788	-0.00784	0.00321	0.00028	-0.01241	-0.01639	-0.03357			
1024	-0.11181	-0.06576	-0.05412	-0.04953	-0.03601	-0.02822	-0.02925	-0.04931	-0.05067				
512	-0.14947	-0.10359	-0.08512	-0.06923	-0.04541	-0.04060	-0.04804	-0.06123					
256	-0.24232	-0.18369	-0.16057	-0.12674	-0.08404	-0.08467	-0.08929						
128	-0.32209	-0.22034	-0.20666	-0.18264	-0.14830	-0.14701							
64	-0.34106	-0.30757	-0.25530	-0.22868	-0.18987								
32	-0.48556	-0.39953	-0.35267	-0.32420									
16	-0.51734	-0.46655	-0.41311										
8	-0.62608	-0.52940											
4	-0.65031												

(e) $\mathcal{P}^{\mathcal{C}}$ for Scene: [**Cornell Box**] Algorithm: [**M-MLT**] Metric: [**IFC**] True GT: [**PT @ 32768 spp**].

ERPT	2	4	8	16	32	64	128	256	512	1024	2048	4096	8192
GT (PT)													
16384	0.00397	-0.01143	0.01234	-0.01394	0.00661	0.00412	-0.00262	0.00171	-0.01062	0.01316	0.01283	0.00683	-0.01319
8192	-0.01346	0.00872	-0.00717	0.00659	-0.00442	0.00062	0.01659	-0.00677	-0.01051	0.00011	0.00879	-0.00303	
4096	-0.01271	-0.02777	0.01001	-0.02675	-0.00556	-0.01726	-0.01250	-0.01077	-0.01397	0.01535	-0.00961		
2048	-0.00255	-0.00023	0.00006	-0.00753	-0.00380	0.00152	-0.00384	-0.00393	-0.01223	0.00119			
1024	-0.02840	-0.01812	0.01025	-0.01611	-0.00866	-0.01914	-0.01428	-0.02728	-0.02998				
512	-0.04873	-0.04269	-0.03276	-0.03925	-0.05441	-0.04455	-0.04225	-0.06002					
256	-0.07716	-0.11098	-0.08409	-0.09403	-0.09014	-0.10173	-0.08086						
128	-0.16018	-0.15939	-0.15205	-0.15896	-0.15642	-0.17091							
64	-0.24769	-0.24724	-0.22748	-0.24666	-0.23225								
32	-0.37338	-0.36923	-0.35932	-0.37872									
16	-0.48796	-0.50484	-0.50787										
8	-0.66835	-0.69185											
4	-0.83293												

(f) $\mathcal{P}^{\mathcal{C}}$ for Scene: [**Cornell Box**] Algorithm: [**ERPT**] Metric: [**IFC**] True GT: [**PT @ 32768 spp**].

M-ERPT	2	4	8	16	32	64	128	256	512	1024	2048	4096	8192
GT (PT)													
16384	0.01651	-0.01776	-0.00687	0.00369	0.00882	-0.00708	-0.00906	-0.01205	-0.00502	0.00127	0.01125	-0.00836	0.00060
8192	0.00091	-0.00487	-0.00173	-0.01575	-0.00027	-0.00601	0.00213	0.00213	0.00814	-0.01134	-0.00355	-0.01003	
4096	0.01584	-0.01274	-0.02349	-0.00675	-0.01548	-0.01853	-0.00209	0.01358	-0.00738	-0.00231	0.00056		
2048	0.01179	-0.01562	-0.02021	-0.00067	-0.02027	-0.02836	0.00310	0.01438	-0.00076	-0.01164			
1024	-0.00086	-0.02290	-0.02570	-0.02057	-0.00920	-0.02851	-0.01432	-0.01644	-0.02470				
512	-0.02041	-0.05905	-0.04410	-0.04007	-0.05793	-0.05121	-0.04609	-0.01697					
256	-0.08044	-0.08957	-0.09319	-0.07674	-0.09926	-0.09450	-0.08699						
128	-0.14169	-0.16517	-0.18233	-0.16726	-0.16724	-0.17033							
64	-0.24839	-0.25253	-0.25463	-0.23730	-0.26012								
32	-0.34785	-0.37484	-0.37201	-0.35442									
16	-0.47177	-0.50646	-0.49867										
8	-0.65100	-0.67549											
4	-0.81264												

(g) $\mathcal{P}^{\mathcal{C}}$ for Scene: [**Cornell Box**] Algorithm: [**M-ERPT**] Metric: [**IFC**] True GT: [**PT @ 32768 spp**].

Table 96

21.2 Torus

PT	2	4	8	16	32	64	128	256	512	1024	2048	4096	8192	16384	32768	65536	131072
GT (BDPT)																	
262144	0.02017	0.02128	0.00688	0.01109	0.00729	-0.00807	0.00450	0.01315	0.01526	0.02177	-0.00002	0.00295	0.00147	0.01038	0.00664	-0.01773	-0.05212
131072	-0.00179	-0.00650	0.01379	0.00647	-0.00558	-0.00083	-0.00626	0.00882	0.00058	0.00923	0.00539	-0.00738	-0.00239	-0.01539	-0.03145	-0.06394	
65536	0.00106	-0.00369	-0.00746	-0.00068	-0.01331	-0.03081	-0.02022	-0.00761	-0.02379	-0.00869	-0.01465	-0.02515	-0.03365	-0.04752	-0.06971		
32768	-0.03634	-0.02063	-0.04162	-0.04819	-0.05283	-0.06132	-0.05316	-0.04755	-0.05957	-0.03228	-0.03447	-0.05962	-0.07614	-0.11270			
16384	-0.03918	-0.05982	-0.08997	-0.07807	-0.08559	-0.11815	-0.09739	-0.10121	-0.10114	-0.09022	-0.08297	-0.11824	-0.14180				
8192	-0.11763	-0.12313	-0.13165	-0.16343	-0.16409	-0.19837	-0.17680	-0.19022	-0.17545	-0.16293	-0.15262	-0.19683					
4096	-0.18081	-0.16900	-0.20046	-0.21776	-0.22601	-0.27628	-0.24159	-0.24879	-0.25089	-0.21757	-0.24045						
2048	-0.20108	-0.21410	-0.27802	-0.28447	-0.29255	-0.35226	-0.33935	-0.33297	-0.30744	-0.28280							
1024	-0.20501	-0.24655	-0.27296	-0.33872	-0.31519	-0.36924	-0.35057	-0.31620	-0.35665								
512	-0.24015	-0.27693	-0.33111	-0.33909	-0.36193	-0.38835	-0.36915	-0.38242									
256	-0.22003	-0.26549	-0.29740	-0.31302	-0.31207	-0.36418	-0.35061										
128	-0.18099	-0.16608	-0.18776	-0.20676	-0.19460	-0.28199											
64	-0.17398	-0.18685	-0.18322	-0.19643	-0.17084												
32	-0.10807	-0.09343	-0.12923	-0.17411													
16	-0.07257	-0.08573	-0.10665														
8	-0.08045	-0.08922															
4	-0.11585																

(a) $\mathcal{P}^{\mathcal{C}}$ for Scene: [**Torus**] Algorithm: [**PT**] Metric: [**IFC**] True GT: [**BDPT @ 524288 spp**].

BDPT	2	4	8	16	32	64	128	256	512	1024	2048	4096	8192	16384	32768	65536	131072
GT (BDPT)																	
262144	0.01562	0.02029	0.01515	0.00872	-0.00144	0.00592	-0.00047	0.01293	0.00415	-0.00344	-0.00263	0.01383	0.00806	0.00733	-0.00673	-0.02045	-0.04748
131072	0.00749	0.01176	0.00906	0.02273	0.00658	-0.00036	-0.01951	0.00589	-0.00481	0.00358	-0.00723	0.00509	-0.00252	-0.00723	-0.02596	-0.05967	
65536	0.02028	0.00054	-0.00812	-0.00782	-0.02098	-0.01501	-0.02309	-0.01261	-0.02924	-0.01344	-0.01426	-0.00698	-0.02010	-0.05161	-0.08571		
32768	-0.03127	-0.03926	-0.02327	-0.03256	-0.04424	-0.04716	-0.06003	-0.05343	-0.03617	-0.05288	-0.05832	-0.05378	-0.07047	-0.10768			
16384	-0.04603	-0.04439	-0.06404	-0.06471	-0.08852	-0.10562	-0.10589	-0.08957	-0.10085	-0.11167	-0.09513	-0.11070	-0.11125				
8192	-0.10458	-0.10534	-0.13442	-0.12634	-0.14651	-0.17160	-0.19514	-0.15552	-0.17828	-0.16746	-0.14519	-0.18201					
4096	-0.16819	-0.16930	-0.16386	-0.18905	-0.22331	-0.23652	-0.23948	-0.23949	-0.24251	-0.22588	-0.20346						
2048	-0.21504	-0.21412	-0.21767	-0.24131	-0.29627	-0.32316	-0.34313	-0.29967	-0.29740	-0.30177							
1024	-0.23616	-0.25083	-0.26774	-0.28383	-0.34128	-0.33349	-0.34991	-0.30684	-0.33008								
512	-0.25367	-0.27413	-0.29197	-0.30414	-0.34431	-0.34572	-0.38722	-0.34636									
256	-0.22171	-0.26551	-0.27485	-0.25261	-0.32427	-0.34106	-0.31229										
128	-0.17182	-0.17878	-0.16647	-0.16026	-0.19672	-0.22846											
64	-0.15651	-0.16856	-0.13410	-0.13722	-0.18593												
32	-0.09826	-0.09231	-0.12490	-0.09011													
16	-0.06435	-0.07687	-0.07407														
8	-0.06432	-0.07957															
4	-0.10803																

(b) $\mathcal{P}^{\mathcal{C}}$ for Scene: [**Torus**] Algorithm: [**BDPT**] Metric: [**IFC**] True GT: [**BDPT @ 524288 spp**].

PSSMLT	2	4	8	16	32	64	128	256	512	1024	2048	4096	8192	16384
GT (BDPT)														
262144	0.01349	0.02028	0.01390	0.01065	0.00365	0.01230	0.01449	0.01284	0.00375	-0.00056	0.00739	0.00280	-0.00914	-0.00920
131072	-0.00386	0.01202	0.00474	0.00284	-0.00420	0.00093	-0.00587	-0.00162	0.00291	-0.00726	-0.01406	-0.01574	-0.03574	-0.04310
65536	-0.00601	0.00826	-0.01891	-0.01969	-0.01993	-0.01746	-0.02107	-0.02048	-0.02669	-0.03042	-0.03042	-0.05055	-0.07796	-0.10712
32768	-0.02968	-0.01923	-0.02578	-0.02145	-0.04528	-0.03706	-0.04852	-0.05588	-0.07117	-0.09033	-0.08854	-0.10875	-0.15915	-0.20121
16384	-0.08244	-0.06696	-0.08907	-0.10110	-0.10183	-0.11173	-0.12171	-0.11907	-0.13217	-0.15474	-0.16639	-0.20631	-0.26233	
8192	-0.11820	-0.12356	-0.12878	-0.13643	-0.14511	-0.17945	-0.19276	-0.21096	-0.24183	-0.25383	-0.28678	-0.33336		
4096	-0.14728	-0.16251	-0.17480	-0.18777	-0.21524	-0.22994	-0.25283	-0.26477	-0.30426	-0.33518	-0.38192			
2048	-0.22621	-0.24608	-0.26995	-0.29270	-0.30693	-0.33521	-0.35430	-0.39459	-0.42130	-0.47491				
1024	-0.23097	-0.25882	-0.30775	-0.31863	-0.35105	-0.37373	-0.40966	-0.43676	-0.48562					
512	-0.31333	-0.32339	-0.34545	-0.34284	-0.37660	-0.41787	-0.46584	-0.49186						
256	-0.28178	-0.30355	-0.34575	-0.36050	-0.38258	-0.43082	-0.46610							
128	-0.21279	-0.21792	-0.24850	-0.26260	-0.31239	-0.36046								
64	-0.23933	-0.25489	-0.25988	-0.27127	-0.32872									
32	-0.21520	-0.21659	-0.27415	-0.26829										
16	-0.19397	-0.20145	-0.25815											
8	-0.21682	-0.24246												
4	-0.29239													

(c) $\mathcal{P}^{\mathcal{C}}$ for Scene: [**Torus**] Algorithm: [**PSSMLT**] Metric: [**IFC**] True GT: [**BDPT @ 524288 spp**].

MLT	2	4	8	16	32	64	128	256	512	1024	2048	4096	8192	16384
GT (BDPT)														
262144	0.02038	0.00555	0.03605	0.00476	0.00468	0.00950	0.01020	0.00759	0.01166	-0.00623	-0.00398	-0.01145	-0.01463	-0.01463
131072	0.00099	0.00312	0.00115	-0.00777	-0.01070	-0.00352	-0.00617	-0.01147	-0.03353	-0.03849	-0.04061	-0.05829	-0.06247	-0.06735
65536	-0.01357	-0.02514	-0.00428	-0.01621	-0.02947	-0.03571	-0.03302	-0.04807	-0.07176	-0.07935	-0.08468	-0.10991	-0.12971	-0.12977
32768	-0.05790	-0.05130	-0.02651	-0.05523	-0.08347	-0.06636	-0.08651	-0.10982	-0.13169	-0.15792	-0.16386	-0.18545	-0.21295	-0.21153
16384	-0.09283	-0.10389	-0.05548	-0.09738	-0.11760	-0.12609	-0.15778	-0.17082	-0.21833	-0.24726	-0.26781	-0.28908	-0.31635	
8192	-0.15525	-0.15604	-0.15168	-0.16550	-0.20192	-0.22670	-0.26010	-0.28923	-0.33847	-0.37717	-0.39251	-0.41733		
4096	-0.24328	-0.23459	-0.21864	-0.24649	-0.26987	-0.32390	-0.35988	-0.40510	-0.45298	-0.49503	-0.51713			
2048	-0.26873	-0.30064	-0.27835	-0.30986	-0.35721	-0.43191	-0.48668	-0.52262	-0.57595	-0.63025				
1024	-0.33599	-0.36433	-0.31886	-0.38948	-0.43692	-0.50510	-0.56213	-0.59046	-0.66747					
512	-0.37225	-0.41278	-0.36273	-0.43099	-0.48131	-0.54924	-0.63316	-0.69273						
256	-0.36084	-0.37333	-0.37215	-0.42719	-0.51982	-0.58147	-0.65532							
128	-0.27687	-0.29807	-0.28679	-0.36483	-0.42064	-0.48486								
64	-0.30919	-0.33741	-0.29410	-0.41321	-0.45400									
32	-0.25728	-0.33620	-0.27684	-0.37366										
16	-0.25201	-0.31620	-0.27108											
8	-0.33151	-0.40433												
4	-0.43882													

(d) $\mathcal{P}^{\mathcal{C}}$ for Scene: [**Torus**] Algorithm: [**MLT**] Metric: [**IFC**] True GT: [**BDPT @ 524288 spp**].

M-MLT	2	4	8	16	32	64	128	256	512	1024	2048	4096	8192	16384
GT (BDPT)														
262144	0.02581	0.02893	0.00001	0.04199	0.02429	0.01549	0.02056	0.00304	0.00546	-0.00590	-0.00775	-0.01489	-0.02044	-0.03660
131072	-0.00819	0.01284	0.00785	0.03419	0.03068	-0.00146	0.00471	-0.00455	-0.02610	-0.03852	-0.03688	-0.06176	-0.07392	-0.08644
65536	-0.02120	0.01379	-0.01558	-0.02001	-0.00418	-0.03934	-0.03890	-0.06215	-0.06379	-0.08902	-0.09055	-0.11406	-0.13630	-0.16125
32768	-0.06349	-0.04474	-0.05320	-0.05364	-0.04509	-0.08392	-0.07465	-0.10355	-0.12853	-0.15715	-0.17760	-0.19941	-0.23138	-0.25590
16384	-0.12429	-0.07141	-0.09025	-0.08378	-0.08411	-0.14841	-0.14577	-0.17784	-0.22234	-0.26693	-0.27736	-0.31384	-0.35275	
8192	-0.19508	-0.14806	-0.16555	-0.17782	-0.18360	-0.23426	-0.24484	-0.30964	-0.34612	-0.37949	-0.41168	-0.46616		
4096	-0.26465	-0.20293	-0.24463	-0.22826	-0.23880	-0.33320	-0.33459	-0.40985	-0.45165	-0.50239	-0.53294			
2048	-0.35622	-0.25363	-0.28899	-0.33850	-0.35570	-0.43465	-0.45089	-0.52146	-0.58821	-0.64390				
1024	-0.41380	-0.31216	-0.37132	-0.38589	-0.39107	-0.51909	-0.52433	-0.60694	-0.65406					
512	-0.48053	-0.33350	-0.40084	-0.47414	-0.43226	-0.58693	-0.61089	-0.68684						
256	-0.44300	-0.32416	-0.41696	-0.46772	-0.45520	-0.59306	-0.64441							
128	-0.32891	-0.23666	-0.37416	-0.40631	-0.38825	-0.51847								
64	-0.36807	-0.28746	-0.39213	-0.45190	-0.41789									
32	-0.32701	-0.24199	-0.38781	-0.41778										
16	-0.36233	-0.24861	-0.37496											
8	-0.44599	-0.27836												
4	-0.57655													

(e) $\mathcal{P}^{\mathcal{C}}$ for Scene: [**Torus**] Algorithm: [**M-MLT**] Metric: [**IFC**] True GT: [**BDPT @ 524288 spp**].

ERPT	2	4	8	16	32	64	128	256	512	1024	2048	4096	8192	16384	32768	65536	131072
GT (BDPT)																	
262144	0.00898	0.00951	0.00437	-0.00078	0.00075	-0.00361	0.00119	0.01360	0.01221	0.01290	0.00725	-0.00014	0.00749	0.00549	0.01294	0.00813	0.00875
131072	-0.00409	-0.00497	-0.00564	-0.01807	-0.00664	-0.02046	-0.01032	0.00535	-0.00813	-0.01188	-0.00173	-0.01307	-0.01575	-0.00579	0.00001	-0.00972	
65536	-0.01456	-0.04663	-0.03041	-0.03091	-0.02285	-0.03986	-0.03039	-0.01762	-0.02615	-0.03337	-0.03277	-0.04298	-0.02899	-0.04322	-0.02422		
32768	-0.07635	-0.06654	-0.07672	-0.07853	-0.07038	-0.08148	-0.08210	-0.05914	-0.06855	-0.07670	-0.07709	-0.10343	-0.09863	-0.07585			
16384	-0.13452	-0.13466	-0.14699	-0.14569	-0.13499	-0.15760	-0.14018	-0.12960	-0.13752	-0.14339	-0.15645	-0.17511	-0.16684				
8192	-0.22374	-0.22401	-0.24249	-0.23779	-0.23232	-0.24614	-0.24701	-0.23449	-0.22924	-0.25519	-0.26165	-0.27573					
4096	-0.31008	-0.29621	-0.31884	-0.32811	-0.31475	-0.34339	-0.32935	-0.33166	-0.34230	-0.36591	-0.36924						
2048	-0.40221	-0.40572	-0.44205	-0.43408	-0.44764	-0.44094	-0.44922	-0.44878	-0.45340	-0.49545							
1024	-0.45635	-0.49809	-0.50353	-0.50841	-0.52896	-0.52411	-0.55555	-0.54550	-0.56923								
512	-0.50264	-0.54890	-0.55846	-0.58320	-0.59649	-0.60727	-0.62937	-0.63303									
256	-0.50855	-0.54963	-0.56665	-0.59193	-0.61421	-0.64087	-0.69410										
128	-0.39740	-0.44585	-0.48726	-0.52902	-0.55306	-0.58435											
64	-0.41175	-0.44780	-0.50455	-0.54377	-0.54154												
32	-0.38633	-0.44794	-0.49679	-0.52509													
16	-0.38245	-0.47283	-0.52420														
8	-0.47085	-0.55854															
4	-0.57979																

(f) $\mathcal{P}^{\mathcal{C}}$ for Scene: [**Torus**] Algorithm: [**ERPT**] Metric: [**IFC**] True GT: [**BDPT @ 524288 spp**].

M-ERPT	2	4	8	16	32	64	128	256	512	1024	2048	4096	8192	16384	32768	65536	131072
GT (BDPT)																	
262144	0.00679	0.00853	-0.00105	0.01186	0.00452	0.01257	-0.00266	0.00404	0.01132	0.00161	-0.00557	-0.00256	-0.00214	0.00194	0.00392	0.00321	0.00654
131072	-0.00880	-0.01481	-0.00750	-0.00654	-0.00923	0.00019	0.00024	-0.01186	-0.02034	-0.00675	-0.00628	-0.01668	-0.01156	-0.02434	-0.02348	-0.01943	
65536	-0.03307	-0.03635	-0.03751	-0.03399	-0.03677	-0.03317	-0.03264	-0.03679	-0.03599	-0.03363	-0.04318	-0.04425	-0.03740	-0.05325	-0.04951		
32768	-0.07577	-0.07179	-0.06435	-0.06198	-0.07263	-0.07279	-0.07907	-0.09006	-0.09630	-0.08486	-0.09807	-0.09498	-0.10083	-0.10264			
16384	-0.12287	-0.13827	-0.13568	-0.13199	-0.13983	-0.14069	-0.15068	-0.15384	-0.15551	-0.16399	-0.17160	-0.16808	-0.17662				
8192	-0.20115	-0.21059	-0.23867	-0.23713	-0.20691	-0.22793	-0.25542	-0.25450	-0.26663	-0.27413	-0.29583	-0.26568					
4096	-0.29841	-0.30071	-0.31063	-0.33075	-0.30751	-0.33734	-0.33505	-0.34281	-0.35794	-0.38288	-0.38353						
2048	-0.39337	-0.39161	-0.42883	-0.43765	-0.43158	-0.43533	-0.45818	-0.47290	-0.48668	-0.50517							
1024	-0.46999	-0.44921	-0.47070	-0.52042	-0.48665	-0.54595	-0.57343	-0.56026	-0.59561								
512	-0.50395	-0.50429	-0.53548	-0.59218	-0.54270	-0.60582	-0.63558	-0.65908									
256	-0.48093	-0.52243	-0.53247	-0.59228	-0.56732	-0.64608	-0.67150										
128	-0.38811	-0.40603	-0.44531	-0.51390	-0.49891	-0.60151											
64	-0.40359	-0.40330	-0.44482	-0.53967	-0.48859												
32	-0.35457	-0.39939	-0.45505	-0.51350													
16	-0.34608	-0.43468	-0.45419														
8	-0.42219	-0.49239															
4	-0.51712																

(g) $\mathcal{P}^{\mathcal{C}}$ for Scene: [**Torus**] Algorithm: [**M-ERPT**] Metric: [**IFC**] True GT: [**BDPT @ 524288 spp**].

Table 97

21.3 Veach Bidir

PT	2	4	8	16	32	64	128	256	512	1024	2048	4096	8192	16384	32768	65536	131072
GT (BDPT)																	
262144	-0.02658	0.03040	0.01458	-0.03594	-0.01715	0.00990	0.00222	-0.01700	0.00270	-0.00973	-0.01071	0.00976	0.00046	0.00878	0.00450	-0.00387	0.00103
131072	-0.03460	0.00675	0.01604	-0.04894	-0.00492	0.01290	0.01650	-0.01018	0.02391	-0.01406	-0.00621	-0.00130	0.00992	0.00777	-0.00048	-0.00127	
65536	-0.00587	0.00799	0.02368	-0.04869	0.01545	0.02379	0.01564	0.00041	0.00281	-0.00647	-0.00610	-0.00895	0.00824	0.00459	-0.00168		
32768	0.03234	0.01195	-0.00708	-0.01712	-0.00652	-0.00130	0.01690	0.00331	0.00892	-0.01485	-0.01206	-0.00063	0.01143	-0.00695			
16384	0.02458	-0.01436	0.03498	-0.00105	0.01877	0.00006	0.01672	0.00086	0.01647	-0.00221	-0.00015	0.01223	0.00911				
8192	0.00056	-0.00709	0.02452	0.01157	-0.00478	0.01172	0.02657	0.00714	0.00464	-0.00853	-0.01379	0.00011					
4096	0.01360	0.01804	0.01341	0.00366	-0.00354	-0.01074	0.00310	-0.00875	-0.00411	-0.00447	-0.01602						
2048	-0.00739	-0.02353	-0.01346	0.00938	-0.01527	0.00864	-0.00886	-0.01384	-0.00835	-0.03151							
1024	-0.01234	-0.04410	-0.01010	-0.01579	-0.02571	0.00545	0.01761	0.00007	0.00217								
512	-0.02372	-0.03759	-0.02163	-0.06030	-0.02523	-0.00493	-0.00392	-0.03050									
256	-0.09923	-0.07312	-0.06675	-0.06029	-0.02696	-0.03094	-0.04776										
128	-0.09519	-0.09094	-0.05927	-0.10375	-0.06127	-0.05409											
64	-0.17526	-0.14482	-0.11237	-0.12203	-0.11775												
32	-0.22461	-0.23477	-0.17409	-0.20744													
16	-0.31785	-0.29499	-0.28798														
8	-0.42348	-0.39452															
4	-0.46223																

(a) $\mathcal{P}^{\mathcal{C}}$ for Scene: [**Veach Bidir**] Algorithm: [**PT**] Metric: [**IFC**] True GT: [**BDPT @ 524288 spp**].

BDPT	2	4	8	16	32	64	128	256	512	1024	2048	4096	8192	16384	32768	65536	131072
GT (BDPT)																	
262144	0.00220	0.00569	-0.00973	-0.00756	0.00385	0.00212	0.00099	-0.00179	0.00180	-0.00079	-0.00226	-0.00141	0.00065	0.00163	0.00584	0.00553	0.00621
131072	0.01828	-0.00006	0.00434	-0.00649	0.00645	0.00824	-0.00500	-0.00165	0.00308	-0.00462	-0.00575	-0.00465	-0.00014	0.00236	-0.00186	-0.00450	
65536	0.01101	0.00923	-0.00653	-0.01079	0.01151	0.00419	-0.00226	-0.00176	-0.00037	-0.01294	-0.00575	-0.01258	-0.01541	-0.02422	-0.04157		
32768	0.02923	-0.00269	0.00485	-0.00672	-0.00159	0.00442	-0.00767	-0.00582	0.00259	-0.00999	-0.01694	-0.02481	-0.04055	-0.06600			
16384	0.02132	0.01755	0.02803	0.00340	0.00221	0.01528	0.00141	-0.00004	-0.00322	-0.01667	-0.02718	-0.04614	-0.06983				
8192	0.01093	0.02520	0.02670	-0.00641	0.00888	0.00965	-0.00055	-0.00594	-0.01296	-0.03456	-0.05111	-0.07739					
4096	0.02174	0.01418	0.00299	0.00691	0.00041	-0.00404	-0.01402	-0.01804	-0.03088	-0.05972	-0.08898						
2048	0.02376	0.01598	0.00953	0.00555	0.00179	0.00889	-0.01088	-0.02465	-0.05102	-0.09624							
1024	0.00694	0.00393	0.02196	0.01229	0.00833	0.00572	-0.02191	-0.03940	-0.07733								
512	0.00553	0.00157	0.01411	0.00237	0.00569	0.00288	-0.04331	-0.08892									
256	-0.02102	0.00329	-0.00330	-0.01448	-0.02247	-0.03591	-0.09143										
128	-0.02262	-0.02126	-0.02881	-0.03657	-0.05207	-0.08764											
64	-0.06305	-0.06415	-0.06271	-0.09327	-0.12367												
32	-0.11049	-0.12157	-0.13019	-0.14570													
16	-0.17871	-0.19465	-0.20641														
8	-0.25274	-0.26749															
4	-0.33302																

(b) $\mathcal{P}^{\mathcal{C}}$ for Scene: [**Veach Bidir**] Algorithm: [**BDPT**] Metric: [**IFC**] True GT: [**BDPT @ 524288 spp**].

PSSMLT	2	4	8	16	32	64	128	256	512	1024	2048	4096	8192	16384
GT (BDPT)														
262144	-0.01709	0.00151	0.01205	0.01005	0.01137	0.01089	0.01076	0.01062	0.00429	-0.00070	-0.00578	0.00026	-0.00403	-0.00466
131072	-0.00395	-0.00638	0.01449	0.00559	0.01162	0.01047	0.01463	0.00620	-0.00296	-0.00629	-0.00770	-0.00381	-0.01048	-0.01382
65536	-0.01127	0.00055	0.00145	-0.00007	0.01073	0.00450	0.00259	-0.00157	-0.00329	-0.01109	-0.01446	-0.01464	-0.01832	-0.02926
32768	-0.01691	-0.00700	-0.00303	-0.00136	-0.00187	-0.01066	-0.00679	-0.00253	-0.00430	-0.01594	-0.01940	-0.02214	-0.03967	-0.05847
16384	-0.01125	-0.00319	0.00614	0.01124	0.01328	0.01299	0.01289	0.00153	-0.00547	-0.01009	-0.02420	-0.03478	-0.06079	
8192	-0.01832	-0.00332	0.00560	0.00990	0.00898	0.00378	0.00139	-0.00041	-0.00559	-0.02088	-0.03664	-0.06107		
4096	-0.00682	-0.00608	0.00077	0.00136	0.00205	-0.00099	-0.00250	-0.00558	-0.01832	-0.03944	-0.06634			
2048	-0.01529	0.00492	0.00409	-0.00471	0.01244	-0.00537	-0.00555	-0.01873	-0.03797	-0.06041				
1024	-0.02151	-0.00987	0.00815	0.00630	0.00843	0.00855	-0.00125	-0.01473	-0.04463					
512	-0.02604	-0.00615	0.00725	0.01056	0.00443	-0.00954	-0.00972	-0.03844						
256	-0.07190	-0.04403	-0.03134	-0.03020	-0.02913	-0.04866	-0.06064							
128	-0.10360	-0.06229	-0.05139	-0.05633	-0.05872	-0.08935								
64	-0.12676	-0.09297	-0.07512	-0.09123	-0.10389									
32	-0.21012	-0.19978	-0.18407	-0.20502										
16	-0.28230	-0.26188	-0.26654											
8	-0.34826	-0.33998												
4	-0.45000													

(c) $\mathcal{P}^{\mathcal{C}}$ for Scene: [**Veach Bidir**] Algorithm: [**PSSMLT**] Metric: [**IFC**] True GT: [**BDPT @ 524288 spp**].

MLT	2	4	8	16	32	64	128	256	512	1024	2048	4096	8192	16384
GT (BDPT)														
262144	0.01069	0.01334	0.01622	0.00972	0.02443	0.01278	-0.00841	-0.00252	-0.00158	0.00482	0.00733	0.00692	0.00380	-0.00229
131072	-0.00356	0.02365	0.03898	0.02284	0.01601	0.01138	-0.00022	-0.00473	0.00068	-0.00168	0.00781	0.00267	0.00173	-0.00080
65536	0.01651	0.03157	0.03283	0.03516	0.02448	0.00610	-0.01367	-0.01539	-0.01153	-0.00058	0.00466	-0.00136	-0.00878	-0.01207
32768	-0.03445	-0.00734	0.01030	0.00753	0.01963	0.00930	-0.01148	-0.01224	-0.00440	-0.00857	-0.00104	-0.00654	-0.00706	-0.01822
16384	0.02697	0.01144	0.02824	0.03760	0.02799	0.01871	-0.00271	-0.00640	0.00102	-0.00208	-0.00231	-0.00314	-0.01252	
8192	-0.00249	-0.00558	0.00385	-0.00667	0.01026	-0.00483	-0.00423	-0.00931	-0.00562	-0.00657	-0.00265	-0.01015		
4096	0.01106	-0.00448	0.01263	0.01294	0.00573	-0.00692	-0.01840	-0.01558	-0.01153	-0.00834	-0.00732			
2048	0.01457	0.03733	0.03318	0.03893	0.01553	0.00164	-0.01568	-0.00933	-0.00751	-0.01118				
1024	0.00757	0.01658	0.02970	0.00366	0.00398	-0.00317	-0.01563	-0.00599	-0.00252					
512	-0.01482	-0.03097	0.00393	-0.01198	-0.00955	-0.01411	-0.03515	-0.03172						
256	-0.08542	-0.08333	-0.02897	-0.01240	-0.01844	-0.02872	-0.04454							
128	-0.10423	-0.09871	-0.09686	-0.07147	-0.06229	-0.05834								
64	-0.14700	-0.13407	-0.12351	-0.11033	-0.10269									
32	-0.25363	-0.22324	-0.18453	-0.17907										
16	-0.33609	-0.30566	-0.28169											
8	-0.39195	-0.37107												
4	-0.46518													

(d) $\mathcal{P}^{\mathcal{C}}$ for Scene: [**Veach Bidir**] Algorithm: [**MLT**] Metric: [**IFC**] True GT: [**BDPT @ 524288 spp**].

M-MLT	2	4	8	16	32	64	128	256	512	1024	2048	4096	8192	16384
GT (BDPT)														
262144	0.01072	0.02088	0.00650	0.00397	-0.00550	-0.00590	0.00094	0.00620	0.00546	0.00821	-0.00197	-0.00223	0.00351	0.00035
131072	0.01521	0.01108	-0.00624	0.01093	-0.00478	-0.00936	-0.00099	-0.00100	-0.00010	0.00503	0.00253	0.00002	-0.00312	-0.00526
65536	-0.00056	-0.00681	0.01471	0.00357	-0.00175	-0.00258	0.00446	0.00158	-0.00637	0.00220	-0.00439	-0.00538	-0.00634	-0.01255
32768	-0.00708	0.05100	0.02215	0.01458	-0.00361	0.01040	-0.00566	-0.00701	-0.01295	-0.00663	-0.01162	-0.01118	-0.01433	-0.02452
16384	0.02648	0.02454	0.00282	0.00809	-0.01283	-0.01053	0.00446	0.00532	-0.00198	0.00837	-0.00349	-0.00831	-0.02028	
8192	0.01178	0.01634	0.01479	0.02261	-0.01282	-0.00158	0.00972	0.00466	-0.00130	0.00210	-0.00796	-0.01855		
4096	0.01008	-0.00272	-0.00265	-0.00225	-0.02418	-0.02298	-0.00670	-0.00576	-0.01348	-0.00070	-0.01989			
2048	0.01576	-0.01740	-0.00220	0.02388	-0.01132	-0.00242	-0.01136	-0.01072	-0.01621	-0.01512				
1024	-0.03020	-0.01040	-0.01444	0.00832	-0.03506	-0.01649	-0.00028	0.00361	-0.00929					
512	-0.02532	-0.04018	-0.04845	-0.03563	-0.04681	-0.02173	-0.02464	-0.02413						
256	-0.06275	-0.03687	-0.04347	-0.02988	-0.06735	-0.05141	-0.03966							
128	-0.10878	-0.07678	-0.07660	-0.05082	-0.07951	-0.06510								
64	-0.15208	-0.11471	-0.12194	-0.08807	-0.11652									
32	-0.26054	-0.20310	-0.17439	-0.15322										
16	-0.31987	-0.26312	-0.26229											
8	-0.38495	-0.34861												
4	-0.45181													

(e) $\mathcal{P}^{\mathcal{C}}$ for Scene: [**Veach Bidir**] Algorithm: [**M-MLT**] Metric: [**IFC**] True GT: [**BDPT @ 524288 spp**].

ERPT	2	4	8	16	32	64	128	256	512	1024	2048	4096	8192	16384	32768	65536	131072
GT (BDPT)																	
262144	0.00328	0.00624	0.00218	-0.00033	-0.00702	0.00841	-0.00112	-0.00371	-0.00254	0.00059	0.00599	-0.00150	0.01685	-0.00087	-0.01104	0.01096	-0.00327
131072	0.00732	0.00549	0.00063	0.00317	-0.00161	0.00614	-0.00989	-0.00350	0.00462	-0.00480	0.00215	-0.00180	0.00319	-0.00804	-0.00393	0.00588	
65536	0.00601	0.00542	-0.02075	-0.01370	0.00603	-0.00717	-0.01004	0.00455	-0.00880	-0.01224	-0.01132	-0.00095	0.00599	0.01029	-0.00129		
32768	-0.00920	0.01643	-0.00675	-0.01438	-0.00103	0.00434	0.00873	0.01040	-0.00831	-0.02046	-0.00736	-0.00049	0.00768	-0.00350			
16384	-0.00729	0.00388	0.01502	-0.00774	-0.01192	0.00923	-0.00866	-0.00234	-0.00710	-0.00255	-0.00122	-0.00652	0.01023				
8192	0.00257	-0.00192	0.00364	-0.02074	-0.00998	0.00213	-0.01435	0.01596	-0.00682	-0.00998	0.00197	-0.01180					
4096	-0.01864	0.00598	-0.01360	-0.01790	-0.01587	-0.01389	-0.01610	-0.01091	-0.03051	-0.00752	-0.01473						
2048	-0.01412	-0.01273	-0.01215	-0.03025	-0.01536	-0.01150	-0.02619	-0.01275	-0.01962	-0.01090							
1024	-0.00999	0.00661	-0.00260	-0.01491	-0.00706	-0.01606	-0.00517	-0.00956	-0.01780								
512	-0.01197	0.00575	-0.00066	-0.02480	-0.01724	-0.02256	-0.02858	-0.01038									
256	-0.03017	-0.02884	-0.03787	-0.06652	-0.05476	-0.03794	-0.05401										
128	-0.08164	-0.06337	-0.08586	-0.07368	-0.07364	-0.07481											
64	-0.13262	-0.10725	-0.10848	-0.12833	-0.12434												
32	-0.22731	-0.20184	-0.22830	-0.24392													
16	-0.34788	-0.33511	-0.33872														
8	-0.47895	-0.45556															
4	-0.65145																

(f) $\mathcal{P}^{\mathcal{C}}$ for Scene: [**Veach Bidir**] Algorithm: [**ERPT**] Metric: [**IFC**] True GT: [**BDPT @ 524288 spp**].

M-ERPT	2	4	8	16	32	64	128	256	512	1024	2048	4096	8192	16384	32768	65536	131072
GT (BDPT)																	
262144	-0.01008	0.00752	0.00846	-0.00007	0.00362	-0.00050	0.00351	0.00106	-0.01018	-0.00243	0.00225	0.01436	0.00725	-0.00091	-0.00176	0.00245	0.00814
131072	0.00175	0.00172	0.00558	0.01027	0.00217	0.00962	0.00027	0.00251	-0.01307	-0.00252	-0.00416	0.00632	-0.00557	0.00694	0.01024	-0.00206	
65536	-0.00741	0.01213	0.00884	-0.01548	-0.01118	-0.00505	-0.01041	-0.00000	-0.01239	0.00194	-0.00695	0.00820	-0.00004	-0.00944	0.00182		
32768	-0.00142	-0.01218	-0.00357	0.00957	-0.00951	-0.00645	-0.01482	0.00208	-0.01609	-0.00466	-0.00363	-0.01184	-0.01268	-0.00606			
16384	-0.00860	0.00172	0.00541	-0.00846	0.01311	0.00583	0.00051	-0.01069	-0.01068	-0.00988	-0.01527	-0.00614	-0.02656				
8192	-0.00351	0.00896	0.00366	-0.00378	-0.01010	-0.00133	0.00215	-0.00568	-0.01577	0.00833	0.00160	-0.00013					
4096	-0.01733	-0.00554	-0.00760	-0.00037	-0.00021	-0.00955	-0.00895	-0.01810	-0.03062	-0.00892	-0.00515						
2048	-0.01080	-0.01172	-0.01297	-0.02248	-0.02476	-0.02710	-0.00972	-0.01724	-0.01965	-0.01570							
1024	-0.02324	-0.01293	-0.01225	-0.00023	-0.00398	-0.01097	-0.02200	-0.00224	-0.03009								
512	-0.02328	0.00395	-0.02079	-0.03754	-0.01852	-0.02763	-0.02794	-0.02906									
256	-0.03824	-0.03605	-0.03873	-0.06941	-0.04883	-0.06127	-0.05271										
128	-0.08539	-0.07502	-0.08309	-0.09297	-0.10013	-0.08634											
64	-0.11704	-0.12406	-0.14011	-0.14717	-0.15145												
32	-0.21427	-0.20015	-0.22658	-0.23789													
16	-0.33675	-0.33108	-0.35057														
8	-0.45759	-0.45865															
4	-0.63240																

(g) $\mathcal{P}^{\mathcal{C}}$ for Scene: [**Veach Bidir**] Algorithm: [**M-ERPT**] Metric: [**IFC**] True GT: [**BDPT @ 524288 spp**].

Table 98

21.4 Veach Door

PT	2	4	8	16	32	64	128	256	512	1024	2048	4096	8192	16384	32768	65536	131072
GT (BDPT)																	
262144	-0.00302	-0.00556	-0.02564	-0.01822	-0.00625	0.01116	0.00228	-0.00345	0.00317	0.00031	-0.00061	-0.00155	-0.00117	0.00365	0.00013	-0.00550	-0.00362
131072	0.01253	-0.03591	-0.01179	-0.02614	-0.02114	0.01509	-0.00621	-0.00915	0.00033	0.00408	-0.00130	-0.00189	0.00179	0.00370	-0.00336	-0.01036	
65536	-0.05344	-0.05235	-0.02321	-0.01561	-0.01955	0.01157	-0.00334	-0.00805	0.00026	0.00674	-0.00268	-0.00043	-0.00102	0.00071	-0.00514		
32768	-0.04672	-0.05238	-0.02646	-0.02216	-0.01047	0.01306	-0.00818	-0.01309	0.00057	0.00371	0.00155	-0.00032	-0.00078	-0.00191			
16384	-0.06060	-0.04218	-0.02409	-0.02087	-0.02130	0.01028	0.00021	-0.00355	-0.00146	0.00712	0.00967	0.00337	-0.00620				
8192	-0.04341	-0.05264	-0.05726	-0.01301	-0.01243	0.00675	0.00053	0.00686	0.00864	0.00915	0.00414	-0.00382					
4096	-0.05569	-0.04928	-0.05297	-0.05042	-0.01152	0.00738	0.00783	0.00679	0.01228	0.00851	0.00556						
2048	-0.06355	-0.05201	-0.02227	-0.04335	-0.01509	0.02367	0.01111	0.00803	0.00473	0.00035							
1024	-0.11004	-0.09056	-0.07119	-0.04477	-0.01753	0.01389	-0.00787	-0.01469	-0.01151								
512	-0.17245	-0.12987	-0.10173	-0.07532	-0.05084	-0.01123	-0.03229	-0.04505									
256	-0.18838	-0.16579	-0.13970	-0.09491	-0.07627	-0.05829	-0.07761										
128	-0.26405	-0.23928	-0.19351	-0.14843	-0.12923	-0.10990											
64	-0.29834	-0.27304	-0.22143	-0.20622	-0.19024												
32	-0.34706	-0.34521	-0.29852	-0.28770													
16	-0.40874	-0.37395	-0.40665														
8	-0.51601	-0.48958															
4	-0.58058																

(a) $\mathcal{P}^{\mathcal{C}}$ for Scene: [**Veach Door**] Algorithm: [**PT**] Metric: [**IFC**] True GT: [**BDPT @ 524288 spp**].

BDPT	2	4	8	16	32	64	128	256	512	1024	2048	4096	8192	16384	32768	65536	131072
GT (BDPT)																	
262144	-0.01399	0.01989	-0.00638	-0.00976	0.00063	-0.00184	0.00074	-0.00013	0.00346	-0.00069	-0.00160	-0.00161	-0.00049	-0.00150	-0.01023	-0.01416	-0.02427
131072	-0.05693	-0.00234	0.01638	-0.00531	0.00358	0.00192	-0.01459	-0.00771	-0.00218	-0.00199	-0.00088	-0.00118	-0.00471	-0.01301	-0.02675	-0.04363	
65536	-0.03236	0.00389	-0.01886	-0.01166	-0.00173	-0.00787	-0.00409	-0.00117	0.00056	0.00127	0.00148	-0.00465	-0.01339	-0.02956	-0.05916		
32768	-0.05189	-0.02208	0.00592	-0.02424	-0.00381	-0.00426	-0.00395	-0.00461	0.00107	0.00157	-0.00347	-0.01435	-0.03881	-0.06608			
16384	-0.02500	-0.01550	-0.01676	-0.01597	0.00163	-0.00236	-0.00252	-0.00245	0.00327	0.00466	-0.00887	-0.03117	-0.06706				
8192	-0.07158	-0.00097	-0.00470	-0.01227	0.01038	0.00213	0.00106	0.00428	0.00206	-0.00206	-0.02325	-0.07059					
4096	-0.05374	-0.01754	-0.02206	-0.02491	0.00242	0.00619	-0.00365	0.00916	-0.00509	-0.02447	-0.06353						
2048	-0.06592	-0.01451	-0.01368	-0.01903	0.00506	0.00606	-0.00395	-0.00708	-0.02354	-0.06168							
1024	-0.09300	-0.05320	-0.02397	-0.02462	-0.00648	-0.01044	-0.02103	-0.03789	-0.07320								
512	-0.13956	-0.06193	-0.06530	-0.05033	-0.02179	-0.03861	-0.07188	-0.10016									
256	-0.17453	-0.11026	-0.09120	-0.07505	-0.06795	-0.08990	-0.13740										
128	-0.23973	-0.13000	-0.14398	-0.13989	-0.13478	-0.16976											
64	-0.26911	-0.17613	-0.19442	-0.20396	-0.22377												
32	-0.32901	-0.24966	-0.25568	-0.32423													
16	-0.39557	-0.36920	-0.36067														
8	-0.50171	-0.44249															
4	-0.62489																

(b) $\mathcal{P}^{\mathcal{C}}$ for Scene: [**Veach Door**] Algorithm: [**BDPT**] Metric: [**IFC**] True GT: [**BDPT @ 524288 spp**].

PSSMLT	2	4	8	16	32	64	128	256	512	1024	2048	4096	8192	16384
GT (BDPT)														
262144	-0.01659	-0.01333	0.00032	-0.00018	0.00022	-0.00151	-0.00235	0.00153	-0.00469	0.00087	0.00110	-0.00036	-0.00348	-0.00948
131072	-0.01065	-0.00753	0.00555	0.00396	-0.01154	-0.01122	-0.01159	-0.00024	0.00055	-0.00092	-0.00164	-0.00551	-0.01432	-0.02972
65536	-0.01899	-0.01715	0.00254	0.00486	-0.00436	-0.00366	-0.00694	0.00076	0.00004	-0.00226	-0.00679	-0.01550	-0.03177	-0.05987
32768	-0.04242	-0.02790	-0.00699	-0.00465	-0.01192	-0.01179	-0.00905	-0.00220	-0.00518	-0.01088	-0.01744	-0.03788	-0.07260	-0.11680
16384	-0.02492	-0.01460	-0.00490	-0.00279	-0.01022	-0.00273	-0.00147	0.00352	-0.00539	-0.01368	-0.03304	-0.06736	-0.11884	
8192	-0.02591	-0.02204	0.00355	0.00622	0.00812	0.00683	0.00705	0.01149	-0.00283	-0.02623	-0.06532	-0.12293		
4096	-0.01831	-0.00882	0.01566	0.01664	0.01110	0.01564	0.01024	-0.00262	-0.02669	-0.07121	-0.12428			
2048	-0.03235	-0.01276	0.00326	0.00767	0.01351	0.00618	-0.00453	-0.02089	-0.06659	-0.12965				
1024	-0.03992	-0.03918	-0.00990	-0.00419	-0.00642	-0.01991	-0.04043	-0.08068	-0.14457					
512	-0.07611	-0.05915	-0.03738	-0.03282	-0.04540	-0.06754	-0.10222	-0.16405						
256	-0.11843	-0.08907	-0.06040	-0.06977	-0.10187	-0.13953	-0.20005							
128	-0.18118	-0.15024	-0.11913	-0.13383	-0.17798	-0.23892								
64	-0.25096	-0.21243	-0.20601	-0.23350	-0.30230									
32	-0.28989	-0.30293	-0.32744	-0.37293										
16	-0.42614	-0.41443	-0.46324											
8	-0.54673	-0.58697												
4	-0.76071													

(c) $\mathcal{P}^{\mathcal{C}}$ for Scene: [**Veach Door**] Algorithm: [**PSSMLT**] Metric: [**IFC**] True GT: [**BDPT @ 524288 spp**].

MLT	2	4	8	16	32	64	128	256	512	1024	2048	4096	8192	16384
GT (BDPT)														
262144	-0.00542	-0.00109	-0.00071	0.00804	0.00475	0.00204	0.00053	-0.00102	0.00276	0.00256	-0.00010	-0.00292	-0.00849	-0.01030
131072	-0.00826	-0.00905	-0.00905	0.00268	0.00096	-0.00425	0.00183	-0.00059	0.00161	0.00153	-0.00232	-0.00626	-0.02216	-0.03617
65536	-0.02415	-0.01269	-0.00833	0.00399	-0.00046	-0.00333	-0.00177	-0.00338	0.00153	0.00028	-0.00590	-0.02246	-0.04172	-0.07112
32768	-0.03212	-0.01727	-0.01799	0.00750	-0.00118	0.00319	0.00117	-0.00088	0.00403	-0.00254	-0.01622	-0.04916	-0.08926	-0.13207
16384	-0.03518	-0.01655	-0.00270	-0.00090	0.00243	0.00474	-0.00118	0.00114	-0.00231	-0.02002	-0.04562	-0.08835	-0.14842	
8192	-0.04005	0.00361	-0.00032	0.01092	0.01168	0.01005	0.00017	0.00236	-0.01318	-0.04578	-0.08900	-0.15959		
4096	-0.04001	-0.00996	0.00267	0.01494	0.01789	0.01626	0.00353	-0.01500	-0.03977	-0.08777	-0.16120			
2048	-0.05900	-0.01194	-0.00309	0.01206	0.01650	0.00434	-0.00376	-0.03650	-0.08695	-0.16063				
1024	-0.06862	-0.02589	-0.01576	0.00650	-0.00618	-0.03029	-0.04632	-0.09658	-0.16869					
512	-0.09337	-0.05218	-0.03314	-0.02161	-0.03969	-0.07238	-0.12290	-0.19289						
256	-0.13221	-0.09698	-0.08243	-0.06918	-0.09708	-0.14905	-0.21059							
128	-0.16143	-0.14192	-0.13464	-0.14082	-0.19175	-0.27055								
64	-0.25788	-0.21891	-0.22190	-0.24567	-0.32344									
32	-0.30065	-0.31107	-0.33007	-0.39228										
16	-0.39461	-0.41839	-0.46514											
8	-0.54439	-0.59804												
4	-0.74738													

(d) $\mathcal{P}^{\mathcal{C}}$ for Scene: [**Veach Door**] Algorithm: [**MLT**] Metric: [**IFC**] True GT: [**BDPT @ 524288 spp**].

M-MLT	2	4	8	16	32	64	128	256	512	1024	2048	4096	8192	16384
GT (BDPT)														
262144	-0.02566	0.01432	-0.01101	-0.01364	-0.00405	0.00003	-0.00330	0.00382	-0.00496	0.00032	-0.00206	-0.00322	-0.00484	-0.01126
131072	-0.03446	-0.00402	-0.00557	-0.00907	-0.00734	-0.00613	-0.00425	0.00012	-0.00482	-0.00176	-0.00780	-0.01025	-0.01966	-0.03028
65536	-0.04135	-0.00463	-0.00665	-0.00100	-0.00849	-0.00180	0.00008	0.00161	-0.00127	-0.00576	-0.01201	-0.02344	-0.04416	-0.07323
32768	-0.05557	-0.01914	-0.00945	-0.01038	-0.00692	-0.00255	-0.00266	0.00085	-0.00395	-0.01095	-0.02704	-0.05194	-0.09087	-0.13569
16384	-0.05462	-0.00411	-0.01725	-0.00358	-0.00530	0.00791	0.00700	0.00595	-0.00217	-0.02305	-0.05025	-0.09058	-0.15018	
8192	-0.03986	-0.01890	-0.00616	-0.00840	-0.00612	0.00838	0.00822	0.00355	-0.01830	-0.04571	-0.09588	-0.16166		
4096	-0.04350	-0.00242	0.00471	0.00294	0.00199	0.01016	-0.00178	-0.01143	-0.04516	-0.09334	-0.16732			
2048	-0.05383	-0.01007	-0.01473	0.01036	-0.00221	0.00473	-0.01762	-0.04341	-0.09595	-0.16760				
1024	-0.07361	-0.03256	-0.01952	-0.00191	-0.01913	-0.02169	-0.04960	-0.09843	-0.18151					
512	-0.09563	-0.05583	-0.05067	-0.03127	-0.06256	-0.07230	-0.12895	-0.19453						
256	-0.12732	-0.08267	-0.08512	-0.08255	-0.12062	-0.15437	-0.22233							
128	-0.18832	-0.13779	-0.14685	-0.16419	-0.21471	-0.27047								
64	-0.23772	-0.19520	-0.21202	-0.25585	-0.34039									
32	-0.31852	-0.29746	-0.34353	-0.40093										
16	-0.40710	-0.39322	-0.48016											
8	-0.54687	-0.57035												
4	-0.75365													

(e) $\mathcal{P}^{\mathcal{C}}$ for Scene: [**Veach Door**] Algorithm: [**M-MLT**] Metric: [**IFC**] True GT: [**BDPT @ 524288 spp**].

ERPT	2	4	8	16	32	64	128	256	512	1024	2048	4096	8192	16384	32768	65536	131072
GT (BDPT)																	
262144	-0.00187	0.00567	0.00221	-0.00093	-0.00414	0.00103	-0.00129	0.00097	-0.00202	0.00439	0.00090	0.00467	0.00256	-0.00281	0.00498	0.00377	-0.00152
131072	-0.00080	0.00090	0.00004	-0.00215	-0.00597	-0.00610	-0.00310	0.00247	-0.00584	-0.00344	0.00297	-0.00153	-0.00192	-0.00326	-0.00045	0.00072	
65536	-0.00595	0.00056	-0.00096	-0.00105	-0.00649	-0.00626	-0.00361	-0.00255	-0.00520	-0.00268	0.00054	-0.00026	-0.00257	-0.00193	-0.00079		
32768	-0.00479	0.00103	0.00147	-0.00349	-0.00392	-0.00318	-0.00542	0.00549	-0.00500	-0.00219	0.00353	0.00158	0.00057	0.00257			
16384	0.00373	0.00386	0.00569	-0.00324	0.00130	0.00191	0.00384	0.00903	0.00038	0.00102	0.00811	0.00836	0.00214				
8192	0.01060	0.01141	0.00721	0.00441	-0.00189	0.00131	0.00378	0.00663	0.00469	-0.00077	0.00748	0.01528					
4096	-0.00221	0.00029	0.00077	-0.00420	0.00102	-0.00606	-0.00201	-0.00040	-0.00607	0.00453	0.00296						
2048	-0.01726	-0.01743	-0.02186	-0.02355	-0.01826	-0.02038	-0.02002	-0.02248	-0.02443	-0.01770							
1024	-0.06631	-0.06561	-0.06652	-0.06733	-0.06629	-0.06526	-0.06817	-0.06294	-0.07350								
512	-0.14722	-0.14322	-0.14895	-0.14272	-0.14722	-0.15270	-0.14459	-0.14837									
256	-0.25449	-0.24879	-0.24959	-0.24857	-0.25462	-0.25721	-0.25794										
128	-0.39656	-0.39440	-0.40101	-0.40740	-0.41163	-0.40536											
64	-0.56888	-0.55784	-0.57077	-0.56994	-0.56933												
32	-0.76242	-0.76497	-0.77549	-0.78503													
16	-1.00398	-1.01438	-1.00989														
8	-1.29804	-1.29998															
4	-1.62164																

(f) $\mathcal{P}^{\mathcal{C}}$ for Scene: [**Veach Door**] Algorithm: [**ERPT**] Metric: [**IFC**] True GT: [**BDPT @ 524288 spp**].

M-ERPT	2	4	8	16	32	64	128	256	512	1024	2048	4096	8192	16384	32768	65536	131072
GT (BDPT)																	
262144	-0.00128	-0.00268	0.00310	0.00364	-0.00244	0.00361	0.00008	0.00289	-0.00195	-0.00021	0.00745	-0.00089	-0.00341	0.00445	-0.00552	-0.00297	-0.00441
131072	-0.00280	-0.00582	-0.00099	-0.00497	0.00622	0.00199	0.00069	0.00041	-0.00468	-0.00478	0.00758	-0.00326	-0.00489	-0.00253	-0.00352	-0.00377	
65536	-0.00630	-0.00623	-0.00324	-0.00114	-0.00518	0.00442	0.00306	0.00227	0.00243	-0.00481	0.00855	0.00013	-0.00862	-0.00234	-0.00406		
32768	0.00044	-0.00517	0.00422	-0.00117	-0.00260	-0.00150	0.00544	0.00507	0.00145	0.00110	0.00743	0.00048	-0.00648	0.00316			
16384	0.00811	-0.00334	0.00735	0.00045	0.00188	0.00743	0.00985	0.00063	0.00490	0.00306	0.01030	0.00219	0.00388				
8192	0.00842	0.00456	0.01333	0.00815	0.00385	0.01128	0.00858	0.00933	0.00094	-0.00182	0.01303	0.00200					
4096	0.00342	-0.00310	0.00559	-0.00204	0.00100	0.00232	0.00715	0.00058	-0.00421	-0.00120	0.00873						
2048	-0.01414	-0.02125	-0.02011	-0.01453	-0.01381	-0.01816	-0.01670	-0.02047	-0.01787	-0.02420							
1024	-0.05114	-0.05849	-0.05666	-0.05989	-0.05886	-0.05849	-0.05600	-0.06699	-0.05692								
512	-0.12298	-0.13141	-0.12632	-0.14305	-0.13436	-0.13370	-0.13574	-0.13947									
256	-0.23161	-0.23790	-0.23224	-0.24012	-0.23493	-0.23565	-0.23785										
128	-0.36389	-0.36865	-0.37097	-0.38364	-0.38917	-0.38331											
64	-0.52545	-0.54193	-0.54111	-0.56043	-0.55416												
32	-0.73333	-0.75204	-0.73578	-0.76303													
16	-0.95636	-0.97295	-0.98084														
8	-1.25284	-1.28220															
4	-1.57825																

(g) $\mathcal{P}^{\mathcal{C}}$ for Scene: [**Veach Door**] Algorithm: [**M-ERPT**] Metric: [**IFC**] True GT: [**BDPT @ 524288 spp**].

Table 99

21.5 Sponza

PT	2	4	8	16	32	64	128	256	512	1024	2048	4096	8192
GT (PT)													
16384	0.00096	-0.00394	0.00080	-0.00404	0.00142	0.00061	0.00430	-0.00346	-0.00585	-0.00093	-0.01241	-0.02874	-0.03245
8192	0.01862	0.00749	0.00862	0.00819	-0.00270	-0.00195	0.00505	-0.00181	-0.01627	-0.01588	-0.03641	-0.07454	
4096	0.00637	0.00500	0.00368	-0.00015	-0.00405	-0.00173	-0.00159	-0.01661	-0.02499	-0.04680	-0.08352		
2048	0.00076	-0.00056	0.00337	0.00258	-0.00663	-0.00676	-0.01172	-0.03004	-0.06180	-0.09532			
1024	0.00275	-0.01008	-0.01350	-0.01346	-0.02578	-0.03103	-0.04680	-0.07403	-0.12881				
512	-0.03979	-0.03955	-0.03182	-0.04706	-0.05460	-0.06731	-0.09711	-0.14167					
256	-0.04818	-0.05284	-0.07603	-0.08512	-0.09148	-0.14043	-0.14916						
128	-0.11942	-0.10569	-0.11018	-0.13172	-0.16900	-0.24945							
64	-0.13820	-0.15169	-0.18765	-0.22686	-0.29342								
32	-0.17079	-0.20264	-0.25229	-0.31290									
16	-0.24364	-0.28575	-0.32614										
8	-0.33147	-0.38626											
4	-0.41969												

(a) $\mathcal{P}^{\mathcal{C}}$ for Scene: [**Sponza**] Algorithm: [**PT**] Metric: [**IFC**] True GT: [**PT @ 32768 spp**].

BDPT	2	4	8	16	32	64	128	256	512	1024	2048	4096	8192
GT (PT)													
16384	0.00812	-0.00135	0.00170	0.00290	-0.00034	0.00215	0.00108	-0.00111	0.00072	-0.00724	-0.00765	-0.01741	-0.03203
8192	0.01035	0.00472	-0.00004	0.00697	0.00005	0.00034	0.00133	-0.00120	-0.00518	-0.01566	-0.02968	-0.05717	
4096	0.02180	0.01676	-0.00034	0.00427	-0.00404	-0.00105	-0.00287	-0.00447	-0.01597	-0.04139	-0.07631		
2048	0.00439	0.00805	-0.00487	0.00438	-0.00601	-0.00393	-0.01389	-0.02526	-0.04753	-0.09133			
1024	0.00608	0.00029	-0.02662	-0.02041	-0.02369	-0.02829	-0.03753	-0.06436	-0.10853				
512	-0.01064	-0.02591	-0.03543	-0.05197	-0.04852	-0.06493	-0.08668	-0.13692					
256	-0.03909	-0.05892	-0.07028	-0.07197	-0.09465	-0.11830	-0.17044						
128	-0.08414	-0.10505	-0.13001	-0.13823	-0.17269	-0.22688							
64	-0.11307	-0.13636	-0.17623	-0.21145	-0.26860								
32	-0.16851	-0.21804	-0.24470	-0.30047									
16	-0.24548	-0.26048	-0.34374										
8	-0.28009	-0.38897											
4	-0.38813												

(b) $\mathcal{P}^{\mathcal{C}}$ for Scene: [**Sponza**] Algorithm: [**BDPT**] Metric: [**IFC**] True GT: [**PT @ 32768 spp**].

PSSMLT	2	4	8	16	32	64	128	256	512	1024	2048	4096	8192
GT (PT)													
16384	0.00186	-0.00223	0.00302	0.00149	0.00071	0.00452	0.00033	0.00061	-0.00057	-0.00175	-0.00451	-0.01159	-0.02102
8192	0.00955	0.00481	0.00175	0.00222	0.00229	0.01036	0.00434	0.00031	-0.00071	-0.00444	-0.01825	-0.03455	
4096	0.00201	-0.00036	0.00146	0.00159	0.00006	0.00337	-0.00178	-0.00605	-0.00975	-0.02447	-0.04370		
2048	0.00069	-0.00397	-0.00089	-0.00807	-0.00355	-0.00300	-0.01217	-0.01811	-0.03331	-0.05464			
1024	-0.00206	-0.00857	-0.01391	-0.01823	-0.01240	-0.04107	-0.03838	-0.05410	-0.08350				
512	-0.04489	-0.05264	-0.04913	-0.04313	-0.05536	-0.05801	-0.08925	-0.11962					
256	-0.07001	-0.08410	-0.07910	-0.09423	-0.10629	-0.14214	-0.16282						
128	-0.13608	-0.14115	-0.13466	-0.16464	-0.19103	-0.24359							
64	-0.18722	-0.21341	-0.22621	-0.25938	-0.29024								
32	-0.24704	-0.27631	-0.28914	-0.34045									
16	-0.35796	-0.39867	-0.45872										
8	-0.42419	-0.48949											
4	-0.54644												

(c) $\mathcal{P}^{\mathcal{C}}$ for Scene: [**Sponza**] Algorithm: [**PSSMLT**] Metric: [**IFC**] True GT: [**PT @ 32768 spp**].

MLT	2	4	8	16	32	64	128	256	512	1024	2048	4096	8192
GT (PT)													
16384	0.00182	-0.00508	0.00587	0.00044	0.00418	0.00645	0.00497	0.00224	0.00063	-0.00665	-0.01452	-0.02716	-0.03587
8192	0.01377	0.00688	0.00524	0.00154	0.00944	0.00995	0.00136	-0.00139	-0.00886	-0.02671	-0.04691	-0.07071	
4096	0.00947	0.00758	-0.00118	-0.00025	0.00487	0.00598	-0.00314	-0.01249	-0.03161	-0.06227	-0.09366		
2048	0.00695	-0.00240	-0.00333	-0.00511	-0.00755	-0.00306	-0.01966	-0.03453	-0.06688	-0.11480			
1024	-0.01657	-0.02149	-0.02110	-0.03352	-0.02883	-0.04217	-0.06616	-0.08781	-0.10703				
512	-0.03789	-0.05520	-0.04912	-0.05557	-0.07439	-0.08142	-0.12673	-0.17427					
256	-0.07783	-0.10460	-0.09573	-0.14185	-0.14044	-0.17201	-0.23049						
128	-0.12794	-0.15405	-0.16514	-0.20085	-0.23291	-0.29567							
64	-0.17875	-0.21482	-0.24528	-0.30270	-0.34535								
32	-0.23869	-0.28142	-0.34373	-0.41055									
16	-0.31108	-0.40793	-0.48066										
8	-0.39809	-0.53790											
4	-0.53754												

(d) $\mathcal{P}^{\mathcal{C}}$ for Scene: [**Sponza**] Algorithm: [**MLT**] Metric: [**IFC**] True GT: [**PT @ 32768 spp**].

M-MLT	2	4	8	16	32	64	128	256	512	1024	2048	4096	8192
GT (PT)													
16384	-0.00959	-0.00175	0.00173	0.00294	0.00224	0.00541	0.00175	0.00001	-0.00069	-0.00540	-0.01453	-0.02503	-0.03829
8192	0.00799	0.00392	0.00426	0.00698	0.00131	0.00638	0.00023	0.00018	-0.01507	-0.02434	-0.04472	-0.07036	
4096	-0.00112	0.00729	0.00884	0.00968	0.00091	-0.00009	-0.00309	-0.01777	-0.03290	-0.05283	-0.09648		
2048	-0.00393	0.00571	-0.00994	-0.00905	-0.00697	-0.00539	-0.02045	-0.03780	-0.07469	-0.12264			
1024	-0.01895	-0.01798	-0.02470	-0.01540	-0.02600	-0.04017	-0.06134	-0.07843	-0.15317				
512	-0.04379	-0.04157	-0.05306	-0.06080	-0.07596	-0.09901	-0.12925	-0.18444					
256	-0.10020	-0.08501	-0.10003	-0.11055	-0.13201	-0.18981	-0.22754						
128	-0.13104	-0.14787	-0.16613	-0.19287	-0.23409	-0.31630							
64	-0.20777	-0.23558	-0.25414	-0.29802	-0.36619								
32	-0.24952	-0.28620	-0.33951	-0.42347									
16	-0.34472	-0.40976	-0.49923										
8	-0.45426	-0.53355											
4	-0.59603												

(e) $\mathcal{P}^{\mathcal{C}}$ for Scene: [**Sponza**] Algorithm: [**M-MLT**] Metric: [**IFC**] True GT: [**PT @ 32768 spp**].

ERPT	2	4	8	16	32	64	128	256	512	1024	2048	4096	8192
GT (PT)													
16384	-0.00383	0.00036	0.00294	0.00383	-0.00054	0.00058	-0.00096	0.00116	0.00093	-0.00167	0.00054	-0.00063	0.00116
8192	-0.00115	-0.00420	-0.00192	0.00109	-0.00074	0.00155	-0.00296	0.00061	-0.00148	-0.00164	0.00001	-0.00302	
4096	-0.01056	-0.00857	-0.00623	-0.00616	-0.00942	-0.00909	-0.00990	-0.00317	-0.00864	-0.00816	-0.00392		
2048	-0.02261	-0.02956	-0.02747	-0.02301	-0.02682	-0.03007	-0.02882	-0.02838	-0.03294	-0.02865			
1024	-0.07077	-0.08038	-0.08011	-0.07152	-0.07704	-0.07987	-0.08294	-0.06956	-0.08057				
512	-0.13731	-0.14352	-0.13995	-0.14182	-0.14837	-0.15353	-0.15079	-0.14033					
256	-0.24407	-0.26981	-0.26046	-0.25429	-0.25527	-0.26280	-0.26202						
128	-0.39972	-0.41820	-0.40841	-0.41426	-0.41060	-0.41630							
64	-0.57045	-0.58825	-0.60073	-0.59560	-0.58395								
32	-0.75678	-0.77704	-0.77308	-0.77481									
16	-0.99424	-1.03306	-1.02067										
8	-1.24345	-1.26756											
4	-1.50391												

(f) $\mathcal{P}^{\mathcal{C}}$ for Scene: [**Sponza**] Algorithm: [**ERPT**] Metric: [**IFC**] True GT: [**PT @ 32768 spp**].

M-ERPT	2	4	8	16	32	64	128	256	512	1024	2048	4096	8192
GT (PT)													
16384	0.00389	-0.00446	0.00050	-0.00008	0.00014	0.00374	0.00071	0.00374	0.00249	0.00271	-0.00005	0.00171	0.00518
8192	0.00001	-0.00442	-0.00092	0.00117	0.00003	-0.00213	-0.00020	0.00298	0.00169	0.00102	-0.00350	0.00216	
4096	-0.00591	-0.00906	-0.01462	-0.00757	-0.00624	-0.00776	-0.00950	-0.00314	-0.00105	-0.00811	-0.00581		
2048	-0.02548	-0.03315	-0.02423	-0.02356	-0.02984	-0.02604	-0.02161	-0.02321	-0.02880	-0.02112			
1024	-0.07572	-0.07598	-0.07353	-0.06995	-0.07126	-0.07157	-0.07918	-0.07522	-0.07036				
512	-0.13422	-0.14697	-0.14469	-0.13795	-0.14870	-0.14017	-0.14215	-0.14144					
256	-0.23612	-0.25890	-0.25149	-0.24908	-0.25754	-0.24772	-0.24456						
128	-0.38374	-0.41980	-0.40257	-0.39146	-0.40587	-0.40106							
64	-0.57442	-0.57882	-0.57416	-0.57759	-0.57677								
32	-0.73190	-0.78232	-0.75229	-0.77628									
16	-0.98515	-1.01234	-1.01007										
8	-1.22050	-1.26266											
4	-1.49581												

(g) $\mathcal{P}^{\mathcal{C}}$ for Scene: [**Sponza**] Algorithm: [**M-ERPT**] Metric: [**IFC**] True GT: [**PT @ 32768 spp**].

Table 100

22 VIF

22.1 Cornell Box

PT	2	4	8	16	32	64	128	256	512	1024	2048	4096	8192
GT (PT)													
16384	-0.00210	-0.00211	-0.00234	-0.00180	-0.00268	-0.00317	-0.00323	-0.00325	-0.00436	-0.00503	-0.00563	-0.00563	-0.00630
8192	-0.00664	-0.00654	-0.00603	-0.00682	-0.00663	-0.00768	-0.00925	-0.01041	-0.01168	-0.01349	-0.01522	-0.01611	
4096	-0.01439	-0.01451	-0.01453	-0.01564	-0.01717	-0.01816	-0.02171	-0.02506	-0.02807	-0.03109	-0.03370		
2048	-0.02864	-0.02741	-0.02956	-0.03075	-0.03339	-0.03802	-0.04303	-0.04959	-0.05573	-0.06156			
1024	-0.05344	-0.05387	-0.05683	-0.06040	-0.06638	-0.07438	-0.08257	-0.09298	-0.10477				
512	-0.09602	-0.09862	-0.10344	-0.11175	-0.12112	-0.13402	-0.14958	-0.16574					
256	-0.16241	-0.16825	-0.17503	-0.18892	-0.20440	-0.22874	-0.25735						
128	-0.25969	-0.27053	-0.28636	-0.30675	-0.33370	-0.37004							
64	-0.38558	-0.39907	-0.42676	-0.45660	-0.50209								
32	-0.54037	-0.56534	-0.60151	-0.65487									
16	-0.72288	-0.76788	-0.81943										
8	-0.93295	-0.99426											
4	-1.15257												

(a) $\mathcal{P}^{\mathcal{C}}$ for Scene: [**Cornell Box**] Algorithm: [**PT**] Metric: [**VIF**] True GT: [**PT @ 32768 spp**].

BDPT	2	4	8	16	32	64	128	256	512	1024	2048	4096	8192
GT (PT)													
16384	-0.00220	-0.00245	-0.00226	-0.00297	-0.00345	-0.00391	-0.00436	-0.00465	-0.00547	-0.00540	-0.00597	-0.00623	-0.00681
8192	-0.00615	-0.00624	-0.00723	-0.00845	-0.00899	-0.01050	-0.01224	-0.01361	-0.01478	-0.01565	-0.01678	-0.01760	
4096	-0.01403	-0.01611	-0.01684	-0.01841	-0.02120	-0.02472	-0.02762	-0.03124	-0.03331	-0.03580	-0.03766		
2048	-0.02817	-0.02970	-0.03269	-0.03682	-0.04304	-0.04905	-0.05489	-0.06046	-0.06549	-0.06902			
1024	-0.05707	-0.06009	-0.06457	-0.07179	-0.08089	-0.09143	-0.10328	-0.11357	-0.12051				
512	-0.10132	-0.10886	-0.11870	-0.13149	-0.14697	-0.16575	-0.18370	-0.19860					
256	-0.17229	-0.18211	-0.19971	-0.22460	-0.25048	-0.27817	-0.30856						
128	-0.27769	-0.29942	-0.32306	-0.35713	-0.40423	-0.44500							
64	-0.40747	-0.44486	-0.49189	-0.53931	-0.59707								
32	-0.58385	-0.63379	-0.69616	-0.76560									
16	-0.79189	-0.86063	-0.94063										
8	-1.02780	-1.11807											
4	-1.28597												

(b) $\mathcal{P}^{\mathcal{C}}$ for Scene: [**Cornell Box**] Algorithm: [**BDPT**] Metric: [**VIF**] True GT: [**PT @ 32768 spp**].

PSSMLT	2	4	8	16	32	64	128	256	512	1024	2048	4096	8192
GT (PT)													
16384	-0.00318	-0.00320	-0.00266	-0.00259	-0.00331	-0.00420	-0.00443	-0.00463	-0.00503	-0.00542	-0.00572	-0.00601	-0.00586
8192	-0.00684	-0.00710	-0.00665	-0.00734	-0.00882	-0.01050	-0.01141	-0.01294	-0.01390	-0.01475	-0.01559	-0.01612	
4096	-0.01584	-0.01628	-0.01733	-0.01873	-0.02123	-0.02409	-0.02715	-0.02960	-0.03214	-0.03410	-0.03556		
2048	-0.03107	-0.03190	-0.03480	-0.03774	-0.04313	-0.04769	-0.05317	-0.05877	-0.06315	-0.06666			
1024	-0.06031	-0.06301	-0.06682	-0.07270	-0.08038	-0.09096	-0.10035	-0.10945	-0.11641				
512	-0.10840	-0.11221	-0.11993	-0.13167	-0.14665	-0.16374	-0.17980	-0.19376					
256	-0.18127	-0.19277	-0.20684	-0.22648	-0.25181	-0.27675	-0.30049						
128	-0.29181	-0.30979	-0.33236	-0.36449	-0.40178	-0.43706							
64	-0.43112	-0.45996	-0.49726	-0.54578	-0.59698								
32	-0.60775	-0.65448	-0.71028	-0.77375									
16	-0.81850	-0.88177	-0.95940										
8	-1.05666	-1.14478											
4	-1.32579												

(c) $\mathcal{P}^{\mathcal{C}}$ for Scene: [**Cornell Box**] Algorithm: [**PSSMLT**] Metric: [**VIF**] True GT: [**PT @ 32768 spp**].

MLT	2	4	8	16	32	64	128	256	512	1024	2048	4096	8192
GT (PT)													
16384	-0.00295	-0.00237	-0.00199	-0.00222	-0.00224	-0.00273	-0.00299	-0.00383	-0.00418	-0.00445	-0.00470	-0.00531	-0.00552
8192	-0.00644	-0.00626	-0.00613	-0.00652	-0.00667	-0.00742	-0.00889	-0.01052	-0.01162	-0.01298	-0.01400	-0.01495	
4096	-0.01456	-0.01440	-0.01451	-0.01584	-0.01739	-0.01921	-0.02163	-0.02449	-0.02778	-0.02965	-0.03221		
2048	-0.02971	-0.02817	-0.02797	-0.02917	-0.03272	-0.03660	-0.04126	-0.04740	-0.05300	-0.05750			
1024	-0.05800	-0.05739	-0.05775	-0.06178	-0.06609	-0.07229	-0.08035	-0.08945	-0.09913				
512	-0.10344	-0.10284	-0.10488	-0.11173	-0.11968	-0.13038	-0.14387	-0.16035					
256	-0.17301	-0.17576	-0.18044	-0.18916	-0.20245	-0.22082	-0.24326						
128	-0.27234	-0.27270	-0.28053	-0.29748	-0.31814	-0.34816							
64	-0.40074	-0.40767	-0.42508	-0.44833	-0.47717								
32	-0.55654	-0.56544	-0.58730	-0.62911									
16	-0.73546	-0.75461	-0.79091										
8	-0.92823	-0.96676											
4	-1.16013												

(d) $\mathcal{P}^{\mathcal{C}}$ for Scene: [**Cornell Box**] Algorithm: [**MLT**] Metric: [**VIF**] True GT: [**PT @ 32768 spp**].

M-MLT	2	4	8	16	32	64	128	256	512	1024	2048	4096	8192
GT (PT)													
16384	-0.00234	-0.00204	-0.00187	-0.00226	-0.00192	-0.00282	-0.00349	-0.00403	-0.00400	-0.00498	-0.00508	-0.00535	-0.00575
8192	-0.00705	-0.00634	-0.00653	-0.00706	-0.00728	-0.00854	-0.00918	-0.01081	-0.01255	-0.01353	-0.01466	-0.01496	
4096	-0.01588	-0.01463	-0.01553	-0.01652	-0.01733	-0.01969	-0.02199	-0.02474	-0.02753	-0.03031	-0.03270		
2048	-0.03005	-0.03012	-0.03057	-0.03217	-0.03432	-0.03639	-0.04131	-0.04719	-0.05258	-0.05836			
1024	-0.05857	-0.05760	-0.06028	-0.06391	-0.06853	-0.07402	-0.08204	-0.09215	-0.10030				
512	-0.09981	-0.10302	-0.10712	-0.11360	-0.11946	-0.12987	-0.14392	-0.16065					
256	-0.17307	-0.17561	-0.18111	-0.19069	-0.20321	-0.22104	-0.24577						
128	-0.27277	-0.27573	-0.28713	-0.30446	-0.32581	-0.35539							
64	-0.38898	-0.40764	-0.42251	-0.44865	-0.47975								
32	-0.55841	-0.57773	-0.59923	-0.63869									
16	-0.74054	-0.76390	-0.79934										
8	-0.95538	-0.97788											
4	-1.16449												

(e) $\mathcal{P}^{\mathcal{C}}$ for Scene: [**Cornell Box**] Algorithm: [**M-MLT**] Metric: [**VIF**] True GT: [**PT @ 32768 spp**].

ERPT	2	4	8	16	32	64	128	256	512	1024	2048	4096	8192
GT (PT)													
16384	-0.00297	-0.00396	-0.00324	-0.00374	-0.00283	-0.00355	-0.00362	-0.00367	-0.00394	-0.00305	-0.00277	-0.00334	-0.00384
8192	-0.00981	-0.00940	-0.00996	-0.00930	-0.00930	-0.00961	-0.00905	-0.01022	-0.00948	-0.00933	-0.00941	-0.00960	
4096	-0.02142	-0.02268	-0.02180	-0.02136	-0.02175	-0.02251	-0.02210	-0.02207	-0.02162	-0.02197	-0.02270		
2048	-0.04305	-0.04329	-0.04365	-0.04293	-0.04269	-0.04356	-0.04293	-0.04265	-0.04163	-0.04335			
1024	-0.08267	-0.08091	-0.08095	-0.08117	-0.08068	-0.08336	-0.08280	-0.08230	-0.08290				
512	-0.14811	-0.14690	-0.14716	-0.14668	-0.14764	-0.14867	-0.14630	-0.14813					
256	-0.24866	-0.25125	-0.24929	-0.24816	-0.24754	-0.25002	-0.24798						
128	-0.39364	-0.39316	-0.39618	-0.39537	-0.39472	-0.39598							
64	-0.58397	-0.58498	-0.58383	-0.58458	-0.58058								
32	-0.82115	-0.82365	-0.82717	-0.82476									
16	-1.09517	-1.09903	-1.10565										
8	-1.40547	-1.40968											
4	-1.72872												

(f) $\mathcal{P}^{\mathcal{C}}$ for Scene: [**Cornell Box**] Algorithm: [**ERPT**] Metric: [**VIF**] True GT: [**PT @ 32768 spp**].

M-ERPT	2	4	8	16	32	64	128	256	512	1024	2048	4096	8192
GT (PT)													
16384	-0.00327	-0.00346	-0.00358	-0.00344	-0.00326	-0.00401	-0.00346	-0.00353	-0.00379	-0.00310	-0.00300	-0.00372	-0.00336
8192	-0.00964	-0.00956	-0.00946	-0.01030	-0.00914	-0.00928	-0.00877	-0.00937	-0.00919	-0.00930	-0.00937	-0.00937	
4096	-0.02121	-0.02112	-0.02164	-0.02185	-0.02167	-0.02176	-0.02112	-0.02093	-0.02176	-0.02187	-0.02180		
2048	-0.04268	-0.04289	-0.04297	-0.04277	-0.04312	-0.04346	-0.04159	-0.04122	-0.04219	-0.04228			
1024	-0.08068	-0.08047	-0.08201	-0.08133	-0.07986	-0.08134	-0.07999	-0.08186	-0.08107				
512	-0.14465	-0.14550	-0.14628	-0.14607	-0.14536	-0.14496	-0.14726	-0.14372					
256	-0.24563	-0.24505	-0.24758	-0.24392	-0.24536	-0.24681	-0.24798						
128	-0.39011	-0.38972	-0.39293	-0.39254	-0.39030	-0.38965							
64	-0.58634	-0.58127	-0.58054	-0.57887	-0.57797								
32	-0.82065	-0.81736	-0.81817	-0.81846									
16	-1.08898	-1.09415	-1.09238										
8	-1.39938	-1.39888											
4	-1.72764												

(g) $\mathcal{P}^{\mathcal{C}}$ for Scene: [**Cornell Box**] Algorithm: [**M-ERPT**] Metric: [**VIF**] True GT: [**PT @ 32768 spp**].

Table 101

22.2 Torus

PT	2	4	8	16	32	64	128	256	512	1024	2048	4096	8192	16384	32768	65536	131072
GT (BDPT)																	
262144	-0.01219	-0.01055	-0.01542	-0.01601	-0.01542	-0.01923	-0.01902	-0.01811	-0.01830	-0.01708	-0.01766	-0.01868	-0.02003	-0.02189	-0.02583	-0.03442	-0.03924
131072	-0.04312	-0.04464	-0.04599	-0.05278	-0.05181	-0.05619	-0.05583	-0.05531	-0.05021	-0.05008	-0.04989	-0.05733	-0.05880	-0.06924	-0.07953	-0.09263	
65536	-0.09099	-0.09641	-0.10306	-0.10819	-0.11146	-0.11987	-0.11909	-0.11244	-0.11162	-0.10828	-0.10870	-0.11886	-0.12696	-0.13999	-0.16120		
32768	-0.16258	-0.16894	-0.18060	-0.19724	-0.20088	-0.21093	-0.20967	-0.20117	-0.19707	-0.19110	-0.19182	-0.20963	-0.22779	-0.25696			
16384	-0.25537	-0.26632	-0.29125	-0.30884	-0.31661	-0.33487	-0.33130	-0.32465	-0.30888	-0.30583	-0.30893	-0.33541	-0.36389				
8192	-0.38457	-0.39647	-0.42345	-0.46157	-0.47196	-0.49013	-0.48127	-0.47803	-0.46190	-0.45032	-0.45100	-0.49773					
4096	-0.50506	-0.52562	-0.55465	-0.59410	-0.60221	-0.63512	-0.62139	-0.61058	-0.59736	-0.59169	-0.60547						
2048	-0.61689	-0.64701	-0.69174	-0.72737	-0.73959	-0.77322	-0.77052	-0.74614	-0.73042	-0.72853							
1024	-0.68629	-0.73547	-0.77104	-0.82550	-0.81759	-0.84964	-0.84838	-0.81006	-0.82746								
512	-0.73609	-0.77288	-0.81239	-0.85409	-0.86333	-0.87646	-0.87708	-0.86726									
256	-0.72222	-0.76456	-0.79096	-0.82544	-0.81544	-0.85246	-0.87246										
128	-0.66465	-0.67704	-0.68806	-0.72346	-0.73146	-0.78073											
64	-0.66840	-0.69874	-0.71546	-0.74832	-0.75184												
32	-0.68499	-0.70474	-0.73383	-0.79098													
16	-0.77116	-0.81068	-0.84538														
8	-0.90844	-0.97831															
4	-1.13681																

(a) $\mathcal{P}^{\mathcal{C}}$ for Scene: [**Torus**] Algorithm: [**PT**] Metric: [**VIF**] True GT: [**BDPT @ 524288 spp**].

BDPT	2	4	8	16	32	64	128	256	512	1024	2048	4096	8192	16384	32768	65536	131072
GT (BDPT)																	
262144	-0.01256	-0.01488	-0.01679	-0.01636	-0.01800	-0.01674	-0.01929	-0.01888	-0.01749	-0.01787	-0.01877	-0.01713	-0.02047	-0.02253	-0.02632	-0.03290	-0.03963
131072	-0.04417	-0.04111	-0.04753	-0.04843	-0.05206	-0.05248	-0.05886	-0.05459	-0.05127	-0.05107	-0.05256	-0.05433	-0.05965	-0.06566	-0.07589	-0.09373	
65536	-0.08749	-0.09545	-0.09786	-0.10504	-0.11372	-0.11358	-0.11902	-0.11357	-0.11197	-0.10571	-0.11103	-0.11000	-0.12216	-0.13959	-0.16027		
32768	-0.15775	-0.17004	-0.17554	-0.18979	-0.19567	-0.19975	-0.20854	-0.20659	-0.19055	-0.19412	-0.20137	-0.20931	-0.22371	-0.24771			
16384	-0.25252	-0.26754	-0.28283	-0.29854	-0.31300	-0.32610	-0.33005	-0.31950	-0.31452	-0.31485	-0.31340	-0.33065	-0.35256				
8192	-0.37415	-0.39089	-0.41845	-0.44423	-0.45499	-0.47606	-0.48995	-0.46610	-0.45778	-0.45247	-0.45353	-0.48616					
4096	-0.48883	-0.52241	-0.54298	-0.57667	-0.59239	-0.61478	-0.62140	-0.61310	-0.59675	-0.58802	-0.60040						
2048	-0.60731	-0.64094	-0.66715	-0.70379	-0.73245	-0.75459	-0.76207	-0.73667	-0.73382	-0.73663							
1024	-0.68428	-0.72418	-0.76141	-0.79658	-0.81573	-0.83074	-0.83789	-0.81061	-0.81695								
512	-0.72025	-0.76479	-0.79136	-0.82381	-0.84471	-0.86186	-0.86735	-0.85823									
256	-0.70881	-0.75287	-0.77072	-0.79182	-0.81853	-0.84985	-0.83479										
128	-0.64671	-0.67944	-0.68988	-0.70824	-0.72143	-0.76663											
64	-0.66213	-0.68442	-0.68833	-0.71897	-0.74176												
32	-0.67837	-0.68986	-0.72437	-0.75545													
16	-0.74648	-0.79487	-0.83218														
8	-0.88514	-0.94475															
4	-1.11208																

(b) $\mathcal{P}^{\mathcal{C}}$ for Scene: [**Torus**] Algorithm: [**BDPT**] Metric: [**VIF**] True GT: [**BDPT @ 524288 spp**].

PSSMLT	2	4	8	16	32	64	128	256	512	1024	2048	4096	8192	16384
GT (BDPT)														
262144	-0.01426	-0.01525	-0.01460	-0.01445	-0.01769	-0.01802	-0.01950	-0.01944	-0.02153	-0.02382	-0.02430	-0.02642	-0.03123	-0.03338
131072	-0.04931	-0.04776	-0.05003	-0.04930	-0.05179	-0.05460	-0.05779	-0.05982	-0.05918	-0.06494	-0.06956	-0.07570	-0.08678	-0.09505
65536	-0.10058	-0.10164	-0.10999	-0.11111	-0.11629	-0.11879	-0.11924	-0.12283	-0.12590	-0.13453	-0.14328	-0.15470	-0.17442	-0.19262
32768	-0.17743	-0.18020	-0.18894	-0.18732	-0.19594	-0.20354	-0.21173	-0.21932	-0.22829	-0.24338	-0.25661	-0.27511	-0.30401	-0.32934
16384	-0.28087	-0.28826	-0.30765	-0.31073	-0.32022	-0.33363	-0.34399	-0.35014	-0.35941	-0.38075	-0.40211	-0.43182	-0.46895	
8192	-0.40770	-0.41934	-0.44157	-0.44691	-0.46197	-0.48512	-0.49766	-0.51143	-0.53390	-0.55104	-0.58656	-0.62520		
4096	-0.52245	-0.54757	-0.56972	-0.58181	-0.60392	-0.62260	-0.64646	-0.66299	-0.69112	-0.72010	-0.76135			
2048	-0.65742	-0.68208	-0.72376	-0.73527	-0.75458	-0.78266	-0.80310	-0.83611	-0.86171	-0.90474				
1024	-0.75240	-0.77879	-0.81575	-0.82601	-0.85883	-0.88890	-0.91623	-0.94358	-0.98391					
512	-0.82296	-0.83723	-0.87590	-0.87446	-0.90918	-0.94331	-0.98037	-1.01595						
256	-0.81342	-0.83334	-0.87882	-0.87809	-0.91207	-0.95973	-0.99421							
128	-0.75955	-0.76136	-0.80203	-0.80406	-0.85465	-0.90318								
64	-0.79956	-0.81520	-0.83936	-0.83709	-0.89990									
32	-0.84869	-0.85437	-0.91278	-0.90484										
16	-0.96222	-0.97442	-1.04223											
8	-1.14220	-1.16156												
4	-1.40585													

(c) $\mathcal{P}^{\mathcal{C}}$ for Scene: [**Torus**] Algorithm: [**PSSMLT**] Metric: [**VIF**] True GT: [**BDPT @ 524288 spp**].

MLT	2	4	8	16	32	64	128	256	512	1024	2048	4096	8192	16384
GT (BDPT)														
262144	-0.01715	-0.02124	-0.01694	-0.01964	-0.01964	-0.02230	-0.02584	-0.02675	-0.02890	-0.03047	-0.03286	-0.03390	-0.03750	-0.03506
131072	-0.05544	-0.05916	-0.05578	-0.06351	-0.06236	-0.06818	-0.07389	-0.07704	-0.08596	-0.09043	-0.09553	-0.09726	-0.10489	-0.10229
65536	-0.10779	-0.12374	-0.10969	-0.12281	-0.12674	-0.13846	-0.14755	-0.15255	-0.16932	-0.17669	-0.18296	-0.18882	-0.20003	-0.19852
32768	-0.19433	-0.21289	-0.19390	-0.21491	-0.22038	-0.24412	-0.26418	-0.27359	-0.29028	-0.30124	-0.31418	-0.31685	-0.33488	-0.33038
16384	-0.29958	-0.32860	-0.30157	-0.33192	-0.35033	-0.37550	-0.40306	-0.41217	-0.44202	-0.45831	-0.47343	-0.48057	-0.50017	
8192	-0.44919	-0.48153	-0.45158	-0.48579	-0.51190	-0.54961	-0.58228	-0.59735	-0.63180	-0.65174	-0.67084	-0.67609		
4096	-0.57858	-0.62191	-0.58520	-0.64590	-0.65552	-0.71534	-0.76103	-0.77917	-0.82169	-0.84284	-0.86144			
2048	-0.70001	-0.75815	-0.72273	-0.77703	-0.81853	-0.88029	-0.93200	-0.95185	-0.99468	-1.02091				
1024	-0.81471	-0.88718	-0.83662	-0.92447	-0.95307	-1.01484	-1.07637	-1.08384	-1.13253					
512	-0.86376	-0.94096	-0.89563	-0.98577	-1.01331	-1.08826	-1.15269	-1.17722						
256	-0.86592	-0.93877	-0.90138	-1.00380	-1.03251	-1.10681	-1.17916							
128	-0.78707	-0.88404	-0.84161	-0.94427	-0.97462	-1.03756								
64	-0.83215	-0.92896	-0.87604	-0.99667	-1.03303									
32	-0.87190	-0.99644	-0.92958	-1.05799										
16	-1.00803	-1.12785	-1.05605											
8	-1.23407	-1.36399												
4	-1.52339													

(d) $\mathcal{P}^{\mathcal{C}}$ for Scene: [**Torus**] Algorithm: [**MLT**] Metric: [**VIF**] True GT: [**BDPT @ 524288 spp**].

M-MLT	2	4	8	16	32	64	128	256	512	1024	2048	4096	8192	16384
GT (BDPT)														
262144	-0.02060	-0.01572	-0.01834	-0.01745	-0.01811	-0.02406	-0.02882	-0.02974	-0.03241	-0.03496	-0.03407	-0.03807	-0.03828	-0.04149
131072	-0.06407	-0.05088	-0.05641	-0.05746	-0.05512	-0.07029	-0.07532	-0.07689	-0.09026	-0.09601	-0.09898	-0.10290	-0.10940	-0.11337
65536	-0.12776	-0.10974	-0.11513	-0.12540	-0.12056	-0.14490	-0.15505	-0.15932	-0.17569	-0.18443	-0.18999	-0.19725	-0.20571	-0.21355
32768	-0.22383	-0.19477	-0.20626	-0.22029	-0.22161	-0.25656	-0.26680	-0.27222	-0.29974	-0.31278	-0.32467	-0.33282	-0.34593	-0.35628
16384	-0.35207	-0.30528	-0.31579	-0.33914	-0.33641	-0.39262	-0.41147	-0.41835	-0.45589	-0.48007	-0.48879	-0.50045	-0.52162	
8192	-0.50990	-0.45052	-0.46216	-0.49897	-0.49761	-0.56934	-0.58537	-0.60408	-0.65507	-0.66862	-0.69175	-0.70700		
4096	-0.65188	-0.58821	-0.62595	-0.65072	-0.64558	-0.74593	-0.76283	-0.78757	-0.83664	-0.86374	-0.88314			
2048	-0.78729	-0.71257	-0.75630	-0.81279	-0.81322	-0.90622	-0.93602	-0.96021	-1.02230	-1.04658				
1024	-0.89450	-0.82158	-0.90243	-0.94235	-0.92152	-1.06226	-1.07935	-1.10621	-1.15986					
512	-0.97064	-0.87968	-0.97776	-1.03273	-0.99037	-1.13189	-1.16832	-1.19166						
256	-0.95288	-0.87843	-0.99709	-1.04966	-1.01193	-1.15663	-1.20483							
128	-0.86918	-0.81153	-0.95810	-1.00917	-0.95736	-1.10684								
64	-0.93284	-0.88596	-1.01760	-1.04939	-1.02283									
32	-0.99932	-0.93180	-1.08832	-1.11244										
16	-1.16526	-1.07155	-1.21819											
8	-1.40867	-1.28227												
4	-1.71558													

(e) $\mathcal{P}^{\mathcal{C}}$ for Scene: [**Torus**] Algorithm: [**M-MLT**] Metric: [**VIF**] True GT: [**BDPT @ 524288 spp**].

ERPT	2	4	8	16	32	64	128	256	512	1024	2048	4096	8192	16384	32768	65536	131072
GT (BDPT)																	
262144	-0.02220	-0.02465	-0.02677	-0.02599	-0.02670	-0.02468	-0.02339	-0.02428	-0.02313	-0.02417	-0.02479	-0.02433	-0.02563	-0.02711	-0.02597	-0.02582	-0.02578
131072	-0.06679	-0.07146	-0.07676	-0.07577	-0.07126	-0.07068	-0.06898	-0.06630	-0.07033	-0.07174	-0.06939	-0.07421	-0.07508	-0.07449	-0.07319	-0.07414	
65536	-0.13768	-0.14576	-0.14908	-0.14229	-0.14709	-0.14343	-0.14276	-0.13830	-0.13952	-0.14199	-0.14591	-0.15006	-0.15113	-0.15484	-0.15157		
32768	-0.24090	-0.25134	-0.25312	-0.25207	-0.25430	-0.24757	-0.25116	-0.24575	-0.24745	-0.25234	-0.25683	-0.26809	-0.26785	-0.26392			
16384	-0.37561	-0.39081	-0.40136	-0.39271	-0.39580	-0.39242	-0.39311	-0.38703	-0.39364	-0.40006	-0.40774	-0.41640	-0.41790				
8192	-0.54008	-0.56324	-0.57627	-0.56345	-0.56800	-0.56878	-0.56603	-0.56386	-0.56847	-0.57764	-0.59363	-0.60307					
4096	-0.70118	-0.72612	-0.73986	-0.73588	-0.74342	-0.74399	-0.74568	-0.74864	-0.75276	-0.76543	-0.78243						
2048	-0.85643	-0.89313	-0.92013	-0.90788	-0.92966	-0.91476	-0.92228	-0.93067	-0.93419	-0.95442							
1024	-0.97404	-1.04056	-1.04134	-1.03938	-1.07459	-1.04874	-1.07175	-1.07461	-1.09823								
512	-1.02310	-1.08843	-1.10869	-1.12148	-1.14112	-1.13059	-1.15438	-1.16766									
256	-1.03642	-1.11712	-1.13107	-1.12404	-1.17249	-1.16563	-1.20815										
128	-0.94799	-1.02700	-1.05204	-1.07511	-1.12282	-1.12674											
64	-1.00067	-1.05817	-1.09781	-1.12579	-1.15574												
32	-1.05556	-1.13807	-1.16754	-1.19212													
16	-1.19658	-1.29788	-1.33194														
8	-1.42244	-1.52826															
4	-1.73215																

(f) $\mathcal{P}^{\mathcal{C}}$ for Scene: [**Torus**] Algorithm: [**ERPT**] Metric: [**VIF**] True GT: [**BDPT @ 524288 spp**].

M-ERPT	2	4	8	16	32	64	128	256	512	1024	2048	4096	8192	16384	32768	65536	131072
GT (BDPT)																	
262144	-0.02376	-0.02657	-0.02406	-0.02332	-0.02371	-0.02564	-0.02634	-0.02559	-0.02499	-0.02743	-0.02876	-0.02771	-0.02933	-0.02743	-0.02582	-0.02528	-0.02689
131072	-0.07133	-0.07815	-0.06607	-0.06656	-0.06852	-0.06853	-0.06963	-0.07599	-0.07577	-0.07157	-0.07293	-0.07328	-0.07472	-0.07769	-0.07454	-0.07416	
65536	-0.13633	-0.14596	-0.14159	-0.14478	-0.13855	-0.14136	-0.14411	-0.14657	-0.15096	-0.14864	-0.14955	-0.14890	-0.14996	-0.15494	-0.15018		
32768	-0.24056	-0.24859	-0.23786	-0.24851	-0.23797	-0.25012	-0.25646	-0.25822	-0.26326	-0.26313	-0.26685	-0.26751	-0.26899	-0.26922			
16384	-0.37021	-0.38188	-0.37989	-0.38739	-0.37157	-0.39289	-0.39995	-0.40383	-0.40845	-0.41149	-0.41759	-0.41650	-0.42336				
8192	-0.52529	-0.54467	-0.55133	-0.55869	-0.53584	-0.56730	-0.58011	-0.57928	-0.59556	-0.59516	-0.60502	-0.59389					
4096	-0.69080	-0.70822	-0.71015	-0.72859	-0.70657	-0.74752	-0.75544	-0.76096	-0.77541	-0.78098	-0.78411						
2048	-0.84288	-0.85965	-0.88006	-0.89861	-0.87805	-0.91994	-0.94052	-0.94165	-0.96360	-0.97440							
1024	-0.96839	-0.97793	-0.99335	-1.03312	-1.00974	-1.07473	-1.09612	-1.09298	-1.12091								
512	-1.01190	-1.02551	-1.05508	-1.10634	-1.08012	-1.14701	-1.17041	-1.19359									
256	-1.00765	-1.04951	-1.06374	-1.12457	-1.09987	-1.17765	-1.22008										
128	-0.92944	-0.96537	-0.99484	-1.05855	-1.04547	-1.13557											
64	-0.97036	-0.99340	-1.03424	-1.11341	-1.08118												
32	-1.00955	-1.07167	-1.11675	-1.18269													
16	-1.14808	-1.22300	-1.25833														
8	-1.36017	-1.43797															
4	-1.66148																

(g) $\mathcal{P}^{\mathcal{C}}$ for Scene: [**Torus**] Algorithm: [**M-ERPT**] Metric: [**VIF**] True GT: [**BDPT @ 524288 spp**].

Table 102

22.3 Veach Bidir

PT	2	4	8	16	32	64	128	256	512	1024	2048	4096	8192	16384	32768	65536	131072
GT (BDPT)																	
262144	-0.00009	-0.00026	-0.00044	-0.00017	-0.00024	-0.00054	-0.00019	-0.00045	-0.00022	-0.00036	-0.00039	-0.00004	-0.00057	-0.00042	-0.00041	-0.00064	-0.00087
131072	-0.00092	-0.00138	-0.00101	-0.00096	-0.00110	-0.00110	-0.00071	-0.00096	-0.00114	-0.00132	-0.00120	-0.00118	-0.00117	-0.00119	-0.00187	-0.00202	
65536	-0.00169	-0.00194	-0.00151	-0.00165	-0.00103	-0.00168	-0.00130	-0.00160	-0.00212	-0.00194	-0.00138	-0.00129	-0.00192	-0.00290	-0.00305		
32768	-0.00237	-0.00351	-0.00271	-0.00245	-0.00291	-0.00309	-0.00231	-0.00279	-0.00296	-0.00314	-0.00327	-0.00330	-0.00414	-0.00512			
16384	-0.00450	-0.00464	-0.00366	-0.00335	-0.00376	-0.00437	-0.00356	-0.00395	-0.00446	-0.00429	-0.00491	-0.00480	-0.00584				
8192	-0.00744	-0.00783	-0.00628	-0.00637	-0.00748	-0.00740	-0.00706	-0.00749	-0.00797	-0.00817	-0.00958	-0.01026					
4096	-0.01323	-0.01239	-0.01285	-0.01194	-0.01235	-0.01394	-0.01413	-0.01431	-0.01461	-0.01451	-0.01659						
2048	-0.02445	-0.02423	-0.02263	-0.02253	-0.02275	-0.02240	-0.02433	-0.02530	-0.02595	-0.02799							
1024	-0.04410	-0.04415	-0.04217	-0.04090	-0.04159	-0.04273	-0.04400	-0.04531	-0.04590								
512	-0.07650	-0.07710	-0.07559	-0.07868	-0.07609	-0.07826	-0.08016	-0.08359									
256	-0.13251	-0.12992	-0.13000	-0.12979	-0.12953	-0.13586	-0.13972										
128	-0.20870	-0.21026	-0.20189	-0.21144	-0.21333	-0.21832											
64	-0.30849	-0.30210	-0.30070	-0.30953	-0.31982												
32	-0.42656	-0.43705	-0.43289	-0.44770													
16	-0.56226	-0.58070	-0.58986														
8	-0.73012	-0.74781															
4	-0.91074																

(a) $\mathcal{P}^{\mathcal{C}}$ for Scene: [**Veach Bidir**] Algorithm: [**PT**] Metric: [**VIF**] True GT: [**BDPT @ 524288 spp**].

BDPT	2	4	8	16	32	64	128	256	512	1024	2048	4096	8192	16384	32768	65536	131072
GT (BDPT)																	
262144	-0.00021	-0.00018	-0.00022	-0.00029	-0.00036	-0.00039	-0.00049	-0.00052	-0.00041	-0.00045	-0.00065	-0.00100	-0.00049	-0.00045	-0.00041	-0.00051	-0.00076
131072	-0.00087	-0.00088	-0.00101	-0.00137	-0.00108	-0.00112	-0.00151	-0.00132	-0.00154	-0.00178	-0.00191	-0.00200	-0.00167	-0.00181	-0.00174	-0.00202	
65536	-0.00106	-0.00122	-0.00127	-0.00158	-0.00145	-0.00187	-0.00220	-0.00242	-0.00250	-0.00317	-0.00306	-0.00338	-0.00333	-0.00419	-0.00423		
32768	-0.00235	-0.00260	-0.00259	-0.00309	-0.00310	-0.00401	-0.00462	-0.00475	-0.00492	-0.00567	-0.00595	-0.00734	-0.00757	-0.00819			
16384	-0.00329	-0.00369	-0.00316	-0.00445	-0.00466	-0.00492	-0.00575	-0.00702	-0.00805	-0.00884	-0.00988	-0.01160	-0.01138				
8192	-0.00695	-0.00656	-0.00625	-0.00791	-0.00899	-0.01003	-0.01141	-0.01323	-0.01544	-0.01702	-0.01872	-0.02046					
4096	-0.01263	-0.01356	-0.01427	-0.01551	-0.01677	-0.01945	-0.02224	-0.02527	-0.02912	-0.03115	-0.03412						
2048	-0.02190	-0.02251	-0.02418	-0.02609	-0.02838	-0.03280	-0.03863	-0.04450	-0.04906	-0.05631							
1024	-0.04168	-0.04325	-0.04469	-0.04721	-0.05199	-0.06086	-0.06974	-0.08081	-0.09012								
512	-0.07437	-0.07743	-0.08023	-0.08722	-0.09462	-0.10851	-0.12655	-0.14540									
256	-0.12979	-0.13337	-0.14030	-0.15256	-0.16736	-0.18922	-0.21699										
128	-0.20817	-0.21726	-0.22934	-0.25202	-0.27353	-0.30876											
64	-0.31403	-0.32724	-0.34942	-0.38372	-0.42316												
32	-0.44521	-0.47490	-0.50830	-0.55799													
16	-0.60950	-0.64604	-0.70069														
8	-0.79494	-0.85686															
4	-1.01546																

(b) $\mathcal{P}^{\mathcal{C}}$ for Scene: [**Veach Bidir**] Algorithm: [**BDPT**] Metric: [**VIF**] True GT: [**BDPT @ 524288 spp**].

PSSMLT	2	4	8	16	32	64	128	256	512	1024	2048	4096	8192	16384
GT (BDPT)														
262144	-0.00006	-0.00020	-0.00016	-0.00014	-0.00019	-0.00023	-0.00049	-0.00049	-0.00054	-0.00063	-0.00061	-0.00060	-0.00051	-0.00067
131072	-0.00086	-0.00101	-0.00099	-0.00111	-0.00097	-0.00120	-0.00125	-0.00138	-0.00153	-0.00156	-0.00168	-0.00187	-0.00208	-0.00204
65536	-0.00138	-0.00123	-0.00161	-0.00155	-0.00160	-0.00202	-0.00249	-0.00297	-0.00297	-0.00319	-0.00315	-0.00339	-0.00380	-0.00413
32768	-0.00285	-0.00287	-0.00307	-0.00301	-0.00357	-0.00419	-0.00491	-0.00513	-0.00573	-0.00597	-0.00611	-0.00687	-0.00784	-0.00858
16384	-0.00414	-0.00393	-0.00412	-0.00423	-0.00457	-0.00521	-0.00647	-0.00741	-0.00889	-0.00994	-0.01078	-0.01111	-0.01252	
8192	-0.00732	-0.00784	-0.00798	-0.00792	-0.00905	-0.01038	-0.01201	-0.01362	-0.01569	-0.01715	-0.01861	-0.02019		
4096	-0.01295	-0.01370	-0.01409	-0.01503	-0.01726	-0.01923	-0.02241	-0.02486	-0.02863	-0.03162	-0.03463			
2048	-0.02451	-0.02457	-0.02533	-0.02708	-0.03013	-0.03419	-0.03879	-0.04462	-0.05168	-0.05671				
1024	-0.04384	-0.04540	-0.04634	-0.04863	-0.05358	-0.05932	-0.06824	-0.07695	-0.08843					
512	-0.07833	-0.08065	-0.08281	-0.08654	-0.09654	-0.10862	-0.12243	-0.13882						
256	-0.13637	-0.13956	-0.14630	-0.15602	-0.17129	-0.18945	-0.21079							
128	-0.22434	-0.22967	-0.23951	-0.25419	-0.27798	-0.30510								
64	-0.33024	-0.33835	-0.35700	-0.38146	-0.41797									
32	-0.47823	-0.50069	-0.52784	-0.56736										
16	-0.65261	-0.68356	-0.72629											
8	-0.85725	-0.90326												
4	-1.09519													

(c) $\mathcal{P}^{\mathcal{C}}$ for Scene: [**Veach Bidir**] Algorithm: [**PSSMLT**] Metric: [**VIF**] True GT: [**BDPT @ 524288 spp**].

MLT	2	4	8	16	32	64	128	256	512	1024	2048	4096	8192	16384
GT (BDPT)														
262144	-0.00026	-0.00019	-0.00045	-0.00051	-0.00033	-0.00025	-0.00025	-0.00032	-0.00036	-0.00023	-0.00030	-0.00035	-0.00042	-0.00043
131072	-0.00096	-0.00080	-0.00078	-0.00075	-0.00071	-0.00090	-0.00089	-0.00101	-0.00111	-0.00117	-0.00098	-0.00115	-0.00134	-0.00152
65536	-0.00082	-0.00070	-0.00098	-0.00097	-0.00108	-0.00134	-0.00161	-0.00166	-0.00231	-0.00229	-0.00224	-0.00276	-0.00291	-0.00302
32768	-0.00322	-0.00298	-0.00271	-0.00242	-0.00273	-0.00281	-0.00321	-0.00345	-0.00383	-0.00426	-0.00450	-0.00511	-0.00548	-0.00619
16384	-0.00424	-0.00451	-0.00417	-0.00376	-0.00386	-0.00434	-0.00437	-0.00529	-0.00566	-0.00640	-0.00763	-0.00854	-0.00923	
8192	-0.00900	-0.00846	-0.00766	-0.00808	-0.00848	-0.00905	-0.00886	-0.01012	-0.01111	-0.01274	-0.01442	-0.01606		
4096	-0.01377	-0.01378	-0.01333	-0.01336	-0.01436	-0.01588	-0.01729	-0.01849	-0.02100	-0.02410	-0.02697			
2048	-0.02373	-0.02380	-0.02400	-0.02453	-0.02603	-0.02725	-0.02906	-0.03277	-0.03709	-0.04245				
1024	-0.04791	-0.04558	-0.04382	-0.04496	-0.04650	-0.04925	-0.05250	-0.05763	-0.06472					
512	-0.08010	-0.08026	-0.07934	-0.08072	-0.08574	-0.09014	-0.09673	-0.10702						
256	-0.14321	-0.14051	-0.13848	-0.13828	-0.14446	-0.15293	-0.16258							
128	-0.22377	-0.22235	-0.22599	-0.22774	-0.23615	-0.25024								
64	-0.31992	-0.32243	-0.33239	-0.34467	-0.35807									
32	-0.46578	-0.46776	-0.47855	-0.49361										
16	-0.62450	-0.62863	-0.64890											
8	-0.80093	-0.81586												
4	-0.99874													

(d) $\mathcal{P}^{\mathcal{C}}$ for Scene: [**Veach Bidir**] Algorithm: [**MLT**] Metric: [**VIF**] True GT: [**BDPT @ 524288 spp**].

M-MLT	2	4	8	16	32	64	128	256	512	1024	2048	4096	8192	16384
GT (BDPT)														
262144	-0.00041	-0.00035	-0.00013	-0.00021	-0.00037	-0.00046	-0.00012	-0.00015	-0.00015	-0.00039	-0.00062	-0.00067	-0.00046	-0.00053
131072	-0.00098	-0.00103	-0.00073	-0.00063	-0.00094	-0.00113	-0.00117	-0.00127	-0.00118	-0.00123	-0.00153	-0.00146	-0.00171	-0.00174
65536	-0.00206	-0.00173	-0.00145	-0.00134	-0.00144	-0.00150	-0.00161	-0.00155	-0.00165	-0.00193	-0.00283	-0.00274	-0.00313	-0.00327
32768	-0.00285	-0.00292	-0.00246	-0.00252	-0.00284	-0.00269	-0.00309	-0.00354	-0.00406	-0.00511	-0.00560	-0.00606	-0.00631	-0.00669
16384	-0.00343	-0.00472	-0.00422	-0.00403	-0.00394	-0.00449	-0.00453	-0.00511	-0.00575	-0.00630	-0.00773	-0.00878	-0.00951	
8192	-0.00720	-0.00824	-0.00756	-0.00764	-0.00824	-0.00814	-0.00827	-0.00985	-0.01128	-0.01326	-0.01491	-0.01668		
4096	-0.01453	-0.01507	-0.01470	-0.01446	-0.01521	-0.01553	-0.01663	-0.01844	-0.02095	-0.02380	-0.02796			
2048	-0.02566	-0.02584	-0.02448	-0.02415	-0.02539	-0.02671	-0.02938	-0.03218	-0.03642	-0.04319				
1024	-0.04666	-0.04456	-0.04707	-0.04672	-0.04959	-0.04996	-0.05285	-0.05826	-0.06669					
512	-0.08318	-0.08242	-0.08300	-0.08499	-0.08644	-0.09136	-0.09654	-0.10788						
256	-0.13988	-0.13682	-0.13911	-0.14341	-0.14889	-0.15625	-0.16675							
128	-0.22643	-0.21850	-0.22404	-0.22758	-0.23864	-0.25455								
64	-0.32523	-0.32526	-0.33324	-0.34584	-0.36071									
32	-0.46933	-0.46447	-0.47926	-0.49461										
16	-0.62397	-0.62244	-0.64822											
8	-0.80200	-0.81790												
4	-1.00518													

(e) $\mathcal{P}^{\mathcal{C}}$ for Scene: [**Veach Bidir**] Algorithm: [**M-MLT**] Metric: [**VIF**] True GT: [**BDPT @ 524288 spp**].

ERPT	2	4	8	16	32	64	128	256	512	1024	2048	4096	8192	16384	32768	65536	131072
GT (BDPT)																	
262144	-0.00014	-0.00038	-0.00018	-0.00041	-0.00023	-0.00029	-0.00011	-0.00048	-0.00026	-0.00043	-0.00050	-0.00075	-0.00017	-0.00079	-0.00055	-0.00034	-0.00049
131072	-0.00105	-0.00090	-0.00113	-0.00130	-0.00114	-0.00095	-0.00105	-0.00101	-0.00130	-0.00117	-0.00099	-0.00132	-0.00134	-0.00157	-0.00148	-0.00126	
65536	-0.00177	-0.00160	-0.00193	-0.00204	-0.00173	-0.00180	-0.00192	-0.00213	-0.00265	-0.00262	-0.00279	-0.00240	-0.00283	-0.00257	-0.00250		
32768	-0.00365	-0.00336	-0.00375	-0.00327	-0.00336	-0.00372	-0.00370	-0.00334	-0.00390	-0.00432	-0.00433	-0.00451	-0.00464	-0.00448			
16384	-0.00529	-0.00498	-0.00486	-0.00530	-0.00505	-0.00575	-0.00533	-0.00644	-0.00706	-0.00584	-0.00626	-0.00786	-0.00691				
8192	-0.00902	-0.00939	-0.00919	-0.00969	-0.00948	-0.01052	-0.01010	-0.00928	-0.01084	-0.01088	-0.01166	-0.01254					
4096	-0.01824	-0.01690	-0.01824	-0.01822	-0.01814	-0.01836	-0.01838	-0.01811	-0.02107	-0.01901	-0.01929						
2048	-0.03088	-0.03129	-0.02955	-0.03142	-0.03032	-0.03089	-0.03159	-0.03240	-0.03407	-0.03301							
1024	-0.05394	-0.05542	-0.05398	-0.05503	-0.05457	-0.05851	-0.05476	-0.05743	-0.05899								
512	-0.09746	-0.09772	-0.09770	-0.09993	-0.09937	-0.10450	-0.10141	-0.10114									
256	-0.16918	-0.16870	-0.17019	-0.17127	-0.17161	-0.17354	-0.17282										
128	-0.27783	-0.27439	-0.27777	-0.27549	-0.27741	-0.27954											
64	-0.41739	-0.41277	-0.41417	-0.41382	-0.41882												
32	-0.60836	-0.60644	-0.60741	-0.61426													
16	-0.83294	-0.83341	-0.83545														
8	-1.10943	-1.10578															
4	-1.41870																

(f) $\mathcal{P}^{\mathcal{C}}$ for Scene: [**Veach Bidir**] Algorithm: [**ERPT**] Metric: [**VIF**] True GT: [**BDPT @ 524288 spp**].

M-ERPT	2	4	8	16	32	64	128	256	512	1024	2048	4096	8192	16384	32768	65536	131072
GT (BDPT)																	
262144	-0.00032	-0.00009	-0.00026	-0.00024	-0.00031	-0.00049	-0.00062	-0.00025	-0.00075	-0.00063	-0.00042	-0.00011	-0.00050	-0.00054	-0.00043	-0.00071	-0.00007
131072	-0.00112	-0.00128	-0.00139	-0.00116	-0.00119	-0.00135	-0.00118	-0.00126	-0.00146	-0.00150	-0.00129	-0.00093	-0.00115	-0.00129	-0.00123	-0.00147	
65536	-0.00155	-0.00156	-0.00189	-0.00196	-0.00185	-0.00212	-0.00236	-0.00230	-0.00244	-0.00255	-0.00293	-0.00209	-0.00272	-0.00256	-0.00217		
32768	-0.00306	-0.00376	-0.00386	-0.00344	-0.00352	-0.00396	-0.00463	-0.00381	-0.00402	-0.00439	-0.00467	-0.00426	-0.00489	-0.00428			
16384	-0.00536	-0.00474	-0.00599	-0.00557	-0.00451	-0.00586	-0.00585	-0.00664	-0.00617	-0.00750	-0.00766	-0.00699	-0.00790				
8192	-0.01007	-0.00917	-0.01074	-0.00985	-0.00966	-0.00986	-0.01163	-0.01048	-0.01109	-0.01138	-0.01233	-0.01155					
4096	-0.01776	-0.01774	-0.01958	-0.01836	-0.01705	-0.01922	-0.01896	-0.02037	-0.02034	-0.02051	-0.02088						
2048	-0.02912	-0.02900	-0.03291	-0.03263	-0.03189	-0.03240	-0.03154	-0.03270	-0.03180	-0.03457							
1024	-0.05536	-0.05337	-0.05887	-0.05469	-0.05390	-0.05804	-0.06057	-0.05810	-0.05933								
512	-0.09898	-0.09507	-0.10218	-0.10271	-0.09964	-0.10290	-0.10674	-0.10349									
256	-0.16612	-0.16773	-0.17350	-0.17592	-0.17150	-0.17577	-0.17473										
128	-0.27430	-0.27110	-0.28094	-0.27818	-0.27881	-0.27945											
64	-0.41085	-0.41115	-0.42263	-0.42240	-0.42223												
32	-0.59771	-0.59790	-0.60848	-0.60657													
16	-0.82529	-0.82056	-0.83774														
8	-1.09030	-1.09223															
4	-1.40384																

(g) $\mathcal{P}^{\mathcal{C}}$ for Scene: [**Veach Bidir**] Algorithm: [**M-ERPT**] Metric: [**VIF**] True GT: [**BDPT @ 524288 spp**].

Table 103

22.4 Veach Door

PT	2	4	8	16	32	64	128	256	512	1024	2048	4096	8192	16384	32768	65536	131072
GT (BDPT)																	
262144	-0.00367	-0.00248	-0.00229	-0.00244	-0.00268	-0.00202	-0.00223	-0.00231	-0.00202	-0.00220	-0.00217	-0.00216	-0.00254	-0.00283	-0.00314	-0.00440	-0.00421
131072	-0.00551	-0.00867	-0.00582	-0.00591	-0.00618	-0.00492	-0.00594	-0.00564	-0.00565	-0.00557	-0.00590	-0.00639	-0.00689	-0.00785	-0.00944	-0.01116	
65536	-0.02020	-0.01447	-0.01294	-0.01137	-0.01248	-0.01175	-0.01216	-0.01247	-0.01267	-0.01276	-0.01385	-0.01423	-0.01608	-0.01828	-0.02103		
32768	-0.03677	-0.03243	-0.02566	-0.02564	-0.02552	-0.02514	-0.02577	-0.02648	-0.02599	-0.02686	-0.02857	-0.03068	-0.03333	-0.03809			
16384	-0.06022	-0.05128	-0.04897	-0.04829	-0.04611	-0.04592	-0.04710	-0.04749	-0.04895	-0.05065	-0.05266	-0.05680	-0.06352				
8192	-0.09727	-0.08582	-0.08234	-0.07939	-0.07846	-0.07956	-0.08057	-0.08070	-0.08414	-0.08711	-0.09285	-0.10062					
4096	-0.14710	-0.13637	-0.12933	-0.12928	-0.12811	-0.12716	-0.12848	-0.13237	-0.13658	-0.14380	-0.15224						
2048	-0.21069	-0.19773	-0.18831	-0.18778	-0.18685	-0.19030	-0.19190	-0.19872	-0.20805	-0.21906							
1024	-0.28921	-0.26818	-0.26025	-0.26233	-0.26447	-0.26707	-0.27720	-0.28919	-0.30174								
512	-0.37247	-0.35869	-0.35110	-0.35514	-0.36057	-0.36443	-0.37524	-0.39637									
256	-0.45631	-0.43955	-0.44104	-0.44118	-0.46238	-0.47754	-0.49784										
128	-0.56105	-0.55567	-0.55118	-0.55847	-0.58311	-0.60839											
64	-0.67562	-0.66936	-0.66240	-0.69095	-0.72160												
32	-0.78983	-0.79612	-0.80233	-0.84385													
16	-0.88719	-0.90493	-0.95965														
8	-1.04450	-1.07028															
4	-1.20939																

(a) $\mathcal{P}^{\mathcal{C}}$ for Scene: [**Veach Door**] Algorithm: [**PT**] Metric: [**VIF**] True GT: [**BDPT @ 524288 spp**].

BDPT	2	4	8	16	32	64	128	256	512	1024	2048	4096	8192	16384	32768	65536	131072
GT (BDPT)																	
262144	-0.00193	-0.00189	-0.00229	-0.00182	-0.00204	-0.00193	-0.00187	-0.00188	-0.00239	-0.00233	-0.00297	-0.00323	-0.00371	-0.00420	-0.00512	-0.00550	-0.00580
131072	-0.00612	-0.00574	-0.00472	-0.00511	-0.00546	-0.00512	-0.00566	-0.00584	-0.00667	-0.00686	-0.00761	-0.00898	-0.01066	-0.01287	-0.01429	-0.01580	
65536	-0.01292	-0.01213	-0.01132	-0.01207	-0.01234	-0.01172	-0.01216	-0.01288	-0.01382	-0.01545	-0.01777	-0.02101	-0.02445	-0.02866	-0.03288		
32768	-0.02774	-0.02576	-0.02428	-0.02649	-0.02517	-0.02564	-0.02598	-0.02775	-0.02995	-0.03232	-0.03740	-0.04287	-0.05203	-0.05922			
16384	-0.04880	-0.04843	-0.04798	-0.04670	-0.04685	-0.04817	-0.04951	-0.05115	-0.05521	-0.05980	-0.06856	-0.08016	-0.09356				
8192	-0.08533	-0.07929	-0.07799	-0.07870	-0.08030	-0.08175	-0.08450	-0.08948	-0.09663	-0.10644	-0.12078	-0.14249					
4096	-0.12649	-0.12853	-0.12765	-0.12840	-0.12968	-0.13301	-0.13678	-0.14447	-0.15862	-0.17587	-0.20419						
2048	-0.18907	-0.18880	-0.18864	-0.18909	-0.19094	-0.19868	-0.20896	-0.22360	-0.24265	-0.27502							
1024	-0.26317	-0.26334	-0.26609	-0.26833	-0.27514	-0.28706	-0.30455	-0.32935	-0.36462								
512	-0.35585	-0.34924	-0.35707	-0.36218	-0.37030	-0.39467	-0.42203	-0.46270									
256	-0.44145	-0.44476	-0.44947	-0.46799	-0.48613	-0.52536	-0.56571										
128	-0.55900	-0.55032	-0.56620	-0.59516	-0.63116	-0.68199											
64	-0.66464	-0.66856	-0.69642	-0.74250	-0.79109												
32	-0.78049	-0.80711	-0.85543	-0.92009													
16	-0.93296	-0.98030	-1.03538														
8	-1.09055	-1.15537															
4	-1.30753																

(b) $\mathcal{P}^{\mathcal{C}}$ for Scene: [**Veach Door**] Algorithm: [**BDPT**] Metric: [**VIF**] True GT: [**BDPT @ 524288 spp**].

PSSMLT	2	4	8	16	32	64	128	256	512	1024	2048	4096	8192	16384
GT (BDPT)														
262144	-0.00396	-0.00333	-0.00207	-0.00190	-0.00224	-0.00247	-0.00248	-0.00243	-0.00271	-0.00296	-0.00320	-0.00374	-0.00438	-0.00510
131072	-0.00773	-0.00766	-0.00665	-0.00606	-0.00640	-0.00655	-0.00668	-0.00654	-0.00715	-0.00791	-0.00947	-0.01134	-0.01311	-0.01471
65536	-0.01745	-0.01574	-0.01405	-0.01327	-0.01367	-0.01384	-0.01430	-0.01489	-0.01657	-0.01867	-0.02169	-0.02535	-0.02882	-0.03248
32768	-0.03351	-0.03141	-0.02833	-0.02770	-0.02816	-0.02988	-0.03073	-0.03234	-0.03574	-0.03970	-0.04523	-0.05261	-0.05978	-0.06598
16384	-0.05704	-0.05315	-0.05181	-0.05090	-0.05135	-0.05240	-0.05511	-0.05808	-0.06397	-0.07186	-0.08297	-0.09542	-0.10764	
8192	-0.09447	-0.08934	-0.08678	-0.08606	-0.08771	-0.09010	-0.09440	-0.10096	-0.11154	-0.12675	-0.14523	-0.16563		
4096	-0.14504	-0.14035	-0.13656	-0.13704	-0.14076	-0.14482	-0.15346	-0.16672	-0.18486	-0.21164	-0.23899			
2048	-0.20888	-0.20471	-0.20362	-0.20477	-0.21065	-0.21985	-0.23426	-0.25415	-0.28526	-0.32343				
1024	-0.28951	-0.28899	-0.28680	-0.29386	-0.30378	-0.31912	-0.34533	-0.38200	-0.42754					
512	-0.38612	-0.38443	-0.39062	-0.40132	-0.41993	-0.44509	-0.48432	-0.53703						
256	-0.49129	-0.49141	-0.50039	-0.52289	-0.55709	-0.59531	-0.65076							
128	-0.61484	-0.62424	-0.64319	-0.67067	-0.71794	-0.77908								
64	-0.75945	-0.76754	-0.79718	-0.84682	-0.91312									
32	-0.90472	-0.94149	-0.99643	-1.05783										
16	-1.09402	-1.12977	-1.21147											
8	-1.30996	-1.37078												
4	-1.60404													

(c) $\mathcal{P}^{\mathcal{C}}$ for Scene: [**Veach Door**] Algorithm: [**PSSMLT**] Metric: [**VIF**] True GT: [**BDPT @ 524288 spp**].

MLT	2	4	8	16	32	64	128	256	512	1024	2048	4096	8192	16384
GT (BDPT)														
262144	-0.00196	-0.00220	-0.00213	-0.00226	-0.00232	-0.00255	-0.00228	-0.00268	-0.00262	-0.00308	-0.00353	-0.00431	-0.00516	-0.00523
131072	-0.00586	-0.00619	-0.00601	-0.00560	-0.00592	-0.00625	-0.00644	-0.00706	-0.00784	-0.00859	-0.01081	-0.01233	-0.01394	-0.01541
65536	-0.01380	-0.01392	-0.01323	-0.01310	-0.01335	-0.01444	-0.01449	-0.01607	-0.01800	-0.02044	-0.02317	-0.02749	-0.03062	-0.03410
32768	-0.02691	-0.02762	-0.02728	-0.02615	-0.02770	-0.02854	-0.03055	-0.03277	-0.03691	-0.04187	-0.04832	-0.05692	-0.06360	-0.06844
16384	-0.04952	-0.05042	-0.04901	-0.05084	-0.05060	-0.05267	-0.05603	-0.06145	-0.06872	-0.07922	-0.09127	-0.10257	-0.11351	
8192	-0.08573	-0.08404	-0.08381	-0.08576	-0.08695	-0.09180	-0.09838	-0.10717	-0.12062	-0.13900	-0.15739	-0.17772		
4096	-0.13403	-0.13431	-0.13452	-0.13634	-0.14116	-0.14933	-0.15821	-0.17634	-0.19823	-0.22603	-0.25698			
2048	-0.19823	-0.19746	-0.19917	-0.20741	-0.21214	-0.22764	-0.24069	-0.27026	-0.30732	-0.34809				
1024	-0.27781	-0.28145	-0.28545	-0.29498	-0.31129	-0.33705	-0.35815	-0.40185	-0.45201					
512	-0.36919	-0.37373	-0.38422	-0.40443	-0.42814	-0.46532	-0.50677	-0.56549						
256	-0.47684	-0.48841	-0.50289	-0.52951	-0.56510	-0.62256	-0.67911							
128	-0.58968	-0.61231	-0.64107	-0.68597	-0.74583	-0.82138								
64	-0.73204	-0.76315	-0.80265	-0.86645	-0.94616									
32	-0.87659	-0.93394	-0.99254	-1.08910										
16	-1.04713	-1.13118	-1.22295											
8	-1.26977	-1.39231												
4	-1.58447													

(d) $\mathcal{P}^{\mathcal{C}}$ for Scene: [**Veach Door**] Algorithm: [**MLT**] Metric: [**VIF**] True GT: [**BDPT @ 524288 spp**].

M-MLT	2	4	8	16	32	64	128	256	512	1024	2048	4096	8192	16384
GT (BDPT)														
262144	-0.00271	-0.00181	-0.00266	-0.00228	-0.00175	-0.00223	-0.00232	-0.00257	-0.00311	-0.00330	-0.00397	-0.00437	-0.00476	-0.00537
131072	-0.00787	-0.00551	-0.00545	-0.00621	-0.00601	-0.00585	-0.00674	-0.00708	-0.00839	-0.00928	-0.01112	-0.01231	-0.01384	-0.01492
65536	-0.01524	-0.01276	-0.01270	-0.01288	-0.01318	-0.01436	-0.01468	-0.01647	-0.01830	-0.02134	-0.02443	-0.02789	-0.03183	-0.03438
32768	-0.02937	-0.02749	-0.02740	-0.02754	-0.02752	-0.02882	-0.03157	-0.03308	-0.03680	-0.04262	-0.05018	-0.05675	-0.06360	-0.06872
16384	-0.05185	-0.04890	-0.05000	-0.05085	-0.05144	-0.05276	-0.05624	-0.06079	-0.06836	-0.07873	-0.09083	-0.10269	-0.11447	
8192	-0.08588	-0.08340	-0.08451	-0.08694	-0.08942	-0.09290	-0.09653	-0.10670	-0.12078	-0.13737	-0.15828	-0.17825		
4096	-0.13661	-0.13240	-0.13423	-0.13813	-0.14247	-0.14971	-0.16240	-0.17527	-0.19858	-0.22652	-0.25784			
2048	-0.19480	-0.19450	-0.20138	-0.20482	-0.21574	-0.22792	-0.24640	-0.27364	-0.30606	-0.34749				
1024	-0.27968	-0.27688	-0.28547	-0.29373	-0.31249	-0.33417	-0.36211	-0.40339	-0.45496					
512	-0.36819	-0.37139	-0.38679	-0.40490	-0.43004	-0.46446	-0.51156	-0.56661						
256	-0.47360	-0.47553	-0.49761	-0.52692	-0.57147	-0.62372	-0.68185							
128	-0.59197	-0.60982	-0.64177	-0.69273	-0.74545	-0.81971								
64	-0.72691	-0.74450	-0.79607	-0.86360	-0.94756									
32	-0.87973	-0.92539	-1.00129	-1.09222										
16	-1.04792	-1.11671	-1.23034											
8	-1.27696	-1.36454												
4	-1.57807													

(e) $\mathcal{P}^{\mathcal{C}}$ for Scene: [**Veach Door**] Algorithm: [**M-MLT**] Metric: [**VIF**] True GT: [**BDPT @ 524288 spp**].

ERPT	2	4	8	16	32	64	128	256	512	1024	2048	4096	8192	16384	32768	65536	131072
GT (BDPT)																	
262144	-0.00238	-0.00224	-0.00245	-0.00244	-0.00266	-0.00217	-0.00278	-0.00259	-0.00260	-0.00256	-0.00239	-0.00210	-0.00266	-0.00257	-0.00250	-0.00262	-0.00252
131072	-0.00681	-0.00676	-0.00692	-0.00682	-0.00713	-0.00686	-0.00672	-0.00695	-0.00715	-0.00701	-0.00662	-0.00679	-0.00705	-0.00666	-0.00671	-0.00700	
65536	-0.01588	-0.01512	-0.01554	-0.01536	-0.01514	-0.01534	-0.01499	-0.01568	-0.01619	-0.01516	-0.01463	-0.01558	-0.01555	-0.01514	-0.01539		
32768	-0.03253	-0.03172	-0.03207	-0.03205	-0.03201	-0.03135	-0.03127	-0.03144	-0.03201	-0.03128	-0.03074	-0.03207	-0.03182	-0.03144			
16384	-0.05770	-0.05838	-0.05768	-0.05805	-0.05797	-0.05830	-0.05782	-0.05868	-0.05744	-0.05886	-0.05767	-0.05874	-0.05837				
8192	-0.10139	-0.09951	-0.10131	-0.10073	-0.10077	-0.10192	-0.10090	-0.10180	-0.10146	-0.10235	-0.10103	-0.10001					
4096	-0.16626	-0.16661	-0.16385	-0.16513	-0.16371	-0.16482	-0.16453	-0.16556	-0.16693	-0.16362	-0.16565						
2048	-0.25675	-0.25490	-0.25323	-0.25423	-0.25295	-0.25215	-0.25315	-0.25360	-0.25474	-0.25470							
1024	-0.37885	-0.37749	-0.37417	-0.37708	-0.37402	-0.37564	-0.37508	-0.37473	-0.37926								
512	-0.53061	-0.52879	-0.53008	-0.52773	-0.52737	-0.52966	-0.52757	-0.52944									
256	-0.71289	-0.70754	-0.70496	-0.70610	-0.70779	-0.70980	-0.71231										
128	-0.92762	-0.92693	-0.92687	-0.93014	-0.93220	-0.92879											
64	-1.17268	-1.16182	-1.16261	-1.17058	-1.16728												
32	-1.44034	-1.44247	-1.44341	-1.44428													
16	-1.73919	-1.74373	-1.73871														
8	-2.09441	-2.09284															
4	-2.49507																

(f) $\mathcal{P}^{\mathcal{C}}$ for Scene: [**Veach Door**] Algorithm: [**ERPT**] Metric: [**VIF**] True GT: [**BDPT @ 524288 spp**].

M-ERPT	2	4	8	16	32	64	128	256	512	1024	2048	4096	8192	16384	32768	65536	131072
GT (BDPT)																	
262144	-0.00205	-0.00263	-0.00232	-0.00225	-0.00240	-0.00256	-0.00280	-0.00234	-0.00247	-0.00266	-0.00209	-0.00234	-0.00228	-0.00217	-0.00280	-0.00252	-0.00244
131072	-0.00669	-0.00660	-0.00670	-0.00676	-0.00596	-0.00694	-0.00691	-0.00677	-0.00644	-0.00724	-0.00629	-0.00689	-0.00685	-0.00650	-0.00674	-0.00680	
65536	-0.01487	-0.01500	-0.01445	-0.01476	-0.01516	-0.01495	-0.01521	-0.01520	-0.01449	-0.01484	-0.01454	-0.01519	-0.01563	-0.01470	-0.01513		
32768	-0.03031	-0.03103	-0.03055	-0.03084	-0.03176	-0.03135	-0.03054	-0.03081	-0.03099	-0.03085	-0.03042	-0.03104	-0.03136	-0.03082			
16384	-0.05639	-0.05681	-0.05698	-0.05771	-0.05760	-0.05723	-0.05641	-0.05729	-0.05657	-0.05752	-0.05621	-0.05773	-0.05760				
8192	-0.09841	-0.09883	-0.09804	-0.09890	-0.09934	-0.09848	-0.09922	-0.09979	-0.09904	-0.10002	-0.10023	-0.10058					
4096	-0.16155	-0.16165	-0.16116	-0.16230	-0.16252	-0.16180	-0.16238	-0.16250	-0.16299	-0.16185	-0.16173						
2048	-0.24727	-0.24826	-0.24962	-0.24826	-0.24807	-0.25121	-0.24956	-0.25123	-0.24874	-0.24922							
1024	-0.36263	-0.36716	-0.36875	-0.36684	-0.37002	-0.36915	-0.36915	-0.37143	-0.36660								
512	-0.50957	-0.51246	-0.51393	-0.51758	-0.51781	-0.51982	-0.51866	-0.51722									
256	-0.69220	-0.69120	-0.69250	-0.69417	-0.69259	-0.69491	-0.69614										
128	-0.89720	-0.89797	-0.90707	-0.91071	-0.91218	-0.90881											
64	-1.12753	-1.14135	-1.14395	-1.15135	-1.15000												
32	-1.40597	-1.41528	-1.41702	-1.42817													
16	-1.69609	-1.70286	-1.71688														
8	-2.04472	-2.06351															
4	-2.44762																

(g) $\mathcal{P}^{\mathcal{C}}$ for Scene: [**Veach Door**] Algorithm: [**M-ERPT**] Metric: [**VIF**] True GT: [**BDPT @ 524288 spp**].

Table 104

22.5 Sponza

PT	2	4	8	16	32	64	128	256	512	1024	2048	4096	8192
GT (PT)													
16384	-0.00817	-0.00971	-0.00958	-0.00987	-0.00946	-0.01063	-0.01019	-0.01395	-0.01435	-0.01653	-0.02121	-0.02500	-0.02814
8192	-0.02223	-0.02473	-0.02511	-0.02491	-0.02742	-0.02839	-0.02943	-0.03394	-0.04139	-0.04754	-0.05696	-0.06762	
4096	-0.05287	-0.05099	-0.05312	-0.05403	-0.05648	-0.05933	-0.06286	-0.07414	-0.08690	-0.10011	-0.11977		
2048	-0.09284	-0.08934	-0.09006	-0.09453	-0.09923	-0.10639	-0.11530	-0.13348	-0.15471	-0.18176			
1024	-0.14804	-0.15162	-0.15001	-0.15471	-0.16632	-0.17892	-0.19721	-0.22396	-0.26239				
512	-0.22520	-0.22768	-0.23034	-0.24121	-0.25658	-0.27266	-0.30844	-0.35111					
256	-0.31248	-0.31768	-0.32933	-0.34624	-0.37223	-0.40273	-0.45493						
128	-0.42833	-0.42748	-0.44443	-0.47415	-0.52316	-0.58379							
64	-0.54499	-0.56521	-0.59578	-0.64608	-0.70790								
32	-0.68257	-0.70885	-0.76050	-0.83391									
16	-0.84458	-0.88436	-0.95772										
8	-1.03705	-1.09519											
4	-1.22021												

(a) $\mathcal{P}^{\mathcal{C}}$ for Scene: [**Sponza**] Algorithm: [**PT**] Metric: [**VIF**] True GT: [**PT @ 32768 spp**].

BDPT	2	4	8	16	32	64	128	256	512	1024	2048	4096	8192
GT (PT)													
16384	-0.01143	-0.01067	-0.01049	-0.00996	-0.00913	-0.00991	-0.01085	-0.01238	-0.01383	-0.01736	-0.01868	-0.02322	-0.02718
8192	-0.02697	-0.02488	-0.02565	-0.02471	-0.02647	-0.02746	-0.03001	-0.03385	-0.03769	-0.04547	-0.05338	-0.06531	
4096	-0.05173	-0.05094	-0.05165	-0.05336	-0.05670	-0.05797	-0.06247	-0.06929	-0.07992	-0.09555	-0.11455		
2048	-0.09488	-0.09145	-0.09248	-0.09398	-0.09807	-0.10363	-0.11444	-0.12761	-0.14771	-0.17611			
1024	-0.15151	-0.14567	-0.15163	-0.15518	-0.16361	-0.17511	-0.18861	-0.21432	-0.25012				
512	-0.22020	-0.22803	-0.22884	-0.24088	-0.25298	-0.26751	-0.29606	-0.34164					
256	-0.31514	-0.31841	-0.32651	-0.34147	-0.36194	-0.39464	-0.44135						
128	-0.43036	-0.43293	-0.45317	-0.47027	-0.50855	-0.56631							
64	-0.54026	-0.56020	-0.58753	-0.62574	-0.68661								
32	-0.68606	-0.70997	-0.75322	-0.82281									
16	-0.85780	-0.87628	-0.94736										
8	-1.02203	-1.09056											
4	-1.21845												

(b) $\mathcal{P}^{\mathcal{C}}$ for Scene: [**Sponza**] Algorithm: [**BDPT**] Metric: [**VIF**] True GT: [**PT @ 32768 spp**].

PSSMLT	2	4	8	16	32	64	128	256	512	1024	2048	4096	8192
GT (PT)													
16384	-0.00992	-0.01060	-0.00986	-0.00987	-0.00970	-0.00883	-0.01076	-0.01173	-0.01346	-0.01528	-0.01766	-0.02170	-0.02467
8192	-0.02785	-0.02680	-0.02663	-0.02777	-0.02720	-0.02799	-0.03081	-0.03317	-0.03741	-0.04197	-0.05026	-0.05859	
4096	-0.05724	-0.05533	-0.05565	-0.05584	-0.05778	-0.05925	-0.06482	-0.07016	-0.07799	-0.08942	-0.10372		
2048	-0.09766	-0.09581	-0.09708	-0.09904	-0.10270	-0.10579	-0.11595	-0.12666	-0.14269	-0.16151			
1024	-0.16010	-0.15818	-0.15904	-0.16431	-0.16657	-0.17768	-0.19309	-0.21178	-0.23963				
512	-0.24388	-0.24329	-0.24380	-0.24945	-0.26171	-0.27639	-0.30379	-0.33669					
256	-0.34557	-0.34286	-0.34583	-0.36042	-0.37971	-0.40893	-0.44815						
128	-0.48001	-0.47895	-0.48060	-0.50013	-0.53635	-0.58011							
64	-0.61007	-0.61660	-0.63531	-0.66667	-0.71422								
32	-0.77631	-0.78685	-0.80846	-0.85872									
16	-0.96446	-0.99290	-1.04520										
8	-1.17193	-1.21578											
4	-1.39363												

(c) $\mathcal{P}^{\mathcal{C}}$ for Scene: [**Sponza**] Algorithm: [**PSSMLT**] Metric: [**VIF**] True GT: [**PT @ 32768 spp**].

MLT	2	4	8	16	32	64	128	256	512	1024	2048	4096	8192
GT (PT)													
16384	-0.01038	-0.01108	-0.00983	-0.01017	-0.01084	-0.01008	-0.01143	-0.01497	-0.01617	-0.02008	-0.02377	-0.02631	-0.02795
8192	-0.02501	-0.02451	-0.02751	-0.02651	-0.02871	-0.03005	-0.03473	-0.04046	-0.04772	-0.05656	-0.06511	-0.07058	
4096	-0.05308	-0.05487	-0.05834	-0.05819	-0.06128	-0.06511	-0.07280	-0.08451	-0.09884	-0.11618	-0.12948		
2048	-0.09615	-0.09837	-0.09955	-0.10284	-0.11240	-0.11981	-0.13407	-0.15316	-0.17801	-0.20483			
1024	-0.15860	-0.16233	-0.16441	-0.17158	-0.18585	-0.20309	-0.22771	-0.25716	-0.29679				
512	-0.23445	-0.24426	-0.24971	-0.26289	-0.28508	-0.31147	-0.35258	-0.39645					
256	-0.33827	-0.35297	-0.36451	-0.39175	-0.41804	-0.46431	-0.51659						
128	-0.45469	-0.47753	-0.49967	-0.53663	-0.58846	-0.65245							
64	-0.58895	-0.62154	-0.66606	-0.71584	-0.78960								
32	-0.74511	-0.79890	-0.85894	-0.92766									
16	-0.92248	-1.00993	-1.09788										
8	-1.12420	-1.23630											
4	-1.38726												

(d) $\mathcal{P}^{\mathcal{C}}$ for Scene: [**Sponza**] Algorithm: [**MLT**] Metric: [**VIF**] True GT: [**PT @ 32768 spp**].

M-MLT	2	4	8	16	32	64	128	256	512	1024	2048	4096	8192
GT (PT)													
16384	-0.01215	-0.01006	-0.01070	-0.00934	-0.01079	-0.01122	-0.01331	-0.01504	-0.01637	-0.02105	-0.02340	-0.02621	-0.02821
8192	-0.02544	-0.02482	-0.02685	-0.02754	-0.02911	-0.03208	-0.03696	-0.04064	-0.04944	-0.05526	-0.06381	-0.06996	
4096	-0.05552	-0.05435	-0.05559	-0.05647	-0.06228	-0.06791	-0.07534	-0.08773	-0.10055	-0.11607	-0.13087		
2048	-0.09414	-0.09620	-0.10201	-0.10493	-0.11168	-0.12170	-0.13663	-0.15557	-0.18083	-0.20304			
1024	-0.15959	-0.16117	-0.16974	-0.17380	-0.18578	-0.20593	-0.22825	-0.26040	-0.30062				
512	-0.23757	-0.24266	-0.25094	-0.26756	-0.28987	-0.31662	-0.35794	-0.40126					
256	-0.34277	-0.34374	-0.37019	-0.38825	-0.41786	-0.46545	-0.52058						
128	-0.46065	-0.47495	-0.50577	-0.54028	-0.59088	-0.65865							
64	-0.60196	-0.63074	-0.67175	-0.72825	-0.79635								
32	-0.76787	-0.80016	-0.86316	-0.94742									
16	-0.96525	-1.01577	-1.10912										
8	-1.16204	-1.24361											
4	-1.41971												

(e) $\mathcal{P}^{\mathcal{C}}$ for Scene: [**Sponza**] Algorithm: [**M-MLT**] Metric: [**VIF**] True GT: [**PT @ 32768 spp**].

ERPT	2	4	8	16	32	64	128	256	512	1024	2048	4096	8192
GT (PT)													
16384	-0.01518	-0.01349	-0.01288	-0.01273	-0.01442	-0.01464	-0.01337	-0.01375	-0.01427	-0.01428	-0.01397	-0.01381	-0.01304
8192	-0.03849	-0.03752	-0.03759	-0.03748	-0.03908	-0.03865	-0.03670	-0.03741	-0.03823	-0.03709	-0.03742	-0.03804	
4096	-0.07924	-0.07815	-0.07696	-0.07809	-0.07854	-0.08035	-0.07779	-0.07840	-0.07898	-0.07929	-0.07900		
2048	-0.14132	-0.14178	-0.14218	-0.14117	-0.14398	-0.14495	-0.14060	-0.14276	-0.14502	-0.14240			
1024	-0.23789	-0.24078	-0.24127	-0.23795	-0.24055	-0.24214	-0.24122	-0.23685	-0.23999				
512	-0.36652	-0.37051	-0.36824	-0.36990	-0.37096	-0.37614	-0.37155	-0.36985					
256	-0.53275	-0.54120	-0.54172	-0.53979	-0.54409	-0.54463	-0.53997						
128	-0.74828	-0.75846	-0.75443	-0.76189	-0.76021	-0.76074							
64	-0.98809	-1.00334	-1.01032	-1.00962	-1.00409								
32	-1.26748	-1.27674	-1.27888	-1.28152									
16	-1.58918	-1.60717	-1.60396										
8	-1.91148	-1.92905											
4	-2.27001												

(f) $\mathcal{P}^{\mathcal{C}}$ for Scene: [**Sponza**] Algorithm: [**ERPT**] Metric: [**VIF**] True GT: [**PT @ 32768 spp**].

M-ERPT	2	4	8	16	32	64	128	256	512	1024	2048	4096	8192
GT (PT)													
16384	-0.01360	-0.01306	-0.01319	-0.01397	-0.01395	-0.01247	-0.01275	-0.01179	-0.01369	-0.01283	-0.01337	-0.01312	-0.01274
8192	-0.03776	-0.03717	-0.03711	-0.03712	-0.03704	-0.03772	-0.03649	-0.03691	-0.03548	-0.03677	-0.03741	-0.03644	
4096	-0.07887	-0.07787	-0.07951	-0.07807	-0.07868	-0.07720	-0.07747	-0.07569	-0.07490	-0.07710	-0.07659		
2048	-0.13945	-0.13883	-0.14055	-0.13912	-0.14304	-0.14019	-0.13785	-0.13996	-0.14122	-0.13879			
1024	-0.23703	-0.23013	-0.23533	-0.23383	-0.23460	-0.23489	-0.23665	-0.23705	-0.23540				
512	-0.36214	-0.36284	-0.36506	-0.36358	-0.36810	-0.36571	-0.36447	-0.36648					
256	-0.52265	-0.53249	-0.53344	-0.52918	-0.53746	-0.53248	-0.52944						
128	-0.73798	-0.74636	-0.74883	-0.74320	-0.74839	-0.75111							
64	-0.98664	-0.98283	-0.99359	-0.99471	-0.99018								
32	-1.25098	-1.26615	-1.26490	-1.27263									
16	-1.57364	-1.58298	-1.59164										
8	-1.89853	-1.91402											
4	-2.25631												

(g) $\mathcal{P}^{\mathcal{C}}$ for Scene: [**Sponza**] Algorithm: [**M-ERPT**] Metric: [**VIF**] True GT: [**PT @ 32768 spp**].

Table 105
